# Multiantigen pan-sarbecovirus DNA vaccines generate protective T cell immune responses

**DOI:** 10.1172/jci.insight.172488

**Published:** 2023-11-08

**Authors:** Jeroen van Bergen, Marcel G.M. Camps, Iris N. Pardieck, Dominique Veerkamp, Wing Yan Leung, Anouk A. Leijs, Sebenzile K. Myeni, Marjolein Kikkert, Ramon Arens, Gerben C. Zondag, Ferry Ossendorp

**Affiliations:** 1 Immunetune BV, Leiden, Netherlands.; 2 Department of Immunology, Leiden University Medical Centre, Leiden, Netherlands.; 3 Synvolux BV, Leiden, Netherlands.; 4 Department of Medical Microbiology, Leiden University Medical Centre, Leiden, Netherlands.

**Keywords:** COVID-19, Vaccines, MHC class 1, MHC class 2, T cells

## Abstract

SARS-CoV-2 is the third zoonotic coronavirus to cause a major outbreak in humans in recent years, and many more SARS-like coronaviruses with pandemic potential are circulating in several animal species. Vaccines inducing T cell immunity against broadly conserved viral antigens may protect against hospitalization and death caused by outbreaks of such viruses. We report the design and preclinical testing of 2 T cell–based pan-sarbecovirus vaccines, based on conserved regions within viral proteins of sarbecovirus isolates of human and other carrier animals, like bats and pangolins. One vaccine (CoVAX_ORF1ab) encoded antigens derived from nonstructural proteins, and the other (CoVAX_MNS) encoded antigens from structural proteins. Both multiantigen DNA vaccines contained a large set of antigens shared across sarbecoviruses and were rich in predicted and experimentally validated human T cell epitopes. In mice, the multiantigen vaccines generated both CD8^+^ and CD4^+^ T cell responses to shared epitopes. Upon encounter of full-length spike antigen, CoVAX_MNS-induced CD4^+^ T cells were responsible for accelerated CD8^+^ T cell and IgG Ab responses specific to the incoming spike, irrespective of its sarbecovirus origin. Finally, both vaccines elicited partial protection against a lethal SARS-CoV-2 challenge in human angiotensin-converting enzyme 2–transgenic mice. These results support clinical testing of these universal sarbecovirus vaccines for pandemic preparedness.

## Graphical abstract

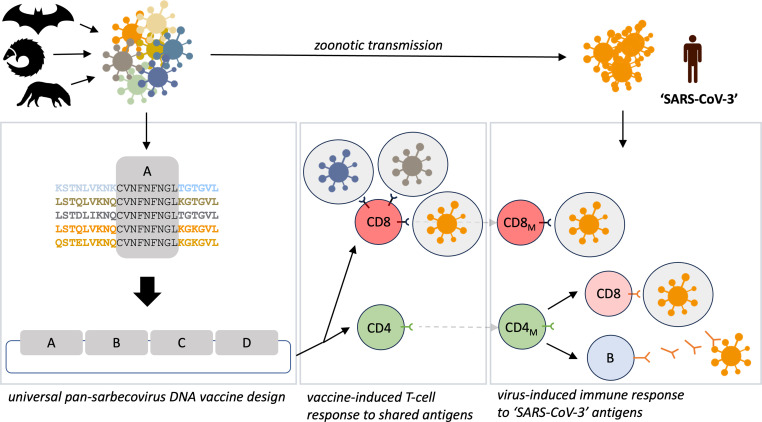

## Introduction

Within a year after the start of the SARS-CoV-2 outbreak, the first COVID-19 vaccines received Emergency Use Authorization from the FDA. The mRNA- and adenovirus-based vaccines were highly effective against infection, infection spreading, disease, and death caused by the original SARS-CoV-2 Wuhan-Hu-1 strain and its D614G variant (1–4). The approved mRNA and adenoviral vaccines all encode the spike (S) protein and are aimed primarily at generating neutralizing Abs. However, viral adaptation to its new human host resulted in S protein alterations, primarily in the receptor-binding domain (RBD). These resulted in better binding to the human angiotensin-converting enzyme 2 (hACE2) receptor and often in reduced Ab neutralization of variants of concern (VOCs) by Abs raised by these vaccines (5–8). As a result, immunity induced by these vaccines now offers limited protection against infection and dissemination of the Omicron VOCs, although vaccines are still highly effective against hospitalization and death, particularly after a booster dose (9–12).

The SARS-CoV-2 pandemic represents the third major outbreak of a zoonotic coronavirus in recent years. Before SARS-CoV-2, MERS-CoV, with an estimated 35% mortality rate, caused an outbreak in several countries in the Middle East, Africa, and South Asia in 2012 (13). In 2002–2004, SARS-CoV (called SARS-CoV-1 hereafter, for clarity) killed approximately 9% of people with a confirmed infection (14). It is likely that common cold coronaviruses such as 229E, NL63, OC43, and HKU1 caused similar outbreaks when they first jumped the species barrier (15). Of these coronaviruses, SARS-CoV-1 and SARS-CoV-2, both from the *Sarbecovirus* subgenus, share the greatest sequence similarity, and both viruses rely on the ACE2 receptor to enter cells. Few Abs raised by SARS-CoV-1 infection or vaccination neutralize SARS-CoV-2, and the Abs raised by SARS-CoV-2 infection or vaccination have limited activity against SARS-CoV-1 (16–21). The current SARS-CoV-2 vaccines, therefore, are unlikely to be effective against a future zoonotic sarbecovirus outbreak (22), and such an outbreak may well cause many deaths before a specific vaccine becomes available.

T cells play a central role in antiviral immunity and can prevent severe infection in the absence of a detectable B cell Ab response (23–25). Although CD8^+^ T cells lyse virus-infected cells directly, thereby limiting viral replication and disease progression, CD4^+^ T cells are required for class-switched, high-affinity B cell responses and more effective, long-lasting CD8^+^ T cell responses. In addition to S-specific Abs, current COVID-19 vaccines also induce CD4^+^ and CD8^+^ T cell immunity against S, and their epitopes remain largely unaffected by VOC mutations (26–29). Vaccine-induced T rather than B cell immunity against S epitopes shared between SARS-CoV-2 Wuhan-Hu-1 and subsequent VOCs is likely to be key to protection against disease and death (30). Unlike effective antiviral B cells, which recognize a limited set of S epitopes focused on the highly variable RBD region, antiviral T cells can recognize epitopes in any viral gene, including highly conserved ones such as *ORF1ab* (31). Indeed, in unvaccinated individuals, reactivation of preexisting T cell responses against such shared epitopes correlates with Ab-independent protection from disease (32, 33). Similar observations by McMichael et al. (34–37) and others (38) support the notion that, in influenza infection, T cells recognizing conserved epitopes can reduce viral titers and disease severity.

Vaccines inducing T rather than B cell responses have been proposed as a solution to ever-changing viruses such as the influenza virus. Two universal T cell–based influenza vaccines, both synthetic peptide vaccines, have been in clinical trials (39): Flu-V (40–43) and FP-01.1 (44). In a placebo-controlled phase IIb trial, a single dose of the Flu-V vaccine significantly reduced the likelihood to develop influenza symptoms upon i.n. virus challenge in healthy adult and mostly White men (40). However, because this vaccine consisted of only 4 peptides of 20–32 aa each, selected for their ability to induce an HLA-A*0201–restricted T cell response (45), it is unlikely to generate a broad T cell response in most of the human population. Another conserved, synthetic, peptide influenza vaccine with larger numbers of sequences has shown promising results in mice and ferrets (46). In contrast to peptide vaccines, plasmid DNA vaccines are easy to produce and offer the possibility to encode artificial polypeptides consisting of many large, conserved antigens, connected by small linker sequences. Here, we designed and tested in mice the immunogenicity and effectiveness of such multiantigen DNA vaccines containing highly conserved antigens shared by sarbecoviruses circulating in humans, bats, pangolins, and civets.

## Results

### Antigen selection and vaccine design.

Shared sarbecovirus T cell antigens were selected from the structural membrane (M), nucleoprotein (N), or S or from the nonstructural ORF1ab proteins of the SARS-CoV-2 Wuhan-Hu-1 sequence (NC_045512.2), based on sequence conservation and HLA class I binding. To this end, the aa sequences of 16 sarbecoviruses (Supplemental Table 1; supplemental material available online with this article; https://doi.org/10.1172/jci.insight.172488DS1), together representing a cross section of all sarbecovirus branches (47, 48), were aligned with the corresponding Wuhan-Hu-1 sequences. Because a single, conservative aa substitution can greatly impair T cell recognition (49, 50), only sequences identical in all 16 sarbecoviruses were selected. In addition, NetMHCpan (51) was used to identify SARS-CoV-2 peptides predicted to bind at least 1 of the 12 most prominent *HLA-A* or *HLA-B* alleles, together covering about 85% of the global human population (52). This resulted in the selection of 17 M, N, and S (Supplemental Table 3) and 17 ORF1ab (Supplemental Table 4) conserved sequences, varying in length from 16 to 76 aa, with the collective potential to elicit broad T cell responses in most, if not all, of the human population.

**Figure 1:**
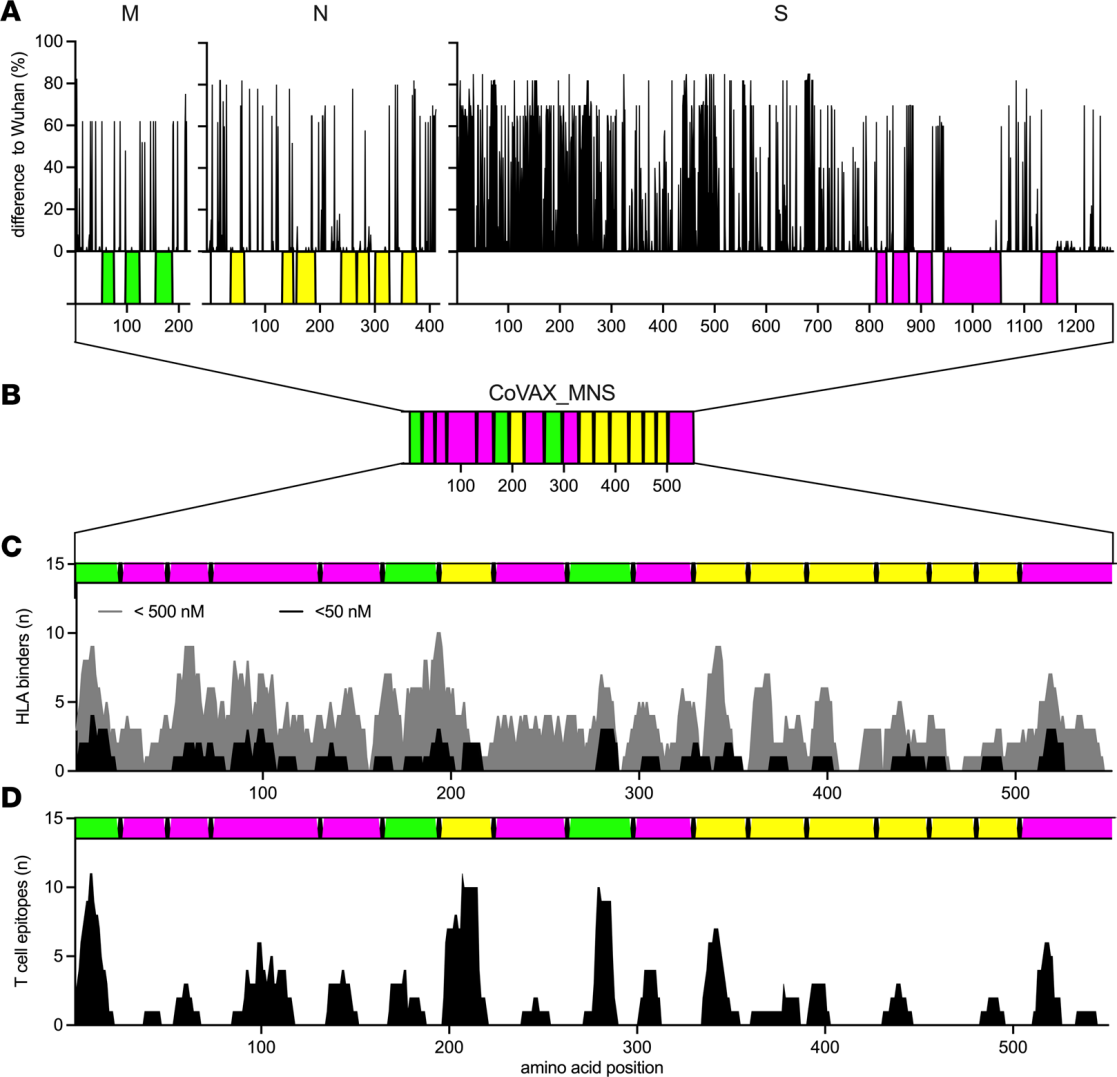
Antigen selection for T cell–based pan-sarbecovirus vaccine CoVAX_MNS. (**A**) Using the SARS-CoV-2 Wuhan-Hu-1 isolate (NC_045512.2) as the reference sequence, M, N, and S aa sequences from 41 sarbecoviruses (Supplemental Table 2) were aligned using Clustal Omega. For every Wuhan aa position in these proteins, the fraction of sarbecovirus sequences with a different aa at that position is plotted (difference to Wuhan). Full alignment results are presented in Supplemental Figures 1–3. (**B**) The selected conserved antigenic regions were incorporated into a multiantigen DNA vaccine in which these regions were separated by AAA spacers and reordered to minimize artificial junctional epitopes containing spacer-derived alanines. (**C** and **D**) Subsequently, the number of predicted HLA class I binders and experimentally validated CD8^+^ T cell epitopes present in the resulting vaccines were calculated using online tools. (**C**) First, peptides predicted to bind any of the most prominent HLA-A (A*01:01, A*02:01, A*03:01, A*11:01, A*23:01, A*24:02) or HLA-B (B*07:02, B*08:01, B*35:01, B*40:01, B*44:02, B*44:03) alleles (83) with affinities below 50 nM (black) or 500 nM (gray) were identified using MHCflurry (54). At every aa position, the number of predicted HLA-binding peptides to which this aa residue contributes is indicated (HLA binders). (**D**) Next, known human SARS-CoV-2 CD8^+^ T cell epitopes presented via the abovementioned HLA alleles were obtained from the IEDB database (55). For every aa position, the number of confirmed CD8^+^ T cell epitopes this aa residue contributes to is plotted (T cell epitopes).

**Figure 2:**
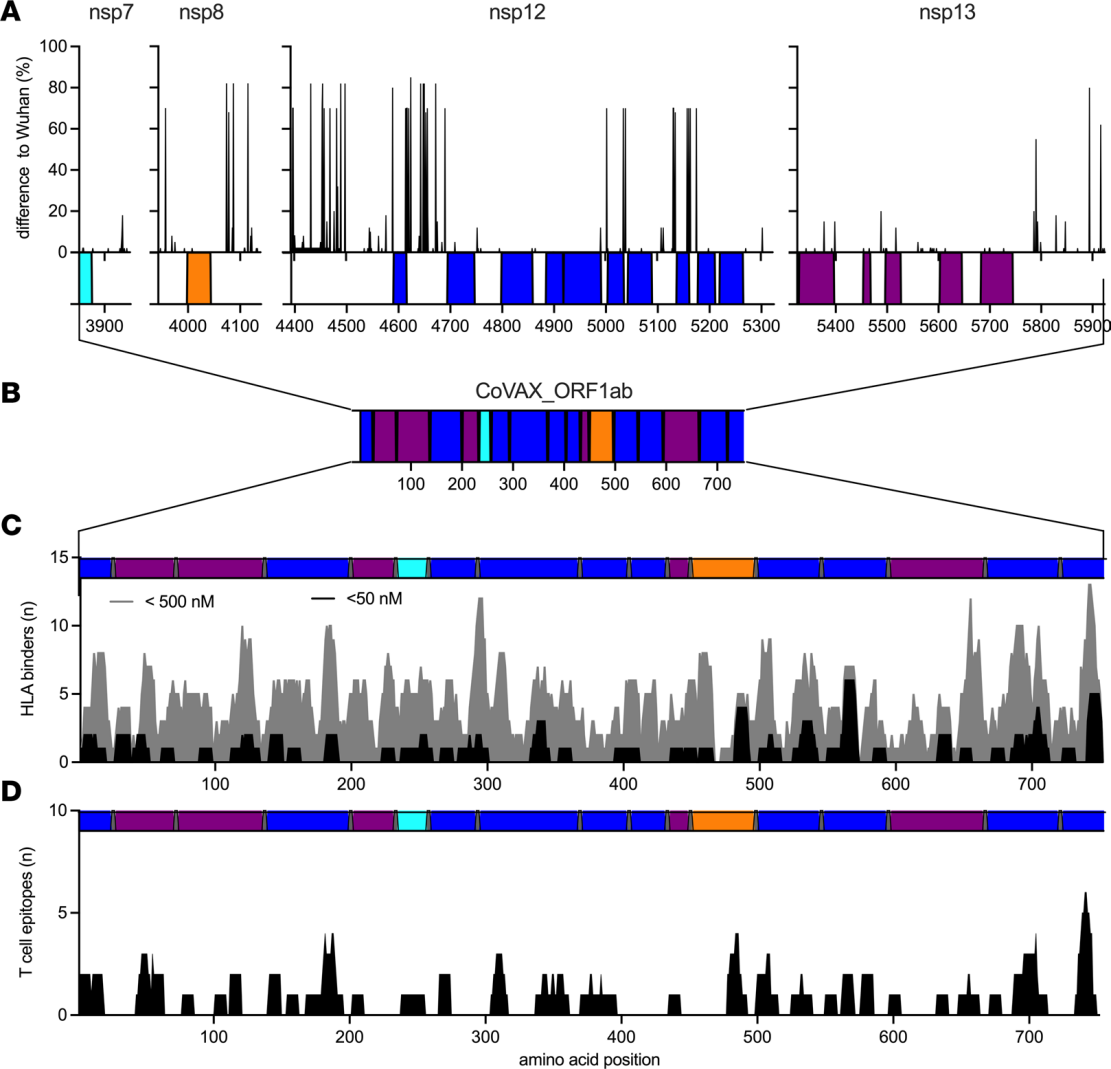
Antigen selection for T cell–based pan-sarbecovirus vaccine CoVAX_ORF1ab. (**A**) Using the SARS-CoV-2 Wuhan-Hu-1 isolate (NC_045512.2) as the reference sequence, ORF1ab aa sequences from 41 sarbecoviruses (Supplemental Table 2) were aligned using Clustal Omega. For every Wuhan aa position in these proteins, the fraction of sarbecovirus sequences with a different aa at that position is plotted (difference to Wuhan). For the full alignment, see Supplemental Figure 4. (**B**) The selected conserved antigenic regions were incorporated into a multiantigen DNA vaccine in which these regions were separated by AAA spacers and reordered to minimize artificial junctional epitopes containing spacer-derived alanines. (**C** and **D**) Subsequently, the number of predicted HLA class I binders and experimentally validated CD8^+^ T cell epitopes present in the resulting vaccines were calculated using online tools. (**C**) First, peptides predicted to bind any of the most prominent HLA-A (A*01:01, A*02:01, A*03:01, A*11:01, A*23:01, A*24:02) or HLA-B (B*07:02, B*08:01, B*35:01, B*40:01, B*44:02, B*44:03) alleles (83) with affinities below 50 nM (black) or 500 nM (gray) were identified using MHCflurry (54). At every aa position, the number of predicted HLA-binding peptides to which this aa residue contributes is indicated (HLA binders). (**D**) Next, known human SARS-CoV-2 CD8^+^ T cell epitopes presented via the abovementioned HLA alleles were obtained from the IEDB database (55). For every aa position, the number of confirmed CD8^+^ T cell epitopes this aa residue contributes to is plotted (T cell epitopes).

To verify the selection of vaccine antigens, we first examined their conservation among sarbecoviruses, using a larger set of sequences from 41 viruses (Supplemental Table 2), selected to represent the full diversity of the *Sarbecovirus* subgenus (53). Alignment of the M, N, S (Figure 1A), and ORF1ab antigen-containing sequences from nsp7, nsp8, nsp12, and nsp13 (Figure 2A) with Wuhan-Hu-1 verified that the selected antigens were, indeed, virtually identical across sarbecoviruses. This allowed the design of the CoVAX_MNS (Figure 1B) and CoVAX_ORF1ab (Figure 2B) polypeptides, in which the antigens were separated by triple-alanine (AAA) spacers to promote proteasomal cleavage and reordered to minimize the potential to generate artificial epitopes containing spacer alanines. Together with the fact that only small segments of the coronavirus proteins were included, this further reduced the risk of generating functional proteins. Next, we checked whether the polypeptides, indeed, contained HLA-binding peptides able to generate CD8^+^ T cell responses in humans. To this end, their sequences were analyzed by MHCflurry (54), an open-source tool with similar performance to NetMHC, to predict peptides binding the most prominent HLA class I alleles (52) (Figure 1C and Figure 2C). Finally, their CD8^+^ T cell epitope content was verified using lists of T cell epitopes, again using the same set of HLA class I alleles (52), from the Immune Epitope Database (IEDB) (55), experimentally verified by T cell assays using human blood samples (52) (Figure 1D and Figure 2D). Together, these analyses showed that the selected antigens were shared across sarbecoviruses and were rich in predicted and experimentally validated human T cell epitopes.

The selected and validated antigens were incorporated in 2 plasmid DNA vaccines, in which expression was driven by a strong CMV promoter: 1 DNA vaccine for ORF1ab (CoVAX_ORF1ab) and 1 for M, N, and S antigens (CoVAX_MNS). Two versions of each vaccine were produced, 1 lacking and 1 containing 3 C-terminal S–derived CD8^+^ T cell reporter antigens, to be able to check the immunogenicity of the vaccines in mice and humans.

### Pan-sarbecovirus DNA vaccines induce CD8^+^ T cell responses to shared sarbecovirus antigens.

**Figure 3:**
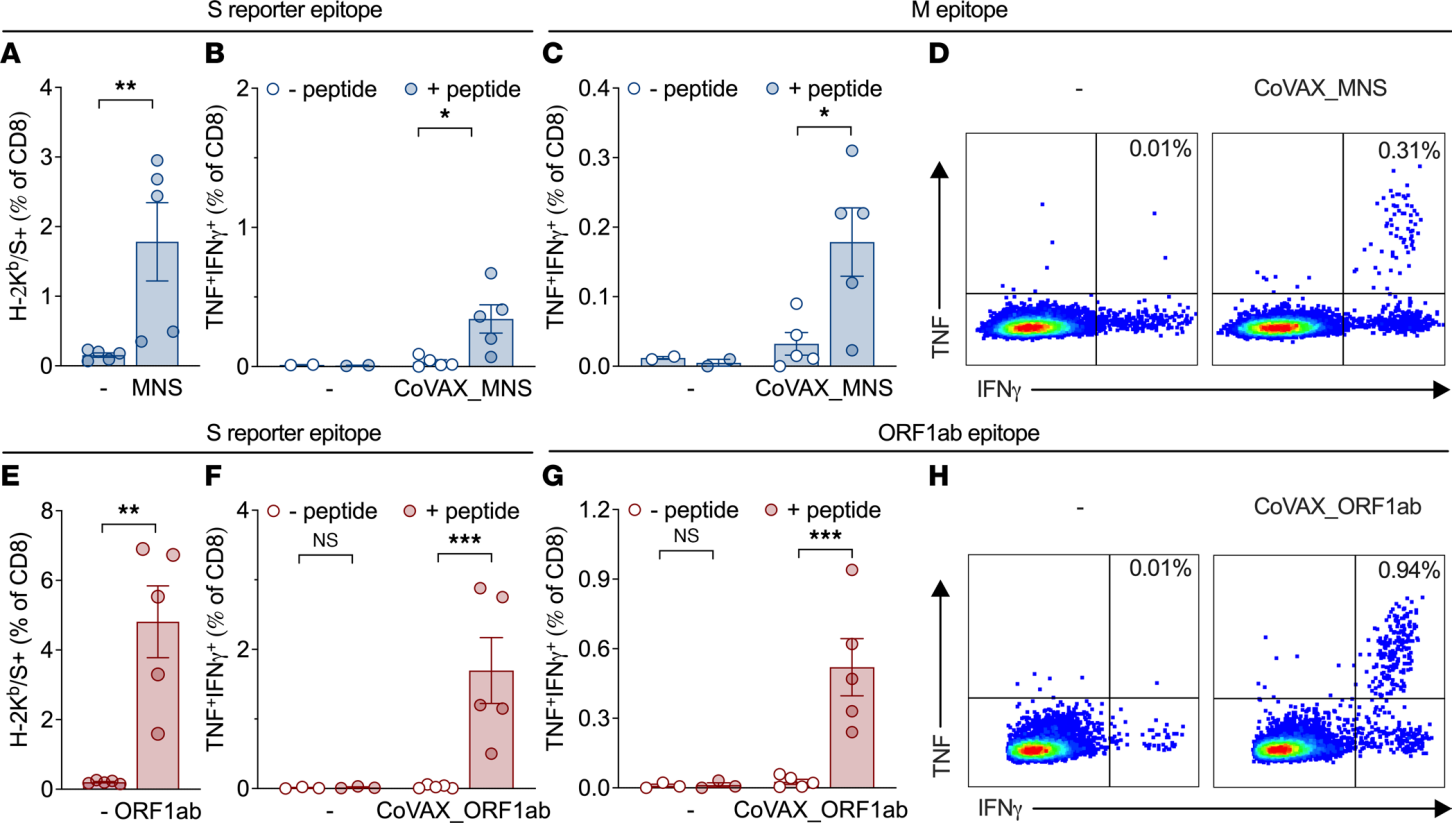
CoVAX_MNS and CoVAX_ORF1ab DNA vaccines generate CD8^+^ T cell responses to conserved antigens. C57BL/6 mice (5 mice/group) were vaccinated intradermally 3 times at 3-week intervals with (**A**–**D**) CoVAX_MNS or (**E**–**H**) CoVAX_ORF1ab, both including a C-terminal H-2K^b^–restricted S reporter epitope (VNFNFNGL). (**A** and **E**) Ten days after the third vaccination, CD8^+^ T cell responses to this H-2K^b^/S reporter epitope were measured in blood, using tetramers. (**B**–**D** and **F**–**H**) Eleven days after the third vaccination, isolated splenocytes were exposed to DCs (D1), then either peptide loaded (+ peptide) or not (– peptide), and CD8^+^ T cell cytokine responses were evaluated by ICS for IFN-γ and TNF. (**C** and **G**) Summarized responses to the S reporter epitope, a known H-2K^b^–restricted M epitope (RTLSYYKL), and a newly discovered H-2K^b^–restricted ORF1ab epitope (TGYHFREL) are shown, as well as (**D** and **H**) representative FACS plots gated on CD8^+^ T cells. (**A**–**C** and **E**–**G**) Dots represent individual mice; bars and whiskers indicate means and SEM. Tetramer responses were evaluated using a 2-tailed Mann-Whitney test and ICS responses by 2-way ANOVA using Holm-Šídák multiple comparisons test. For multiplicity-adjusted *P* values: *P ≤ 0.05; **P ≤ 0.01; ***P ≤ 0.002.

To test the immunogenicity of the pan-sarbecovirus DNA vaccines in a preclinical setting, C57BL/6 mice were vaccinated intradermally with the plasmid vaccines containing reporter antigens, and responses to an H-2K^b^-restricted reporter CD8^+^ T cell epitope were tracked in blood using tetramer staining. Both the CoVAX_MNS (Figure 3A) and the CoVAX_ORF1ab (Figure 3E) vaccines generated strong (>1% of CD8, on average) CD8^+^ T cell responses to this C-terminal reporter antigen, establishing expression of the full-length, vaccine-encoded, multiantigen proteins as well as their immunogenicity in this in vivo setting.

Even though the shared vaccine antigens were selected to contain human HLA class I–restricted T cell epitopes, the sheer size and number of antigens meant that they were also likely to contain H-2^b^–restricted CD8^+^ T cell epitopes, relevant in C57BL/6 mice. Indeed, CoVAX_MNS contained a known H-2K^b^–restricted M epitope (56), in response to which up to 0.3% of splenic CD8^+^ T cells from vaccinated, but not control, mice, produced TNF and IFN-γ (Figure 3, B–D). Thus far, no C57BL/6 ORF1ab epitopes have been described (57). Therefore, we evaluated CD8^+^ T cell responses to 11 candidate epitopes predicted to bind H-2K^b^ or H-2D^b^ in spleens of CoVAX_ORF1ab vaccinated mice (data not shown). Up to 0.9% of CD8^+^ T cells produced TNF and IFN-γ in response to 1 of these peptides (TGYHFREL), predicted to bind H-2K^b^ (Figure 3, F–H). Thus, both DNA vaccines were immunogenic in C57BL/6 mice and generated a pro-inflammatory CD8^+^ T cell response to at least 1 shared sarbecovirus epitope. To avoid interference of immunodominant responses to the reporter antigens with responses to the selected conserved antigens, vaccines lacking the reporter antigen unit were used from this point onward, unless otherwise indicated.

### CoVAX_MNS-induced CD4^+^ T cell responses improve CD8^+^ T cell and Ab responses upon exposure to SARS-CoV-2 S.

**Figure 4:**
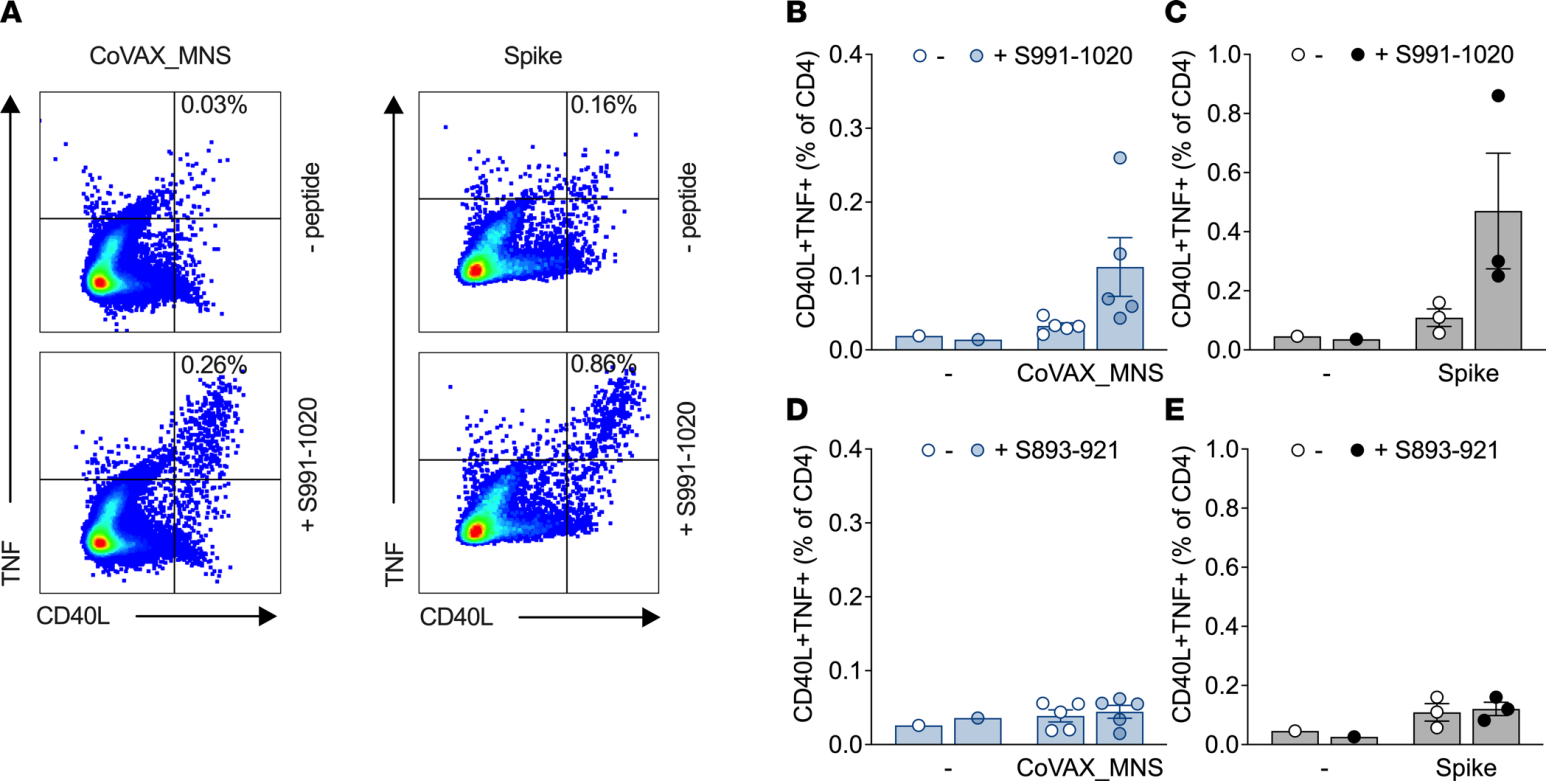
CoVAX_MNS induces a CD4^+^ T cell response to a conserved S antigen. C57BL/6 mice (5 mice/group) were vaccinated intradermally with CoVAX_MNS, from which the reporter antigens had been removed (“norep” in Supplemental Table 5), 3 times at 3-week intervals. Additional control mice (3 mice/group) were mock-vaccinated or vaccinated with DNA encoding SARS-CoV-2 Wuhan S (p393 or p422; see Supplemental Table 5). A week after the final vaccination, spleen cells were cultured for 7 days with peptide-loaded syngeneic DCs (D1) and another 2 days with IL-2, after which the cultured splenocytes were exposed for 6 hours to D1; preloaded (+ peptide), or not (– peptide), with shared sarbecovirus S peptides comprising (**A**–**C**) S residues 966–1020 (S991–1020) or (**D** and **E**) 893–921 (S893–921); and analyzed by ICS. Activated CD4^+^ T cells were identified by coexpression of CD40L and TNF after restimulation with these S peptides. (**A**) Representative dot plots for the S991–1020 peptide. (**B**–**E**) Summarized data.

First, we determined whether CoVAX_MNS elicited S-specific CD4^+^ T cell responses in C57BL/6 mice (Figure 4). Because CD4^+^ T cell responses tend not to be abundant (58) and, therefore, are poorly detectable directly ex vivo, spleen cells from vaccinated mice were cultured for 7 days with DCs loaded with a mixture of peptides before testing CD4^+^ T cell responses against individual peptides by intracellular cytokine staining (ICS). CD4^+^ T cells in bulk cultures from vaccinated, but not control, mice upregulated CD40L and produced TNF specifically in response to CoVAX_MNS-derived shared S peptide VQIDRLITGRLQSLQTYVTQQLIRAAEIRA (S991–1020; Figure 4, A and B), but not in response to S peptide ALQIPFAMQMAYRFNGIGVTQNVLYENQK (S893–921; Figure 4D), also contained in CoVAX_MNS. CD4^+^ T cells from control mice vaccinated with an S-encoding DNA vaccine also responded to the former (Figure 4, A and C), but not the latter (Figure 4E), peptide, suggesting that processing and presentation of CoVAX_MNS antigens to CD4^+^ T cells mirror those of full-length S.

**Figure 5:**
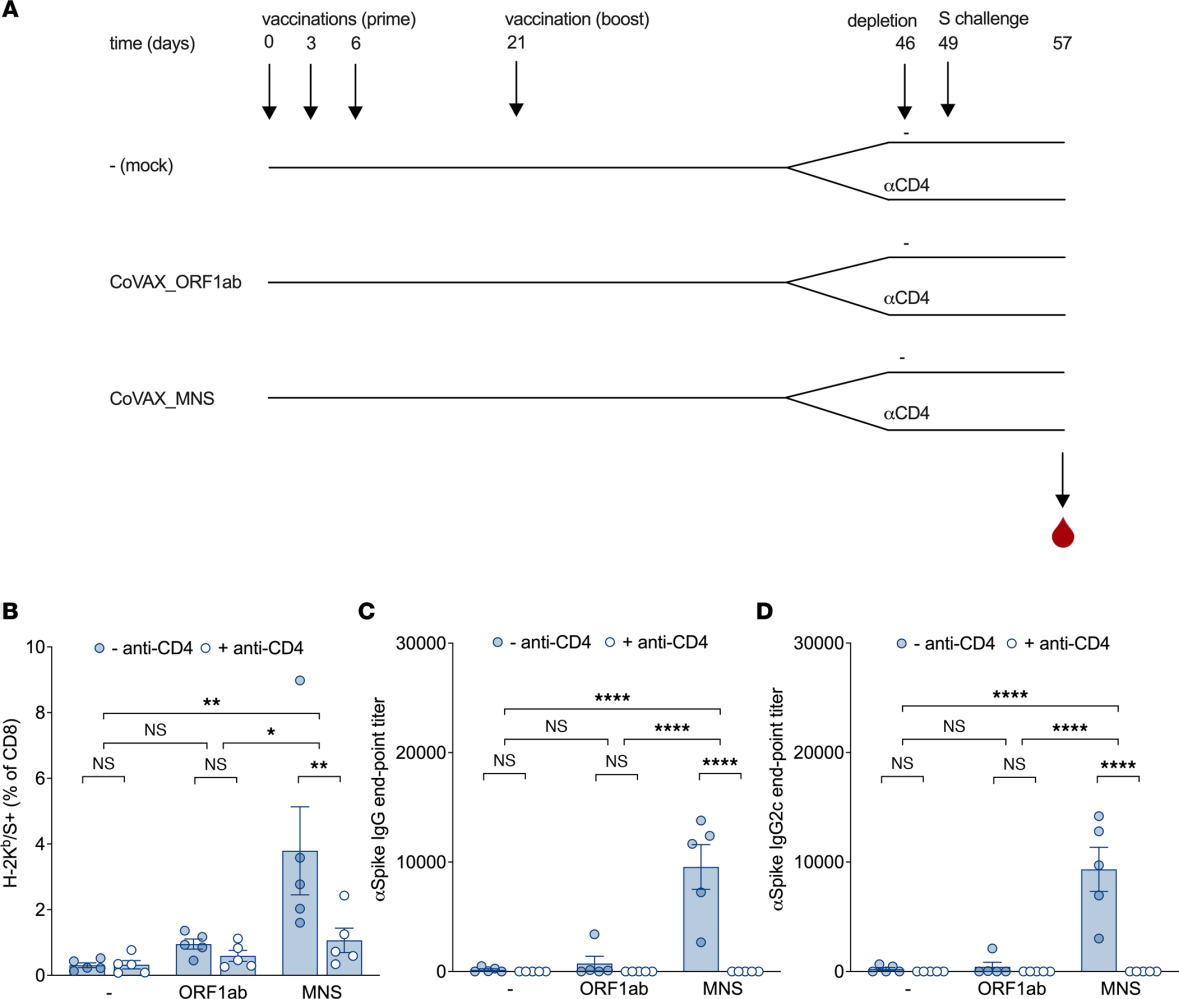
CoVAX_MNS-induced CD4^+^ T cell responses improve CD8^+^ T and B cell responses to full-length S. (**A**) Schematic representation of the S challenge experiment. C57BL/6 mice (5 mice/group) were vaccinated intradermally with mock (–), CoVAX_ORF1ab (ORF1ab), or CoVAX_MNS DNA vaccines, from which the reporter antigens had been removed (“norep” in Supplemental Table 5), on days 0, 3, 6, and 21. On day 46, CD4^+^ T cells were depleted, or not, by i.p. injection of CD4-specific Abs (αCD4), and 3 days later, mice were exposed to full-length WT SARS-CoV-2 (Omicron) S. To this end, mice were injected with DNA encoding S from SARS-CoV-2 Omicron VOC. (**B**) Eight days after exposure to these spikes, blood samples were analyzed for CD8^+^ T cell responses to the H-2K^b^–restricted S reporter antigen VNFNFNGL (absent from the vaccines, present in full-length S) as well as (**C**) IgG and (**D**) IgG2c Ab responses to the SARS-CoV-2 Omicron spikes. As expected, in the absence of an S challenge, CoVAX_MNS-vaccinated mice did not generate detectable S-specific CD8^+^ T cell (VNFNFNGL) or Ab (IgG, IgG2c) responses (data not shown). Dots represent individual mice; bars and whiskers indicate means and SEM. Tetramer (**B**) and Ab (**C** and **D**) responses were evaluated by 2-way ANOVA using Holm-Šídák multiple comparisons test. For multiplicity-adjusted *P* values: *P ≤ 0.05; **P ≤ 0.01; ****P ≤ 0.0004.

Upon infection with a sarbecovirus, vaccine-induced CD4^+^ T cells may improve CD8^+^ T cell and B cell responses specific to the incoming virus. To model this situation, mice were first vaccinated with the CoVAX_MNS vaccine and then exposed to full-length S by a subsequent DNA vaccination (Figure 5A). In this experiment, the C57BL/6 reporter epitope, absent from the CoVAX_MNS and CoVAX_ORF1ab vaccines but present in full-length S, was used to read out CD8^+^ T cell responses elicited by full-length S. Shortly after S exposure (day 8), CD8^+^ T cells with this specificity were approximately 5-fold more frequent in the blood of CoVAX_MNS compared with control mock- or CoVAX_ORF1ab-vaccinated mice (Figure 5B). At the same time point, ELISA revealed approximately 20-fold higher S-specific IgG (Figure 5C) and 30-fold higher IgG2c levels (Figure 5D) in CoVAX_MNS-vaccinated compared with control mice. In the absence of S exposure, neither S-specific CD8^+^ T cells nor Abs were detected (data not shown). For both types of responses, depletion of CD4^+^ T cells prior to S exposure eliminated this CoVAX_MNS effect (Figure 5, C–E). In conclusion, CoVAX_MNS-induced CD4^+^ T cells substantially improved S-specific CD8^+^ T cell and class-switched Ab responses.

### CoVAX_MNS vaccination improves S-specific CD8^+^ T cell and Ab responses upon exposure to SARS-CoV-1 and SARS-CoV-2 S.

**Figure 6:**
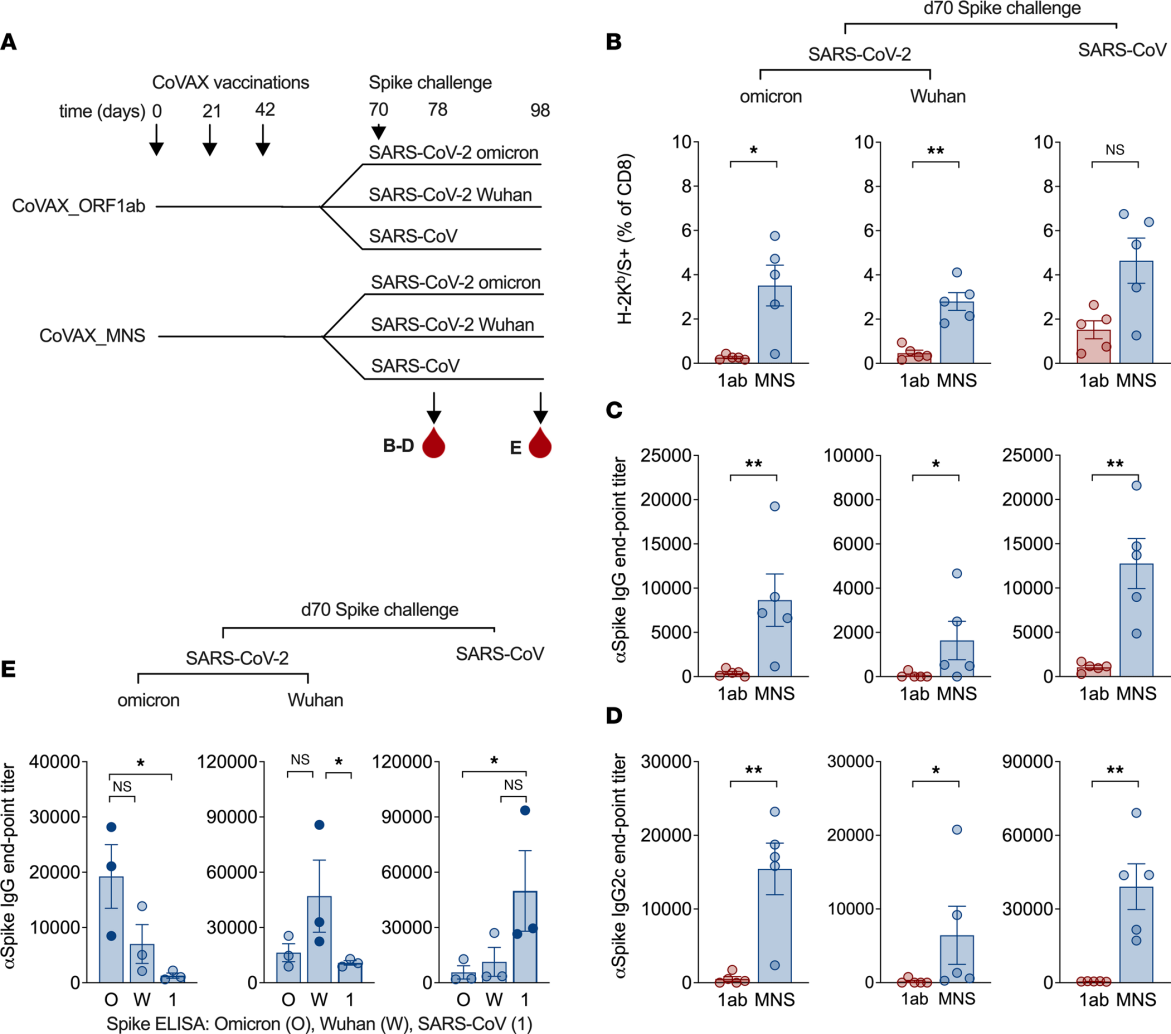
CoVAX_MNS vaccination improves CD8^+^ T and B cell responses to multiple sarbecovirus S variants. (**A**) Schematic representation of the experiment. C57BL/6 mice (5 mice/group) were vaccinated intradermally with CoVAX_ORF1ab (1ab) or CoVAX_MNS (MNS) DNA vaccines, from which the reporter antigens had been removed (“norep” in Supplemental Table 5), 3 times at 3-week intervals (days 0, 21, and 42). Three weeks after the final vaccination (day 70), mice were challenged with full-length WT S from SARS-CoV-2 Omicron, SARS-CoV-2 Wuhan, or SARS-CoV-1, also by intradermal DNA injection. Eight days after exposure to these spikes (day 78), blood samples were analyzed for (**B**) CD8^+^ T cell responses to the H-2K^b^–restricted S reporter antigen VNFNFNGL (absent from the vaccines, present in all 3 S DNAs) as well as (**C**) IgG1 and (**D**) IgG2c Ab responses to the different spikes. In the absence of an S challenge, CoVAX_MNS-vaccinated mice did not generate detectable S-specific CD8^+^ T cell (VNFNFNGL) or Ab (IgG, IgG2c) responses (data not shown). (**E**) Four weeks after exposure to full-length S (day 98), the specificity of these IgG responses was determined by testing the sera not only against the S to which the mice had been exposed (closed circles) but also against the other 2 spikes (open circles). Dots represent individual mice; bars and whiskers indicate means and SEM. Tetramer and Ab responses were evaluated by a 2-tailed Mann-Whitney (**B**–**D**) or Kruskal-Wallis test using Dunn’s multiple comparisons test (**E**). For multiplicity-adjusted *P* values: *P ≤ 0.05; **P ≤ 0.01.

Because the CoVAX_MNS antigens are shared across sarbecoviruses by design, vaccine-induced T cell responses to conserved epitopes would be expected to improve not only CD8^+^ T cell and B cell responses to SARS-CoV-2 S but also their responses to other sarbecovirus spikes, including SARS-CoV-1. To test this, mice vaccinated with CoVAX_MNS, as well as control CoVAX_ORF1ab-vaccinated mice, were exposed to full-length, WT S from SARS-CoV-2 Omicron, SARS-CoV-2 Wuhan, or SARS-CoV-1 by DNA vaccination (Figure 6A). Compared with control mice, CoVAX_MNS-vaccinated mice displayed approximately 8-fold stronger CD8^+^ T cell (Figure 6B), 20-fold higher IgG (Figure 6C), and 50-fold higher IgG2c (Figure 6D) Ab responses to all 3 S variants 8 days after injection of S DNA. Analysis of sera obtained 4 weeks after S exposure showed these Ab responses to be largely specific to the immunizing S variant (Figure 6E). At this time point, S-specific CD8^+^ T cell and Ab responses did not differ between CoVAX_ORF1ab and CoVAX_MNS-vaccinated mice (data not shown). Thus, T cell responses against shared sarbecovirus antigens induced by vaccination accelerated CD8^+^ T and B cell responses specific to a diverse array of incoming sarbecovirus S proteins.

### Pan-sarbecovirus DNA vaccines can protect against a lethal SARS-CoV-2 challenge.

**Figure 7:**
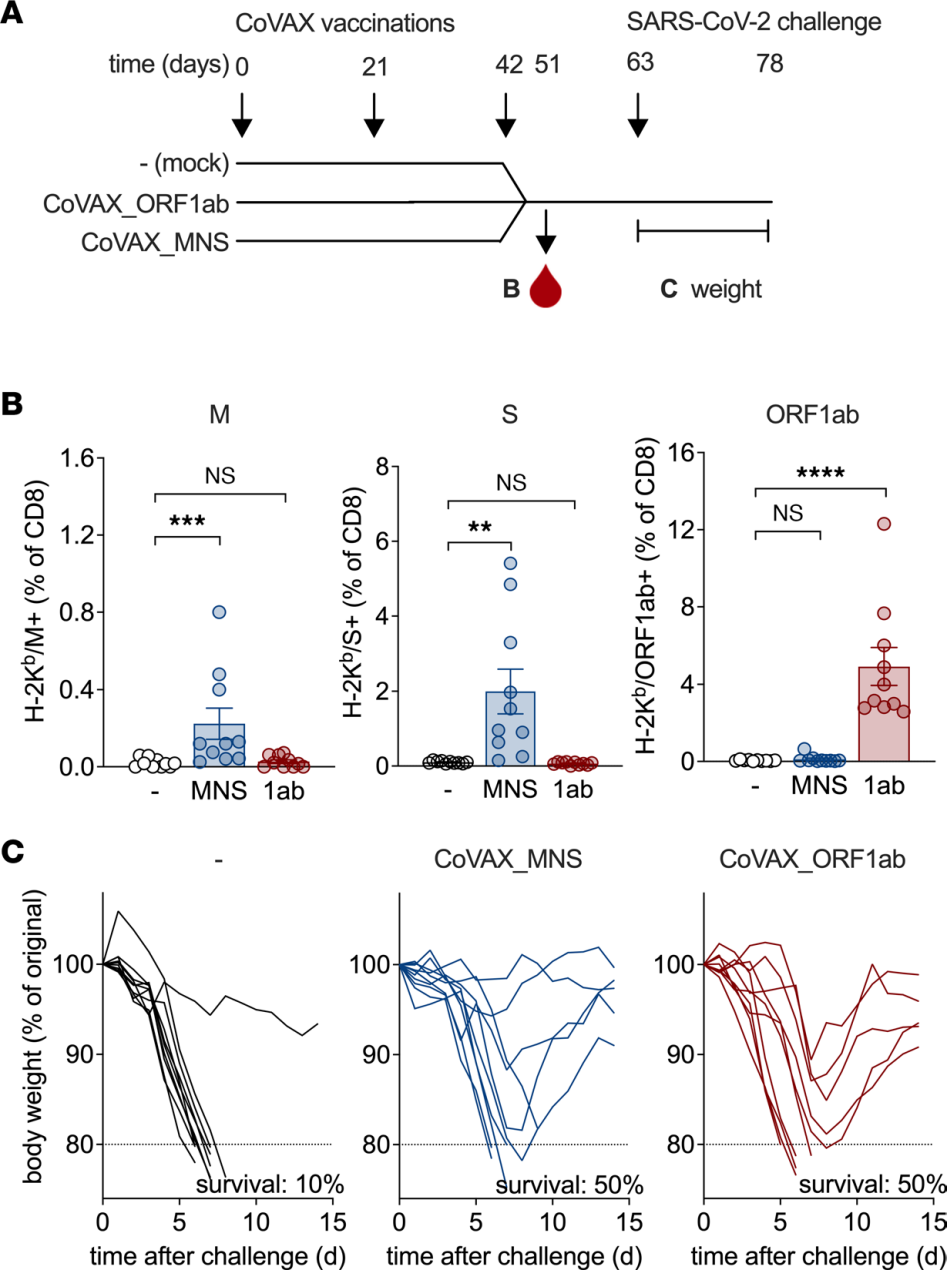
Pan-sarbecovirus DNA vaccines induce CD8^+^ T cell responses and partial protection against SARS-CoV-2 in K18-hACE2tg mice. (**A**) Schematic representation of the experiment. K18-hACE2tg mice (10 mice/group) were vaccinated intradermally with the indicated plasmid DNA vaccines thrice at 3-week intervals (days 0, 21, and 42). Mock-vaccinated animals served as negative controls. Nine days after the final vaccination (day 51), blood samples were collected to measure vaccine-specific CD8^+^ T cell responses. Three weeks after the final vaccination (day 63), the mice were challenged i.n. with a lethal dose of the Leiden-0008 SARS-CoV-2 isolate and BWs were measured daily as a parameter of disease. (**B**) CD8^+^ T cell responses in blood measured after 3 vaccinations (day 51) using H-2K^b^ tetramers containing ORF1ab nsp12-, M- (see Figure 3), or S-derived epitopes. In this experiment, CoVAX_MNS, but not CoVAX_ORF1ab, retained the C-terminal S reporter cassette; therefore, only CoVAX_MNS was able to induce responses to the S reporter epitope. Dots represent individual mice; bars and whiskers indicate means and SEM. These tetramer responses were evaluated by a Kruskal-Wallis test using Dunn’s multiple comparisons test. For multiplicity-adjusted *P* values: **P ≤ 0.01; ***P ≤ 0.002; ****P ≤ 0.0004. (**C**) BWs and survival of individual mice after SARS-CoV-2 challenge. Statistical analysis comparing the survival curves of vaccinated versus control mice by a log-rank (Mantel-Cox) test: for CoVAX_MNS, *P* = 0.06; for CoVAX_ORF1ab, *P* = 0.24.

The 2 new vaccine designs were tested for protection against a lethal SARS-CoV-2 infection. In K18-hACE2tg mice, SARS-CoV-2 infection results in quick weight loss and death within a week (59–61). To test the potency of the DNA vaccines, K18-hACE2tg mice were vaccinated 3 times with CoVAX_MNS or CoVAX_ORF1ab and then challenged i.n. with SARS-CoV-2 (Figure 7A) (25). In this experiment, CoVAX_MNS, but not CoVAX_ORF1ab, did contain the S reporter antigens. After 3 vaccinations, CD8^+^ T cell responses to the conserved M and ORF1ab antigens (Figure 3) were detected by tetramer staining of blood samples (Figure 7B). Upon SARS-CoV-2 infection, virtually all (90%) mock-vaccinated mice quickly lost weight and died, but 60%–70% of the mice vaccinated with either CoVAX_ORF1ab or CoVAX_MNS vaccine experienced delayed weight loss, and 50% of the mice regained weight and survived (Figure 7C). Protection afforded by CoVAX_ORF1ab was mediated by the ORF1ab-specific immune response because it contained only ORF1ab antigens, and no S reporter antigens. These data indicate that vaccine-induced T cell immunity can provide immune control of a lethal SARS-CoV-2 infection.

## Discussion

We designed and tested 2 T cell–based pan-sarbecovirus vaccines, 1 encoding antigens derived from nonstructural proteins (CoVAX_ORF1ab: nsp7, nsp8, nsp12, nsp13); the other, antigens from structural proteins (CoVAX_MNS: M, N, S). Both DNA vaccines contained a large set of antigens shared across sarbecoviruses and rich in predicted and validated human T cell epitopes. Injected intradermally into C57BL/6 mice without adjuvant, the multiantigen vaccines generated both CD8^+^ and CD4^+^ T cell responses to shared epitopes. Upon encounter of full-length S antigen, CoVAX_MNS-induced CD4^+^ T cells were responsible for accelerated CD8^+^ T and IgG Ab responses specific to the incoming S, irrespective of its sarbecovirus origin. Finally, both vaccines appeared to reduce the sensitivity of hACE2-transgenic mice to a lethal SARS-CoV-2 challenge.

The shared antigenic domains were selected for inclusion based on strict criteria regarding aa sequence identity to ensure that all T cell epitopes would be identical across sarbecoviruses. First and foremost, this reduces the risk that the fraction of vaccine-induced T cells contributing to protection will diverge among sarbecoviruses. Second, this reduces the risk that the “original antigenic sin” phenomenon (62, 63) reduces vaccine efficacy. In short, individuals primed with a vaccine epitope might respond to a subsequent infection by a coronavirus containing a variant of that epitope by expanding T cells directed against the initial epitope rather than priming T cells against the new variant epitope (64). This would be caused by the presence of an expanded population of vaccine-primed T cells, with a suboptimal affinity for the variant epitope, that interferes with the priming of naive T cells with a high affinity for the variant epitope. Both issues may also play a role when using pan-sarbecovirus vaccine candidates encoding full-length viral proteins such as N (65–68), because these may raise a suboptimal T cell repertoire containing T cells with divergent cross-reactivities to incoming sarbecoviruses. The risks associated with forced expression of functional viral enzymes such as nsp12 are difficult to predict. In contrast, CoVAX_MNS and CoVAX_ORF1ab contain only small (16–76 aa), almost perfectly conserved antigens that are unlikely to be enzymatically active and that can generate equally strong T cell responses to all sarbecoviruses. Of note, because even the CoVAX_ORF1ab antigens are, on average, only 80% identical to the corresponding merbecovirus sequences, these vaccines should be considered sarbecovirus specific.

For antigen selection and validation, a set of 12 HLA class I alleles was used that collectively covers 85% of the global human population (52). Each of these is part of 1 of the following 6 HLA supertypes (69, 70), which are larger groups of HLA alleles with similar peptide binding characteristics: A01-A03-A66 (A*01:01, A*02:01, A*03:01, A*11:01), A24 (A*23:01, A*24:02), B07-B35 (B*07:02, B*35:01), B08-B18-B39 (B*08:01), B15-B40 (B*40:01), and B44 (B*44:02, B*44:03) (71). In addition, the vaccine antigens also contain epitopes restricted by other HLA class I alleles. Taken together, this means that the estimated HLA class I–based population coverage will be considerably greater than 85%. Because algorithms predicting HLA class II binding are less well developed than those for HLA class I, estimating population coverage for HLA class II is more challenging. However, according to the IEDB database (55), CoVAX_ORF1ab contains 26 and CoVAX_MNS contains 127 experimentally validated CD4^+^ T cell epitopes, restricted by a diverse set of HLA alleles, suggesting that population coverage for HLA class II is also acceptable. For example, CoVAX_MNS includes a CD4^+^ T cell epitope broadly recognized across human populations (72). In short, genetic vaccines, in contrast to peptide vaccines, allow the inclusion of many large antigens, which thus resulted in vaccines with broad global population coverage.

When mice were exposed to S antigen after vaccination, CoVAX_MNS-induced CD4^+^ T cells accelerated the generation of class-switched IgG Abs specific for the incoming S. In vitro, these CD4^+^ T cells upregulated CD40L and produced TNF upon S peptide stimulation, demonstrating that vaccination elicited S-specific T helper type 1 (T_H_1) cells, which explains class switching to IgG2c. The most likely scenario is that upon encountering the S, the S-specific CD4^+^ T cells, including T_H_1 and, potentially, T follicular helper cells, are recruited to lymph nodes and promote a germinal center response, which involves interaction with naive, S-specific B cells to promote their activation and differentiation into Ab-secreting plasmablasts (73). Similarly, CoVAX_MNS-vaccinated mice exposed to full-length S had accelerated generation of CD8^+^ T cells specific for an S epitope not contained within the vaccine. Because this CoVAX_MNS effect was also dependent on the presence of CD4^+^ T cells at the time of S exposure, it was most likely mediated by S-specific CD4^+^ T cells interacting with cDC1 (cross-)presenting S epitopes to CD4^+^ and CD8^+^ T cells in lymph nodes. This effect on CD8^+^ T cell responses is also likely to occur with CoVAX_ORF1ab. Thus, when a vaccinated individual is infected with a sarbecovirus, vaccine-induced T cells specific for conserved antigens will not only attack infected cells directly but also accelerate the activation of new T and B cells recognizing nonconserved epitopes. These accelerated CD8^+^ T and Ab responses specific for the incoming sarbecovirus may well form a crucial part of the protection afforded by this type of vaccine. Similarly, our data suggest that individuals vaccinated with this type of pan-sarbecovirus vaccine will demonstrate improved responses to conventional S-based vaccines directed against any sarbecovirus.

To our knowledge, no T cell–based pan-sarbecovirus vaccines have been described to date (74, 75). One study describes clinical testing of protein vaccines that consist of RBD, the nonconserved part of SARS-CoV-2 S, combined with 5 synthetic peptides (74). These entailed promiscuous designer CD4^+^ T cell as well as conserved sarbecovirus CD8^+^ T cell epitope peptides from N, M, and the S2 part of the S proteins known to bind to multiple class I and class II HLA alleles (74, 76). Another study describes a protein vaccine that is based on a recombinant DC-targeting CD40 Ab. The SARS-CoV-2 RBD region was connected to the C-terminus of the heavy chain of this Ab and 3 conserved N (1×) and S (2×) domains to the C-terminus of its light chain (75). However, only 2 CD4 epitopes and 9 CD8 epitopes encoded by these domains were 100% identical across all sarbecoviruses. As a result, this vaccine raises SARS-CoV-2 Wuhan Abs with limited cross-reactivity to Omicron and virtually none to other sarbecoviruses, and a T cell repertoire of which a small or large fraction cross-reacts with other sarbecoviruses, depending on the sarbecovirus strain. In short, these vaccines primarily are aimed at generating SARS-CoV-2–specific Abs, together with a small, more broadly sarbecovirus-specific T cell repertoire. In contrast, the vaccines presented in this study are aimed to raise a broad T cell repertoire that recognizes multiple epitopes identical in all sarbecoviruses in all individuals.

In K18-ACE2tg mice, the T cell–based DNA vaccines protected 50% of mice from death by a SARS-CoV-2 infection that killed 90% of unvaccinated mice within a week. In the same setting, an S-encoding DNA vaccine using the same vector backbone, dose, formulation, and administration route induced neutralizing Abs and protected 100% of mice against a lethal virus challenge (25). This aligns with the notion that neutralizing Ab–inducing vaccines can prevent viral entry into cells and thereby provide sterilizing immunity. In contrast, vaccine-induced CD8^+^ T cells can kill only already infected cells, and T cell–based vaccines, therefore, probably are unable to generate sterilizing immunity. Such vaccines are more likely to protect against severe disease and death, perhaps the most important goal of vaccines. The antigens presented in this study are compatible with multiple vaccine platforms, including adenoviral and mRNA vaccines, and it remains to be determined which of these will be most effective in humans for T cell–based vaccines.

By generating broad T cell responses in most of the global human population against conserved, nontoxic, antigenic domains derived from multiple proteins that elicit a diverse array of downstream immune effector mechanisms, these next-generation vaccines have the potential to protect against severe disease caused by novel zoonotic SARS infections coming from animals like bats, pangolins, or civets. This pandemic preparedness vaccine could be tested for safety and immunogenicity in small phase I/II trials and stockpiled for immediate phase III testing as a first line of defense against a new pandemic.

## Methods

### Shared sarbecovirus antigen selection.

Potentially immunogenic antigens shared among sarbecoviruses were selected from the M, N, S, and ORF1ab proteins of the SARS-CoV-2 Wuhan-Hu-1 isolate (NC_045512.2) based on the following 2 criteria: shared and immunogenic.

“Shared” refers to identical in the sarbecoviruses listed in Supplemental Table 1. The proteins were aligned using Clustal Omega (version 1.2.2), and sequences with a minimum length of 20 aa (with one 14 aa exception) were selected that were shared between these sarbecoviruses. At each aa position, a single outlier was accepted, because this would be attributed most likely to a sequencing error.

“Immunogenic” refers to rich in peptides predicted to bind at least 1 of the most prominent HLA-A (A*01:01, A*02:01, A*03:01, A*11:01, A*23:01, A*24:02) or HLA-B (B*07:02, B*08:01, B*35:01, B*40:01, B*44:02, B*44:03) alleles (52). The aa sequence of each viral protein was analyzed by NetMHCpan EL 4.1 (51), and peptides scoring in the percentile rank <1% and *ann_IC50* < 500 nM for binding to the abovementioned HLA alleles were considered predicted binders.

For the verification of the selected antigens (listed in Supplemental Tables 3 and 4 and depicted in Figures 1 and 2), the sarbecoviruses listed in Supplemental Table 2 were used. Again, multiple sequence alignment using Clustal Omega (version 1.2.2) was performed using NC_045512.2 as the source of reference protein sequences. For every NC_045512.2 M, N, S, and ORF1ab aa position, the fraction of sarbecovirus sequences with a different aa at that position was calculated. MHCflurry (54) was used to identify peptides predicted to bind any of the most prominent HLA-A (A*01:01, A*02:01, A*03:01, A*11:01, A*23:01, A*24:02) or HLA-B (B*07:02, B*08:01, B*35:01, B*40:01, B*44:02, B*44:03) alleles (52) with affinities below 5, nM, 50 nM, or 500 nM. The open-source nature of MHCflurry allowed integration into the tool generated to produce Figures 1 and 2, which combined Clustal alignment with MHC-binding prediction. Validated SARS-CoV-2 CD8^+^ T cell epitopes presented by the aforementioned HLA alleles (52) were obtained from the IEDB Immunomebrowser (77).

### Plasmid DNA vaccine design and production.

Two plasmid DNA vaccines were designed, 1 containing M, N, and S antigens (CoVAX_MNS; Supplemental Table 3) and 1 containing ORF1ab antigens (CoVAX_ORF1ab; Supplemental Table 4). For each vaccine, the antigens were separated by AAA spacers and rationally ordered to avoid the creation of artificial peptides containing (part of) a spacer sequence and binding HLA class I (52) with high affinity, also known as junctional epitopes. When indicated, both vaccines also contained the same C-terminal CD8 S–derived reporter antigens, containing H-2K^b^ (C57BL/6) epitope VNFNFNGL (78), H-2D^d^ (Balb/c) epitope (K)CYGVSATKL (79), and HLA-A*0201 (human) epitope FIAGLIAIV (80), followed by an HA-tag. In addition, plasmid vectors encoding full-length S from SARS-CoV-1 (AAX16192.1), SARS-CoV-2 Wuhan Hu-1 (YP_009724390.1), and SARS-CoV-2 Omicron (UFO69279.1) were generated. All constructs contained a C-terminal HA-tag to verify protein expression. Codon-optimized DNA sequences coding for the resulting multiantigen or S proteins (Supplemental Table 5) were introduced into a plasmid DNA vector in which expression was driven by a strong CMV promoter. Plasmids were propagated in *E*. *coli* cultures and purified using Nucleobond Xtra Maxi EF columns (MACHEREY-NAGEL) according to the manufacturer’s instructions. For vaccination, plasmids were column-purified twice, each time using a fresh column. Flow cytometry and Western blot analysis demonstrated that 293T cells (ATCC) transfected with CoVAX_MNS or CoVAX_ORF1ab, indeed, expressed HA-tagged proteins of the correct size (data not shown).

### Mice.

WT C57BL/6J mice were purchased from Janvier Labs. The K18-hACE2tg mice, expressing the human ACE2 receptor (hACE2) under control of the cytokeratin 18 (K18) promoter (81), were obtained from The Jackson Laboratory [B6.Cg-Tg(K18-ACE2)2Prlmn/J] and bred in-house. At the start of the experiments, male and female mice were 6–8 weeks old. The mice were housed under specific pathogen–free conditions in individually ventilated cages at the Leiden University Medical Center (LUMC) animal facility.

### DNA vaccinations.

For each DNA vaccination, 50 μg of DNA, dissolved in 30 μL of a sterile buffer (0.9% NaCl, 0.09 mM Tris at pH 8.0, and 0.009 mM EDTA at pH 8.0), was injected intradermally at the base of the tail using a 0.5 mL U-100 Insulin 29G Micro-Fine needle (Becton Dickinson, catalog 324892). Mock-vaccinated mice received 30 μL of 0.9% NaCl, 0.09 mM Tris at pH 8.0, and 0.009 mM EDTA at pH 8.0.

### Flow cytometry: tetramer and ICS.

Peripheral blood from the tail vein was collected in heparin tubes (Sarstedt). Splenocytes were obtained by mincing the tissue through 70 μm nylon cell strainers (Falcon, Corning). Blood cells and splenocytes were depleted of erythrocytes using ammonium chloride lysis buffer.

For tetramer staining, cells were washed with PBA (PBS supplemented with 0.1% BSA from Sigma-Aldrich, now Merck, and 0.02% sodium azide from LUMC pharmacy) and incubated for 30 minutes at room temperature (RT) with tetramers, followed by another 30 minutes on ice in the presence of anti-CD8α.

For ICS, splenocytes were cultured with peptide-loaded D1 (82) cells for 5 hours, the final 4.5 hours of which were in the presence of 2.5 μg/mL Brefeldin A (Merck). As a positive control, splenocytes were incubated with Abs against CD3 and CD28. After this incubation, cells were washed with PBA and stained with Abs against surface markers CD3, CD4, and CD8. Subsequently, cells were fixed and permeabilized with Fixation buffer (BioLegend) and stained with Abs against intracellular markers of activation (namely, IL-2, TNF, IFN-γ, and CD40L).

Ab details are listed in Supplemental Methods, Key Resources Tables. After washing in PBA, the cells were acquired on an LSR II Flow Cytometer (BD Biosciences). Data were analyzed with FlowJo software, version 10.8.1 (Tree Star Inc.).

### Determination of Ab titers.

ELISAs were performed to determine Ab titers in sera. Nunc MaxiSorp ELISA plates (Merck) were coated with 1 μg/mL spike S1 plus S2ECD-His recombinant protein (Sino Biologicals, catalog 40589-V08B1) in ELISA coating buffer (BioLegend) overnight at 4°C. Plates were washed 5 times and blocked with 1% BSA in PBS with 0.05% Tween 20 (Sigma-Aldrich, now Merck) for 1 hour at RT. Plates were washed and incubated with serial dilutions of mouse sera and incubated for 1 hour at RT. Plates were again washed and then incubated with a 1:4,000 dilution of HRP-conjugated anti–mouse IgG secondary Ab (Southern Biotech, catalog 1030-05) and incubated for 1 hour at RT. To develop the plates, 50 μL of 3′3′,5′,5′-tetramethylbenzidine (Sigma-Aldrich, now Merck) was added to each well and incubated for 5 minutes at RT. The reaction was stopped by the addition of 50 μL 1 M H_2_SO_4_, and within 5 minutes, the plates were measured with a microplate reader (model 680; Bio-Rad) at 450 nm.

### SARS-CoV-2 lethal challenge.

Clinical isolate SARS-CoV-2/human/NLD/Leiden-0008/2020 (here, called SARS-CoV-2) was used for the SARS-CoV-2 infection of mice. The next-generation sequencing data of this virus isolate are available under GenBank accession number MT705206.1 and show 1 mutation in the Leiden-0008 virus S protein compared with the Wuhan S protein resulting in Asp>Gly substitution at position 614 (D614G). In addition, several nonsilent (C12846U and C18928U) and silent mutations (C241U, C3037U, and C1448U) in other genes were found. Isolate Leiden-0008 was propagated and titrated in Vero-E6 cells (ATCC, catalog CRL-1586). K18-hACE2tg mice were anesthetized with isoflurane gas and infected i.n. with 5 × 10^3^ PFU of SARS-CoV-2 in a total volume of 50 μL DMEM. Mouse weight and clinical discomfort were monitored daily. Euthanasia criteria were weight loss of greater than 20% of BW compared with the prestudy weight and a moribund state. All experiments with SARS-CoV-2 were performed in the Biosafety Level 3 laboratories at the LUMC.

### Statistics.

Statistical analyses were performed using GraphPad Prism (version 9.4.1), using the statistical tests mentioned in the figure legends. Multiplicity-adjusted *P* values are depicted in the figures as follows: *P ≤ 0.05, **P ≤ 0.01, ***P ≤ 0.002, and ****P ≤ 0.0004.

### Study approval.

All animal experiments were performed in accordance with Dutch Animal Ethical Committee guidelines and were approved by the Animal Welfare body of LUMC (dierexperimentencommissie [Animal Experiment Committee] consult number: AVD11600202013796), and performed according to the recommendations and guidelines set by LUMC and by the Dutch Experiments on Animals Act.

### Data availability.

Values for all data points in graphs are reported in the Supporting Data Values file. The underlying raw data files (e.g., fcs files) are available from the corresponding author upon request via email.

## Author contributions

JVB and MGMC were responsible for antigen selection, GCZ was responsible for vaccine design, cloning, and production. MGMC, INP, DV, WYL, SKM, and AAL performed experiments. MGMC, JVB, GCZ, and FO interpreted experiment results. MK and RA provided essential advice. FO supervised the project. JVB wrote the manuscript. On these grounds, and given that MGMC performed the large majority of the experiments, we consider the contributions of JVB and MGMC of comparable value and shared first authorships justified. Because JVB had a greater intellectual contribution to the project and wrote the manuscript, his name precedes that of MGMC on the author list.

## Supplementary Material

Supplemental data

Supporting data values

## Version 1 09/14/2023

In-Press Preview

## Version 2 11/08/2023

Electronic publication
