# Supplementary material for: Multiantigen pan-sarbecovirus DNA vaccines generate protective T cell immune responses
Source: JCI Insight. 2023 Nov 8;8(21):e172488. doi: 10.1172/jci.insight.172488 (PMC10721273; doi:10.1172/jci.insight.172488)
Supplement: Supplemental data [file jciinsight-8-172488-s255.pdf]

Multi-antigen pan-sarbecovirus DNA vaccines generate protective T cell immune responses

Supplemental material

2 Supplemental Methods. Key resources tables

5 Supplemental Table 1. Sarbecoviruses (discovery)

6 Supplemental Table 2. Sarbecoviruses (verification)

8 Supplemental Table 3. Shared sarbecovirus antigens included in CoVAX_MNS

9 Supplemental Table 4. Shared sarbecovirus antigens included in CoVAX_ORF1ab

10 Supplemental Table 5. Plasmid DNA ORFs

13 Supplemental Figure 1. M clustal aligment

17 Supplemental Figure 2. N clustal aligment

25 Supplemental Figure 3. S clustal aligment

47 Supplemental Figure 4. ORF1ab clustal aligment

Supplemental Methods. Key resources tables

REAGENT or RESOURCE	SOURCE	IDENTIFIER
Antibodies		
CD3ε- PerCP-Cy5.5 (clone 145-2C11)	BioLegend	100328
CD4- Brilliant Violet 605 (clone (RM4-5)	BioLegend	100547
CD8α- APC-Fire750 (clone 53-6.7)	BioLegend	100766
CD8α-Brilliant Violet 421 (clone 53-6.7)	BioLegend	100753
IL-2 APC (clone JES6-5H4)	BD Biosciences	554429
TNF FITC (clone MP6-XT22)	BioLegend	506304
IFNγ PE-Cy7 (clone XMG1.2)	BD Biosciences	557649
CD154 PE (clone MR1)	Invitrogen	12-1541-82
CD3ε NA/LE (clone 145-2C11)	BD Pharmingen	553057
CD28 NA/LE (clone 37.51)	BD Pharmingen	553294
OX40 (clone OX-86)	made in-house	n.a.
HRP-conjugated anti-mouse IgG	Southern Biotech	1030-05
HRP-conjugated anti-mouse IgG2c	Southern Biotech	1079-05
Tetramers		
H2-K^b/VNFNFNGL APC	LUMC tetramer facility	H2-K^b/S
H2-K^b/RTLSYYKL PE	LUMC tetramer facility	H2-K^b/M
H2-K^b/TGYHFREL PE	LUMC tetramer facility	H2-K^b/ORF1ab
Buffers and media		
Fixation Buffer	BioLegend	420801
Intracellular Staining Permeabilization Wash Buffer	BioLegend	421002
ammonium chloride lysis buffer	LUMC Pharmacy	n.a.
PBS	LUMC Pharmacy	n.a.
Bovine Serum Albumin Fraction V (BSA)	Roche	10735086001
PBS/0,1% BSA/0,02% NaAzide	made in-house	n.a.
PBS/0,5% BSA/0,02% NaAzide	made in-house	n.a.
rhIL-2	Cetus corporation	201-0301
IMDM	Lonza/Capricorn Scientific	/IMDM-A
Fetal Bovine Serum (FBS)	Bodinco BV	S00FD10003
B-mercaptoethanol (2-ME)	Merck	8057400259
L-glutamine (L-Glut)	Gibco	25030-024

Penicillin/Streptomycin (PS)	Gibco	15140-122
IMDM/8%FBS/25µM 2-ME/2mM L-Glut/100 U/ml PS	made in-house	n.a.
ELISA coating buffer	BioLegend	421701
Chemicals		
poly(I:C) LMW	InvivoGen	tlrl-picw
SARS-CoV spike S1+S2 ECD-His	Sino Biological	40634-V08B
SARS-CoV-2 (2019-nCoV) spike S1+S2 ECD-His	Sino Biological	40589-V08B1
SARS-CoV-2 B.1.1.529 (Omicron) S1+S2 ECD-His	Sino Biological	40589-V08H26
TMB	Sigma-Aldrich	T4444-100ML
Experimental models: organisms, strains		
SARS-CoV-2/human/NLD/Leiden-0008/2020	isolated in-house	n.a.
C57BL/6Rj	Janvier Labs, France	n.a.
B6.Cg-Tg(K18-ACE2)2Prlmn/J	Jackson Laboratory (bred in-house)	IMSR_JAX:034860
D1 cells	Paola Ricciardi-Castagnoli	n.a.
Software and algorithms		
FACSDiva v9.0	BD Biosciences	n.a.
FlowJo v10.8	BD Biosciences	n.a.
GraphPad Prism v9.4.1	Dotmatics	n.a.

SYNTHETIC PEPTIDES (produced in-house)				
amino acid sequence	protein (aa)	CoVAX Ag	application	experiment
WLLWPVTLACFVLAAVYRIN	M (55-74)	MNS #1	ICS	Figure 3, B-D (n.s.)
VTLACFVL	M (60-67)	MNS #1	ICS	Figure 3, B-D (n.s.)
LGRCDIKDLPKEITVATSRTLSYYKLGASQRV	M (156-187)	MNS #9	ICS	Figure 3, B-D (n.s.)
RTLSYYKL	M (174-181)	MNS #9	ICS	Figure 3, B-D
WPQIAQFAPSASAFFGMSRIGMEVTP	N (301-326)	MNS #11	ICS	Figure 3, B-D (n.s.)
SASAFFGM	N (310-317)	MNS #11	ICS	Figure 3, B-D (n.s.)
SAFFGMRSI	N (312-320)	MNS #11	ICS	Figure 3, B-D (n.s.)
VILLNKHIDAYKTFPPTEPKKDKKKK	N (350-375)	MNS #7	ICS	Figure 3, B-D (n.s.)
VILLNKHID	N (350-358)	MNS #7	ICS	Figure 3, B-D (n.s.)
LSTDLIKNQCVNFNFNGLTGTGVLTPSS	S* (515-542)	rep #1	ICS	Figure 3, B-D/ Figure 3, F-H (n.s.)
VNFNFNGL	S* (525-532)	rep #1	ICS	Figure 3, B-D / Figure 3, F-H
IKNQCVNFNFNGLTGTGVLTESNK	S** (n.a.)	n.a.	ICS	Figure 4 (n.s.)
KRSFIEDLLFNKVTLADAGF	S (814-833)	MNS #3	ICS	Figure 4 (n.s.)
ARDLICAQKFNGLTVLPPLLTD	S (846-867)	MNS #2	ICS	Figure 4 (n.s.)
ALQIPFAMQMAYRFNGIGVTQNVLYENQK	S (893-921)	MNS #10	ICS, IVE	Figure 4, D and E
LSSNFGAISSVLNDILSRLDKVEAE	S (966-990)	MNS #4	ICS	Figure 4 (n.s.)
VQIDRLITGRLQSLQTYVTQQLIRAAEIRA	S (991-1020)	MNS #4	ICS, IVE	Figure 3, B-D (n.s.)/ Figure 4, A-C
QTYVTQQL	S (1005-1012)	MNS #4	ICS	Figure 3, B-D (n.s.)
NNTVYDPLQPELDSFKEELDKYFKNHTSPD	S (1134-1163)	MNS #5	ICS	Figure 4 (n.s.)
FNVLFSTV	ORF1ab (4705-4712)	ORF1ab #16	ICS	Figure 3, F-H (n.s.)
TVFPPTSFGPL	ORF1ab (4711-4721)	ORF1ab #16	ICS	Figure 3, F-H (n.s.)
STGYHFREL	ORF1ab (4735-4743)	ORF1ab #16	ICS	Figure 3, F-H (n.s.)
TGYHFREL	ORF1ab (4736-4743)	ORF1ab #16	ICS	Figure 3, F-H
RAMPNMLRIM	ORF1ab (5016-5025)	ORF1ab #17	ICS	Figure 3, F-H (n.s.)
RAMPNMLRI	ORF1ab (5016-5024)	ORF1ab #17	ICS	Figure 3, F-H (n.s.)
TAYANSVFNI	ORF1ab (5079-5088)	ORF1ab #14	ICS	Figure 3, F-H (n.s.)
LMIERFVSL	ORF1ab (5246-5254)	ORF1ab #13	ICS	Figure 3, F-H (n.s.)
MIERFVSL	ORF1ab (5247-5254)	ORF1ab #13	ICS	Figure 3, F-H (n.s.)
LAIDAYPL	ORF1ab (5254-5261)	ORF1ab #13	ICS	Figure 3, F-H (n.s.)
VTQLYLGGM	ORF1ab (5384-5392)	ORF1ab #15	ICS	Figure 3, F-H (n.s.)

Membrane (M), nucleoprotein (N), spike (S) and ORF1ab amino acid positions mentioned under protein (aa) are based on SARS-CoV-2 (NC_045512.2), except those marked with *, which derive from SARS-CoV-1. **This sequence does not occur in the wild but is a hybrid of SARS-CoV spike 520-538 (IKNQCVNFNFNGLTGTGVL) and SARS-CoV-2 spike 538-557 (CVNFNFNGLTGTGVLTESNK). The sequence CVNFNFNGL is shared among sarbecoviruses. CoVAX_MNS and CoVAX_ORF1ab DNA vaccine antigens can be found in Supplemental tables 3 and 4, respectively. ICS: intracellular cytokines staining. IVE: in vitro expansion. n.a.: not applicable. n.s.: data not shown.

Supplemental Table 1. Sarbecoviruses (discovery)

**GenBank**	**Organism**	**isolate**	**host**
NC_045512.2	SARS coronavirus 2	Wuhan-Hu-1	*Homo sapiens*
MN988713.1	SARS coronavirus 2	SARS-CoV-2/human/USA/IL-CDC-02983522-001/2020	*Homo sapiens*
MN985325.1	SARS coronavirus 2	SARS-CoV-2/human/USA/WA-CDC-02982586-001/2020	*Homo sapiens*
MN938384.1	SARS coronavirus 2	2019-nCoV_HKU-SZ-002a_2020	*Homo sapiens*
MN975262.1	SARS coronavirus 2	2019-nCoV_HKU-SZ-005b_2020	*Homo sapiens*
AY278489.2	SARS coronavirus	GD01	*Homo sapiens*
AY278488.2	SARS coronavirus	BJ01	*Homo sapiens*
AY274119	SARS coronavirus	Tor2	*Homo sapiens*
FJ882957.1	SARS coronavirus	MA15	*Mus musculus*
AY572034.1	SARS coronavirus	civet007	*Civet*
MG772933	Bat SARS-like coronavirus	bat-SL-CoVZC45	*Rhinolophus pusillus*
MG772934	Bat SARS-like coronavirus	bat-SL-CoVZXC21	*Rhinolophus pusillus*
DQ412043.1	Bat SARS-like coronavirus	Rm1	*Rhinolophus macrotis*
KY417144.1	Bat SARS-like coronavirus	Rs4084	*Rhinolophus sinicus*
DQ071615	Bat SARS-like coronavirus	Rp3	*Bat*
DQ022305	Bat SARS-like coronavirus	HKU3-1	*Rhinolophus*
GU190215	Bat coronavirus	BtCoV/BM48-31/BGR/2008	*Rhinolophus blasii*

To obtain a cross-section of all sarbecovirus branches, sarbecoviruses were selected from Figures 3 in references 46 and 47, based on the availability of full genomic sequences.

Supplemental Table 2. Sarbecoviruses (verification)

GenBank	organism	isolate	host
NC_045512.2	SARS coronavirus 2	Wuhan-Hu-1	*Homo sapiens*
MN996529.1	SARS coronavirus 2	WIV05	*Homo sapiens*
MT093571.1	SARS coronavirus 2	SARS-CoV-2/human/SWE/01/2020	*Homo sapiens*
MN988713.1	SARS coronavirus 2	SARS-CoV-2/human/USA/IL-CDC-02983522-001/2020	*Homo sapiens*
MT072688.1	SARS coronavirus 2	SARS-CoV-2/human/NPL/61-TW/2020	*Homo sapiens*
MN994467.1	SARS coronavirus 2	SARS-CoV-2/human/USA/CA-CDC-02993471-001/2020	*Homo sapiens*
AP006560.1	SARS coronavirus	TWS	*Homo sapiens*
AP006557.1	SARS coronavirus	TWH	*Homo sapiens*
AY485277.1	SARS coronavirus	Sino1-11	*Homo sapiens*
AY278488.2	SARS coronavirus	BJ01	*Homo sapiens*
AY274119	SARS coronavirus	Tor2	*Homo sapiens*
FJ959407.1	SARS coronavirus	A001	*palm civet*
AY572038.1	SARS coronavirus	civet020	*Civet*
AY572034.1	SARS coronavirus	civet007	*Civet*
KY417146.1	Bat SARS-like coronavirus	Rs4231	*Rhinolophus sinicus*
KC881006.1	Bat SARS-like coronavirus	Rs3367	*Rhinolophus sinicus*
KF367457.1	Bat SARS-like coronavirus	WIV1	*Rhinolophus sinicus*
KY417144.1	Bat SARS-like coronavirus	Rs4084	*Rhinolophus sinicus*
KC881005.1	Bat SARS-like coronavirus	RsSHC014	*Rhinolophus sinicus*
KY417152.1	Bat SARS-like coronavirus	Rs9401	*Rhinolophus sinicus*
KY417151.1	Bat SARS-like coronavirus	Rs7327	*Rhinolophus sinicus*
KY417145.1	Bat SARS-like coronavirus	Rf4092	*Rhinilophus ferrumequinum*
KY417147.1	Bat SARS-like coronavirus	Rs4237	*Rhinolophus sinicus*
KY417148.1	Bat SARS-like coronavirus	Rs4247	*Rhinolophus sinicus*
KY417142.1	Bat SARS-like coronavirus	As6526	*Aselliscus stoliczkanus*
KY417143.1	Bat SARS-like coronavirus	Rs4081	*Rhinolophus sinicus*
FJ588686.1	Bat SARS-like coronavirus	Rs672	*Rhinolophus sinicus*
KP886809.1	Bat SARS-like coronavirus	YNLF_34C	*Rhinolophus Ferrumequinum*
KF569996.1	Bat SARS-like coronavirus	LYRa11	*Rhinolophus affinis*
GQ153542.1	Bat SARS-like coronavirus	HKU3-7	*Rhinolophus bat*
DQ022305	Bat SARS-like coronavirus	HKU3-1	*Rhinolophus bat*
MN996532.2	Bat SARS-like coronavirus	RaTG13	*Rhinolophus affinis*

MG772933.1	Bat SARS-like coronavirus	bat-SL-CoVZC45	*Rhinolophus pusillus*
MG772934.1	Bat SARS-like coronavirus	bat-SL-CoVZXC21	*Rhinolophus pusillus*
KT444582.1	Bat SARS-like coronavirus	WIV16	*Rhinolophus sinicus*
GU190215	Bat coronavirus	BtCoV/BM48-31/BGR/2008	*Rhinolophus blasii*
MW532698.1	Pangolin coronavirus	GX_P2V	*Pangolin*
MT040336.1	Pangolin coronavirus	PCoV_GX-P5E	*Manis javanica*
MT040335.1	Pangolin coronavirus	PCoV_GX-P5L	*Manis javanica*
MT040334.1	Pangolin coronavirus	PCoV_GX-P1E	*Manis javanica*
MT072865.1	Pangolin coronavirus	PCoV_GX-P3B	*Pangolin*
MT040333.1	Pangolin coronavirus	PCoV_GX-P4L	*Manis javanica*

To obtain cross-section of all sarbecovirus branches, all sarbecoviruses from https://nextstrain.org/groups/blab/sars-like-cov (reference 52) were selected, provided that their full genomic sequences were available.

Supplemental Table 3. Shared sarbecovirus antigens included in CoVAX_MNS

#	protein	AA (protein)	AA (vaccine)	sequence
1	M	53-75	3-25	FLWLLWPVTLACFVLAAVYRINW
2	S	846-867	29-50	ARDLICAQKFNGLTVLPPLLTD
3	S	814-833	54-73	KRSFIEDLLFNKVTLADAGF
4	S	966-920	77-131	LSSNFGAISSVLNDILSRLDKVEAEVQIDRLITGRLQSLQTYVTQQLIRAAEIRA
5	S	1134-1163	135-164	NNTVYDPLQPELDSFKEELDKYFKNHTSPD
6	M	98-124	168-194	ASFRLFARTRSMWSFNPETNILLNVPL
7	N	350-375	198-223	VILLNKHIDAYKTFPPTEPKKDKKKK
8	S	944-979	227-262	ALGKLQDVVNQNAQALNTLVKQLSSNFGAISSVLND
9	M	156-178	266-297	LGRCDIKDLPKEITVATS**RTLSYYKL**GASQRV
10	S	893-921	301-329	ALQIPFAMQMAYRFNGIGVTQNVLYENQK
11	N	301-326	333-358	WPQIAQFAPSASAFFGMSRIGMEVTP
12	N	239-266	362-389	QQQQGQTVTKKSAAEASKKPRQKRTATK
13	N	158-191	393-426	VLQLPQGTTLPKGFYAEGSRGGSQASSRSSSRSR
14	N	38-62	430-454	KQRRPQGLPNNTASWFTALTQHGKE
15	N	268-289	458-479	YNVTQAFGRRGPEQTQGNFGDQ
16	N	132-151	483-502	WVATEGALNTPKDHIGTRNP
17	S	1007-1054	506-553	YVTQQLIRAAEIRASANLAATKMSECVLGQSKRVDFCGKGYHLMSFPQ
rep1	S*	356-384	557-585	YNSTFFSTFK**CYGVSATKL**NDLCFSNVYA
rep2	S*	515-542	588-615	LSTDLIKNQC**VNFNFNGL**TGTGVLTPSS
rep3	S	1210-1238	619-647	IKWPWYIWLG**FIAGLIAIV**MVTIMLCCMT
tag	HA	-	652-660	YPYDVPDYA

Amino acid positions are based on the NC_045512.2 M, N and S proteins. Three C-terminal non-shared reporter antigens from SARS-CoV-1 or SARS-CoV-2 spike were added to facilitate tracking of CD8 T cell immune responses in BALB/c (K^d/(K)CYGVSATKL), C57BL/6 (K^b/VNFNFNGL) and HLA-A2-transgenic (HLA-A*02:01/FIAGLIAIV) mice. The N-terminus of the vaccine (AA 1-2) coded for MG to obtain a Kozak-optimised translation start site, antigens were separated by triple-alanine-spacers, and the C-terminus of the polyprotein consisted of an HA-tag for Western blot and FACS detection. In CoVAX_MNS_norep (see Supplemental Table 5), the reporter antigens (residues 554-647) were deleted. *SARS-CoV-1 sequence.

Supplemental Table 4. Shared sarbecovirus antigens included in CoVAX_ORF1ab

#	ORF1ab protein	AA (protein)	AA (vaccine)	sequence
1	nsp12	5137-5160	3-26	FYAYLRKHFSMMILSDDAVVCFNS
2	nsp13	5603-5645	30-72	TLQGPPGTGKSHFAIGLALYYPSARIVYTACSHAAVDALCEKA
3	nsp13	5683-5744	76-137	TVNALPETTADIVVFDEISMATNYDLSVVNARLRAKHYVYIGDPAQLPAPRTLLTKGTLEPE
4	nsp12	4799-4858	141-200	FQTVKPGNFNKDFYDFAVSKGFFKEGSSVELKHFFFAQDGNAAISDYDYYRYNLPTMCDI
5	nsp13	5497-5526	204-233	RPPLNRNYVFTGYRVTKNSKVQIGEYTFEK
6	nsp7	3859-3879	237-257	QSKMSDVKCTSVVLLSVLQQL
7	nsp12	4885-4917	261-293	VIVNNLDKSAGFPFNKWGKARLYYDSMSYEDQD
8	nsp12	4919-4990	297-368	LFAYTKRNVIPTITQMNLKYAISAKNRARTVAGVSICSTMTNRQFHQKLLKSIAATRGATVVIGTSKFYGGW
9	nsp12	5178-5210	372-404	LYYQNNVFMSEAKCWTETDLTKGPHEFCSQHTM
10	nsp12	4591-4615	408-432	AGIVGVLTLDNQDLNGNWYDFGDFI
11	nsp13	5454-5467	436-449	LKLFAAETLKATEE
12	nsp8	3999-4043	453-497	RKLEKMADQAMTQMYKQARSEDKRAKVTSAMQTMLFTMLRKLDND
13	nsp12	5220-5264	501-545	YLPYPDPSRILGAGCFVDDIVKTDGTLMIERFVSLAIDAYPLTKH
14	nsp12	5043-5088	549-594	RFYRLANECAQVLSEMVMCGGSLYVKPGGTSSGDATTAYANSVFNI
15	nsp13	5329-5396	598-665	CVLCNSQTSLRCGACIRRPFLCCKCCYDHVISTSHKLVLSVNPYVCNAPGCDVTDVTQLYLGGMSYYC
16	nsp12	4695-4746	669-720	DDRCILHCANFNVLFSTVFPPTSFGPLVRKIFVDGVPFVVS**TGYHFREL**GVV
17	nsp12	5004-5034	724-754	PHLMGWDYPKCDRAMPNMLRIMASLVLARKH
rep	S*	356-384	758-786	YNSTFFSTFK**CYGVSATKL**NDLCFSNVYA
rep	S*	515-542	789-816	LSTDLIKNQC**VNFNFNGL**TGTGVLTPSS
rep	S	1210-1238	820-848	IKWPWYIWLG**FIAGLIAIV**MVTIMLCCMT
tag	- (HA)	-	853-861	YPYDVPDYA

Amino acid positions are based on the ORF1ab protein of NC_045512.2. Three C-terminal non-shared reporter antigens from SARS-CoV-1 or SARS-CoV-2 spike were added to facilitate tracking of CD8 T cell immune responses in BALB/c (Kd/(K)CYGVSATKL), C57BL/6 (Kb/VNFNFNGL) and HLA-A2-transgenic (HLA-A*02:01/FIAGLIAIV) mice. The N-terminus of the vaccine (AA 1-2) coded for MG to obtain a Kozak-optimised translation start site, antigens were separated by triple-alanine-spacers, and the C-terminus of the polyprotein consisted of an HA-tag for Western blot and FACS detection. In CoVAX_ORF1ab_norep (see Supplemental Table 5), the reporter antigens (residues 755-848) were deleted. *SARS-CoV-1 sequence

Supplemental Table 5. Plasmid DNA ORFs

#	name	description	amino acid sequence
p380	CoVAX_MNS	see Table S3	MGFLWLLWPVTLACFVLAAVYRINWAAAARDLICAQKFNGLTVLPPLLTDAAAKR<br>SFIEDLLFNKVTLADAGFAAALSSNFGAISSVLNDILSRLDKVEAEVQIDRLITGRLQSL<br>QTYVTQQLIRAAEIRAAAANNTVYDPLQPELDSFKEELDKYFKNHTSPDAAAASFRL<br>FARTRSMWSFNPETNILLNVPLAAAVILLNKHIDAYKTFPPTEPKKDKKKKAAAALG<br>KLQDVVNQNAQALNTLVKQLSSNFGAISSVLNDAAALGRCDIKDLPKEITVATSRTL<br>SYYKLGASQRVAAAALQIPFAMQMAYRFNGIGVTQNVLYENQKAAAWPQIAQFA<br>PSASAFFGMSRIGMEVTPAAAQQQQGQTVTKKSAAEASKKPRQKRTATKAAAVL<br>QLPQGTTLPKGFYAEGSRGGSQASSRSSSRSRAAAKQRRPQGLPNNTASWFTALT<br>QHGKEAAAYNVTQAFGRRGPEQTQGNFGDQAAAWVATEGALNTPKDHIGTRN<br>PAAAYVTQQLIRAAEIRASANLAATKMSECVLGQSKRVDFCGKGYHLMSFPQAAA<br>YNSTFFSTFKCYGVSATKLNDLCFSNVYAAALSTDLIKNQCVNFNFNGLTGTGVLTP<br>SSAAAIKWPWYIWLGFIAGLIAIVMVTIMLCCMTGSGSYPYDVPDYA
p413	CoVAX_MNS_norep	see Table S3	MGFLWLLWPVTLACFVLAAVYRINWAAAARDLICAQKFNGLTVLPPLLTDAAAKR<br>SFIEDLLFNKVTLADAGFAAALSSNFGAISSVLNDILSRLDKVEAEVQIDRLITGRLQSL<br>QTYVTQQLIRAAEIRAAAANNTVYDPLQPELDSFKEELDKYFKNHTSPDAAAASFRL<br>FARTRSMWSFNPETNILLNVPLAAAVILLNKHIDAYKTFPPTEPKKDKKKKAAAALG<br>KLQDVVNQNAQALNTLVKQLSSNFGAISSVLNDAAALGRCDIKDLPKEITVATSRTL<br>SYYKLGASQRVAAAALQIPFAMQMAYRFNGIGVTQNVLYENQKAAAWPQIAQFA<br>PSASAFFGMSRIGMEVTPAAAQQQQGQTVTKKSAAEASKKPRQKRTATKAAAVL<br>QLPQGTTLPKGFYAEGSRGGSQASSRSSSRSRAAAKQRRPQGLPNNTASWFTALT<br>QHGKEAAAYNVTQAFGRRGPEQTQGNFGDQAAAWVATEGALNTPKDHIGTRN<br>PAAAYVTQQLIRAAEIRASANLAATKMSECVLGQSKRVDFCGKGYHLMSFPQGSG<br>SYPYDVPDYA
p367	CoVAX_ORF1ab	see Table S4	MGFYAYLRKHFSMMILSDDAVVCFNSAAATLQGPPGTGKSHFAIGLALYYPSARIV<br>YTACSHAAVDALCEKAAAATVNALPETTADIVVFDEISMATNYDLSVVNARLRAKH<br>YVVIGDPAQLPAPRTLLTKGTLEPEAAAFQTVKPGNFNKDFYDFAVSKGFFKEGSSV<br>ELKHFFFAQDGNAAISDYDYYRYNLPTMCDIAAARPPLNRNYVFTGYRVTKNSKVQ<br>IGEYTFEKAAAQSKMSDVKCTSVVLLSVLQQLAAAVIVNNLDKSAGFPFNKWGKA<br>RLYYDSMSYEDQDAAALFAYTKRNVIPTITQMNLKYAISAKNRARTVAGVSICSTM<br>TNRQFHQKLLKSIAATRGATVVIGTSKFYGGWAAALYYQNNVFMSEAKCWTETDL<br>TKGPHEFCSQHTMAAAAGIVGVLTLDNQDLNGNWYDFGDFIAAALKLFAAETLKA<br>TEEAAARKLEKMADQAMTQMYKQARSEDKRAKVTSAMQTMLFTMLRKLDNDA<br>AAYLPYPDPSRILGAGCFVDDIVKTDGTLMIERFVSLAIDAYPLTKHAAARFYRLANE<br>CAQVLSEMVMCGGSLYVKPGGTSSGDATTAYANSVFNIAAACVLCNSQTSLRCGA<br>CIRRPFLCCKCCYDHVISTSHKLVLSVNPYVCNAPGCDVTDVTQLYLGGMSYYCAA<br>ADDRCILHCANFNVLFSTVFPPTSFGPLVRKIFVDGVPFVVSTGYHFRELGVVAAAP<br>HLMGWDYPKCDRAMPNMLRIMASLVLARKHAAAYNSTFFSTFKCYGVSATKLND<br>LCFSNVYAAALSTDLIKNQCVNFNFNGLTGTGVLTPSSAAAIKWPWYIWLGFIAGLI<br>AIVMVTIMLCCMTGSGSYPYDVPDYA
p415	CoVAX_ORF1ab_norep	see Table S4	MGFYAYLRKHFSMMILSDDAVVCFNSAAATLQGPPGTGKSHFAIGLALYYPSARIV<br>YTACSHAAVDALCEKAAAATVNALPETTADIVVFDEISMATNYDLSVVNARLRAKH<br>YVVIGDPAQLPAPRTLLTKGTLEPEAAAFQTVKPGNFNKDFYDFAVSKGFFKEGSSV<br>ELKHFFFAQDGNAAISDYDYYRYNLPTMCDIAAARPPLNRNYVFTGYRVTKNSKVQ<br>IGEYTFEKAAAQSKMSDVKCTSVVLLSVLQQLAAAVIVNNLDKSAGFPFNKWGKA<br>RLYYDSMSYEDQDAAALFAYTKRNVIPTITQMNLKYAISAKNRARTVAGVSICSTM<br>TNRQFHQKLLKSIAATRGATVVIGTSKFYGGWAAALYYQNNVFMSEAKCWTETDL<br>TKGPHEFCSQHTMAAAAGIVGVLTLDNQDLNGNWYDFGDFIAAALKLFAAETLKA<br>TEEAAARKLEKMADQAMTQMYKQARSEDKRAKVTSAMQTMLFTMLRKLDNDA<br>AAYLPYPDPSRILGAGCFVDDIVKTDGTLMIERFVSLAIDAYPLTKHAAARFYRLANE<br>CAQVLSEMVMCGGSLYVKPGGTSSGDATTAYANSVFNIAAACVLCNSQTSLRCGA<br>CIRRPFLCCKCCYDHVISTSHKLVLSVNPYVCNAPGCDVTDVTQLYLGGMSYYCAA<br>ADDRCILHCANFNVLFSTVFPPTSFGPLVRKIFVDGVPFVVSTGYHFRELGVVAAAP<br>HLMGWDYPKCDRAMPNMLRIMASLVLARKHGSGSYPYDVPDYA
p391	spike_FL2_HA	spike SARS-CoV-2 'Wuhan-Hu-1' (YP_009724390.1)	MFVFLVLLPLVSSQCVNLTTRTQLPPAYTNSFTRGVYYPDKVFRSSVLHSTQDLFLPF<br>FSNVTWFHAIHVSGTNGTKRFDNPVLPFNDGVYFASTEKSNIIRGWIFGTTLDSKT<br>QSLLIVNNATNVVIKVCEFQFCNDPFLGVYYHKNNKSWMESEFRVYSSANNCTFEY<br>VSQPFLMDLEGKQGNFKNLREFVFKNIDGYFKIYSKHTPINLVRDLPQGFSALEPLV<br>DLPIGINITRFQTLLALHRSYLTPGDSSSGWTAGAAAYYVGYLQPRTFLLKYNENGTI<br>TDAVDCALDPLSETKCTLKSFTVEKGIYQTSNFRVQPTESIVRFPNITNLCPFGEVFN<br>ATRFASVYAWNRKRISNCVADYSVLYNSASFSTFKCYGVSPTKLNDLCFTNVYADSF<br>VIRGDEVRQIAPGQTGKIADYNYKLPDDFTGCVIAWNSNNLDSKVGGNYNYLYRLF<br>RKSNLKPFERDISTEIYQAGSTPCNGVEGFNCYFPLQSYGFQPTNGVGYQPYRVVVL<br>SFELLHAPATVCGPKKSTNLVKNKCVNFNFNGLTGTGVLTESNKKFLPFQQFGRDIA<br>DTTDAVRDPQTLEILDITPCSFGGVSVITPGTNTSNQVAVLYQDVNCTEVPVAIHAD<br>QLTPTWRVYSTGSNVFQTRAGCLIGAEHVNNSYECDIPIGAGICASYQTQTNSPRR<br>ARSVASQSIIAYTMSLGAENSVAYSNNSIAIPTNFTISVTTEILPVSMTKTSVDCTMYI

			CGDSTECSNLLLQYGSFCTQLNRALTGIAVEQDKNTQEVFAQVKQIYKTPPIKDFGG<br>FNFSQILPDPSKPSKRSFIEDLLFNKVTLADAGFIKQYGDCLGDIAARDLICAQKFNGL<br>TVLPPLLTDEMIAQYTSALLAGTITSGWTFGAGAALQIPFAMQMAYRFNGIGVTQ<br>NVLYENQKLIANQFNSAIGKIQDSLSSTASALGKLQDVVNQNAQALNTLVKQLSSN<br>FGAISSVLNDILSRLDKVEAEVQIDRLITGRLQSLQTYVTQQLIRAAEIRASANLAATK<br>MSECVLGQSKRVDFCGKGYHLMSFPQSAPHGVVFLHVTYVPAQEKNFTTAPAICH<br>DGKAHFPREGVFVSNGTHWFVTQRNFYEPQIITTDNTFVSGNCDVVIGIVNNTVY<br>DPLQPELDSFKEELDKYFKNHTSPDVDLGDISGINASVVNIQKEIDRLNEVAKNLNES<br>LIDLQELGKYEQYIKWPWYIWLGFIAGLIAIVMVTIMLCCMTSCCSCLKGCCSCGSC<br>CKFDEDDSEPVLKGVKLHYTGSGSYPYDVPDYA
p393	sspike-noF	spike SARS-CoV-2 'Wuhan', optimised: (prefusion-stabilised (S2P), removed transmembrane domain and furin cleavage sites, added trimerisation signal and universal helper antigens)	MFVFLVLLPLVSSQCVNLTTRTQLPPAYTNSFTRGVYYPDKVFRSSVLHSTQDLFLPF<br>FSNVTWFHAIHVSGTNGTKRFDNPVLPFNDGVYFASTEKSNIIRGWIFGTTLDSKT<br>QSLLIVNNATNVVIKVCEFQFCNDPFLGVYYHKNNKSWMESEFRVYSSANNCTFEY<br>VSQPFLMDLEGKQGNFKNLREFVFKNIDGYFKIYSKHTPINLVRDLPQGFSALEPLV<br>DLPIGINITRFQTLLALHRSYLTPGDSSSGWTAGAAAYYVGYLQPRTFLLKYNENGTI<br>TDAVDCALDPLSETKCTLKSFTVEKGIYQTSNFRVQPTESIVRFPNITNLCPFGEVFN<br>ATRFASVYAWNRKRISNCVADYSVLYNSASFSTFKCYGVSPTKLNDLCFTNVYADSF<br>VIRGDEVRQIAPGQTGKIADYNYKLPDDFTGCVIAWNSNNLDSKVGGNYNYLYRLF<br>RKSNLKPFERDISTEIYQAGSTPCNGVEGFNCYFPLQSYGFQPTNGVGYQPYRVVVL<br>SFELLHAPATVCGPKKSTNLVKNKCVNFNFNGLTGTGVLTESNKKFLPFQQFGRDIA<br>DTTDAVRDPQTLEILDITPCSFGGVSVITPGTNTSNQVAVLYQDVNCTEVPVAIHAD<br>QLTPTWRVYSTGSNVFQTRAGCLIGAEHVNNSYECDIPIGAGICASYQTQTNSPSS<br>ARSVASQSIIAYTMSLGAENSVAYSNNSIAIPTNFTISVTTEILPVSMTKTSVDCTMYI<br>CGDSTECSNLLLQYGSFCTQLNRALTGIAVEQDKNTQEVFAQVKQIYKTPPIKDFGG<br>FNFSQILPDPSKPSKRSFIEDLLFNKVTLADAGFIKQYGDCLGDIAARDLICAQKFNGL<br>TVLPPLLTDEMIAQYTSALLAGTITSGWTFGAGAALQIPFAMQMAYRFNGIGVTQ<br>NVLYENQKLIANQFNSAIGKIQDSLSSTASALGKLQDVVNQNAQALNTLVKQLSSN<br>FGAISSVLNDILSRLDPPEAEVQIDRLITGRLQSLQTYVTQQLIRAAEIRASANLAATK<br>MSECVLGQSKRVDFCGKGYHLMSFPQSAPHGVVFLHVTYVPAQEKNFTTAPAICH<br>DGKAHFPREGVFVSNGTHWFVTQRNFYEPQIITTDNTFVSGNCDVVIGIVNNTVY<br>DPLQPELDSFKEELDKYFKNHTSPDVDLGDISGINASVVNIQKEIDRLNEVAKNLNES<br>LIDLQELGKYEQYIKWPGGGTGGGNGTGRMKQIEDKIEEILSKIYNITNEIARIKKLIG<br>NRTGGGGSGFNNFTVSFWLRVPKVSASHLEGPGPGAKFVAAWTLKAAAGPGPGY<br>PYDVPDYA
p422	sspike-noF-noHELP	spike SARS-CoV-2 'Wuhan-Hu-1', optimised: (prefusion-stabilised (S2P), removed transmembrane domain and furin cleavage sites, added trimerisation signal)	MFVFLVLLPLVSSQCVNLTTRTQLPPAYTNSFTRGVYYPDKVFRSSVLHSTQDLFLPF<br>FSNVTWFHAIHVSGTNGTKRFDNPVLPFNDGVYFASTEKSNIIRGWIFGTTLDSKT<br>QSLLIVNNATNVVIKVCEFQFCNDPFLGVYYHKNNKSWMESEFRVYSSANNCTFEY<br>VSQPFLMDLEGKQGNFKNLREFVFKNIDGYFKIYSKHTPINLVRDLPQGFSALEPLV<br>DLPIGINITRFQTLLALHRSYLTPGDSSSGWTAGAAAYYVGYLQPRTFLLKYNENGTI<br>TDAVDCALDPLSETKCTLKSFTVEKGIYQTSNFRVQPTESIVRFPNITNLCPFGEVFN<br>ATRFASVYAWNRKRISNCVADYSVLYNSASFSTFKCYGVSPTKLNDLCFTNVYADSF<br>VIRGDEVRQIAPGQTGKIADYNYKLPDDFTGCVIAWNSNNLDSKVGGNYNYLYRLF<br>RKSNLKPFERDISTEIYQAGSTPCNGVEGFNCYFPLQSYGFQPTNGVGYQPYRVVVL<br>SFELLHAPATVCGPKKSTNLVKNKCVNFNFNGLTGTGVLTESNKKFLPFQQFGRDIA<br>DTTDAVRDPQTLEILDITPCSFGGVSVITPGTNTSNQVAVLYQDVNCTEVPVAIHAD<br>QLTPTWRVYSTGSNVFQTRAGCLIGAEHVNNSYECDIPIGAGICASYQTQTNSPSS<br>ARSVASQSIIAYTMSLGAENSVAYSNNSIAIPTNFTISVTTEILPVSMTKTSVDCTMYI<br>CGDSTECSNLLLQYGSFCTQLNRALTGIAVEQDKNTQEVFAQVKQIYKTPPIKDFGG<br>FNFSQILPDPSKPSKRSFIEDLLFNKVTLADAGFIKQYGDCLGDIAARDLICAQKFNGL<br>TVLPPLLTDEMIAQYTSALLAGTITSGWTFGAGAALQIPFAMQMAYRFNGIGVTQ<br>NVLYENQKLIANQFNSAIGKIQDSLSSTASALGKLQDVVNQNAQALNTLVKQLSSN<br>FGAISSVLNDILSRLDPPEAEVQIDRLITGRLQSLQTYVTQQLIRAAEIRASANLAATK<br>MSECVLGQSKRVDFCGKGYHLMSFPQSAPHGVVFLHVTYVPAQEKNFTTAPAICH<br>DGKAHFPREGVFVSNGTHWFVTQRNFYEPQIITTDNTFVSGNCDVVIGIVNNTVY<br>DPLQPELDSFKEELDKYFKNHTSPDVDLGDISGINASVVNIQKEIDRLNEVAKNLNES<br>LIDLQELGKYEQYIKWPGGGTGGGNGTGRMKQIEDKIEEILSKIYNITNEIARIKKLIG<br>NRTGGGYPYDVPDYA
p489	Omicron-HA_p310bb	spike SARS-CoV-2 B.1.1.529 'Omicron' (UFO69279.1)	MFVFLVLLPLVSSQCVNLTTRTQLPPAYTNSFTRGVYYPDKVFRSSVLHSTQDLFLPF<br>FSNVTWFHVISGTNGTKRFDNPVLPFNDGVYFASIEKSNIIRGWIFGTTLDSKTQSLL<br>IVNNATNVVIKVCEFQFCNDPFLDHKNNKSWMESEFRVYSSANNCTFEYVSQPFL<br>MDLEGKQGNFKNLREFVFKNIDGYFKIYSKHTPIIVREPEDLPQGFSALEPLVDLPIGI<br>NITRFQTLLALHRSYLTPGDSSSGWTAGAAAYYVGYLQPRTFLLKYNENGTITDAVD<br>CALDPLSETKCTLKSFTVEKGIYQTSNFRVQPTESIVRFPNITNLCPFDEVFNATRFAS<br>VYAWNRKRISNCVADYSVLYNLAPFFTFKCYGVSPTKLNDLCFTNVYADSFVIRGDE<br>VRQIAPGQTGNIADYNYKLPDDFTGCVIAWNSNKLDSKVSGNYNYLYRLFRKSNLK<br>PFERDISTEIYQAGNKPCNGVAGFNCYFPLRSYSFRPTYGVGHQPYRVVVLSFELLH<br>APATVCGPKKSTNLVKNKCVNFNFNGLKGTGVLTESNKKFLPFQQFGRDIADTTDA<br>VRDPQTLEILDITPCSFGGVSVITPGTNTSNQVAVLYQGVNCTEVPVAIHADQLTPT<br>WRVYSTGSNVFQTRAGCLIGAEYVNNSYECDIPIGAGICASYQTQTKSHRRARSVA<br>SQSIIAYTMSLGAENSVAYSNNSIAIPTNFTISVTTEILPVSMTKTSVDCTMYICGDST<br>ECSNLLLQYGSFCTQLKRALTGIAVEQDKNTQEVFAQVKQIYKTPPIKYFGGFNFSQI

			LPDPSKPSKRSFIEDLLFNKVTLADAGFIKQYGDCLGDIAARDLICAQKFKGLTVLPPL<br>LTDEMIAQYTSALLAGTITSGWTFGAGAALQIPFAMQMAYRFNGIGVTQNVLYEN<br>QKLIANQFNSAIGKIQDSLSSTASALGKLQDVVNHNAQALNTLVKQLSSKFGAISSV<br>LNDIFSRLDKVEAEVQIDRLITGRLQSLQTYVTQQLIRAAEIRASANLAATKMSECVL<br>GQSKRVDFCGKGYHLMSFPQSAPHGVVFLHVTYVPAQEKNFTTAPAICHDGKAHF<br>PREGVFVSNGTHWFVTQRNFYEPQIITTDNTFVSGNCDVVIGIVNNTVYDPLQPEL<br>DSFKEELDKYFKNHTSPDVDLGDISGINASVVNIQKEIDRLNEVAKNLNESLIDLQEL<br>GKYEQYIKWPWYIWLGFIAGLIAIVMVTIMLCCMTSCCSCLKGCCSCGSCCKFDED<br>DSEPVLKGVKLHYTGSGSYPYDVPDYA
p492	Sars1-HA_p310bb	spike SARS-CoV WH20 (AAX16192.1)	MFIFLLFLTLTSGSDLDRCTTFDDVQAPNYTQHTSSMRGVYYPDEIFRSDTLYLTQD<br>LFLPFYSNVTGFHTINHTFDNPVIPFKDGIYFAATEKSNVVRGWVFGSTMNNKSQS<br>VIIINNSTNVVIRACNFELCDNPFFAVSKPMGTQTHTMIFDNAFNCTFEYISDAFSLD<br>VSEKSGNFKHLREFVFKNKDGFLYVYKGYQPIDVVRDLPSGFNTLKPIFKLPLGINIT<br>NFRAILTAFSPAQDTWGTSAAAYFVGYLKPTTFMLKYDENGTITDAVDCSQNPLAE<br>LKCSVKSFEIDKGIYQTSNFRVVPSGDVVRFPNITNLCPFGEVFNATKFPSVYAWER<br>KKISNCVADYSVLYNSTFFSTFKCYGVSATKLNDLCFSNVYADSFVVKGDDVRQIAP<br>GQTGVIADYNYKLPDDFMGCVLAWNTRNIDATSTGNYNYKYRYLRHGKLRPFERD<br>ISNVPFSPDGKPCTPPALNCYWPLNDYGFYTTTGIGYQPYRVVVLSFELLNAPATVC<br>GPKLSTDLIKNQCVNFNFNGLTGTGVLTPSSKRFQPFQQFGRDVSDFTDSVRDPKT<br>SEILDISPCSFGGVSVITPGTNASSEVAVLYQDVNCTDVSTAIHADQLTPAWRIYSTG<br>NNVFQTQAGCLIGAEHVDTSYECDIPIGAGICASYHTVSLLRSTSQKSIVAYTMSLGA<br>DSSIAYSNNTIAIPTNFSISITTEVMPVSMAKTSVDCNMYICGDSTECANLLLQYGSF<br>CTQLNRALSGIAAEQDRNTREVFAQVKQMYKTPTLKYFGGFNFSQILPDPLKPTKR<br>SFIEDLLFNKVTLADAGFMKQYGECLGDINARDLICAQKFNGLTVLPPLLTDDMIAA<br>YTAALVSGTATAGWTFGAGAALQIPFAMQMAYRFNGIGVTQNVLYENQKQIAN<br>QFNKAISQIQESLTTTSTALGKLQDVVNQNAQALNTLVKQLSSNFGAISSVLNDILS<br>RLDKVEAEVQIDRLITGRLQSLQTYVTQQLIRAAEIRASANLAATKMSECVLGQSKR<br>VDFCGKGYHLMSFPQAAPHGVVFLHVTYVPSQERNFTTAPAICHEGKAYFPREGV<br>FVFNGTSWFITQRNFFSPQIITTDNTFVSGNCDVVIGIINNTVYDPLQPELDSFKEEL<br>DKYFKNHTSPDVDLGDISGINASVVNIQKEIDRLNEVAKNLNESLIDLQELGKYEQYI<br>KWPWYVWLGFIAGLMAIVMVTILLCCMTSCCSCLKGACSCGSCCKFDEDDSEPVL<br>KGVKLHYTGSGSYPYDVPDYA

Supplemental Figure 1. M clustal aligment. Clustal O (version 1.2.4) multiple sequence alignment using the sarbecoviruses listed in Supplemental Table 2. The Wuhan-Hu-1 (NC_045512.2) sequence is indicated in bold, and selected antigens (Supplemental Table 3) are boxed and shaded.

```
GQ153542.1       -----MADSNGTITVEELKKLLEQWNLVIGFLFLTWICLLQFAYANRNRFLYIIKLIFLW      55
MN996529.1       -----MADSNGTITVEELKKLLEQWNLVIGFLFLTWICLLQFAYANRNRFLYIIKLIFLW      55
MT093571.1       -----MADSNGTITVEELKKLLEQWNLVIGFLFLTWICLLQFAYANRNRFLYIIKLIFLW      55
MN988713.1       -----MADSNGTITVEELKKLLEQWNLVIGFLFLTWICLLQFAYANRNRFLYIIKLIFLW      55
MT072688.1       -----MADSNGTITVEELKKLLEQWNLVIGFLFLTWICLLQFAYANRNRFLYIIKLIFLW      55
MN994467.1       -----MADSNGTITVEELKKLLEQWNLVIGFLFLTWICLLQFAYANRNRFLYIIKLIFLW      55
NC_045512.2      -----MADSNGTITVEELKKLLEQWNLVIGFLFLTWICLLQFAYANRNRFLYIIKLIFLW      55
MN996532.2       ------MADNGTITVEELKKLLEQWNLVIGFLFLTWICLLQFAYANRNRFLYIIKLIFLW      54
MG772933.1       -----MSGDNGTITVEELKKLLEQWNLVIGFLFLTWICLLQFAYANRNRFLYIIKLIFLW      55
MG772934.1       -----MSGDNGTITVEELKKLLEQWNLVIGFLFLTWICLLQFAYANRNRFLYIIKLIFLW      55
MW532698.1       -----MSANNGTITVEELKKLLEQWNLVIGFLFLTWICLLQFAYANRNRFLYIIKLIFLW      55
MT040336.1       -----MSADNGTITVEELKKLLEQWNLVIGFLFLTWICLLQFAYANRNRFLYIIKLIFLW      55
MT040335.1       -----MSADNGTITVEELKKLLEQWNLVIGFLFLTWICLLQFAYANRNRFLYIIKLIFLW      55
MT040334.1       -----MSADNGTITVEELKKLLEQWNLVIGFLFLTWICLLQFAYANRNRFLYIIKLIFLW      55
MT040333.1       -----MSADNGTITVEELKKLLEQWNLVIGFLFLTWICLLQFAYANRNRFLYIIKLIFLW      55
NC_014470.1      MTNSSASPPTETITVEQLKHLLEQWNLVIGFLFFAWILLLQFAYSNRNRFLYIIKLVFLW      60
KP886809.1       ------MADYGTITVEELKQLLEQWNLLIGFLFLPWIMLLQFAYSNRNRFLHIIKLVFLW      54
KF569996.1       ------MAENGTISVEELKRLLEQWNLVIGFIFLAWIMLLQFAYSNRNRFLYIIKLVFLW      54
KY417147.1       ------MAENGTISVEELKRLLEQWNLVIGFLFLAWIMLLQFAYSNRNRFLYIIKLVFLW      54
KY417142.1       ------MAENGTISVEELKRLLEQWNLVIGFLFLAWIMLLQFAYSNRNRFLYIIKLVFLW      54
KC881006.1       ------MAENGTISVEELKRLLEQWNLVIGFLFLAWIMLLQFAYSNRNRFLYIIKLVFLW      54
KF367457.1       ------MAENGTISVEELKRLLEQWNLVIGFLFLAWIMLLQFAYSNRNRFLYIIKLVFLW      54
KY417144.1       ------MAENGTISVEELKRLLEQWNLVIGFLFLAWIMLLQFAYSNRNRFLYIIKLVFLW      54
KC881005.1       ------MAENGTISVEELKRLLEQWNLVIGFLFLAWIMLLQFAYSNRNRFLYIIKLVFLW      54
KY417152.1       ------MAENGTISVEELKRLLEQWNLVIGFLFLAWIMLLQFAYSNRNRFLYIIKLVFLW      54
KT444582.1       ------MAENGTISVEELKRLLEQWNLVIGFLFLAWIMLLQFAYSNRNRFLYIIKLVFLW      54
KY417151.1       ------MAENGTISVEELKRLLEQWNLVIGFLFLAWIMLLQFAYSNRNRFLYIIKLVFLW      54
KY417143.1       ------MAENGTISVEELKRLLEQWNLVIGFLFLAWIMLLQFAYSNRNRFLYIIKLVFLW      54
FJ588686.1       ------MAENGTISVEELKRLLEQWNLVIGFLFLAWIMLLQFAYSNRNRFLYIIKLVFLW      54
DQ022305         ------MADNGTITVEELKQLLEQWNLVIGFIFLAWIMLLQFAYSNRNRFLYIIKLVFLW      54
KY417146.1       ------MADNGTITVEELKQLLEQWNLVIGFLFLAWIMLLQFAYSNRNRFLYVIKLVFLW      54
KY417148.1       ------MADNGTITVEELKQLLEQWNLVIGFLFLAWIMLLQFAYSNRNRFLYIIKLVFLW      54
AY572038.1       ------MADNSTITVEELKQLLEQWNLVIGFLFLAWIMLLQFAYSNRNRFLYIIKLVFLW      54
AY572034.1       ------MADNSTITVEELKQLLEQWNLVIGFLFLAWIMLLQFAYSNRNRFLYIIKLVFLW      54
AP006560.1       ------MADNGTITVEELKQLLEQWNLVIGFLCLAWIMLLQFAYSNRNRFLYIIKLVFLW      54
AP006557.1       ------MADNGTITVEELKQLLEQWNLVIGFLCLAWIMLLQFAYSNRNRFLYIIKLVFLW      54
AY485277.1       ------MADNGTITVEELKQLLEQWNLVIGFLCLAWIMLLQFAYSNRNRFLYIIKLVFLW      54
AY278488.2       ------MADNGTITVEELKQLLEQWNLVIGFLFLAWIMLLQFAYSNRNRFLYIIKLVFLW      54
KY417145.1       ------MADNGTITVEELKQLLEQWNLVIGFLFLAWIMLLQFAYSNRNRFLYIIKLVFLW      54
AY274119         ------MADNGTITVEELKQLLEQWNLVIGFLFLAWIMLLQFAYSNRNRFLYIIKLVFLW      54
                            **:**:**:*******:***: : ** ******:******::***:***
```

CoVAX_MNS#1

```
GQ153542.1     LLWPVTLACFVLAAVYRINWITGGIAIAMACLVGLMWLSYFIASFRLFARTRSMWSFNPE     115
MN996529.1     LLWPVTLACFVLAAVYRINWITGGIAIAMACLVGLMWLSYFIASFRLFARTRSMWSFNPE     115
MT093571.1     LLWPVTLACFVLAAVYRINWITGGIAIAMACLVGLMWLSYFIASFRLFARTRSMWSFNPE     115
MN988713.1     LLWPVTLACFVLAAVYRINWITGGIAIAMACLVGLMWLSYFIASFRLFARTRSMWSFNPE     115
MT072688.1     LLWPVTLACFVLAAVYRINWITGGIAIAMACLVGLMWLSYFIASFRLFARTRSMWSFNPE     115
MN994467.1     LLWPVTLACFVLAAVYRINWITGGIAIAMACLVGLMWLSYFIASFRLFARTRSMWSFNPE     115
NC_045512.2    LLWPVTLACFVLAAVYRINWITGGIAIAMACLVGLMWLSYFIASFRLFARTRSMWSFNPE     115
MN996532.2     LLWPVTLACFVLAAVYRINWITGGIAIAMACLVGLMWLSYFIASFRLFARTRSMWSFNPE     114
MG772933.1     LLWPVTLACFVLAAVYRINWITGGIAIAMACLVGLMWLSYFIASFRLFARTRSMWSFNPE     115
MG772934.1     LLWPVTLACFVLAAVYRINWITGGIAIAMACLVGLMWLSYFIASFRLFARTRSMWSFNPE     115
MW532698.1     LLWPVTLACFVLAAVYRINWITGGIAIAMTCLVGLMWLSYFIASFRLFARTRSMWSFNPE     115
MT040336.1     LLWPVTLACFVLAAVYRINWITGGIAIAMACLVGLMWLSYFIASFRLFARTRSMWSFNPE     115
MT040335.1     LLWPVTLACFVLAAVYRINWITGGIAIAMACLVGLMWLSYFIASFRLFARTRSMWSFNPE     115
MT040334.1     LLWPVTLACFVLAAVYRINWITGGIAIAMACLVGLMWLSYFIASFRLFARTRSMWSFNPE     115
MT040333.1     LLWPVTLACFVLAAVYRINWITGGIAIAMACLVGLMWLSYFIASFRLFARTRSMWSFNPE     115
NC_014470.1    LLWPITLACFVLAAVYRINWVTGGIAIAMACIVGLMWLSYFVASFRLFARTRSWWSFNPE     120
KP886809.1     LLWPVTLACFVLAAVYRINWVTGGIAIAMACIVGLMWLSYFVASFRLFARTRSMWSFNPE     114
KF569996.1     LLWPVTLACFVLAAVYRINWVTGGIAIAMACIVGLMWLSYFVASFRLFARTRSMWSFNPE     114
KY417147.1     LLWPVTLACFVLAAVYRINWVTGGIAIAMACIVGLMWLSYFVASFRLFARTRSMWSFNPE     114
KY417142.1     LLWPVTLACFVLAAVYRINWVTGGIAIAMACIVGLMWLSYFVASFRLFARTRSMWSFNPE     114
KC881006.1     LLWPVTLACFVLAAVYRINWVTGGIAIAMACIVGLMWLSYFIASFRLFARTRSMWSFNPE     114
KF367457.1     LLWPVTLACFVLAAVYRINWVTGGIAIAMACIVGLMWLSYFIASFRLFARTRSMWSFNPE     114
KY417144.1     LLWPVTLACFVLAAVYRINWVTGGIAIAMACIVGLMWLSYFIASFRLFARTRSMWSFNPE     114
KC881005.1     LLWPVTLACFVLAAVYRINWVTGGIAIAMACIVGLMWLSYFIASFRLFARTRSMWSFNPE     114
KY417152.1     LLWPVTLACFVLAAVYRINWVTGGIAIAMACIVGLMWLSYFIASFRLFARTRSMWSFNPE     114
KT444582.1     LLWPVTLACFVLAAVYRINWVTGGIAIAMACIVGLMWLSYFIASFRLFARTRSMWSFNPE     114
KY417151.1     LLWPVTLACFVLAAVYRINWVTGGIAIAMACIVGLMWLSYFVASFRLFARTRSMWSFNPE     114
KY417143.1     LLWPVTLACFVLAAVYRINWVTGGIAIAMACIVGLMWLSYFVASFRLFARTRSMWSFNPE     114
FJ588686.1     LLWPVTLACFVLAAVYRINWVTGGIAIAMACIVGLMWLSYFVASFRLFARTRSMWSFNPE     114
DQ022305       LLWPVTLACFVLAAVYRINWVTGGIAIAMACIVGLMWLSYFVASFRLFARTRSMWSFNPE     114
KY417146.1     LLWPVTLACFVLAAVYRINWVTGGIAIAMACIVGLMWLSYFVASFRLFARTRSMWSFNPE     114
KY417148.1     LLWPVTLACFVLAAVYRINWVTGGIAIAMACIVGLMWLSYFVASFRLFARTRSMWSFNPE     114
AY572038.1     LLWPVTLACFVLAAVYRINWVTGGIAIAMACIVGLMWLSYFVASFRLFARTRSMWSFNPE     114
AY572034.1     LLWPVTLACFVLAAVYRINWVTGGIAIAMACIVGLMWLSYFVASFRLFARTRSMWSFNPE     114
AP006560.1     LLWPVTLACFVLAAVYRINWVTGGIAIAMACIVGLMWLSYFVASFRLFARTRSMWSFNPE     114
AP006557.1     LLWPVTLACFVLAAVYRINWVTGGIAIAMACIVGLMWLSYFVASFRLFARTRSMWSFNPE     114
AY485277.1     LLWPVTLACFVLAAVYRINWVTGGIAIAMACIVGLMWLSYFVASFRLFARTRSMWSFNPE     114
AY278488.2     LLWPVTLACFVLAAVYRINWVTGGIAIAMACIVGLMWLSYFVASFRLFARTRSMWSFNPE     114
KY417145.1     LLWPVTLACFVLAAVYRINWVTGGIAIAMACIVGLMWLSYFVASFRLFARTRSMWSFNPE     114
AY274119       LLWPVTLACFVLAAVYRINWVTGGIAIAMACIVGLMWLSYFVASFRLFARTRSMWSFNPE     114
               ****:***************:********:*:*********:*********** ******
```

CoVAX_MNS#1 CoVAX_MNS#6

```
GQ153542.1     TNILLNVPLHGTILTRPLLESELVIGAVILRGHLRIAGHHLGRCDIKDLPKEITVATSRT     175
MN996529.1     TNILLNVPLHGTILTRPLLESELVIGAVILRGHLRIAGHHLGRCDIKDLPKEITVATSRT     175
MT093571.1     TNILLNVPLHGTILTRPLLESELVIGAVILRGHLRIAGHHLGRCDIKDLPKEITVATSRT     175
MN988713.1     TNILLNVPLHGTILTRPLLESELVIGAVILRGHLRIAGHHLGRCDIKDLPKEITVATSRT     175
MT072688.1     TNILLNVPLHGTILTRPLLESELVIGAVILRGHLRIAGHHLGRCDIKDLPKEITVATSRT     175
MN994467.1     TNILLNVPLHGTILTRPLLESELVIGAVILRGHLRIAGHHLGRCDIKDLPKEITVATSRT     175
NC_045512.2    TNILLNVPLHGTILTRPLLESELVIGAVILRGHLRIAGHHLGRCDIKDLPKEITVATSRT     175
MN996532.2     TNILLNVPLHGTILTRPLLESELVIGAVILRGHLRIAGHHLGRCDIKDLPKEITVATSRT     174
MG772933.1     TNILLNVPLHGTILTRPLLESELVIGAVILRGHLRIAGHHLGRCDIKDLPKEITVATSRT     175
MG772934.1     TNILLNVPLHGTILTRPLLESELVIGAVILRGHLRIAGHHLGRCDIKDLPKEITVATSRT     175
MW532698.1     TNILLNVPLHGTILTRPLLESELVIGAVILRGHLRIAGHHLGRCDIKDLPKEITVATSRT     175
MT040336.1     TNILLNVPLHGTILTRPLLESELVIGAVILRGHLRIAGHHLGRCDIKDLPKEITVATSRT     175
MT040335.1     TNILLNVPLHGTILTRPLLESELVIGAVILRGHLRIAGHHLGRCDIKDLPKEITVATSRT     175
MT040334.1     TNILLNVPLHGTILTRPLLESELVIGAVILRGHLRIAGHHLGRCDIKDLPKEITVATSRT     175
MT040333.1     TNILLNVPLHGTILTRPLLESELVIGAVILRGHLRIAGHHLGRCDIKDLPKEITVATSRT     175
NC_014470.1    TNILLNVPLRGTILTRPLLESELVIGAVIIRGHLRMAGHSLGRCDIKDLPKEITVATSRT     180
KP886809.1     TNILLNVPLRGTILTRPLMESELVIGAVIIRGHLRMAGHSLGRCDIKDLPKEITVATSRT     174
KF569996.1     TNILLNVPLRGTILTRPLMESELVIGAVIIRGHLRMAGHSLGRCDIKDLPKEITVATSRT     174
KY417147.1     TNILLNVPLRGTIVTRPLLESELVIGAVIIRGHLRMAGHSLGRCDIKDLPKEITVATSRT     174
KY417142.1     TNILLNVPLRGTIVTRPLLESELVIGAVIIRGHLRMAGHSLGRCDIKDLPKEITVATSRT     174
KC881006.1     TNILLNVPLRGTIVTRPLMESELVIGAVIIRGHLRMAGHSLGRCDIKDLPKEITVATSRT     174
KF367457.1     TNILLNVPLRGTIVTRPLMESELVIGAVIIRGHLRMAGHSLGRCDIKDLPKEITVATSRT     174
KY417144.1     TNILLNVPLRGTIVTRPLMESELVIGAVIIRGHLRMAGHSLGRCDIKDLPKEITVATSRT     174
KC881005.1     TNILLNVPLRGTIVTRPLMESELVIGAVIIRGHLRMAGHSLGRCDIKDLPKEITVATSRT     174
KY417152.1     TNILLNVPLRGTIVTRPLMESELVIGAVIIRGHLRMAGHSLGRCDIKDLPKEITVATSRT     174
KT444582.1     TNILLNVPLRGTIVTRPLMESELVIGAVIIRGHLRMAGHSLGRCDIKDLPKEITVATSRT     174
KY417151.1     TNILLNVPLRGTIVTRPLMESELVIGAVIIRGHLRMAGHSLGRCDIKDLPKEITVATSRT     174
KY417143.1     TNILLNVPLRGTIVTRPLMESELVIGAVIIRGHLRMAGHSLGRCDIKDLPKEITVATSRT     174
FJ588686.1     TNILLNVPLRGTIVTRPLMESELVIGAVIIRGHLRMAGHSLGRCDIKDLPKEITVATSRT     174
DQ022305       TNILLNVPLRGTILTRPLMESELVIGAVIIRGHLRMAGHSLGRCDIKDLPKEITVATSRT     174
KY417146.1     TNILLNVPLRGTIVTRPLMESELVIGAVIIRGHLRMAGHSLGRCDIKDLPKEITVATSRT     174
KY417148.1     TNILLNVPLRGTIVTRPLLESELVIGAVIIRGHLRMAGHSLGRCDIKDLPKEITVATSRT     174
AY572038.1     TNILLNVPLRGTIVTRPLMESELVIGAVIIRGHLRMAGHSLGRCDIKDLPKEITVATSRT     174
AY572034.1     TNILLNVPLRGTIVTRPLMESELVIGAVIIRGHLRMAGHSLGRCDIKDLPKEITVATSRT     174
AP006560.1     TNILLNVPLRGTIVTRPLMESELVIGAVIIRGHLRMAGHSLGRCDIKDLPKEITVATSRT     174
AP006557.1     TNILLNVPLRGTIVTRPLMESELVIGAVIIRGHLRMAGHSLGRCDIKDLPKEITVATSRT     174
AY485277.1     TNILLNVPLRGTIVTRPLMESELVIGAVIIRGHLRMAGHSLGRCDIKDLPKEITVATSRT     174
AY278488.2     TNILLNVPLRGTIVTRPLMESELVIGAVIIRGHLRMAGHSLGRCDIKDLPKEITVATSRT     174
KY417145.1     TNILLNVPLRGTIVTRPLMESELVIGAVIIRGHLRMAGHSLGRCDIKDLPKEITVATSRT     174
AY274119       TNILLNVPLRGTIVTRPLMESELVIGAVIIRGHLRMAGHSLGRCDIKDLPKEITVATSRT     174
               *********:***:****:**********:*****:*** ********************
```

CoVAX_MNS#6 CoVAX_MNS#9

```
GQ153542.1     LSYYKLGASQRVAGDSGFAAYSRYRIGNYKLNTDHSSSSDNIALLVQ    222
MN996529.1     LSYYKLGASQRVAGDSGFAAYSRYRIGNYKLNTDHSSSSDNIALLVQ    222
MT093571.1     LSYYKLGASQRVAGDSGFAAYSRYRIGNYKLNTDHSSSSDNIALLVQ    222
MN988713.1     LSYYKLGASQRVAGDSGFAAYSRYRIGNYKLNTDHSSSSDNIALLVQ    222
MT072688.1     LSYYKLGASQRVAGDSGFAAYSRYRIGNYKLNTDHSSSSDNIALLVQ    222
MN994467.1     LSYYKLGASQRVAGDSGFAAYSRYRIGNYKLNTDHSSSSDNIALLVQ    222
NC_045512.2    LSYYKLGASQRVAGDSGFAAYSRYRIGNYKLNTDHSSSSDNIALLVQ    222
MN996532.2     LSYYKLGASQRVAGDSGFAAYSRYRIGNYKLNTDHSSSSDNIALLVQ    221
MG772933.1     LSYYKLGASQRVAGDSGFAAYSRYRIGNYKLNTDHSSSSDNIALLVQ    222
MG772934.1     LSYYKLGASQRVAGDSGFAAYSRYRIGNYKLNTDHSSSSDNIALLVQ    222
MW532698.1     LSYYKLGASQRVAGDSGFAAYSRYRIGNYKLNTDHSNSSDNIALLVQ    222
MT040336.1     LSYYKLGASQRVAGDSGFAAYSRYRIGNYKLNTDHSNSSDNIALLVQ    222
MT040335.1     LSYYKLGASQRVAGDSGFAAYSRYRIGNYKLNTDHSNSSDNIALLVQ    222
MT040334.1     LSYYKLGASQRVAGDSGFAAYSRYRIGNYKLNTDHSNSSDNIALLVQ    222
MT040333.1     LSYYKLGASQRVAGDSGFAAYSRYRIGNYKLNTDHSNSSDNIALLVQ    222
NC_014470.1    LSYYRLGASQRVASDSGFAVYHRYRIGNYKLNTDHIGSDDNIALLVQ    227
KP886809.1     LSYYKLGASQRVGTDSGFAAYNRYRIGNYKLNTDHAGSNDNIALLVQ    221
KF569996.1     LSYYKLGASQRVGTDSGFAAYNRYRIGNYKLNTDHSGSNDNIALLVQ    221
KY417147.1     LSYYKLGASQRVGTDSGFAAYNRYRIGNYKLNTDHAGSNDNIALLVQ    221
KY417142.1     LSYYKLGASQRVGTDSGFAAYNRYRIGNYKLNTDHAGSNDNIALLVQ    221
KC881006.1     LSYYKLGASQRVGTDSGFAAYNRYRIGNYKLNTDHAGSNDNIALLVQ    221
KF367457.1     LSYYKLGASQRVGTDSGFAAYNRYRIGNYKLNTDHAGSNDNIALLVQ    221
KY417144.1     LSYYKLGASQRVGTDSGFAAYNRYRIGNYKLNTDHAGSNDNIALLVQ    221
KC881005.1     LSYYKLGASQRVGTDSGFAAYNRYRIGNYKLNTDHAGSNDNIALLVQ    221
KY417152.1     LSYYKLGASQRVGTDSGFAAYNRYRIGNYKLNTDHAGSNDNIALLVQ    221
KT444582.1     LSYYKLGASQRVGTDSGFAAYNRYRIGNYKLNTDHAGSNDNIALLVQ    221
KY417151.1     LSYYKLGASQRVGTDSGFAAYNRYRIGNYKLNTDHAGSNDNIALLVQ    221
KY417143.1     LSYYKLGASQRVGTDSGFAAYNRYRIGNYKLNTDHAGSNDNIALLVQ    221
FJ588686.1     LSYYKLGASQRVGTDSGFAAYNRYRIGNYKLNTDHAGSNDNIALLVQ    221
DQ022305       LSYYKLGASQRVGTDSGFAAYNRYRIGNYKLNTDHSGSNDNIALLVQ    221
KY417146.1     LSYYKLGASQRVGIDSGFAAYNRYRIGNYKLNTDHAGSNDNIALLVQ    221
KY417148.1     LSYYKLGASQRVGTDSGFAAYNRYRIGNYKLNTDHAGSNDNIALLVQ    221
AY572038.1     LSYYKLGASQRVGTDSGFAAYNRYRIGNYKLNTDHAGSNDNIALLVQ    221
AY572034.1     LSYYKLGASQRVGTDSGFAAYNRYRIGNYKLNTDHAGSNDNIALLVQ    221
AP006560.1     LSYYKLGASQRVGTDSGFAAYNRYRIGNYKLNTDHAGSNDNIALLVQ    221
AP006557.1     LSYYKLGASQRVGTDSGFAAYNRYRIGNYKLNTDHAGSNDNIALLVQ    221
AY485277.1     LSYYKLGASQRVGTDSGFAAYNRYRIGNYKLNTDHAGSNDNIALLVQ    221
AY278488.2     LSYYKLGASQRVGTDSGFAAYNRYRIGNYKLNTDHAGSNDNIALLVQ    221
KY417145.1     LSYYKLGASQRVGTDSGFAAYNRYRIGNYKLNTDHAGSNDNIALLVQ    221
AY274119       LSYYKLGASQRVGTDSGFAAYNRYRIGNYKLNTDHAGSNDNIALLVQ    221
               ****:*******. *****.* ************* .*.********
```

CoVAX_MNS#9

Supplemental Figure 2. N clustal aligment. Clustal O (version 1.2.4) multiple sequence alignment using the sarbecoviruses listed in Supplemental Table 2. The Wuhan-Hu-1 (NC_045512.2) sequence is indicated in bold, and selected antigens (Supplemental Table 3) are boxed and shaded.

```
NC_014470.1      MTDNGQ-SNSRNAPRITFGV-SDTSDNNQNAERAGARPKQRRPQGPPNNTASWFTALTQH    58
KP886809.1       MSDNGPH-NQRSASRITFGGPTDSTDNNQNGGRNGARPKQRRPQGLPNNTASWFTALTQH    59
DQ022305         MSDNGPQ-SQRSAPRITFGGPADSNDNNQDGGRSGARPKQRRPQGLPNNTASWFTALTQH    59
KF569996.1       MSDNGPQQNQRSAPRITFGGPTDSADNNQNGERSGARPKQRRPQGLPNNTASWFTALTQH    60
GQ153542.1       MSDNGPQ-DQRSAPRITFGGPTDSTDNNQDGGRSGARPKQRRPQGLPNNTASWFTALTQH    59
FJ588686.1       MSDNGPQPNQRSAPRITFGGPTDSTDNNQDGGRSGARPKQRRPQGLPNNTASWFTALTQH    60
KY417151.1       MSDNGPQSNQRSAPRITFGGPTDSTDNNQNGGRNGARSKQRRPQGLPNNTASWFTALTQH    60
KY417145.1       MSDNGPQSNQRSAPRITFGGPTDSTDNNQNGGRNGARSKQRRPQGLPNNTASWFTALTQH    60
AY572038.1       MSDNGPQSNQRSAPRITFGGPIDSTDNNQNGGRNGARPKQRRPQGLPNNTASWFTALTQH    60
KT444582.1       MSDNGPQPNQRSAPRITFGGPTDSIDNNQNGGRNGARPKQRRPQGLPNNTASWFTALTQH    60
KY417143.1       MSDNGPQPNQRSAPRITFGGPTDPTDNNQNGGRNGARPKQRRPQGLPNNTASWFTALTQH    60
KY417152.1       MSDNGPQPNQRSAPRITFGGPTDSTDNNQNGGRNGARPKQRRPQGLPNNTASWFTALTQH    60
KC881005.1       MSDNGPQPNQRSAPRITFGGPTDPTDNNQNGGRNGARPKQRRPQGLPNNTASWFTALTQH    60
KY417146.1       MSDNGPQPNQRSAPRITFGGPTDSTDNNQNGGRNGARPKQRRPQGLPNNTASWFTALTQH    60
KY417148.1       MSDNGPQPNQRSAPRITFGGPTDSTDNNQNGGRNGARPKQRRPQGLPNNTASWFTALTQH    60
KY417142.1       MSDNGPQSNQRSAPRITFGGPTDSTDNNQNGGRNGARPKQRRPQGLPNNTASWFTALTQH    60
KY417144.1       MSDNGPQSNQRSAPRITFGGPTDSTDNNQNGGRNGARPKQRRPQGLPNNTASWFTALTQH    60
AY572034.1       MSDNGPQSNQRSAPRITFGGPTDSTDNNQNGGRNGARPKQRRPQGLPNNTASWFTALTQH    60
KY417147.1       MSDNGPQSNQRSAPRITFGGPTDSTDNNQNGGRNGARPKQRRPQGLPNNTASWFTALTQH    60
AP006560.1       MSDNGPQSNQRSAPRITFGGPTDSTDNNQNGGRNGARPKQRRPQGLPNNTASWFTALTQH    60
AP006557.1       MSDNGPQSNQRSAPRITFGGPTDSTDNNQNGGRNGARPKQRRPQGLPNNTASWFTALTQH    60
AY485277.1       MSDNGPQSNQRSAPRITFGGPTDSTDNNQNGGRNGARPKQRRPQGLPNNTASWFTALTQH    60
AY278488.2       MSDNGPQSNQRSAPRITFGGPTDSTDNNQNGGRNGARPKQRRPQGLPNNTASWFTALTQH    60
KC881006.1       MSDNGPQSNQRSAPRITFGGPTDSTDNNQNGGRNGARPKQRRPQGLPNNTASWFTALTQH    60
AY274119         MSDNGPQSNQRSAPRITFGGPTDSTDNNQNGGRNGARPKQRRPQGLPNNTASWFTALTQH    60
KF367457.1       MSDNGPQSNQRSAPRITFGGPTDSTDNNQNGGRNGARPKQRRPQGLPNNTASWFTALTQH    60
MT040336.1       MSDNGPQ-N--RAPRITFGGPSDSTDNNQNGDRSGARPKQRRPQGLPNSTASWFTALTQH    57
MT040333.1       MSDNGPQ-N--RAPRITFGGPSDSTDNNQNGDRSGARPKQRRPQGLPNNTASWFTALTQH    57
MT040335.1       MSDNGPQ-N--RAPRITFGGPSDSTDNNQNGDRSGARPKQRRPQGLPNNTASWFTALTQH    57
MW532698.1       MSDNGPQ-N--RAPRITFGGPSDSTDNNQNGDRSGARPKQRRPQGLPNNTASWFTALTQH    57
MT040334.1       MSDNGPQ-N--RAPRITFGGPSDSTDNNQNGDRSGARPKQRRPQGLPNNTASWFTALTQH    57
MG772933.1       MSDNGPQ-NQRSAPRITFGGPSDSSDNSKNGERNGARPKQRRPQGLPNNTASWFTALTQH    59
MG772934.1       MSDNGPQ-NQSSAPRITFGGPSDSSDNSQNGERNGARPKQRRPQGLPNNTASWFTALTQH    59
MN996532.2       MSDNGPQ-NQRNAPRITFGGPSDSTGSNQNGERSGARPKQRRPQGLPNNTASWFTALTQH    59
MN996529.1       MSDNGPQ-NQRNAPRITFGGPSDSTGSNQNGERSGARSKQRRPQGLPNNTASWFTALTQH    59
MT093571.1       MSDNGPQ-NQRNAPRITFGGPSDSTGSNQNGERSGARSKQRRPQGLPNNTASWFTALTQH    59
MT072688.1       MSDNGPQ-NQRNAPRITFGGPSDSTGSNQNGERSGARSKQRRPQGLPNNTASWFTALTQH    59
MN994467.1       MSDNGPQ-NQRNAPRITFGGPSDSTGSNQNGERSGARSKQRRPQGLPNNTASWFTALTQH    59
NC_045512.2      MSDNGPQ-NQRNAPRITFGGPSDSTGSNQNGERSGARSKQRRPQGLPNNTASWFTALTQH    59
MN988713.1       MSDNGPQ-NQRNAPRITFGGPSDSTGSNQNGERSGARSKQRRPQGLPNNTASWFTALTQH    59
                 *:***   .   * *****   *  ...::. * *** ******* **.***********
```

CoVAX_MNS#14

```
NC_014470.1      GKEGLSFPRGQGVPVNTNSTRDDQIGYYRRATRRVRGGDGKMKELSPRWYFYYLGTGPEA   118
KP886809.1       GKEELRFPQGQGVPINTNSGPDDQIGYYRRATRRVRGGDGKMKELSPRWYFYYLGTGPEA   119
DQ022305         GKEELRFPRGQGVPINTNSGKDDQIGYYRRATRRVRGGDGKMKELSPRWYFYYLGTGPEA   119
KF569996.1       GKEELRFPRGQGVPINTNSGTDDQIGYYRRATRRVRGGDGKMKELSPRWYFYYLGTGPEA   120
GQ153542.1       GKEELRFPRGQGVPINTNSGKDDQIGYYRRATRRVRGGDGKMKELSPRWYFYYLGTGPEA   119
FJ588686.1       GKEELRFPRGQGVPINTNSGKDDQIGYYRRATRRVRGGDGKMKELSPRWYFYYLGTGPEA   120
KY417151.1       GKEELRFPRGQGVPINTNSGPDDQIGYYRRATRRVRGGDGKMKELSPRWYFYYLGTGPEA   120
KY417145.1       GKEELRFPRGQGVPINTNSGPDDQIGYYRRATRRVRGGDGKMKELSPRWYFYYLGTGPEA   120
AY572038.1       GKEELRFPRGQGVPINTNSGPDDQIGYYRRATRRVRGGDGKMKELSPRWYFYYLGTGPEA   120
KT444582.1       GKEELRFPRGQGVPINTNSGPDDQIGYYRRATRRVRGGDGKMKELSPRWYFYYLGTGPEA   120
KY417143.1       GKEELRFPRGQGVPINTNSGPDDQIGYYRRATRRVRGGDGKMKELSPRWYFYYLGTGPEA   120
KY417152.1       GKEELRFPRGQGVPINTNSGPDDQIGYYRRATRRVRGGDGKMKELSPRWYFYYLGTGPEA   120
KC881005.1       GKEELRFPRGQGVPINTNSGPDDQIGYYRRATRRVRGGDGKMKELSPRWYFYYLGTGPEA   120
KY417146.1       GKEELRFPRGQGVPINTNSGPDDQIGYYRRATRRVRGGDGKMKELSPRWYFYYLGTGPEA   120
KY417148.1       GKEELRFPRGQGVPINTNSGPDDQIGYYRRATRRVRGGDGKMKELSPRWYFYYLGTGPEA   120
KY417142.1       GKEELRFPRGQGVPINTNSGPDDQIGYYRRATRRVRGGDGKMKELSPRWYFYYLGTGPEA   120
KY417144.1       GKEELRFPRGQGVPINTNSGPDDQIGYYRRATRRVRGGDGKMKELSPRWYFYYLGTGPEA   120
AY572034.1       GKEELRFPRGQGVPINTNSGPDDQIGYYRRATRRVRGGDGKMKELSPRWYFYYLGTGPEA   120
KY417147.1       GKEELRFPRGQGVPINTNSGPDDQIGYYRRATRRVRGGDGKMKELSPRWYFYYLGTGPEA   120
AP006560.1       GKEELRFPRGQGVPINTNSGPDDQIGYYRRATRRVRGGDGKMKELSPRWYFYYLGTGPEA   120
AP006557.1       GKEELRFPRGQGVPINTNSGPDDQIGYYRRATRRVRGGDGKMKELSPRWYFYYLGTGPEA   120
AY485277.1       GKEELRFPRGQGVPINTNSGPDDQIGYYRRATRRVRGGDGKMKELSPRWYFYYLGTGPEA   120
AY278488.2       GKEELRFPRGQGVPINTNSGPDDQIGYYRRATRRVRGGDGKMKELSPRWYFYYLGTGPEA   120
KC881006.1       GKEELRFPRGQGVPINTNSGPDDQIGYYRRATRRVRGGDGKMKELSPRWYFYYLGTGPEA   120
AY274119         GKEELRFPRGQGVPINTNSGPDDQIGYYRRATRRVRGGDGKMKELSPRWYFYYLGTGPEA   120
KF367457.1       GKEELRFPRGQGVPINTNSGPDDQIGYYRRATRRVRGGDGKMKELSPRWYFYYLGTGPEA   120
MT040336.1       GKEDLRFPRGQGVPINTNSTKDDQIGYYRRATRRVRGGDGKMKDLSPRWYFYYLGTGPEA   117
MT040333.1       GKEDLRFPRGQGVPINTNSTKDDQIGYYRRATRRVRGGDGKMKDLSPRWYFYYLGTGPEA   117
MT040335.1       GKEDLRFPRGQGVPINTNSTKDDQIGYYRRATRRVRGGDGKMKDLSPRWYFYYLGTGPEA   117
MW532698.1       GKEDLRFPRGQGVPINTNSTKDDQIGYYRRATRRVRGGDGKMKDLSPRWYFYYLGTGPEA   117
MT040334.1       GKEDLRFPRGQGVPINTNSTKDDQIGYYRRATRRVRGGDGKMKDLSPRWYFYYLGTGPEA   117
MG772933.1       GKENLTFPRGQGVPINTNSSKDDQIGYYRRATRRIRGGDGKMKELSPRWYFYYLGTGPEA   119
MG772934.1       GKENLTFPRGQGVPINTNSSKDDQIGYYRRATRRIRGGDGKMKELSPRWYFYYLGTGPEA   119
MN996532.2       GKEDLKFPRGQGVPINTNSSPDDQIGYYRRATRRIRGGDGKMKDLSPRWYFYYLGTGPEA   119
MN996529.1       GKEDLKFPRGQGVPINTNSSPDDQIGYYRRATRRIRGGDGKMKDLSPRWYFYYLGTGPEA   119
MT093571.1       GKEDLKFPRGQGVPINTNSSPDDQIGYYRRATRRIRGGDGKMKDLSPRWYFYYLGTGPEA   119
MT072688.1       GKEDLKFPRGQGVPINTNSSPDDQIGYYRRATRRIRGGDGKMKDLSPRWYFYYLGTGPEA   119
MN994467.1       GKEDLKFPRGQGVPINTNSSPDDQIGYYRRATRRIRGGDGKMKDLSPRWYFYYLGTGPEA   119
NC_045512.2      GKEDLKFPRGQGVPINTNSSPDDQIGYYRRATRRIRGGDGKMKDLSPRWYFYYLGTGPEA   119
MN988713.1       GKEDLKFPRGQGVPINTNSSPDDQIGYYRRATRRIRGGDGKMKDLSPRWYFYYLGTGPEA   119
                 *** * **:*****:****  *************:********:****************
```

CoVAX_MNS#14

```
NC_014470.1      ALPYGANKDGIVWVATEGALNTPKDHIGTRNPNNNAAIVIQLPQGTTLPKGFYAEGSRGG   178
KP886809.1       SLPYGANKEGIIWVATEGALNTPKDHIGTRNPNNNAATVLQLPQGTTLPKGFYAEGSRGG   179
DQ022305         SLPYGANKEGIVWVATEGALNTPKDHIGTRNPNNNAAIVLQLPQGTTLPKGFYAEGSRGG   179
KF569996.1       SLPYGANKEGIVWVATEGALNTPKDHIGTRNPNNNAAIVLQLPQGTTLPKGFYAEGSRGG   180
GQ153542.1       SLPYGANKEGIVWVATEGALNTPKDHIGTRNPNNNAAIVLQLPQGTTLPKGFYAEGSRGG   179
FJ588686.1       SLPYGANKQGIVWVATEGALNTPKDHIGTRNPNNNAAIVLQLPQGTTLPKGFYAEGSRGG   180
KY417151.1       SLLYGANKEGIVWVATEGALNTPKDHIGTRNPNNNAATVLQLPQGTTLPKGFYAEGSRGG   180
KY417145.1       SLLYGANKEGIVWVATEGALNTPKDHIGTRNPNNNAATVLQLPQGTTLPKGFYAEGSRGG   180
AY572038.1       SLPYGANKEGIVWVATEGALNTPKDHIGTRNPNNNAATVLQLPQGTTLPKGFYAEGSRGG   180
KT444582.1       SLPYGANKEGIVWVATEGALNTPKDHIGTRNPNNNAATVLQLPQGTTLPKGFYAEGSRGG   180
KY417143.1       SLPYGANKEGIVWVATEGALNTPKDHIGTRNPNNNAATVLQLPQGTTLPKGFYAEGSRGG   180
KY417152.1       SLPYGANKEGIVWVATEGALNTPKDHIGTRNPNNNAATVLQLPQGTTLPKGFYAEGSRGG   180
KC881005.1       SLPYGANKEGIVWVATEGALNTPKDHIGTRNPNNNAATVLQLPQGTTLPKGFYAEGSRGG   180
KY417146.1       SLPYGANKEGIIWVATEGALNTPKDHIGTRNPNNNAATVLQLPQGTTLPKGFYAEGSRGG   180
KY417148.1       SLPYGANKEGIVWVATEGALNTPKDHIGTRNPNNNAATVLQLPQGTTLPKGFYAEGSRGG   180
KY417142.1       SLPYGANKEGIVWVATEGALNTPKDHIGTRNPNNNVATVLQLPQGTTLPKGFYAEGSRGG   180
KY417144.1       SLPYGANKEGIVWVATEGALNTPKDHIGTRNPNNNAATVLQLPQGTTLPKGFYAEGSRGG   180
AY572034.1       SLPYGANKEGIVWVATEGALNTPKDHIGTRNPNNNAATVLQLPQGTTLPKGFYAEGSRGG   180
KY417147.1       SLPYGANKEGIVWVATEGALNTPKDHIGTRNPNNNAATVLQLPQGTTLPKGFYAEGSRGG   180
AP006560.1       SLPYGANKEGIVWVATEGALNTPKDHIGTRNPNNNAATVLQLPQGTTLPKGFYAEGSRGG   180
AP006557.1       SLPYGANKEGIVWVATEGALNTPKDHIGTRNPNNNAATVLQLPQGTTLPKGFYAEGSRGG   180
AY485277.1       SLPYGANKEGIVWVATEGALNTPKDHIGTRNPNNNAATVLQLPQGTTLPKGFYAEGSRGG   180
AY278488.2       SLPYGANKEGIVWVATEGALNTPKDHIGTRNPNNNAATVLQLPQGTTLPKGFYAEGSRGG   180
KC881006.1       SLPYGANKEGIVWVATEGALNTPKDHIGTRNPNNNAATVLQLPQGTTLPKGFYAEGSRGG   180
AY274119         SLPYGANKEGIVWVATEGALNTPKDHIGTRNPNNNAATVLQLPQGTTLPKGFYAEGSRGG   180
KF367457.1       SLPYGANKEGIVWVATEGALNTPKDHIGTRNPNNNAATVLQLPQGTTLPKGFYAEGSRGG   180
MT040336.1       GLPYGANKEGIIWVATEGALNTPKDHIGTRNPNNNAAIVLQLPQGTALPKGFYAEGSRGG   177
MT040333.1       GLPYGANKEGIIWVATEGALNTPKDHIGTRNPNNNAAIVLQLPQGTALPKGFYAEGSRGG   177
MT040335.1       GLPYGANKEGIIWVATEGALNTPKDHIGTRNPNNNAAIVLQLPQGTALPKGFYAEGSRGG   177
MW532698.1       GLPYGANKEGIIWVATEGALNTPKDHIGTRNPNNNAAIVLQLPQGTALPKGFYAEGSRGG   177
MT040334.1       GLPYGANKEGIIWVATEGALNTPKDHIGTRNPNNNAAIVLQLPQGTALPKGFYAEGSRGG   177
MG772933.1       GLPYGANKEGIIWVATEGALNTPKDHIGTRNPANNAAIVLQLPQGTTLPKGFYAEGSRGG   179
MG772934.1       GLPYGANKEGIIWVATEGALNTPKDHIGTRNPANNAAIVLQLPQGTTLPKGFYAEGSRGG   179
MN996532.2       GLPYGANKDGIIWVATEGALNTPKDHIGTRNPANNAAIVLQLPQGTTLPKGFYAEGSRGG   179
MN996529.1       GLPYGANKDGIIWVATEGALNTPKDHIGTRNPANNAAIVLQLPQGTTLPKGFYAEGSRGG   179
MT093571.1       GLPYGANKDGIIWVATEGALNTPKDHIGTRNPANNAAIVLQLPQGTTLPKGFYAEGSRGG   179
MT072688.1       GLPYGANKDGIIWVATEGALNTPKDHIGTRNPANNAAIVLQLPQGTTLPKGFYAEGSRGG   179
MN994467.1       GLPYGANKDGIIWVATEGALNTPKDHIGTRNPANNAAIVLQLPQGTTLPKGFYAEGSRGG   179
NC_045512.2      GLPYGANKDGIIWVATEGALNTPKDHIGTRNPANNAAIVLQLPQGTTLPKGFYAEGSRGG   179
MN988713.1       GLPYGANKDGIIWVATEGALNTPKDHIGTRNPANNAAIVLQLPQGTTLPKGFYAEGSRGG   179
                 .* *****:**:******************** **.* *:******:*************

                             CoVAX_MNS#16                 CoVAX_MNS#13
```

```
NC_014470.1    SQASSRSNSRSRGNSRNSTPSSSRGSSPARMAAG-GDTALALLLLDRLNQLESKVSGKTP     237
KP886809.1     SQASSRSSSRSRGNSRNSTPGSSRGTSPARIASGGGETALALLLLDRLNQLESKVSGKGQ     239
DQ022305       SQSSSRSSSRSRGNSRNSTPGSSRGSSPARLASGGGETALALLLLDRLNQLESKVSGKGQ     239
KF569996.1     SQASSRSSSRSRGNSRNSTPGSSRGNSPARMASGGGETALALLLLDRLNQLESKVSGKGQ     240
GQ153542.1     SQASSRSSSRSRGNSRNSTPGSSRGNSPARLASGGGETALALLLLDRLNQLESKVSGKGQ     239
FJ588686.1     SQASSRSSSRSRGNSRNSTPGSSRGNSPARMASGGGETALALLLLDRLNQLESKVSGKGQ     240
KY417151.1     SQASSRSSSRSRGNSRNSTPGSSRGNSPARMASGGGETALALLLLDRLNQLESKVSGKGQ     240
KY417145.1     SQASSRSSSRSRGNSRNSTPGSSRGNSPARMASGGGETALALLLLDRLNQLESKVSGKGQ     240
AY572038.1     SQASSRSSSRSRGNSRNSTPGSSRGNSPARMASGGGETALALLLLDRLNQLESKVSGKGQ     240
KT444582.1     SQASSRSSSRSRGNSRNSTPGSSRGNSPARMASGGGETALALLLLDRLNQLESKVSGKGQ     240
KY417143.1     SQASSRSSSRSRGDSRNSTPGSSRGNSPARMASGGGETALALLLLDRLNQLESKVSGKGQ     240
KY417152.1     SQASSRSSSRSRGNSRNSTPGSSRGNSPARMASGGGETALALLLLDRLNQLESKVSGKGQ     240
KC881005.1     SQASSRSSSRSRGNSRNSTPGSSRGNSPARMASGGGETALALLLLDRLNQLESKVSGKGQ     240
KY417146.1     SQASSRSSSRSRGNSRNSTPGSSRGNSPARMASGGGETALALLLLDRLNQLESKVSGKGQ     240
KY417148.1     SQASSRSSSRSRGNSRNSTPGSSRGNSPARMASGGGETALALLLLDRLNQLESKVSGKGQ     240
KY417142.1     SQASSRSSSRSRGNSRNSTPGSSRGNSPARMASGGGETALALLLLDRLNQLESKVSGKGQ     240
KY417144.1     SQASSRSSSRSRGNSRNSTPGSSRGNSPARMASGGGETALALLLLDRLNQLESKVSGKGQ     240
AY572034.1     SQASSRSSSRSRGNSRNSTPGSSRGNSPARMASGGGETALALLLLDRLNQLESKVSGKGQ     240
KY417147.1     SQASSRSSSRSRGNSRNSTPGSSRGNSPARMASGGGETALALLLLDRLNQLESKVSGKGQ     240
AP006560.1     SQASSRSSSRSRGNSRNSTPGSSRGNSPARMASGGGETALALLLLDRLNQLESKVSGKGQ     240
AP006557.1     SQASSRSSSRSRGNSRNSTPGSSRGNSPARMASGGGETALALLLLDRLNQLESKVSGKGQ     240
AY485277.1     SQASSRSSSRSRGNSRNSTPGSSRGNSPARMASGGGETALALLLLDRLNQLESKVSGKGQ     240
AY278488.2     SQASSRSSSRSRGNSRNSTPGSSRGNSPARMASGGGETALALLLLDRLNQLESKVSGKGQ     240
KC881006.1     SQASSRSSSRSRGNSRNSTPGSSRGNSPARMASGGGETALALLLLDRLNQLESKVSGKGQ     240
AY274119       SQASSRSSSRSRGNSRNSTPGSSRGNSPARMASGGGETALALLLLDRLNQLESKVSGKGQ     240
KF367457.1     SQASSRSSSRSRGNSRNSTPGSSRGNSPARMASGGGETALALLLLDRLNQLESKVSGKGQ     240
MT040336.1     SQASSRSSSRSRNSSRNSTPGSSRGTSPARIAGNGGDAALALLLLDRLNALESKMSGKGS     237
MT040333.1     SQASSRSSSRSRNSSRNSTPGSSRGTSPARIAGNGGDAALALLLLDRLNALESKMSGKGS     237
MT040335.1     SQASSRSSSRSRNSSRNSTPGSSRGTSPARIAGNGGDAALALLLLDRLNALESKMSGKGS     237
MW532698.1     SQASSRSSSRSRNSSRNSTPGSSRGTSPARIAGNGGDAALALLLLDRLNALESKMSGKGS     237
MT040334.1     SQASSRSSSRSRNSSRNSTPGSSRGTSPARIAGNGGDAALALLLLDRLNALESKMSGKGS     237
MG772933.1     SQASSRSSSRSRNSSRNSTPGSSRGTSPARMAGNGGDTALALLLLDRLNQLENKVSGKGQ     239
MG772934.1     SQASSRSSSRSRNSSRNSTPGSSRGTSPARMAGNGGDTALALLLLDRLNQLENKISGKGQ     239
MN996532.2     SQASSRSSSRSRNSSRNSTPGSSRGTSPARMAGNGSDAALALLLLDRLNQLESKMSGKGQ     239
MN996529.1     SQASSRSSSRSRNSSRNSTPGSSRGTSPARMAGNGGDAALALLLLDRLNQLESKMSGKGQ     239
MT093571.1     SQASSRSSSRSRNSSRNSTPGSSRGTSPARMAGNGGDAALALLLLDRLNQLESKMSGKGQ     239
MT072688.1     SQASSRSSSRSRNSSRNSTPGSSRGTSPARMAGNGGDAALALLLLDRLNQLESKMSGKGQ     239
MN994467.1     SQASSRSSSRSRNSSRNSTPGSSRGTSPARMAGNGGDAALALLLLDRLNQLESKMSGKGQ     239
NC_045512.2    SQASSRSSSRSRNSSRNSTPGSSRGTSPARMAGNGGDAALALLLLDRLNQLESKMSGKGQ     239
MN988713.1     SQASSRSSSRSRNSXRNSTPGSSRGTSPARMAGNGGDAALALLLLDRLNQLESKMSGKGQ     239
               **:****.****.. *****.****.****:*.. .::*********** **.*:***
```

CoVAX_MNS#13 CoVAX_MNS#12

```
NC_014470.1    Q-QSQVVTKKTAAEASKKPRQKRTATKAYNVTQAFGRRGPEPTQGNFGDQELIRLGTDYK    296
KP886809.1     QQQGQTVTKKSAAEASKKPRQKRTATKQYNVTQAFGRRGPDQTQGNFGDQDLIRQGTDYK    299
DQ022305       QQPGQTVTKKSAAEASKKPRQKRTATKQYNVTQAFGRRGPEQTQGNFGDQELIRQGIDYK    299
KF569996.1     QQQGQTVTKKSAAEASKKPRQKRTATKAYNVTQAFGRRGPEQTQGNFGDQELIRQGTDYK    300
GQ153542.1     QQQGQTVTKKSAAEASKKPRQKRTATKQYNVTQAFGRRGPEQTQGNFGDQDLIRQGTDYK    299
FJ588686.1     QQQGQTVTKKSAAEASKKPRQKRTATKQYNVTQAFGRRGPEQTQGNFGDQDLIRQGTDYK    300
KY417151.1     QQQGQTVTKKSAAEASKKPRQKRTATKQYNVTQAFGRRGPEQTQGNFGDQDLIRQGTDYK    300
KY417145.1     QQQGQTVTKKSAAEASKKPRQKRTATKQYNVTQAFGRRGPEQTQGNFGDQDLIRQGTDYK    300
AY572038.1     QQQGQTVTKKSAAEASKKPRQKRTATKQYNVTQAFGRRGPEQTQGNFGDQDLIRQGIDYK    300
KT444582.1     QQQGQTVTKKSAAEASKKPRQKRTATKQYNVTQAFGRRGPEQTQGNFGDQDLIRQGTDYK    300
KY417143.1     QQQGQTVTKKSAAEASKKPRQKRTATKQYNVTQAFGRRGPEQTQGNFGDQDLIRQGTDYK    300
KY417152.1     QQQGQTVTKKSAAEASKKPRQKRTATKQYNVTQAFGRRGPEQTQGNFGDQDLIRQGTDYK    300
KC881005.1     QQQGQTVTKKSAAEASKKPRQKRTATKQYNVTQAFGRRGPEQTQGNFGDQDLIRQGTDYK    300
KY417146.1     QQQGQTVTKKSAAEASKKPRQKRTATKQYNVTQAFGRRGPEQTQGNFGDQDLIRQGTDYK    300
KY417148.1     QQQGQTVTKKSAAEASKKPRQKRTATKQYNVTQAFGRRGPEQTQGNFGDQDLIRQGTDYK    300
KY417142.1     QQQGQTVTKKSAAEASKKPRQKRTATKQYNVTQAFGRRGPEQTQGNFGDQDLIRQGTDYK    300
KY417144.1     QQQGQTVTKKSAAEASKKPRQKRTATKQYSVTQAFGRRGPEQTQGNFGDQDLIRQGTDYK    300
AY572034.1     QQQGQTVTKKSAAEASKKPRQKRTATKQYNVTQAFGRRGPEQTQGNFGDQDLIRQGTDYK    300
KY417147.1     QQQGQTVTKKSAAEASKKPRQKRTATKAYNVTQAFGRRGPEQTQGNFGDQDLIRQGTDYK    300
AP006560.1     QQQGQTVTKKSAAEASKKPRQKRTATKQYNVTQAFGRRGPEQTQGNFGDQDLIRQGTDYK    300
AP006557.1     QQQGQTVTKKSAAEASKKPRQKRTATKQYNVTQAFGRRGPEQTQGNFGDQDLIRQGTDYK    300
AY485277.1     QQQGQTVTKKSAAEASKKPRQKRTATKQYNVTQAFGRRGPEQTQGNFGDQDLIRQGTDYK    300
AY278488.2     QQQGQTVTKKSAAEASKKPRQKRTATKQYNVTQAFGRRGPEQTQGNFGDQDLIRQGTDYK    300
KC881006.1     QQQGQTVTKKSAAEASKKPRQKRTATKQYNVTQAFGRRGPEQTQGNFGDQDLIRQGTDYK    300
AY274119       QQQGQTVTKKSAAEASKKPRQKRTATKQYNVTQAFGRRGPEQTQGNFGDQDLIRQGTDYK    300
KF367457.1     QQQGQTVTKKSAAEASRKPRQKRTATKQYNVTQAFGRRGPEQTQGNFGDQDLIRQGTDYK    300
MT040336.1     QQQSQTVTKKSAAEASKKPRQKRTATKQYNVTQAFGRRGPEKTQGNFGDKELIRQGTEYK    297
MT040333.1     QQQSQTVTKKSAAEASKKPRQKRTATKQYNVTQAFGRRGPEQTQGNFGDQELIRQGTEYK    297
MT040335.1     QKQSQTVTKKSAAEASKKPRQKRTATKQYNVTQAFGRRGPEQTQGNFGDQELIRQGTEYK    297
MW532698.1     QQQSQTVTKKSAAEASKKPRQKRTATKQYNVTQAFGRRGPEQTQGNFGDQELIRQGTEYK    297
MT040334.1     QQQSQTVTKKSAAEASKKPRQKRTATKQYNVTQAFGRRGPEQTQGNFGDQELIRQGTEYK    297
MG772933.1     QQQGQTVTKKSAAEASKKPRQKRTATKQYNVTQAFGRRGPEQTQGNFGDQELIRQGTDYK    299
MG772934.1     QQQGQTVTKKSAAEASKKPRQKRTATKQYNVTQAFGRRGPEQTQGNFGDQELIRQGTDYK    299
MN996532.2     QQQSQTVTKKSAAEASKKPRQKRTATKQYNVTQAFGRRGPEQTQGNFGDQELIRQGTDYK    299
MN996529.1     QQQGQTVTKKSAAEASKKPRQKRTATKAYNVTQAFGRRGPEQTQGNFGDQELIRQGTDYK    299
MT093571.1     QQQGQTVTKKSAAEASKKPRQKRTATKAYNVTQAFGRRGPEQTQGNFGDQELIRQGTDYK    299
MT072688.1     QQQGQTVTKKSAAEASKKPRQKRTATKAYNVTQAFGRRGPEQTQGNFGDQELIRQGTDYK    299
MN994467.1     QQQGQTVTKKSAAEASKKPRQKRTATKAYNVTQAFGRRGPEQTQGNFGDQELIRQGTDYK    299
NC_045512.2    QQQGQTVTKKSAAEASKKPRQKRTATKAYNVTQAFGRRGPEQTQGNFGDQELIRQGTDYK    299
MN988713.1     QQQGQTVTKKSAAEASKKPRQKRTATKAYNVTQAFGRRGPEQTQGNFGDQELIRQGTDYK    299
               *  .*.****:*****:********** *.**********: *******::*** * :**
```

CoVAX_MNS#12 CoVAX_MNS#15

```
NC_014470.1      NWPQIAQFAPSASAFFGMSRIGMEVTPTGTWLTYNGAIKLDDKDPNFKDQVILLNKHIDA     356
KP886809.1       HWPQIAQFAPSASAFFGMSRIGMEVTPSGTWLTYHGAIKLDDKDPQFKDNVILLNKHIDA     359
DQ022305         HWPQIAQFAPSASAFFGMSRIGMEVTPSGTWLTYHGAIKLDDKDPQFKDNVILLNKHIDA     359
KF569996.1       HWPQIAQFAPSASAFFGMSRIGMEVTPSGTWLTYHGAIKLDDKDPQFKDNVIMLNKHIDA     360
GQ153542.1       HWPQIAQFAPSASAFFGMSRIGMEVTPSGTWLTYHGAIKLDDKDPQFKDNVILLNKHIDA     359
FJ588686.1       HWPQIAQFAPSASAFFGMSRIGMEVTPSGTWLTYHGAIKLDDKDPQFKDNVILLNKHIDA     360
KY417151.1       HWPQIAQFAPSASAFFGMSRIGMEVTPSGTWLTYHGAIKLDDKDPQFKDNVILLNKHIDA     360
KY417145.1       HWPQIAQFAPSASAFFGMSRIGMEVTPSGTWLTYHGAIKLDDKDPQFKDNVILLNKHIDA     360
AY572038.1       QWPQIAQFAPSASAFFGMSRIGMEVTPSGTWLTYHGAIKLDDKDPQFKDNVILLNKHIDA     360
KT444582.1       HWPQIAQFAPSASAFFGMSRIGMEVTPSGTWLTYHGAIKLDDKDPQFKDNVILLNKHIDA     360
KY417143.1       HWPQIAQFAPSASAFFGMSRIGMEVTPSGTWLTYHGAIKLDDKDPQFKDNVILLNKHIDA     360
KY417152.1       HWPQIAQFAPSASAFFGMSRIGMEVTPSGTWLTYHGAIKLDDKDPQFKDSVILLNKHIDA     360
KC881005.1       HWPQIAQFAPSASAFFGMSRIGMEVTPSGTWLTYHGAIKLDDKDPQFKDNVILLNKHIDA     360
KY417146.1       HWPQIAQFAPSASAFFGMSRIGMEVTPSGTWLTYHGAIKLDDKDPQFKDNVILLNKHIDA     360
KY417148.1       HWPQIAQFAPSASAFFGMSRIGMEVTPSGTWLTYHGAIKLDDKDPQFKDNVILLNKHIDA     360
KY417142.1       HWPQIAQFAPSASAFFGMSRIGMEVTPSGTWLTYHGAIKLDDKDPQFKDNVILLNKHIDA     360
KY417144.1       HWPQIAQFAPSASAFFGMSRIGMEVTLSGTWLTYHGAIKLDDKDPQFKDNVILLNKHIDA     360
AY572034.1       QWPQIAQFAPSASAFFGMSRIGMEVTPSGTWLTYHGAIKLDDKDPQFKDNVILLNKHIDA     360
KY417147.1       HWPQIAQFAPSASAFFGMSRIGMEVTPSGTWLTYHGAIKLDDKDPQFKDNVILLNKHIDA     360
AP006560.1       HWPQIAQFAPSASAFFGMSRIGMEVTPSGTWLTYHGAIKLDDKDPQFKDNVILLNKHIDA     360
AP006557.1       HWPQIAQFAPSASAFFGMSRIGMEVTPSGTWLTYHGAIKLDDKDPQFKDNVILLNKHIDA     360
AY485277.1       HWPQIAQFAPSASAFFGMSRIGMEVTPSGTWLTYHGAIKLDDKDPQFKDNVILLNKHIDA     360
AY278488.2       HWPQIAQFAPSASAFFGMSRIGMEVTPSGTWLTYHGAIKLDDKDPQFKDNVILLNKHIDA     360
KC881006.1       HWPQIAQFAPSASAFFGMSRIGMEVTPSGTWLTYHGAIKLDDKDPQFKDNVILLNKHIDA     360
AY274119         HWPQIAQFAPSASAFFGMSRIGMEVTPSGTWLTYHGAIKLDDKDPQFKDNVILLNKHIDA     360
KF367457.1       HWPQIAQFAPSASAFFGMSRIGMEVTPSGTWLTYHGAIKLDDKDPQFKDNVILLNKHIDA     360
MT040336.1       HWPQIAQFAPSASAFFGMSRIGMEVTPSGTWLTYTGAIKLDDKDPSFKDNVILLNKHIDA     357
MT040333.1       HWPQIAQFAPSASAFFGMSRIGMEVTPSGTWLTYTGAIKLDDKDPSFKDNVILLNKHIDA     357
MT040335.1       HWPQIAQFAPSASAFFGMSRIGMEVTPSGTWLTYTGAIKLDDKDPSFKDNVILLNKHIDA     357
MW532698.1       HWPQIAQFAPSASAFFGMSRIGMEVTPSGTWLTYTGAIKLDDKDPSFKDNVILLNKHIDA     357
MT040334.1       HWPQIAQFAPSASAFFGMSRIGMEVTPSGTWLTYTGAIKLDDKDPSFKDNVILLNKHIDA     357
MG772933.1       HWPQIAQFAPSASAFFGMSRIGMEVTPSGTWLTYHGAIKLDDKDPQFKDNVILLNKHIDA     359
MG772934.1       HWPQIAQFAPSASAFFGMSRIGMEVTPSGTWLTYHGAIKLDDKDPQFKDNVILLNKHIDA     359
MN996532.2       HWPQIAQFAPSASAFFGMSRIGMEVTPSGTWLTYTGAIKLDDKDPNFKDQVILLNKHIDA     359
MN996529.1       HWPQIAQFAPSASAFFGMSRIGMEVTPSGTWLTYTGAIKLDDKDPNFKDQVILLNKHIDA     359
MT093571.1       HWPQIAQFAPSASAFFGMSRIGMEVTPSGTWLTYTGAIKLDDKDPNFKDQVILLNKHIDA     359
MT072688.1       HWPQIAQFAPSASAFFGMSRIGMEVTPSGTWLTYTGAIKLDDKDPNFKDQVILLNKHIDA     359
MN994467.1       HWPQIAQFAPSASAFFGMSRIGMEVTPSGTWLTYTGAIKLDDKDPNFKDQVILLNKHIDA     359
NC_045512.2      HWPQIAQFAPSASAFFGMSRIGMEVTPSGTWLTYTGAIKLDDKDPNFKDQVILLNKHIDA     359
MN988713.1       HWPQIAQFAPSASAFFGMSRIGMEVTPSGTWLTYTGAIKLDDKDPNFKDQVILLNKHIDA     359
                 :************************* :****** **********.***.**:*******
```

CoVAX_MNS#11 CoVAX_MNS#7

```
NC_014470.1       YKTFPPTEPKKDKKKKADEVQSLPQRQKKQATVTLLPAADLDDFSKQLQNSMNASPDST-      415
KP886809.1        YKTFPPTEPKKDKKKKTDEAQPLPQRQKKQPTVTLLPAADMDDFSRQLQNSMSEASADST      419
DQ022305          YKTFPPTEPKKDKKKKTDEAQPLPQRQKKQPTVTLLPAADMDDFSRQLQHSMSGASADST      419
KF569996.1        YKTFPPTEPKKDKKKKTDEAQPLPQRQKKQPTVTLLPAADMDDFSRQLQNSMSGASADST      420
GQ153542.1        YKTFPPTEPKKDKKKKTDEAQPLPQRQKKQPTVTLLPAADMDDFSRQLQKSMSGASADST      419
FJ588686.1        YKTFPPTEPKKDKKKKTDEAQPLPQRQKKQPTVTLLPAADMDDFSRQLQNSMSGASADST      420
KY417151.1        YKTFPPTEPKKDKKKKTDEAQPLPQRQKKQPTVTLLPAADMDDFSRQLQSSMSGASADST      420
KY417145.1        YKTFPPTEPKKDKKKKTDEAQPLPQRQKKQPTVTLLPAADMDDFSRQLQSSMSGASADST      420
AY572038.1        YKTFPPTEPKKDKKKKTDEAQPLPQRQKKQPTVTLLPAADMDDFSRQLQNSMSGASADST      420
KT444582.1        YKTFPPTEPKKDKKKKTDEAQPLPQRQKKQPTVTLLPAADMDDFSRQLQNSMSGASADST      420
KY417143.1        YKTFPPTEPKKDKKKKTDEAQPLPQRQKKQPTVTLLPAADMDDFSRQLQNSMSGASADST      420
KY417152.1        YKTFPPTEPKKDKKKKTDEAQPLPQRQKKQPTVTLLPAADMDDFSRQLQNSMSGASADST      420
KC881005.1        YKTFPPTEPKKDKKKKTDEAQPLPQRQKKQPTVTLLPAADMDDFSRQLQNSMSGASADST      420
KY417146.1        YKTFPPTEPKKDKKKKTDEAQPLPQRQKKQPTVTLLPAADMDDFSRQLQNSMSGASADST      420
KY417148.1        YKTFPPTEPKKDKKKKTDEAQPLPQRQKKQPTVTLLPAADMDDFSRQLQNSMSGASADST      420
KY417142.1        YKTFPPTEPKKDKKKKTDEAQPLPQRQKKQPIVTLLPAADMDDFSRQLQNSMSGASADST      420
KY417144.1        YKTFPPTEPKKDKKKKTDEAQPLPQRQKKQPTVTLLPAADMDDFSRQLQNSMSGASADST      420
AY572034.1        YKTFPPTEPKKDKKKKTDEAQPLLQRQKKQPTVTLLPAADMDDFSRQLQNSMSGASADST      420
KY417147.1        YKTFPPTEPKKDKKKKTDEAQPLPQRQKKQPTVTLLPAADMDDFSRQLQNSMSGASADST      420
AP006560.1        YKTFPPTEPKKDKKKKTDEAQPLPQRQKKQPTVTLLPAADMDDFSRQLQNSMSGASADST      420
AP006557.1        YKTFPPTEPKKDKKKKTDEAQPLPQRQKKQPTVTLLPAADMDDFSRQLQNSMSGASADST      420
AY485277.1        YKTFPPTEPKKDKKKKTDEAQPLPQRQKKQPTVTLLPAADMDDFSRQLQNSMSGASADST      420
AY278488.2        YKTFPPTEPKKDKKKKTDEAQPLPQRQKKQPTVTLLPAADMDDFSRQLQNSMSGASADST      420
KC881006.1        YKTFPPTEPKKDKKKKTDEAQPLPQRQKKQPTVTLLPAADMDDFSRQLQNSMSGASADST      420
AY274119          YKTFPPTEPKKDKKKKTDEAQPLPQRQKKQPTVTLLPAADMDDFSRQLQNSMSGASADST      420
KF367457.1        YKTFPPTEPKKDKKKKTDEAQPLPQRQKKQPTVTLLPAADMDDFSRQLQNSMSGASADST      420
MT040336.1        YKTFPPTEPKKDKKKKTDESQPLPQRQKKQQTVTLLPAADLDDFSKQLQQSMSSADSTQA      417
MT040333.1        YKTFPPTEPKKDKKKKTDESQPLPQRQKKQQTVTRLPAADLDDFSKQLQQSMSSADSTQA      417
MT040335.1        YKTFPPTEPKKDKKKKTDESQPLPQRQKKQQTVTLLPAADLDDFSKQLQQSMSSADSTQA      417
MW532698.1        YKTFPPTEPKKDKKKKTDESQPLPQRQKKQQTVTLLPAADLDDFSKQLQQSMSSADSTQV      417
MT040334.1        YKTFPPTEPKKDKKKKTDESQPLPQRQKKQQTVTLLPAADLDDFSKQLQQSMSSADSTQA      417
MG772933.1        YKTFPPTEPKKDKKKKADELQALPQRQKKQQTVTLLPAADLDEFSKQLQQSMSGTDSTQA      419
MG772934.1        YKTFPPTEPKKDKKKKADELQALPQRQKKQQTVTLLPAADLDEFSKQLQQSMSGTDSTQA      419
MN996532.2        YKTFPPTEPKKDKKKKADETQALPQRQKKQQTVTLLPAADLDDFSKQLQQSMSSADSTQA      419
MN996529.1        YKTFPPTEPKKDKKKKADETQALPQRQKKQQTVTLLPAADLDDFSKQLQQSMSSADSTQA      419
MT093571.1        YKTFPPTEPKKDKKKKADETQALPQRQKKQQTVTLLPAADLDDFSKQLQQSMSSADSTQA      419
MT072688.1        YKTFPPTEPKKDKKKKADETQALPQRQKKQQTVTLLPAADLDDFSKQLQQSMSSADSTQA      419
MN994467.1        YKTFPPTEPKKDKKKKADETQALPQRQKKQQTVTLLPAADLDDFSKQLQQSMSSADSTQA      419
NC_045512.2       YKTFPPTEPKKDKKKKADETQALPQRQKKQQTVTLLPAADLDDFSKQLQQSMSSADSTQA      419
MN988713.1        YKTFPPTEPKKDKKKKADETQALPQRQKKQQTVTLLPAADLDDFSKQLQQSMSSADSTQA      419
                  ****************:** * * ******  ** *****:*:**:*** **. :
```

CoVAX_MNS#7

NC_014470.1	QA	417
KP886809.1	QA	421
DQ022305	QA	421
KF569996.1	QA	422
GQ153542.1	QA	421
FJ588686.1	QA	422
KY417151.1	QA	422
KY417145.1	QA	422
AY572038.1	QA	422
KT444582.1	QA	422
KY417143.1	QA	422
KY417152.1	QA	422
KC881005.1	QA	422
KY417146.1	QA	422
KY417148.1	QA	422
KY417142.1	QA	422
KY417144.1	QA	422
AY572034.1	QA	422
KY417147.1	QA	422
AP006560.1	QA	422
AP006557.1	QA	422
AY485277.1	QA	422
AY278488.2	QA	422
KC881006.1	QA	422
AY274119	QA	422
KF367457.1	QA	422
MT040336.1	--	417
MT040333.1	--	417
MT040335.1	--	417
MW532698.1	--	417
MT040334.1	--	417
MG772933.1	--	419
MG772934.1	--	419
MN996532.2	--	419
MN996529.1	--	419
MT093571.1	--	419
MT072688.1	--	419
MN994467.1	--	419
**NC_045512.2**	**--**	**419**
MN988713.1	--	419

Supplemental Figure 3. S clustal aligment. Clustal O (version 1.2.4) multiple sequence alignment using the sarbecoviruses listed in Supplemental Table 2. The Wuhan-Hu-1 (NC_045512.2) sequence is indicated in bold, and selected antigens (Supplemental Table 3) are boxed and shaded.

```
NC_014470.1      ---MKFLAFLCLLGFANAQDGKCGTLSNKSPSKLTQTPSSRRGFYYFDDIFRSSIRVLTT     57
KF569996.1       MFLTCFILSFSLFCVSGDSIDTCETFDDVSPPQQNLVSSSKRGVYYPDDIYRSDVHHLVQ     60
KY417144.1       ---MKLLVLVFATLVSSYTIEKCLDFDDRTPPANTQFLSSHRGVYYPDDIFRSNVLHLVQ     57
KC881005.1       ---MKLLVLVFATLVSSYTIEKCLDFDDRTPPANTQFLSSHRGVYYPDDIFRSNVLHLVQ     57
KC881006.1       ---MKLLVLVFATLVSSYTIEKCLDFDDRTPPANTQFLSSHRGVYYPDDIFRSNVLHLVQ     57
KF367457.1       ---MKLLVLVFATLVSSYTIEKCLDFDDRTPPANTQFLSSHRGVYYPDDIFRSNVLHLVQ     57
KY417152.1       ---MKLLVLVFATLVSSYTIEKCLDFDDRTPPANTQFLSSHRGVYYPDDIFRSNVLHLVQ     57
KY417151.1       ---MKLLVLVFATLVSSYTIEKCLDFDDRTPPANTQFLSSHRGVYYPDDIFRSNVLHLVQ     57
KY417146.1       ----MFIFLFFLTLTSGSDLESCTTFDDVQAPNYPQHSSSRRGVYYPDEIFRSDTLYLTQ     56
KT444582.1       ----MFIFLFFLTLTSGSDLESCTTFDDVQAPNYPQHSSSRRGVYYPDEIFRSDTLYLTQ     56
AY278488.2       ----MFIFLLFLTLTSGSDLDRCTTFDDVQAPNYTQHTSSMRGVYYPDEIFRSDTLYLTQ     56
AY274119         ----MFIFLLFLTLTSGSDLDRCTTFDDVQAPNYTQHTSSMRGVYYPDEIFRSDTLYLTQ     56
AP006560.1       ----MFIFLLFLTLTSGSDLDRCTTFDDVQAPNYTQHTSSMRGVYYPDEIFRSDTLYLTQ     56
AP006557.1       ----MFIFLLFLTLTSGSDLDRCTTFDDVQAPNYTQHTSSMRGVYYPDEIFRSDTLYLTQ     56
AY485277.1       ----MFIFLLFLTLTSGSDLDRCTTFDDVQAPNYTQHTSSMRGVYYPDEIFRSDTLYLTQ     56
AY572038.1       ----MFIFLLFLTLTSGSDLDRCTTFDDVQAPNYTQHTSSMRGVYYPDEIFRSDTLYLTQ     56
AY572034.1       ----MFIFLLFLTLTSGSDLDRCTTFDDVQAPNYTQHTSSMRGVYYPDEIFRSDTLYLTQ     56
MT040334.1       -------MFVFL-FVLPLVSSQCVNLTTRTGIQPGYTNSSTRGVYYPDKVFRSSILHLTQ     52
MW532698.1       -------MFVFL-FVLPLVSSQCVNLTTRTGIPPGYTNSSTRGVYYPDKVFRSSILHLTQ     52
MT040333.1       -------MFVFL-FVLPLVSSQCVNLTTRTGIPPGYTNSSTRGVYYPDKVFRSSILHLTQ     52
MT040336.1       -------MFVFL-FVLPLVSSQCVNLTTRTGIPPGYTNSSTRGVYYPDKVFRSSILHLTQ     52
MT040335.1       -------MFVFL-FVLPLVSSQCVNLTTRTGIPPGYTNSSTRGVYYPDKVFRSSILHLTQ     52
MN996532.2       -------MFVFL-VLLPLVSSQCVNLTTRTQLPPAYTNSSTRGVYYPDKVFRSSVLHLTQ     52
MN996529.1       -------MFVFL-VLLPLVSSQCVNLTTRTQLPPAYTNSFTRGVYYPDKVFRSSVLHSTQ     52
MN988713.1       -------MFVFL-VLLPLVSSQCVNLTTRTQLPPAYTNSFTRGVYYPDKVFRSSVLHSTQ     52
MT072688.1       -------MFVFL-VLLPLVSSQCVNLTTRTQLPPAYTNSFTRGVYYPDKVFRSSVLHSTQ     52
MN994467.1       -------MFVFL-VLLPLVSSQCVNLTTRTQLPPAYTNSFTRGVYYPDKVFRSSVLHSTQ     52
NC_045512.2      -------MFVFL-VLLPLVSSQCVNLTTRTQLPPAYTNSFTRGVYYPDKVFRSSVLHSTQ     52
MT093571.1       -------MFVFL-VLLPLVSSQCVNLTTRTQLPPAYTNSFTRGVYYPDKVFRSSVLHSTQ     52
MG772933.1       ---MLF--FLFL-Q-FALVNSQCVNLTGRTPLNPNYTNSSQRGVYYPDTIYRSDTLVLSQ     53
MG772934.1       ---MLF--FLFL-Q-FALVNSQC-DLTGRTPLNPNYTNSSQRGVYYPDTIYRSDTLVLSQ     52
KY417145.1       ----MFFLIGYT-AFLIGYTAATTCVTGPTTENKLNISSGSRGVYYPDDIFRSDVSVLVT     55
KP886809.1       ---MKILIFAFL-VTLVKAQEGCGVINLRTQPKLSQVSSSRRGVYYNDDIFRSDVLHLTQ     56
KY417143.1       ---MKVLIVLLS-LGLVTAQDGCGHISTKPQPLMDKFSSSRRGVYYNDDIFRSDVLHLTQ     56
FJ588686.1       ---MKVLIVLLC-LGLVTAQDGCGHISTKPQPLMDKFSSSRRGVYYNDDIFRSDVLHLTQ     56
KY417148.1       ---MKILIFAFL-VTLVKAQEGCGIISRKPQPKMAQVSSSRRGVYYNDDIFRSDVLHLTQ     56
KY417147.1       ---MKILIFAFL-VTLVKAQEGCGLISRKPQPKMAQVSSSRRGVYYNDDIFRSDVLHLTQ     56
KY417142.1       ---MKILIFAFL-VTLVKAQEGCGIISRKPQPKMAQVSSSRRGVYYNDDIFRSDVLHLTQ     56
GQ153542.1       ---MKILIFAFL-ASLAKAQEGCGIISRKPQPKMAQVSSSRRGVYYNDDIFRSDVLHLTQ     56
DQ022305         ---MKILIFAFL-ANLAKAQEGCGIISRKPQPKMAQVSSSRRGVYYNDDIFRSDVLHLTQ     56
                                          .            *  **.** * ::**.
```

```
NC_014470.1      GHFLPFNTNLTWYLTLKSNGKQ-RIYYDNPNINFGDGVYFGLTEKSNVFRGWIFGSTLDN      116
KF569996.1       DLFLPFNSNVVGLMSFN-------YRFDNPIIPFKDGVYFAATEKSNVVRGWVFGSTMNN      113
KY417144.1       DHFLPFDSNVTRFITFG-------LNFDNPIIPFRDGIYFAATEKSNVIRGWVFGSTMNN      110
KC881005.1       DHFLPFDSNVTRFITFG-------LNFDNPIIPFRDGIYFAATEKSNVIRGWVFGSTMNN      110
KC881006.1       DHFLPFDSNVTRFITFG-------LNFDNPIIPFKDGIYFAATEKSNVIRGWVFGSTMNN      110
KF367457.1       DHFLPFDSNVTRFITFG-------LNFDNPIIPFKDGIYFAATEKSNVIRGWVFGSTMNN      110
KY417152.1       DHFLPFDSNVTRFITFG-------LNFDNPIIPFRDGIYFAATEKSNVIRGWVFGSTMNN      110
KY417151.1       DHFLPFDSNVTRFITFG-------LNFDNPIIPFRDGIYFAATEKSNVIRGWVFGSTMNN      110
KY417146.1       DLFLPFYSNVTGFHTIN-------HRFDNPVIPFKDGVYFAATEKSNVVRGWVFGSTMNN      109
KT444582.1       DLFLPFYSNVTGFHTIN-------HRFDNPVIPFKDGVYFAATEKSNVVRGWVFGSTMNN      109
AY278488.2       DLFLPFYSNVTGFHTIN-------HTFDNPVIPFKDGIYFAATEKSNVVRGWVFGSTMNN      109
AY274119         DLFLPFYSNVTGFHTIN-------HTFGNPVIPFKDGIYFAATEKSNVVRGWVFGSTMNN      109
AP006560.1       DLFLPFYSNVTGFHTIN-------HTFGNPVIPFKDGIYFAATEKSNVVRGWVFGSTMNN      109
AP006557.1       DLFLPFYSNVTGFHTIN-------HTFGNPVIPFKDGIYFAATEKSNVVRGWVFGSTMNN      109
AY485277.1       DLFLPFYSNVTGFHTIN-------HTFGNPVIPFKDGIYFAATEKSNVVRGWVFGSTMNN      109
AY572038.1       DLFLPFYSNVTGFHTIN-------HTFDNPVIPFKDGIYFAATEKSNVVRGWVFGSTMNN      109
AY572034.1       DLFLPFYSNVTGFHTIN-------HTFDNPVIPFKDGIYFAATEKSNVVRGWVFGSTMNN      109
MT040334.1       DLFLPFFSNVTWFNTI--NYQGGFKKFDNPVLPFNDGVYFASTEKSNIIRGWIFGTTLDA      110
MW532698.1       DLFLPFFSNVTWFNTIHLNYQGGFKKFDNPVLPFNDGVYFASTEKSNIIRGWIFGTTLDA      112
MT040333.1       DLFLPFFSNVTWFNTI--NYQGGFKKFDNPVLPFNDGVYFASTEKSNIIRGWIFGTTLDA      110
MT040336.1       DLFLPFFSNVTWFNTI--TYQGGSKKFDNPVLPFNDGVYFASTEKSNIIRGWIFGTTLDA      110
MT040335.1       DLFLPFFSNVTWFNTI--NYQGGFKKFDNPVLPFNDGVYFASTEKSNIIRGWIFGTTLDA      110
MN996532.2       DLFLPFFSNVTWFHAIHVSGTNGIKRFDNPVLPFNDGVYFASTEKSNIIRGWIFGTTLDS      112
MN996529.1       DLFLPFFSNVTWFHAIHVSGTNGTKRFDNPVLPFNDGVYFASTEKSNIIRGWIFGTTLDS      112
MN988713.1       DLFLPFFSNVTWFHAIHVSGTNGTKRFDNPVLPFNDGVYFASTEKSNIIRGWIFGTTLDS      112
MT072688.1       DLFLPFFSNVTWFHAIHVSGTNGTKRFDNPVLPFNDGVYFASTEKSNIIRGWIFGTTLDS      112
MN994467.1       DLFLPFFSNVTWFHAIHVSGTNGTKRFDNPVLPFNDGVYFASTEKSNIIRGWIFGTTLDS      112
NC_045512.2      DLFLPFFSNVTWFHAIHVSGTNGTKRFDNPVLPFNDGVYFASTEKSNIIRGWIFGTTLDS      112
MT093571.1       DLFLPFFSNVTWFHAIHVSGTNGTKRFDNPVLPFNDGVYFASTEKSNIIRGWIFGTTLDS      112
MG772933.1       GYFLPFYSNVSWYYSLTTNN-AATKRTDNPILDFKDGIYFAATEHSNIIRGWIFGTTLDN      112
MG772934.1       GYFLPFYSNVSWYYSLTTNN-AATKRTDNPILDFKDGIYFAATEHSNIVRGWIFGTTLDN      111
KY417145.1       GPFLRFNTTLTWYNSWN-------QAYSSPILPFGHGVYFSTIDKSNVVRGWIFGTTLDN      108
KP886809.1       DYFLPFHSNLTQYFSLSIES-DKIVYFDNPILKFGDGIYFAATEKSNVIRGWVFGSTFDN      115
KY417143.1       DYFLPFDTNLTRYLSFNMDS-ATKVYFDNPTLPFGDGIYFAATEKSNVVRGWIFGSTMDN      115
FJ588686.1       DYFLPFDTNLTRYLSFNMDS-ATKVYFDNPTLPFGDGIYFAATEKSNVVRGWIFGSTMDN      115
KY417148.1       DYFLPFDSNLTQYFSLNVDS-DRYTYFDNPILDFGDGVYFAATEKSNVIRGWIFGSTFDN      115
KY417147.1       DYFLPFDSNLTQYFSLNVDS-DRYTYFDNPILDFGDGVYFAATEKSNVIRGWIFGSTFDN      115
KY417142.1       DYFLPFDSNLTQYFSLNVDS-DRYTYFDNPILDFGDGVYFAATEKSNVIRGWIFGSTFDN      115
GQ153542.1       DYFLPFDSNLTQYFSLNVDS-DRYTYFDNPILDFGDGVYFAATEKSNVVRGWIFGSSFDN      115
DQ022305         DYFLPFDSNLTQYFSLNVDS-DRYTYFDNPILDFGDGVYFAATEKSNVIRGWIFGSSFDN      115
                 . ** * :.:    :            ..* : * .*:**.  ::**:.***:**::::
```

```
NC_014470.1       TTQSAVLFNNGTHIVIDVCNFNFCADPMFAVNSGQPYK------TWIYTSAANCTYHRA-      169
KF569996.1        KSQSVIIMNNSTNVVIRACNFQLCDNPFFAVIRPTS----QQIETILFENAFNCTFEYVS      169
KY417144.1        KSQSVIIMNNSTNLVIRACNFELCDNPFFVVLKSNN----TQIPSYIFNNAFNCTFEYVS      166
KC881005.1        KSQSVIIMNNSTNLVIRACNFELCDNPFFVVLKSNN----TQIPSYIFNNAFNCTFEYVS      166
KC881006.1        KSQSVIIMNNSTNLVIRACNFELCDNPFFVVLKSNN----TQIPSYIFNNAFNCTFEYVS      166
KF367457.1        KSQSVIIMNNSTNLVIRACNFELCDNPFFVVLKSNN----TQIPSYIFNNAFNCTFEYVS      166
KY417152.1        KSQSVIIMNNSTNLVIRACNFELCDNPFFVVLKSNN----TQIPSYIFNNAFNCTFEYVS      166
KY417151.1        KSQSVIIMNNSTNLVIRACNFELCDNPFFVVLKSNN----TQIPSYIFNNAFNCTFEYVS      166
KY417146.1        KSQSVIIINNSTNVVIRACNFELCDNPFFAVSKPTG----TQTHTMIFDNAFNCTFEYIS      165
KT444582.1        KSQSVIIINNSTNVVIRACNFELCDNPFFAVSKPTG----TQTHTMIFDNAFNCTFEYIS      165
AY278488.2        KSQSVIIINNSTNVVIRACNFELCDNPFFAVSKPMG----TQTHTMIFDNAFNCTFEYIS      165
AY274119          KSQSVIIINNSTNVVIRACNFELCDNPFFAVSKPMG----TQTHTMIFDNAFNCTFEYIS      165
AP006560.1        KSQSVIIINNSTNVVIRACNFELCDNPFFAVSKPMG----TQTHTMIFDNAFNCTFEYIS      165
AP006557.1        KSQSVIIINNSTNVVIRACNFELCDNPFFAVSKPMG----TQTHTMIFDNAFNCTFEYIS      165
AY485277.1        KSQSVIIINNSTNVVIRACNFELCDNPFFAVSKPMG----TQTHTMIFDNAFNCTFEYIS      165
AY572038.1        KSQSVIIINNSTNVVIRACNFELCDNPFFVVSKPMG----TRTHTMIFDNAFNCTFEYIS      165
AY572034.1        KSQSVIIINNSTNVVIRACNFELCDNPFFVVSKPMG----TQTHTMIFDNAFNCTFEYIS      165
MT040334.1        RTQSLLIVNNATNVVIKVCEFQFCTDPFLGVYYHNNNKTWVENEFRVYSSANNCTFEYIS      170
MW532698.1        RTQSLLIVNNATNVVIKVCEFQFCTDPFLGVYYHNNNKTWVENEFRVYSSANNCTFEYIS      172
MT040333.1        RTQSLLIVNNATNVVIKVCEFQFCTDPFLGVYYHNNNKTWVENEFRVYSSANNCTFEYIS      170
MT040336.1        RTQSLLIVNNATNVVIKVCEFQFCTDPFLGVYYHNNNKTWVENEFRVYSSANNCTFEYIS      170
MT040335.1        RTQSLLIVNNATNVVIKVCEFQFCTDPFLGVYYHNNNKTWVENEFRVYSSANNCTFEYIS      170
MN996532.2        KTQSLLIVNNATNVVIKVCEFQFCNDPFLGVYYHKNNKSWMESEFRVYSSANNCTFEYVS      172
MN996529.1        KTQSLLIVNNATNVVIKVCEFQFCNDPFLGVYYHKNNKSWMESEFRVYSSANNCTFEYVS      172
MN988713.1        KTQSLLIVNNATNVVIKVCEFQFCNDPFLGVYYHKNNKSWMESEFRVYSSANNCTFEYVS      172
MT072688.1        KTQSLLIVNNATNVVIKVCEFQFCNDPFLGVYYHKNNKSWMESEFRVYSSANNCTFEYVS      172
MN994467.1        KTQSLLIVNNATNVVIKVCEFQFCNDPFLGVYYHKNNKSWMESEFRVYSSANNCTFEYVS      172
NC_045512.2       KTQSLLIVNNATNVVIKVCEFQFCNDPFLGVYYHKNNKSWMESEFRVYSSANNCTFEYVS      172
MT093571.1        KTQSLLIVNNATNVVIKVCEFQFCNDPFLGVYYHKNNKSWMESEFRVYSSANNCTFEYVS      172
MG772933.1        TSQSLLIVNNATNVIIKVCNFDFCYDPYLSGYYH-NNKTWSIREFAVYSSYANCTFEYVS      171
MG772934.1        TSQSLLIVNNATNVIIKVCNFDFCYDPYLSGYYH-NNKTWSIREFAVYSFYANCTFEYVS      170
KY417145.1        TTQSALLVNNGSAITIQVCYFQFCDNPAFVVTAG-A-----QTSTAIYTNLRNCTYVDTL      162
KP886809.1        TTQSAIIVNNSTHIIIRVCYFNLCKDPMYTVSAG-T-----QISSWVYQNAFNCTYDRVE      169
KY417143.1        TTQSAIIVNNSTHIIIRVCYFNLCKEPMYAISNE-Q-----HYKSWVYQNAYNCTYDRVE      169
FJ588686.1        TTQSAIIVNNSTHIIIRVCYFNLCKEPMYAISNE-Q-----HYKSWVYQNAYNCTYDRVE      169
KY417148.1        TTQSAVIVNNSTHIIIRVCNFNLCKEPMYTVSRG-T-----QQSSWVYQSAFNCTYDRVE      169
KY417147.1        TTQSAVIVNNSTHIIIRVCNFNLCKEPMYTVSRG-T-----QQSSWVYQSAFNCTYDRVE      169
KY417142.1        TTQSAVIVNNSTHIIIRVCNFNLCKEPMYTVSRG-T-----QQSSWVYQSAFNCTYDRVE      169
GQ153542.1        TTQSAVIVNNSTHIIIRVCNFNLCKEPMYTVSRG-T-----QQNSWVYQSAFNCTYDRVE      169
DQ022305          TTQSAVIVNNSTHIIIRVCNFNLCKEPMYTVSRG-T-----QQNAWVYQSAFNCTYDRVE      169
                   :** ::.**.: : * .* *::* :*                   ::    ***:
```

```
NC_014470.1         HAFNISTNMNPGKFKHFREHLFKNVDGFLYVYHNYEPIDLN----SGFPSGFSVLKPILK     225
KF569996.1          DSFLMDVGEKPGNFKHLREFIFKNKDGFLNIYSGFQNIDVA----NGLPSGFSLLKPLLK     225
KY417144.1          KDFNLDLGEKPGNFKDLREFVFRNKDGFLHVYSGYQPISAA----SGLPTGFNALKPIFK     222
KC881005.1          KDFNLDLGEKPGNFKDLREFVFRNKDGFLHVYSGYQPISAA----SGLPTGFNALKPIFK     222
KC881006.1          KDFNLDLGEKPGNFKDLREFVFRNKDGFLHVYSGYQPISAA----SGLPTGFNALKPIFK     222
KF367457.1          KDFNLDLGEKPGNFKDLREFVFRNKDGFLHVYSGYQPISAA----SGLPTGFNALKPIFK     222
KY417152.1          KDFNLDLGEKPGNFKDLREFVFRNKDGFLHVYSGYQPISAA----SGLPTGFNALKPIFK     222
KY417151.1          KDFNLDLGEKPGNFKDLREFVFRNKDGFLHVYSGYQPISAA----SGLPTGFNALKPIFK     222
KY417146.1          DSFSLDVAEKSGNFKHLREFVFKNKDGFLYVYKGYQPIDVV----RDLPSGFNILKPIFK     221
KT444582.1          DSFSLDVAEKSGNFKHLREFVFKNKDGFLYVYKGYQPIDVV----RDLPSGFNILKPIFK     221
AY278488.2          DAFSLDVSEKSGNFKHLREFVFKNKDGFLYVYKGYQPIDVV----RDLPSGFNTLKPIFK     221
AY274119            DAFSLDVSEKSGNFKHLREFVFKNKDGFLYVYKGYQPIDVV----RDLPSGFNTLKPIFK     221
AP006560.1          DAFSLDVSEKSGNFKHLREFVFKNKDGFLYVYKGYQPIDVV----RDLPSGFNTLKPIFK     221
AP006557.1          DAFSLDVSEKSGNFKHLREFVFKNKDGFLYVYKGYQPIDVV----RDLPSGFNTLKPIFK     221
AY485277.1          DAFSLDVSEKSGNFKHLREFVFKNKDGFLYVYKGYQPIDVV----RDLPSGFNTLKPIFK     221
AY572038.1          DAFSLDVSEKSGNFKHLREFVFKNKDGFLYVYKGYQPIDVV----RDLPSGFNTLKPIFK     221
AY572034.1          DAFSLDVSEKSGNFKHLREFVFKNKDGFLYVYKGYQPIDVV----RDLPSGFNTLKPIFK     221
MT040334.1          QPFLMDLEGKQGNFKNLREFVFKNVDGYFKIYSKHTPIDLV----RDLPRGFAALEPLVD     226
MW532698.1          QPFLMDLEGKQGNFKNLREFVFKNVDGYFKIYSKHTPIDLV----RDLPRGFAALEPLVD     228
MT040333.1          QPFLMDLEGKQGNFKNLREFVFKNVDGYFKIYSKHTPIDLV----RDLPRGFAALEPLVD     226
MT040336.1          QPFLMDLEGKQGNFKNLREFVFKNVDGYFKIYSKHTPIDLV----RDLPRGFAALEPLVD     226
MT040335.1          QPFLMDLEGKQGNFKNLREFVFKNVDGYFKIYSKHTPIDLV----RDLPRGFAALEPLVD     226
MN996532.2          QPFLMDLEGKQGNFKNLREFVFKNIDGYFKIYSKHTPINLV----RDLPPGFSALEPLVD     228
MN996529.1          QPFLMDLEGKQGNFKNLREFVFKNIDGYFKIYSKHTPINLV----RDLPQGFSALEPLVD     228
MN988713.1          QPFLMDLEGKQGNFKNLREFVFKNIDGYFKIYSKHTPINLV----RDLPQGFSALEPLVD     228
MT072688.1          QPFLMDLEGKQGNFKNLREFVFKNIDGYFKIYSKHTPINLV----RDLPQGFSALEPLVD     228
MN994467.1          QPFLMDLEGKQGNFKNLREFVFKNIDGYFKIYSKHTPINLV----RDLPQGFSALEPLVD     228
NC_045512.2         QPFLMDLEGKQGNFKNLREFVFKNIDGYFKIYSKHTPINLV----RDLPQGFSALEPLVD     228
MT093571.1          QPFLMDLEGKQGNFKNLREFVFKNIDGYFKIYSKHTPINLV----RDLPQGFSALEPLVD     228
MG772933.1          KSFMLNISGNGGLFNTLREFVFRNVDGHFKIYSKFTPVNLN----RGLPTGLSVLQPLVE     227
MG772934.1          KSFMLNISGNGGLFNTLREFVFRNVDGHFKIYSKFTPVNLN----RGLPTGLSVLQPLVE     226
KY417145.1          RDLPLSLTEVDGGFKHLREFVFKTSDGFLHIYGAYQPYDHAIGATAALPAQFLPLKPLWK     222
KP886809.1          KSFQLDTAPKSGNFIALREFVFKNRDGFFTVYQDYTPVNLL----RGLPAGLSVLKPILK     225
KY417143.1          QSFQLDTAPQTGNFKDLREYVFKNKDGFLSVYNAYSPIDIP----RGLPVGFSVLKPILK     225
FJ588686.1          QSFQLDTAPQTGNFKDLREYVFKNKDGFLSVYNAYSPIDIP----RGLPVGFSVLKPILK     225
KY417148.1          RSFQLDTAPKTGNFKDLREYVFKNRDGFLSVYQTYTAVNLP----RGLPTGFSVLRPILK     225
KY417147.1          RSFQLDTAPKTGNFKDLREYVFKNRDGFLSVYQTYTAVNLP----RGLPTGFSVLRPILK     225
KY417142.1          RSFQLDTAPKTGNFKDLREYVFKNRDGFLSVYQTYTAVNLP----RGLPIGFSVLRPILK     225
GQ153542.1          KSFQLDTVPKTGNFKDLREYVFKNRDGFLSVYQTYTAVNLP----RGLPEGFSVLRPILK     225
DQ022305            KSFQLDTTPKTGNFKDLREYVFKNRDGFLSVYQTYTAVNLP----RGLPTGFSVLKPILK     225
                      : :.     * *  :**.:*:. **.: :*  .   .        :*  :  *.*: .
```

```
NC_014470.1      LPFGLNITYVKAIMTLFS------STQSNFDADASAYFVGHLKPLTMLVDFDENGTIIDA   279
KF569996.1       LPLGLNITNFRVLLTAFI------PNIGTWGTSPVAYFVGYLKPTTFMLKYDYNGTIVDA   279
KY417144.1       LPLGINITNFRTLLTAFP------PRPDYWGTSAAAYFVGYLKPTTFMLKYDENGTITDA   276
KC881005.1       LPLGINITNFRTLLTAFP------PRPDYWGTSAAAYFVGYLKPTTFMLKYDENGTITDA   276
KC881006.1       LPLGINITNFRTLLTAFP------PRPDYWGTSAAAYFVGYLKPTTFMLKYDENGTITDA   276
KF367457.1       LPLGINITNFRTLLTAFP------PRPDYWGTSAAAYFVGYLKPTTFMLKYDENGTITDA   276
KY417152.1       LPLGINITNFRTLLTAFP------PRPDYWGTSAAAYFVGYLKPTTFMLKYDENGTITDA   276
KY417151.1       LPLGINITNFRTLLTAFP------PRPDYWGTSAAAYFVGYLKPTTFMLKYDENGTITDA   276
KY417146.1       LPLGINITNFRAILTAFL------PAQDTWGTSAAAYFVGYLKPATFMLKYDENGTITDA   275
KT444582.1       LPLGINITNFRAILTAFL------PAQDTWGTSAAAYFVGYLKPATFMLKYDENGTITDA   275
AY278488.2       LPLGINITNFRAILTAFS------PAQDTWGTSAAAYFVGYLKPTTFMLKYDENGTITDA   275
AY274119         LPLGINITNFRAILTAFS------PAQDIWGTSAAAYFVGYLKPTTFMLKYDENGTITDA   275
AP006560.1       LPLGINITNFRAILTAFS------PAQDIWGTSAAAYFVGYLKPTTFMLKYDENGTITDA   275
AP006557.1       LPLGINITNFRAILTAFS------PAQDIWGTSAAAYFVGYLKPTTFMLKYDENGTITDA   275
AY485277.1       LPLGINITNFRAILTAFS------PAQDIWGTSAAAYFVGYLKPTTFMLKYDENGTITDA   275
AY572038.1       LPLGINITNFRAILTAFS------PAQDTWGTSAAAYFVGYLKPTTFMLKYDENGTITDA   275
AY572034.1       LPLGIKITNFRAILTAFS------PAQGTWGTSAAAYFVGYLKPTTFMLKYDENGTITDA   275
MT040334.1       LPIGINITRFQTLLALHRSYLTPGKLESGWTTGAAAYYVGYLQQRTFLLSYNQNGTITDA   286
MW532698.1       LPIGINITRFQTLLALHRSYLTPGKLESGWTTGAAAYYVGYLQQRTFLLSYNQNGTITDA   288
MT040333.1       LPIGINITRFQTLLALHRSYLTPGNLESGWTTGAAAYYVGYLQQRTFLLSYNQNGTITDA   286
MT040336.1       LPIGINITRFQTLLALHRSYLTPGKLESGWTTGAAAYYVGYLQQRTFLLSYNQNGTITDA   286
MT040335.1       LPIGINITRFQTLLALHRSYLTPGKLESGWTTGAAAYYVGYLQQRTFLLSYNQNGTITDA   286
MN996532.2       LPIGINITRFQTLLALHRSYLTPGDSSSGWTAGAAAYYVGYLQPRTFLLKYNENGTITDA   288
MN996529.1       LPIGINITRFQTLLALHRSYLTPGDSSSGWTAGAAAYYVGYLQPRTFLLKYNENGTITDA   288
MN988713.1       LPIGINITRFQTLLALHRSYLTPGDSSSGWTAGAAAYYVGYLQPRTFLLKYNENGTITDA   288
MT072688.1       LPIGINITRFQTLLALHRSYLTPGDSSSGWTAGAAAYYVGYLQPRTFLLKYNENGTITDA   288
MN994467.1       LPIGINITRFQTLLALHRSYLTPGDSSSGWTAGAAAYYVGYLQPRTFLLKYNENGTITDA   288
NC_045512.2      LPIGINITRFQTLLALHRSYLTPGDSSSGWTAGAAAYYVGYLQPRTFLLKYNENGTITDA   288
MT093571.1       LPIGINITRFQTLLALHRSYLTPGDSSSGWTAGAAAYYVGYLQPRTFLLKYNENGTITDA   288
MG772933.1       LPVSINITKFRTLLTIHRGDP---MPNNGWTAFSAAYFVGYLKPRTFMLKYNENGTITDA   284
MG772934.1       LPVSINITKFRTLLTIHRGDP---MSNNGWTAFSAAYFVGYLKPRTFMLKYNENGTITDA   283
KY417145.1       LPLGLNITSYKVVTTLKP------TN----QAFQAVYIVGNLKHTTMMLSFNENGTMSNA   272
KP886809.1       LPFGINITSFRVVMAMFS------KTTSNYVPESAAYYVGNLKQSTFMLSFNQNGTITDA   279
KY417143.1       LPIGINITSFKVVMSMFS------RTTSNFLPEIAAYFVGNLKYSTFMLNFNENGTITDA   279
FJ588686.1       LPISINITSFKVVMSMFS------RTTSNFLPEVAAYFVGNLKYSTFMLNFNENGTITDA   279
KY417148.1       LPFGINITSYRVVMAMFS------QTTSNFLPESAAYYVGNLKYTTFMLSFNENGTITDA   279
KY417147.1       LPFGINITSYRVVMAMFS------QTTSNFLPESAAYYVGNLKYTTFMLSFNENGTITDA   279
KY417142.1       LPFGINITSYRVVMAMFS------QTTSNFLPESAAYYVGNLKYTTFMLRFNENGTITDA   279
GQ153542.1       LPFGINITSYRVVMAMFS------QTTSNFLPESAAYYVGNLKYSTFMLRFNENGTITDA   279
DQ022305         LPFGINITSYRVVMAMFS------QTTSNFLPESAAYYVGNLKYSTFMLRFNENGTITDA   279
                 **..::**  :.: :                    .* ** *:  *::: :: ***: :*
```

```
NC_014470.1     IDCSQDPLSELKCTTKSFTVEKGIYQTSNFRVTPTTEVVRFPNITQLCPFNEVFNITSFP     339
KF569996.1      VDCSQHPLAELKCSVKSFEIDKGIYQTSNFRVSPSKEVVRFPNITNLCPFGEVFNATTFP     339
KY417144.1      VDCSQNPLAELKCSVKSFEIDKGIYQTSNFRVAPSKEVVRFPNITNLCPFGEVFNATTFP     336
KC881005.1      VDCSQNPLAELKCSVKSFEIDKGIYQTSNFRVAPSKEVVRFPNITNLCPFGEVFNATTFP     336
KC881006.1      VDCSQNPLAELKCSVKSFEIDKGIYQTSNFRVAPSKEVVRFPNITNLCPFGEVFNATTFP     336
KF367457.1      VDCSQNPLAELKCSVKSFEIDKGIYQTSNFRVAPSKEVVRFPNITNLCPFGEVFNATTFP     336
KY417152.1      VDCSQNPLAELKCSVKSFEIDKGIYQTSNFRVAPSKEVVRFPNITNLCPFGEVFNATTFP     336
KY417151.1      VDCSQNPLAELKCSVKSFEIDKGIYQTSNFRVAPSKEVVRFPNITNLCPFGEVFNATTFP     336
KY417146.1      VDCSQNPLAELKCSVKSFEIDKGIYQTSNFRVAPSKEVVRFPNITNLCPFGEVFNATTFP     335
KT444582.1      VDCSQNPLAELKCSVKSFEIDKGIYQTSNFRVAPSKEVVRFPNITNLCPFGEVFNATTFP     335
AY278488.2      VDCSQNPLAELKCSVKSFEIDKGIYQTSNFRVVPSGDVVRFPNITNLCPFGEVFNATKFP     335
AY274119        VDCSQNPLAELKCSVKSFEIDKGIYQTSNFRVVPSGDVVRFPNITNLCPFGEVFNATKFP     335
AP006560.1      VDCSQNPLAELKCSVKSFEIDKGIYQTSNFRVVPSGDVVRFPNITNLCPFGEVFNATKFP     335
AP006557.1      VDCSQNPLAELKCSVKSFEIDKGIYQTSNFRVVPSGDVVRFPNITNLCPFGEVFNATKFP     335
AY485277.1      VDCSQNPLAELKCSVKSFEIDKGIYQTSNFRVVPSGDVVRFPNITNLCPFGEVFNATKFP     335
AY572038.1      VDCSQNPLAELKCSVKSFEIDKGIYQTSNFRVVPSGDVVRFPNITNLCPFGEVFNATKFP     335
AY572034.1      VDCSQNPLAELKCSVKSFEIDKGIYQTSNFRVVPSGDVVRFPNITNLCPFGEVFNATKFP     335
MT040334.1      VDCSLDPLSETKCTLKSLTVEKGIYQTSNFRVQPTISIVRFPNITNLCPFGEVFNASKFA     346
MW532698.1      VDCSLDPLSETKCTLKSLTVEKGIYQTSNFRVQPTISIVRFPNITNLCPFGEVFNASKFA     348
MT040333.1      VDCSLDPLSETKCTLKSLTVEKGIYQTSNFRVQPTISIVRFPNITNLCPFGEVFNASKFA     346
MT040336.1      VDCSLDPLSETKCTLKSLTVEKGIYQTSNFRVQPTISIVRFPNITNLCPFGEVFNASKFA     346
MT040335.1      VDCSLDPLSETKCTLKSLTVEKGIYQTSNFRVQPTISIVRFPNITNLCPFGEVFNASKFA     346
MN996532.2      VDCALDPLSETKCTLKSFTVEKGIYQTSNFRVQPTDSIVRFPNITNLCPFGEVFNATTFA     348
MN996529.1      VDCALDPLSETKCTLKSFTVEKGIYQTSNFRVQPTESIVRFPNITNLCPFGEVFNATRFA     348
MN988713.1      VDCALDPLSETKCTLKSFTVEKGIYQTSNFRVQPTESIVRFPNITNLCPFGEVFNATRFA     348
MT072688.1      VDCALDPLSETKCTLKSFTVEKGIYQTSNFRVQPTESIVRFPNITNLCPFGEVFNATRFA     348
MN994467.1      VDCALDPLSETKCTLKSFTVEKGIYQTSNFRVQPTESIVRFPNITNLCPFGEVFNATRFA     348
NC_045512.2     VDCALDPLSETKCTLKSFTVEKGIYQTSNFRVQPTESIVRFPNITNLCPFGEVFNATRFA     348
MT093571.1      VDCALDPLSETKCTLKSFTVEKGIYQTSNFRVQPTESIVRFPNITNLCPFGEVFNATRFA     348
MG772933.1      VDCALDPLSETKCTLKSLTVQKGIYQTSNFRVQPTQSVVRFPNITNVCPFHKVFNATRFP     344
MG772934.1      VDCALDPLSETKCTLKSLSVQKGIYQTSNFRVQPTQSIVRFPNITNVCPFHKVFNATRFP     343
KY417145.1      VDCSQDPLAELKCTLKLSDVGKGIYQTSNFRVQPTVDVVRFPNITNLCPFDAVFNATRFP     332
KP886809.1      VDCSQNPLAELKCTTKSFNVSKGIYQTSNFRVAPVTEVVRFPNITNLCPFDKVFNATRFP     339
KY417143.1      IDCAQNPLSELKCTIKNFNVSKGIYQTSNFRVSPTHEVVRFPNITNRCPFDKVFNASRFP     339
FJ588686.1      IDCAQNPLSELKCTIKNFNVSKGIYQTSNFRVSPTHEVIRFPNITNRCPFDKVFNASRFP     339
KY417148.1      IDCAQNPLAELKCTIKNFNVSKGIYQTSNFRVSPTQEVIRFPNITNRCPFDKVFNASRFP     339
KY417147.1      IDCAQNPLAELKCTIKNFNVSKGIYQTSNFRVSPTQEVIRFPNITNRCPFDKVFNASRFP     339
KY417142.1      IDCAQNPLAELKCTIKNFNVSKGIYQTSNFRVSPTQEVVRFPNITNRCPFDKVFNATRFP     339
GQ153542.1      VDCSQNPLAELKCTIKNFNVDKGIYQTSNFRVSPTQEVIRFPNITNRCPFDRVFNASRFP     339
DQ022305        VDCSQNPLAELKCTIKNFNVDKGIYQTSNFRVSPTQEVIRFPNITNRCPFDKVFNATRFP     339
                :**: .**:* **: *   : *********** *  .::******: ***  *** : *
```

```
NC_014470.1     SVYAWERMRITNCVADYSVLYNSSASFSTFQCYGVSPTKLNDLCFSSVYADYFVVKGDDV    399
KF569996.1      SVYAWERKRISNCVADYSVLYNS-TSFSTFKCYGVSAIKLNDLCFSNVYADSFVVKGDDV    398
KY417144.1      SVYAWERKRISNCVADYSILYNS-TSFSTFKCYGVSATKLNDLCFSNVYADSFVVKGDDV    395
KC881005.1      SVYAWERKRISNCVADYSVLYNS-TSFSTFKCYGVSATKLNDLCFSNVYADSFVVKGDDV    395
KC881006.1      SVYAWERKRISNCVADYSVLYNS-TSFSTFKCYGVSATKLNDLCFSNVYADSFVVKGDDV    395
KF367457.1      SVYAWERKRISNCVADYSVLYNS-TSFSTFKCYGVSATKLNDLCFSNVYADSFVVKGDDV    395
KY417152.1      SVYAWERKRISNCVADYSVLYNS-TSFSTFKCYGVSATKLNDLCFSNVYADSFVVKGDDV    395
KY417151.1      SVYAWERKRISNCVADYSVLYNS-TSFSTFKCYGVSATKLNDLCFSNVYADSFVVKGDDV    395
KY417146.1      SVYAWERKRISNCVADYSVLYNS-TSFSTFKCYGVSATKLNDLCFSNVYADSFVVKGDDV    394
KT444582.1      SVYAWERKRISNCVADYSVLYNS-TSFSTFKCYGVSATKLNDLCFSNVYADSFVVKGDDV    394
AY278488.2      SVYAWERKKISNCVADYSVLYNS-TFFSTFKCYGVSATKLNDLCFSNVYADSFVVKGDDV    394
AY274119        SVYAWERKKISNCVADYSVLYNS-TFFSTFKCYGVSATKLNDLCFSNVYADSFVVKGDDV    394
AP006560.1      SVYAWERKKISNCVADYSVLYNS-TFFSTFKCYGVSATKLNDLCFSNVYADSFVVKGDDV    394
AP006557.1      SVYAWERKKISNCVADYSVLYNS-TFFSTFKCYGVSATKLNDLCFSNVYADSFVVKGDDV    394
AY485277.1      SVYAWERKKISNCVADYSVLYNS-TFFSTFKCYGVSATKLNDLCFSNVYADSFVVKGDDV    394
AY572038.1      SVYAWERKRISNCVADYSVLYNS-TSFSTFKCYGVSATKLNDLCFSNVYADSFVVKGDDV    394
AY572034.1      SVYAWERKRISNCVADYSVLYNS-TSFSTFKCYGVSATKLNDLCFSNVYADSFVVKGDDV    394
MT040334.1      SVYAWNRKRISNCVADYSVLYNS-TSFSTFKCYGVSPTKLNDLCFTNVYADSFVVKGDEV    405
MW532698.1      SVYAWNRKRISNCVADYSVLYNS-TSFSTFKCYGVSPTKLNDLCFTNVYADSFVVKGDEV    407
MT040333.1      SVYAWNRKRISNCVADYSVLYNS-TSFSTFKCYGVSPTKLNDLCFTNVYADSFVVKGDEV    405
MT040336.1      SVYAWNRKRISNCVADYSVLYNS-TSFSTFKCYGVSPTKLNDLCFTNVYADSFVVKGDEV    405
MT040335.1      SVYAWNRKRISNCVADYSVLYNS-TSFSTFKCYGVSPTKLNDLCFTNVYADSFVVKGDEV    405
MN996532.2      SVYAWNRKRISNCVADYSVLYNS-TSFSTFKCYGVSPTKLNDLCFTNVYADSFVITGDEV    407
MN996529.1      SVYAWNRKRISNCVADYSVLYNS-ASFSTFKCYGVSPTKLNDLCFTNVYADSFVIRGDEV    407
MN988713.1      SVYAWNRKRISNCVADYSVLYNS-ASFSTFKCYGVSPTKLNDLCFTNVYADSFVIRGDEV    407
MT072688.1      SVYAWNRKRISNCVADYSVLYNS-ASFSTFKCYGVSPTKLNDLCFTNVYADSFVIRGDEV    407
MN994467.1      SVYAWNRKRISNCVADYSVLYNS-ASFSTFKCYGVSPTKLNDLCFTNVYADSFVIRGDEV    407
NC_045512.2     SVYAWNRKRISNCVADYSVLYNS-ASFSTFKCYGVSPTKLNDLCFTNVYADSFVIRGDEV    407
MT093571.1      SVYAWNRKRISNCVADYSVLYNS-ASFSTFKCYGVSPTKLNDLCFTNVYADSFVIRGDEV    407
MG772933.1      SVYAWERTKISDCIADYTVFYNS-TSFSTFKCYGVSPSKLIDLCFTSVYADTFLIRFSEV    403
MG772934.1      SVYAWERTKISDCIADYTVFYNS-TSFSTFKCYGVSPSKLIDLCFTSVYADTFLIRFSEV    402
KY417145.1      SVYAWERVKISNCVADYTAFYNS-TSFSTFKCYGVSPSKLIDLCFTSVYADTFLIRFSEV    391
KP886809.1      SVYAWERTKISDCVADYTVFYNS-TSFSTFNCYGVSPSKLIDLCFTSVYADTFLIRFSEV    398
KY417143.1      NVYAWERTKISDCVADYTVLYNS-TSFSTFKCYGVSPSKLIDLCFTSVYADTFLIRSSEV    398
FJ588686.1      NVYAWERTKISDCVADYTVLYNS-TSFSTFKCYGVSPSKLIDLCFTSVYADTFLIRSSEV    398
KY417148.1      NVYAWERTKISDCVADYTVLYNS-TSFSTFKCYGVSPSKLIDLCFTSVYADTFLIRSSEV    398
KY417147.1      NVYAWERTKISDCVADYTVLYNS-TSFSTFKCYGVSPSKLIDLCFTSVYADTFLIRSSEV    398
KY417142.1      SVYAWERTKISDCVADYTVLYNS-TSFSTFKCYGVSPSKLIDLCFTSVYADTFLIRSSEV    398
GQ153542.1      SVYAWERTKISECVADYTVLYNS-TSFSTFKCYGVSPSKLIDLCFTSVYADTFLIRSSEV    398
DQ022305        NVYAWERTKISDCVADYTVLYNS-TSFSTFKCYGVSPSKLIDLCFTSVYADTFLIRSSEV    398
                .****:* :*::*:***: :*** : ****:*****  ** ****:.**** *::  .:*
```

```
NC_014470.1       RQIAPAQTGVIADYNYKLPDDFTGCVIAWNTNSLDSS----NEFFYRRFRHGKIKPYGRD    455
KF569996.1        RQIAPGQTGVIADYNYKLPDDFMGCVLAWNTRNIDATSSGNFNYKYRSLRHGKLRPFERD    458
KY417144.1        RQIAPGQTGVIADYNYKLPDDFLGCVLAWNTNSKDSSTSGNYNYLYRWVRRSKLNPYERD    455
KC881005.1        RQIAPGQTGVIADYNYKLPDDFLGCVLAWNTNSKDSSTSGNYNYLYRWVRRSKLNPYERD    455
KC881006.1        RQIAPGQTGVIADYNYKLPDDFTGCVLAWNTRNIDATQTGNYNYKYRSLRHGKLRPFERD    455
KF367457.1        RQIAPGQTGVIADYNYKLPDDFTGCVLAWNTRNIDATQTGNYNYKYRSLRHGKLRPFERD    455
KY417152.1        RQIAPGQTGVIADYNYKLPDDFMGCVLAWNTRNIDATSTGNYNYKYRSLRHGKLRPFERD    455
KY417151.1        RQIAPGQTGVIADYNYKLPDDFMGCVLAWNTRNIDATSTGNYNYKYRSLRHGKLRPFERD    455
KY417146.1        RQIAPGQTGVIADYNYKLPDDFLGCVLAWNTNSKDSSTSGNYNYLYRWVRRSKLNPYERD    454
KT444582.1        RQIAPGQTGVIADYNYKLPDDFTGCVLAWNTRNIDATQTGNYNYKYRSLRHGKLRPFERD    454
AY278488.2        RQIAPGQTGVIADYNYKLPDDFMGCVLAWNTRNIDATSTGNYNYKYRYLRHGKLRPFERD    454
AY274119          RQIAPGQTGVIADYNYKLPDDFMGCVLAWNTRNIDATSTGNYNYKYRYLRHGKLRPFERD    454
AP006560.1        RQIAPGQTGVIADYNYKLPDDFMGCVLAWNTRNIDATSTGNYNYKYRYLRHGKLRPFERD    454
AP006557.1        RQIAPGQTGVIADYNYKLPDDFMGCVLAWNTRNIDATSTGNYNYKYRYLRHGKLRPFERD    454
AY485277.1        RQIAPGQTGVIADYNYKLPDDFMGCVLAWNTRNIDATSTGNYNYKYRSLRHGKLRPFERD    454
AY572038.1        RQIAPGQTGVIADYNYKLPDDFMGCVLAWNTRNIDATSTGNYNYKYRYLRHGKLRPFERD    454
AY572034.1        RQIAPGQTGVIADYNYKLPDDFMGCVLAWNTRNIDATSTGNYNYKYRYLRHGKLRPFERD    454
MT040334.1        RQIAPGQTGVIADYNYKLPDDFTGCVIAWNSVKQDALTGGNY--LYRLFRKSKLKPFERD    463
MW532698.1        RQIAPGQTGVIADYNYKLPDDFTGCVIAWNSVKQDALTGGNYGYLYRLFRKSKLKPFERD    467
MT040333.1        RQIAPGQTGVIADYNYKLPDDFTGCVIAWNSVKQDALTGGNYGYLYRLFRKSKLKPFERD    465
MT040336.1        RQIAPGQTGVIADYNYKLPDDFTGCVIAWNSVKQDALTGDNYGYLYRLFRKSKLKPFERD    465
MT040335.1        RQIAPGQTGVIADYNYKLPDDFTGCVIAWNSVKQDALTGGNYGYLYRLFRKSKLKPFERD    465
MN996532.2        RQIAPGQTGKIADYNYKLPDDFTGCVIAWNSKHIDAKEGGNFNYLYRLFRKANLKPFERD    467
MN996529.1        RQIAPGQTGKIADYNYKLPDDFTGCVIAWNSNNLDSKVGGNYNYLYRLFRKSNLKPFERD    467
MN988713.1        RQIAPGQTGKIADYNYKLPDDFTGCVIAWNSNNLDSKVGGNYNYLYRLFRKSNLKPFERD    467
MT072688.1        RQIAPGQTGKIADYNYKLPDDFTGCVIAWNSNNLDSKVGGNYNYLYRLFRKSNLKPFERD    467
MN994467.1        RQIAPGQTGKIADYNYKLPDDFTGCVIAWNSNNLDSKVGGNYNYLYRLFRKSNLKPFERD    467
NC_045512.2       RQIAPGQTGKIADYNYKLPDDFTGCVIAWNSNNLDSKVGGNYNYLYRLFRKSNLKPFERD    467
MT093571.1        RQIAPGQTGKIADYNYKLPDDFTGCVIAWNSNNLDSKVGGNYNYLYRLFRKSNLKPFERD    467
MG772933.1        RQVAPGQTGVIADYNYKLPDDFTGCVIAWNTAKQDV-----GNYFYRSHRSTKLKPFERD    458
MG772934.1        RQVAPGQTGVIADYNYKLPDDFTGCVIAWNTAKQDT-----GHYFYRSHRSTKLKPFERD    457
KY417145.1        RQVAPGETGVIADYNYKLPDDFTGCVIAWNTAKQDV-----GSYFYRSHRSSKLKPFERD    446
KP886809.1        RQVAPGQTGVIADYNYKLPDDFTGCVIAWNTAKYDV-----GSYFYRSHRSSKLKPFERD    453
KY417143.1        RQVAPGETGVIADYNYKLPDDFTGCVIAWNTAKQDQ-----GQYYYRSSRKTKLKPFERD    453
FJ588686.1        RQVAPGETGVIADYNYKLPDDFTGCVIAWNTAKQDQ-----GQYYYRSSRKTKLKPFERD    453
KY417148.1        RQVAPGETGVIADYNYKLPDDFTGCVIAWNTAKQDT-----GHYYYRSHRKTKLKPFERD    453
KY417147.1        RQVAPGETGVIADYNYKLPDDFTGCVIAWNTAKQDQ-----GQYYYRSSRKTKLKPFERD    453
KY417142.1        RQVAPGETGVIADYNYKLPDDFTGCVIAWNTAQQDK-----GQYYYRSSRKTKLKPFERD    453
GQ153542.1        RQVAPGETGVIADYNYKLPDDFTGCVIAWNTAKQDT-----GNYYYRSHRKTKLKPFERD    453
DQ022305          RQVAPGETGVIADYNYKLPDDFTGCVIAWNTAKHDT-----GNYYYRSHRKTKLKPFERD    453
                  **:**.:** ************ ***:***:   *          **  *  ::.*: **
```

```
NC_014470.1       LSNVLFNPSGGTCSA-EGLNCYKPLASYGFTQSSGIGFQPYRVVVLSFELLNAPATVCGP    514
KF569996.1        ISNVPFSPDGKPCTP-PAFNCYWPLNDYGFYTTNGIGYQPYRVVVLSFELLNAPATVCGP    517
KY417144.1        LSNDIYSPGGQSCSA-VGPNCYNPLRPYGFFTTAGVGHQPYRVVVLSFELLNAPATVCGP    514
KC881005.1        LSNDIYSPGGQSCSA-VGPNCYNPLRPYGFFTTAGVGHQPYRVVVLSFELLNAPATVCGP    514
KC881006.1        ISNVPFSPDGKPCTP-PAFNCYWPLNDYGFYITNGIGYQPYRVVVLSFELLNAPATVCGP    514
KF367457.1        ISNVPFSPDGKPCTP-PAFNCYWPLNDYGFYITNGIGYQPYRVVVLSFELLNAPATVCGP    514
KY417152.1        ISNVPFSPDGKPCTP-PAFNCYWPLNDYGFFTTNGIGYQPYRVVVLSFELLNAPATVCGP    514
KY417151.1        ISNVPFSPDGKPCTP-PAFNCYWPLNDYGFFTTNGIGYQPYRVVVLSFELLNAPATVCGP    514
KY417146.1        LSNDIYSPGGQSCSA-IGPNCYNPLRPYGFFTTAGVGHQPYRVVVLSFELLNAPATVCGP    513
KT444582.1        ISNVPFSPDGKPCTP-PAFNCYWPLNDYGFYITNGIGYQPYRVVVLSFELLNAPATVCGP    513
AY278488.2        ISNVPFSPDGKPCTP-PALNCYWPLNDYGFYTTTGIGYQPYRVVVLSFELLNAPATVCGP    513
AY274119          ISNVPFSPDGKPCTP-PALNCYWPLNDYGFYTTTGIGYQPYRVVVLSFELLNAPATVCGP    513
AP006560.1        ISNVPFSPDGKPCTP-PALNCYWPLNDYGFYTTTGIGYQPYRVVVLSFELLNAPATVCGP    513
AP006557.1        ISNVPFSPDGKPCTP-PALNCYWPLNDYGFYTTTGIGYQPYRVVVLSFELLNAPATVCGP    513
AY485277.1        ISNVPFSPDGKPCTP-PALNCYWPLNDYGFYTTTGIGYQPYRVVVLSFELLNAPATVCGP    513
AY572038.1        ISNVPFSPDGKPCTP-PAPNCYWPLRGYGFYTTSGIGYQPYRVVVLSFELLNAPATVCGP    513
AY572034.1        ISNVPFSSDGKPCTP-PAPNCYWPLRGYGFYTTSGIGYQPYRVVVLSFELLNAPATVCGP    513
MT040334.1        ISTEIYQAGSTPCNGQVGLNCYYPLERYGFHPTTGVNYQPFRVVVLSFELLNGPATVCGP    523
MW532698.1        ISTEIYQAGSTPCNGQVGLNCYYPLERYGFHPTTGVNYQPFRVVVLSFELLNGPATVCGP    527
MT040333.1        ISTEIYQAGSTPCNGQVGLNCYYPLERYGFHPTTGVNYQPFRVVVLSFELLNGPATVCGP    525
MT040336.1        ISTEIYQAGSTPCNGQVGLNCYYPLERYGFHPTTGVNYQPFRVVVLSFELLNGPATVCGP    525
MT040335.1        ISTEIYQAGSTPCNGQVGLNCYYPLERYGFHPTTGVNYQPFRVVVLSFELLNGPATVCGP    525
MN996532.2        ISTEIYQAGSKPCNGQTGLNCYYPLYRYGFYPTDGVGHQPYRVVVLSFELLNAPATVCGP    527
MN996529.1        ISTEIYQAGSTPCNGVEGFNCYFPLQSYGFQPTNGVGYQPYRVVVLSFELLHAPATVCGP    527
MN988713.1        ISTEIYQAGSTPCNGVEGFNCYFPLQSYGFQPTNGVGYQPYRVVVLSFELLHAPATVCGP    527
MT072688.1        ISTEIYQAGSTPCNGVEGFNCYFPLQSYGFQPTNGVGYQPYRVVVLSFELLHAPATVCGP    527
MN994467.1        ISTEIYQAGSTPCNGVEGFNCYFPLQSYGFQPTNGVGYQPYRVVVLSFELLHAPATVCGP    527
NC_045512.2       ISTEIYQAGSTPCNGVEGFNCYFPLQSYGFQPTNGVGYQPYRVVVLSFELLHAPATVCGP    527
MT093571.1        ISTEIYQAGSTPCNGVEGFNCYFPLQSYGFQPTNGVGYQPYRVVVLSFELLHAPATVCGP    527
MG772933.1        LSSD-E-------------NGVRTLSTYDFNPNVPLEYQATRVVVLSFELLNAPATVCGP    504
MG772934.1        LSSD-E-------------NGVRTLSTYDFNPNVPLEYQATRVVVLSFELLNAPATVCGP    503
KY417145.1        LSSD-E-------------NGVRTLSTYDFNPNVPLDYQATRVVVLSFELLNAPATVCGP    492
KP886809.1        LSSE-E-------------NGARTLSTYDFNQNVPLEYQATRVVVLSFELLNAPATVCGP    499
KY417143.1        LTSD-E-------------NGVRTLSTYDFYPNVPIEYQATRVVVLSFELLNAPATVCGP    499
FJ588686.1        LTSD-E-------------NGVRTLSTYDFYPNVPIEYQATRVVVLSFELLNAPATVCGP    499
KY417148.1        LSSDDG-------------NGVYTLSTYDFNPNVPVAYQATRVVVLSFELLNAPATVCGP    500
KY417147.1        LSSD-E-------------NGVRTLSTYDFYPTVPIEYQATRVVVLSFELLNAPATVCGP    499
KY417142.1        LSSD-E-------------NGVRTLSTYDFYPTVPIEYQATRVVVLSFELLNAPATVCGP    499
GQ153542.1        LSSDDG-------------NGVYTLSTYDFNPNVPVAYQATRVVVLSFELLNAPATVCGP    500
DQ022305          LSSDDG-------------NGVYTLSTYDFNPNVPVAYQATRVVVLSFELLNAPATVCGP    500
                  ::.                *    *  *.*  .  : .*  **********:.*******
```

```
NC_014470.1       KQSTELVKNKCVNFNFNGLTGTGVLTNSTKKFQPFQQFGRDVSDFTDSVRDPKTLEILDI    574
KF569996.1        KLSTDLITNQCVNFNFNGLTGTGVLTPSLKRFQPFQQFGRDFSDFTDSVRDPKTLEVLDI    577
KY417144.1        KLSTDLIKNQCVNFNFNGLTGTGVLTPSSKRFQPFQQFGRDVSDFTDSVRDPKTSEILDI    574
KC881005.1        KLSTDLIKNQCVNFNFNGLTGTGVLTPSSKRFQPFQQFGRDVSDFTDSVRDPKTSEILDI    574
KC881006.1        KLSTDLIKNQCVNFNFNGLTGTGVLTPSSKRFQPFQQFGRDVSDFTDSVRDPKTSEILDI    574
KF367457.1        KLSTDLIKNQCVNFNFNGLTGTGVLTPSSKRFQPFQQFGRDVSDFTDSVRDPKTSEILDI    574
KY417152.1        KLSTDLIKNQCVNFNFNGLTGTGVLTSSSKRFQPFQQFGRDVSDFTDSVRDPKTSEILDI    574
KY417151.1        KLSTDLIKNQCVNFNFNGLTGTGVLTSSSKRFQPFQQFGRDVSDFTDSVRDPKTSEILDI    574
KY417146.1        KLSTDLIKNQCVNFNFNGLTGTGVLTSSSKRFQPFQQFGRDVSDFTDSVRDPKTSEILDI    573
KT444582.1        KLSTDLIKNQCVNFNFNGLTGTGVLTPSSKRFQPFQQFGRDVLDFTDSVRDPKTSEILDI    573
AY278488.2        KLSTDLIKNQCVNFNFNGLTGTGVLTPSSKRFQPFQQFGRDVSDFTDSVRDPKTSEILDI    573
AY274119          KLSTDLIKNQCVNFNFNGLTGTGVLTPSSKRFQPFQQFGRDVSDFTDSVRDPKTSEILDI    573
AP006560.1        KLSTDLIKNQCVNFNFNGLTGTGVLTPSSKRFQPFQQFGRDVSDFTDSVRDPKTSEILDI    573
AP006557.1        KLSTDLIKNQCVNFNFNGLTGTGVLTPSSKRFQPFQQFGRDVSDFTDSVRDPKTSEILDI    573
AY485277.1        KLSTDLIKNQCVNFNFNGLTGTGVLTPSSKRFQPFQQFGRDVSDFTDSVRDPKTSEILDI    573
AY572038.1        KLSTDLIKNQCVNFNFNGLTGTGVLTPSSKRFQPFQQFGRDVSDFTDSVRDPKTSEILDI    573
AY572034.1        KLSTDLIKNQCVNFNFNGLTGTGVLTPSSKRFQPFQQFGRDVSDFTDSVRDPKTSEILDI    573
MT040334.1        KLSTTLVKDKCVNFNFNGLTGTGVLTTSKKQFLPFQQFGRDISDTTDAVRDPQTLEILDI    583
MW532698.1        KLSTTLVKDKCVNFNFNGLTGTGVLTTSKKQFLPFQQFGRDISDTTDAVRDPQTLEILDI    587
MT040333.1        KLSTTLVKDKCVNFNFNGLTGTGVLTTSKKQFLPFQQFGRDISDTTDAVRDPQTLEILDI    585
MT040336.1        KLSTTLVKDKCVNFNFNGLTGTGVLTTSKKQFLPFQQFGRDISDTTDAVRDPQTLEILDI    585
MT040335.1        KLSTTLVKDKCVNFNFNGLTGTGVLTTSKKQFLPFQQFGRDISDTTDAVRDPQTLEILDI    585
MN996532.2        KKSTNLVKNKCVNFNFNGLTGTGVLTESNKKFLPFQQFGRDIADTTDAVRDPQTLEILDI    587
MN996529.1        KKSTNLVKNKCVNFNFNGLTGTGVLTESNKKFLPFQQFGRDIADTTDAVRDPQTLEILDI    587
MN988713.1        KKSTNLVKNKCVNFNFNGLTGTGVLTESNKKFLPFQQFGRDIADTTDAVRDPQTLEILDI    587
MT072688.1        KKSTNLVKNKCVNFNFNGLTGTGVLTESNKKFLPFQQFGRDIADTTDAVRDPQTLEILDI    587
MN994467.1        KKSTNLVKNKCVNFNFNGLTGTGVLTESNKKFLPFQQFGRDIADTTDAVRDPQTLEILDI    587
NC_045512.2       KKSTNLVKNKCVNFNFNGLTGTGVLTESNKKFLPFQQFGRDIADTTDAVRDPQTLEILDI    587
MT093571.1        KKSTNLVKNKCVNFNFNGLTGTGVLTESNKKFLPFQQFGRDIADTTDAVRDPQTLEILDI    587
MG772933.1        KLSTQLVKNQCVNFNFNGLKGTGVLTDSSKRFQSFQQFGKDASDFIDSVRDPQTLEILDI    564
MG772934.1        KLSTQLVKNQCVNFNFNGLKGTGVLTDSSKRFQSFQQFGKDASDFIDSVRDPQTLEILDI    563
KY417145.1        KLSTQLVKNQCVNFNFNGLKGTGVLTDSSKRFQSFQQFGRDTSDFTDSVRDPQTLDILDI    552
KP886809.1        KLSTSLVKNQCVNFNFNGFKGTGVLTDSSKTFQSFQQFGRDASDFTDSVRDPKTLQILDI    559
KY417143.1        KLSTALVKNQCVNFNFNGLKGIGVLTDSSKRFQSFQQFGRDTSDFTDSVRDPQTLQILDI    559
FJ588686.1        KLSTGLVKNQCVNFNFNGLKGTGVLTDSSKRFQSFQQFGRDTSDFTDSVRDPQTLQILDI    559
KY417148.1        KLSTQLVKNQCVNFNFNGLKGTGVLTDSSKRFQSFQQFGRDTSDFTDSVRDPQTLEILDI    560
KY417147.1        KLSTGLVKNQCVNFNFNGLKGTGVLTDSSKRFQSFQQFGRDMSDFTDSVRDPQTLQILDI    559
KY417142.1        KLSTGLVKNQCVNFNFNGLKGTGVLTDSSKRFQSFQQFGRDTSDFTDSVRDPQTLQVLDI    559
GQ153542.1        KLSTQLVKNQCVNFNFNGLKGTGVLTPSSKRFQSFQQFGRDTSDFTDSVRDPQTLEILDI    560
DQ022305          KLSTELVKNQCVNFNFNGLKGTGVLTSSSKRFQSFQQFGRDTSDFTDSVRDPQTLEILDI    560
                  * ** *:.::********:.* **** * * *  *****:*  *  *:****:* ::***
```

```
NC_014470.1      APCSYGGVSVITPGTNASSSVAVLYQDVNCTDVPTMLHADQISHDWRVYAFRNDGNIFQT      634
KF569996.1       SPCSFGGVSVITPGTNISSEVAVLYQDVNCTDVPTAIHADQLTPAWRIYSA--GVNVFQT      635
KY417144.1       SPCSFGGVSVITPGTNTSSEVAVLYQDVNCTDVPVAIHADQLTPSWRVYST--GNNVFQT      632
KC881005.1       SPCSFGGVSVITPGTNTSSEVAVLYQDVNCTDVPVAIHADQLTPSWRVYST--GNNVFQT      632
KC881006.1       SPCSFGGVSVITPGTNTSSEVAVLYQDVNCTDVPVAIHADQLTPSWRVYST--GNNVFQT      632
KF367457.1       SPCSFGGVSVITPGTNTSSEVAVLYQDVNCTDVPVAIHADQLTPSWRVHST--GNNVFQT      632
KY417152.1       SPCSFGGVSVITPGTNTSSEVAVLYQDVNCTDVPVAIHADQLTPAWRIYST--GNNVFQT      632
KY417151.1       SPCSFGGVSVITPGTNTSSEVAVLYQDVNCTDVPVAIHADQLTPAWRIYST--GNNVFQT      632
KY417146.1       SPCSFGGVSVITPGTNTSSEVAVLYQDVNCTDVPVAIHADQLTPAWRIYST--GNNVFQT      631
KT444582.1       SPCSFGGVSVITPGTNTSSEVAVLYQDVNCTDVPVAIHADQLTPSWRVYST--GNNVFQT      631
AY278488.2       SPCSFGGVSVITPGTNASSEVAVLYQDVNCTDVSTAIHADQLTPAWRIYST--GNNVFQT      631
AY274119         SPCAFGGVSVITPGTNASSEVAVLYQDVNCTDVSTAIHADQLTPAWRIYST--GNNVFQT      631
AP006560.1       SPCSFGGVSVITPGTNASSEVAVLYQDVNCTDVSTAIHADQLTPAWRIYST--GNNVFQT      631
AP006557.1       SPCSFGGVSVITPGTNASSEVAVLYQDVNCTDVSTAIHADQLTPAWRIYST--GNNVFQT      631
AY485277.1       SPCSFGGVSVITPGTNASSEVAVLYQDVNCTDVSTAIHADQLTPAWRIYST--GNNVFQT      631
AY572038.1       SPCSFGGVSVITPGTNASSEVAVLYQDVNCTDVSTLIHAEQLTPAWRIYST--GNNVFQT      631
AY572034.1       SPCSFGGVSVITPGTNASSEVAVLYQDVNCTDVSTLIHAEQLTPAWRIYST--GNNVFQT      631
MT040334.1       TPCSFGGVSVITPGTNTSNQVAVLYQDVNCTEVPMAIHAEQLTPAWRVYSA--GANVFQT      641
MW532698.1       TPCSFGGVSVITPGTNTSNQVAVLYQDVNCTEVPMAIHAEQLTPAWRVYSA--GANVFQT      645
MT040333.1       TPCSFGGVSVITPGTNTSNQVAVLYQDVNCTEVPMAIHAEQLTPAWRVYSA--GANVFQT      643
MT040336.1       TPCSFGGVSVITPGTNTSNQVAVLYQDVNCTEVPMAIHAEQLTPAWRVYSA--GANVFQT      643
MT040335.1       TPCSFGGVSVITPGTNTSNQVAVLYQDVNCTEVPMAIHAEQLTPAWRVYSA--GANVFQT      643
MN996532.2       TPCSFGGVSVITPGTNASNQVAVLYQDVNCTEVPVAIHADQLTPTWRVYST--GSNVFQT      645
MN996529.1       TPCSFGGVSVITPGTNTSNQVAVLYQDVNCTEVPVAIHADQLTPTWRVYST--GSNVFQT      645
MN988713.1       TPCSFGGVSVITPGTNTSNQVAVLYQDVNCTEVPVAIHADQLTPTWRVYST--GSNVFQT      645
MT072688.1       TPCSFGGVSVITPGTNTSNQVAVLYQDVNCTEVPVAIHADQLTPTWRVYST--GSNVFQT      645
MN994467.1       TPCSFGGVSVITPGTNTSNQVAVLYQDVNCTEVPVAIHADQLTPTWRVYST--GSNVFQT      645
NC_045512.2      TPCSFGGVSVITPGTNTSNQVAVLYQDVNCTEVPVAIHADQLTPTWRVYST--GSNVFQT      645
MT093571.1       TPCSFGGVSVITPGTNTSNQVAVLYQDVNCTEVPVAIHADQLTPTWRVYST--GSNVFQT      645
MG772933.1       TPCSFGGVSVITPGTNTSLEVAVLYQDVNCTDVPTTIHADQLTPAWRIYAT--GTNVFQT      622
MG772934.1       TPCSFGGVSVITPGTNTSSEVAVLYQDVNCTDVPTTIHADQLTPAWRIYAI--GTSVFQT      621
KY417145.1       TPCSFGGVSVITPGTNASSEVAVLYQDVNCTDVPTVIRADQLTPAWRVYST--GVNVFQT      610
KP886809.1       SPCSFGGVSVITPGTNTSSAVAVLYQDVNCTDVPKTIHADQLAPSWRVYTS--GPFVFQT      617
KY417143.1       TPCSFGGVSVITPGTNASSEVAVLYQDVNCTDVPTAIRADQLTPAWRVYST--GINVFQT      617
FJ588686.1       TPCSFGGVSVITPGTNASSEVAVLYQDVNCTDVPTAIRADQLTPAWRVYST--GVNVFQT      617
KY417148.1       TPCSFGGVSVITPGTNASSEVAVLYQDVNCTDVPTAIRADQLTPAWRVYST--GINVFQT      618
KY417147.1       TPCSFGGVSVITPGTNASSEVAVLYQDVNCTDVPTAIRADQLTPAWRVYST--GINVFQT      617
KY417142.1       TPCSFGGVSVITPGTNASSEVAVLYQDVNCTDVPTAIRADQLTPAWRVYST--GVNVFQT      617
GQ153542.1       SPCSFGGVSVITPGTNASSEVAVLYQDVNCTDVPTAIRADQLTPAWRVYST--GVNVFQT      618
DQ022305         SPCSFGGVSVITPGTNASSEVAVLYQDVNCTDVPTAIRADQLTPAWRVYST--GVNVFQT      618
                 :**::*********** *  ***********:*   ::*:*::  **:::   .  :***
```

```
NC_014470.1     QAGCLIGAAYDNSSYECDIPIGAGICAKYTNVSS---TLVRSGGHSILAYTMSLGDNQDI    691
KF569996.1      QAGCLIGAEHVNASYECDIPIGAGICASYHTAS----LLRNTDQKSIVAYTMSLGAENSI    691
KY417144.1      QAGCLIGAEHVDTSYECDIPIGAGICASYHTVS----SLRSTSQKSIVAYTMSLGADSSI    688
KC881005.1      QAGCLIGAEHVDTSYECDIPIGAGICASYHTVS----SLRSTSQKSIVAYTMSLGADSSI    688
KC881006.1      QAGCLIGAEHVDTSYECDIPIGAGICASYHTVS----SLRSTSQKSIVAYTMSLGADSSI    688
KF367457.1      QAGCLIGAEHVDTSYECDIPIGAGICASYHTVS----SLRSTSQKSIVAYTMSLGADSSI    688
KY417152.1      QAGCLIGAEHVDTSYECDIPIGAGICASYHTVS----SLRSTSQKSIVAYTMSLGADSSI    688
KY417151.1      QAGCLIGAEHVDTSYECDIPIGAGICASYHTVS----SLRSTSQKSIVAYTMSLGADSSI    688
KY417146.1      QAGCLIGAEHVDTSYECDIPIGAGICASYHTVS----SLRSTSQKSIVAYTMSLGADSSI    687
KT444582.1      QAGCLIGAEHVDTSYECDIPIGAGICASYHTVS----SLRSTSQKSIVAYTMSLGADSSI    687
AY278488.2      QAGCLIGAEHVDTSYECDIPIGAGICASYHTVS----LLRSTSQKSIVAYTMSLGADSSI    687
AY274119        QAGCLIGAEHVDTSYECDIPIGAGICASYHTVS----LLRSTSQKSIVAYTMSLGADSSI    687
AP006560.1      QAGCLIGAEHVDTSYECDIPIGAGICASYHTVS----LLRSTSQKSIVAYTMSLGADSSI    687
AP006557.1      QAGCLIGAEHVDTSYECDIPIGAGICASYHTVS----LLRSTSQKSIVAYTMSLGADSSI    687
AY485277.1      QAGCLIGAEHVDTSYECDIPIGAGICASYHTVS----LLRSTSQKSIVAYTMSLGADSSI    687
AY572038.1      QAGCLIGAEHVDTSYECDIPIGAGICASYHTVS----SLRSTSQKSIVAYTMSLGADSSI    687
AY572034.1      QAGCLIGAEHVDTSYECDIPIGAGICASYHTVS----SLRSTSQKSIVAYTMSLGADSSI    687
MT040334.1      RAGCLVGAEHVNNSYECDIPVGAGICASYHSMS----SLRSVNQRSIIAYTMSLGAENSV    697
MW532698.1      RAGCLVGAEHVNNSYECDIPVGAGICASYHSMS----SFRSVNQRSIIAYTMSLGAENSV    701
MT040333.1      RAGCLVGAEHVNNSYECDIPVGAGICASYHSMS----SLRSVNQRSIIAYTMSLGAENSV    699
MT040336.1      RAGCLVGAEHVNNSYECDIPVGAGICASYHSMS----SFRSVNQRSIIAYTMSLGAENSV    699
MT040335.1      RAGCLVGAEHVNNSYECDIPVGAGICASYHSMS----SFRSVNQRSIIAYTMSLGAENSV    699
MN996532.2      RAGCLIGAEHVNNSYECDIPIGAGICASYQTQTNS----RSVASQSIIAYTMSLGAENSV    701
MN996529.1      RAGCLIGAEHVNNSYECDIPIGAGICASYQTQTNSPRRARSVASQSIIAYTMSLGAENSV    705
MN988713.1      RAGCLIGAEHVNNSYECDIPIGAGICASYQTQTNSPRRARSVASQSIIAYTMSLGAENSV    705
MT072688.1      RAGCLIGAEHVNNSYECDIPIGAGICASYQTQTNSPRRARSVASQSIIAYTMSLGAENSV    705
MN994467.1      RAGCLIGAEHVNNSYECDIPIGAGICASYQTQTNSPRRARSVASQSIIAYTMSLGAENSV    705
NC_045512.2     RAGCLIGAEHVNNSYECDIPIGAGICASYQTQTNSPRRARSVASQSIIAYTMSLGAENSV    705
MT093571.1      RAGCLIGAEHVNNSYECDIPIGAGICASYQTQTNSPRRARSVASQSIIAYTMSLGAENSV    705
MG772933.1      QAGCLIGAEHVNASYECDIPIGAGICASYHTAS----ILRSTSQKAIVAYTMSLGAENSI    678
MG772934.1      QAGCLIGAEHVNASYECDIPIGAGICASYHTAS----ILRSTGQKAIVAYTMSLGAENSI    677
KY417145.1      QAGCLIGAEHVNASYECDIPIGAGICASYHTAS----TLRGVGQKSIVAYTMSLGAENSI    666
KP886809.1      QAGCLIGAEHVNSSYECDIPIGAGICASYHTAS----VLRSTGQKSIVAYTMSLGAENSV    673
KY417143.1      QAGCLIGAEHVNASYECDIPIGAGICASYHTAS----TLRSVGQKSIVAYTMSLGAENSI    673
FJ588686.1      QAGCLIGAEHVNASYECDIPIGAGICASYHTAS----TLRSVGQKSIVAYTMSLGAENSI    673
KY417148.1      QAGCLIGAEHVNASYECDIPIGAGICASYHTAS----TLRSVGQKSIVAYTMSLGAENSI    674
KY417147.1      QAGCLIGAEHVNASYECDIPIGAGICASYHTAS----TLRSVGQKSIVAYTMSLGAENSI    673
KY417142.1      QAGCLIGAEHVNASYECDIPIGAGICASYHTAS----TLRSVGQKSIVAYTMSLGAENSI    673
GQ153542.1      QAGCLIGAEHVNASYECDIPIGAGICASYHTAS----VLRSTGQKSIVAYTMSLGAENSI    674
DQ022305        QAGCLIGAEHVNASYECDIPIGAGICASYHTAS----VLRSTGQKSIVAYTMSLGAENSI    674
                :****:** : : *******:******.* . :           ::*:******* :..:
```

NC_014470.1	VYSNNTIAIPMNFSISVTTEVLPVSMTKTSVDCNMYICGDSTECSNLLLQYGSFCTQLNR	751
KF569996.1	AYANNSIAIPTNFSISITTEVMPVSMAKTSVDCTMYICGDSQECSNLLLQYGSFCTQLNR	751
KY417144.1	AYSNNTIAIPTNFSISITTEVMPVSMAKTSVDCNMYICGDSTECANLLLQYGSFCTQLNR	748
KC881005.1	AYSNNTIAIPTNFSISITTEVMPVSMAKTSVDCNMYICGDSTECANLLLQYGSFCTQLNR	748
KC881006.1	AYSNNTIAIPTNFSISITTEVMPVSMAKTSVDCNMYICGDSTECANLLLQYGSFCTQLNR	748
KF367457.1	AYSNNTIAIPTNFSISITTEVMPVSMAKTSVDCNMYICGDSTECANLLLQYGSFCTQLNR	748
KY417152.1	VYSNNTIAIPTNFSISITTEVMPVSMAKTSVDCNMYICGDSTECANLLLQYGSFCTQLNR	748
KY417151.1	VYSNNTIAIPTNFSISITTEVMPVSMAKTSVDCNMYICGDSTECANLLLQYGSFCTQLNR	748
KY417146.1	AYSNNTIAIPTNFSISITTEVMPVSMAKTSVDCNMYICGDSTECANLLLQYGSFCTQLNR	747
KT444582.1	AYSNNTIAIPTNFSISITTEVMPVSMAKTSVDCNMYICGDSTECANLLLQYGSFCTQLNR	747
AY278488.2	AYSNNTIAIPTNFSISITTEVMPVSMAKTSVDCNMYICGDSTECANLLLQYGSFCTQLNR	747
AY274119	AYSNNTIAIPTNFSISITTEVMPVSMAKTSVDCNMYICGDSTECANLLLQYGSFCTQLNR	747
AP006560.1	AYSNNTIAIPTNFSISITTEVMPVSMAKTSVDCNMYICGDSTECANLLLQYGSFCTQLNR	747
AP006557.1	AYSNNTIAIPTNFSISITTEVMPVSMAKTSVDCNMYICGDSTECANLLLQYGSFCTQLNR	747
AY485277.1	AYSNNTIAIPTNFSISITTEVMPVSMAKTSVDCNMYICGDSTECANLLLQYGSFCTQLNR	747
AY572038.1	AYSNNTIAIPTNFSISITTEVMPVSMAKTSVDCNMYICGDSTECANLLLQYGSFCRQLNR	747
AY572034.1	AYSNNTIAIPTNFSISITTEVMPVSMAKTSVDCNMYICGDSTECANLLLQYGSFCRQLNR	747
MT040334.1	AYSNNSIAIPTNFTISVTTEILPVSMTKTSVDCTMYICGDSIECSNLLLQYGSFCTQLNR	757
MW532698.1	AYSNNSIAIPTNFTISVTTEILPVSMTKTSVDCTMYICGDSIECSNLLLQYGSFCTQLNR	761
MT040333.1	AYSNNSIAIPTNFTISVTTEILPVSMTKTSVDCTMYICGDSIECSNLLLQYGSFCTQLNR	759
MT040336.1	AYSNNSIAIPTNFTISVTTEILPVSMTKTSVDCTMYICGDSIECSNLLLQYGSFCTQLNR	759
MT040335.1	AYSNNSIAIPTNFTISVTTEILPVSMTKTSVDCTMYICGDSIECSNLLLQYGSFCTQLNR	759
MN996532.2	AYSNNSIAIPTNFTISVTTEILPVSMTKTSVDCTMYICGDSTECSNLLLQYGSFCTQLNR	761
MN996529.1	AYSNNSIAIPTNFTISVTTEILPVSMTKTSVDCTMYICGDSTECSNLLLQYGSFCTQLNR	765
MN988713.1	AYSNNSIAIPTNFTISVTTEILPVSMTKTSVDCTMYICGDSTECSNLLLQYGSFCTQLNR	765
MT072688.1	AYSNNSIAIPTNFTISVTTEILPVSMTKTSVDCTMYICGDSTECSNLLLQYGSFCTQLNR	765
MN994467.1	AYSNNSIAIPTNFTISVTTEILPVSMTKTSVDCTMYICGDSTECSNLLLQYGSFCTQLNR	765
**NC_045512.2**	**AYSNNSIAIPTNFTISVTTEILPVSMTKTSVDCTMYICGDSTECSNLLLQYGSFCTQLNR**	**765**
MT093571.1	AYSNNSIAIPTNFTISVTTEILPVSMTKTSVDCTMYICGDSTECSNLLLQYGSFCTQLNR	765
MG772933.1	AYANNSIAIPTNFSISVTTEVMPVSMAKTSVDCTMYICGDSIECSNLLLQYGSFCTQLNR	738
MG772934.1	AYANNSIAIPTNFSISVTTEVMPVSMAKTSVDCTMYICGDSIECSNLLLQYGSFCTQLNR	737
KY417145.1	AYANNSIAIPTNFSISVTTEVMPVSMAKTSVDCTMYICGDSQECSNLLLQYGSFCTQLNR	726
KP886809.1	AYANNSIAIPTNFSISVTTEVMPVSMAKTSVDCTMYICGDSAECSNLLLQYGSFCTQLNR	733
KY417143.1	AYANNSIAIPTNFSISVTTEVMPVSMSKTSVDCTMYICGDSQECSNLLLQYGSFCTQLNR	733
FJ588686.1	AYANNSIAIPTNFSISVTTEVMPVSMAKTSVDCTMYICGDSQECSNLLLQYGSFCTQLNR	733
KY417148.1	AYANNSIAIPTNFSISVTTEVMPVSMSKTSVDCTMYICGDSQECSNLLLQYGSFCTQLNR	734
KY417147.1	AYANNSIAIPTNFSISVTTEVMPVSMSKTSVDCTMYICGDSQECSNLLLQYGSFCTQLNR	733
KY417142.1	AYANNSIAIPTNFSISVTTEVMPVSMAKTSVDCTMYICGDSQECSNLLLQYGSFCTQLNR	733
GQ153542.1	AYANNSIAIPTNFSISVTIEVMPVSMAKTSVDCTMYICGDSLECSNLLLQYGSFCTQLNR	734
DQ022305	AYANNSIAIPTNFSISVTTEVMPVSMAKTAVDCTMYICGDSLECSNLLLQYGSFCTQLNR	734
	.*:**:**** **:**:* *::****:**:***.******* **:********** ****	

```
NC_014470.1       ALAGIAVEQDRNTRDVFAQTKAMYKTPSLKDFGGFNFSQILPDPAKPSSRSFIEDLLYNK     811
KF569996.1        ALSGIAVEQDKNTQEVFAQVKQIYKTPAIKDFGGFNFSQILPDPSKPTKRSFIEDLLFNK     811
KY417144.1        ALSGIAVEQDRNTREVFAQVKQMYKTPTLKDFGGFNFSQILPDPLKPTKRSFIEDLLFNK     808
KC881005.1        ALSGIAVEQDRNTREVFAQVKQMYKTPTLKDFGGFNFSQILPDPLKPTKRSFIEDLLFNK     808
KC881006.1        ALSGIAVEQDRNTREVFAQVKQMYKTPTLKDFGGFNFSQILPDPLKPTKRSFIEDLLFNK     808
KF367457.1        ALSGIAVEQDRNTREVFAQVKQMYKTPTLKDFGGFNFSQILPDPLKPTKRSFIEDLLFNK     808
KY417152.1        ALSGIAVEQDRNTREVFAQVKQMYKTPTLKDFGGFNFSQILPDPLKPTKRSFIEDLLFNK     808
KY417151.1        ALSGIAVEQDRNTREVFAQVKQMYKTPTLKDFGGFNFSQILPDPLKPTKRSFIEDLLFNK     808
KY417146.1        ALSGIAVEQDRNTREVFAQVKQMYKTPALKDFGGFNFSQILPDPLKPTKRSFIEDLLFNK     807
KT444582.1        ALSGIAVEQDRNTREVFAQVKQMYKTPTLKDFGGFNFSQILPDPLKPTKRSFIEDLLFNK     807
AY278488.2        ALSGIAAEQDRNTREVFAQVKQMYKTPTLKYFGGFNFSQILPDPLKPTKRSFIEDLLFNK     807
AY274119          ALSGIAAEQDRNTREVFAQVKQMYKTPTLKYFGGFNFSQILPDPLKPTKRSFIEDLLFNK     807
AP006560.1        ALSGIAAEQDRNTREVFAQVKQMYKTPTLKYFGGFNFSQILPDPLKPTKRSFIEDLLFNK     807
AP006557.1        ALSGIAAEQDRNTREVFAQVKQMYKTPTLKYFGGFNFSQILPDPLKPTKRSFIEDLLFNK     807
AY485277.1        ALSGIAAEQDRNTREVFAQVKQMYKTPTLKYFGGFNFSQILPDPLKPTKRSFIEDLLFNK     807
AY572038.1        ALSGIAAEQDRNTREVFVQVKQMYKTPTLKDFGGFNFSQILPDPLKPTKRSFIEDLLFNK     807
AY572034.1        ALSGIAAEQDRNTREVFVQVKQMYKTPTLKDFGGFNFSQILPDPLKPTKRSFIEDLLFNK     807
MT040334.1        ALTGIAVEQDKNTQEVFAQVKQIYKTPPIKDFGGFNFSQILPDPSKPSKRSFIEDLLFNK     817
MW532698.1        ALTGIAVEQDKNTQEVFAQVKQIYKTPPIKDFGGFNFLQILPDPSKPSKRSFIEDLLFNK     821
MT040333.1        ALTGIAVEQDKNTQEVFAQVKQIYKTPPIKDFGGFNFSQILPDPSKPSKRSFIEDLLFNK     819
MT040336.1        ALTGIAVEQDKNTQEVFAQVKQIYKTPPIKDFGGFNFSQILPDPSKPSKRSFIEDLLFNK     819
MT040335.1        ALTGIAVEQDKNTQEVFAQVKQIYKTPPIKDFGGFNFSQILPDPSKPSKRSFIEDLLFNK     819
MN996532.2        ALTGIAVEQDKNTQEVFAQVKQIYKTPPIKDFGGFNFSQILPDPSKPSKRSFIEDLLFNK     821
MN996529.1        ALTGIAVEQDKNTQEVFAQVKQIYKTPPIKDFGGFNFSQILPDPSKPSKRSFIEDLLFNK     825
MN988713.1        ALTGIAVEQDKNTQEVFAQVKQIYKTPPIKDFGGFNFSQILPDPSKPSKRSFIEDLLFNK     825
MT072688.1        ALTGIAVEQDKNTQEVFAQVKQIYKTPPIKDFGGFNFSQILPDPSKPSKRSFIEDLLFNK     825
MN994467.1        ALTGIAVEQDKNTQEVFAQVKQIYKTPPIKDFGGFNFSQILPDPSKPSKRSFIEDLLFNK     825
NC_045512.2       ALTGIAVEQDKNTQEVFAQVKQIYKTPPIKDFGGFNFSQILPDPSKPSKRSFIEDLLFNK     825
MT093571.1        ALTGIAVEQDKNTQEVFAQVKQIYKTPPIKDCGGFNFSQILPDPSKPSKRSFIEDLLFNK     825
MG772933.1        ALSGIAIEQDKNTQEVFAQVKQIYKTPPIKDFGGFNFSQILPDPSKPSKRSFIEDLLFNK     798
MG772934.1        ALSGIAIEQDKNTQEVFAQVKQIYKTPPIKDFGGFNFSQILPDPSKPSKRSFIEDLLFNK     797
KY417145.1        ALTGVALEQDKNTQDVFAQVKQMYKTPAIKDFGGFNFSQILPDPSKPTKRSFIEDLLFNK     786
KP886809.1        ALSGIAVEQDKNTQEVFAQVKQMYKTPTLRDFGGFNFSQILPDPLKPTKRSFIEDLLYNK     793
KY417143.1        ALTGIAIEQDKNTQEVFAQVKQMYKTPAIKDFGGFNFSQILPDPSKPTKRSFIEDLLFNK     793
FJ588686.1        ALTGVALEQDKNTQEVFAQVKQMYKTPAIKDFGGFNFSQILPDPSKPTKRSFIEDLLFNK     793
KY417148.1        ALTGIAIEQDKNTQEVFAQVKQMYKTPAIKDFGGFNFSQILPDPSKPTKRSFIEDLLFNK     794
KY417147.1        ALTGIAIEQDKNTQEVFAQVKQMYKTPAIKDFGGFNFSQILPDPSKPTKRSFIEDLLFNK     793
KY417142.1        ALTGVALEQDKNTQEVFAQVKQMYKTPAIKDFGGFNFSQILPDPSKPTKRSFIEDLLFNK     793
GQ153542.1        ALTGIAIEQDKNTQEVFAQVKQMYKTPAIKDFGGFNFSQILPDPSKPTKRSFIEDLLFNK     794
DQ022305          ALTGIAIEQDKNTQEVFAQVKQMYKTPAIKDFGGFNFSQILPDPSKPTKRSFIEDLLFNK     794
                  **:*:* ***:**::**.*.* :**** ::  ***** ****** **:.********:**
```

CoVAX_MNS#3

```
NC_014470.1      VTLADPGFMKQYGDCLGGVNARDLICAQKFNGLTVLPPLLTDEMIAAYTAALISGTATAG      871
KF569996.1       VTLADAGFMKQYGECLGDISARDLICAQKFNGLTVLPPLLTDEMIAAYTAALVSGTATAG      871
KY417144.1       VTLADAGFMKQYGECLGDINARDLICAQKFNGLTVLPPLLTDDMIAAYTAALVSGTATAG      868
KC881005.1       VTLADAGFMKQYGECLGDINARDLICAQKFNGLTVLPPLLTDDMIAAYTAALVSGTATAG      868
KC881006.1       VTLADAGFMKQYGECLGDINARDLICAQKFNGLTVLPPLLTDDMIAAYTAALVSGTATAG      868
KF367457.1       VTLADAGFMKQYGECLGDINARDLICAQKFNGLTVLPPLLTDDMIAAYTAALVSGTATAG      868
KY417152.1       VTLADAGFMKQYGECLGDINARDLICAQKFNGLTVLPPLLTDDMIAAYTAALVSGTATAG      868
KY417151.1       VTLADAGFMKQYGECLGDINARDLICAQKFNGLTVLPPLLTDDMIAAYTAALVSGTATAG      868
KY417146.1       VTLADAGFMKQYGECLGDVNARDLICAQKFNGLTVLPPLLTDDMIAAYTAALVSGTATAG      867
KT444582.1       VTLADAGFMKQYGECLGDINARDLICAQKFNGLTVLPPLLTDDMIAAYTAALVSGTATAG      867
AY278488.2       VTLADAGFMKQYGECLGDINARDLICAQKFNGLTVLPPLLTDDMIAAYTAALVSGTATAG      867
AY274119         VTLADAGFMKQYGECLGDINARDLICAQKFNGLTVLPPLLTDDMIAAYTAALVSGTATAG      867
AP006560.1       VTLADAGFMKQYGECLGDINARDLICAQKFNGLTVLPPLLTDDMIAAYTAALVSGTATAG      867
AP006557.1       VTLADAGFMKQYGECLGDINARDLICAQKFNGLTVLPPLLTDDMIAAYTAALVSGTATAG      867
AY485277.1       VTLADAGFMKQYGECLGDINARDLICAQKFNGLTVLPPLLTDDMIAAYTAALVSGTATAG      867
AY572038.1       VTLADAGFMKQYGECLGDINARDLICAQKFNGLTVLPPLLTDDMIAAYTAALVSGTATAG      867
AY572034.1       VTLADAGFMKQYGECLGDINARDLICAQKFNGLTVLPPLLTDDMIAAYTAALVSGTATAG      867
MT040334.1       VTLADAGFIKQYGDCLGDIAARDLICAQKFNGLTVLPPLLTDEMIAQYTSALLAGTITSG      877
MW532698.1       VTLADAGFIKQYGDCLGDIAARDLICAQKFNGLTVLPPLLTDEMIAQYTSALLAGTITSG      881
MT040333.1       VTLADAGFIKQYGDCLGDIAARDLICAQKFNGLTVLPPLLTDEMIAQYTSALLAGTITSG      879
MT040336.1       VTLADAGFIKQYGDCLGDIAARDLICAQKFNGLTVLPPLLTDEMIAQYTSALLAGTITSG      879
MT040335.1       VTLADAGFIKQYGDCLGDIAARDLICAQKFNGLTVLPPLLTDEMIAQYTSALLAGTITSG      879
MN996532.2       VTLADAGFIKQYGDCLGDIAARDLICAQKFNGLTVLPPLLTDEMIAQYTSALLAGTITSG      881
MN996529.1       VTLADAGFIKQYGDCLGDIAARDLICAQKFNGLTVLPPLLTDEMIAQYTSALLAGTITSG      885
MN988713.1       VTLADAGFIKQYGDCLGDIAARDLICAQKFNGLTVLPPLLTDEMIAQYTSALLAGTITSG      885
MT072688.1       VTLADAGFIKQYGDCLGDIAARDLICAQKFNGLTVLPPLLTDEMIAQYTSALLAGTITSG      885
MN994467.1       VTLADAGFIKQYGDCLGDIAARDLICAQKFNGLTVLPPLLTDEMIAQYTSALLAGTITSG      885
NC_045512.2      VTLADAGFIKQYGDCLGDIAARDLICAQKFNGLTVLPPLLTDEMIAQYTSALLAGTITSG      885
MT093571.1       VTLADAGFIKQYGDCLGDIAARDLICAQKFNGLTVLPPLLTDEMIAQYTSALLAGTITSG      885
MG772933.1       VTLADAGFIKQYGDCLGGISARDLICAQKFNGLTVLPPLLTDEMIAAYTAALISGTATAG      858
MG772934.1       VTLADAGFIKQYGDCLGDISARDLICAQKFNGLTVLPPLLTDEMIAAYTAALISGTATAG      857
KY417145.1       VTLADAGFMKQYGECLGDISARDLICAQKFNGLTVLPPLLTDEMIAAYTAALVSGTATAG      846
KP886809.1       VTLADAGFMKQYADCLGGINARDLICAQKFNGLTVLPPLLTDDMIAAYTAALISGTATAG      853
KY417143.1       VTLADAGFMKQYGECLGDINARDLICAQKFNGLTVLPPLLTDDMIAAYTAALVSGTATAG      853
FJ588686.1       VTLADAGFMKQYGECLGDISARDLICAQKFNGLTVLPPLLTDEMIAAYTAALVSGTATAG      853
KY417148.1       VTLADAGFMKQYGECLGDINARDLICAQKFNGLTVLPPLLTDDMIAAYTAALVSGTATAG      854
KY417147.1       VTLADAGFMKQYGECLGDINARDLICAQKFNGLTVLPPLLTDDMIAAYTAALVSGTATAG      853
KY417142.1       VTLADAGFMKQYGECLGDINARDLICAQKFNGLTVLPPLLTDDMIAAYTAALVSGTATAG      853
GQ153542.1       VTLADAGFMKQYGECLGDVSARDLICAQKFNGLTVLPPLLTDEMIAAYTAALVSGTATAG      854
DQ022305         VTLADAGFMKQYGDCLGDVSARDLICAQKFNGLTVLPPLLTDEMVAAYTAALVSGTATAG      854
                 ***** **:***.:***.: **********************:*:* **:**::** *:*
```

CoVAX_MNS#3   CoVAX_MNS#2

```
NC_014470.1       FTFGAGAALQIPFAMQMAYRFNGIGVTQNVLYENQKQIANQFNKAISQIQDSLSTTTTAL       931
KF569996.1        WTFGAGAALQIPFAMQMAYRFNGIGVTQNVLYENQKQIANQFNKAISQIQESLTTTSTAL       931
KY417144.1        WTFGAGAALQIPFAMQMAYRFNGIGVTQNVLYENQKQIANQFNKAISQIQESLTTTSTAL       928
KC881005.1        WTFGAGAALQIPFAMQMAYRFNGIGVTQNVLYENQKQIANQFNKAISQIQESLTTTSTAL       928
KC881006.1        WTFGAGAALQIPFAMQMAYRFNGIGVTQNVLYENQKQIANQFNKAISQIQESLTTTSTAL       928
KF367457.1        WTFGAGAALQIPFAMQMAYRFNGIGVTQNVLYENQKQIANQFNKAISQIQESLTTTSTAL       928
KY417152.1        WTFGAGAALQIPFAMQMAYRFNGIGVTQNVLYENQKQIANQFNKAISQIQESLTTTSTAL       928
KY417151.1        WTFGAGAALQIPFAMQMAYRFNGIGVTQNVLYENQKQIANQFNKAISQIQESLTTTSTAL       928
KY417146.1        WTFGAGAALQIPFAMQMAYRFNGIGVTQNVLYENQKQIANQFNKAISQIQESLTTTSTAL       927
KT444582.1        WTFGAGAALQIPFAMQMAYRFNGIGVTQNVLYENQKQIANQFNKAISQIQESLTTTSTAL       927
AY278488.2        WTFGAGAALQIPFAMQMAYRFNGIGVTQNVLYENQKQIANQFNKAISQIQESLTTTSTAL       927
AY274119          WTFGAGAALQIPFAMQMAYRFNGIGVTQNVLYENQKQIANQFNKAISQIQESLTTTSTAL       927
AP006560.1        WTFGAGAALQIPFAMQMAYRFNGIGVTQNVLYENQKQIANQFNKAISQIQESLTTTSTAL       927
AP006557.1        WTFGAGAALQIPFAMQMAYRFNGIGVTQNVLYENQKQIANQFNKAISQIQESLTTTSTAL       927
AY485277.1        WTFGAGAALQIPFAMQMAYRFNGIGVTQNVLYENQKQIANQFNKAISQIQESLTTTSTAL       927
AY572038.1        WTFGAGAALQIPFAMQMAYRFNGIGVTQNVLYENQKQIANQFNKAISQIQESLTTTSTAL       927
AY572034.1        WTFGAGAALQIPFAMQMAYRFNGIGVTQNVLYENQKQIANQFNKAISQIQESLTTTSTAL       927
MT040334.1        WTFGAGAALQIPFAMQMAYRFNGIGVTQNVLYENQKLIANQFNSAIGKIQDSLSSTASAL       937
MW532698.1        WTFGAGAALQIPFAMQMAYRFNGIGVTQNVLYENQKLIANQFNSAIGKIQDSLSSTASAL       941
MT040333.1        WTFGAGAALQIPFAMQMAYRFNGIGVTQNVLYENQKLIANQFNSAIGKIQDSLSSTASAL       939
MT040336.1        WTFGAGAALQIPFAMQMAYRFNGIGVTQNVLYENQKLIANQFNSAIGKIQDSLSSTASAL       939
MT040335.1        WTFGAGAALQIPFAMQMAYRFNGIGVTQNVLYENQKLIANQFNSAIGKIQDSLSSTASAL       939
MN996532.2        WTFGAGAALQIPFAMQMAYRFNGIGVTQNVLYENQKLIANQFNSAIGKIQDSLSSTASAL       941
MN996529.1        WTFGAGAALQIPFAMQMAYRFNGIGVTQNVLYENQKLIANQFNSAIGKIQDSLSSTASAL       945
MN988713.1        WTFGAGAALQIPFAMQMAYRFNGIGVTQNVLYENQKLIANQFNSAIGKIQDSLSSTASAL       945
MT072688.1        WTFGAGAALQIPFAMQMAYRFNGIGVTQNVLYENQKLIANQFNSAIGKIQDSLSSTASAL       945
MN994467.1        WTFGAGAALQIPFAMQMAYRFNGIGVTQNVLYENQKLIANQFNSAIGKIQDSLSSTASAL       945
NC_045512.2       WTFGAGAALQIPFAMQMAYRFNGIGVTQNVLYENQKLIANQFNSAIGKIQDSLSSTASAL       945
MT093571.1        WTFGAGAALQIPFAMQMAYRFNGIGVTQNVLYENQKLIANQFNSAIGKIQDSLSSTASAL       945
MG772933.1        WTFGAGAALQIPFAMQMAYRFNGIGVTQNVLYENQKLIANQFNSAIGKIQESLTSTASAL       918
MG772934.1        WTFGAGAALQIPFAMQMAYRFNGIGVTQNVLYENQKLIANQFNSAIGKIQESLTSTASAL       917
KY417145.1        WTFGAGAALQIPFAMQMAYRFNGIGVTQNVLYENQKQIANQFNKAISQIQESLTTTSTAL       906
KP886809.1        WTFGAGAALQIPFAMQMAYRFNGIGVTQNVLYENQKQIANQFNKAITQIQESLTTTSTAL       913
KY417143.1        WTFGAGAALQIPFAMQMAYRFNGIGVTQNVLYENQKQIANQFNKAISQIQESLTTTSTAL       913
FJ588686.1        WTFGAGAALQIPFAMQMAYRFNGIGVTQNVLYENQKQIANQFNKAISQIQESLTTTSTAL       913
KY417148.1        WTFGAGAALQIPFAMQMAYRFNGIGVTQNVLYENQKQIANQFNKAISQIQESLTTTSTAL       914
KY417147.1        WTFGAGAALQIPFAMQMAYRFNGIGVTQNVLYENQKQIANQFNKAISQIQESLTTTSTAL       913
KY417142.1        WTFGAGAALQIPFAMQMAYRFNGIGVTQNVLYENQKQIANQFNKAISQIQESLTTTSTAL       913
GQ153542.1        WTFGAGAALQIPFAMQMAYRFNGIGVTQNVLYENQKLIANQFNSAIGKIQDSLSSTASAL       914
DQ022305          WTFGAGAALQIPFAMQMAYRFNGIGVTQNVLYENQKLIANQFNSAIGKIQESLSSTASAL       914
                  :*********************************** ******.** :**:**::*::**
```

CoVAX_MNS#10   CoVAX_MNS#8

```
NC_014470.1     GKLQDVINQNAIALNTLVKQLSSNFGAISSVLNDILSRLDKVEAEVQIDRLITGRLQSLQ   991
KF569996.1      GKLQDVVNQNAQALNTLVKQLSSNFGAISSVLNDILSRLDKVEAEVQIDRLITGRLQSLQ   991
KY417144.1      GKLQDVVNQNAQALNTLVKQLSSNFGAISSVLNDILSRLDKVEAEVQIDRLITGRLQSLQ   988
KC881005.1      GKLQDVVNQNAQALNTLVKQLSSNFGAISSVLNDILSRLDKVEAEVQIDRLITGRLQSLQ   988
KC881006.1      GKLQDVVNQNAQALNTLVKQLSSNFGAISSVLNDILSRLDKVEAEVQIDRLITGRLQSLQ   988
KF367457.1      GKLQDVVNQNAQALNTLVKQLSSNFGAISSVLNDILSRLDKVEAEVQIDRLITGRLQSLQ   988
KY417152.1      GKLQDVVNQNAQALNTLVKQLSSNFGAISSVLNDILSRLDKVEAEVQIDRLITGRLQSLQ   988
KY417151.1      GKLQDVVNQNAQALNTLVKQLSSNFGAISSVLNDILSRLDKVEAEVQIDRLITGRLQSLQ   988
KY417146.1      GKLQDVVNQNAQALNTLVKQLSSNFGAISSVLNDILSRLDKVEAEVQIDRLITGRLQSLQ   987
KT444582.1      GKLQDVVNQNAQALNTLVKQLSSNFGAISSVLNDILSRLDKVEAEVQIDRLITGRLQSLQ   987
AY278488.2      GKLQDVVNQNAQALNTLVKQLSSNFGAISSVLNDILSRLDKVEAEVQIDRLITGRLQSLQ   987
AY274119        GKLQDVVNQNAQALNTLVKQLSSNFGAISSVLNDILSRLDKVEAEVQIDRLITGRLQSLQ   987
AP006560.1      GKLQDVVNQNAQALNTLVKQLSSNFGAISSVLNDILSRLDKVEAEVQIDRLITGRLQSLQ   987
AP006557.1      GKLQDVVNQNAQALNTLVKQLSSNFGAISSVLNDILSRLDKVEAEVQIDRLITGRLQSLQ   987
AY485277.1      GKLQDVVNQNAQALNTLVKQLSSNFGAISSVLNDILSRLDKVEAEVQIDRLITGRLQSLQ   987
AY572038.1      GKLQDVVNQNAQALNTLVKQLSSNFGAISSVLNDILSRLDKVEAEVQIDRLITGRLQSLQ   987
AY572034.1      GKLQDVVNQNAQALNTLVKQLSSNFGAISSVLNDILSRLDKVEAEVQIDRLITGRLQSLQ   987
MT040334.1      GKLQDVVNQNAQALNTLVKQLSSNFGAISSVLNDILSRLDKVEAEVQIDRLITGRLQSLQ   997
MW532698.1      GKLQDVVNQNAQALNTLVKQLSSNFGAISSVLNDILSRLDKVEAEVQIDRLITGRLQSLQ   1001
MT040333.1      GKLQDVVNQNAQALNTLVKQLSSNFGAISSVLNDILSRLDKVEAEVQIDRLITGRLQSLQ   999
MT040336.1      GKLQDVVNQNAQALNTLVKQLSSNFGAISSVLNDILSRLDKVEAEVQIDRLITGRLQSLQ   999
MT040335.1      GKLQDVVNQNAQALNTLVKQLSSNFGAISSVLNDILSRLDKVEAEVQIDRLITGRLQSLQ   999
MN996532.2      GKLQDVVNQNAQALNTLVKQLSSNFGAISSVLNDILSRLDKVEAEVQIDRLITGRLQSLQ   1001
MN996529.1      GKLQDVVNQNAQALNTLVKQLSSNFGAISSVLNDILSRLDKVEAEVQIDRLITGRLQSLQ   1005
MN988713.1      GKLQDVVNQNAQALNTLVKQLSSNFGAISSVLNDILSRLDKVEAEVQIDRLITGRLQSLQ   1005
MT072688.1      GKLQDVVNQNAQALNTLVKQLSSNFGAISSVLNDILSRLDKVEAEVQIDRLITGRLQSLQ   1005
MN994467.1      GKLQDVVNQNAQALNTLVKQLSSNFGAISSVLNDILSRLDKVEAEVQIDRLITGRLQSLQ   1005
NC_045512.2     GKLQDVVNQNAQALNTLVKQLSSNFGAISSVLNDILSRLDKVEAEVQIDRLITGRLQSLQ   1005
MT093571.1      GKLQDVVNQNAQALNTLVKQLSSNFGAISSVLNDILSRLDKVEAEVQIDRLITGRLQSLQ   1005
MG772933.1      GKLQDVVNQNAQALNTLVKQLSSNFGAISSVLNDILSRLDKVEAEVQIDRLITGRLQSLQ   978
MG772934.1      GKLQDVVNQNAQALNTLVKQLSSNFGAISSVLNDILSRLDKVEAEVQIDRLITGRLQSLQ   977
KY417145.1      GKLQDVVNQNAQALNTLVKQLSSNFGAISSVLNDILSRLDKVEAEVQIDRLITGRLQSLQ   966
KP886809.1      GKLQDVVNQNAQALNTLVKQLSSNFGAISSVLNDILSRLDKVEAEVQIDRLITGRLQSLQ   973
KY417143.1      GKLQDVVNQNAQALNTLVKQLSSNFGAISSVLNDILSRLDKVEAEVQIDRLITGRLQSLQ   973
FJ588686.1      GKLQDVVNQNAQALNTLVKQLSSNFGAISSVLNDILSRLDKVEAEVQIDRLITGRLQSLQ   973
KY417148.1      GKLQDVVNQNAQALNTLVKQLSSNFGAISSVLNDILSRLDKVEAEVQIDRLITGRLQSLQ   974
KY417147.1      GKLQDVVNQNAQALNTLVKQLSSNFGAISSVLNDILSRLDKVEAEVQIDRLITGRLQSLQ   973
KY417142.1      GKLQDVVNQNAQALNTLVKQLSSNFGAISSVLNDILSRLDKVEAEVQIDRLITGRLQSLQ   973
GQ153542.1      GKLQDVVNQNAQALNTLVKQLSSNFGAISSVLNDILSRLDKVEAEVQIDRLITGRLQSLQ   974
DQ022305        GKLQDVVNQNAQALNTLVKQLSSNFGAISSVLNDILSRLDKVEAEVQIDRLITGRLQSLQ   974
                ******:**** ************************************************
```

CoVAX_MNS#8

CoVAX_MNS#4

```
NC_014470.1     TYVTQQLIRAAEIRASANLAATKMSECVLGQSKRVDFCGKGYHLMSFPQAAPHGVVFLHV     1051
KF569996.1      TYVTQQLIRAAEIRASANLAATKMSECVLGQSKRVDFCGKGYHLMSFPQAAPHGVVFLHV     1051
KY417144.1      TYVTQQLIRAAEIRASANLAATKMSECVLGQSKRVDFCGKGYHLMSFPQAAPHGVVFLHV     1048
KC881005.1      TYVTQQLIRAAEIRASANLAATKMSECVLGQSKRVDFCGKGYHLMSFPQAAPHGVVFLHV     1048
KC881006.1      TYVTQQLIRAAEIRASANLAATKMSECVLGQSKRVDFCGKGYHLMSFPQAAPHGVVFLHV     1048
KF367457.1      TYVTQQLIRAAEIRASANLAATKMSECVLGQSKRVDFCGKGYHLMSFPQAAPHGVVFLHV     1048
KY417152.1      TYVTQQLIRAAEIRASANLAATKMSECVLGQSKRVDFCGKGYHLMSFPQAAPHGVVFLHV     1048
KY417151.1      TYVTQQLIRAAEIRASANLAATKMSECVLGQSKRVDFCGKGYHLMSFPQAAPHGVVFLHV     1048
KY417146.1      TYVTQQLIRAAEIRASANLAATKMSECVLGQSKRVDFCGKGYHLMSFPQAAPHGVVFLHV     1047
KT444582.1      TYVTQQLIRAAEIRASANLAATKMSECVLGQSKRVDFCGKGYHLMSFPQAAPHGVVFLHV     1047
AY278488.2      TYVTQQLIRAAEIRASANLAATKMSECVLGQSKRVDFCGKGYHLMSFPQAAPHGVVFLHV     1047
AY274119        TYVTQQLIRAAEIRASANLAATKMSECVLGQSKRVDFCGKGYHLMSFPQAAPHGVVFLHV     1047
AP006560.1      TYVTQQLIRAAEIRASANLAATKMSECVLGQSKRVDFCGKGYHLMSFPQAAPHGVVFLHV     1047
AP006557.1      TYVTQQLIRAAEIRASANLAATKMSECVLGQSKRVDFCGKGYHLMSFPQAAPHGVVFLHV     1047
AY485277.1      TYVTQQLIRAAEIRASANLAATKMSECVLGQSKRVDFCGKGYHLMSFPQAAPHGVVFLHV     1047
AY572038.1      TYVTQQLIRAAEIRASANLAATKMSECVLGQSKRVDFCGKGYHLMSFPQAAPHGVVFLHV     1047
AY572034.1      TYVTQQLIRAAEIRASANLAATKMSECVLGQSKRVDFCGKGYHLMSFPQAAPHGVVFLHV     1047
MT040334.1      TYVTQQLIRAAEIRASANLAATKMSECVLGQSKRVDFCGKGYHLMSFPQSAPHGVVFLHV     1057
MW532698.1      TYVTQQLIRAAEIRASANLAATKMSECVLGQSKRVDFCGKGYHLMSFPQSAPHGVVFLHV     1061
MT040333.1      TYVTQQLIRAAEIRASANLAATKMSECVLGQSKRVDFCGKGYHLMSFPQSAPHGVVFLHV     1059
MT040336.1      TYVTQQLIRAAEIRASANLAATKMSECVLGQSKRVDFCGKGYHLMSFPQSAPHGVVFLHV     1059
MT040335.1      TYVTQQLIRAAEIRASANLAATKMSECVLGQSKRVDFCGKGYHLMSFPQSAPHGVVFLHV     1059
MN996532.2      TYVTQQLIRAAEIRASANLAATKMSECVLGQSKRVDFCGKGYHLMSFPQSAPHGVVFLHV     1061
MN996529.1      TYVTQQLIRAAEIRASANLAATKMSECVLGQSKRVDFCGKGYHLMSFPQSAPHGVVFLHV     1065
MN988713.1      TYVTQQLIRAAEIRASANLAATKMSECVLGQSKRVDFCGKGYHLMSFPQSAPHGVVFLHV     1065
MT072688.1      TYVTQQLIRAAEIRASANLAATKMSECVLGQSKRVDFCGKGYHLMSFPQSAPHGVVFLHV     1065
MN994467.1      TYVTQQLIRAAEIRASANLAATKMSECVLGQSKRVDFCGKGYHLMSFPQSAPHGVVFLHV     1065
NC_045512.2     TYVTQQLIRAAEIRASANLAATKMSECVLGQSKRVDFCGKGYHLMSFPQSAPHGVVFLHV     1065
MT093571.1      TYVTQQLIRAAEIRASANLAATKMSECVLGQSKRVDFCGKGYHLMSFPQSAPHGVVFLHV     1065
MG772933.1      TYVTQQLIRAAEIRASANLAATKMSECVLGQSKRVDFCGKGYHLMSFPQSAPHGVVFLHV     1038
MG772934.1      TYVTQQLIRAAEIRASANLAATKMSECVLGQSKRVDFCGKGYHLMSFPQSAPHGVVFLHV     1037
KY417145.1      TYVTQQLIRAAEIRASANLAATKMSECVLGQSKRVDFCGRGYHLMSFPQAAPHGVVFLHV     1026
KP886809.1      TYVTQQLIRAAEIRASANLAATKMSECVLGQSKRVDFCGKGYHLMSFPQAAPHGVVFLHV     1033
KY417143.1      TYVTQQLIRAAEIRASANLAATKMSECVLGQSKRVDFCGRGYHLMSFPQAAPHGVVFLHV     1033
FJ588686.1      TYVTQQLIRAAEIRASANLAATKMSECVPGQSKRVDFCGRGYHLMSFPQAAPHGVVFLHV     1033
KY417148.1      TYVTQQLIRAAEIRASANLAATKMSECVLGQSKRVDFCGRGYHLMSFPQAAPHGVVFLHV     1034
KY417147.1      TYVTQQLIRAAEIRASANLAATKMSECVLGQSKRVDFCGRGYHLMSFPQAAPHGVVFLHV     1033
KY417142.1      TYVTQQLIRAAEIRASANLAATKMSECVLGQSKRVDFCGRGYHLMSFPQAAPHGVVFLHV     1033
GQ153542.1      TYVTQQLIRAAEIRASANLAATKMSECVLGQSKRVDFCGKGYHLMSFPQSAPHGVVFLHV     1034
DQ022305        TYVTQQLIRAAEIRASANLAATKMSECVLGQSKRVDFCGKGYHLMSFPQSAPHGVVFLHV     1034
                **************************** **********:*********:**********
```

CoVAX_MNS#4

CoVAX_MNS#17

NC_014470.1	TYVPSQEQNFTTAPAICHEGKAHFPREGVFVTNGTHWFITQRNFYSPQPITTDNTFVSGN	1111
KF569996.1	TYVPSQERNFTTAPAICHEGKAYFPREGVFVSNGTSWFITQRNFFSPQLITTDNTFVSGN	1111
KY417144.1	TYVPSQERNFTTAPAICHEGKAYFPREGVFVFNGTSWFITQRNFFSPQIITTDNTFVSGS	1108
KC881005.1	TYVPSQERNFTTAPAICHEGKAYFPREGVFVFNGTSWFITQRNFFSPQIITTDNTFVSGS	1108
KC881006.1	TYVPSQERNFTTAPAICHEGKAYFPREGVFVFNGTSWFITQRNFFSPQIITTDNTFVSGS	1108
KF367457.1	TYVPSQERNFTTAPAICHEGKAYFPREGVFVFNGTSWFITQRNFFSPQIITTDNTFVSGS	1108
KY417152.1	TYVPSQERNFTTAPAICHEGKAYFPREGVFVFNGTSWFITQRNFFSPQIITTDNTFVSGN	1108
KY417151.1	TYVPSQERNFTTAPAICHEGKAYFPREGVFVFNGTSWFITQRNFFSPQIITTDNTFVSGN	1108
KY417146.1	TCVPSQERNFTTAPAICHEGKAYFPREGVFVFNGTSWFITQRNFFSPQIITTDNTFVSGS	1107
KT444582.1	TYVPSQERNFTTAPAICHEGKAYFPREGVFVFNGTSWFITQRNFFSPQIITTDNTFVSGS	1107
AY278488.2	TYVPSQERNFTTAPAICHEGKAYFPREGVFVFNGTSWFITQRNFFSPQIITTDNTFVSGN	1107
AY274119	TYVPSQERNFTTAPAICHEGKAYFPREGVFVFNGTSWFITQRNFFSPQIITTDNTFVSGN	1107
AP006560.1	TYVPSQERNFTTAPAICHEGKAYFPREGVFVFNGTSWFITQRNFFSPQIITTDNTFVSGN	1107
AP006557.1	TYVPSQERNFTTAPAICHEGKAYFPREGVFVFNGTSWFITQRNFFSPQIITTDNTFVSGN	1107
AY485277.1	TYVPSQERNFTTAPAICHEGKAYFPREGVFVFNGTSWFITQRNFFSPQIITTDNTFVSGN	1107
AY572038.1	TYVPSQERNFTTAPAICHEGKAYFPREGVFVFNGTSWFITQRNFFSPQIITTDNTFVSGN	1107
AY572034.1	TYVPSQERNFTTAPAICHEGKAYFPREGVFVFSGTSWFITQRNFFSPQIITTDNTFVSGN	1107
MT040334.1	TYVPAQEKNFTTAPAICHEGKAHFPREGVFVSNGTHWFITQRNFYEPQIITTDNTFVSGS	1117
MW532698.1	TYVPAQEKNFTTAPAICHEGKAHFPREGVFVSNGTHWFITQRNFYEPQIITTDNTFVSGS	1121
MT040333.1	TYVPAQEKNFTTAPAICHEGKAHFPREGVFVSNGTHWFITQRNFYEPQIITTDNTFVSGS	1119
MT040336.1	TYVPAQEKNFTTAPAICHEGKAHFPREGVFVSNGTHWFITQRNFYEPQIITTDNTFVSGS	1119
MT040335.1	TYVPAQEKNFTTAPAICHEGKAHFPREGVFVSNGTHWFITQRNFYEPQIITTDNTFVSGS	1119
MN996532.2	TYVPAQEKNFTTAPAICHDGKAHFPREGVFVSNGTHWFVTQRNFYEPQIITTDNTFVSGS	1121
MN996529.1	TYVPAQEKNFTTAPAICHDGKAHFPREGVFVSNGTHWFVTQRNFYEPQIITTDNTFVSGN	1125
MN988713.1	TYVPAQEKNFTTAPAICHDGKAHFPREGVFVSNGTHWFVTQRNFYEPQIITTDNTFVSGN	1125
MT072688.1	TYVPAQEKNFTTAPAICHDGKAHFPREGVFVSNGTHWFVTQRNFYEPQIITTDNTFVSGN	1125
MN994467.1	TYVPAQEKNFTTAPAICHDGKAHFPREGVFVSNGTHWFVTQRNFYEPQIITTDNTFVSGN	1125
**NC_045512.2**	**TYVPAQEKNFTTAPAICHDGKAHFPREGVFVSNGTHWFVTQRNFYEPQIITTDNTFVSGN**	**1125**
MT093571.1	TYVPAQEKNFTTAPAICHDGKAHFPREGVFVSNGTHWFVTQRNFYEPQIITTDNTFVSGN	1125
MG772933.1	TYIPSQEKNFTTAPAICHEGKAHFPREGVFVSNGTHWFVTQRNFYEPKIITTDNTFVSGN	1098
MG772934.1	TYIPSQEKNFTTAPAICHEGKAHFPREGVFVSNGTHWFVTQRNFYEPQIITTDNTFVSGN	1097
KY417145.1	TYVPSHEKNFTTAPAICHEGKAYFPREGVFVSNGTSWFITQRNFYSPQIITTDNTFVAGN	1086
KP886809.1	TYVPSQEKNFTTAPAICHEGKAYFPREGVFVFNGTSWFITQRNFYSPQTITTDNTFVAGN	1093
KY417143.1	TYVPSQEKNFTTAPAICHEGKAYFPREGVFVSNGTSWFITQRNFYSPQIITTDNTFVAGN	1093
FJ588686.1	TYVPSQEKNFTTAPAICHEGKAYFPREGVFVSNGTSWFITQRNFYSPQIITTDNTFVAGN	1093
KY417148.1	TYVPSQEKNFTTAPAICHEGKAYFPREGVFVSNGTSWFITQRNFYSPQIITTDNTFVAGN	1094
KY417147.1	TYVPSQEKNFTTAPAICHEGKAYFPREGVFVSNGTSWFITQRNFYSPQIITTDNTFVAGN	1093
KY417142.1	TYVPSQEKNFTTAPAICHEGKAYFPREGVFVSNGTSWFITQRNFYSPQIITTDNTFVAGN	1093
GQ153542.1	TYVPSQERNFTTAPAICHEGKAYFPREGVFVSNGTSWFITQRNFYSPQLITTDNTFVSGN	1094
DQ022305	TYVPSQEKNFTTAPAICHEGKAYFPREGVFVSNGTSWFITQRNFYSPQLITTDNTFVSGN	1094
	* :*::*:*********:***:******** .** **:*****:.*: *******:*.	

```
NC_014470.1      CDVVIGIVNNTVYDPLQPELDSFKEELDKYFKNHTSQNVSLDGLNNINASVVDIKKEIEH      1171
KF569996.1       CDVVIGIINNTVYDPLQPELDSFKEELDKYFKNHTSPDVDLGDISRINASVVNIQKEIDR      1171
KY417144.1       CDVVIGIINNTVYDPLQPELDSFKEELDKYFKNHTSPDVDLGDISGINASVVNIQKEIDR      1168
KC881005.1       CDVVIGIINNTVYDPLQPELDSFKEELDKYFKNHTSPDVDLGDISGINASVVNIQKEIDR      1168
KC881006.1       CDVVIGIINNTVYDPLQPELDSFKEELDKYFKNHTSPDVDLGDISGINASVVNIQKEINR      1168
KF367457.1       CDVVIGIINNTVYDPLQPELDSFKEELDKYFKNHTSPDVDLGDISGINASVVNIQKEIDR      1168
KY417152.1       CDVVIGIINNTVYDPLQPELDSFKEELDKYFKNHTSPDVDLGDISGINASVVNIQKEIDR      1168
KY417151.1       CDVVIGIINNTVYDPLQPELDSFKEELDKYFKNHTSPDVDLGDISGINASVVNIQKEIDR      1168
KY417146.1       CDVVIGIINNTVYDPLQPELDSFKEELDKYFKNHTSPDVDLGDISGINASVVNIQKEIDR      1167
KT444582.1       CDVVIGIINNTVYDPLQPELDSFKEELDKYFKNHTSPDVDLGDISGINASVVNIQKEIDR      1167
AY278488.2       CDVVIGIINNTVYDPLQPELDSFKEELDKYFKNHTSPDVDLGDISGINASVVNIQKEIDR      1167
AY274119         CDVVIGIINNTVYDPLQPELDSFKEELDKYFKNHTSPDVDLGDISGINASVVNIQKEIDR      1167
AP006560.1       CDVVIGIINNTVYDPLQPELDSFKEELDKYFKNHTSPDVDLGDISGINASVVNIQKEIDR      1167
AP006557.1       CDVVIGIINNTVYDPLQPELDSFKEELDKYFKNHTSPDVDLGDISGINASVVNIQKEIDR      1167
AY485277.1       CDVVIGIINNTVYDPLQPELDSFKEELDKYFKNHTSPDVDLGDISGINASVVNIQKEIDR      1167
AY572038.1       CDVVIGIINNTVYDPLQPELDSFKEELDKYFKNHTSPDVDLGDISGINASVVNIQEEIDR      1167
AY572034.1       CDVVIGIINNTVYDPLQPELDSFKEELDKYFKNHTSPDVDLGDISGINASVVNIQEEIDR      1167
MT040334.1       CDVVIGIVNNTVYDPLQPELDSFKEELDKYFKNHTSPDVDLGDISGINASVVNIQKEIDR      1177
MW532698.1       CDVVIGIVNNTVYDPLQPELDSFKEELDKYFKNHTSPDVDLGDISGINASVVNIQKEIDR      1181
MT040333.1       CDVVIGIVNNTVYDPLQPELDSFKEELDKYFKNHTSPDVDLGDISGINASVVNIQKEIDR      1179
MT040336.1       CDVVIGIVNNTVYDPLQPELDSFKEELDKYFKNHTSPDVDLGDISGINASVVNIQKEIDR      1179
MT040335.1       CDVVIGIVNNTVYDPLQPELDSFKEELDKYFKNHTSPDVDLGDISGINASVVNIQKEIDR      1179
MN996532.2       CDVVIGIVNNTVYDPLQPELDSFKEELDKYFKNHTSPDVDLGDISGINASVVNIQKEIDR      1181
MN996529.1       CDVVIGIVNNTVYDPLQPELDSFKEELDKYFKNHTSPDVDLGDISGINASVVNIQKEIDR      1185
MN988713.1       CDVVIGIVNNTVYDPLQPELDSFKEELDKYFKNHTSPDVDLGDISGINASVVNIQKEIDR      1185
MT072688.1       CDVVIGIVNNTVYDPLQPELDSFKEELDKYFKNHTSPDVDLGDISGINASVVNIQKEIDR      1185
MN994467.1       CDVVIGIVNNTVYDPLQPELDSFKEELDKYFKNHTSPDVDLGDISGINASVVNIQKEIDR      1185
NC_045512.2      CDVVIGIVNNTVYDPLQPELDSFKEELDKYFKNHTSPDVDLGDISGINASVVNIQKEIDR      1185
MT093571.1       CDVVIGIVNNTVYDPLQPELDSFKEELDKYFKNHTSPDVDLGDISGINASVVNIQKEIDR      1185
MG772933.1       CDVVIGIINNTVYDPLQPELDSFKEELDKYFKNHTSPDIDLGDISGINASVVNIQKEIDR      1158
MG772934.1       CDVVIGIINNTVYDPLQPELDSFKEELDKYFKNHTSPDIDLGDISGINASVVNIQKEIDR      1157
KY417145.1       CDVVIGIINNTVYDPLQPELDSFKEELDKYFKNHTSPDVDLGDISGINASVVNIQKEIDR      1146
KP886809.1       CDVVIGIINNTVYDPLQPELDSFKEELDKYFKNHTSPDVDLGDISGINASVVNIQKEIDR      1153
KY417143.1       CDVVIGIINNTVYDPLQPELDSFKEELDKYFKNHTSPDVDLGDISGINASVVNIQKEIDR      1153
FJ588686.1       CDVVIGIINNTVYDPLQPELDSFKEELDKYFKNHTSPDVDLGDISGINASVVNIQKEIDR      1153
KY417148.1       CDVVIGIINNTVYDPLQPELDSFKEELDKYFKNHTSPDVDLGDISGINASVVNIQKEIDR      1154
KY417147.1       CDVVIGIINNTVYDPLQPELDSFKEELDKYFKNHTSPDVDLGDISGINASVVNIQKEIDR      1153
KY417142.1       CDVVIGIINNTVYDPLQPELDSFKEELDKYFKNHTSPDVDLGDISGINASVVNIQKEIDR      1153
GQ153542.1       CDVVIGIINNTVYDPLQPELDSFKEELDKYFKNHTSPDVDLGDISGINASVVNIQKEIDR      1154
DQ022305         CDVVIGIINNTVYDPLQPELDSFKEELDKYFKNHTSPDVDLGDISGINASVVNIQKEIDR      1154
                 *******:**************************** ::.*..:. ******:*::**::
```

CoVAX_MNS#5

NC_014470.1	LNEIAKSLNESLIDLQELGKYEQYIKWPWYVWLGFIAGLIAIVMATIMLCCMTSCCSCLK	1231
KF569996.1	LNEVAKNLNESLIDLQELGKYEQYIKWPWYVWLGFIAGLIAIVMATILLCCMTSCCSCLK	1231
KY417144.1	LNEVAKNLNESLIDLQELGKYEQYIKWPWYVWLGFIAGLIAIVMVTILLCCMTSCCSCLK	1228
KC881005.1	LNEVAKNLNESLIDLQELGKYEQYIKWPWYVWLGFIAGLIAIVMVTILLCCMTSCCSCLK	1228
KC881006.1	LNEVAKNLNESLIDLQELGKYEQYIKWPWYVWLGFIAGLIAIVMVTILLCCMTSCCSCLK	1228
KF367457.1	LNEVAKNLNESLIDLQELGKYEQYIKWPWYVWLGFIAGLIAIVMVTILLCCMTSCCSCLK	1228
KY417152.1	LNEVAKNLNESLIDLQELGKYEQYIKWPWYVWLGFIAGLIAIVMVTILLCCMTSCCSCLK	1228
KY417151.1	LNEVAKNLNESLIDLQELGKYEQYIKWPWYVWLGFIAGLIAIVMVTILLCCMTSCCSCLK	1228
KY417146.1	LNEVAKNLNESLIDLQELGKYEQYIKWPWYVWLGFIAGLIAIVMVTILLCCMTSCCSCLK	1227
KT444582.1	LNEVAKNLNESLIDLQELGKYEQYIKWPWYVWLGFIAGLIAIVMVTILLCCMTSCCSCLK	1227
AY278488.2	LNEVAKNLNESLIDLQELGKYEQYIKWPWYVWLGFIAGLIAIVMVTILLCCMTSCCSCLK	1227
AY274119	LNEVAKNLNESLIDLQELGKYEQYIKWPWYVWLGFIAGLIAIVMVTILLCCMTSCCSCLK	1227
AP006560.1	LNEVAKNLNESLIDLQELGKYEQYIKWPWYVWLGFIAGLIAIVMVTILLCCMTSCCSCLK	1227
AP006557.1	LNEVAKNLNESLIDLQELGKYEQYIKWPWYVWLGFIAGLIAIVMVTILLCCMTSCCSCLK	1227
AY485277.1	LNEVAKNLNESLIDLQELGKYEQYIKWPWYVWLGFIAGLIAIVMVTILLCCMTSCCSCLK	1227
AY572038.1	LNEVAKNLNESLIDLQELGKYEQYIKWPWYVWLGFIAGLIAIVMVTILLCCMTSCCSCLK	1227
AY572034.1	LNEVAKNLNESLIDLQELGKYEQYIKWPWYVWLGFIAGLIAIVMVTILLCCMTSCCSCLK	1227
MT040334.1	LNEVAKNLNESLIDLQELGKYEQYIKWPWYIWLGFIAGLIAIIMVTIMLCCMTSCCSCLK	1237
MW532698.1	LNEVAKNLNESLIDLQELGKYEQYIKWPWYIWLGFIAGLIAIIMVTIMLCCMTSCCSCLK	1241
MT040333.1	LNEVAKNLNESPIDLQELGKYEQYIKWPWYIWLGFIAGLIAIIMVTIMLCCMTSCCSCLK	1239
MT040336.1	LNEVAKNLNESLIDLQELGKYEQYIKWPWYIWLGFIAGLIAIIMVTIMLCCMTSCCSCLK	1239
MT040335.1	LNEVAKNLNESLIDLQELGKYEQYIKWPWYIWLGFIAGLIAIIMVTIMLCCMTSCCSCLK	1239
MN996532.2	LNEVAKNLNESLIDLQELGKYEQYIKWPWYIWLGFIAGLIAIIMVTIMLCCMTSCCSCLK	1241
MN996529.1	LNEVAKNLNESLIDLQELGKYEQYIKWPWYIWLGFIAGLIAIVMVTIMLCCMTSCCSCLK	1245
MN988713.1	LNEVAKNLNESLIDLQELGKYEQYIKWPWYIWLGFIAGLIAIVMVTIMLCCMTSCCSCLK	1245
MT072688.1	LNEVAKNLNESLIDLQELGKYEQYIKWPWYIWLGFIAGLIAIVMVTIMLCCMTSCCSCLK	1245
MN994467.1	LNEVAKNLNESLIDLQELGKYEQYIKWPWYIWLGFIAGLIAIVMVTIMLCCMTSCCSCLK	1245
**NC_045512.2**	**LNEVAKNLNESLIDLQELGKYEQYIKWPWYIWLGFIAGLIAIVMVTIMLCCMTSCCSCLK**	**1245**
MT093571.1	LNEVAKNLNESLIDLQELGKYEQYIKWPWYIWLGFIAGLIAIVMVTIMLCCMTSCCSCLK	1245
MG772933.1	LNEVARNLNESLIDLQELGKYEQYIKWPWYVWLGFIAGLIAIVMVTILLCCMTSCCSCLK	1218
MG772934.1	LNEVARNLNESLIDLQELGKYEHYIKWPWYVWLGFIAGLIAIVMVTILLCCMTSCCSCLK	1217
KY417145.1	LNEVAKNLNESLIDLQELGKYEQYIKWPWYVWLGFIAGLIAIVMATILLCCMTSCCSCLK	1206
KP886809.1	LNEVAKNLNDSLIDLQELGKYEQYIKWPWYVWLGFIAGLVGLFMAIILLCYFTSCCSCCK	1213
KY417143.1	LNEVAKNLNESLIDLQELGKYEQYIKWPWYVWLGFIAGLIAIVMATILLCCMTSCCSCLK	1213
FJ588686.1	LNEVAKNLNESLIDLQELGKYEQYIKWPWYVWLGFIAGLIAIVMATILLCCMTSCCSCLK	1213
KY417148.1	LNEVAKNLNESLIDLQELGKYEQYIKWPWYVWLGFIAGLIAIVMATILLCCMTSCCSCLK	1214
KY417147.1	LNEVAKNLNESLIDLQELGKYEQYIKWPWYVWLGFIAGLIAIVMATILLCCMTSCCSCLK	1213
KY417142.1	LNEVAKNLNESLIDLQELGKYEQYIKWPWYVWLGFIAGLIAIVMATILLCCMTSCCSCLK	1213
GQ153542.1	LNEVAKNLNESLIDLQELGKYEQYIKWPWYVWLGFIAGLIAIVMVTILLCCMTSCCSCLK	1214
DQ022305	LNEVAKNLNESLIDLQELGKYEQYIKWPWYVWLGFIAGLIAIVMVTILLCCMTSCCSCLK	1214
	:*:.**:* *******:*******:********:.:.*. *:** :****** *	

NC_014470.1	GVCSCASCCKFDEDHSEPVLTGVKLHYT	1259
KF569996.1	GACSCGSCCKFDEDDSEPVLKGVKLHYT	1259
KY417144.1	GACSCGSCCKFDEDDSEPVLKGVKLHYT	1256
KC881005.1	GACSCGSCCKFDEDDSEPVLKGVKLHYT	1256
KC881006.1	GACSCGSCCKFDEDDSEPVLKGVKLHYT	1256
KF367457.1	GACSCGSCCKFDEDDSEPVLKGVKLHYT	1256
KY417152.1	GACSCGSCCKFDEDDPEPVLKGVKLHYT	1256
KY417151.1	GACSCGSCCKFDEDDSEPVLKGVKLHYT	1256
KY417146.1	GACSCGSCCKFDEDDSEPVLKGVKLHYT	1255
KT444582.1	GACSCGSCCKFDEDDSEPVLKGVKLHYT	1255
AY278488.2	GACSCGSCCKFDEDDSEPVLKGVKLHYT	1255
AY274119	GACSCGSCCKFDEDDSEPVLKGVKLHYT	1255
AP006560.1	GACSCGSCCKFDEDDSEPVLKGVKLHYT	1255
AP006557.1	GACSCGSCCKFDEDDSEPVLKGVKLHYT	1255
AY485277.1	GACSCGSCCKFDEDDSEPVLKGVKLHYT	1255
AY572038.1	GACSCGSCCKFDEDDSEPVLKGVKLHYT	1255
AY572034.1	GACSCGSCCKFDEDDSEPVLKGVKLHYT	1255
MT040334.1	GCCSCGSCCKFDEDDSEPVLKGVKLHYT	1265
MW532698.1	GCCSCGSCCKFDEDDSEPVLKGVKLHYT	1269
MT040333.1	GCCSCGSCCKFDEDDSEPVLKGVKLHYT	1267
MT040336.1	GCCSCGSCCKFDEDDSEPVLKGVKLHYT	1267
MT040335.1	GCCSCGSCCKFDEDDSEPVLKGVKLHYT	1267
MN996532.2	GCCSCGSCCKFDEDDSEPVLKGVKLHYT	1269
MN996529.1	GCCSCGSCCKFDEDDSEPVLKGVKLHYT	1273
MN988713.1	GCCSCGSCCKFDEDDSEPVLKGVKLHYT	1273
MT072688.1	GCCSCGSCCKFDEDDSEPVLKGVKLHYT	1273
MN994467.1	GCCSCGSCCKFDEDDSEPVLKGVKLHYT	1273
**NC_045512.2**	**GCCSCGSCCKFDEDDSEPVLKGVKLHYT**	**1273**
MT093571.1	GCCSCGSCCKFDEDDSEPVLKGVKLHYT	1273
MG772933.1	GCCSCGSCCKFDEDDSEPVLKGVKLHYT	1246
MG772934.1	GCCSCGFCCKFDEDDSEPVLKGVKLHYT	1245
KY417145.1	GACSCGSCCKFDEDDSEPVLKGVKLHYT	1234
KP886809.1	GMCSCGSCCRFDEDDSEPVLKGVKLHYT	1241
KY417143.1	GACSCGSCCKFDEDDSEPVLKGVKLHYT	1241
FJ588686.1	GACSCGSCCKFDEDDSEPVLKGVKLHYT	1241
KY417148.1	GACSCGSCCKFDEDDSEPVLKGVKLHYT	1242
KY417147.1	GACSCGSCCKFDEDDSEPVLKGVKLHYT	1241
KY417142.1	GACSCGSCCKFDEDDSEPVLKGVKLHYT	1241
GQ153542.1	GACSCGSCCKFDEDDSEPVLKGVKLHYT	1242
DQ022305	GACSCGSCCKFDEDDSEPVLKGVKLHYT	1242
	* ***. **:****. ****.*******	

Supplemental Figure 4. ORF1ab clustal aligment. Clustal O (version 1.2.4) multiple sequence alignment using the sarbecoviruses listed in Supplemental Table 2. The Wuhan-Hu-1 (NC_045512.2) sequence is indicated in bold, and selected antigens (Supplemental Table 4) are boxed and shaded.

```
MW532698.1      MESLVPGFNEKTHVQLSLPVLQVRDVLVRGFGDSVEEALSEARQHLLDGTCGIIDVEKGV        60
MT040336.1      MESLVPGFNEKTHVQLSLPVLQVRDVLVRGFGDSVEEALSEARQHLLDGTCGIIDVEKGV        60
MT040335.1      MESLVPGFNEKTHVQLSLPVLQVRDVLVRGFGDSVEEALSEARQHLLDGTCGIIDVEKGV        60
MT040334.1      MESLVPGFNEKTHVQLSLPVLQVRDVLVRGFGDSVEEALSEARQHLLDGTCGIIDVEKGV        60
MT040333.1      MESLVPGFNEKTHVQLSLPVLQVRDVLVRGFGDSVEEALSEARQHLLDGTCGIIDVEKGV        60
MN996532.2      MESLVPGFNEKTHVQLSLPVLQVRDVLVRGFGDSVEEALSEARQHLKDGTCGLVEVEKGV        60
MN988713.1      MESLVPGFNEKTHVQLSLPVLQVRDVLVRGFGDSVEEVLSEARQHLKDGTCGLVEVEKGV        60
MT093571.1      MESLVPGFNEKTHVQLSLPVLQVRDVLVRGFGDSVEEVLSEARQHLKDGTCGLVEVEKGV        60
MN996529.1      MESLVPGFNEKTHVQLSLPVLQVRDVLVRGFGDSVEEVLSEARQHLKDGTCGLVEVEKGV        60
MT072688.1      MESLVPGFNEKTHVQLSLPVLQVRDVLVRGFGDSVEEVLSEARQHLKDGTCGLVEVEKGV        60
NC_045512.2     MESLVPGFNEKTHVQLSLPVLQVRDVLVRGFGDSVEEVLSEARQHLKDGTCGLVEVEKGV        60
MN994467.1      MESLVPGFNEKTHVQLSLPVLQVRDVLVRGFGDSVEEVLSEARQHLKDGTCGLVEVEKGV        60
MG772933.1      MESLVPGFNEKTHGPLSLPVLQVRDVLVRGFGDSVEEALSEARQHLKDGTCGLVEVEKGV        60
MG772934.1      MESLVPGFNEKTHVQLSLPVLQVCDVLVRGFGDSVEEALSEARQHLKDGTCGLVEVEKGV        60
NC_014470.1     MESLVPGFNEKTHVQLSLPVLQVRDVLVRGFGDTVEEAVAEARQHLIEGTCGIVDLQKGV        60
GQ153542.1      MESLVLGVNEKTHVQLSLPVLQVRDVLVRGFGDSVEEALSEAREHLKNGTCGLVELEKGV        60
DQ022305        MESLVLGVNEKTHVQLSLPVLQVRDVLVRGFGDSVEEALSEAREHLKNGTCGLVELEKGV        60
KF569996.1      MESLVLGINEKTHVQLSLPVLQVRDVLVRGFGDSVEEALSEAREHLKSGTCGIVELEKGV        60
KP886809.1      MESLVLGVNEKTHVQLSLPVLQVGDVLVRGFGDTVEEALTEAREHLKNGTCGLVELEKGV        60
AY278488.2      MESLVLGVNEKTHVQLSLPVLQVRDVLVRGFGDSVEEALSEAREHLKNGTCGLVELEKGV        60
AY485277.1      MESLVLGVNEKTHVQLSLPVLQVRDVLVRGFGDSVEEALSEAREHLKNGTCGLVELEKGV        60
AP006560.1      MESLVLGVNEKTHVQLSLPVLQVRDVLVRGFGDSVEEALSEAREHLKNGTCGLVELEKGV        60
AP006557.1      MESLVLGVNEKTHVQLSLPVLQVRDVLVRGFGDSVEEALSEAREHLKNGTCGLVELEKGV        60
AY274119        MESLVLGVNEKTHVQLSLPVLQVRDVLVRGFGDSVEEALSEAREHLKNGTCGLVELEKGV        60
AY572038.1      MESLVLGVNEKTHVQLSLPVLQVRDVLVRGFGDSVEEALSEAREHLKNGTCGLVELEKGV        60
AY572034.1      MESLVLGVNEKTHVQLSLPVLQVRDVLVRGFGDSVEEALSEAREHLKNGTCGLVELEKGV        60
FJ588686.1      MESLVLGVNEKTHVQLSLPVLQVRDVLVRGFGDSVEEALSEAREHLKNGTCGLVELEKGV        60
KY417145.1      MESLVLGVNEKTHVQLSLPVLQVRDVLVRGFGDSVEEALSEAREHLKNGTCGLVELEKGV        60
KY417144.1      MESLVLGVNEKTHVQLSLPVLQVRDVLVRGFGDSVEEALSEAREHLKNGTCGLVELEKGV        60
KY417147.1      MESLVLGVNEKTHVQLSLPVLQVRDVLVRGFGDSVEEALSEAREHLKNGTCGLVELEKGV        60
KY417148.1      MESLVLGVNEKTHVQLSLPVLQVRDVLVRGFGDSVEEALSEAREHLKNGTCGLVELEKGV        60
KY417143.1      MESLVLGVNEKTHVQLSLPVLQVRDVLVRGFGDSVEEALSEAREHLKNGTCGLVELEKGV        60
KT444582.1      MESLVLGVNEKTHVQLSLPVLQVRDVLVRGFGDSVEEALSEAREHLKNGTCGLVELEKGV        60
KC881005.1      MESLVLGVNEKTHVQLSLPVLQVRDVLVRGFGDSVEEALSEAREHLKNGTCGLVELEKGV        60
KC881006.1      MESLVLGVNEKTHVQLSLPVLQVRDVLVRGFGDSVEEALSEAREHLKNGTCGLVELEKGV        60
KF367457.1      MESLVLGVNEKTHVQLSLPVLQVRDVLVRGFGDSVEEALSEAREHLKNGTCGLVELEKGV        60
KY417152.1      MESLVLGVNEKTHVQLSLPVLQVRDVLVRGFGDSVEEALSEAREHLKNGTCGLVELEKGV        60
KY417146.1      MESLVLGVNEKTHVQLSLPVLQVRDVLVRGFGDSVEEALSEAREHLKNGTCGLVELEKGV        60
KY417151.1      MESLVLGVNEKTHVQLSLPVLQVRDVLVRGFGDSVEEALSEAREHLKNGTCGLVELEKGV        60
KY417142.1      MESLVLGVNEKTHVQLSLPVLQVRDVLVRGFGDSVEEALSEAREHLKNGTCGLVELEKGV        60
                ***** *.*****  ******** *********:***.::***:** .****:::::***
```

```
MW532698.1        LPQLEQPYVFVKRSDARTAPHGHVMVELVAELDGVQYGRSGETLGVLVPHVGETPVAYRK        120
MT040336.1        LPQLEQPYVFVKRSDARTAPHGHVMVELVAELDGVQYGRSGETLGVLVPHVGETPVAYRK        120
MT040335.1        LPQLEQPYVFVKRSDARTAPHGHVMVELVAELDGVQYGRSGETLGVLVPHVGETPVAYRK        120
MT040334.1        LPQLEQPYVFVKRSDARTAPHGHVMVELVAELDGVQYGRSGETLGVLVPHVGETPVAYRK        120
MT040333.1        LPQLEQPYVFVKRSDARTAPHGHVMVELVAELDGVQYGRSGETLGVLVPHVGETPVAYRK        120
MN996532.2        LPQLEQPYVFIKRSDARTAPHGHVMVELVAELNGIQYGRSGETLGVLVPYVGETPVVYRK        120
MN988713.1        LPQLEQPYVFIKRSXARTAPHGHVMVELVAELEGIQYGRSGETLGVLVPHVGEIPVAYRK        120
MT093571.1        LPQLEQPYVFIKRSDARTAPHGHVMVELVAELEGIQYGRSGETLGVLVPHVGEIPVAYRK        120
MN996529.1        LPQLEQPYVFIKRSDARTAPHGHVMVELVAELEGIQYGRSGETLGVLVPHVGEIPVAYRK        120
MT072688.1        LPQLEQPYVFIKRSDARTAPHGHVMVELVAELEGIQYGRSGETLGVLVPHVGEIPVAYRK        120
NC_045512.2       LPQLEQPYVFIKRSDARTAPHGHVMVELVAELEGIQYGRSGETLGVLVPHVGEIPVAYRK        120
MN994467.1        LPQLEQPYVFIKRSDARTAPHGHVMVELVAELEGIQYGRSGETLGVLVPHVGEIPVAYRK        120
MG772933.1        LPQLEQPYVFIKRSDARTAPHGHVMVELVAELDGIQYGRSGETLGVLVPHVGEVPVAYRK        120
MG772934.1        LPQLEQPYVFIKRSDARTAHHGHVMVELVAELDGIQYGRSGETLGVLVPHVGEVPVAYRK        120
NC_014470.1       LPQLEQPYIFLKRCDARTAPHGHVMVELVAELDGVQYGRSGESLGVLVPHVGETPIGYRK        120
GQ153542.1        LPQLEQPYVFIKRSDALSTNHGHKVVELVAELDGIQFGRSGITLGVLVPHVGETPIAYRN        120
DQ022305          LPQLEQPYVFIKRSDALSTNHGHKVVELVAELDGIQFGRSGITLGVLVPHVGETPIAYRN        120
KF569996.1        LPQLEQPYVFIKRSDAQGTGHGHKVCELVAELDGVQFGRSGITLGVLVPHVGETPIAYRT        120
KP886809.1        LRQLEEPYVFIKRSEALSTTHGHKVVELVAEMNGIQFGRSGTTLGVLVPHVGETPIAYRN        120
AY278488.2        LPQLEQPYVFIKRSDALSTNHGHKVVELVAEMDGIQYGRSGITLGVLVPHVGETPIAYRN        120
AY485277.1        LPQLEQPYVFIKRSDALSTNHGHKVVELVAEMDGIQYGRSGITLGVLVPHVGETPIAYRN        120
AP006560.1        LPQLEQPYVFIKRSDALSTNHGHKVVELVAEMDGIQYGRSGITLGVLVPHVGETPIAYRN        120
AP006557.1        LPQLEQPYVFIKRSDALSTNHGHKVVELVAEMDGIQYGRSGITLGVLVPHVGETPIAYRN        120
AY274119          LPQLEQPYVFIKRSDALSTNHGHKVVELVAEMDGIQYGRSGITLGVLVPHVGETPIAYRN        120
AY572038.1        LPQLEQPYVFIKRSDALSTNHGHKVVELVAEMDGIQYGRSGITLGVLVPHVGETPIAYRN        120
AY572034.1        LPQLEQPYVFIKRSDALSTNHGHKVVELVAEMDGIQYGRSGITLGVLVPHVGETPIAYRN        120
FJ588686.1        LPQLEQPYVFIKRSDALSTNHGHKVVELVAEKEGIQYGRSGITLGVLVPHVGETPIAYRN        120
KY417145.1        LPQLEQPYVFIKRSDALSTNHGHKVVELVAELDGIQYGRSGITLGVLVPHVGETPIAYRN        120
KY417144.1        LPQLEQPYVFIKRSDALSTNHGHKVVELVAELDGIQYGRSGITLGVLVPHVGETPIAYRN        120
KY417147.1        LPQLEQPYVFIKRSDALSTNHGHKVVELVAELDGIQYGRSGITLGVLVPHVGETPIAYRN        120
KY417148.1        LPQLEQPYVFIKRSDALSTNHGHKVVELVAELDGVQYGRSGITLGVLVPHVGETPIAYRN        120
KY417143.1        LPQLEQPYVFIKRSDALSTNHGHKVVELVAELDGIQYGRSGITLGVLVPHVGETPIAYRN        120
KT444582.1        LPQLEQPYVFIKRSDALSTNHGHKVVELVAELDGIQYGRSGITLGVLVPHVGETPIAYRN        120
KC881005.1        LPQLEQPYVFIKRSDALSTNHGHKVVELVAELDGIQYGRSGITLGVLVPHVGETPIAYRN        120
KC881006.1        LPQLEQPYVFIKRSDALSTNHGHKVVELVAELDGIQYGRSGITLGVLVPHVGETPIAYRN        120
KF367457.1        LPQLEQPYVFIKRSDALSTNHGHKVVELVAELDGIQYGRSGITLGVLVPHVGETPIAYRN        120
KY417152.1        LPQLEQPYVFIKRSDALSTNHGHKVVELVAELDGIQYGRSGITLGVLVPHVGETPIAYRN        120
KY417146.1        LPQLEQPYVFIKRSDALSTNHGHKVVELVAELDGIQYGRSGITLGVLVPHVGETPIAYRN        120
KY417151.1        LPQLEQPYVFIKRSDALSTNHGHKVVELVAELDGIQYGRSGITLGVLVPHVGETPIAYRN        120
KY417142.1        LPQLEQPYVFIKRSDALSTNHGHKVVELVAELDGIQYGRSGITLGVLVPHVGETPIAYRN        120
                  * ***:**:*:**. *  : *** : ***** :*:*:**** :******:*** *: **.
```

```
MW532698.1      ILLRKNGNKGAGGHSFGIDLKSYDLGDELGTDPIDDFQVNWNTKHGSGVTRELMRELNGG      180
MT040336.1      ILLRKNGNKGAGGHSFGIDLKSYDLGDELGTDPIDDFQVNWNTKHGSGVTRELMRELNGG      180
MT040335.1      ILLRKNGNKGAGGHSFGIDLKSYDLGDELGTDPIDDFQVNWNTKHGSGVTRELMRELNGG      180
MT040334.1      ILLRKNGNKGAGGHSFGIDLKSYDLGDELGTDPIDDFQVNWNTKHGSGVTRELMRELNGG      180
MT040333.1      ILLRKNGNKGAGGHSFGIDLKSYDLGDELGTDPIDDFQVNWNTKHGSGVTRELMRELNGG      180
MN996532.2      VLLRKNGNKGAGGHSYGADLKSFDLGDELGTDPYEDFQENWNTKHSSGVTRDLMRELNGG      180
MN988713.1      VLLRKNGNKGAGGHSYGADLKSFDLGDELGTDPYEDFQENWNTKHSSGVTRELMRELNGG      180
MT093571.1      VLLRKNGNKGAGGHSYGADLKSFDLGDELGTDPYEDFQENWNTKHSSGVTRELMRELNGG      180
MN996529.1      VLLRKNGNKGAGGHSYGADLKSFDLGDELGTDPYEDFQENWNTKHSSGVTRELMRELNGG      180
MT072688.1      VLLRKNGNKGAGGHSYGADLKSFDLGDELGTDPYEDFQENWNTKHSSGVTRELMRELNGG      180
NC_045512.2     VLLRKNGNKGAGGHSYGADLKSFDLGDELGTDPYEDFQENWNTKHSSGVTRELMRELNGG      180
MN994467.1      VLLRKNGNKGAGGHSYGADLKSFDLGDELGTDPYEDFQENWNTKHSSGVTRELMRELNGG      180
MG772933.1      VLLRKNGNKGAGGHSYGADLKSFDLGDELGTDPIEDFQENWNTKHGSGVTRELKRELNGG      180
MG772934.1      VLLRKNGNKGAGGHSYGADLKSFDLGDELGTDPIEDFQENWNTKHGSGVTRELKRELNGG      180
NC_014470.1     VLVRKNGNKGAGGHLYGADLRFYDLGDELGTDPLDDFQQDWNTKHGSGLRRDLFRELNGG      180
GQ153542.1      VLLRKNGNKGAGGHSFGIDLKSYDLGDELGTDPIEDYEQNWNTKHGSGALRELTRELNGG      180
DQ022305        VLLRKNGNKGAGGHSFGIDLKSYDLGDELGTDPIEDYEQNWNTKHGSGALRELTRELNGG      180
KF569996.1      VLLRKNGNKGAGGHSFGIDLKSYDLGDELGTDPIEDYEQNWNTKHGSGALRELTRELNGG      180
KP886809.1      VLLRKNGNKGAGGHSYGIDLKSYDLGDVLNTDPVEDYEQQWNTKHGSGVLRELIRELNGG      180
AY278488.2      VLLRKNGNKGAGGHSYGIDLKSYDLGDELGTDPIEDYEQNWNTKHGSGALRELTRELNGG      180
AY485277.1      VLLRKNGNKGAGGHSYGIDLKSYDLGDELGTDPIEDYEQNWNTKHGSGALRELTRELNGG      180
AP006560.1      VLLRKNGNKGAGGHSYGIDLKSYDLGDELGTDPIEDYEQNWNTKHGSGALRELTRELNGG      180
AP006557.1      VLLRKNGNKGAGGHSYGIDLKSYDLGDELGTDPIEDYEQNWNTKHGSGALRELTRELNGG      180
AY274119        VLLRKNGNKGAGGHSYGIDLKSYDLGDELGTDPIEDYEQNWNTKHGSGALRELTRELNGG      180
AY572038.1      VLLRKNGNKGAGGHSYGIDLKSYDLGDELGTDPIEDYEQNWNTKHGSGALRELTRELNGG      180
AY572034.1      VLLRKNGNKGAGGHSYGIDLKSYDLGDELGTDPIEDYEQNWNTKHGSGALRELTRELNGG      180
FJ588686.1      VLLRKNGNKGAGGHSYGIDLKSYDLGDELGTDPIEDYEQNWNTKHGSGALRELTRELNGG      180
KY417145.1      VLLRKNGNKGAGGHSFGIDLRSYDLGDELGTDPIEDYEQNWNTKHGSGVLRELTRELNGG      180
KY417144.1      VLLRKNGNKGAGGHSFGIDLRSYDLGDELGTDPIEDYEQNWNTKHGSGVLRELTRELNGG      180
KY417147.1      VLLRKNGNKGAGGHSFGIDLKSYDLGDKLGTDPIEDYEQNWNTKHGSGALRELTRELNGG      180
KY417148.1      VLLRKNGNKGAGGHSFGIDLKSYDLGDELGTDPIEDYEQNWNTKHGSGALRELTRELNGG      180
KY417143.1      VLLRKNGNKGAGGHSFGIDLKSYDLGDELGTDPIEDYEQNWNTKHGSGVLRELTRELNGG      180
KT444582.1      VLLRKNGNKGAGGHSFGIDLKSYDLGDELGTDPIEDYEQNWNTKHGSGVLRELTRELNGG      180
KC881005.1      VLLRKNGNKGAGGHSFGIDLKSYDLGDELGTDPIEDYEQNWNTKHGSGVLRELTRELNGG      180
KC881006.1      VLLRKNGNKGAGGHSFGIDLKSYDLGDELGTDPIEDYEQNWNTKHGSGVLRELTRELNGG      180
KF367457.1      VLLRKNGNKGAGGHSFGIDLKSYDLGDELGTDPIEDYEQNWNTKHGSGVLRELTRELNGG      180
KY417152.1      VLLRKNGNKGAGGHSFGIDLRSYDLGDELGTDPIEDYEQNWNTKHGSGVLRELTRELNGG      180
KY417146.1      VLLRKNGNKGAGGHSFGIDLKSYDLGDELGTDPIEDYEQNWNTKHGSGVLRELTRELNGG      180
KY417151.1      VLLRKNGNKGAGGHSFGIDLKSYDLGDELGTDPIEDYEQNWNTKHGSGALRELTRELNGG      180
KY417142.1      VLLRKNGNKGAGGHSFGIDLKSYDLGDELGTDPIEDYEQNWNTKHGSGALRELTRELNGG      180
                :*:*********** :* **: :**** *.*** :*:: :*****.**  *:* ******
```

```
MW532698.1        AYTRYVDNNFCGPDGYPLECIKDLLARAGKSSCSLSEQLDFIDTKRGVYCCREHEHEIVW         240
MT040336.1        AYTRYVDNNFCGPDGYPLECIKDLLARAGKSSCSLSEQLDFIDTKRGVYCCREHEHEIVW         240
MT040335.1        AYTRYVDNNFCGPDGYPLECIKDLLARAGKSSCSLSEQLDFIDTKRGVYCCREHEHEIVW         240
MT040334.1        AYTRYVDNNFCGPDGYPLECIKDLLARAGKSSCSLSEQLDFIDTKRGVYCCREHEHEIVW         240
MT040333.1        AYTRYVDNNFCGPDGYPLECIKDLLARAGKSSCSLSEQLDFIDTKRGVYCCREHEHEIVW         240
MN996532.2        AYTRYVDNNFCGPDGYPLECIKDLLARAGKASCTLSEQLDFIDTKRGVYCCREHEHEIAW         240
MN988713.1        AYTRYVDNNFCGPDGYPLECIKDLLARAGKASCTLSEQLDFIDTKRGVYCCREHEHEIAW         240
MT093571.1        AYTRYVDNNFCGPDGYPLECIKDLLARAGKASCTLSEQLDFIDTKRGVYCCREHEHEIAW         240
MN996529.1        AYTRYVDNNFCGPDGYPLECIKDLLARAGKASCTLSEQLDFIDTKRGVYCCREHEHEIAW         240
MT072688.1        AYTRYVDNNFCGPDGYPLECIKDLLARAGKASCTLSEQLDFIDTKRGVYCCREHEHEIAW         240
NC_045512.2       AYTRYVDNNFCGPDGYPLECIKDLLARAGKASCTLSEQLDFIDTKRGVYCCREHEHEIAW         240
MN994467.1        AYTRYVDNNFCGPDGYPLECIKDLLARAGKASCTLSEQLDFIDTKRGVYCCREHEHEIAW         240
MG772933.1        AYTRYVDNNFCGPDGYPLECIKDLLARAGKASYALSEQLDFIDTKRGVYCCREHEHEIAW         240
MG772934.1        AYTRYVDNNFCGPDGYPLECIKNFLARAGKASCTLSEQLDFIDTKRGVYCCREHEHEIAW         240
NC_014470.1       VYTRYVDNNFCGPDGYPLECIKDLLARAGKSSAPLAEQLDFLESKRGVYCCREHEHEIAW         240
GQ153542.1        VVTRYVDNNFCGPDGYPLECIKDFLARAGKSMCTLSEQLDYIESKRGVYCCREHEHEIVW         240
DQ022305          VVTRYVDNNFCGPDGYPLECIKDFLARAGKSMCTLSEQLDYIESKRGVYCCREHEHEIVW         240
KF569996.1        VVTRYVDNNFCGPDGYPLECIKDLLARAGKSMCTLSEQLDFIESKRGVYCCREHEHEIAW         240
KP886809.1        AVTRYVDNNFCGPDGYPLECIKGLLARAGKSMCTLSEQLDFIESKRGVYCCREHEHEIAW         240
AY278488.2        AVTRYVDNNFCGPDGYPLDCIKDFLARAGKSMCTLSEQLDYIESKRGVYCCRDHEHEIAW         240
AY485277.1        AVTRYVDNNFCGPDGYPLDCIKDFLARAGKSMCTLSEQLDYIESKRGVYCCRDHEHEIAW         240
AP006560.1        AVTRYVDNNFCGPDGYPLDCIKDFLARAGKSMCTLSEQLDYIESKRGVYCCRDHEHEIAW         240
AP006557.1        AVTRYVDNNFCGPDGYPLDCIKDFLARAGKSMCTLSEQLDYIESKRGVYCCRDHEHEIAW         240
AY274119          AVTRYVDNNFCGPDGYPLDCIKDFLARAGKSMCTLSEQLDYIESKRGVYCCRDHEHEIAW         240
AY572038.1        AVTRYVDNNFCGPDGYPLDCIKDFLARAGKSMCTLSEQLDYIESKRGVYCCRDHEHEIAW         240
AY572034.1        AVTRYVDNNFCGPDGYPLDCIKDFLARAGKSMCTLSEQLDYIESKRGVYCCRDHEHEIAW         240
FJ588686.1        AVTRYVDNNLCGPDGYPLDCIKDFLARAGKSMCTLSEQLDYIESKRGVYCCRDHEHEIAW         240
KY417145.1        AFTRYVDNNFCGPDGYPLDCIKDFLARAGKSMCTLSEQLDYIESKRGVYCCRGHEHEIAW         240
KY417144.1        AFTRYVDNNFCGPDGYPLDCIKDFLARAGKSMCTLSEQLDYIESKRGVYCCRDHEHEIAW         240
KY417147.1        AVTRYVDNNFCGPDGYPLDCIKDFLARAGKSMCTLSEQLDYIESKRGVYCCRDHEHEVAW         240
KY417148.1        AVTRYVDNNFCGPDGYPLDCIKDFLARAGKSMCTLSEQLDYIESKRGVYCCRDHDHEIAW         240
KY417143.1        AVTRYVDNNFCGPDGYPLDCIKDFLARAGKSMCTLSEQLDYIESKRGVYCCRDHEHEIAW         240
KT444582.1        AVTRYVDNNFCGPDGYPLDCIKDFLARAGKSMCTLSEQLDYIESKRGVYCCRDHEHEIAW         240
KC881005.1        AVTRYVDNNFCGPDGYPLDCIKDFLARAGKSMCTLSEQLDYIESKRGVYCCRDHEHEIAW         240
KC881006.1        AFTRYVDNNFCGPDGYPLDCIKDFLARAGKSMCTLSEQLDYIESKRGVYCCRDHEHEVAW         240
KF367457.1        AFTRYVDNNFCGPDGYPLDCIKDFLARAGKSMCTLSEQLDYIESKRGVYCCRDHEHEVAW         240
KY417152.1        AFTRYVDNNFCGPDGYPLDCIKDFLARAGKSMCTLSEQLDYIESKRGVYCCRDHEHEIAW         240
KY417146.1        AFTRYVDNNFCGPDGYPLDCIKDFLARAGKSMCTLSEQLDYIESKRGVYCCRDHEHEVAW         240
KY417151.1        AFTRYVDNNFCGPDGYPLDCIKDFLARAGKSMCTLSEQLDYIESKRGVYCCRDHEHEVAW         240
KY417142.1        AFTRYVDNNFCGPDGYPLDCIKDFLARAGKSMCTLSEQLDYIESKRGVYCCRDHEHEVAW         240
                  . *******:********:***.:******:   *:****::::******** *:**:.*
```

```
MW532698.1        YTERSDKSYELQIPFEIKLAKKFDNFTGECPNFVFPLNSTIKTIQPRVEKKKLEGFMGRI          300
MT040336.1        YTERSDKSYELQTPFEIKLAKKFDNFTGECPNFVFPLNSTIKTIQPRVEKKKLEGFMGRI          300
MT040335.1        YTERSDKSYELQTPFEIKLAKKFDNFTGECPNFVFPLNSTIKTIQPRVEKKKLEGFMGRI          300
MT040334.1        YTERSDKSYELQTPFEIKLAKKFDNFTGECPNFVFPLNSTIKTIQPRVEKKKLEGFMGRI          300
MT040333.1        YTERSDKSYELQTPFEIKLAKKFDNFTGECPNFVFPLNSTIKTIQPRVEKKKLEGFMGRI          300
MN996532.2        YTERSEKSYELQTPFEIKLAKKFDTFNGECPNFVFPLNSTIKTIQPRVEKKKLDGFMGRI          300
MN988713.1        YTERSEKSYELQTPFEIKLAKKFDTFNGECPNFVFPLNSIIKTIQPRVEKKKLDGFMGRI          300
MT093571.1        YTERSEKSYELQTPFEIKLAKKFDTFNGECPNFVFPLNSIIKTIQPRVEKKKLDGFMGRI          300
MN996529.1        YTERSEKSYELQTPFEIKLAKKFDTFNGECPNFVFPLNSIIKTIQPRVEKKKLDGFMGRI          300
MT072688.1        YTERSEKSYELQTPFEIKLAKKFDTFNGECPNFVFPLNSIIKTIQPRVEKKKLDGFMGRI          300
NC_045512.2       YTERSEKSYELQTPFEIKLAKKFDTFNGECPNFVFPLNSIIKTIQPRVEKKKLDGFMGRI          300
MN994467.1        YTERSEKSYELQTPFEIKLAKKFDTFNGECPNFVFPLNSIIKTIQPRVEKKKLDGFMGRI          300
MG772933.1        YTERSEKSYELQTPFEIKLAKKFDTFNGECPNFVFPLNSTIKTIQPRVEKKKLDGFMGRI          300
MG772934.1        YTERSEKSYESQTPFEIKLAKKFDTFNGECPNFVFPLNSTIKTIQPRVEKKKLDGFMGRI          300
NC_014470.1       YTERSDKSYELQTPFDITNAKKFDSFKGECPKFVFPLNSTVKVLQPRVEKKKTEGFLGRI          300
GQ153542.1        FTERSEKSYEHQTPFEIKSAKKFDTFKGECPKFVFPLNSKVKVIQPRVEKKKTEGFMGRI          300
DQ022305          FTERSEKSYEHQTPFEIKSAKKFDTFKGECPKFVFPLNSKVKVIQPRVEKKKTEGFMGRI          300
KF569996.1        FTERSEKSYEHQTPFDIKSAKKFDTFKGECPKFVFPLNSKVKVIQPRVEKKKTEGFMGRI          300
KP886809.1        YTERSDKSYEHQTPFDIKSAKKFDTFKEECPKFVFPLNSTVKVIQPRVEKKKTEGFMGRI          300
AY278488.2        FTERSDKSYEHQTPFEIKSAKKFDTFKGECPKFVFPLNSKVKVIQPRVEKKKTEGFMGRI          300
AY485277.1        FTERSDKSYEHQTPFEIKSAKKFDTFKGECPKFVFPLNSKVKVIQPRVEKKKTEGFMGRI          300
AP006560.1        FTERSDKSYEHQTPFEIKSAKKFDTFKGECPKFVFPLNSKVKVIQPRVEKKKTEGFMGRI          300
AP006557.1        FTERSDKSYEHQTPFEIKSAKKFDTFKGECPKFVFPLNSKVKVIQPRVEKKKTEGFMGRI          300
AY274119          FTERSDKSYEHQTPFEIKSAKKFDTFKGECPKFVFPLNSKVKVIQPRVEKKKTEGFMGRI          300
AY572038.1        FTERSDKSYEHQTPFEIKSAKKFDTFKGECPKFVFPLNSKVKVIQPRVEKKKTEGFMGRI          300
AY572034.1        FTERSDKSYEHQTPFEIKSAKKFDTFKGECPKFVFPLNSKVKVIQPRVEKKKTEGFMGRI          300
FJ588686.1        FTERSDKSYEHQTPFEIKSAKKFDTFKGECPKFVFPLNSKVKVIQPRVEKKKTEGFMGRI          300
KY417145.1        FTERSDKSYEHQTPFEIKSAKKFDTFKGECPKFVFPLNSKVKVIQPRVEKKKTEGFMGRI          300
KY417144.1        FTERSDKSYEHQTPFEIKSAKKFDTFKGECPKFVFPLNSKVKVIQPRVEKKKTEGFMGRI          300
KY417147.1        FTERSDKSYEHQTPFEIKSAKKFDTFKGECPKFVFPLDSKVKVIQPRVEKKKTEGFMGRI          300
KY417148.1        FTERSDKSYEHQTPFEIKSAKKFDTFKGECPKFVFPLNSKVKVIQPRVEKKKTEGFMGRI          300
KY417143.1        FTERSDKSYEHQTPFEIKSAKKFDTFKGECPKFVFPLNSKVKVIQPRVEKKKTEGFMGRI          300
KT444582.1        FTERSDKSYEHQTPFEIKSAKKFDTFKGECPKFVFPLNSKVKVIQPRVEKKKTEGFMGRI          300
KC881005.1        FTERSDKSYEHQTPFEIKSAKKFDTFKGECPKFVFPLNSKVKVIQPRVEKKKTEGFMGRI          300
KC881006.1        FTERSDKSYEHQTPFEIKSAKKFDTFKGECPKFVFPLDSKVKVIQPRVEKKKTEGFMGRI          300
KF367457.1        FTERSDKSYEHQTPFEIKSAKKFDTFKGECPKFVFPLDSKVKVIQPRVEKKKTEGFMGRI          300
KY417152.1        FTERSDKSYEHQTPFEIKSAKKFDTFKGECPKFVFPLNSKVKVIQPRVEKKKTEGFMGRI          300
KY417146.1        FTERSDKSYEHQTPFEIKSAKKFDTFKGECPKFVFPLNSKVKVIQPRVDKKKTEGFMGRI          300
KY417151.1        FTERSDKSYEHQTPFEIKSAKKFDTFKGECPKFVFPLNSKVKVIQPRVEKKKTEGFMGRI          300
KY417142.1        FTERSDKSYEHQTPFEIKSAKKFDTFKGECPKFVFPLNSKVKVIQPRVEKKKTEGFMGRI          300
                  :****:**** * **:*. *****.*. ***:*****:* :*.:****:*** :**:***
```

```
MW532698.1      RSVYPVASPNECNPMHLSTLMKCEHCSETSWQTGDFLKATCEFCGTENQVKEGPTTCGYL   360
MT040336.1      RSVYPVASPNECNPMHLSTLMKCEHCSETSWQTGDFLKATCEFCGTENQVKEGPTTCGYL   360
MT040335.1      RSVYPVASPNECNPMHLSTLMKCEHCSETSWQTGDFLKATCEFCGTENQVKEGPTTCGYL   360
MT040334.1      RSVYPVASPNECNPMHLSTLMKCEHCSETSWQTGDFLKATCEFCGTENQVKEGPTTCGYL   360
MT040333.1      RSVYPVASPNECNPMHLSTLMKCEHCSETSWQTGDFLKATCEFCGTENQVKEGPTTCGYL   360
MN996532.2      RSVYPVASPNECNQMCLSTLMKCDHCGETSWQTGDFVKATCEFCGTENLTKEGATTCGYL   360
MN988713.1      RSVYPVASPNECNQMCLSTLMKCDHCGETSWQTGDFVKATCEFCGTENLTKEGATTCGYL   360
MT093571.1      RSVYPVASPNECNQMCLSTLMKCDHCGETSWQTGDFVKATCEFCGTENLTKEGATTCGYL   360
MN996529.1      RSVYPVASPNECNQMCLSTLMKCDHCGETSWQTGDFVKATCEFCGTENLTKEGATTCGYL   360
MT072688.1      RSVYPVASPNECNQMCLSTLMKCDHCGETSWQTGDFVKATCEFCGTENLTKEGATTCGYL   360
NC_045512.2     RSVYPVASPNECNQMCLSTLMKCDHCGETSWQTGDFVKATCEFCGTENLTKEGATTCGYL   360
MN994467.1      RSVYPVASPNECNQMCLSTLMKCDHCGETSWQTGDFVKATCEFCGTENLTKEGATTCGYL   360
MG772933.1      RSVYPVASPNECNQMCLSILMKCDHCGETSWQTGDFVRATCEFCGTENLTKEGATTCGYL   360
MG772934.1      RSVYPVASPNECNQMCLSTLMKCDHCGETSWQTGDFVRATCEFCGTENLTKEGATTCGYL   360
NC_014470.1     RTVYQVASPGECNSMHLSTYMNCNHCGEKSWQTCDFLTATCEMCGNQNTVEEGPTTCGYV   360
GQ153542.1      RSVYPVATPQECNDMHLSTLMKCNHCDAVSWQTCDFLKATCEQCGTENLVCEGPTTCGYL   360
DQ022305        RSVYPVATPQECNDMHLSTLMKCNHCDEVSWQTCDFLKATCEQCGTENLVCEGPTTCGYL   360
KF569996.1      RSVYPVASPQECNDMHLSTLMKCNHCDEVSWQTCDFLKATCEQCGTENLVCEGPTTCGYL   360
KP886809.1      RSVYPVASPQECNNMHLSTLMKCSHCDEVSWQTCDFLKATCEHCGTENLVVEGPTTCGYL   360
AY278488.2      RSVYPVASPQECNNMHLSTLMKCNHCDEVSWQTCDFLKATCEHCGTENLVIEGPTTCGYL   360
AY485277.1      RSVYPVASPQECNNMHLSTLMKCNHCDEVSWQTCDFLKATCEHCGTENLVIEGPTTCGYL   360
AP006560.1      RSVYPVASPQECNNMHLSTLMKCNHCDEVSWQTCDFLKATCEHCGTENLVIEGPTTCGYL   360
AP006557.1      RSVYPVASPQECNNMHLSTLMKCNHCDEVSWQTCDFLKATCEHCGTENLVIEGPTTCGYL   360
AY274119        RSVYPVASPQECNNMHLSTLMKCNHCDEVSWQTCDFLKATCEHCGTENLVIEGPTTCGYL   360
AY572038.1      RSVYPVASPQECNNMHLSTLMKCNHCDEVSWQTCDFLKATCEHCGTENLVIEGPTTCGYL   360
AY572034.1      RSVYPVASPQECNNMHLSTLMKCNHCDEVSWQTCDFLKATCEHCGTENLVIEGPTTCGYL   360
FJ588686.1      RSVYPVASPQECNNMHLSTLMKCNHCDEVSWQTCDFLKATCEHCGIENLITEGPTTCGYL   360
KY417145.1      RSVYSVASPQECNNMHLSTLMKCNHCDEVSWQTCDFLKATCEHCGTENSIIEGPTTCGYL   360
KY417144.1      RSVYPVASPQECNNMHLSTLMKCNHCDEVSWQTCDFLKATCEHCGTENLVTEGPTTCGYL   360
KY417147.1      RSVYPVASPQECNNMHLSTLMKCNHCDEVSWQTCDFLKATCEHCGTENSVTEGPTTCGYL   360
KY417148.1      RSVYPVASPQECNNMHLSTLMKCNHCDEVSWQTCDFLKATCEHCGTENSITEGPTTCGYL   360
KY417143.1      RSVYPVASPQECNNMHLSTLMKCNHCDEVSWQTCDFLKATCEHCGTENAVTEGPTTCGYL   360
KT444582.1      RSVYPVASPQECNNMHLSTLMKCNHCDEVSWQTCDFLKATCEHCGTENAVTEGPTTCGYL   360
KC881005.1      RSVYPVASPQECNNMHLSTLMKCNHCDEVSWQTCDFLKATCEHCGTENSVTEGPTTCGYL   360
KC881006.1      RSVYPVASPQECNNMHLSTLMKCNHCDEVSWQTCDFLKATCEHCGTENSVTEGPTTCGYL   360
KF367457.1      RSVYPVASPQECNNMHLSTLMKCNHCDEVSWQTCDFLKATCEHCGTENSVTEGPTTCGYL   360
KY417152.1      RSVYPVASPQECNNMHLSTLMKCNHCDEVSWQTCDFLKATCEHCGTENLVTEGPTTCGYL   360
KY417146.1      RSVYPVASPQECNNMHLSTLMKCNHCDEVSWQTCDFLKATCEQCGTENLVTEGPTTCGYL   360
KY417151.1      RSVYPVASPQECNNMHLSTLMKCNHCDEVSWQTCDFLKATCEHCGTENLVTEGPTTCGYL   360
KY417142.1      RSVYPVASPQECNNMHLSTLMKCNHCDEVSWQTCDFLKATCEHCGTENLVTEGPTTCGYL   360
                *:** **:* *** * **  *:*.**.  **** **: **** ** :*   ** *****:
```

MW532698.1	PQNAVVKIFCPACHNPEMGPEHSLAEYHNESGIKTTLRKGGRTKAFGGCVFSYVGCHNKC	420
MT040336.1	PQNAVVKIFCPACHNPEMGPEHSLAEYHNESGIKTTLRKGGRTKAFGGCVFSYVGCHNKC	420
MT040335.1	PQNAVVKIFCPACHNPEMGPEHSLAEYHNESGIKTTLRKGGRTKAFGGCVFSYVGCHNKC	420
MT040334.1	PQNAVVKIFCPACHNPEMGPEHSLAEYHNESGIKTTLRKGGRTKAFGGCVFSYVGCHNKC	420
MT040333.1	PQNAVVKIFCPACHNPEMGPEHSLAEYHNESGIKTTLRKGGRTKAFGGCVFSYVGCHNKC	420
MN996532.2	PQNAVVKIYCPACHNPEVGPEHSLAEYHNESGLKPILRKGGRTIAFGGCVFSYVGCYNKC	420
MN988713.1	PQNAVVKIYCPACHNSEVGPEHSLAEYHNESGLKTILRKGGRTIAFGGCVFSYVGCHNKC	420
MT093571.1	PQNAVVKIYCPACHNSEVGPEHSLAEYHNESGLKTILRKGGRTIAFGGCVFSYVGCHNKC	420
MN996529.1	PQNAVVKIYCPACHNSEVGPEHSLAEYHNESGLKTILRKGGRTIAFGGCVFSYVGCHNKC	420
MT072688.1	PQNAVVKIYCPACHNSEVGPEHSLAEYHNESGLKTILRKGGRTIAFGGCVFSYVGCHNKC	420
**NC_045512.2**	**PQNAVVKIYCPACHNSEVGPEHSLAEYHNESGLKTILRKGGRTIAFGGCVFSYVGCHNKC**	**420**
MN994467.1	PQNAVVKIYCPACHNSEVGPEHSLAEYHNESGLKTILRKGGRTIAFGGCVFSYVGCHNKC	420
MG772933.1	PQNAVVKLYCPACHNPEVGPEHSLAEYHNESGLKTVLRKGGRTIAYGGCVFAYVGCYNKC	420
MG772934.1	PQNAVVKIYCPACHNPEVGPEHSLAEYHNESGLKTVLRKGGRTIAYGGCVFAYVGCYNKC	420
NC_014470.1	PSNAVVKMVCPACQNPEIGPDHSVADYHNNSKIETRLRKGGRIKSFGGCVFSYVGCYNKR	420
GQ153542.1	PTNAVVKMPCPACQDPEVGPEHSVADYHNHSNIETRLRKGGRTKCFGGCVFSYVGCYNKR	420
DQ022305	PTNAVVKMPCPACQDPEVGPEHSVADYHNHSNIETRLRKGGRTKCFGGCVFSYVGCYNKR	420
KF569996.1	PTNAVLKMPCPACQDPEIGPEHSVADYHNHSNIETRLRKGGRTKCFGGCVFAYVGCYNKR	420
KP886809.1	PINAVVKMPCPACQNKEVGPEHSVADYHNYSNIETRLRKGGRTKCFGGCVFSYVGCYNKR	420
AY278488.2	PTNAVVKMPCPACQDPEIGPEHSVADYHNHSNIETRLRKGGRTRCFGGCVFAYVGCYNKR	420
AY485277.1	PTNAVVKMPCPACQDPEIGPEHSVADYHNHSNIETRLRKGGRTRCFGGCVFAYVGCYNKR	420
AP006560.1	PTNAVVKMPCPACQDPEIGPEHSVADYHNHSNIETRLRKGGRTRCFGGCVFAYVGCYNKR	420
AP006557.1	PTNAVVKMPCPACQDPEIGPEHSVADYHNHSNIETRLRKGGRTRCFGGCVFAYVGCYNKR	420
AY274119	PTNAVVKMPCPACQDPEIGPEHSVADYHNHSNIETRLRKGGRTRCFGGCVFAYVGCYNKR	420
AY572038.1	PTNAVVKMPCPACQDPEIGPEHSVADYHNHSNVETRLRKGGRTRCFGGCVFAYVGCYNKR	420
AY572034.1	PTNAVVKMPCPACQDPEIGPEHSVADYHNHSNIETRLRKGGRTRCFGGCVFAYVGCYNKR	420
FJ588686.1	PTNAVVKMPCPACQDPEIGPEHSVADYHNHSNIETRLRKGGRTRCFGGCVFAYVGCYNKR	420
KY417145.1	PTNAVVKMPCPACQDPEIGPEHSVADYHNHSNIETRLRKGGRTRCFGGCVFAYVGCYNKR	420
KY417144.1	PTNAVVKMPCPACQDPEIGPEHSVADYHNHSNIETRLRKGGRTRCFGGCVFAYVGCYNKR	420
KY417147.1	PTNAVVKMPCPACQDPEIGPEHSVADYHNHSNIETRLRKGGRTRCFGGCVFAYVGCYNKR	420
KY417148.1	PTNAVVKMPCPACQDPEIGPEHSVADYHNHSNIETRLRKGGRTRCFGGCVFAYVGCYNKR	420
KY417143.1	PTNAVVKMPCPACQDQEIGPEHSVADYHNHSNIETRLRKGGRTRCFGGCVFAYVGCYNKR	420
KT444582.1	PTNAVVKMPCPACQDQEIGPEHSVADYHNHSNIETRLRKGGRTRCFGGCVFAYVGCYNKR	420
KC881005.1	PTNAVVKMPCPACQDPEIGPEHSVVDYHNHSNIETRLRKGGRTRCFGGCVFAYVGCYNKR	420
KC881006.1	PTNAVVKMPCPACQDPEIGPEHSVADYHNHSNIETRLRKGGRTRCFGGCVFAYVGCYNKR	420
KF367457.1	PTNAVVKMPCPACQDPEIGPEHSVADYHNHSNIETRLRKGGRTRCFGGCVFAYVGCYNKR	420
KY417152.1	PTNAVVKMPCPACQDPEIGPEHSVADYHNHSNIETRLRKGGRTRCFGGCVFAYVGCYNKR	420
KY417146.1	PTNAVVKMPCPACQDPEIGPEHSVADYHNHSNIETRLRKGGRTRCFGGCVFAYVGCYNKR	420
KY417151.1	PTNAVVKMPCPACQDPEIGPEHSVADYHNHSNIETRLRKGGRTRCFGGCVFAYVGCYNKR	420
KY417142.1	PTNAVVKMPCPACQDPEIGPEHSVADYHNHSNIETRLRKGGRTRCFGGCVFAYVGCYNKR	420
	* ***:*: ****:: *:**:**:.:*** * :: ****** .:*****:****:**	

```
MW532698.1      AYWVPRAAANVGCNHTGVVGEGSESLNDNLLEILTKEKVNINIVGDFKLTEEIAIILASF      480
MT040336.1      AYWVPRAAANVGCNHTGVVGEGSESLNDNLLEILTKEKVNINIVGDFKLTEEIAIILASF      480
MT040335.1      AYWVPRAAANVGCNHTGVVGEGSESLNDNLLEILTKEKVNINIVGDFKLTEEIAIILASF      480
MT040334.1      AYWVPRAAANVGCNHTGVVGEGSESLNDNLLEILTKEKVNINIVGDFKLTEEIAIILASF      480
MT040333.1      AYWVPRAAANVGCNHTGVVGEGSESLNDNLLEILTKEKVNINIVGDFKLTEEIAIILASF      480
MN996532.2      AYWIPRASANIGCNHTGVVGEGSEGLNDNLLEILQKEKVNINIVGDFKLNEEIAIILASF      480
MN988713.1      AYWVPRASANIGCNHTGVVGEGSEGLNDNLLEILQKEKVNINIVGDFKLNEEIAIILASF      480
MT093571.1      AYWVPRASANIGCNHTGVVGEGSEGLNDNLLEILQKEKVNINIVGDFKLNEEIAIILASF      480
MN996529.1      AYWVPRASANIGCNHTGVVGEGSEGLNDNLLEILQKEKVNINIVGDFKLNEEIAIILASF      480
MT072688.1      AYWVPRASANIGCNHTGVVGEGSEGLNDNLLEILQKEKVNINIVGDFKLNEEIAIILASF      480
NC_045512.2     AYWVPRASANIGCNHTGVVGEGSEGLNDNLLEILQKEKVNINIVGDFKLNEEIAIILASF      480
MN994467.1      AYWVPRANANIGCNHTGVVGEGSEGLNDNLLEILQKEKVNINIVGDFKLNEEIAIILASF      480
MG772933.1      AYWVPRASANIGCNHTGVVGEGSESLNDNLLEILQKEKVNINIVGDFKLNEEIAIILASF      480
MG772934.1      AYWVPRASANIGCNHTGVVGEGSESLNDNLLEILQKEKVNINIVGDFKLNEEIAIILASF      480
NC_014470.1     AFWVPRAAANIGSNHTGVVGEGVETMNEDLLQILSRERVVINIVGEFCLNEEIAILLASL      480
GQ153542.1      AYWVPRASADIGANHTGITGENVETLNEDLLEILNRERVNINIVGDFQLNDEIAIILASF      480
DQ022305        AYWVPRASANIGANHTGITGENVETLNEDLLEILNRERVNINIVGDFRFNEEVAIILASF      480
KF569996.1      AYWVPRASADIGANHTGITGDNVETLNEDLLEILHRERVNINIVGDFQLTEEVAIILASF      480
KP886809.1      AYWVPRASADIGSNHTGIVGDNVETLNEDLLEILSRERVNINIVGDFQLNEEVAIILASL      480
AY278488.2      AYWVPRASADIGSGHTGITGDNVETLNEDLLEILSRERVNINIVGDFHLNEEVAIILASF      480
AY485277.1      AYWVPRASADIGSGHTGITGDNVETLNEDLLEILSRERVNINIVGDFHLNEEVAIILASF      480
AP006560.1      AYWVPRASADIGSGHTGITGDNVETLNEDLLEILSRERVNINIVGDFHLNEEVAIILASF      480
AP006557.1      AYWVPRASADIGSGHTGITGDNVETLNEDLLEILSRERVNINIVGDFHLNEEVAIILASF      480
AY274119        AYWVPRASADIGSGHTGITGDNVETLNEDLLEILSRERVNINIVGDFHLNEEVAIILASF      480
AY572038.1      AYWVPRASADIGSGHTGITGDNVETLNEDLLEILSRERVNINIVGDFHLNEEVAIILASF      480
AY572034.1      AYWVPRASADIGSGHTGITGDNVETLNEDLLEILSRERVNINIVGDFHLNEEVAIILASF      480
FJ588686.1      AYWVPRASADIGSGHTGITGDNVETLNEDLLEILSRERVNINIVGDFQLNEEVAIILASF      480
KY417145.1      AYWVPRASADIGSGHTGITGDNVETLNEDLLEILNRERVNINIVGDFQLNEEVAIILASF      480
KY417144.1      AYWVPRASADIGSGHTGITGDNVETLNEDLLEILSRERVNINIVGDFQLTEEVAIILASF      480
KY417147.1      AYWVPRASADIGSGHTGITGDNVETLNEDLLEILSRERVNINIVGDFQLNEEVAIILASF      480
KY417148.1      AYWVPRASADIGSGHTGITGDNVETLNEDLLEILSRERVNINIVGDFQLNEEVAIILASF      480
KY417143.1      AYWVPRASADIGSGHTGITGDNVETLNEDLLEILSRERVNINIVGDFQLNEEVAIILASF      480
KT444582.1      AYWVPRASADIGSGHTGITGDNVETLNEDLLEILSRERVNINIVGDFQLNEEVAIILASF      480
KC881005.1      AYWVPRASADIGSGHTGITGDNVETLNEDLLEILSRERVNINIVGDFQLTEEVAIILASF      480
KC881006.1      AYWVPRASADIGSGHTGITGDNVETLNEDLLEILSRERVNINIVGDFQLNEEVAIILASF      480
KF367457.1      AYWVPRASADIGSGHTGITGDNVETLNEDLLEILSRERVNINIVGDFQLNEEVAIILASF      480
KY417152.1      AYWVPRASADIGSGHTGITGDNVETLNEDLLEILSRERVNINIVGDFQLTEEVAIILASF      480
KY417146.1      AYWVPRASADIGSGHTGITGDNVETLNEDLLEILSRERVNINIVGDFQLTEEVAIILASF      480
KY417151.1      AYWVPRASADIGSGHTGITGDNVETLNEDLLEILSRERVNINIVGDFQLTEEVAIILASF      480
KY417142.1      AYWVPRASADIGSGHTGITGDNVETLNEDLLEILSRERVNINIVGDFQLTEEVAIILASF      480
                *:*:*** *::*..***:.*:. * :*::**:** :*:* *****:* :.:*:**:***:
```

```
MW532698.1      SASTSAFVETVKGLDYKSFKQIVESCGNFKVTKGKFKKNAWNIGEPKSILSPLYAFPSEA    540
MT040336.1      SASTSAFVETVKGLDCKSFKQIVESCGNFKVTKGKFKKNAWNIGEPKSILSPLYAFPSEA    540
MT040335.1      SASTSAFVETVKGLDYKSFKQIVESCGNFKVTKGKFKKNAWNIGEPKSILSPLYAFPSEA    540
MT040334.1      SASTSAFVETVKGLDYKSFKQIVESCGNFKVTKGKFKKNAWNIGEPKSILSPLYAFPSEA    540
MT040333.1      SASTCAFVETVKGLDYKSFKQIVESCGNFKVTKGKFKKNAWNIGEPKSILSPLYAFPSEA    540
MN996532.2      SASTSAFVETVKGLDYKTFKQIVESCGNFKVTKGKAKKGAWNIGEQKSILSPLYAFASEA    540
MN988713.1      SASTSAFVETVKGLDYKAFKQIVESCGNFKVTKGKAKKGAWNIGEQKSILSPLYAFASEA    540
MT093571.1      SASTSAFVETVKGLDYKAFKQIVESCGNFKVTKGKAKKGAWNIGEQKSILSPLYAFASEA    540
MN996529.1      SASTSAFVETVKGLDYKAFKQIVESCGNFKVTKGKAKKGAWNIGEQKSILSPLYAFASEA    540
MT072688.1      SASTSAFVETVKGLDYKAFKQIVESCGNFKVTKGKAKKGAWNIGEQKSILSPLYAFASEA    540
NC_045512.2     SASTSAFVETVKGLDYKAFKQIVESCGNFKVTKGKAKKGAWNIGEQKSILSPLYAFASEA    540
MN994467.1      SASTSAFVETVKGLDYKAFKQIVESCGNFKVTKGKAKKGAWNIGEQKSILSPLYAFASEA    540
MG772933.1      SASISAFVETVKGLDYKTFKQIVESCGNFKVTKGKAKKGAWNIGEQSSILSPLYAFPSDA    540
MG772934.1      SASTSAFVETVKGLDYKTFKQIVESCGNFKVTKGKAKKGAWNIGEQKSILSPLYAFASEA    540
NC_014470.1     SASTSAFVETVKNLDFKTFKKIIESCGNYKVTKGKFKPGVWNIGTSKSLLTPLHCFSSQA    540
GQ153542.1      SASASAFIETVRGLDYKSFKAIVESCGNYKVTKGKPVAGAWNIGQQRSILTPLCGFPSQA    540
DQ022305        SASPSAFIETVKGLDYKSFKVIVESCGNYKVTNGKPVTGAWNIGQQRSILTPLCGFPSQA    540
KF569996.1      SASTSVFIDTVKGLDYKTFKAIVESCGNYKVTRGKPVQGAWNIGQQKSILTPLCGFPSQA    540
KP886809.1      SASTSAFIDTIKSLDYKSFKSIVESCGNYKVTKGKPIKGAWNIGQQRSVLTPLCGFPSQA    540
AY278488.2      SASTSAFIDTIKSLDYKSFKTIVESCGNYKVTKGKPVKGAWNIGQQRSVLTPLCGFPSQA    540
AY485277.1      SASTSAFIDTIKSLDYKSFKTIVESCGNYKVTKGKPVKGAWNIGQQRSVLTPLCGFPSQA    540
AP006560.1      SASTSAFIDTIKSLDYKSFKTIVESCGNYKVTKGKPVKGAWNIGQQRSVLTPLCGFPSQA    540
AP006557.1      SASTSAFIDTIKSLDYKSFKTIVESCGNYKVTKGKPVKGAWNIGQQRSVLTPLCGFPSQA    540
AY274119        SASTSAFIDTIKSLDYKSFKTIVESCGNYKVTKGKPVKGAWNIGQQRSVLTPLCGFPSQA    540
AY572038.1      SASTSAFIDTIKSLDYKSFKTIVESCGNYKVTKGKPVKGAWNIGQQRSVLTPLCGFPSQA    540
AY572034.1      SASTSAFIDTIKSLDYKSFKTIVESCGNYKVTKGKPVKGAWNIGQQRSVLTPLCGFPSQA    540
FJ588686.1      SASTSAFIDTIKSLDYKSFKSIVESCGNYKVTKGKPVKGAWNIGQQRSVLTPLCGFPSQA    540
KY417145.1      SASTSAFIDTIKSLDYKSFKSIVESCGNYKVTKGKPVKGAWNIGQRRSVLTPLCGFPSQA    540
KY417144.1      SASTSAFIDTIKSLDYKSFKAIVESCGNYKVTKGKPVKGAWNIGQQRSVLTPLCGFPSQA    540
KY417147.1      SASTSAFIDTIRSLDYKSFKAIVESCGNYKVTKGKPVKGAWNIGQQRSVLTPLCGFPSQA    540
KY417148.1      SASTSAFIDTIKSLDYKSFKSIVESCGNYQVTKGKSVKGAWNIGQQRSVLTPLCGFPSQA    540
KY417143.1      SASTSAFIDTIRSLDYKSFKAIVESCGNYKVTKGKPVKGAWNIGQQRSVLTPLCGFPSQA    540
KT444582.1      SASTSAFIDTIRSLDYKSFKAIVESCGNYKVTKGKPVKGAWNIGQQRSVLTPLCGFPSQA    540
KC881005.1      SASTSAFIDTIKSLDYKSFKAIVESCGNYKVTKGKPVKGAWNIGQQRSVLTPLCGFPSQA    540
KC881006.1      SASTSAFIDTIRSLDYKSFKAIVESCGNYKVTKGKPVKGAWNIGQQRSVLTPLCGFPSQA    540
KF367457.1      SASTSAFIDTIRSLDYKSFKAIVESCGNYKVTKGKPVKGAWNIGQQRSVLTPLCGFPSQA    540
KY417152.1      SASTSAFIDTIKSLDYKSFKAIVESCGNYKVTKGKPVKGAWNIGQQRSILTPLCGFPSQA    540
KY417146.1      SASTSAFIDTIKSLDYKSFKAIVESCGNYKVTKGKPVKGAWNIGQQRSVLTPLCGFPSQA    540
KY417151.1      SASTSAFIGTIKSLDYKSFKAIVESCGNYKVTKGKPVKGAWNIGQQRSVLTPLCGFPSQA    540
KY417142.1      SASTSAFIGTIKSLDYKSFKAIVESCGNYKVTKGKPVKGAWNIGQQRSVLTPLCGFPSQA    540
                *** ..*: *::.** *:** *:*****::**.**   ..****   *:*:**  * *:*
```

```
MW532698.1       ARVVRSIFSRTLETAQHSVRVLQKAAITILDGISQYSLRLIDAMLFTSELTTDSIVVMAY       600
MT040336.1       ARVVRSIFSRTLETAQHSVRVLQKAAITILDGISQYSLRLIDAMLFTSELTTDSIVVMAY       600
MT040335.1       ARVVRSIFSRTLETAQHSVRVLQKAAITILDGISQYSLRLIDAMLFTSELTTDSIVVMAY       600
MT040334.1       ARVVRSIFSRTLETAQHSVRVLQKAAITILDGISQYSLRLIDAMLFTSELTTDSIVVMAY       600
MT040333.1       ARVVRSIFSRTLETAQHSVRVLQKAAITILDGISQYSLRLIDAMLFTSELTTDSIVVMAY       600
MN996532.2       ARVVRSIFSRTLETAQNSVRALQKAAITILDGISQYSLRLIDAMMFTSDLVTNNLVVMAY       600
MN988713.1       ARVVRSIFSRTLETAQNSVRVLQKAAITILDGISQYSLRLIDAMMFTSDLATNNLVVMAY       600
MT093571.1       ARVVRSIFSRTLETAQNSVRVLQKAAITILDGISQYSLRLIDAMMFTSDLATNNLVVMAY       600
MN996529.1       ARVVRSIFSRTLETAQNSVRVLQKAAITILDGISQYSLRLIDAMMFTSDLATNNLVVMAY       600
MT072688.1       ARVVRSIFSRTLETAQNSVRVLQKAAITILDGISQYSLRLIDAMMFTSDLATNNLVVMAY       600
NC_045512.2      ARVVRSIFSRTLETAQNSVRVLQKAAITILDGISQYSLRLIDAMMFTSDLATNNLVVMAY       600
MN994467.1       ARVVRSIFSRTLETAQNSVRVLQKAAITILDGISQYSLRLIDAMMFTSDLATNNLVVMAY       600
MG772933.1       AHVVRSIFSRTLETAHHSVHVLQKAAIIILDGISQYSLRLIDAMMFTSDLVTNNLVVMAY       600
MG772934.1       ARVVRSIFSRTLETAQYSVRVLQQAAITILDGISQYSLRLIDAMMFTSDLVTNNLVVMAY       600
NC_014470.1      AGVVRSIFSRTLATANHSIVDLHRAAMIIFSDISDQANRVLDAMVNTSDLVTESVVVMAY       600
GQ153542.1       AGVIRSVFSRTLDAANHSIPDLQRAAVTTLDGISEQSLRLVDAMVYTSDLLTNSVVVMAY       600
DQ022305         AGVIRSIFSRTLDAANHSILDLQRAAVTTLDGISEQSLRLVDAMVYTSDLLTNSVVVMAY       600
KF569996.1       ASVIRAIFSRTLDAANHSIPDLQKAAVTILDGISEHSLRLVDAMTYTSDLLTNSVIVMAY       600
KP886809.1       AGVIRSIFARTLDAANHSIPDLQRAAVTILGGISEQSLRLVDAMVYTSDLLTNSVIVMAY       600
AY278488.2       AGVIRSIFARTLDAANHSIPDLQRAAVTILDGISEQSLRLVDAMVYTSDLLTNSVIIMAY       600
AY485277.1       AGVIRSIFARTLDAANHSIPDLQRAAVTILDGISEQSLRLVDAMVYTSDLLTNSVIIMAY       600
AP006560.1       AGVIRSIFARTLDAANHSIPDLQRAAVTILDGISEQSLRLVDAMVYTSDLLTNSVIIMAY       600
AP006557.1       AGVIRSIFARTLDAANHSIPDLQRAAVTILDGISEQSLRLVDAMVYTSDLLTNSVIIMAY       600
AY274119         AGVIRSIFARTLDAANHSIPDLQRAAVTILDGISEQSLRLVDAMVYTSDLLTNSVIIMAY       600
AY572038.1       AGVIRSIFARTLDAANHSIPDLQRAAVTILDGISEQSLRLVDAMVYTSDLLTNSVIIMAY       600
AY572034.1       AGVIRSIFARTLDAANHSIPDLQRAAVTILDGIFEQSLRLVDAMVYTSDLLTNSVIIMAY       600
FJ588686.1       AGVIRSIFARTLDAANHSIPDLQRAAVTILDGISEQSLRLVDAMVYTSDLLTNSVIIMAY       600
KY417145.1       AGVIRSIFARTLDAANHSIPDLQRAAGTILDGISEQSLRLVDAMVYTSDLLTNSVIIMAY       600
KY417144.1       AGVIRSIFARTLDAANHSIPDLQRAAVTILDGISEQSLRLVDAMVYTSDLLTNSVIIMAY       600
KY417147.1       AGVIRSIFARTLDAANHSIPDLQRAAVTILDGISEQSLRLVDAMVYTSDLLTNSVIIMAY       600
KY417148.1       AGVIRSIFARTLDAANHSIPDLQRAAVTILDGISEQSLRLVDAMVYTSDLLTNSVIIMAY       600
KY417143.1       AGVIRSIFARTLDAANHSIPDLQRAAVTILDGISEQSLRLVDAMVYTSDLITNSVIIMAY       600
KT444582.1       AGVIRSIFARTLDAANHSIPDLQRAAVTILDGISEQSLRLVDAMVYTSDLITNSVIIMAY       600
KC881005.1       AGVIRSIFARTLDAANHSIPDLQRAAVTILDGISEQSLRLVDAMVYTSDLLTNSVIIMAY       600
KC881006.1       AGVIRSIFARTLDAANHSIPDLQRAAVTILDGISEQSLRLVDVMVYTSDLLTNSVIIMAY       600
KF367457.1       AGVIRSIFARTLDAANHSIPDLQRAAVTILDGISEQSLRLVDVMVYTSDLLTNSVIIMAY       600
KY417152.1       AGVIRSIFARTLDAANHSIPDLQRAAVTILDGISEQSLRLVDAMVYTSDLLTNSVIIMAY       600
KY417146.1       AGVIRSIFARTLDAANHSIPDLQRAAVTILDGISEQSLRLVDAMVYTSDLLTNSVIIMAY       600
KY417151.1       AGVIRSIFARTLDAANHSIPDLQRAAVTILDGISEQSLRLVDAMVYTSDLITNSVIIMAY       600
KY417142.1       AGVIRSIFARTLDAANHSIPDLQRAAVTILDGISEQSLRLVDAMVYTSDLITNSVIIMAY       600
                 * *:*::*:*** :*: *:  *::**   :..* : : *::*.*  **:* *:.:::***
```

```
MW532698.1      VTGGVVQMTTQWLTNIFGTVYEKLKPILDWLEEKFKEGIEFLKDGWEIVKFITTCSCEII      660
MT040336.1      VTGGVVQMTTQWLTNIFGTVYEKLKPILDWLEEKFKEGIKFLKDGWEIVKFITTCSCEII      660
MT040335.1      VTGGVVQMTTQWLTNIFGTVYEKLKPILDWLEEKFKEGIEFLKDGWEIVKFITTCSCEII      660
MT040334.1      VTGGVVQMTTQWLTNIFGTVYEKLKPILDWLEEKFKEGIEFLKDGWEIVKFITTCSCEII      660
MT040333.1      VTGGVVQMTTQWLTNIFGTVYEKLKPILDWLEEKFKEGIEFLKDGWEIVKFITTCSCEII      660
MN996532.2      ITGGVVQLTSQWLTNIFGTVYEKLKPVLDWLEEKFKEGVEFLRDGWEIVKFISTCACEIV      660
MN988713.1      ITGGVVQLTSQWLTNIFGTVYEKLKPVLDWLEEKFKEGVEFLRDGWEIVKFISTCACEIV      660
MT093571.1      ITGGVVQLTSQWLTNIFGTVYEKLKPVLDWLEEKFKEGVEFLRDGWEIVKFISTCACEIV      660
MN996529.1      ITGGVVQLTSQWLTNIFGTVYEKLKPVLDWLEEKFKEGVEFLRDGWEIVKFISTCACEIV      660
MT072688.1      ITGGVVQLTSQWLTNIFGTVYEKLKPVLDWLEEKFKEGVEFLRDGWEIVKFISTCACEIV      660
NC_045512.2     ITGGVVQLTSQWLTNIFGTVYEKLKPVLDWLEEKFKEGVEFLRDGWEIVKFISTCACEIV      660
MN994467.1      ITGGVVQLTSQWLTNIFGTVYEKLKPVLDWLEEKFKEGVEFLRDGWEIVKFISTCACEIV      660
MG772933.1      ITGGVVQMTSQWLTNIFGTVYEKLKPVLDWLEEKFKEGIEFLRDGWEIVKFISTCACEIV      660
MG772934.1      ITGGVVQMTSQWLTNIFGTVYEKLKPVLDWLEEKFKEGVEFLRDGWEIVKFISTCACEIV      660
NC_014470.1     LTGGLVQQVSTWLSQLLNTSVDKFSAVLRWLEQKLQGGIDFLRQAWGILKLLVTGAYVVI      660
GQ153542.1      VTGGLVQQTMQWLSNMLGTAVDKLKPVFTWVEAKLSAGVEFLRDAWEILKFLITGVFDVI      660
DQ022305        VTGGLVQQTMQWLSNMLGTAVDKLKPVFTWVEAKLSAGVEFLRDAWEILKFLITGVFDVI      660
KF569996.1      VTGGLVQQVSQWLSNLVGSAAEKLRPVFAWVESRLSDGIEFLKDAWEILKFLIRGVFDIV      660
KP886809.1      VTGGLVQQTSQWLSNLLGTTVEKLRPIFAWIEAKLSAGVEFLKDAWEILKFLITGVFDIV      660
AY278488.2      VTGGLVQQTSQWLSNLLGTTVEKLRPIFEWIEAKLSAGVEFLKDAWEILKFLITGVFDIV      660
AY485277.1      VTGGLVQQTSQWLSNLLGTTVEKLRPIFEWIEAKLSAGVEFLKDAWEILKFLITGVFDIV      660
AP006560.1      VTGGLVQQTSQWLSNLLGTTVEKLRPIFEWIEAKLSAGVEFLKDAWEILKFLITGVFDIV      660
AP006557.1      VTGGLVQQTSQWLSNLLGTTVEKLRPIFEWIEAKLSAGVEFLKDAWEILKFLITGVFDIV      660
AY274119        VTGGLVQQTSQWLSNLLGTTVEKLRPIFEWIEAKLSAGVEFLKDAWEILKFLITGVFDIV      660
AY572038.1      VTGGLVQQTSQWLSNLLGTTVEKLRPIFEWIEAKLSAGVEFLKDAWEILKFLITGVFDIV      660
AY572034.1      VTGGLVQQTSQWLSNLLGTTVEKLRPIFEWIEAKLSAGVEFLKDAWEILKFLITGVFDIV      660
FJ588686.1      VTGGLVQQTSQWLSNLLGTTVEKLRPIFVWIEAKLSAGVEFLKDAWEILKFLITGVFDIV      660
KY417145.1      VTGGLVQQTSQWLSNLLGTTVEKLRPIFAWIEAKLSAGVEFLKDAWEILKFLITGVFDIV      660
KY417144.1      VTGGLVQQTSQWLSNLLGTTVEKLRPIFAWIEAKLSAGVEFLKDAWEILKFLITGVFDIV      660
KY417147.1      VTGGLVQQTSQWLSNLLGTTVEKLRPIFAWIEAKLSAGVEFLKDAWEILKFLITGVFDIV      660
KY417148.1      VTGGLVQQTSQWLSNLLGTTVEKLRPIFAWIEAKLSAGVEFLKDAWEILKFLITGVFDIV      660
KY417143.1      VTGGLVQQISQWLSNLLGTTVEKLRPIFAWIEAKLSAGVEFLKDAWEILKFLITGVFDIV      660
KT444582.1      VTGGLVQQISQWLSNLLGTTVEKLRPIFAWIEAKLSAGVEFLKDAWEILKFLITGVFDIV      660
KC881005.1      VTGGLVQQTSQWLSNLLGTTVEKLRPIFAWVEAKLSAGVEFLKDAWEILKFLITGVFDIV      660
KC881006.1      VTGGLVQQTSQWLSNLLGTTVEKLRPIFVWIEAKLSAGVEFLKDAWEILKFLITGVFDIV      660
KF367457.1      VTGGLVQQTSQWLSNLLGTTVEKLRPIFVWIEAKLSAGVEFLKDAWEILKFLITGVFDIV      660
KY417152.1      VTGGLVQQTSQWLSNLLGTTVEKLRPIFAWIEAKLSAGVEFLKDAWEILKFLITGVFDIV      660
KY417146.1      VTGGLVQQTSQWLSNLLGTTVEKLRPIFAWIEAKLSAGVEFLKDAWEILKFLITGVFDIV      660
KY417151.1      VTGGLVQQISQWLSNLLGTTVEKLRPIFAWIEAKFSAGVEFLKDAWEILKFLITGVFDIV      660
KY417142.1      VTGGLVQQISQWLSNLLGTTVEKLRPIFAWIEAKFSAGVEFLKDAWEILKFLITGVFDIV      660
                :***:**    **:::..:  :*:  :: *:* ::. *:.**::.* *:*::      ::
```

```
MW532698.1          GGQLVAFTTELKDSVKKFFKLVNKFLALCADSIVIGGAKLKALNLGETFVAHSRGLYKKC    720
MT040336.1          GGQLVAFTTELKDSVKKFFKLVNKFLALCADSIVIGGAKLKALNLGETFVAHSRGLYKKC    720
MT040335.1          GGQLVAFTTELKDSVKKFFKLVNKFLALCADSIVIGGAKLKALNLGETFVAHSRGLYKKC    720
MT040334.1          GGQLVAFTTELKDSVKKFFKLVNKFLALCADSIVIGGAKLKALNLGETFVAHSRGLYKKC    720
MT040333.1          GGQLVAFTTELKDSVKKFFKLVNKFLALCADSIVIGGAKLKALNLGETFVAHSRGLYKKC    720
MN996532.2          GGQIVTCAKEIKESVQTFFKLVNKFLALCADSIIIGGAKLKALNLGETFVTHSKGLYRKC    720
MN988713.1          GGQIVTCAKEIKESVQTFFKLVNKFLALCADSIIIGGAKLKALNLGETFVTHSKGLYRKC    720
MT093571.1          GGQIVTCAKEIKESVQTFFKLVNKFLALCADSIIIGGAKLKALNLGETFVTHSKGLYRKC    720
MN996529.1          GGQIVTCAKEIKESVQTFFKLVNKFLALCADSIIIGGAKLKALNLGETFVTHSKGLYRKC    720
MT072688.1          GGQIVTCAKEIKESVQTFFKLVNKFLALCADSIIIGGAKLKALNLGETFVTHSKGLYRKC    720
NC_045512.2         GGQIVTCAKEIKESVQTFFKLVNKFLALCADSIIIGGAKLKALNLGETFVTHSKGLYRKC    720
MN994467.1          GGQIVTCAKEIKESVQTFFKLVNKFLALCADSIIIGGAKLKALNLGETFVTHSKGLYRKC    720
MG772933.1          GGQIVTCAKEIKESVQTFFKLVNKFLALCADSIIIGGAKLKALNLGETFVTHSKGLYRKC    720
MG772934.1          GGQIVTRAKEVKESVQTFFKLVNKFLALCADSIIIGGAKLKALNLGETFVTHSKGLYRKC    720
NC_014470.1         RGKIQVVNTSLIECVTSFVDVVNKVFELCTDYITVAGARVRAINFGEVLIAQSRGLYRQC    720
GQ153542.1          KGQIQVATDNIKECVKIFLGVVNKALEMCLDQVTIAGTTLRALNLGEVFIAQSRGLYRQC    720
DQ022305            KGQIQVATDNIKECVKIFLGVVNKALEMCLDQVTIAGTKLRALNLGEVFIAQSRGLYRQC    720
KF569996.1          KGQIQVTSDNIKECVKSFIDVINKALEMCIDYVTVAGTKLRSLNLGEIFIAQSKGLYRQC    720
KP886809.1          KGQIQVASDNIKDCVKCFIDIVNKALEMCIDQVTIAGVKLRSLNLGEVFIAQSKGLYRQC    720
AY278488.2          KGQIQVASDNIKDCVKCFIDVVNKALEMCIDQVTIAGAKLRSLNLGEVFIAQSKGLYRQC    720
AY485277.1          KGQIQVASDNIKDCVKCFIDVVNKALEMCIDQVTIAGAKLRSLNLGEVFIAQSKGLYRQC    720
AP006560.1          KGQIQVASDNIKDCVKCFIDVVNKALEMCIDQVTIAGAKLRSLNLGEVFIAQSKGLYRQC    720
AP006557.1          KGQIQVASDNIKDCVKCFIDVVNKALEMCIDQVTIAGAKLRSLNLGEVFIAQSKGLYRQC    720
AY274119            KGQIQVASDNIKDCVKCFIDVVNKALEMCIDQVTIAGAKLRSLNLGEVFIAQSKGLYRQC    720
AY572038.1          KGQIQVASDNIKDCVKCFIDVVNKALEMCIDQVTIAGVKLRSLNLGEVFIAQSKGLYRQC    720
AY572034.1          KGQIQVASDNIKDCVKCFIDVVNKALEMCIDQVTIAGAKLRSLNLGEVFIAQSKGLYRQC    720
FJ588686.1          KGQIQVASDNIKDCVKCFIDVVNKALEMCIDQVTIAGAKLRSLNLGEVFIAQSKGLYRQC    720
KY417145.1          KGQIQVASDNIKDCVKCFIDVVNKALEMCIDQVTIAGAKLRSLNLGEIFIAQSKGLYRQC    720
KY417144.1          KGQIQVASDNIKDCVKCFIDVVNKALEMCIDQVTIAGAKLRSLNLGEVFIAQSKGLYRQC    720
KY417147.1          KGQIQVASDNIKDCVKCFIDVVNKALEMCIDQVTIAGAKLRSLNLGEVFIAQSKGLYRQC    720
KY417148.1          KGQIQVVSDNIKDCVKCFIDVVNKALEMCIDQVTIAGTKLRSLNLGEVFIAQSKGLYRQC    720
KY417143.1          KGQIQVASDNIKDCVKCFIDVVNKALEMCIDQVTIAGAKLRSLNLGEVFIAQSKGLYRQC    720
KT444582.1          KGQIQVASDNIKDCVKCFIDVVNKALEMCIDQVTIAGAKLRSLNLGEVFIAQSKGLYRQC    720
KC881005.1          KGQIQVALDNIKDCVKCFIDVVNKALEMCIDQVTIAGAKLRSLNLGEVFIAQSKGLYRQC    720
KC881006.1          KGQIQVASDNIKGCVKCFIDVVNKALEMCIDQVTIAGTKLRSLNLGEVFIAQSKGLYRQC    720
KF367457.1          KGQIQVASDNIKGCVKCFIDVVNKALEMCIDQVTIAGTKLRSLNLGEVFIAQSKGLYRQC    720
KY417152.1          KGQIQVASDNIKDCVKCFIDVVNKALEMCIDQVTIAGAKLRSLNLGEVFIAQSKGLYRQC    720
KY417146.1          KGQIQVASDNIKDCVKCFIDVVNKALEMCIDQVTIAGAKLRSLNLGEVFIAQSKGLYRQC    720
KY417151.1          KGQIQVASDNIKDCVKCFIDVVNKALEMCIDQVTIAGVKLRSLNLGEVFIAQSKGLYRQC    720
KY417142.1          KGQIQVASDNIKDCVKCFIDVVNKALEMCIDQVTIAGVKLRSLNLGEVFIAQSKGLYRQC    720
                     *:: .   .:  .*  *. ::** : :* * : :.*. ::::*:** ::::*:***::*
```

```
MW532698.1      VKSRGDSGLLMPLKAPKEVIFLDGETLPTEVLSEEVILKTGELQPLEEPTAQAVEVPLVG    780
MT040336.1      VKSRGDSGLLMPLKAPKEVIFLDGETLPTEVLSEEVILKTGELQPLEEPTAQAVEVPLVG    780
MT040335.1      VKSRGDSGLLMPLKAPKEVIFLDGETLPTEVLSEEVILKTGELQPLEEPTAQAVEVPLVG    780
MT040334.1      VKSRGDSGLLMPLKAPKEVIFLDGETLPTEVLSEEVILKTGELQPLEEPTAQAVEVPLVG    780
MT040333.1      VKSRGDSGLLMPLKAPKEVIFLDGETLPTEVLSEEVILKTGELQPLEEPTAQAVEVPLVG    780
MN996532.2      VKPKEETGLLMPLKAPKEIIFLEGETLPTEVLTEEVVLKTGDLQPLEQPTSEAVEAPLVG    780
MN988713.1      VKSREETGLLMPLKAPKEIIFLEGETLPTEVLTEEVVLKTGDLQPLEQPTSEAVEAPLVG    780
MT093571.1      VKSREETGLLMPLKAPKEIIFLEGETLPTEVLTEEVVLKTGDLQPLEQPTSEAVEAPLVG    780
MN996529.1      VKSREETGLLMPLKAPKEIIFLEGETLPTEVLTEEVVLKTGDLQPLEQPTSEAVEAPLVG    780
MT072688.1      VKSREETGLLMPLKAPKEIIFLEGETLPTEVLTEEVVLKTGDLQPLEQPTSEAVEAPLVG    780
NC_045512.2     VKSREETGLLMPLKAPKEIIFLEGETLPTEVLTEEVVLKTGDLQPLEQPTSEAVEAPLVG    780
MN994467.1      VKSREETGLLMPLKAPKEIIFLEGETLPTEVLTEEVVLKTGDLQPLEQPTSEAVEAPLVG    780
MG772933.1      VRSREETGLLLPLKAPKEIIFLEGETLPTEVLTEEVVLKTGVLQPLEQPTNEAVEAPLIG    780
MG772934.1      VRSREETGLLLPLKAPKEIIFLEGETLPTEVLTEEVVLKTGVLQPLEQPTNEAVEAPLIG    780
NC_014470.1     VRARDQLQLLMPLKSPKDVVFLDGDAYDTLLTSEEVTVKNGTLEALDLELSDVVTGVAEG    780
GQ153542.1      IRGKEQLQLLMPLKAPKEVTFLEGDAHDTVLTSEEVVLKSGELEVLETPIDSFISGAVVG    780
DQ022305        IRGKEQLQLLMPLKAPKEVTFLEGDAHDTVLTSEEVVLKSGELEALETPIDSFTSGAVVG    780
KF569996.1      IRGKEQLQLLMPLKAPKDVTFLEGDVHDTVLTSEEVVLKNGELEALETPVDSFTNGAVVG    780
KP886809.1      IRGKEQLQLLMPLKAPKEVTFLEGDSHDTVLTSEEVVLKNGELEALETPVDSFTNGAVVG    780
AY278488.2      IRGKEQLQLLMPLKAPKEVTFLEGDSHDTVLTSEEVVLKNGELEALETPVDSFTNGAIVG    780
AY485277.1      IRGKEQLQLLMPLKAPKEVTFLEGDSHDTVLTSEEVVLKNGELEALETPVDSFTNGAIVG    780
AP006560.1      IRGKEQLQLLMPLKAPKEVTFLEGDSHDTVLTSEEVVLKNGELEALETPVDSFTNGAIVG    780
AP006557.1      IRGKEQLQLLMPLKAPKEVTFLEGDSHDTVLTSEEVVLKNGELEALETPVDSFTNGAIVG    780
AY274119        IRGKEQLQLLMPLKAPKEVTFLEGDSHDTVLTSEEVVLKNGELEALETPVDSFTNGAIVG    780
AY572038.1      IRGKEQLQLLMPLKAPKEVTFLEGDSHDTVLTSEEVVLKNGELEALETPVDSFTNGAIVG    780
AY572034.1      IRGKEQLQLLMPLKAPKEVTFLEGDSHDTVLTSEEVVLKNGELEALETPVDSFTNGAIVG    780
FJ588686.1      IRGKEQLQLLMPLKAPKEVTFLEGDSHDTVLTSEEVVLKNGELEALETPVDSFKNGAVVG    780
KY417145.1      IRGKEQLQLLMPLKAPKEVTFLEGDSHDTVLTSEEVVLKNGELEALETPVDSFINGAVVG    780
KY417144.1      IRGKEQLQLLMPLKAPKEITFLEGDSHDTVLTSEEVVLKNGELEALETPVDSFTNGAVVG    780
KY417147.1      IRGKEQLQLLMPLKAPKEVTFLEGDSHDTVLTSEEVVLKNGELEALEAPVDSFTKGAVVG    780
KY417148.1      IRGKEQLQLLMPLKAPKEVTFLEGDSHDTVLTSEEVVLKNGELEALEAPVDSFTNGAVVG    780
KY417143.1      IRGKEQLQLLMPLKAPKEVTFLEGDSHDTVLTSEEVVLKNGELEALEAPVDSFTNGAVVG    780
KT444582.1      IRGKEQLQLLMPLKAPKEVTFLEGDSHDTVLTSEEVVLKNGELEALEAPVDSFTNGAVVG    780
KC881005.1      IRGKEQLQLLMPLKAPKEVTFLEGDSHDTVLTSEEVVLKNGELEALETPVDSFTNGAVVG    780
KC881006.1      IRGKEQLQLLMPLKAPKEVTFLEGDSHDTVLTSEEVVLKNGELEALETPVDSFTNGAVVG    780
KF367457.1      IRGKEQLQLLMPLKAPKEVTFLEGDSHDTVLTSEEVVLKNGELEALETPVDSFTNGAVVG    780
KY417152.1      IRGKEQLQLLMPLKAPKEVTFLEGDSHDTVLTSEEVVLKNGELEALETPVDSFTNGAVVG    780
KY417146.1      IRGKEQLQLLMPLKAPKEVTFLEGDSHDTVLTSEEVVLKNGELEALETPVDSFTNGAVVG    780
KY417151.1      IRGKEQLQLLMPLKAPKEVTFLEGDSHDTVLTSEEVVLKNGELEVLETPVDSFTNGAVVG    780
KY417142.1      IRGKEQLQLLMPLKAPKEVTFLEGDSHDTVLTSEEVVLKNGELEALETPVDSFTNGAVVG    780
                :: : :  **:***:**:: **:*:   * : :*** :*.* *: *:    .       *
```

```
MW532698.1        TPVCINGLMLLEIKDTEKYCALAPNMMVTNNTFTLKGGAPTK-VTFGDDTVIEVQGYKNV         839
MT040336.1        TPVCINGLMLLEIKDTEKYCALAPNMMVTNNTFTLKGGAPTK-VTFGDDTVIEVQGYKNV         839
MT040335.1        TPVCINGLMLLEIKDTEKYCALAPNMMVTNNTFTLKGGAPTK-VTFGDDTVIEVQGYKNV         839
MT040334.1        TPVCINGLMLLEIKDTEKYCALAPNMMVTNNTFTLKGGAPTK-VTFGDDTVIEVQGYKNV         839
MT040333.1        TPVCINGLMLLEIKDTEKYCALAPNMMVTNNTFTLKGGAPTK-VTFGDDTVIEVQGYKNV         839
MN996532.2        TPVCINGLMLLEIKDTEKYCALAPNMMVTNNTFTLKGGAPTK-VTFGDDTVIEVQGYKSV         839
MN988713.1        TPVCINGLMLLEIKDTEKYCALAPNMMVTNNTFTLKGGAPTK-VTFGDDTVIEVQGYKSV         839
MT093571.1        TPVCINGLMLLEIKDTEKYCALAPNMMVTNNTFTLKGSAPTK-VTFGDDTVIEVQGYKSV         839
MN996529.1        TPVCINGLMLLEIKDTEKYCALAPNMMVTNNTFTLKGGAPTK-VTFGDDTVIEVQGYKSV         839
MT072688.1        TPVCINGLMLLEIKDTEKYCALAPNMMVTNNTFTLKGGAPTK-VTFGDDTVIEVQGYKSV         839
NC_045512.2       TPVCINGLMLLEIKDTEKYCALAPNMMVTNNTFTLKGGAPTK-VTFGDDTVIEVQGYKSV         839
MN994467.1        TPVCINGLMLLEIKDTEKYCALAPNMMVTNNTFTLKGGAPTK-VTFGDDTVIEVQGYKSV         839
MG772933.1        TPVCINGLMLLEIKDTEKYCALAPNMMVTNNTFTLKGGAPTK-VTFGDDTVIEVQGYKSV         839
MG772934.1        TPVCINGLMLLEIKDTEKYCALAPNMMVTNNTFTLKGGAPTK-VTFGDDTVIEVQGYKSV         839
NC_014470.1       VPVCVNGLMLLELKEKEQYCALSPSLLATNNVFTLKGGAPTKGVTFGEDTVVEIQGYKSV         840
GQ153542.1        TPVCINGLMLLELENKEQYCALSPGLLATNNVFRLKGGAPVKGVTFGENTVLEVQGYKNV         840
DQ022305          TPVCINGLMLLELENKEQYCALSPGLLATNNVFRLKGGAPVKGVTFGEDTVLEVQGYKNV         840
KF569996.1        TPVCINGLMLLELKDKEQYCALSPGLLATNNVFRLKGGAPIRGVTFGEDTVVEVQGYKNV         840
KP886809.1        TPVCVNGLMLLELKDKEQYCALSPGLLATNNVFRLKGGAPIKGVTFGENTVLEVQGYKNV         840
AY278488.2        TPVCVNGLMLLEIKDKEQYCALSPGLLATNNVFRLKGGAPIKGVTFGEDTVWEVQGYKNV         840
AY485277.1        TPVCVNGLMLLEIKDKEQYCALSPGLLATNNVFRLKGGAPIKGVTFGEDTVWEVQGYKNV         840
AP006560.1        TPVCVNGLMLLEIKDKEQYCALSPGLLATNNVFRLKGGAPIKGVTFGEDTVWEVQGYKNV         840
AP006557.1        TPVCVNGLMLLEIKDKEQYCALSPGLLATNNVFRLKGGAPIKGVTFGEDTVWEVQGYKNV         840
AY274119          TPVCVNGLMLLEIKDKEQYCALSPGLLATNNVFRLKGGAPIKGVTFGEDTVWEVQGYKNV         840
AY572038.1        IPVCVNGLMLLEIKDKEQYCALSPGLLATNNVFRLKGGAPIKGVTFGEDTVFEVQGYKNV         840
AY572034.1        IPVCVNGLMLLEIKDKEQYCALSPGLLATNNVFRLKGGAPIKGVTFGEDTVFEVQGYKNV         840
FJ588686.1        TPVCVNGLMLLEIKDKEQYCALSPGLLATNNVFRLRGGAPTKGVTFGEDTVLEVQGYKNV         840
KY417145.1        TPVCVNGLMLLEIEDKEQYCALSPGLLATNNVFRLKGGAPTKGVTFGEDTVLEFQGYKNV         840
KY417144.1        TPVCVNGLMLLEIKDKEQYCALSPGLLATNNVFCLKGGSPTKGVTFGEDTVLEVQGYKNV         840
KY417147.1        TPVCVNGLMLLEIKDKEQYCALSPGLLATNNVFRLKGGAPTKGVTFGEDTVLEVQGYKNV         840
KY417148.1        TPVCVNGLMLLEIKDKEQYCALSPGLLATNNVFRLKGGAPTKGVTFGEDTVLEVQGYKNV         840
KY417143.1        TPVCVNGLMLLEIKDKEQYCALSPGLLATNNVFRLKGGAPTKGVTFGEDTVLEVQGYKNV         840
KT444582.1        TPVCVNGLMLLEIKDKEQYCALSPGLLATNNVFRLKGGAPTKGVTFGEDTVLEVQGYKNV         840
KC881005.1        TPVCVNGLMLLEIKDKEQYCALSPGLLATNNVFRLKGGAPIKGVTFGEDTVLEVQGYKNV         840
KC881006.1        TPVCVNGLMLLEIKDKEQYCALSPGLLATNNVFRLKGGAPTKVVTFGEDTVLEVQGYKNV         840
KF367457.1        TPVCVNGLMLLEIKDKEQYCALSPGLLATNNVFRLKGGAPTKGVTFGEDTVLEVQGYKNV         840
KY417152.1        TPVCVNGLMLLEIKDKEQYCALSPGLLATNNVFRLKGGAPTKGVTFGEDTVLEVQGYKNV         840
KY417146.1        TPVCVNGLMLLEIKDKEQYCALSPGLLATNNVFRLKGGSPIKGVTFGEDTVLEVQGYKNV         840
KY417151.1        TPVCVNGLMLLEIKDKEQYCALSPGLLATNNVFRLKGGAPIKGVTFGEDTVLEVQGYKNV         840
KY417142.1        TPVCVNGLMLLEIKDKEQYCALSPGLLATNNVFRLKGGAPIKGVTFGEDTVLEVQGYKNV         840
                   ***:*******:::.*:****:*.::.***.* *:*.:* : ****::** *.****.*
```

```
MW532698.1      NITFELDERVDKVLNEKCSNYTVELGTNIDELACVVAEAVIKTLQPVSELLTPLGIDLDE        899
MT040336.1      NITFELDERVDKVLNEKCSNYTVELGTNIDELACVVAEAVIKTLQPVSELLTPLGIDLDE        899
MT040335.1      NITFELDERVDKVLNEKCSNYTVELGTNIDELACVVAEAVIKTLQPVSELLTPLGIDLDE        899
MT040334.1      NITFELDERVDKVLNEKCSNYTVELGTNIDELACVVAEAVIKTLQPVSELLTPLGIDLDE        899
MT040333.1      NITFELDERVDKVLNEKCSNYTVELGTNIDELACVVAEAVIKTLQPVSELLTPLGIDLDE        899
MN996532.2      NITFELDERIDKVLNEKCSTYTVELGTEVNEFACVVADAVIKTLQPVSELLIPLGIDLDE        899
MN988713.1      NITFELDERIDKVLNEKCSAYTVELGTEVNEFACVVADAVIKTLQPVSELLTPLGIDLDE        899
MT093571.1      NITFELDERIDKVLNEKCSAYTVELGTEVNEFACVVADAVIKTLQPVSELLTPLGIDLDE        899
MN996529.1      NITFELDERIDKVLNEKCSAYTVELGTEVNEFACVVADAVIKTLQPVSELLTPLGIDLDE        899
MT072688.1      NITFELDERIDKVLNEKCSAYTVELGTEVNEFACVVADAVIKTLQPVSELLTPLGIDLDE        899
NC_045512.2     NITFELDERIDKVLNEKCSAYTVELGTEVNEFACVVADAVIKTLQPVSELLTPLGIDLDE        899
MN994467.1      NITFELDERIDKVLNEKCSAYTVELGTEVNEFACVVADAVIKTLQPVSELLTPLGIDLDE        899
MG772933.1      NITFELDERIDKVLNEKCSNYTVELGTEVNEFACVVADAVIKTLQPVSELIIPLGIDLDE        899
MG772934.1      NITFELDERIDKVLNEKCSSYTVELGTDVNEFACVVADAVIKTLQPVSELLTPLGIDLDE        899
NC_014470.1     KITFELDERVDKVLNEKCASYTVETGTTAEELACVVAESVVKTLQPISELLTPMGIDLDE        900
GQ153542.1      KITFELDERVDKVLNEKCSVYTVESGTEVTEFACVVAEAVVKTLQPVSDLLTPMGIDLDE        900
DQ022305        KITFELDVRVDKVLNEKCSVYTVESGTEVTEFACVVAEAVVKTLQPVSDLLTPMGIDLDE        900
KF569996.1      KITFELDERVDKVLNEKCSVYTVESGTEVSEFACVVAESVVKTLQPVSDLLTNMGIDLDE        900
KP886809.1      KITFELDERVDKVLNEKCSVYTVESGTEVNEFACVVAETVVKTLQPVSDLLTEMGVDLDE        900
AY278488.2      RITFELDERVDKVLNEKCSVYTVESGTEVTEFACVVAEAVVKTLQPVSDLLTNMGIDLDE        900
AY485277.1      RITFELDERVDKVLNEKCSVYTVESGTEVTEFACVVAEAVVKTLQPVSDLLTNMGIDLDE        900
AP006560.1      RITFELDERVDKVLNEKCSVYTVESGTEVTEFACVVAEAVVKTLQPVSDLLTNMGIDLDE        900
AP006557.1      RITFELDERVDKVLNEKCSVYTVESGTEVTEFACVVAEAVVKTLQPVSDLLTNMGIDLDE        900
AY274119        RITFELDERVDKVLNEKCSVYTVESGTEVTEFACVVAEAVVKTLQPVSDLLTNMGIDLDE        900
AY572038.1      RITFELDERVDKVLNEKCSVYTVESGTEVTEFACVVAEAVVKTLQPVSDLLTNMGIDLDE        900
AY572034.1      RITFELDERVDKVLNEKCSVYTVESGTEVTEFACVVAEAVVKTLQPVSDLLTNMGIDLDE        900
FJ588686.1      RITFELDERVDKVLNEKCSVYTVESGTEVTEFACVIAETVVKTLQPVSDLLTNMGIDLDE        900
KY417145.1      RITFELDERVDKVLNEKCSVYTVESGTEVTEFACVVAEAVVKTLQPVSDLLTNMGIDLDE        900
KY417144.1      RITFELDERVDKVLNEKCSVYTVESGTEVTEFACVVAEAVVKTLQPVSDLLTNMGIDLDE        900
KY417147.1      RITFELDERVDKVLNEKCSVYTVESGTEVTEFACVVAEAVVKTLQPVSDLLTNMGIDLDE        900
KY417148.1      RITFELDERVDKVLNEKCSVYTVESGTEVTEFACVVAEAVVKTLQPVSDLLTNMGIDLDE        900
KY417143.1      RITFELDERVDKVLNEKCSVYTVESGTEVTEFACVVAEAVVKTLQPVSDLLTNMGIDLDE        900
KT444582.1      RITFELDERVDKVLNEKCSVYTVESGTEVTEFACVVAEAVVKTLQPVSDLLTNMGIDLDE        900
KC881005.1      RITFELDERVDKVLNEKCSVYTVESGTEVTEFACVVAEAVVKTLQPVSDLLTNMGIDLDE        900
KC881006.1      RITFELDERVDKVLNEKCSVYTVESGTEVTEFACVVAEAVVKTLQPVSDLLTNMGIDLDE        900
KF367457.1      RITFELDERVDKVLNEKCSVYTVESGTEVTEFACVVAEAVVKTLQPVSDLLTNMGIDLDE        900
KY417152.1      RITFELDERVDKVLNEKCSVYTVESGTEVTEFACVVAEAVVKTLQPVSDLLTNMGIDLDE        900
KY417146.1      RITFELDERVDKVLNEKCSVYTVESGTEVTEFACVVAEAVVKTLQPVSDLLTNMGIDLDE        900
KY417151.1      RITFELDERVDKVLNEKCSVYTVESGTEVTEFACVVAEAVVKTLQPVSDLLTNMGIDLDE        900
KY417142.1      RITFELDERVDKVLNEKCSVYTVESGTEVTEFACVVAEAVVKTLQPVSDLLTNMGIDLDE        900
                .****** *:********: **** **   *:***:*::*:*****:*:*:  :*:****
```

```
MW532698.1        WGVATYYLFDESGEYTLSSRMYCSFYPPDEDYEEEYSEEE--QPEQPTQYEYGTESDYKG        957
MT040336.1        WGVATYYLFDESGEYTLSSRMYCSFYPPDEDYEEEYSEEE--QPEQPTQYEYGTESDYKG        957
MT040335.1        WGVATYYLFDESGEYTLSSRMYCSFYPPDEDYEEEYSEEE--QPEQPTQYEYGTESDYKG        957
MT040334.1        WGVATYYLFDESGEYTLSSRMYCSFYPPDEDYEEEYSEEE--QPEQPTQYEYGTESDYKG        957
MT040333.1        WGVATYYLFDESGEYTLSSRMYCSFYPPDEDYEEEYSEEE--QPEQPTQYEYGTESDYKG        957
MN996532.2        WGMATYYLFDESGEFKLASHMYCSFYPPDEDEEEGDCEEE--DFEPPTQYEYGTEDDYQG        957
MN988713.1        WSMATYYLFDESGEFKLASHMYCSFYPPDEDEEEGDCEEE--EFEPSTQYEYGTEDDYQG        957
MT093571.1        WSMATYYLFDESGEFKLASHMYCSFYPPDEDEEEGDCEEE--EFEPSTQYEYGTEDDYQG        957
MN996529.1        WSMATYYLFDESGEFKLASHMYCSFYPPDEDEEEGDCEEE--EFEPSTQYEYGTEDDYQG        957
MT072688.1        WSMATYYLFDESGEFKLASHMYCSFYPPDEDEEEGDCEEE--EFEPSTQYEYGTEDDYQG        957
NC_045512.2       WSMATYYLFDESGEFKLASHMYCSFYPPDEDEEEGDCEEE--EFEPSTQYEYGTEDDYQG        957
MN994467.1        WSMATYYLFDESGEFKLASHMYCSFYPPDEDEEEGDCEEE--EFEPSTQYEYGTEDDYQG        957
MG772933.1        WSMATYYLFDESGEFKLSSHMYCSFYPPED-EGEDDCEEG--QCEPSTQYEYGTEDDYQG        956
MG772934.1        WSMATYYLFDESGEFKLSSHMYCSFYPPED-EGEDDCEEG--QFEPSTQYEYGTEDDYQG        956
NC_014470.1       WSVAKFYLFDESGEAVLSSHMYCSFYPPDEEEEEDL--------EESEDVEYGTEDDYTG        952
GQ153542.1        WSVATFYLFDDAGEEKLSSRMYCSFYPPDEEEDCEECEDEEEISEETCEHEYGTEDDYKG        960
DQ022305          WSVATFYLFDDAGEEKLSSRMYCSFYPPDEEEDCEECEDEE----ETCEHEYGTEDDYKG        956
KF569996.1        WSVATFYLFDDAGEENLSSRMYCSFYPPDEEEDEDAGCEEEEIADETCEHEYGTEDDYQG        960
KP886809.1        WSVATFYLFDDAGEENVSSRMYCSFYPPDEEEEDDVECEE-EEIDETCEHEYGTEDDYQG        959
AY278488.2        WSVATFYLFDDAGEENFSSRMYCSFYPPDEEEEDDAEC-EEEEIDETCEHEYGTEDDYQG        959
AY485277.1        WSVATFYLFDDAGEENFSSRMYCSFYPPDEEEEDDAEC-EEEEIDETCEHEYGTEDDYQG        959
AP006560.1        WSVATFYLFDDAGEENFSSRMYCSFYPPDEEEEDDAEC-EEEEIDETCEHEYGTEDDYQG        959
AP006557.1        WSVATFYLFDDAGEENFSSRMYCSFYPPDEEEEDDAEC-EEEEIDETCEHEYGTEDDYQG        959
AY274119          WSVATFYLFDDAGEENFSSRMYCSFYPPDEEEEDDAEC-EEEEIDETCEHEYGTEDDYQG        959
AY572038.1        WSVATFYLFDDAGEENFSSRMYCSFYPPDEEEEDDAEC-EEEEIDETCEHEYGTEDDYQG        959
AY572034.1        WSVATFYLFDDAGEENFSSRMYCSFYPPDEEEEDDAEC-EEEEIDETCEHEYGTEDDYQG        959
FJ588686.1        WSVATFYLFDDAGEEKLSSRMYCSFYPPDDEDECDEYEEEEEVSEESCAHEYGTEEDYQG        960
KY417145.1        WSVATFYLFDDAGEEKLSSRMYCSFYPPDEEEDCDEYDEEEEVPEESCAHEYGTEEDYRG        960
KY417144.1        WSVATFYLFDDAGEEKLSSRMYCSFYPPDDEEDCDEYEEEEEVLEESCAHEYGTEEDYQG        960
KY417147.1        WSVATFYLFDDAGEEKLSSRMYCSFYPPDDEEDCDEYEEEEEVLEESCAHEYGTEEDYQG        960
KY417148.1        WSVATFYLFDDAGEEKLSSRMYCSFYPPDDEEDCDEYEEEEEVPEESCAHEYGTEEDYQG        960
KY417143.1        WSVATFYLFDDAGEEKLSSRMYCSFYPPDDEEDCDEYEEEEEVPEESCAHEYGTEEDYQG        960
KT444582.1        WSVATFYLFDDAGEEKLSSRMYCSFYPPDDEEDCDEYEEEEEVPEESCAHEYGTEEDYQG        960
KC881005.1        WSVATFYLFDDAGEEKLSSRMYCSFYPPDDEEDCDECEEEEEVLEESCAHEYGTEEDYQG        960
KC881006.1        WSVATFYLFDDAGEEKLSSRMYCSFYPPDDEEDCDEYDEEEEVLEESCAHEYGTEEDYQG        960
KF367457.1        WSVATFYLFDDAGEEKLSSRMYCSFYPPDDEEDCDEYDEEEEVLEESCAHEYGTEEDYQG        960
KY417152.1        WSVATFYLFDDAGEEKLSSRMYCSFYPPDDEEDCDEYEEEEEVPEESCAHEYGTEEDYRG        960
KY417146.1        WSVATFYLFDDAGEEKLSSRMYCSFYPPDDEEDCDEYEEEEEVPEESCAHEYGTEEDYQG        960
KY417151.1        WSVATFYLFDDAGEEKLSSRMYCSFYPPDDEEDCDEYEEEEEVPEESCAHEYGTEEDYQG        960
KY417142.1        WSVATFYLFDDAGEEKLSSRMYCSFYPPDDEEDCDEYEEEEEVPEESCAHEYGTEEDYQG        960
                  *.:*.:****::**  .:*:********::                    *****.** *
```

```
MW532698.1        LPLEFGASSVQ---QQEEQEEDWLETEAEVVEQ---EVTPTEQEEELPITEIVPAVEQT-         1010
MT040336.1        LPLEFGASSVQ---QQEEQEEDWLETEAEVVEQ---EVTPTEQEEELPITEIVPAVEQT-         1010
MT040335.1        LPLEFGASSVQ---QQEEQEEDWLETEAEVVEQ---EVTPTEQEEELPITEIVPAVEQT-         1010
MT040334.1        LPLEFGASSVQ---QQEEQEEDWLETEAEVVEQ---EVTPTEQEEELPITEIVPAVEQT-         1010
MT040333.1        LPLEFGASSVQ---QQEEQEEDWLETEAEVVEQ---EVTPTEQEEELPITEIVPAVEQT-         1010
MN996532.2        KSLEFGATSVTPQ-PEEELEEDWLDDDSQQTVVQEDDSEVNQTTITQSIAEVQPQLEMEP         1016
MN988713.1        KPLEFGATSAALQ-XEEEQEEDWLDDDSQQTVGQQDGSEDNQTTTIQTIVEVQPQLEMEL         1016
MT093571.1        KPLEFGATSAALQ-PEEEQEEDWLDDDSQQTVGQQDGSEDNQTTTIQTIVEVQPQLEMEL         1016
MN996529.1        KPLEFGATSAALQ-PEEEQEEDWLDDDSQQTVGQQDGSEDNQTTTIQTIVEVQPQLEMEL         1016
MT072688.1        KPLEFGATSAALQ-PEEEQEEDWLDDDSQQTVGQQDGSEDNQTTTIQTIVEVQPQLEMEL         1016
NC_045512.2       KPLEFGATSAALQ-PEEEQEEDWLDDDSQQTVGQQDGSEDNQTTTIQTIVEVQPQLEMEL         1016
MN994467.1        KPLEFGATSAALQ-PEEEQEEDWLDDDSQQTVGQQDGSEDNQTTTIQTIVEVQPQLEMEL         1016
MG772933.1        KPLEFGATSFSSSSQEEEQEEDWLESDSQDGQET--AV----EENKIPSVEVPPVLQVES         1010
MG772934.1        KPLEFGATSFSSSSQEEEQEEDWLESDSQDGQET--AV----------------------         992
NC_014470.1       APLEFGASSTVEQDEVHDEEEDWLAPQE--------------------------------         980
GQ153542.1        LPLEFGASTETPHVEEEEEEEDWLDDAI---EAESE------------------------         993
DQ022305          LPLEFGASTETPHVEEEEEEEDWLDDAI---EAEPE------------------------         989
KF569996.1        LPLEFGASTEAVQVEE-EEEEDWLDDTNEQSEVEPQ------------------------         995
KP886809.1        LPLEFGASAETVQVEE-EEEEDWLDDTTEQSEIEHE------------------------         994
AY278488.2        LPLEFGASAETVRVEE-EEEEDWLDDTTEQSEIEPE------------------------         994
AY485277.1        LPLEFGASAETVRVEE-EEEEDWLDDTTEQSEIEPE------------------------         994
AP006560.1        LPLEFGASAETVRVEE-EEEEDWLDDTTEQSEIEPE------------------------         994
AP006557.1        LPLEFGASAETVRVEE-EEEEDWLDDTTEQSEIEPE------------------------         994
AY274119          LPLEFGASAETVRVEE-EEEEDWLDDTTEQSEIEPE------------------------         994
AY572038.1        LPLEFGASAETVRVEE-EEEEDWLDDTTEQSEIEPE------------------------         994
AY572034.1        LPLEFGASAETVRVEE-EEEEDWLDDTTEQSEIEPE------------------------         994
FJ588686.1        LPLEFGASTE-MQVEE-EEEEDWLDDATELSEHELE------------------------         994
KY417145.1        LPLEFGASTE-MQVEE-EEEEDWLGDATELSEHELE------------------------         994
KY417144.1        LPLEFGASTE-MQVEE-EEEEDWLGDATELSEHEPE------------------------         994
KY417147.1        LPLEFGASTE-MQVEE-EEEEDWLGDATELSEHEPE------------------------         994
KY417148.1        LPLEFGASTE-MQVEE-EEEEDWLGDATELSEHEPE------------------------         994
KY417143.1        LPLEFGASTE-MQVEE-EEEEDWLGDATELSEHEPE------------------------         994
KT444582.1        LLLEFGASTE-MQVEE-EEEEDWLGDATELSEHEPE------------------------         994
KC881005.1        LPLEFGASTE-MQVEE-EEEEDWLGDATELSEHELE------------------------         994
KC881006.1        LSLEFGASTE-MQVEE-EEEEDWLGDATELSEHEPE------------------------         994
KF367457.1        LSLEFGASTE-MQVEE-EEEEDWLGDATELSEHEPE------------------------         994
KY417152.1        LPLEFGASTE-MQVEE-EEEEDWLGDATDLSEHEPE------------------------         994
KY417146.1        LPLEFGASTE-MQVEE-EEEEDWLGDATELSEHEPE------------------------         994
KY417151.1        LPLEFGASTE-MQVEE-EEEEDWLGDATELSEHEPE------------------------         994
KY417142.1        LPLEFGASTE-MQVEE-EEEEDWLGDATELSEHEPE------------------------         994
                    *****::        : *****
```

```
MW532698.1          -T--IVELECDNFTGYLKLTDNVSIKNVDIVSEAKNVKPTIVVNAANVHLKHGGGVAGAL         1067
MT040336.1          -T--IVELECDNFTGYLKLTDNVSIKNVDIVSEAKNVKPTIVVNAANVHLKHGGGVAGAL         1067
MT040335.1          -T--IVELECDNFTGYLKLTDNVSIKNVDIVSEAKNVKPTIVVNAANVHLKHGGGVAGAL         1067
MT040334.1          -T--IVELECDNFTGYLKLTDNVSIKNVDIVSEAKNVKPTIVVNAANVHLKHGGGVAGAL         1067
MT040333.1          -T--IVELECDNFTGYLKLTDNVSIKNVDIVSEAKNVKPTIVVNAANVHLKHGGGVAGAL         1067
MN996532.2          TPVV-QT-EVNSFSGYLKLTDNVYIKNADIVEEAKKVKPTIVVNAANVYLKHGGGVAGAL         1074
MN988713.1          TPVV-QTIEVNSFSGYLKLTDNVYIKNADIVEEAKKVKPTVVVNAANVYLKHGGGVAGAL         1075
MT093571.1          TPVV-QTIEVNSFSGYLKLTDNVYIKNADIVEEAKKVKPTVVVNAANVYLKHGGGVAGAL         1075
MN996529.1          TPVV-QTIEVNSFSGYLKLTDNVYIKNADIVEEAKKVKPTVVVNAANVYLKHGGGVAGAL         1075
MT072688.1          TPVV-QTIEVNSFSGYLKLTDNVYIKNADIVEEAKKVKPTVVVNAANVYLKHGGGVAGAL         1075
NC_045512.2         TPVV-QTIEVNSFSGYLKLTDNVYIKNADIVEEAKKVKPTVVVNAANVYLKHGGGVAGAL         1075
MN994467.1          TPVV-QTIEVNSFSGYLKLTDNVYIKNADIVEEAKKVKPTVVVNAANVYLKHGGGVAGAL         1075
MG772933.1          TPVVTETSEQNNFTGYLKLTDNVFIKNADIVEEAKKVKPTVVVNAANVYLKHGGGVAGAL         1070
MG772934.1          ----TKTSEQNNFTGYLKLTDNVFIKNADIVEEAKKVKPTVVVNAANIYLKHGGGVAGAL         1048
NC_014470.1         ----ESEVLYDQFTDYHKLTDNVFIKCADIVEESLKVNPTVVVNAANIHLKHGGGVARAL         1036
GQ153542.1          -PEPLPEEPVNQFVGYLKLTDNVAIKCIDIVKEAQSAKPTVIVNAANTHLKHGGGVAGAL         1052
DQ022305            -PEPLPEEPVNQFVGYLKLTDNVAIKCIDIVKEAQSAKPTVIVNAANTHLKHGGGVAGAL         1048
KF569996.1          -PEPTLEESVNQFTGYLKLTDNVAIKCVDIVKEAQNANPMVIVNAANIHLKHGGGVAGAL         1054
KP886809.1          -PESTPEEPVNQFTGYLKLTDNVAIKCVDIVKEAQSANPMVVVNAANIHLKHGGGVAGAL         1053
AY278488.2          -PEPTPEEPVNQFTGYLKLTDNVAIKCVDIVKEAQSANPMVIVNAANIHLKHGGGVAGAL         1053
AY485277.1          -PEPTPEEPVNQFTGYLKLTDNVAIKCVDIVKEAQSANPMVIVNAANIHLKHGGGVAGAL         1053
AP006560.1          -PEPTPEEPVNQFTGYLKLTDNVAIKCVDIVKEAQSANPMVIVNAANIHLKHGGGVAGAL         1053
AP006557.1          -PEPTPEEPVNQFTGYLKLTDNVAIKCVDIVKEAQSANPMVIVNAANIHLKHGGGVAGAL         1053
AY274119            -PEPTPEEPVNQFTGYLKLTDNVAIKCVDIVKEAQSANPMVIVNAANIHLKHGGGVAGAL         1053
AY572038.1          -PEPTPEEPVNQFTGYLKLTDNVAIKCVDIVKEAQSANPMVIVNAANIHLKHGGGVAGAL         1053
AY572034.1          -PEPTPEEPVNQFTGYLKLTDNVAIKCVDIVKEAQSANPMVIVNAANIHLKHGGGVAGAL         1053
FJ588686.1          -PEPTSEEPVNQFTGYLKLTDNVAIKCVDIVKEAQNANPTVIVNAANIHLKHGGGVAGAL         1053
KY417145.1          -PELTPEEPVNQFTGYLKLTDNVAIKCVDIVKEAQSANPMVIVNAANIHLKHGGGVAGAL         1053
KY417144.1          -PELTPEEPVNQFTGYLKLTDNVAIKCVDIVKEAQNANPTVIVNAANIHLKHGGGVAGAL         1053
KY417147.1          -PELTPEEPVNQFTGYLKLTDNVAIKCVDIVKEAQNANPTVIVNAANIHLKHGGGVAGAL         1053
KY417148.1          -PELTPEEPVNQFTGYLKLTDNVAIKCVDIVKEAQNANPTVIVNAANIHLKHGGGVAGAL         1053
KY417143.1          -PELTPEEPVNQFTGYLKLTDNVAIKCVDIVKEAQNANPTVIVNAANIHLKHGGGVAGAL         1053
KT444582.1          -PELTLEEPVNQFTGYLKLTDNVAIKCVDIVKEAQNANPTVIVNAANIHLKHGGGVAGAL         1053
KC881005.1          -SEPTPEEPVNQFTGYLKLTDNVAIKCVDIVKEAQNANPTVIVNAANIHLKHGGGVAGAL         1053
KC881006.1          -PELTPEEPVNQFTGYLKLTDNVAIKCVDIVKEAQNANPTVIVNAANIHLKHGGGVAGAL         1053
KF367457.1          -PELTPEEPVNQFTGYLKLTDNVAIKCVDIVKEAQNANPTVIVNAANIHLKHGGGVAGAL         1053
KY417152.1          -PELTPEEPVNQFTGYLKLTDNVAIKCVDIVKEAQNANPTVIVNAANIHLKHGGGVAGAL         1053
KY417146.1          -PELTPEEPVNQFTGYLKLTDNVAIKCVDIVKEAQNANPTVIVNAANIHLKHGGGVAGAL         1053
KY417151.1          -PELTPEEPVNQFTGYLKLTDNVAIKCVDIVKEAQNANPTVIVNAANIHLKHGGGVAGAL         1053
KY417142.1          -PELTPEEPVNQFTGYLKLTDNVAIKCVDIVKEAQNANPTVIVNAANIHLKHGGGVAGAL         1053
                              :.* .* ****** **  ***.*: ..:* ::***** :******** **
```

```
MW532698.1      NKATNNAMQIESDDYIARNGPLNVGGSCLLNGHNLAKNCLHVVGPNLNKGEDIQLLKVAY    1127
MT040336.1      NKATNNAMQIESDDYIARNGPLNVGGSCLLNGHNLAKNCLHVVGPNLNKGEDIQLLKVAY    1127
MT040335.1      NKATNNAMQIESDDYIARNGPLNVGGSCLLNGHNLAKNCLHVVGPNLNKGEDIQLLKVAY    1127
MT040334.1      NKATNNAMQIESDDYIARNGPLNVGGSCLLNGHNLAKNCLHVVGPNLNKGEDIQLLKVAY    1127
MT040333.1      NKATNNAMQIESDDYIARNGPLNVGGSCLLNGHNLAKNCLHVVGPNLNKGEDIQLLKVAY    1127
MN996532.2      NKATNNAMQVESDHYIATNGPLKVGGGCVLSGHNLAKNCLHVVGPNVNRGEDIQLLKSAY    1134
MN988713.1      NKATNNAMQVESDDYIATNGPLKVGGSCVLSGHNLAKHCLHVVGPNVNKGEDIQLLKSAY    1135
MT093571.1      NKATNNAMQVESDDYIATNGPLKVGGSCVLSGHNLAKHCLHVVGPNVNKGEDIQLLKSAY    1135
MN996529.1      NKATNNAMQVESDDYIATNGPLKVGGSCVLSGHNLAKHCLHVVGPNVNKGEDIQLLKSAY    1135
MT072688.1      NKATNNAMQVESDDYIATNGPLKVGGSCVLSGHNLAKHCLHVVGPNVNKGEDIQLLKSAY    1135
NC_045512.2     NKATNNAMQVESDDYIATNGPLKVGGSCVLSGHNLAKHCLHVVGPNVNKGEDIQLLKSAY    1135
MN994467.1      NKATNNAMQVESDDYIATNGPLKVGGSCVLSGHNLAKHCLHVVGPNVNKGEDIQLLKSAY    1135
MG772933.1      NKATNNAMQVESDKYITTNGPLIVGGGCVLSGHNLAKNCLHVVGPNVNRGEDIQLLKNAY    1130
MG772934.1      NKATNNAMQLNLISNITTNGPLIVGGSCVLSGHNLAKNCLHVVGPNVNRGEDIQLLKNAY    1108
NC_014470.1     DKATGGSMQKESNDYISTNGPLRVGGSCLLSGHNLAKHCLHVVGPNKNAGEDIKLLDAAY    1096
GQ153542.1      NKATNGAMQNESDEYIRQNGPLTVGGSCLLSGHNLAEKCLHVVGPNLNAGEDVQLLKRAY    1112
DQ022305        NKATNGAMQNESDEYIRQNGPLTVGGSCLLSGHNLAEKCLHVVGPNLNAGEDVQLLKRAY    1108
KF569996.1      NKATNGAMQKESDDYIKQNGPLRVGGSCLLSGHNLANKCLHVVGPNLNAGEDIQLLKAAY    1114
KP886809.1      NKATNGAMQRESDDYIKLNGPLTVGGSCLLSGHNLAKKCLHVVGPNLNAGEDIQLLKAAY    1113
AY278488.2      NKATNGAMQKESDDYIKLNGPLTVGGSCLLSGHNLAKKCLHVVGPNLNAGEDIQLLKAAY    1113
AY485277.1      NKATNGAMQKESDDYIKLNGPLTVGGSCLLSGHNLAKKCLHVVGPNLNAGEDIQLLKAAY    1113
AP006560.1      NKATNGAMQKESDDYIKLNGPLTVGGSCLLSGHNLAKKCLHVVGPNLNAGEDIQLLKAAY    1113
AP006557.1      NKATNGAMQKESDDYIKLNGPLTVGGSCLLSGHNLAKKCLHVVGPNLNAGEDIQLLKAAY    1113
AY274119        NKATNGAMQKESDDYIKLNGPLTVGGSCLLSGHNLAKKCLHVVGPNLNAGEDIQLLKAAY    1113
AY572038.1      NKATNGAMQKESDDYIKLNGPLTVGGSCLLSGHNLAKKCLHVVGPNLNAGEDIRLFKAAY    1113
AY572034.1      NKATNGAMQKESDDYIKLNGPLTVGGSCLLSGHNLAKKCLHVVGPNLNAGEDIRLLKAAY    1113
FJ588686.1      NKATNGAMQKESDDYIKLNGPLTVGGSCLLSGHNLAKKCLHVVGPNLNAGEDIQLLKAAY    1113
KY417145.1      NKATNGAMQKESDDYIKLNGPLTVGGSCLLSGYNLAKKCLHVVGPNLNAGEDIQLLKAAY    1113
KY417144.1      NKATNGAMQKESDDYIKLNGPLTVGGSCLLSGHNLAKKCLHVVGPNLNAGEDIQLLKAAY    1113
KY417147.1      NKATNGAMQKESDDYIKLNGPLVVGGSCLLSGHNLAKKCLHVVGPNLNAGEDIQLLKAAY    1113
KY417148.1      NKATNGAMQKESDDYIKLNGPLTVGGSCLLSGHNLAKKCLHVVGPNLNAGEDIQLLKAAY    1113
KY417143.1      NKATNGAMQKESDDYIKLNGPLTVGGSCLLSGHNLAKKCLHVVGPNLNAGEDIQLLKAAY    1113
KT444582.1      NKATNGAMQKESDDYIKLNGPLTVGGSCLLSGHNLAKKCLHVVGPNLNAGEDIQLLKAAY    1113
KC881005.1      NKATNGAMQKESDDYIKLNGPLAVGGSCLLSGHNLAKKCLHVVGPNLNAGEDIQLLKAAY    1113
KC881006.1      NKATNGAMQKESDDYIKLNGPLVVGGSCLLSGHNLAKKCLHVVGPNLNAGEDIQLLKAAY    1113
KF367457.1      NKATNGAMQKESDDYIKLNGPLVVGGSCLLSGHNLAKKCLHVVGPNLNAGEDIQLLKAAY    1113
KY417152.1      NKATNGAMQKESDDYIKLNGPLVVGGSCLLSGHNLAKKCLHVVGPNLNAGEDIQLLKAAY    1113
KY417146.1      NKATNGAMQKESDDYIKLNGPLTVGGSCLLSGHNLAKKCLHVVGPNLNAGEDIQLLKAAY    1113
KY417151.1      NKATNGAMQKESDDYIKLNGPLTVGGSCLLSGHNLAKKCLHVVGPNLNAGEDIQLLKAAY    1113
KY417142.1      NKATNGAMQKESDDYIKLNGPLTVGGSCLLSGHNLAKKCLHVVGPNLNAGEDIQLLKAAY    1113
                :***..:** :    *  **** ***.*:*.*:***::******** * ***::*:. **
```

```
MW532698.1      ENFNHHEKLLAPLLSAGIFGAQPIQSLKVCIETVRTQVFLAVFDKDLYEELVASFLEMKS      1187
MT040336.1      ENFNHHEKLLAPLLSAGIFGAQPIQSLKVCIETVRTQVFLAVFDKDLYEELVASFLEMKS      1187
MT040335.1      ENFNHHEKLLAPLLSAGIFGAQPIQSLKVCIETVRTQVFLAVFDKDLYEELVASFLEMKS      1187
MT040334.1      ENFNHHEKLLAPLLSAGIFGAQPIQSLKVCIETVRTQVFLAVFDKDLYEELVASFLEMKS      1187
MT040333.1      ENFNHHEKLLAPLLSAGIFGAQPIQSLKVCIETVRTQVFLAVFDKDLYEELVASFLEMKS      1187
MN996532.2      ENFNQHDVLLAPLLSAGIFGADPVHSLRVCVDTVRTNVYLAVFDKNLYDKLVSSFLEMKS      1194
MN988713.1      ENFNQHEVLLAPLLSAGIFGADPIHSLRVCVDTVRTNVYLAVFDKNLYDKLVSSFLEMKS      1195
MT093571.1      ENFNQHEVLLAPLLSAGIFGADPIHSLRVCVDTVRTNVYLAVFDKNLYDKLVSSFLEMKS      1195
MN996529.1      ENFNQHEVLLAPLLSAGIFGADPIHSLRVCVDTVRTNVYLAVFDKNLYDKLVSSFLEMKS      1195
MT072688.1      ENFNQHEVLLAPLLSAGIFGADPIHSLRVCVDTVRTNVYLAVFDKNLYDKLVSSFLEMKS      1195
NC_045512.2     ENFNQHEVLLAPLLSAGIFGADPIHSLRVCVDTVRTNVYLAVFDKNLYDKLVSSFLEMKS      1195
MN994467.1      ENFNQHEVLLAPLLSAGIFGADPIHSLRVCVDTVRTNVYLAVFDKNLYDKLVSSFLEMKS      1195
MG772933.1      ENFNQHEILLAPLLSAGIFGADPVHSLRVCVETVHTNVYLVVFDKNLYDKLVSSFLEMKS      1190
MG772934.1      ENFNQHEILLAPLLSAGIFGVDPVHSLRVCVETVHTNVYLVVFDKNLYDKLVSSFLEMKS      1168
NC_014470.1     ENFNAYEVVLSPLLSAGIFGVSPIQSLETCKRVVRNTVYIVVNDSVVFDQLLAKTPGKTN      1156
GQ153542.1      ENFNSQDVLLAPLLSAGIFGAKPLQSLKMCVETVRAQVYLAVNDKSLYDQIILDYLDSLK      1172
DQ022305        ENFNSQDVLLAPLLSAGIFGAKPLQSLKMCVEIVRTQVYLAVNDKSLYDQIVLDYLDSLK      1168
KF569996.1      ENFNSQEILLAPLLSAGIFGAKPLDSLKVCVQTVRTHVYIAVNDQKLYDQVVMDYLDSLK      1174
KP886809.1      ENFNSQDTLLAPLLSAGIFGAKPLQSLQVCVQTVRTQVYIAVNDRALYEQVVMDYLDSLK      1173
AY278488.2      ENFNSQDILLAPLLSAGIFGAKPLQSLQVCVQTVRTQVYIAVNDKALYEQVVMDYLDNLK      1173
AY485277.1      ENFNSQDILLAPLLSAGIFGAKPLQSLQVCVQTVRTQVYIAVNDKALYEQVVMDYLDNLK      1173
AP006560.1      ENFNSQDILLAPLLSAGIFGAKPLQSLQVCVQTVRTQVYIAVNDKALYEQVVMDYLDNLK      1173
AP006557.1      ENFNSQDILLAPLLSAGIFGAKPLQSLQVCVQTVRTQVYIAVNDKALYEQVVMDYLDNLK      1173
AY274119        ENFNSQDILLAPLLSAGIFGAKPLQSLQVCVQTVRTQVYIAVNDKALYEQVVMDYLDNLK      1173
AY572038.1      ENFNSQDTLLAPLLSAGIFGAKPLQSLQVCVQTVCTQVYIAVNDKALYEQVVMDYLDNLK      1173
AY572034.1      ENFNSQDTLLAPLLSAGIFGAKPLQSLQVCVQTVRTQVYIAVNDKALYEQVVMDYLDNLK      1173
FJ588686.1      ENFNSQDTLLAPLLSAGIFGAKPLQSLEVCVQTVRTQVYIAVNDKALYEQVVMDYLDSLK      1173
KY417145.1      ENFNSQDTLLAPLLSAGIFGAKPLHSLQVCVQTVRTQVYIAVNDKALYEQVVMDYLDSLK      1173
KY417144.1      ENFNSQDTLLAPLLSAGIFGAKPLHSLQVCVQTVRTQVYIAVNDKALYEQVVMDYLDSLK      1173
KY417147.1      ENFNSQDTLLAPLLSAGIFGAKPLQSLQVCVQTVRTQVYIAVNDKALYEQVVMDYLDSLK      1173
KY417148.1      ENFNSQDTLLAPLLSAGIFGAKPLHSLQVCVQTVRTQVYIAVNDKALYEQVVMDYLDSLK      1173
KY417143.1      ENFNSQDTLLAPLLSAGIFGAKPLHSLQVCVQTVRTQVYIAVNDKALYEQVVMDYLDSLK      1173
KT444582.1      ENFNSQDTLLAPLLSAGIFGAKPLHSLQVCVQTVRTQVYIAVNDKALYEQVVMDYLDSLK      1173
KC881005.1      ENFNSQDTLLAPLLSAGIFGAKPLQSLQVCVQTVRTQVYIAVNDKALYEQVVMDYLDSLK      1173
KC881006.1      ENFNSQDTLLAPLLSAGIFGAKPLQSLQVCVQTVRTQVYIAVNDKALYEQVVMDYLDSLK      1173
KF367457.1      ENFNSQDTLLAPLLSAGIFGAKPLQSLQVCVQTVRTQVYIAVNDKALYEQVVMDYLDSLK      1173
KY417152.1      ENFNSQDTLLAPLLSAGIFGAKPLQSLQVCVQTVRTQVYIAVNDKALYEQVVMDYLDSLK      1173
KY417146.1      ENFNSQDTLLAPLLSAGIFGAKPLHSLQVCVQTVRTQVYIAVNDKALYEQVVMDYLDSLK      1173
KY417151.1      ENFNSQDTLLAPLLSAGIFGAKPLQSLQVCVQTVRTQVYIAVNDKALYEQVVMDYLDSLK      1173
KY417142.1      ENFNSQDTLLAPLLSAGIFGAKPLQSLQVCVQTVRTQVYIAVNDKALYEQVVMDYLDSLK      1173
                ****  : :*:*********..*:.**. *   *   *::.* *  :::::: .     .
```

```
MW532698.1      ETKVQDHFDVV-------ETK--VEITPEESASSEKPTKEEPKKVKPCIEEVTTTLEETK     1238
MT040336.1      ETKVQDHFDVV-------ETK--VEITPEESASSEKPTKEEPKKVKPCIEEVTTTLEETK     1238
MT040335.1      ETKVQDHFDVV-------ETK--VEITPEESASSEKPTKEEPKKVKPCIEEVTTTLEETK     1238
MT040334.1      ETKVQDHFDVV-------ETK--VEITPEESASSEKPTKEEPKKVKPCIEEVTTTLEETK     1238
MT040333.1      ETKVQDHFDVV-------ETK--VEITPEESASSEKPTKEEPKKVKPCIEEVTTTLEETK     1238
MN996532.2      EKQVEQKTAEI-------PKEEVKSSITESKLSVEQRQQV-DKKIKACVEEVTTTLEETK     1246
MN988713.1      EKQVEQKIAEI-------PKEEVKPFITESKPSVEQRKQD-DKKIKACVEEVTTTLEETK     1247
MT093571.1      EKQVEQKIAEI-------PKEEVKPFITESKPSVEQRKQD-DKKIKACVEEVTTTLEETK     1247
MN996529.1      EKQVEQKIAEI-------PKEEVKPFITESKPSVEQRKQD-DKKIKACVEEVTTTLEETK     1247
MT072688.1      EKQVEQKIAEI-------PKEEVKPFITESKPSVEQRKQD-DKKIKACVEEVTTTLEETK     1247
NC_045512.2     EKQVEQKIAEI-------PKEEVKPFITESKPSVEQRKQD-DKKIKACVEEVTTTLEETK     1247
MN994467.1      EKQVEQKIAEI-------PKEEVKPFITESKPSVEQRKQD-DKKIKACVEEVTTTLEETK     1247
MG772933.1      EKQVEQKVAEN-------PKEEVKPFFTENKPSVEQRQQAEEKKIKASIEEVTTTLEETK     1243
MG772934.1      GKQVEQKVAEN-------PKEEVKPFFTENKPSVEQRQQAEEKKIKASIEEVTTTLEETK     1221
NC_014470.1     ERPVVESSEICEEV-NQKPVVEFSE-TKELHEETNQKLKSSEEPVKTRIEELNTTVDEAK     1214
GQ153542.1      --PKVESPNKEEEPKLEEPKA---------VQPVVEKPVDVKPKIKACIDEVTTTLEETK     1221
DQ022305        --PKVESPNKEEEPKLEEPKA---------VQPVAEKPVDVKPKIKACIDEVTTTLEETK     1217
KF569996.1      --PTVETPKEEQ-PKIEDSNVK--------EEPTTQKPVDVKPKIKACIEEVTTTLEETK     1223
KP886809.1      --PRVEAPKQEEPPKTERPKVE--------EKSVVQKPVDVKPKIKACINEVTTTLEETK     1223
AY278488.2      --PRVEAPKQEEPPNTEDSKTE--------EKSVVQKPVDVKPKIKACIDEVTTTLEETK     1223
AY485277.1      --PRVEAPKQEEPPNTEDSKTE--------EKSVVQKPVDVKPKIKACIDEVTTTLEETK     1223
AP006560.1      --PRVEAPKQEEPPNTEDSKTE--------EKSVVQKPVDVKPKIKACIDEVTTTLEETK     1223
AP006557.1      --PRVEAPKQEEPPNTEDSKTE--------EKSVVQKPVDVKPKIKACIDEVTTTLEETK     1223
AY274119        --PRVEAPKQEEPPNTEDSKTE--------EKSVVQKPVDVKPKIKACIDEVTTTLEETK     1223
AY572038.1      --PRVEAPKQEEPPNTEDSKTE--------EKSVEQKPVDVKPKIKACIDEVTTTLEETK     1223
AY572034.1      --PRVEAPKQEEPPNTEDSKTE--------EKSVVQKPVDVKPKIKACIDEVTTTLEETK     1223
FJ588686.1      --PRVEAPKQEEPPKTEHPKIE--------EKSVVQKPVDVKPKIKACIDEVTTTLEETK     1223
KY417145.1      --PRVEAPKQEEPPKTEDPKIE--------EKSVVQKPVDVKPKIKAFIDEVTTTLEETK     1223
KY417144.1      --PRVEAPKQEEPPKTEDPKIE--------EKSVVQKPVDVKPKIKACIDEVTTTLEETK     1223
KY417147.1      --PRVEAPKQEEPPRTEDPKIE--------EKSVVQKPIDVKPKIKACIDEVTTTLEETK     1223
KY417148.1      --PRVEAPKQEEPPKTEDPKIE--------EKSVVQKPVDVKPKIKACIDEVTTTLEETK     1223
KY417143.1      --PRVEAPKQEEPPKTEDPKIE--------EKSVVQKPVDVKPKIKACIDEVTTTLEETK     1223
KT444582.1      --PRVEAPKQEEPPKTEDPKIE--------EKSVVQKPVDVKPKIKACIDEVTTTLEETK     1223
KC881005.1      --PRVEAPKQEEPPRTEDPKIE--------EKSVVQKPIDVKPKIKACIDEVTTTLEETK     1223
KC881006.1      --PRVEAPKQEEPPRTEDPKIE--------EKSVVQKPIDVKPKIKACIDEVTTTLEETK     1223
KF367457.1      --PRVEAPKQEEPPRTEDPKIE--------EKSVVQKPIDVKPKIKACIDEVTTTLEETK     1223
KY417152.1      --PRVEAPKQEEPPRTEDPKIE--------EKSVVQKPIDVKPKIKACIDEVTTTLEETK     1223
KY417146.1      --PRVEAPKQEEPPKTEDPKIE--------EKSVVQKPVDVKPKIKACIDEVTTTLEETK     1223
KY417151.1      --PRVEAPKQEEPPKTEDPEIE--------EKFVVQKPVDVKPKIKACIDEVTTTLEETK     1223
KY417142.1      --PRVEAPKQEEPPKTEDPEIE--------EKFVVQKPVDVKPKIKACIDEVTTTLEETK     1223
                     :                             :        :*  ::*:.**::*:*
```

```
MW532698.1      FLTENLLLYADINGNLYPDSTSLVENVDVTFLKKDAPYIVGDIITSGNLTTVVIPTKKAG     1298
MT040336.1      FLTENLLLYADINGNLYPDSTSLVENVDVTFLKKDAPYIVGDIITSGNLTTVVIPTKKAG     1298
MT040335.1      FLTENLLLYADINGNLYPDSTSLVENVDVTFLKKDAPYIVGDIITSGNLTTVVIPTKKAG     1298
MT040334.1      FLTENLLLYADINGNLYPDSTSLVENVDVTFLKKDAPYIVGDIITSGNLTTVVIPTKKAG     1298
MT040333.1      FLTENLLLYADINGNLYPDSTSLVENVDVTFLKKDAPYIVGDIITSGNLTTVVIPTKKAG     1298
MN996532.2      FLTENLLLYIDINGNLHPDSATLVNDIDITFLKKDAPYIVGDVVQEGVLTAVVIPTKKAG     1306
MN988713.1      FLTENLLLYIDINGNLHPDSATLVSDIDITFLKKDAPYIVGDVVQEGVLTAVVIPTKKAG     1307
MT093571.1      FLTENLLLYIDINGNLHPDSATLVSDIDITFLKKDAPYIVGDVVQEGVLTAVVIPTKKAG     1307
MN996529.1      FLTENLLLYIDINGNLHPDSATLVSDIDITFLKKDAPYIVGDVVQEGVLTAVVIPTKKAG     1307
MT072688.1      FLTENLLLYIDINGNLHPDSATLVSDIDITFLKKDAPYIVGDVVQEGVLTAVVIPTKKAG     1307
NC_045512.2     FLTENLLLYIDINGNLHPDSATLVSDIDITFLKKDAPYIVGDVVQEGVLTAVVIPTKKAG     1307
MN994467.1      FLTENLLLYIDINGNLHPDSATLVSDIDITFLKKDAPYIVGDVVQEGVLTAVVIPTKKAG     1307
MG772933.1      FLTENLLLYIDINGNLHPDSATLVKDIDTTFLKKDVPYIVGDVIKEGALTAVVIPTKKAG     1303
MG772934.1      FLTENLLLYIDINGNLHPDSATLVKDIDITFLKKDVPYIVGDVIKEGALTAVVIPTKKAG     1281
NC_014470.1     FLTTKLLLYADVNGNLSEDSKVLI-GNDGASFKKGAPYIVGDIISEGELTCVVLPTKAVG     1273
GQ153542.1      FLTNKLLLFADINGKLYQDSQNMLRGEDMSFLEKDAPYIVGDVITSGDITCVIIPAKKAG     1281
DQ022305        FLTNKLLLFADINGKLYQDSQNMLRGEDMSFLEKDAPYIVGDVITSGDITCVIIPAKKSG     1277
KF569996.1      FLTNKLLLFADINGKLYQDSQNMLRGEDVSFLERDAPYMVGDVINSGDITCVVIPSKKAG     1283
KP886809.1      FLTNKLLLFADINGKLYRDSQNMLRGEDMSFLEKDAPYMVGDVITSGDITCVVIPSKKAG     1283
AY278488.2      FLTNKLLLFADINGKLYHDSQNMLRGEDMSFLEKDAPYMVGDVITSGDITCVVIPSKKAG     1283
AY485277.1      FLTNKLLLFADINGKLYHDSQNMLRGEDMSFLEKDAPYMVGDVITSGDITCVVIPSKKAG     1283
AP006560.1      FLTNKLLLFADINGKLYHDSQNMLRGEDMSFLEKDAPYMVGDVITSGDITCVVIPSKKAG     1283
AP006557.1      FLTNKLLLFADINGKLYHDSQNMLRGEDMSFLEKDAPYMVGDVITSGDITCVVIPSKKAG     1283
AY274119        FLTNKLLLFADINGKLYHDSQNMLRGEDMSFLEKDAPYMVGDVITSGDITCVVIPSKKAG     1283
AY572038.1      FLTNKLLLFADINGKLYHDSQNMLRGEDMSFLEKDAPYMVGDVITSGDITCVVIPSKKTG     1283
AY572034.1      FLTNKLLLFADINGKLYHDSQNMLRGEDMSFLEKDAPYMVGDVITSGDITCVVIPSKKTG     1283
FJ588686.1      FLTNKLLLFADINGKLYHDSQNMLRGEDMSFLEKDAPYMVGDVITSGDITCVVIPSKKAG     1283
KY417145.1      FLTNKLLLFADINGKLYHDSQNMLRGEDMSFLEKDAPYMVGDVITSGDITCVVIPSKKAG     1283
KY417144.1      FLTNKLLLFADINGKLYHDSQNMLRGEDMSFLEKDAPYVVGDVITSGDITCVVIPSKKAG     1283
KY417147.1      FLTNKLLLFADINGKLYHDSHNMLRGEDMSFLEKDAPYVVGDVITSGDITCVVIPSKKAG     1283
KY417148.1      FLTNKLLLFADINGKLYHDSHNMLRGEDMSFLEKDAPYMVGDVITSGDITCVVIPSKKAG     1283
KY417143.1      FLTNKLLLFADINGKLYHDSHNMLRGEDMSFLEKDAPYMVGDVITSGDITCVVIPSKKAG     1283
KT444582.1      FLTNKLLLFADINGKLYHDSHNMLRGEDMSFLEKDAPYMVGDVITSGDITCVVIPSKKAG     1283
KC881005.1      FLTNKLLLFADINGKLYHDSHNMLRGEDMSFLEKDAPYMVGDVITSGDITCVVIPSKKAG     1283
KC881006.1      FLTNKLLLFADINGKLYHDSHNMLRGEDMSFLEKDAPYVVGDVITSGDITCVVIPSKKAG     1283
KF367457.1      FLTNKLLLFADINGKLYHDSHNMLRGEDMSFLEKDAPYVVGDVITSGDITCVVIPSKKAG     1283
KY417152.1      FLTNKLLLFADINGKLYHDSHNMLRGEDMSFLEKDAPYVVGDVITSGDITCVVIPSKKAG     1283
KY417146.1      FLTNKLLLFADINGKLYHDSQNMLRGEDMSFLEKDAPYMVGDVITSGDITCVVIPSKKAG     1283
KY417151.1      FLTNKLLLFADINGKLYHDSQNMLRGEDMSFLEKDAPYMVGDVITSGDITCVVIPSKKAG     1283
KY417142.1      FLTNKLLLFADINGKLYHDSQNMLRGEDMSFLEKDAPYMVGDVITSGDITCVVIPSKKAG     1283
                *** :***: *:**:*  **  :: . * : :::..**:***:: .* :* *::*:*  *
```

```
MW532698.1      GTTEMLAKALRKVPTDHYITTYPGQGCVGYTIEEAKTALKKSKSAYYVLPSIIPNKKEEI    1358
MT040336.1      GTTEMLAKALRKVPTDHYITTYPGQGCVGYTIEEAKTALKKSKSAYYVLPSIIPNKKEEI    1358
MT040335.1      GTTEMLAKALRKVPTDHYITTYPGQGCVGYTIEEAKTALKKSKSAYYVLPSIIPNKKEEI    1358
MT040334.1      GTTEMLAKALRKVPTDHYITTYPGQGCVGYTIEEAKTALKKSKSAYYVLPSIIPNKKEEI    1358
MT040333.1      GTTEMLAKALRKVPTDHYITTYPGQGCVGYTIEEAKTALKKSKSAYYVLPSIIPNKKEEI    1358
MN996532.2      GTTEMLTKALRKVPTDNYITTYPGQGLNGYTVEEARTVLKKCKSAFYILPSIISNEKQEI    1366
MN988713.1      GTTEMLAKALRKVPTDNYITTYPGQGLNGYTVEEAKTVLKKCKSAFYILPSIISNEKQEI    1367
MT093571.1      GTTEMLAKALRKVPTDNYITTYPGQGLNGYTVEEAKTVLKKCKSAFYILPSIISNEKQEI    1367
MN996529.1      GTTEMLAKALRKVPTDNYITTYPGQGLNGYTVEEAKTVLKKCKSAFYILPSIISNEKQEI    1367
MT072688.1      GTTEMLAKALRKVPTDNYITTYPGQGLNGYTVEEAKTVLKKCKSAFYILPSIISNEKQEI    1367
NC_045512.2     GTTEMLAKALRKVPTDNYITTYPGQGLNGYTVEEAKTVLKKCKSAFYILPSIISNEKQEI    1367
MN994467.1      GTTEMLAKALRKVPTDNYITTYPGQGLNGYTVEEAKTVLKKCKSAFYILPSIISNEKQEI    1367
MG772933.1      GTTEMLAKALRKVPTDNYITTYPGQGLNGYTVEEAKTVLKKCKSAFYILPSIISNAKQEI    1363
MG772934.1      GTTEMLAKALRKVPTDNHITTYPGQGLNGYTVEEAKTVLKKCKSVFYILPSILPNEKQEI    1341
NC_014470.1     GTTHMLTRALKNVPSDTYLTTYPGQGVSGYTLDEAKAALKKSRSVFYILPSANVNAKEEV    1333
GQ153542.1      GTTDMLARALKKVPVAEYITTYPGQGCAGYTLEEAKTALKKCKSAFYVLPSETPNEKEEV    1341
DQ022305        GTTEMLARALKEVPVAEYITTYPGQGCAGYTLEEAKTALKKCKSAFYVLPSETPNEKEEV    1337
KF569996.1      GTTEMLARALKKVPIDEYITTYPGQGCAGYTLDEARTALKKCKSALYVLPSETPNAKEEI    1343
KP886809.1      GTTEMLSRALKKVPVGEYITTYPGQGCAGYTLEEAKTILKKCKSEFYVLPSETPNAKEEV    1343
AY278488.2      GTTEMLSRALKKVPVDEYITTYPGQGCAGYTLEEAKTALKKCKSAFYVLPSEAPNAKEEI    1343
AY485277.1      GTTEMLSRALKKVPVDEYITTYPGQGCAGYTLEEAKTALKKCKSAFYVLPSEAPNAKEEI    1343
AP006560.1      GTTEMLSRALKKVPVDEYITTYPGQGCAGYTLEEAKTALKKCKSAFYVLPSEAPNAKEEI    1343
AP006557.1      GTTEMLSRALKKVPVDEYITTYPGQGCAGYTLEEAKTALKKCKSAFYVLPSEAPNAKEEI    1343
AY274119        GTTEMLSRALKKVPVDEYITTYPGQGCAGYTLEEAKTALKKCKSAFYVLPSEAPNAKEEI    1343
AY572038.1      GTTEMLSRALKKVPVAEYITTYPGQGCAGYTLEEAKTALKKCKSAFYVLPSEAPNAKEEI    1343
AY572034.1      GTTEMLSRALKKVPVDEYITTYPGQGCAGYTLEEAKTALKKCKSAFYVLPSEAPNAKEEI    1343
FJ588686.1      GTTEMLSRALKKVPVDEYITTYPGQGCAGYTLEEAKTALKKCKSAFYVLPSETPNAKEEI    1343
KY417145.1      GTTEMLSRALKKVPVDEYITTYPGQGCAGYTLEEAKTALKKCKSALYVLPSETPNAKEEI    1343
KY417144.1      GTTEMLSRALKKVPVDEYITTYPGQGCAGYTLEEAKTALKKCKSALYVLPSETPNAKEEI    1343
KY417147.1      GTTEMLSRALKKVPVDEYITTYPGQGCAGYTLEEAKTALKKCKSAFYVLPSETPNAKEEI    1343
KY417148.1      GTTEMLSRALKKVPVDEYITTYPGQGCAGYTLEEAKTALKKCKSAFYVLPSETPNAKEEI    1343
KY417143.1      GTTEMLSRALKKVPVDEYITTYPGQGCAGYTLEEAKTALKKCKSAFYVLPSETPNAKEEI    1343
KT444582.1      GTTEMLSRALKKVPVDEYITTYPGQGCAGYTLEEAKTALKKCKSAFYVLPSETPNAKEEI    1343
KC881005.1      GTTEMLSRALKKVPVDEYITTYPGQGCAGYTLEEAKTALKKCKSAFYVLPSETPNAKEEI    1343
KC881006.1      GTTEMLSRALKKVPVDEYITTYPGQGCAGYTLEEAKTALKKCKSAFYVLPSETPNAKEEI    1343
KF367457.1      GTTEMLSRALKKVPVDEYITTYPGQGCAGYTLEEAKTALKKCKSAFYVLPSETPNAKEEI    1343
KY417152.1      GTTEMLSRALKKVPVDEYITTYPGQGCAGYTLEEAKTALKKCKSAFYVLPSETPNAKEEI    1343
KY417146.1      GTTEMLSRALKKVPVDEYITTYPGQGCAGYTLEEAKTALRKCKSAFYVLPSETPNAKEEI    1343
KY417151.1      GTTEMLSRALKKVPVDEYITTYPGQGCAGYTLEEAKTALKKCKSAFYVLPSETPNAKEEI    1343
KY417142.1      GTTEMLSRALKKVPVDEYITTYPGQGCAGYTLEEAKTALKKCKSAFYVLPSETPNAKEEI    1343
                ***.**::**::**   ::*******  ***::**:: *:*.:*  *:***   * *:*:
```

```
MW532698.1      LGTVSWNLREMLTHAEETRKLMPICMDTKAIMSTVQRKYKGIKIQEGVVDYGVRFYFYTS    1418
MT040336.1      LGTVSWNLREMLTHAEETRKLMPICMDTKAIMSTVQRKYKGIKIQEGVVDYGVRFYFYTS    1418
MT040335.1      LGTVSWNLREMLTHAEETRKLMPICMDTKAIMSTVQRKYKGIKIQEGVVDYGVRFYFYTS    1418
MT040334.1      LGTVSWNLREMLTHAEETRKLMPICMDTKAIMSTVQRKYKGIKIQEGVVDYGVRFYFYTS    1418
MT040333.1      LGTVSWNLREMLTHAEETRKLMPICMDTKAIMSTVQRKYKGIKIQEGVVDYGVRFYFYTS    1418
MN996532.2      LGTVSWNLREMLAHAEETRKLMPVCMETKAIVSTIQRKYKGIKIQEGVVDYGARFYFYTS    1426
MN988713.1      LGTVSWNLREMLAHAEETRKLMPVCVETKAIVSTIQRKYKGIKIQEGVVDYGARFYFYTS    1427
MT093571.1      LGTVSWNLREMLAHAEETRKLMPVCVETKAIVSTIQRKYKGIKIQEGVVDYGARFYFYTS    1427
MN996529.1      LGTVSWNLREMLAHAEETRKLMPVCVETKAIVSTIQRKYKGIKIQEGVVDYGARFYFYTS    1427
MT072688.1      LGTVSWNLREMLAHAEETRKLMPVCVETKAIVSTIQRKYKGIKIQEGVVDYGARFYFYTS    1427
NC_045512.2     LGTVSWNLREMLAHAEETRKLMPVCVETKAIVSTIQRKYKGIKIQEGVVDYGARFYFYTS    1427
MN994467.1      LGTVSWNLREMLAHAEETRKLMPVCVETKAIVSTIQRKYKGIKIQEGVVDYGARFYFYTS    1427
MG772933.1      LGTVSWNLREMLAHAEETRKLMPVCMETKAIVSTIQRKYKGIKIQEGVVDYGARFYFYTS    1423
MG772934.1      LGTVSWNLREMLAHAEETRKLMPVCMETKAIVSTIQRKYKGIKIQEGVVDYGARFYFYTS    1401
NC_014470.1     LGTVAWNLREMLAHAEETRKVMPVCMDVRAIISTIQRKYKGIGIQEGLVDYKVRFYFYSS    1393
GQ153542.1      LGTVSWNLREMLAHAEETRKLMPICLDVRAIMATIQRKYKGIKVQEGIVDYGVRFFFYTS    1401
DQ022305        LGTVSWNLREMLAHAEETRKLMPICLDVRAIMATIQRKYKGIKVQEGIVDYGVRFFFYTS    1397
KF569996.1      LGTVSWNLREMLAHAEETRKLMPVCMDVRAIMATIQRKYKGIKIQEGIVDYGVRFFFYTS    1403
KP886809.1      LGTVSWNLREMLAHAEETRKLMPICMDIRAIMATIQRKYKGIKIQEGIVDYGVRFFFYTS    1403
AY278488.2      LGTVSWNLREMLAHAEETRKLMPICMDVRAIMATIQRKYKGIKIQEGIVDYGVRFFFYTS    1403
AY485277.1      LGTVSWNLREMLAHAEETRKLMPICMDVRAIMATIQRKYKGIKIQEGIVDYGVRFFFYTS    1403
AP006560.1      LGTVSWNLREMLAHAEETRKLMPICMDVRAIMATIQRKYKGIKIQEGIVDYGVRFFFYTS    1403
AP006557.1      LGTVSWNLREMLAHAEETRKLMPICMDVRAIMATIQRKYKGIKIQEGIVDYGVRFFFYTS    1403
AY274119        LGTVSWNLREMLAHAEETRKLMPICMDVRAIMATIQRKYKGIKIQEGIVDYGVRFFFYTS    1403
AY572038.1      LGTVSWNLREMLAHAEETRKLMPICMDVRAIMATIQRKYKGIKIQEGIVDYGVRFFFYTS    1403
AY572034.1      LGTVSWNLREMLAHAEETRKLMPICMDVRAIMATIQRKYKGIKIQEGIVDYGVRFFFYTS    1403
FJ588686.1      LGTVSWNLREMLAHAEETRKLMPICMDVRAIMATIQRKYKGIKIQEGIVDYGVRFFFYTS    1403
KY417145.1      LGTVSWNLREMLAHAEETRKLMPICMDVRAIMATIQRKYKGIKIQEGIVDYGVRFFFYTS    1403
KY417144.1      LGTVSWNLREMLAHAEETRKLMPICMDVRAIMATIQRKYKGIKIQEGIVDYGVRFFFYTS    1403
KY417147.1      LGTVSWNLREMLAHAEETRKLMPICMDVRAIMATIQRKYKGIKVQEGIVDYGVRFFFYTS    1403
KY417148.1      LGTVSWNLREMLAHAEETRKLMPICMDVRAIMATIQRKYKGIKIQEGIVDYGVRFFFYTS    1403
KY417143.1      LGTVSWNLREMLAHAEETRKLMPICMDVRAIMATIQRKYKGIKIQEGIVDYGVRFFFYTS    1403
KT444582.1      LGTVSWNLREMLAHAEETRKLMPICMDVRAIMATIQRKYKGIKIQEGIVDYGVRFFFYTS    1403
KC881005.1      LGTVSWNLREMLAHAEETRKLMPICMDVRAIMATIQRKYKGIKVQEGIVDYGVRFFFYTS    1403
KC881006.1      LGTVSWNLREMLAHAEETRKLMPICMDVRAIMATIQRKYKGIKVQEGIVDYGVRFFFYTS    1403
KF367457.1      LGTVSWNLREMLAHAEETRKLMPICMDVRAIMATIQRKYKGIKVQEGIVDYGVRFFFYTS    1403
KY417152.1      LGTVSWNLREMLAHAEETRKLMPICMDVRAIMATIQRKYKGIKVQEGIVDYGVRFFFYTS    1403
KY417146.1      LGTVSWNLREMLAHAEETRKLMPICMDVRAIMATIQRKYKGIKIQEGIVDYGVRFFFYTS    1403
KY417151.1      LGTVSWNLREMLAHAEETRKLMPICMDVRAIMATIQRKYKGIKIQEGIVDYGVRFFFYTS    1403
KY417142.1      LGTVSWNLREMLAHAEETRKLMPICMDVRAIMATIQRKYKGIKIQEGIVDYGVRFFFYTS    1403
                ****:*******:*******:**:*:: :**::*:******* :***:*** .**:**:*
```

```
MW532698.1        KTPVATLIATLNSLGETLVTMPLGYVTHGLNLEEAARYMRSLKVPATVSVSSPDAVTAYN         1478
MT040336.1        KTPVATLIATLNSLGETLVTMPLGYVTHGLNLEEAARYMRSLKVPATVSVSSPDAVTAYN         1478
MT040335.1        KTPVATLIATLNSLGETLVTMPLGYVTHGLNLEEAARYMRSLKVPATVSVSSPDAVTAYN         1478
MT040334.1        KTPVATLIATLNSLGETLVTMPLGYVTHGLNLEEAARYMRSLKVPATVSVSSPDAVTAYN         1478
MT040333.1        KTPVATLIATLNSLGETLVTMPLGYVTHGLNLEEAARYMRSLKVPATVSVSSPDAVTAYN         1478
MN996532.2        KTTVASLINTLNDLNETLVTMPLGYVTHGLNLEEAARYMRSLKVPATVSVSSPDAVTAYN         1486
MN988713.1        KTTVASLINTLNDLNETLVTMPLGYVTHGLNLEEAARYMRSLKVPATVSVSSPDAVTAYN         1487
MT093571.1        KTTVASLINTLNDLNETLVTMPLGYVTHGLNLEEAARYMRSLKVPATVSVSSPDAVTAYN         1487
MN996529.1        KTTVASLINTLNDLNETLVTMPLGYVTHGLNLEEAARYMRSLKVPATVSVSSPDAVTAYN         1487
MT072688.1        KTTVASLINTLNDLNETLVTMPLGYVTHGLNLEEAARYMRSLKVPATVSVSSPDAVTAYN         1487
NC_045512.2       KTTVASLINTLNDLNETLVTMPLGYVTHGLNLEEAARYMRSLKVPATVSVSSPDAVTAYN         1487
MN994467.1        KTTVASLINTLNDLNETLVTMPLGYVTHGLNLEEAARYMRSLKVPATVSVSSPDAVTAYN         1487
MG772933.1        KTTVASLINTLNNLNETLVTMPLGYVTHGLNLEEAARYMRSLKVPATVSVSSPDAVTAYN         1483
MG772934.1        KTTVASLINTLNNLNETLVTMPLGYVTHGLNLEEAARYMRSLKVPATVSVSSPDAVTAYN         1461
NC_014470.1       KTPIARVISNLNSLGEPLITMPLGYVTHGLNLEESARYMRSVKVPVVVSVSSPDAVTSYN         1453
GQ153542.1        KEPVASIITKLNSLNEPLVTMPIGYVTHGLNLEETARCMRSLKAPAVVSVSSPDAVTAYN         1461
DQ022305          KEPVASIITKLNSLNEPLVTMPIGYVTHGLNLEEAARCMRSLKAPAVVSVSSPDAVTAYN         1457
KF569996.1        KEPVASIITKLNSLNEPLVTMPIGYVTHGFNLEEAARCMRSLKAPAVVSVSSPDAVTTYN         1463
KP886809.1        KEPVASIITKLNSLNEPLITMPIGYVTHGFNLEEAARCMRSLKAPAVVSVSSPDAVTTYN         1463
AY278488.2        KEPVASIITKLNSLNEPLVTMPIGYVTHGFNLEEAARCMRSLKAPAVVSVSSPDAVTTYN         1463
AY485277.1        KEPVASIITKLNSLNEPLVTMPIGYVTHGFNLEEAARCMRSLKAPAVVSVSSPDAVTTYN         1463
AP006560.1        KEPVASIITKLNSLNEPLVTMPIGYVTHGFNLEEAARCMRSLKAPAVVSVSSPDAVTTYN         1463
AP006557.1        KEPVASIITKLNSLNEPLVTMPIGYVTHGFNLEEAARCMRSLKAPAVVSVSSPDAVTTYN         1463
AY274119          KEPVASIITKLNSLNEPLVTMPIGYVTHGFNLEEAARCMRSLKAPAVVSVSSPDAVTTYN         1463
AY572038.1        KEPVASIITKLNSLNEPLVTMPIGYVTHGFNLEEAARCMRSLKAPAVVSVSSPDAVTTYN         1463
AY572034.1        KEPVASIITKLNSLNEPLVTMPIGYVTHGFNLEEAARCMRSLKAPAVVSVSSPDAVTTYN         1463
FJ588686.1        KEPVASIITKLNSLNEPLVTMPIGYVTHGFNLEDAARCMRSLKAPAVVSVSSPDAVTTYN         1463
KY417145.1        KEPVASIITKLNSLNEPLVTMPIGYVTHGFNLEEAARCMRSLKAPAVVSVSSPDAVTTYN         1463
KY417144.1        KEPVASIITKLNSLNEPLVTMPIGYVTHGFNLEEAARCMRSLKAPAVVSVSSPDAVTTYN         1463
KY417147.1        KEPVASIITKLNSLNEPLVTMPIGYVTHGFNLEEAARCMRSLKAPAVVSVSSPDAVTTYN         1463
KY417148.1        KEPVASIITKLNSLNEPLVTMPIGYVTHGFNLEEAARCMRSLKAPAVVSVSSPDAVTTYN         1463
KY417143.1        KEPVASIITKLNSLNEPLVTMPIGYVTHGFNLEEAARCMRSLKAPAVVSVSSPDAVTTYN         1463
KT444582.1        KEPVASIITKLNSLNEPLVTMPIGYVTHGFNLEEAARCMRSLKAPAVVSVSSPDAVTTYN         1463
KC881005.1        KEPVASIIMKLNSLNEPLVTMPIGYVTHGFNLEEAARCMRSLKAPAVVSVSSPDAVTTYN         1463
KC881006.1        KEPVASIITKLNSLNEPLVTMPIGYVTHGFNLEEAARCMRSLKAPAVVSVSSPDAVTTYN         1463
KF367457.1        KEPVASIITKLNSLNEPLVTMPIGYVTHGFNLEEAARCMRSLKAPAVVSVSSPDAVTTYN         1463
KY417152.1        KEPVASIITKLNSLNEPLVTMPIGYVTHGFNLEEAARCMRSLKAPAVVSVSSPDAVTTYN         1463
KY417146.1        KEPVASIITKLNSLNEPLVTMPIGYVTHGFNLEEAARCMRSLKAPAVVSVSSPDAVTTYN         1463
KY417151.1        KEPVASIITKLNSLNEPLVTMPIGYVTHGFNLEEAARCMRSLKAPAVVSVSSPDAVTTYN         1463
KY417142.1        KEPVASIITKLNSLNEPLVTMPIGYVTHGFNLEEAARCMRSLKAPAVVSVSSPDAVTTYN         1463
                  *  :* :* .**.*.* *:***:******:***::** ***:*.*..**********:**
```

```
MW532698.1        GYLTSSSKTPEEHFIETISLAGSYKDWSYSGQATELGIEFLKRGDKVVYHTTSKPITFHM        1538
MT040336.1        GYLTSSSKTPEEHFIETISLAGSYKDWSYSGQATELGIEFLKRGDKVVYHTTSKPITFHM        1538
MT040335.1        GYLTSSSKTPEEHFIETISLAGSYKDWSYSGQATELGIEFLKRGDKVVYHTTSKPITFHM        1538
MT040334.1        GYLTSSSKTPEEHFIETISLAGSYKDWSYSGQATELGIEFLKRGDKVVYHTTSKPITFHM        1538
MT040333.1        GYLTSSSKTPEEHFIETISLAGSYKDWSYSGQATELGIEFLKRGDKVVYHTTSKPITFHM        1538
MN996532.2        GYLTSSSKTPEEHFIETISLAGSYKDWSYSGQSTQLGIEFLKRGDKSVYYT-SNPITFHL        1545
MN988713.1        GYLTSSSKTPEEHFIETISLAGSYKDWSYSGQSTQLGIEFLKRGDKSVYYT-SNPTTFHL        1546
MT093571.1        GYLTSSSKTPEEHFIETISLAGSYKDWSYSGQSTQLGIEFLKRGDKSVYYT-SNPTTFHL        1546
MN996529.1        GYLTSSSKTPEEHFIETISLAGSYKDWSYSGQSTQLGIEFLKRGDKSVYYT-SNPTTFHL        1546
MT072688.1        GYLTSSSKTPEEHFIETISLAGSYKDWSYSGQSTQLGIEFLKRGDKSVYYT-SNPTTFHL        1546
NC_045512.2       GYLTSSSKTPEEHFIETISLAGSYKDWSYSGQSTQLGIEFLKRGDKSVYYT-SNPTTFHL        1546
MN994467.1        GYLTSSSKTPEEHFIETISLAGSYKDWSYSGQSTQLGIEFLKRGDKSVYYT-SNPTTFHL        1546
MG772933.1        GYLTSSSKTPEEHFIETVSLAGSYKDWSYSGQSTQLGIEFLKRGDKSVYYT-SNPTTFHL        1542
MG772934.1        GYLTSSSKTPEEHFIETVSLAGSYKDWSYSGQSTELGIEFLKRGDKSVYYT-SNPITFYL        1520
NC_014470.1       GYVTSASKSAEEHFIETVSLAGSYKDWSYSGQRTELGVEFLKRGDKIVYHTVGNVIEFHM        1513
GQ153542.1        GYLTSSSKTPEEHFVETISLAGSYRDWSYSGQRTELGVEFLKRGDKIVYHTTGSPIEFHL        1521
DQ022305          GYLTSSSKTPEEYFVETTSLAGSYRDWSYSGQRTELGVEFLKRGDKIVYHTTGSPIEFHL        1517
KF569996.1        GYLTSSSKTPEEHFVETVSLAGSYRDWSFSGQRTELGVEFLKRGDKIVYHTLESPIEFHL        1523
KP886809.1        GYLTSSSKTPEEHFVETVSLAGSYRDWSYSGQRTELGVEFLKRGDKIVYHTLESPIEFHL        1523
AY278488.2        GYLTSSSKTSEEHFVETVSLAGSYRDWSYSGQRTELGVEFLKRGDKIVYHTLESPVEFHL        1523
AY485277.1        GYLTSSSKTSEEHFVETVSLAGSYRDWSYSGQRTELGVEFLKRGDKIVYHTLESPVEFHL        1523
AP006560.1        GYLTSSSKTSEEHFVETVSLAGSYRDWSYSGQRTELGVEFLKRGDKIVYHTLESPVEFHL        1523
AP006557.1        GYLTSSSKTSEEHFVETVSLAGSYRDWSYSGQRTELGVEFLKRGDKIVYHTLESPVEFHL        1523
AY274119          GYLTSSSKTSEEHFVETVSLAGSYRDWSYSGQRTELGVEFLKRGDKIVYHTLESPVEFHL        1523
AY572038.1        GYLTSSSKTSEEHFVETVSLAGSYRDWSYSGQRTELGVEFLKRGDKIVYHTLESPVEFHL        1523
AY572034.1        GYLTSSSKTSEEHFVETVSLAGSYRDWSYSGQRTELGVEFLKRGDKIVYHTLESPVEFHL        1523
FJ588686.1        GYLTSSSKTSEEHFVETVSLAGSYRDWSYSGQRTELGVEFLKRGEKIVYHTLESPVEFHL        1523
KY417145.1        GYLTSSSKTSEEHFVETVSLAGSYRDWSYSGQRTELGVEFLKRGDKIVYHTLESPVEFHL        1523
KY417144.1        GYLTSSSKTSEEHFVETVSLAGSYRDWSYSGQRTELGVEFLKRGDKIVYHTLESPVEFHL        1523
KY417147.1        GYLTSSSKTSEEHFVETVSLAGSYRDWSYSGQRTELGVEFLKRGDKIVYHTLESPVEFHL        1523
KY417148.1        GYLTSSSKTSEEHFVETVSLAGSYRDWSYSGQRTELGVEFLKRGDKIVYHTLESPVEFHL        1523
KY417143.1        GYLTSSSKTSEEHFVETVSLAGSYRDWSYSGQRTELGVEFLKRGDKIVYHTLESPVEFHL        1523
KT444582.1        GYLTSSSKTSEEHFVETVSLAGSYRDWSYSGQRTELGVEFLKRGDKIVYHTLEGPVEFHL        1523
KC881005.1        GYLTSSSKTPEEHFVETVSLAGSYRDWSYSGQRTELGVEFLKRGDKIVYHTLESPVEFHL        1523
KC881006.1        GYLTSSSKTSEEHFVETVSLAGSYRDWYYSGQRTELGVEFLKRGDKIVYHTLESPVEFHL        1523
KF367457.1        GYLTSSSKTSEEHFVETVSLAGSYRDWYYSGQRTELGVEFLKRGDKIVYHTLESPVEFHL        1523
KY417152.1        GYLTSSSKTSEEHFVETVSLAGSYRDWYYSGQRTELGVEFLKRGDKIVYHTLESPVEFHL        1523
KY417146.1        GYLTSSSKTSEEHFVETVSLAGSYRDWSYSGQRTELGVEFLKRGDKIVYHTLESPVEFHL        1523
KY417151.1        GYLTSSSKTSEEHFVETVSLAGSYRDWSYSGQRTELGVEFLKRGDKIVYHTLESPVEFHL        1523
KY417142.1        GYLTSSSKTSEEHFVETVSLAGSYRDWSYSGQRTELGVEFLKRGDKIVYHTLESPVEFHL        1523
                  **:**:**: **:*:** ******:** :*** *:**:******:* **:*      *::
```

```
MW532698.1      DGEVINIDSLKTLLALREVKTIKVFTTVDNINLHTQVVDMSMTYGQQFGPTYLDGADVTK      1598
MT040336.1      DGEVINIDSLKTLLALREVKTIKVFTTVDNINLHTQVVDMSMTYGQQFGPTYLDGADVTK      1598
MT040335.1      DGEVINIDSLKTLLALREVKTIKVFTTVDNINLHTQVVDMSMTYGQQFGPTYLDGADVTK      1598
MT040334.1      DGEVINIDSLKTLLALREVKTIKVFTTVDNINLHTQVVDMSMTYGQQFGPTYLDGADVTK      1598
MT040333.1      DGEVINIDSLKTLLALREVKTIKVFTTVDNINLHTQVVDMSMTYGQQFGPTYLEGADVTK      1598
MN996532.2      DGEVITFDNLKILLSLKEVRTIKVFTTVDNINLHTQVVDMSMTYGQQFGPTYLDGADVTK      1605
MN988713.1      DGEVITFDNLKTLLSLREVRTIKVFTTVDNINLHTQVVDMSMTYGQQFGPTYLDGADVTK      1606
MT093571.1      DGEVITFDNLKTLLSLREVRTIKVFTTVDNINLHTQVVDMSMTYGQQFGPTYLDGADVTK      1606
MN996529.1      DGEVITFDNLKTLLSLREVRTIKVFTTVDNINLHTQVVDMSMTYGQQFGPTYLDGADVTK      1606
MT072688.1      DGEVITFDNLKTLLSLREVRTIKVFTTVDNINLHTQVVDMSMTYGQQFGPTYLDGADVTK      1606
NC_045512.2     DGEVITFDNLKTLLSLREVRTIKVFTTVDNINLHTQVVDMSMTYGQQFGPTYLDGADVTK      1606
MN994467.1      DGEVITFDNLKTLLSLREVRTIKVFTTVDNINLHTQVVDMSMTYGQQFGPTYLDGADVTK      1606
MG772933.1      DGETITFDNLKTLLSLREVRNIKVFTTVDNINLHTQVVDMSMTYGQQFGPIYLDGADVTK      1602
MG772934.1      DGEVITFDNLKTILSLREVRSIKVFTTVDNINLHTQIVDMSMTYGQQFGPTYLDGADVTK      1580
NC_014470.1     EGEVLPLEKLKTLLALREVKTIKVFTTVDNINLHTQVIDMSMTYGQQLGPTYMDGADLTK      1573
GQ153542.1      DGEVLPLDKLKSLLSLREVKTIKVFTTVDNTNLHTHIVDMSMTYGQQFGPTYLDGADVTK      1581
DQ022305        DGEVLPLDKLKSLLSLREVKTIKVFTTVDNTNLHTHIVDMSMTYGQQFGPTYLDGADVTK      1577
KF569996.1      DGEVLPLDKLKSLLSLREVKTIKVFTTVDNTNLHTQIVDMSMTYGQQFGPIYLDGADVTK      1583
KP886809.1      DGEVQPLDKLKSLLSLRAVKTIKVFTTVDNTNLHTQIVDMSMTYGQQFGPTYLDGADVTK      1583
AY278488.2      DGEVLSLDKLKSLLSLREVKTIKVFTTVDNTNLHTQLVDMSMTYGQQFGPTYLDGADVTK      1583
AY485277.1      DGEVLSLDKLKSLLSLREVKTIKVFTTVDNTNLHTQLVDMSMTYGQQFGPTYLDGADVTK      1583
AP006560.1      DGEVLSLDKLKSLLSLREVKTIKVFTTVDNTNLHTQLVDMSMTYGQQFGPTYLDGADVTK      1583
AP006557.1      DGEVLSLDKLKSLLSLREVKTIKVFTTVDNTNLHTQLVDMSMTYGQQFGPTYLDGADVTK      1583
AY274119        DGEVLSLDKLKSLLSLREVKTIKVFTTVDNTNLHTQLVDMSMTYGQQFGPTYLDGADVTK      1583
AY572038.1      DGEVLSLDKLKSLLSLREVKTIKVFTTVDNTNLHTQLVDMSMTYGQQFGPTYLDGADVTK      1583
AY572034.1      DGEVLSLDKLKSLLSLREVKTIKVFTTVDNTNLHTQLVDMSMTYGQQFGPTYLDGADVTK      1583
FJ588686.1      DGEVLPLDELKSLLSLREVKTIKVFTTVDNTNLHTQLVDMSMTYGQQFGPTYLDGADVTK      1583
KY417145.1      DGEVLPIDKLKSLLSLREVKTIKVFTTVDNTNLHTQLVDMSMTYGQQFGPTYLDGADVTK      1583
KY417144.1      DGEVLPLDKLKSLLSLREVKTIKVFTTVDNTNLHTQLVDMSMTYGQQFGPTYLDGADVTK      1583
KY417147.1      DGEVLPLDKLKSLLSLREVKTIKVFTTVDNTNLHTQLVDMSMTYGQQFGPTYLDGADVTK      1583
KY417148.1      DGEVLPLDKLKSLLSLREVKTIKVFTTVDNTNLHTQLVDMSMTYGQQFGPTYLDGADVTK      1583
KY417143.1      DGEVLPLDKLKSLLSLREVKTIKVFTTVDNTNLHTQLVDMSMTYGQQFGPTYLDGADVTK      1583
KT444582.1      DGEVLPLDKLKSLLSLREVKTIKVFTTVDNTNLHTQLVDMSMTYGQQFGPTYLDGADVTK      1583
KC881005.1      DGEVLPLDKLKSLLSLREVKTIKVFTTVDNTNLHTQLVDMSMTYGQQFGPTYLDGADVTK      1583
KC881006.1      DGEVLPLDKLKSLLSLREVKTIKVFTTVDNTNLHTQLVDMSMTYGQQFGPTYLDGADVTK      1583
KF367457.1      DGEVLPLDKLKSLLSLREVKTIKVFTTVDNTNLHTQLVDMSMTYGQQFGPTYLDGADVTK      1583
KY417152.1      DGEVLPLDKLKSLLSLREVKTIKVFTTVDNTNLHTQLVDMSMTYGQQFGPTYLDGADVTK      1583
KY417146.1      DGEVLPLDKLKSLLSLREVKTIKVFTTVDNTNLHTQLVDMSMTYGQQFGPTYLDGADVTK      1583
KY417151.1      DGEVLPLDKLKSLLSLREVKTIKVFTTVDNTNLHTQLVDMSMTYGQQFGPTYLDGADVTK      1583
KY417142.1      DGEVLPLDKLKSLLSLREVKTIKVFTTVDNTNLHTQLVDMSMTYGQQFGPTYLDGADVTK      1583
                :**.  ::.** :*:*: *:.********* ****:::*********:** *::***:**
```

```
MW532698.1       IKPHASHDSKTFYVLPNDDTLRSEAFEYYHTTDESFLGRYMSALNHTKKWKFPQVNGLTS        1658
MT040336.1       IKPHASHDSKTFYVLPNDDTLRSEAFEYYHTTDESFLGRYMSALNHTKKWKFPQVNGLTS        1658
MT040335.1       IKPHASHDSKTFYVLPNDDTLRSEAFEYYHTTDESFLGRYMSALNHTKKWKFPQVNGLTS        1658
MT040334.1       IKPHASHDSKTFYVLPNDDTLRSEAFEYYHTTDESFLGRYMSALNHTKKWKFPQVNGLTS        1658
MT040333.1       IKPHASHDSKTFYVLPNDDTLRSEAFEYYHTTDESFLGRYMSALNHTKKWKFPQVNGLTS        1658
MN996532.2       IKPHNSHEGKTFYVLPNDDTLRAEAFEYYHTTDPSFLGRYMSALNHTKKWKYPQVNGLTS        1665
MN988713.1       IKPHNSHEGKTFYVLPNDDTLRVEAFEYYHTTDPSFLGRYMSALNHTKKWKYPQVNGLTS        1666
MT093571.1       IKPHNSHEGKTFYVLPNDDTLRVEAFEYYHTTDPSFLGRYMSALNHTKKWKYPQVNGLTS        1666
MN996529.1       IKPHNSHEGKTFYVLPNDDTLRVEAFEYYHTTDPSFLGRYMSALNHTKKWKYPQVNGLTS        1666
MT072688.1       IKPHNSHEGKTFYVLPNDDTLRVEAFEYYHTTDPSFLGRYMSALNHTKKWKYPQVNGLTS        1666
NC_045512.2      IKPHNSHEGKTFYVLPNDDTLRVEAFEYYHTTDPSFLGRYMSALNHTKKWKYPQVNGLTS        1666
MN994467.1       IKPHNSHEGKTFYVLPNDDTLRVEAFEYYHTTDPSFLGRYMSALNHTKKWKYPQVNGLTS        1666
MG772933.1       IKPHNSHEGKTFYVLPNDDTLRAEAFEYYHTTDPSFLGRYMSALNHTKKWKYPQVNGLTS        1662
MG772934.1       IKPHNSHEGKTFYVLPNDDTLRVEAFEYYHTTDSSFLGRYMSALNHTKKWKYPQVNGLTS        1640
NC_014470.1      VKPHASHENKTFFVLPSDDTLRIEAFEYYHTVDESFFGRYMSALNHTKRWKYPQVGGLTS        1633
GQ153542.1       IKPHVNHEGKTFFVLPSDDTLRSEAFEYYHTIDESFLGRYMSALNHTKKWKFPQVGGLTS        1641
DQ022305         IKPHVNHEGKTFFVLPSDDTLRSEAFEYYHTIDESFLGRYMSALNHTKKWKFPQVGGLTS        1637
KF569996.1       IKPHANHEGKTFFVLPSDDTLRSEAFEYYHTLDESFLGRYMSALNHTKKWKFPQVGGLTS        1643
KP886809.1       IKPHVNHEGKTFYVLPSDDTLRSEAFEYYHTLDESFLGRYMSALNHTKKWKFPQVGGLTS        1643
AY278488.2       IKPHVNHEGKTFFVLPSDDTLRSEAFEYYHTLDESFLGRYMSALNHTKKWKFPQVGGLTS        1643
AY485277.1       IKPHVNHEGKTFFVLPSDDTLRSEAFEYYHTLDESFLGRYMSALNHTKKWKFPQVGGLTS        1643
AP006560.1       IKPHVNHEGKTFFVLPSDDTLRSEAFEYYHTLDESFLGRYMSALNHTKKWKFPQVGGLTS        1643
AP006557.1       IKPHVNHEGKTFFVLPSDDTLRSEAFEYYHTLDESFLGRYMSALNHTKKWKFPQVGGLTS        1643
AY274119         IKPHVNHEGKTFFVLPSDDTLRSEAFEYYHTLDESFLGRYMSALNHTKKWKFPQVGGLTS        1643
AY572038.1       IKPHVNHEGKTFFVLPSDDTLRSEAFEYYHTLDESFLGRYMSALNHTKKWKFPQVGGLTS        1643
AY572034.1       IKPHVNHEGKTFFVLPSDDTLRSEAFEYYHTLDESFLGRYMSALNHTKKWKFPQVGGLTS        1643
FJ588686.1       IKPHVNHEGKTFFVLPSDDTLRSEAFEYYHTLDESFLGRYMSALNHTKKWKFPQVGGLTS        1643
KY417145.1       IKPHVNHEGKTFFVLPSDDTLRSEAFEYYHTLDESFLGRYMSALNHTKKWKFPQVGGLTS        1643
KY417144.1       IKPHVNHEGKTFFVLPSDDTLRSEAFEYYHTLDESFLGRYMSALNHTKKWKFPQVGGLTS        1643
KY417147.1       IKPHVNHEGKTFFVLPSDDTLRSEAFEYYHTLDESFLGRYMSALNHTKKWKFPQVGGLTS        1643
KY417148.1       IKPHVNHEGKTFFVLPSDDTLRSEAFEYYHTLDESFLGRYMSALNHTKKWKFPQVGGLTS        1643
KY417143.1       IKPHVNHEGKTFFVLPSDDTLRSEAFEYYHTLDESFLGRYMSALNHTKKWKFPQVGGLTS        1643
KT444582.1       IKPHVNHEGKTFFVLPSDDTLRSEAFEYYHTLDESFLGRYMSALNHTKKWKFPQVGGLTS        1643
KC881005.1       IKPHVNHEGKTFFVLPSDDTLRSEAFEYYHTLDESFLGRYMSALNHTKKWKFPQVGGLTS        1643
KC881006.1       IKPHVNHEGKTFFVLPSDDTLRSEAFEYYHTLDESFLGRYMSALNHTKKWKFPQVGGLTS        1643
KF367457.1       IKPHVNHEGKTFFVLPSDDTLRSEAFEYYHTLDESFLGRYMSALNHTKKWKFPQVGGLTS        1643
KY417152.1       IKPHVNHEGKTFFVLPSDDTLRSEAFEYYHTLDESFLGRYMSALNHTKKWKFPQVGGLTS        1643
KY417146.1       IKPHVNHEGKTFFVLPSDDTLRSEAFEYYHTLDESFLGRYMSALNHTKKWKFPQVGGLTS        1643
KY417151.1       IKPHVNHEGKTFFVLPSDDTLRSEAFEYYHTLDESFLGRYMSALNHTKKWKFPQVGGLTS        1643
KY417142.1       IKPHVNHEGKTFFVLPSDDTLRSEAFEYYHTLDESFLGRYMSALNHTKKWKFPQVGGLTS        1643
                 :*** .*:.***:***.***** ******** * **:***********:**:***.****
```

```
MW532698.1      IKWADNNCYLATALLTLQQIELKFNPPALQDAYYRARAGDAANFCALILAYCNKTVGELG      1718
MT040336.1      IKWADNNCYLATALLTLQQIELKFNPPALQDAYYRARAGDAANFCALILAYCNKTVGELG      1718
MT040335.1      IKWADNNCYLATALLTLQQIELKFNPPALQDAYYRARVGDAANFCALILAYCNKTVGELG      1718
MT040334.1      IKWADNNCYLATALLTLQQIELKFNPPALQDAYYRARVGDAANFCALILAYCNKTVGELG      1718
MT040333.1      IKWADNNCYLATALLTLQQIELKFNPPALQDAYYRARAGDAANFCALILAYCNKTVGELG      1718
MN996532.2      IKWADNNCYLATALLTLQQIELKFNPPALQDAYYRARAGEAANFCALILAYCNKTVGELG      1725
MN988713.1      IKWADNNCYLATALLTLQQIELKFNPPALQDAYYRARAGEAANFCALILAYCNKTVGELG      1726
MT093571.1      IKWADNNCYLATALLTLQQIELKFNPPALQDAYYRARAGEAANFCALILAYCNKTVGELG      1726
MN996529.1      IKWADNNCYLATALLTLQQIELKFNPPALQDAYYRARAGEAANFCALILAYCNKTVGELG      1726
MT072688.1      IKWADNNCYLATALLTLQQIELKFNPPALQDAYYRARAGEAANFCALILAYCNKTVGELG      1726
NC_045512.2     IKWADNNCYLATALLTLQQIELKFNPPALQDAYYRARAGEAANFCALILAYCNKTVGELG      1726
MN994467.1      IKWADNNCYLATALLTLQQIELKFNPPALQDAYYRARAGEAANFCALILAYCNKTVGELG      1726
MG772933.1      IKWADNNCYLATALLTIQQIELKFNPPALQDAYYRARAGEAANFCALILAYCNKTVGELG      1722
MG772934.1      IRWADNNCYLATALLTLQQIELKFNPPALQDAYYRARAGEAANFCALILAYCNKTVGELG      1700
NC_014470.1     IKWADNNCYLSSVLLSLQQIDIKFNAPALQDAYYRARAGDAANFCALVLAYSKKTVGELG      1693
GQ153542.1      IKWADNNCYLSSVLLALQQVEVKFNAPALQEAYYRARAGDAANFCALILAYSNKTVGELG      1701
DQ022305        IKWADNNCYLSSVLLALQQVEVKFNAPALQEAYYRARAGDAANFCALILAYSNKTVGELG      1697
KF569996.1      IKWADNNCYLSSVLLALQQIEVKFNAPALQEAYYRARAGDAANFCALILAYSNKTVGELG      1703
KP886809.1      IKWADNNCYLSSVLLALQQIEVKFNAPALQEAYYRARAGDAANFCALILAYSNKTVGELG      1703
AY278488.2      IKWADNNCYLSSVLLALQQLEVKFNAPALQEAYYRARAGDAANFCALILAYSNKTVGELG      1703
AY485277.1      IKWADNNCYLSSVLLALQQLEVKFNAPALQEAYYRARAGDAANFCALILAYSNKTVGELG      1703
AP006560.1      IKWADNNCYLSSVLLALQQLEVKFNAPALQEAYYRARAGDAANFCALILAYSNKTVGELG      1703
AP006557.1      IKWADNNCYLSSVLLALQQLEVKFNAPALQEAYYRARAGDAANFCALILAYSNKTVGELG      1703
AY274119        IKWADNNCYLSSVLLALQQLEVKFNAPALQEAYYRARAGDAANFCALILAYSNKTVGELG      1703
AY572038.1      IKWADNNCYLSSVLLALQQIEVKFNAPALQEAYYRARAGDAANFCALILAYSNKTVGELG      1703
AY572034.1      IKWADNNCYLSSVLLALQQIEVKFNAPALQEAYYRARAGDAANFCALILAYSNKTVGELG      1703
FJ588686.1      IKWADNNCYLSSVLLALQQIEVKFNAPALQEAYYRARAGDAANFCALILAYSNKTVGELG      1703
KY417145.1      IKWADNNCYLSSVLLALQQIEVKFNAPALQEAYYRARAGDAANFCALILAYSNKTVGELG      1703
KY417144.1      IKWADNNCYLSSVLLALQQIEVKFNAPALQEAYYRARAGDAANFCALILAYSNKTVGELG      1703
KY417147.1      IKWADNNCYLSSVLLALQQIEVKFNAPALQEAYYRARAGDAANFCALILAYSNKTVGELG      1703
KY417148.1      IKWADNNCYLSSVLLTLQQIEVKFNAPALQEAYYRARAGDAANFCALILAYSNKTVGELG      1703
KY417143.1      IKWADNNCYLSSVLLALQQIEVKFNAPALQEAYYRARAGDAANFCALILAYSNKTVGELG      1703
KT444582.1      IKWADNNCYLSSVLLALQQIEVKFNAPALQEAYYRARAGDAANFCALILAYSNKTVGELG      1703
KC881005.1      IKWADNNCYLSSVLLALQQIEVKFNAPALQEAYYRARAGDAANFCALILAYSNKTVGELG      1703
KC881006.1      IKWADNNCYLSSVLLALQQIEVKFNAPALQEAYYRARAGDAANFCALILAYSNKTVGELG      1703
KF367457.1      IKWADNNCYLSSVLLALQQIEVKFNAPALQEAYYRARAGDAANFCALILAYSNKTVGELG      1703
KY417152.1      IKWADNNCYLSSVLLALQQIEVKFNAPALQEAYYRARAGDAANFCALILAYSNKTVGELG      1703
KY417146.1      IKWADNNCYLSSVLLALQQIEVKFNAPALQEAYYRARAGDAANFCALILAYSNKTVGELG      1703
KY417151.1      IKWADNNCYLSSVLLALQQIEVKFNAPALQEAYYRARAGDAANFCALILAYSNKTVGELG      1703
KY417142.1      IKWADNNCYLSSVLLALQQIEVKFNAPALQEAYYRARAGDAANFCALILAYSNKTVGELG      1703
                *:********::.**::**:::*** ****:******.*:*******:***.:*******
```

```
MW532698.1      DVRETMSHLFQHANLDSCKRVLNVVCKTCGQQQTTLKGVEAVMYMGTLSYEQLKRGVTVP      1778
MT040336.1      DVRETMSHLFQHANLDSCKRVLNVVCKTCGQQQTTLKGVEAVMYMGTLSYEQLKRGVTVP      1778
MT040335.1      DVRETMSHLFQHANLDSCKRVLNVVCKTCGQQQTTLKGVEAVMYMGTLSYEQLKRGVTVP      1778
MT040334.1      DVRETMSHLFQHANLDSCKRVLNVVCKTCGQQQTTLKGVEAVMYMGTLSYEQLKRGVTVP      1778
MT040333.1      DVRETMSHLFQHANLDSCKRVLNVVCKTCGQQQTTLKGVEAVMYMGTLSYEQLKRGVTVP      1778
MN996532.2      DVRETMNYLFQHANLDSCKRVLNVVCKTCGQQQTTLKGVEAVMYMGTLSYEQLKKGVQIP      1785
MN988713.1      DVRETMSYLFQHANLDSCKRVLNVVCKTCGQQQTTLKGVEAVMYMGTLSYEQFKKGVQIP      1786
MT093571.1      DVRETMSYLFQHANLDSCKRVLNVVCKTCGQQQTTLKGVEAVMYMGTLSYEQFKKGVQIP      1786
MN996529.1      DVRETMSYLFQHANLDSCKRVLNVVCKTCGQQQTTLKGVEAVMYMGTLSYEQFKKGVQIP      1786
MT072688.1      DVRETMSYLFQHANLDSCKRVLNVVCKTCGQQQTTLKGVEAVMYMGTLSYEQFKKGVQIP      1786
NC_045512.2     DVRETMSYLFQHANLDSCKRVLNVVCKTCGQQQTTLKGVEAVMYMGTLSYEQFKKGVQIP      1786
MN994467.1      DVRETMSYLFQHANLDSCKRVLNVVCKTCGQQQTTLKGVEAVMYMGTLSYEQFKKGVQIP      1786
MG772933.1      DVRETMNYLFQHANLDSCKRVLNVVCKTCGQQQTTLKGVEAVMYMGTLSYEQLKKGVQIP      1782
MG772934.1      DVRETMNYLFQHANLDSCKRVLNVVCKTCGQQQTTLKGVEAVMYMGTLSYEQLKKGVQIP      1760
NC_014470.1     DVRETMAHLLQHANLESAKRVLNVVCKHCGQKSTTLSGVEAVMYMGTLSYDHLKRGVKIP      1753
GQ153542.1      DVRETMTHLLQHANLESAKRVLNVVCKHCGQKTTTLKGVEAVMYMGTLSYDELKTGVSIP      1761
DQ022305        DVRETMTHLLQHANLESAKRVLNVVCKHCGQKTTTLKGVEAVMYMGTLSYDELKTGVSIP      1757
KF569996.1      DVRETMAHLLQHANLESAKRVLNVVCKHCGQKTTTLMGVEAVMYMGTLSYDNLKTGVSVP      1763
KP886809.1      DVRETMTHLLQHANLESAKRVLNVVCKHCGQKTTTLTGVEAVMYMGTLSYDNLKTGVSIP      1763
AY278488.2      DVRETMTHLLQHANLESAKRVLNVVCKHCGQKTTTLTGVEAVMYMGTLSYDNLKTGVSIP      1763
AY485277.1      DVRETMTHLLQHANLESAKRVLNVVCKHCGQKTTTLTGVEAVMYMGTLSYDNLKTGVSIP      1763
AP006560.1      DVRETMTHLLQHANLESAKRVLNVVCKHCGQKTTTLTGVEAVMYMGTLSYDNLKTGVSIP      1763
AP006557.1      DVRETMTHLLQHANLESAKRVLNVVCKHCGQKTTTLTGVEAVMYMGTLSYDNLKTGVSIP      1763
AY274119        DVRETMTHLLQHANLESAKRVLNVVCKHCGQKTTTLTGVEAVMYMGTLSYDNLKTGVSIP      1763
AY572038.1      DVRETMTHLLQHANLESAKRVLNVVCKHCGQKTTTLTGVEAVMYMGTLSYDNLKTGVSIP      1763
AY572034.1      DVRETMTHLLQHANLESAKRVLNVVCKHCGQKTTTLTGVEAVMYMGTLSYDNLKTGVSIP      1763
FJ588686.1      DVRETMTHLLQHANLESAKRVLNVVCKHCGQKTTTLTGVEAVMYMGTLSYDNLKTGVSIP      1763
KY417145.1      DVRETMTHLLQHANLESAKRVLNVVCKHCGQKTTTLTGVEAVMYMGTLSYDNLKTGVSIP      1763
KY417144.1      DVRETMTHLLQHANLESAKRVLNVVCKHCGQKTTTLTGVEAVMYMGTLSYDNLKTGVSIP      1763
KY417147.1      DVRETMTHLLQHANLESAKRVLNVVCKHCGQKTTTLTGVEAVMYMGTLSYDNLKTGVSIP      1763
KY417148.1      DVRETMTHLLQHANLESAKRVLNVVCKHCGQKTTTLTGVEAVMYMGTLSYDNLKTGVSIP      1763
KY417143.1      DVRETMTHLLQHANLESAKRVLNVVCKHCGQKTTTLTGVEAVMYMGTLSYDNLKTGVSIP      1763
KT444582.1      DVRETMTHLLQHANLESAKRVLNVVCKHCGQKTTTLTGVEAVMYMGTLSYDNLKTGVSIP      1763
KC881005.1      DVRETMTHLLQHANLESAKRVLNVVCKHCGQKTTTLTGVEAVMYMGTLSYDNLKTGVSIP      1763
KC881006.1      DVRETMTHLLQHANLESAKRVLNVVCKHCGQKTTTLTGVEAVMYMGTLSYDNLKTGVSIP      1763
KF367457.1      DVRETMTHLLQHANLESAKRVLNVVCKHCGQKTTTLTGVEAVMYMGTLSYDNLKTGVSIP      1763
KY417152.1      DVRETMTHLLQHANLESAKRVLNVVCKHCGQKTTTLTGVEAVMYMGTLSYDNLKTGVSIP      1763
KY417146.1      DVRETMTHLLQHANLESAKRVLNVVCKHCGQKTTTLTGVEAVMYMGTLSYDNLKTGVSIP      1763
KY417151.1      DVRETMTHLLQHANLESAKRVLNVVCKHCGQKTTTLTGVEAVMYMGTLSYDNLKTGVSIP      1763
KY417142.1      DVRETMTHLLQHANLESAKRVLNVVCKHCGQKTTTLTGVEAVMYMGTLSYDNLKTGVSIP      1763
                ****** :*:*****:*.********* ***: *** *************:.:* ** :*
```

```
MW532698.1       CVCGRQATQYLVQQESSFVMMSAPPAEYKLKHGTFLCASEYTGNYQCGHYKHITSKETLY        1838
MT040336.1       CVCGRQATQYLVQQESSFVMMSAPPAEYKLKHGTFLCASEYTGNYQCGHYKHITSKETLY        1838
MT040335.1       CVCGRQATQYLVQQESSFVMMSAPPAEYKLKHGTFLCASEYTGNYQCGHYKHITSKETLY        1838
MT040334.1       CVCGRQATQYLVQQESSFVMMSAPPAEYKLKHGTFLCASEYTGNYQCGHYKHITSKETLY        1838
MT040333.1       CVCGRQATQYLVQQESSFVMMSAPPAEYKLKHGTFLCASEYTGNYQCGHYKHITSKETLY        1838
MN996532.2       CTCGKQATQYLVQQESPFVMMSAPPAQYELKHGTFICASEYTGNYQCGHYKHITSKETLY        1845
MN988713.1       CTCGKQATKYLVQQESPFVMMSAPPAQYELKHGTFTCASEYTGNYQCGHYKHITSKETLY        1846
MT093571.1       CTCGKQATKYLVQQESPFVMMSAPPAQYELKHGTFTCASEYTGNYQCGHYKHITSKETLY        1846
MN996529.1       CTCGKQATKYLVQQESPFVMMSAPPAQYELKHGTFTCASEYTGNYQCGHYKHITSKETLY        1846
MT072688.1       CTCGKQATKYLVQQESPFVMMSAPPAQYELKHGTFTCASEYTGNYQCGHYKHITSKETLY        1846
NC_045512.2      CTCGKQATKYLVQQESPFVMMSAPPAQYELKHGTFTCASEYTGNYQCGHYKHITSKETLY        1846
MN994467.1       CTCGKQATKYLVQQESPFVMMSAPPAQYELKHGTFTCASEYTGNYQCGHYKHITSKETLY        1846
MG772933.1       CMCGKQATQYLVQQESPFVMMSAPPAQYELKHGTFVCASEYTGNYQCGHYKHITSKETLY        1842
MG772934.1       CMCGKQATQYLVQQESPFVMMSAPPAQYELKHGTFVCASEYTGNYQCGHYKHITSKETLY        1820
NC_014470.1      CVCGREATQYLVKQESTFVMMSAPPAEYTLQTGEFLCANEYTGNYQCGHYTHITNRETIY        1813
GQ153542.1       CVCGRNATQYLVQQESSFVMMSAPPAEYKLQQGAFLCANEYTGNYQCGHYTHITAKETLY        1821
DQ022305         CVCGRNATQYLVQQESSFVMMSAPPAEYKLQQGAFLCANEYTGNYQCGHYTHITAKETLY        1817
KF569996.1       CVCGRDATQYLVQQESSFVMMSAPPAEYKLQQGTFLCANEYTGNYQCGHYTHITAKETLY        1823
KP886809.1       CVCGRDATQYLIQQESSFVMMSAPPAEYKLQQGTFLCANEYTGNYQCGHYTHVTAKETLY        1823
AY278488.2       CVCGRDATQYLVQQESSFVMMSAPPAEYKLQQGTFLCANEYTGNYQCGHYTHITAKETLY        1823
AY485277.1       CVCGRDATQYLVQQESSFVMMSAPPAEYKLQQGTFLCANEYTGNYQCGHYTHITAKETLY        1823
AP006560.1       CVCGRDATQYLVQQESSFVMMSAPPAEYKLQQGTFLCANEYTGNYQCGHYTHITAKETLY        1823
AP006557.1       CVCGRDATQYLVQQESSFVMMSAPPAEYKLQQGTFLCANEYTGNYQCGHYTHITAKETLY        1823
AY274119         CVCGRDATQYLVQQESSFVMMSAPPAEYKLQQGTFLCANEYTGNYQCGHYTHITAKETLY        1823
AY572038.1       CVCGRDATQYLVQQESSFVMMSAPPAEYKLQQGTFLCANEYTGNYQCGHYTHITAKETLY        1823
AY572034.1       CVCGRDATQYLVQQESSFVMMSAPPAEYKLQQGTFLCANEYTGNYQCGHYTHITAKETLY        1823
FJ588686.1       CVCGRDATQYLVQQESSFVMMSAPPAEYKLQQGTFLCANEYTGNYQCGHYTHITAKETLY        1823
KY417145.1       CVCGRDATQYLVQQESSFVMMSAPPAEYKLQQGTFLCANEYTGNYQCGHYTHITAKETLY        1823
KY417144.1       CVCGRDATQYLVQQESSFVMMSAPPAEYKLQQGTFLCANEYTGNYQCGHYTHITAKETLY        1823
KY417147.1       CVCGRDATQYLVQQESSFVMMSAPPAEYKLQQGTFLCANEYTGNYQCGHYTHITAKETLY        1823
KY417148.1       CVCGRDATQYLVQQESSFVMMSAPPAEYKLQQGTFLCANEYTGNYQCGHYTHITAKETLY        1823
KY417143.1       CVCGRDATQYLVQQESSFVMMSAPPAEYKLQQGTFLCANEYTGNYQCGHYTHITAKETLY        1823
KT444582.1       CVCGRDATQYLVQQESSFVMMSAPPAEYKLQQGTFLCANEYTGNYQCGHYTHITAKETLY        1823
KC881005.1       CVCGRDATQYLVQQESSFVMMSAPPAEYKLQQGTFLCANEYTGNYQCGHYTHITAKETLY        1823
KC881006.1       CVCGRDATQYLVQQESSFVMMSAPPAEYKLQQGTFLCANEYTGNYQCGHYTHITAKETLY        1823
KF367457.1       CVCGRDATQYLVQQESSFVMMSAPPAEYKLQQGTFLCANEYTGNYQCGHYTHITAKETLY        1823
KY417152.1       CVCGRDATQYLVQQESSFVMMSAPPAEYKLQQGTFLCANEYTGNYQCGHYTHITAKETLY        1823
KY417146.1       CVCGRDATQYLVQQESSFVMMSAPPAEYKLQQGTFLCANEYTGNYQCGHYTHITAKETLY        1823
KY417151.1       CVCGRDATQYLVQQESSFVMMSAPPAEYKLQQGTFLCANEYTGNYQCGHYTHITAKETLY        1823
KY417142.1       CVCGRDATQYLVQQESSFVMMSAPPAEYKLQQGTFLCANEYTGNYQCGHYTHITAKETLY        1823
                 * **::**:**::*** *********:* *: * * **.***********.*:* :**:*
```

```
MW532698.1        VIDGALLSKTSEYKGPVTDVFYKENSYTTTIKPIVYKLDGVVCTEIDPKLDGYYKKDNAY        1898
MT040336.1        VIDGALLSKTSEYKGPVTDVFYKENSYTTTIKPIVYKLDGVVCTEIDPKLDGYYKKDNAY        1898
MT040335.1        VIDGALLSKTSEYKGPVTDVFYKENSYTTTIKPIVYKLDGVVCTEIDPKLDGYYKKDNAY        1898
MT040334.1        VIDGALLSKTSEYKGPVTDVFYKENSYTTTIKPIVYKLDGVVCTEIDPKLDGYYKKDNAY        1898
MT040333.1        VIDGALLSKTSEYKGPVTDVFYKENSYTTTIKPIVYKLDGVVCTEIDPKLDGYYKKDNAY        1898
MN996532.2        CIDGALLTKSSEYKGPITDIFYKENSYITTIKPVIYKLDGVVCTEIDPKLDNYYKKDNSY        1905
MN988713.1        CIDGALLTKSSEYKGPITDVFYKENSYTTTIKPVTYKLDGVVCTEIDPKLDNYYKKDNSY        1906
MT093571.1        CIDGALLTKSSEYKGPITDVFYKENSYTTTIKPVTYKLDGVVCTEIDPKLDNYYKKDNSY        1906
MN996529.1        CIDGALLTKSSEYKGPITDVFYKENSYTTTIKPVTYKLDGVVCTEIDPKLDNYYKKDNSY        1906
MT072688.1        CIDGALLTKSSEYKGPITDVFYKENSYTTTIKPVTYKLDGVVCTEIDPKLDNYYKKDNSY        1906
NC_045512.2       CIDGALLTKSSEYKGPITDVFYKENSYTTTIKPVTYKLDGVVCTEIDPKLDNYYKKDNSY        1906
MN994467.1        CIDGALLTKSSEYKGPITDVFYKENSYTTTIKPVTYKLDGVVCTEIDPKLDNYYKKDNSY        1906
MG772933.1        CIDGALLTKSSEYKGSITDVFYKENSYTTTIKPVTYKLDGVVCTEIDPKLDGYYKKDNSY        1902
MG772934.1        CIDGALLTKSSEYKGSITDVFYKENSYTTTIKPVTYKLDGVVCTEIDPKLDGYYKKDNSY        1880
NC_014470.1       KIDGALLTKITEYKGPVADVFYKETSYSTDIKPVSYKLDGVTYTEINPDLNGYYKKDNAY        1873
GQ153542.1        RVDGAHLTKMSEYKGPVTDVFYKETSYTTAIKPVSYKLDGVTYTEIEPKLDGYYKKDNAY        1881
DQ022305          RVDGAHLTKMSEYKGPVTDVFYKETSYTTAIKPVSYKLDGVTYTEIEPKLDGYYKKGNAY        1877
KF569996.1        RIDGAHLTKMSEYKGPVTDVFYKETSYTTTIKPVSYKLDGVTYTEIEPKLDGYYKKDNAY        1883
KP886809.1        RIDGAHLTKMSEYKGPVTDVFYKETSYTTTIKPVSYKLDGVTYTEIEPKLDGYYKKDNAY        1883
AY278488.2        RIDGAHLTKMSEYKGPVTDVFYKETSYTTTIKPVSYKLDGVTYTEIEPKLDGYYKKDNAY        1883
AY485277.1        RIDGAHLTKMSEYKGPVTDVFYKETSYTTTIKPVSYKLDGVTYTEIEPKLDGYYKKDNAY        1883
AP006560.1        RIDGAHLTKMSEYKGPVTDVFYKETSYTTTIKPVSYKLDGVTYTEIEPKLDGYYKKDNAY        1883
AP006557.1        RIDGAHLTKMSEYKGPVTDVFYKETSYTTTIKPVSYKLDGVTYTEIEPKLDGYYKKDNAY        1883
AY274119          RIDGAHLTKMSEYKGPVTDVFYKETSYTTTIKPVSYKLDGVTYTEIEPKLDGYYKKDNAY        1883
AY572038.1        RIDGAHLTKMSEYKGPVTDVFYKETSYTTTIKPVSYKLDGVTYTEIEPKLDGYYKKDNAY        1883
AY572034.1        RIDGAHLTKMSEYKGPVTDVFYKETSYTTTIKPVSYKLDGVTYTEIEPKLDGYYKKDNAY        1883
FJ588686.1        RIDGAHLTKMSEYKGPVTDVFYKETSYTTTIKPVSYKLDGVTYTEIEPKLDGYYKKDNAY        1883
KY417145.1        RIDGAHLTKMSEYKGPVTDVFYKETSYTTTIRPVSYKLDGVTYTEIEPKLDGYYKKDNAY        1883
KY417144.1        RIDGAHLTKMSEYKGPVTDVFYKETSYTTTIKPVSYKLDGVTYTEIEPKLDGYYKKDNAY        1883
KY417147.1        RIDGAHLTKMSEYKGPVTDVFYKETSYTTTIKPVSYKLDGVTYTEIEPKLDGYYKKDNAY        1883
KY417148.1        RIDGAHLTKMSEYKGPVTDVFYKETSYTTTIKPVSYKLDGVTYTEIEPKLDGYYKKDNAY        1883
KY417143.1        RIDGAHLTKMSEYKGPVTDVFYKETSYTTTIKPVSYKLDGVTYTEIEPKLDGYYKKDNAY        1883
KT444582.1        RIDGAHLTKMSEYKGPVTDVFYKETSYTTTIKPVSYKLDGVTYTEIEPKLDGYYKKDNAY        1883
KC881005.1        RIDGAHLTKMSEYKGPVTDVFYKETSYTTTIKPVSYKLDGVTYTEIEPKLDGYYKKDNAY        1883
KC881006.1        RIDGAHLTKMSEYKGPVTDVFYKETSYTTTIKPVSYKLDGVTYTEIEPKLDGYYKKDNAY        1883
KF367457.1        RIDGAHLTKMSEYKGPVTDVFYKETSYTTTIKPVSYKLDGVTYTEIEPKLDGYYKKDNAY        1883
KY417152.1        RIDGAHLTKMSEYKGPVTDVFYKETSYTTTIKPVSYKLDGVTYTEIEPKLDGYYKKDNAY        1883
KY417146.1        RIDGAHLTKMSEYKGPVTDVFYKETSYTTTIKPVSYKLDGVTYTEIEPKLDGYYKKDNAY        1883
KY417151.1        RIDGAHLTKMSEYKGPVTDVFYKETSYTTTIRPVSYKLDGVTYTEIEPKLDGYYKKDNAY        1883
KY417142.1        RIDGAHLTKMSEYKGPVTDVFYKETSYTTTIKPVSYKLDGVTYTEIEPKLDGYYKKDNAY        1883
                   :*** *:* :**** ::*:****.** * *:*: ******. ***:*.*:.****.*:*
```

```
MW532698.1      FTEQPIDLVPTQPYPNSNFDNFKFVCDNTKFADDLNQMSGYKKPASRELKITFFPDLNGD     1958
MT040336.1      FTEQPIDLVPTQPYPNSNFDNFKFVCDNTKFADDLNQMSGYKKPASRELKITFFPDLNGD     1958
MT040335.1      FTEQPIDLVPTQPYPNSNFDNFKFVCDNTKFADDLNQMSGYKKPASRELKITFFPDLNGD     1958
MT040334.1      FTEQPIDLVPTQPYPNSNFDNFKFVCDNTKFADDLNQMSGYKKPASRELKITFFPDLNGD     1958
MT040333.1      FTEQPIDLVPTQPYPNSNFDNFKFVCDNTKFADDLNQMSGYKKPASRELKITFFPDLNGD     1958
MN996532.2      FTEQPIDLVPNQPYPNASFDNFKFVCDNIKLADDLNQLTGYKKPASREFKVTFFPDLNGD     1965
MN988713.1      FTEQPIDLVPNQPYPNASFDNFKFVCDNIKFADDLNQLTGYKKPASRELKVTFFPDLNGD     1966
MT093571.1      FTEQPIDLVPNQPYPNASFDNFKFVCDNIKFADDLNQLTGYKKPASRELKVTFFPDLNGD     1966
MN996529.1      FTEQPIDLVPNQPYPNASFDNFKFVCDNIKFADDLNQLTGYKKPASRELKVTFFPDLNGD     1966
MT072688.1      FTEQPIDLVPNQPYPNASFDNFKFVCDNIKFADDLNQLTGYKKPASRELKVTFFPDLNGD     1966
NC_045512.2     FTEQPIDLVPNQPYPNASFDNFKFVCDNIKFADDLNQLTGYKKPASRELKVTFFPDLNGD     1966
MN994467.1      FTEQPIDLVPNQPYPNASFDNFKFVCDNIKFADDLNQLTGYKKPASRELKVTFFPDLNGD     1966
MG772933.1      FTEQPIDLVPNQPYPNASFDNFKFVCDNTKFADDLNQLSGYKKPASRELKVTFFPDLNGD     1962
MG772934.1      FTEQPIDLVPNQPYPNASFDNFRFVCDNIKFADDLNQLSGYKKPALRELKVTFFPDLNGD     1940
NC_014470.1     YTEQPIDLVPTQPLPNASFDNFRFVCANTKFADDLNQMTGFKKPPSRDLTITFFPDLNGD     1933
GQ153542.1      YTEQPIDLVPTQPMPNASFDNFKLTCSNTKFADDLNQMTGFKKPASRELTVTFFPDLNGD     1941
DQ022305        YTEQPIDLVPTQPMPNASFDNFKLTCSNTKFADDLNQMTGFKKPASRELTVTFFPDLNGD     1937
KF569996.1      YTEQPIDLVPTQPLPNASFDNFKLTCSNIKFADDLNQMTGFTKPASRELSVTFFPDLNGD     1943
KP886809.1      YTEQPIDLVPTQPLPNASFDNFKLTCSNTKFADDLNQMTGFTKPASRELSVTFFPDLNGD     1943
AY278488.2      YTEQPIDLVPTQPLPNASFDNFKLTCSNTKFADDLNQMTGFTKPASRELSVTFFPDLNGD     1943
AY485277.1      YTEQPIDLVPTQPLPNASFDNFKLTCSNTKFADDLNQMTGFTKPASRELSVTFFPDLNGD     1943
AP006560.1      YTEQPIDLVPTQPLPNASFDNFKLTCSNTKFADDLNQMTGFTKPASRELSVTFFPDLNGD     1943
AP006557.1      YTEQPIDLVPTQPLPNASFDNFKLTCSNTKFADDLNQMTGFTKPASRELSVTFFPDLNGD     1943
AY274119        YTEQPIDLVPTQPLPNASFDNFKLTCSNTKFADDLNQMTGFTKPASRELSVTFFPDLNGD     1943
AY572038.1      YTEQPIDLVPTQPLPNASFDNFKLTCSNTKFADDLNQMTGFTKPASRELSVTFFPDLNGD     1943
AY572034.1      YTEQPIDLVPTQPLPNASFDNFKLTCSNTKFADDLNQMTGFTKPASRELSVTFFPDLNGD     1943
FJ588686.1      ------------------------------------------------------------     1883
KY417145.1      YTEQPIDLVPTQPLPNASFDNFKLTCSNTKFADDLNQMTGFTKPASRELSVTFFPDLNGD     1943
KY417144.1      YTEQPIDLVPTQPLPNASFDNFKLTCSNTKFADALNQMTGFTKPASRELSVTFFPDLNGD     1943
KY417147.1      YTEQPIDLVPTQPLPNASFDNFKLTCSNTKFADDLNQMTGFTKPASRELYVTFFPDLNGD     1943
KY417148.1      YTEQPIDLVPTQPLPNASFDNFKLTCSNTKFADDLNQMTGFTKPASRELSVTFFPDLNGD     1943
KY417143.1      YTEQPIDLVPTQPLPNASFDNFKLTCSNTKFADDLNQMTGFTKPASRELSVTFFPDLNGD     1943
KT444582.1      YTEQPIDLVPTQPLPNASFDNFKLTCSNTKFADDLNQMTGFTKPASRELSVTFFPDLNGD     1943
KC881005.1      YTEQPIDLVPTQPLPNASFDNFKLTCSNTKFADNLNQMTGFTKPASRELSVTFFPDLNGD     1943
KC881006.1      YTEQPIDLVPTQPLPNASFDNFKLTCSNTKFADDLNQMTGFTKPASRELYVTFFPDLNGD     1943
KF367457.1      YTEQPIDLVPTQPLPNASFDNFKLTCSNTKFADDLNQMTGFTKPASRELYVTFFPDLNGD     1943
KY417152.1      YTEQPIDLVPTQPLPNASFDNFKLTCSNTKFADDLNQMTGFTKPASRELSVTFFPDLNGD     1943
KY417146.1      YTEQPIDLVPTQPLPNASFDNFKLTCSNTKFADDLNQMTGFTKPASRELSVTFFPDLNGD     1943
KY417151.1      YTEQPIDLVPTQPLPNASFDNFKLTCSNTKFADDLNQMTGFTKPASRELSVTFFPDLNGD     1943
KY417142.1      YTEQPIDLVPTQPLPNASFDNFKLTCSNTKFADDLNQMTGFTKPASRELSVTFFPDLNGD     1943
```

MW532698.1	VVAIDYKHYTPSFKKGAKLLHKPIVWHVNNTINKATFKPNTWCLRCLWSTKPVETSNIFD	2018
MT040336.1	VVAIDYKHYTPSFKKGAKLLHKPIVWHVNNTINKATFKPNTWCLRCLWSTKPVETSNIFD	2018
MT040335.1	VVAIDYKHYTPSFKKGAKLLHKPIVWHVNNTINKATFKPNTWCLRCLWSTKPVETSNIFD	2018
MT040334.1	VVAIDYKHYTPSFKKGAKLLHKPIVWHVNNTINKATFKPNTWCLRCLWSTKPVETSNIFD	2018
MT040333.1	VVAIDYKHYTPSFKKGAKLLHKPIVWHVNNTINKATFKPNTWCLRCLWSTKPVETSNIFD	2018
MN996532.2	VVAIDYKHYTPSFKKGAKLLHKPIVWHVNNATNKATYKPNTWCIRCLWNTKPVETSNSFD	2025
MN988713.1	VVAIDYKHYTPSFKKGAKLLHKPIVWHVNNATNKATYKPNTWCIRCLWSTKPVETSNSFD	2026
MT093571.1	VVAIDYKHYTPSFKKGAKLLHKPIVWHVNNATNKATYKPNTWCIRCLWSTKPVETSNSFD	2026
MN996529.1	VVAIDYKHYTPSFKKGAKLLHKPIVWHVNNATNKATYKPNTWCIRCLWSTKPVETSNSFD	2026
MT072688.1	VVAIDYKHYTPSFKKGAKLLHKPIVWHVNNATNKATYKPNTWCIRCLWSTKPVETSNSFD	2026
NC_045512.2	VVAIDYKHYTPSFKKGAKLLHKPIVWHVNNATNKATYKPNTWCIRCLWSTKPVETSNSFD	2026
MN994467.1	VVAIDYKHYTPSFKKGAKLLHKPIVWHVNNATNKATYKPNTWCIRCLWSTKPVETSNSFD	2026
MG772933.1	VVAIDYKHYTPSFKKGAKLLHKPIVWHVNNATNKATYKPNIWCIRCLWSTKPVETSNSFD	2022
MG772934.1	VVAIDYKHYTPSFKKGAKLLHKPIVWHVNNATNKATYKPNIWCIRCLWSTKPVETSNSFD	2000
NC_014470.1	VVAIDYRHYTPTFKKGAKLVHKPILWHVNQTTTKSTFKPNMWCLRCLYSTKPVPTSNSFE	1993
GQ153542.1	VVAIDYRHYSTSFKKGAKLLHKPILWHINQTTNKTTYKPNIWCLRCLWSTKPVDTSNSFG	2001
DQ022305	VVAIDYRHYSTSFKKGAKLVHKPILWHINQTTNKTTYKPNIWCLRCLWSTKPVDTSNSFE	1997
KF569996.1	VVAIDYRHYSASFKKGAKLLHKPIVWHINQATTKTTFKPNTWCLRCLWSTKPVDTSNSFE	2003
KP886809.1	VVAIDYRHYSASFKKGAKLLHKPIVWHINQATTKTTFKPNTWCLRCLWSTKPVDTSNSFE	2003
AY278488.2	VVAIDYRHYSASFKKGAKLLHKPIVWHINQATTKTTFKPNTWCLRCLWSTKPVDTSNSFE	2003
AY485277.1	VVAIDYRHYSASFKKGAKLLHKPIVWHINQATTKTTFKPNTWCLRCLWSTKPVDTSNSFE	2003
AP006560.1	VVAIDYRHYSASFKKGAKLLHKPIVWHINQATTKTTFKPNTWCLRCLWSTKPVDTSNSFE	2003
AP006557.1	VVAIDYRHYSASFKKGAKLLHKPIVWHINQATTKTTFKPNTWCLRCLWSTKPVDTSNSFE	2003
AY274119	VVAIDYRHYSASFKKGAKLLHKPIVWHINQATTKTTFKPNTWCLRCLWSTKPVDTSNSFE	2003
AY572038.1	VVAIDYRHYSASFKKGAKLLHKPIVWHINQATTKTTFKPNTWCLRCLWSTKPVDTSNSFE	2003
AY572034.1	VVAIDYRHYSASFKKGAKLLHKPIVWHINQATTKTTFKPNTWCLRCLWSTKPVDTSNSFE	2003
FJ588686.1	--	1883
KY417145.1	VVAIDYRHYSASFKKGAKLLHKPIVWHINQATTKTTFKPNTWCLRCLWSTKPVDTSNSFE	2003
KY417144.1	VVAIDYRHYSASFKKGAKLLHKPIVWHINQATTKTTFKPNTWCLRCLWSTKPVDTSNSFE	2003
KY417147.1	VVAIDYRHYSASFKKGAKLLHKPIVWHINQATTKTTFKPNTWCLRCLWSTKPVDTSNSFE	2003
KY417148.1	VVAIDYRHYSASFKKGAKLLHKPIVWHINQATTKTTFKPNTWCLRCLWSTKPVDTSNSFE	2003
KY417143.1	VVAIDYRHYSASFKKGAKLLHKPIVWHINQATTKTTFKPNTWCLRCLWSTKPVDTSNSFE	2003
KT444582.1	VVAIDYRHYSASFKKGAKLLHKPIVWHINQATTKTTFKPNTWCLRCLWSTKPVDTSNSFE	2003
KC881005.1	VVAIDYRHYSASFKKGAKLLHKPIVWHINQATTKTTFKPNTWCLRCLWSTKPVDTSNSFE	2003
KC881006.1	VVAIDYRHYSASFKKGAKLLHKPIVWHINQATTKTTFKPNTWCLRCLWSTKPVDTSNSFE	2003
KF367457.1	VVAIDYRHYSASFKKGAKLLHKPIVWHINQATTKTTFKPNTWCLRCLWSTKPVDTSNSFE	2003
KY417152.1	VVAIDYRHYSASFKKGAKLLHKPIVWHINQATTKTTFKPNTWCLRCLWSTKPVDTSNSFE	2003
KY417146.1	VVAIDYRHYSASFKKGAKLLHKPIVWHINQATTKTTFKPNTWCLRCLWSTKPVDTSNSFE	2003
KY417151.1	VVAIDYRHYSASFKKGAKLLHKPIVWHINQATTKTTFKPNTWCLRCLWSTKPVDTSNSFE	2003
KY417142.1	VVAIDYRHYSASFKKGAKLLHKPIVWHINQATTKTTFKPNTWCLRCLWSTKPVDTSNSFE	2003

MW532698.1	VLQSEDTQGMETLACEDTKLVTEEVVETPTIQKDIVECDVKTTEVVGDVILKPVQDGVKI	2078
MT040336.1	VLQSEDTQGMETLACEDTKLVTEEVVETPTIQKDIVECDVKTTEVVGDVILKPAQDGVKI	2078
MT040335.1	VLQSEDTQGMETLACEDTKLVTEEVVETPTIQKDIVECDVKTTEVVGDVILKPAQDGVKI	2078
MT040334.1	VLQSEDTQGMETLACEDTKLVTEEVVETPTIQKDIVECDVKTTEVVGDVILKPAQDGVKI	2078
MT040333.1	VLQSEDTQGMETLACEDTKLVTEEVVETPTIQKDIVECDVKTTEVVGDVILKPAQDGVKI	2078
MN996532.2	VLKSEDTQGMDNLACEDLKPVSEEVVENPTIQKDILECNVKTTEVVGDIILKPANDGLKI	2085
MN988713.1	VLKSEDAQGMDNLACEDLKPVSEEVVENPTIQKDVLECNVKTTEVVGDIILKPANNSLKI	2086
MT093571.1	VLKSEDAQGMDNLACEDLKPVSEEVVENPTIQKDVLECNVKTTEVVGDIILKPANNSLKI	2086
MN996529.1	VLKSEDAQGMDNLACEDLKPVSEEVVENPTIQKDVLECNVKTTEVVGDIILKPANNSLKI	2086
MT072688.1	VLKSEDAQGMDNLACEDLKPVSEEVVENPTIQKDVLECNVKTTEVVGDIILKPANNSLKI	2086
NC_045512.2	VLKSEDAQGMDNLACEDLKPVSEEVVENPTIQKDVLECNVKTTEVVGDIILKPANNSLKI	2086
MN994467.1	VLKSEDAQGMDNLACEDLKPVSEEVVENPTIQKDVLECNVKTTEVVGDIILKPANNSLKI	2086
MG772933.1	ALELGDTQGMDNLACEVLKPVSEEVVENPTIQKDILECNVKTTEVVGDIILKPASDGLKI	2082
MG772934.1	ALKLGDTQGMDNLACEVLKPVSEEVVENPTIQKDILECNVKTTEVVGDIILKPAIDGLKI	2060
NC_014470.1	VLSSDDAQGMDNLACESQQTVAEEVVDNPTIQKDIIECDVKTTEVVGNVILKPSADGIKV	2053
GQ153542.1	VLVVEDTQGMDNLACESQTTTSEEVVENPTVQKEIIECDVKTTEVVGNVILKPSEEGVKV	2061
DQ022305	VLVVEDTQGMDNLACESQTTTSEEVVENPTVQKEIIECDVKTTEVVGNVILKPSEEGVKV	2057
KF569996.1	VLAVEDTQGMDNLACESQQPTSEEVVENPTVQKEVIECDVKTTEVVGNVILKPSDEGIKV	2063
KP886809.1	VLAVEDTQGMDNLACESQQPSSEEVVENPTIQKEVIECDVKTTEVVGNVILKPSDEGVKV	2063
AY278488.2	VLAVEDTQGMDNLACESQQPTSEEVVENPTIQKEVIECDVKTTEVVGNVILKPSDEGVKV	2063
AY485277.1	VLAVEDTQGMDNLACESQQPTSEEVVENPTIQKEVIECDVKTTEVVGNVILKPSDEGVKV	2063
AP006560.1	VLAVEDTQGMDNLACESQQPTSEEVVENPTIQKEVIECDVKTTEVVGNVILKPSDEGVKV	2063
AP006557.1	VLAVEDTQGMDNLACESQQPTSEEVVENPTIQKEVIECDVKTTEVVGNVILKPSDEGVKV	2063
AY274119	VLAVEDTQGMDNLACESQQPTSEEVVENPTIQKEVIECDVKTTEVVGNVILKPSDEGVKV	2063
AY572038.1	VLAVEDTQGMDNLACESQQPTSEEVVENPTIQKEVIECDVKTTEVVGNVILKPSDEGVKV	2063
AY572034.1	VLAVEDTQGMDNLACESQQPTSEEVVENPTIQKEVIECDVKTTEVVGNVILKPSDEGVKV	2063
FJ588686.1	--	1883
KY417145.1	VLAVEDTQGMDNLACESQQPTSEEVVENPTIQKEVIECDVKTTEVVGNVILKPSDEGVKV	2063
KY417144.1	VLAVEDTQGMDNLACESQQPTSEAVVENPTIQKEVLECDVKTTEVVGNVILKPSDEGVKV	2063
KY417147.1	VLAVEDTRGMDNLACESQQPTSEEVVENPTIQKEVIECDVKTTEVVGNVILKPSDEGVKV	2063
KY417148.1	VLAVEDTQGMDNLACESQQPTSEEVVENPTIQKEVIECDVKTTEVVGNVILKPSDEGVKV	2063
KY417143.1	VLAVEDTQGMDNLACESQQPTSEEVVENPTIQKEVIECDVKTTEVVGNVILKPSDEGVKV	2063
KT444582.1	VLAVEDTQGMDNLACESQQPTSEEVVENPTIQKEVIECDVKTTEVVGNVILKPSDEGVKV	2063
KC881005.1	VLAVEDTQGMDNLACESQQPTSEAVVENPTIQKEVLECDVKTTEVVGNVILKPSDEGVKV	2063
KC881006.1	VLAVEDTQGMDNLACESQQPTSEEVVENPTIQKEVIECDVKTTEVVGNVILKPSDEGVKV	2063
KF367457.1	VLAVEDTQGMDNLACESQQPTSEEVVENPTIQKEVIECDVKTTEVVGNVILKPSDEGVKV	2063
KY417152.1	VLAVEDTQGMDNLACESQQPTSEEVVENPTIQKEVIECDVKTTEVVGNVILKPSDEGVKV	2063
KY417146.1	VLAVEDTQGMDNLACESQQPTSEEVVENPTIQKEVLECDVKTTEVVGNVILKPSDEGVKV	2063
KY417151.1	VLAVEDTQGMDNLACESQQPTSEEVVENPTIQKEVIECDVKTTEVVGNVILKPSDEGVKV	2063
KY417142.1	VLAVEDTQGMDNLACESQQPTSEEVVENPTIQKEVLECDVKTTEVVGNVILKPSDEGVKV	2063

```
MW532698.1        TEEVGHEDLMAAYVDNTSLTIKKPNELSVMLGLKTLKIHGLAAVNSVPWDTIVTYAKPFL        2138
MT040336.1        TEEVGHEDLMAAYVDNTSLTIKKPNELSVMLGLKTLKTHGLAAVNSVPWDTIVTYAKPFL        2138
MT040335.1        TEEVGHEDLMAAYVDNTSLTIKKPNELSVMLGLKTLKTHGLAAVNSVPWDTIVTYAKPFL        2138
MT040334.1        TEEVGHEDLMAAYVDNTSLTIKKPNELSVMLGLKTLKTHGLAAVNSVPWDTIVTYAKPFL        2138
MT040333.1        TEEVGHEDLMAAYVDNTSLTIKKPNELSVMLGLKTLKTHGLAAVNSVPWDTIVTYAKPFL        2138
MN996532.2        TEEVGHTDLMAAYVDNSSLTIKKPNELSRVLGLKTLVTHGLAAVNSVPWDTIANYAKPFL        2145
MN988713.1        TEEVGHTDLMAAYVDNSSLTIKKPNELSRVLGLKTLATHGLAAVNSVPWDTIANYAKPFL        2146
MT093571.1        TEEVGHTDLMAAYVDNSSLTIKKPNELSRVLGLKTLATHGLAAVNSVPWDTIANYAKPFL        2146
MN996529.1        TEEVGHTDLMAAYVDNSSLTIKKPNELSRVLGLKTLATHGLAAVNSVPWDTIANYAKPFL        2146
MT072688.1        TEEVGHTDLMAAYVDNSSLTIKKPNELSRVLGLKTLATHGLAAVNSVPWDTIANYAKPFL        2146
NC_045512.2       TEEVGHTDLMAAYVDNSSLTIKKPNELSRVLGLKTLATHGLAAVNSVPWDTIANYAKPFL        2146
MN994467.1        TEEVGHTDLMAAYVDNSSLTIKKPNELSRVLGLKTLATHGLAAVNSVPWDTIANYAKPFL        2146
MG772933.1        TKEVGHTDLMAAYVDNSSLTIKKPNELSRVLGLKTLATHGLAAINSVPWDTIANYVKPFL        2142
MG772934.1        TEEVGHTDLMAAYVDNSSLTIKKPSELSRVLGLKTLATHGLAAINSVPWDTIANYAKPFL        2120
NC_014470.1       TSELEHEDLMAAYVNETSITIKKPNELSIMLGLKTIATHGAAAINSVPWIKICAYVKPFL        2113
GQ153542.1        TQELGHEDLMAAYVEETSITIKKPNELSLALGLKTLATHGAAAINSVPWSKILAYVKPFL        2121
DQ022305          TQELGHEDLMAAYVEETSITIKKPNELSLALGLKTLATHGAAAINSVPWSKILAYVKPFL        2117
KF569996.1        TQELGHEDLMAAYVENTSITIKKPNELSLALGLKTIATHGIAAVNSVPWSKILAYVKPFL        2123
KP886809.1        TQELGHEDLMAAYVENTSITIKKPNELSLALGLKTIATHGIAAVNSVPWSKILAYVKPFL        2123
AY278488.2        TQELGHEDLMAAYVENTSITIKKPNELSLALGLKTIATHGIAAINSVPWSKILAYVKPFL        2123
AY485277.1        TQELGHEDLMAAYVENTSITIKKPNELSLALGLKTIATHGIAAINSVPWSKILAYVKPFL        2123
AP006560.1        TQELGHEDLMAAYVENTSITIKKPNELSLALGLKTIATHGIAAINSVPWSKILAYVKPFL        2123
AP006557.1        TQELGHEDLMAAYVENTSITIKKPNELSLALGLKTIATHGIAAINSVPWSKILAYVKPFL        2123
AY274119          TQELGHEDLMAAYVENTSITIKKPNELSLALGLKTIATHGIAAINSVPWSKILAYVKPFL        2123
AY572038.1        TQELGHEDLMAAYVENTSITIKKPNELSLALGLKTIATHGIAAINSVPWSKILAYVKPFL        2123
AY572034.1        TQELGHEDLMAAYVENTSITIKKPNELSLALGLKTIATHGIAAINSVPWSKILAYVKPFL        2123
FJ588686.1        -------------VENTSITIKKPNELSLALGLKTIATHGIAAINSVPWSKILAYVKPFL        1930
KY417145.1        TQELGHEDLMAAYVENTSITIKKPNELSLALGLKTIATHGIAAINSVPWSKIFAYVKPFL        2123
KY417144.1        TQELGHEDLMAAYVENTSITIKKPNELSLALGLKTIATHGIAAINSVPWSKILAYVKPFL        2123
KY417147.1        TQELDHEDLMAAYVENTSITIKKPNELSLALGLKTIVTHGIAAINSVPWSKILAYVKPFL        2123
KY417148.1        TQELDHEDLMAAYVENTSITIKKPNELSLALGLKTIATHGIAAINSVPWSKILAYVKPFL        2123
KY417143.1        TQELDHEDLMAAYVENTSITIKKPNELSLALGLKTIATHGIAAINSVPWSKILAYVKPFL        2123
KT444582.1        TQELDHEDLMAAYVENTSITIKKPNELSLALGLKTIATHGIAAINSVPWSKILAYVKPFL        2123
KC881005.1        TQELGHEDLMAAYVENTSITIKKPNELSLALGLKTIATHGIAAINSVPWSKILAYVKPFL        2123
KC881006.1        TQELDHEDLMAAYVENTSITIKKPNELSLALGLKTIATHGIAAINSVPWSKILAYVKPFL        2123
KF367457.1        TQELDHEDLMAAYVENTSITIKKPNELSLALGLKTIATHGIAAINSVPWSKILAYVKPFL        2123
KY417152.1        TQELGHEDLMAAYVENTSITIKKPNELSLALGLKTIATHGIAAINSVPWSKILAYVKPFL        2123
KY417146.1        TQELGHEDLMAAYVENTSITIKKPNELSLALGLKTIATHGIAAINSVPWSKILAYVKPFL        2123
KY417151.1        TQELGHEDLMAAYVENTSITIKKPNELSLALGLKTIATHGIAAINSVPWSKILAYVKPFL        2123
KY417142.1        TQELGHEDLMAAYVENTSITIKKPNELSLALGLKTIATHGIAAINSVPWSKILAYVKPFL        2123
                               *:::*:*****.***  *****:  ** **:***** .*  *.****
```

```
MW532698.1      NKVTSVAASGVARCLNRICVNYMPYVLTLLLQFCTFTRSTNSRIKASMPTTIAKNTVKSV    2198
MT040336.1      NKVTSVAASGVARCLNRICVNYMPYVLTLLLQFCTFTRSTNSRIKASMPTTIAKNTVKSV    2198
MT040335.1      NKVTSVAASGVARCLNRICVNYMPYVLTLLLQFCTFTRSTNSRIKASMPTTIAKNTVKSV    2198
MT040334.1      NKVTSVAASGVARCLNRICVNYMPYVLTLLLQFCTFTRSTNSRIKASMPTTIAKNTVKSV    2198
MT040333.1      NKVTSVAASGVARCLNRICVNYMPYVLTLLLQFCTFTRSTNSRIKASMPTTIAKNTVKSV    2198
MN996532.2      NKVVSTTTNIVTRCLNRVCTNYMPYFFTLLLQLCTFTRSTNSRIKASMPTTIAKNTVKSV    2205
MN988713.1      NKVVSTTTNIVTRCLNRVCTNYMPYFFTLLLQLCTFTRSTNSRIKASMPTTIAKNTVKSV    2206
MT093571.1      NKVVSTTTNIVTRCLNRVCTNYMPYFFTLLLQLCTFTRSTNSRIKASMPTTIAKNTVKSV    2206
MN996529.1      NKVVSTTTNIVTRCLNRVCTNYMPYFFTLLLQLCTFTRSTNSRIKASMPTTIAKNTVKSV    2206
MT072688.1      NKVVSTTTNIVTRCLNRVCTNYMPYFFTLLLQLCTFTRSTNSRIKASMPTTIAKNTVKSV    2206
NC_045512.2     NKVVSTTTNIVTRCLNRVCTNYMPYFFTLLLQLCTFTRSTNSRIKASMPTTIAKNTVKSV    2206
MN994467.1      NKVVSTTTNIVTRCLNRVCTNYMPYFFTLLLQLCTFTRSTNSRIKASMPTTIAKNTVKSV    2206
MG772933.1      NKVVSTTTNIVTRCLNRVCTNYMPYLFTLLLQLCTFTKSTNSRIRASMPTTIAKNTVKSV    2202
MG772934.1      NKVVSTTTNIVTRCLNRVCTNYMPYLFTLLLQLCTFTKSTTSRIRASMPTTIAKNTVKSV    2180
NC_014470.1     GYVAEQSKNCIKRCFRRVFNDYMPFLLTLLLQLCTFTKSTNFRIKAAMPIVIARNSVIGG    2173
GQ153542.1      GQTAVITSNCIKKCVQRVFSNYMPYVITLLFQLCTFTKSTNSRIKASLPTTIAKNSVKSV    2181
DQ022305        GQTAVITSNCIKKCVQRVFSNYMPYVITLLFQLCTFTKSTNSRIKASLPTTIAKNSVKSV    2177
KF569996.1      GQAATTTSNCAKRLVQRVFNNYMPYVFTLLFQLCTFTKSTNSRIRASLPTTIAKNSVKSV    2183
KP886809.1      GQAATTTSNCARRLVQRVFNNYMPYVFTLLFQLCTFTKSTNSRIRASLPTTIAKNSVKSV    2183
AY278488.2      GQAAITTSNCAKRLAQRVFNNYMPYVFTLLFQLCTFTKSTNSRIRASLPTTIAKNSVKSV    2183
AY485277.1      GQAAITTSNCAKRLAQRVFNNYMPYVFTLLFQLCTFTKSTNSRIRASLPTTIAKNSVKSV    2183
AP006560.1      GQAAITTSNCAKRLAQRVFNNYMPYVFTLLFQLCTFTKSTNSRIRASLPTTIAKNSVKSV    2183
AP006557.1      GQAAITTSNCAKRLAQRVFNNYMPYVFTLLFQLCTFTKSTNSRIRASLPTTIAKNSVKSV    2183
AY274119        GQAAITTSNCAKRLAQRVFNNYMPYVFTLLFQLCTFTKSTNSRIRASLPTTIAKNSVKSV    2183
AY572038.1      GQAAITTSNCAKRLAQRVFNNYMPYVFTLLFQLCTFTKSTNSRIRASLPTTIAKNSVKSV    2183
AY572034.1      GQAAITTSNCAKRLAQRVFNNYMPYVFTLLFQLCTFTKSTNSRIRASLPTTIAKNSVKSV    2183
FJ588686.1      GQAAITTSNCAKRLAQRVFNNYMPYVLTLLFQLCTFTKSTNSRIRASLPTTIAKNSVKGV    1990
KY417145.1      GQAAITTSNCAKRLAQRVFNNYMPYVLTLLFQLCTFTKSTNSRIRASLPTTIAKNSVRGV    2183
KY417144.1      GQTAVTTSNCAKRLVQRMFNNYMPYVLTLLFQLCTFTKSTNSRIRASLPTTIAKNSVKGV    2183
KY417147.1      GQAAITTSNCAKRLAQRVFNNYMPYVLTLLFQLCTFTKSTNSRIRASLPTTIAKNSVRGV    2183
KY417148.1      GQAAVTTSNCAKRLVQRMFNNYMPYVLTLLFQLCTFTKSTNSRIRASLPTTIAKNSVRGV    2183
KY417143.1      GQAAVTTSNCAKRLVQRMFNNYMPYVLTLLFQLCTFTKSTNSRIRASLPTTIAKNSVRGV    2183
KT444582.1      GQAAVTTSNCAKRLVQRMFNNYMPYVLTLLFQLCTFTKSTNSRIRASLPTTIAKNSVKGV    2183
KC881005.1      GQAAVTTSNCAKRLVQRMFNNYMPYVLTLLFQLCTFTKSTNSRIRASLPTTIAKNSVKGV    2183
KC881006.1      GQAAITTSNCAKRLAQRVFNNYMPYVLTLLFQLCTFTKSTNSRIRASLPTTIAKNSVRGV    2183
KF367457.1      GQAAITTSNCAKRLAQRVFNNYMPYVLTLLFQLCTFTKSTNSRIRASLPTTIAKNSVRGV    2183
KY417152.1      GQAAITTSNCAKRLAQRVFNNYMPYVLTLLFQLCTFTKSTNSRIRASLPTTIAKNSVRGV    2183
KY417146.1      GQAAITTSNCAKRLAQCVFNNYMPYVLTLLFQLCTFTKSTNSRIRASLPTTIAKNSVKGV    2183
KY417151.1      GQAAITTSNCAKRLAQRVFNNYMPYVLTLLFQLCTFTKSTNSRIRASLPTTIAKNSVRGV    2183
KY417142.1      GQAAITTSNCAKRLAQCVFNNYMPYVLTLLFQLCTFTKSTNSRIRASLPTTIAKNSVKGV    2183
                . ..  : .   :  . :  :***:.:***:*:****:**. **:*::* .**:*:* .
```

```
MW532698.1      GKFCLEASFNYLKSPNFSKLITIIVWFLLLSVCLGSLIYSSAALGVLMSNLGMPSYCTSY     2258
MT040336.1      GKFCLEASFNYLKSPNFSKLITIIVWFLLLSVCLGSLIYSSAALGVLMSNLGMPSYCTSY     2258
MT040335.1      GKFCLEASFNYLKSPNFSKLITIIVWFLLLSVCLGSLIYSSAALGVLMSNLGMPSYCTSY     2258
MT040334.1      GKFCLEASFNYLKSPNFSKLITIIVWFLLLSVCLGSLIYSSAALGVLMSNLGMPSYCTSY     2258
MT040333.1      GKFCLEASFNYLKSPNFSKLITIIVWFLLLSVCLGSLIYSSAALGVLMSNLGMPSYCTSY     2258
MN996532.2      GKFCLEASFNYLKSPNFFKLINIIIWFLLLSVCLGSLIYSTAALGVLMSNLGMPSYCTGY     2265
MN988713.1      GKFCLEASFNYLKSPNFSKLINIIIWFLLLSVCLGSLIYSTAALGVLMSNLGMPSYCTGY     2266
MT093571.1      GKFCLEASFNYLKSPNFSKLINIIIWFLLLSVCLGSLIYSTAALGVLMSNLGMPSYCTGY     2266
MN996529.1      GKFCLEASFNYLKSPNFSKLINIIIWFLLLSVCLGSLIYSTAALSVLMSNLGMPSYCTGY     2266
MT072688.1      GKFCLEASFNYLKSPNFSKLINIIIWFLLLSVCLGSLIYSTAALGVLMSNLGMPSYCTGY     2266
NC_045512.2     GKFCLEASFNYLKSPNFSKLINIIIWFLLLSVCLGSLIYSTAALGVLMSNLGMPSYCTGY     2266
MN994467.1      GKFCLEASFNYLKSPNFSKLINIIIWFLLLSVCLGSLIYSTAALGVLMSNLGMPSYCTGY     2266
MG772933.1      GKFCIEASFNYLKSPNFSKLINIVIWFLLLSVCLGSLIYSTAALGVLMSNLGMPSYCTVY     2262
MG772934.1      GKFCIEASFNYLKSPNFSKLINIVIWFLLLSVCLGSLIYSTAALGVLMSNLGMPSYCTVY     2240
NC_014470.1     VRFCLDALTMYVKSPKFSGILTVVMWLLLLSVCLGCLVYAVASFGAILSGFGLMSYCDGV     2233
GQ153542.1      AKLCLDVCINYVKSPKFSKLFTIVMWLLLLSTCLGSLTYVTAALGVCLSSLGVPSYCDGV     2241
DQ022305        AKLCLDVCINYVKSPKFSKLFTIVMWLLLLSICLGSLTYVTAVLGVCLSSLGVPSYCDGV     2237
KF569996.1      AKLCLEAGINYVKSPKFSKLFTIAMWLLLLSICLGSLIYVTAAFGVLLSNFGAPSYCNGV     2243
KP886809.1      VKLCLDAGINYVKSPKFSKLFTIAMWLLLLSICLGFLIYVIAAFGVLLSNFGAPSYCNGV     2243
AY278488.2      AKLCLDAGINYVKSPKFSKLFTIAMWLLLLSICLGSLICVTAAFGVLLSNFGAPSYCNGV     2243
AY485277.1      AKLCLDAGINYVKSPKFSKLFTIAMWLLLLSICLGSLICVTAAFGVLLSNFGAPSYCNGV     2243
AP006560.1      AKLCLDAGINYVKSPKFSKLFTIAMWLLLLSICLGSLICVTAAFGVLLSNFGAPSYCNGV     2243
AP006557.1      AKLCLDAGINYVKSPKFSKLFTIAMWLLLLSICLGSLICVTAAFGVLLSNFGAPSYCNGV     2243
AY274119        AKLCLDAGINYVKSPKFSKLFTIAMWLLLLSICLGSLICVTAAFGVLLSNFGAPSYCNGV     2243
AY572038.1      AKLCLDAGINYVKSPKFSKLFTIAMWLLLLSICLGSLICVTAAFGVLLSNFGAPSYCNGV     2243
AY572034.1      AKLCLDAGINYVKSPKFSKLFTIAMWLLLLSICLGSLICVTAAFGVLLSNFGAPSYCNGV     2243
FJ588686.1      ARLCLDAGINYVKSPKFSKLFTIAMWLLLLSICLGSLIYVTAALGVLLSNFGAPSYCSGV     2050
KY417145.1      ARLCLDAGINYIKSPKFSKLFTIATWLLLLSICLGSLIYVTAALGVLLSNFGAPSYCSGV     2243
KY417144.1      ARLCLDAGINYVKSPKFSKLFTIAMWLLLLSICLGSLIYVTAALGVLLSNVGAPSYCSGV     2243
KY417147.1      ARLCLDAGINYVKSPKFSKLFTIAMWLLLLSICLGSLIYVTAALGVLLSNFGAPSYCSGV     2243
KY417148.1      ARLCLDAGINYVKSPKFSKLFTIAMWLLLLSICLGSLIYVTAALGVLLSNFGAPSYCSGV     2243
KY417143.1      ARLCLDAGINYVKSPKFSKLFTIAMWLLLLSICLGSLIYVTAALGVLLSNFGAPSYCSGV     2243
KT444582.1      ARLCLDAGINYVKSPKFSKLFTVAMWLLLLSICLGSLIYVTAALGVLLSNFGAPSYCSGV     2243
KC881005.1      ARLCLDAGINYVKSPKFSKLFTIAMWLLLLSICLGSLIYVTAALGVLLSNVGAPSYCSGV     2243
KC881006.1      ARLCLDAGINYVKSPKFSKLFTIAMWLLLLSICLGSLIYVTAALGVLLSNFGAPSYCSGV     2243
KF367457.1      ARLCLDAGINYVKSPKFSKLFTIAMWLLLLSICLGSLIYVTAALGVLLSNFGAPSYCSGV     2243
KY417152.1      ARLCLDAGINYVKSPKFSKLFTIAMWLLLLSICLGSLIYVTAALGVLLSNFGAPSYCSGV     2243
KY417146.1      ARLCLDAGINYVKSPKFSKLFTIAMWLLLLSICLGSLIYVTAALGVLLSNFGAPSYCSGV     2243
KY417151.1      ARLCLDAGINYVKSPKFSKLFTIAMWLLLLSICLGSLIYVTAALGVLLSNFGAPSYCSGV     2243
KY417142.1      ARLCLDAVINYVKSPKFSKLFTIAMWLLLLSICLGSLIYVTAALGVLLSNFGAPSYCSGV     2243
                 ::*::.   *:***:*  ::.:  *:**** *** *    * :.. :*..*  ***
```

```
MW532698.1      RDGYLNSTNVTTTAYCTGSIPCSVCLSGMDSLDAYPALETIQVTISSFKWDLTAFGIIAE    2318
MT040336.1      RDGYLNSTNVTTTAYCTGSIPCSVCLSGMDSLDAYPALETIQVTISSFKWDLTAFGIIAE    2318
MT040335.1      RDGYLNSTNVTTTAYCTGSIPCSVCLSGMDSLDAYPALETIQVTISSFKWDLTAFGIIAE    2318
MT040334.1      RDGYLNSTNVTTTAYCTGSIPCSVCLSGMDSLDAYPALETIQVTISSFKWDLTAFGIIAE    2318
MT040333.1      RDGYLNSTNVTTTAYCTGSIPCSVCLSGMDSLDAYPALETIQVTISSFKWDLTAFGIIAE    2318
MN996532.2      REGYLNSTNVTTAIYCTGSIPCSVCLSGLDSLDTYPSLETIQITISSFKWDLTAFGLVAE    2325
MN988713.1      REGYLNSTNVTIATYCTGSIPCSVCLSGLDSLDTYPSLETIQITISSFKWDLTAFGLVAE    2326
MT093571.1      REGYLNSTNVTIATYCTGSIPCSVCLSGLDSLDTYPSLETIQITISSFKWDLTAFGLVAE    2326
MN996529.1      REGYLNSTNVTIATYCTGSIPCSVCLSGLDSLDTYPSLETIQITISSFKWDLTAFGLVAE    2326
MT072688.1      REGYLNSTNVTIATYCTGSIPCSVCLSGLDSLDTYPSLETIQITISSFKWDLTAFGLVAE    2326
NC_045512.2     REGYLNSTNVTIATYCTGSIPCSVCLSGLDSLDTYPSLETIQITISSFKWDLTAFGLVAE    2326
MN994467.1      REGYLNSTNVTIATYCTGSIPCSVCLSGLDSLDTYPSLETIQITISSFKWDLTAFGLVAE    2326
MG772933.1      REGYLNSTNVTTATYCTGSIPCSVCLSGLDSLDTYPSLETIQITISSFKWDLTAFGLVAE    2322
MG772934.1      REGYLNSTNVTIATYCTGSIPCSVCLSGLDSLDTYPSLETIQITISSFKWDLTAFGLVAE    2300
NC_014470.1     RAGYVNSSNVTIPDYCAGSLPCGVCLGGLDSLDAYPALETIQVTISSYKLDLTFVGMMAE    2293
GQ153542.1      RELYINSSNVTTMDFCQGSFPCSVCLSGLDSLDSYPALETIQVTISSYKLDLTFLGLAAE    2301
DQ022305        RELYINSSNVTTMDFCQGYFPCSVCLSGLDSLDSYPALETIQVTISSYKLDLTFLGLAAE    2297
KF569996.1      RELYLNSSNVTTMDFCEGSFPCSVCLSGLDSLDSYPALETIQVTISSYKLDLTFLGLAAE    2303
KP886809.1      RELYLNSSNVTTMDFCEGSFPCSICLSGLDSLDSYPALETIQVTISSYKLDLTILGLAAE    2303
AY278488.2      RELYLNSSNVTTMDFCEGSFPCSICLSGLDSLDSYPALETIQVTISSYKLDLTILGLAAE    2303
AY485277.1      RELYLNSSNVTTMDFCEGSFPCSICLSGLDSLDSYPALETIQVTISSYKLDLTILGLAAE    2303
AP006560.1      RELYLNSSNVTTMDFCEGSFPCSICLSGLDSLDSYPALETIQVTISSYKLDLTILGLAAE    2303
AP006557.1      RELYLNSSNVTTMDFCEGSFPCSICLSGLDSLDSYPALETIQVTISSYKLDLTILGLAAE    2303
AY274119        RELYLNSSNVTTMDFCEGSFPCSICLSGLDSLDSYPALETIQVTISSYKLDLTILGLAAE    2303
AY572038.1      RELYLNSSNVTTMDFCEGSFPCSICLSGLDSLDSYPALETIQVTISSYKLDLTILGLAAE    2303
AY572034.1      RELYLNSSNVTTMDFCEGSFPCSICLSGLDSLDSYPALETIQVTISSYKLDLTILGLAAE    2303
FJ588686.1      RELYLSSSNVTTMDFCEGSFPCSVCLSGLDSLDSYPALETIQVTISSYKLDLTILGLAAE    2110
KY417145.1      RESYLNSSNVTTMDFCEGSFPCSVCLSGLDSLDSYPALETIQVTISSYKLDLTILGLAAE    2303
KY417144.1      RESYLNSSNVTTMDFCEGSFPCSVCLSGLDSLDSYPALETIQVTISSYKLDLTILGLAAE    2303
KY417147.1      RESYLNSSNVTTMDFCEGSFPCSVCLSGLDSLDSYPALETIQVTISSYKLDLTILGLAAE    2303
KY417148.1      RESYLNSSNVTTMDFCEGSFPCSVCLSGLDSLDSYPALETIQVTISSYKLDLTILGLAAE    2303
KY417143.1      RESYLNSSNVTTMDFCEGSFPCSVCLSGLDSLDSYPALETIQVTISSYKLDLTILGLAAE    2303
KT444582.1      RESYLNSSNVTTMDFCEGSFPCSVCLSGLDSLDSYPALETIQVTISSYKLDLTILGLAAE    2303
KC881005.1      RESYLNSSNVTTMDFCEGSFPCSVCLSGLDSLDSYPALETIQVTISSYKLDLTILGLAAE    2303
KC881006.1      RESYLNSSNVTTMDFCEGSFPCSVCLSGLDSLDSYPALETIQVTISSYKLDLTILGLAAE    2303
KF367457.1      RESYLNSSNVTTMDFCEGSFPCSVCLSGLDSLDSYPALETIQVTISSYKLDLTILGLAAE    2303
KY417152.1      RESYLNSSNVTTMDFCEGSFPCSVCLSGLDSLDSYPALETIQVTISSYKLDLTILGLAAE    2303
KY417146.1      RESYLNSSNVTTMDFCEGSFPCSVCLSGLDSLDSYPALETIQVTISSYKLDLTILGLAAE    2303
KY417151.1      RESYLNSSNVTTMDFCEGSFPCSVCLSGLDSLDSYPALETIQVTISSYKLDLTILGLAAE    2303
KY417142.1      RESYLNSSNVTTMDFCEGSFPCSVCLSGLDSLDSYPALETIQVTISSYKLDLTILGLAAE    2303
                *  *:.*:***   :* * :**.:**.*:****:**:*****:****:* *** .*: **
```

```
MW532698.1        WCLAYILFTRFFYVLGLAAIMQLFFGYFAVHFISNSWLMWLIINLVQMAPISAMVRMYIF        2378
MT040336.1        WCLAYILFTRFFYVLGLAAIMQLFFGYFAVHFISNSWLMWLIINLVQMAPISAMVRMYIF        2378
MT040335.1        WCLAYILFTRFFYVLGLAAIMQLFFGYFAVHFISNSWLMWLIINLVQMAPISAMVRMYIF        2378
MT040334.1        WCLAYILFTRFFYVLGLAAIMQLFFGYFAVHFISNSWLMWLIINLVQMAPISAMVRMYIF        2378
MT040333.1        WCLAYILFTRFFYVLGLAAIMQLFFGYFAVHFISNSWLMWLIINLVQMAPISAMVRMYIF        2378
MN996532.2        WFLAYILFTRFFYVLGLAAIMQLFFSYFAVHFISNSWLMWLIINLVQMAPISAMVRMYIF        2385
MN988713.1        WFLAYILFTRFFYVLGLAAIMQLFFSYFAVHFISNSWLMWLIINLVQMAPISAMVRMYIF        2386
MT093571.1        WFLAYILFTRFFYVLGLAAIMQLFFSYFAVHFISNSWLMWLIINLVQMAPISAMVRMYIF        2386
MN996529.1        WFLAYILFTRFFYVLGLAAIMQLFFSYFAVHFISNSWLMWLIINLVQMAPISAMVRMYIF        2386
MT072688.1        WFLAYILFTRFFYVLGLAAIMQLFFSYFAVHFISNSWLMWLIINLVQMAPISAMVRMYIF        2386
NC_045512.2       WFLAYILFTRFFYVLGLAAIMQLFFSYFAVHFISNSWLMWLIINLVQMAPISAMVRMYIF        2386
MN994467.1        WFLAYILFTRFFYVLGLAAIMQLFFSYFAVHFISNSWLMWLIINLVQMAPISAMVRMYIF        2386
MG772933.1        WFLAYILFTRFFYVLGLAAIMQLFFSYFAVHFISNSWLMWLIINLVQMAPISAMVRMYIF        2382
MG772934.1        WFLAYILFTRFFYVLGLAAIMQLFFSYFAVHFISNSWLMWLIINLVQMAPISAMVRMYIF        2360
NC_014470.1       WFLAYMLFTKFFYLLGLFALMQLFFGLFATHFVNNSWLMWLIINVVQMAPISAMVRMYVF        2353
GQ153542.1        WLLAYMLFTKFFYLLGLSAIMQVFFGYFASHFISNSWLMWFIISIVQMAPVSAMVRMYIF        2361
DQ022305          WLLAYMLFTKFFYLLGLSAIMQAFFGYFASHFISNSWLMWFIISIVQMAPVSAMVRMYIF        2357
KF569996.1        WFLAYMLFTKFFYLLGLSAVMQVFFGYFASHFISNSWLMWFIISIVQMAPVSAMVRMYIF        2363
KP886809.1        WVLAYMLFTKFFYLLGLSAIMQVFFGYFASHFISNSWLMWFIISIVQMAPVSAMVRMYIF        2363
AY278488.2        WVLAYMLFTKFFYLLGLSAIMQVFFGYFASHFISNSWLMWFIISIVQMAPVSAMVRMYIF        2363
AY485277.1        WVLAYMLFTKFFYLLGLSAIMQVFFGYFASHFISNSWLMWFIISIVQMAPVSAMVRMYIF        2363
AP006560.1        WVLAYMLFTKFFYLLGLSAIMQVFFGYFASHFISNSWLMWFIISIVQMAPVSAMVRMYIF        2363
AP006557.1        WVLAYMLFTKFFYLLGLSAIMQVFFGYFASHFISNSWLMWFIISIVQMAPVSAMVRMYIF        2363
AY274119          WVLAYMLFTKFFYLLGLSAIMQVFFGYFASHFISNSWLMWFIISIVQMAPVSAMVRMYIF        2363
AY572038.1        WVLAYMLFTKFFYLLGLSAIMQVFFGYFASHFISNSWLMWFIISIVQMAPVSAMVRMYIF        2363
AY572034.1        WVLAYMLFTKFFYLLGLSAIMQVFFGYFASHFISNSWLMWFIISIVQMAPVSAMVRMYIF        2363
FJ588686.1        WVLAYMLFTKFFYLLGLSAIMQVFFGYFASHFISNSWLMWFIISIVQMAPVSAMVRMYIF        2170
KY417145.1        WFLAYMLFTKFFYLLGLSAIMQVFFGYFASHFISNSWLMWFIISIVQMAPVSAMVRMYIF        2363
KY417144.1        WFLAYMLFTKFFYLLGLSAIMQVFFGYFASHFISNSWLMWFIISIVQMAPVSAMVRMYIF        2363
KY417147.1        WFLAYMLFTKFFYLLGLSAIMQVFFGYFASHFISNSWLMWFIISIVQMAPVSAMVRMYIF        2363
KY417148.1        WFLAYMLFTKFFYLLGLSAIMQVFFGYFASHFISNSWLMWFIISIVQMAPVSAMVRMYIF        2363
KY417143.1        WFLAYMLFTKFFYLLGLSAIMQVFFGYFASHFISNSWLMWFIISIVQMAPVSAMVRMYIF        2363
KT444582.1        WFLAYMLFTKFFYLLGLSAIMQVFFGYFASHFISNSWLMWFIISIVQMAPVSAMVRMYIF        2363
KC881005.1        WVLAYMLFTKFFYLLGLSAIMQVFFGYFASHFISNSWLMWFIISIVQMAPVSAMVRMYIF        2363
KC881006.1        WFLAYMLFTKFFYLLGLSAIMQVFFGYFASHFISNSWLMWFIISIVQMAPVSAMVRMYIF        2363
KF367457.1        WFLAYMLFTKFFYLLGLSAIMQVFFGYFASHFISNSWLMWFIISIVQMAPVSAMVRMYIF        2363
KY417152.1        WFLAYMLFTKFFYLLGLSAIMQVFFGYFASHFISNSWLMWFIISIVQMAPVSAMVRMYIF        2363
KY417146.1        WFLAYMLFTKFFYLLGLSAIMQVFFGYFASHFISNSWLMWFIISIVQMAPVSAMVRMYIF        2363
KY417151.1        WFLAYMLFTKFFYLLGLSAIMQVFFGYFASHFISNSWLMWFIISIVQMAPVSAMVRMYIF        2363
KY417142.1        WFLAYMLFTKFFYLLGLSAIMQVFFGYFASHFISNSWLMWFIISIVQMAPVSAMVRMYIF        2363
                  * ***:***:***:*** *:** **. ** **:.******:**.:*****:*******:*
```

```
MW532698.1      FASFYYVWKSYIHVVDGCTSSTCMMCYKRNRATRVECTTIVNGVRRSFYVYANGGKGFCK      2438
MT040336.1      FASFYYVWKSYIHVVDGCTSSTCMMCYKRNRATRVECTTIVNGVRRSFYVYANGGKGFCK      2438
MT040335.1      FASFYYVWKSYIHVVDGCTSSTCMMCYKRNRATRVECTTIVNGVRRSFYVYANGGKGFCK      2438
MT040334.1      FASFYYVWKSYIHVVDGCTSSTCMMCYKRNRATRVECTTIVNGVRRSFYVYANGGKGFCK      2438
MT040333.1      FASFYYVWKSYIHVVDGCTSSTCMMCYKRNRATRVECTTIVNGVRRSFYVYANGGKGFCK      2438
MN996532.2      FASFYYVWKSYVHVVDGCTSSTCMMCYKRNRATRVECTTIVNGVRRSFYVYANGGKGFCK      2445
MN988713.1      FASFYYVWKSYVHVVDGCNSSTCMMCYKRNRATRVECTTIVNGVRRSFYVYANGGKGFCK      2446
MT093571.1      FASFYYVWKSYVHVVDGCNSSTCMMCYKRNRATRVECTTIVNGVRRSFYVYANGGKGFCK      2446
MN996529.1      FASFYYVWKSYVHVVDGCNSSTCMMCYKRNRATRVECTTIVNGVRRSFYVYANGGKGFCK      2446
MT072688.1      FASFYYVWKSYVHVVDGCNSSTCMMCYKRNRATRVECTTIVNGVRRSFYVYANGGKGFCK      2446
NC_045512.2     FASFYYVWKSYVHVVDGCNSSTCMMCYKRNRATRVECTTIVNGVRRSFYVYANGGKGFCK      2446
MN994467.1      FASFYYVWKSYVHVVDGCNSSTCMMCYKRNRATRVECTTIVNGVRRSFYVYANGGKGFCK      2446
MG772933.1      FASFYYVWKSYVHVVDGCTSSTCMMCYKRNRATRVECTTIVNGVRRSFYVYANGGKGFCK      2442
MG772934.1      FASFYYVWKSYVHVVDGCTSSTCMMCYKRNRATRVECTTIVNGVRRSFYVYANGGKGFCK      2420
NC_014470.1     FASFYYVWKAYIHVINGCTSSTCIMCYKRNRATRVECTTIVNGMKKSFYVYANGGQGFCK      2413
GQ153542.1      FASFYYVWKSYVHIMDGCTSSTCMMCYKRNRATRVECTTIVNGVKRSFYVYANGGRGFCK      2421
DQ022305        FASFYYVWKSYVHIMDGCTSSTCMMCYKRNRATRVECTTIVNGVKRSFYVYANGGRGFCK      2417
KF569996.1      FASFYYIWKSYVHIMDGCTSSTCMMCYKRNRATRVECTTIVNGMKRSFYVHANGGRGFCK      2423
KP886809.1      FASFYYIWKSYVHIMDGCTSSTCMMCYKRNRATRVECTTIVNGMKRSFYVYANGGRGFCK      2423
AY278488.2      FASFYYIWKSYVHIMDGCTSSTCMMCYKRNRATRVECTTIVNGMKRSFYVYANGGRGFCK      2423
AY485277.1      FASFYYIWKSYVHIMDGCTSSTCMMCYKRNRATRVECTTIVNGMKRSFYVYANGGRGFCK      2423
AP006560.1      FASFYYIWKSYVHIMDGCTSSTCMMCYKRNRATRVECTTIVNGMKRSFYVYANGGRGFCK      2423
AP006557.1      FASFYYIWKSYVHIMDGCTSSTCMMCYKRNRATRVECTTIVNGMKRSFYVYANGGRGFCK      2423
AY274119        FASFYYIWKSYVHIMDGCTSSTCMMCYKRNRATRVECTTIVNGMKRSFYVYANGGRGFCK      2423
AY572038.1      FASFYYIWKSYVHIMDGCTSSTCMMCYKRNRATRVECTTIVNGMKRSFYVYANGGRGFCK      2423
AY572034.1      FASFYYIWKSYVHIMDGCTYSTCMMCYKRNRATRVECTTIVNGMKRSFYVYANGGRGFCK      2423
FJ588686.1      FASFYYIWKSYVHIMDGCTSSTCMMCYKRNRATRVECTTIVNGMKRSFYVYANGGRGFCK      2230
KY417145.1      FASFYYIWKSYVHIMDGCTSSTCMMCYKRNRATRVECTTIVNGMKRSFYVYANGGRGFCK      2423
KY417144.1      FASFYYIWKSYVHIMDGCNSSTCMMCYKRNRATRVECTTIVNGMKRSFYVYANGGRGFCK      2423
KY417147.1      FASFYYIWKSYVHIMDGCTSSTCMMCYKRNRATRVECTTIVNGMKRSFYVYANGGRGFCK      2423
KY417148.1      FASFYYIWKSYVHIMDGCTSSTCMMCYKRNRATRVECTTIVNGMKRSFYVYANGGRGFCK      2423
KY417143.1      FASFYYIWKSYVHIMDGCTSSTCMMCYKRNRATRVECTTIVNGMKRSFYVYANGGRGFCK      2423
KT444582.1      FASFYYIWKSYVHIMDGCTSSTCMMCYKRNRATRVECTTIVNGMKRSFYVYANGGRGFCK      2423
KC881005.1      FASFYYIWKSYVHIMDGCTSSTCMMCYKRNRATRVECTTIVNGMKRSFYVYANGGRGFCK      2423
KC881006.1      FASFYYIWKSYVHIMDGCTSSTCMMCYKRNRATRVECTTIVNGMKRSFYVYANGGRGFCK      2423
KF367457.1      FASFYYIWKSYVHIMDGCTSSTCMMCYKRNRATRVECTTIVNGMKRSFYVYANGGRGFCK      2423
KY417152.1      FASFYYIWKSYVHIMDGCTSSTCMMCYKRNRATRVECTTIVNGMKRSFYVYANGGRGFCK      2423
KY417146.1      FASFYYIWKSYVHIMDGCTSSTCMMCYKRNRATRVECTTIVNGMKRSFYVYANGGRGFCK      2423
KY417151.1      FASFYYIWKSYVHIMDGCTSSTCMMCYKRNRATRVECTTIVNGMKRSFYVYANGGRGFCK      2423
KY417142.1      FASFYYIWKSYVHIMDGCTSSTCMMCYKRNRATRVECTTIVNGMKRSFYVYANGGRGFCK      2423
                ******:**:*:*:::**. ***:*******************:::****:****:****
```

```
MW532698.1       LHNWNCVNCDTFCAGSTFISDEVARDLSLQFKRPINPTDQSSYVVDSVAVKNGSLHLYFD       2498
MT040336.1       LHNWNCVNCDTFCAGSTFISDEVARDLSLQFKRPINPTDQSSYVVDSVAVKNGSLHLYFD       2498
MT040335.1       LHNWNCVNCDTFCAGSTFISDEVARDLSLQFKRPINPTDQSSYVVDSVAVKNGSLHLYFD       2498
MT040334.1       LHNWNCVNCDTFCAGSTFISDEVARDLSLQFKRPINPTDQSSYVVDSVAVKNGSLHLYFD       2498
MT040333.1       LHNWNCVNCDTFCAGSTFISDEVARDLSLQFKRPINPTDQSSYVVDSVAVKNGSLHLYFD       2498
MN996532.2       LHNWNCVNCDTFCAGSTFISDEVARDLSLQFKRPINPTDQSSYIVDSVTVKNGSIHLYFD       2505
MN988713.1       LHNWNCVNCDTFCAGSTFISDEVARDLSLQFKRPINPTDQSSYIVDSVTVKNGSIHLYFD       2506
MT093571.1       LHNWNCVNCDTFCAGSTFISDEVARDLSLQFKRPINPTDQSSYIVDSVTVKNGSIHLYFD       2506
MN996529.1       LHNWNCVNCDTFCAGSTFISDEVARDLSLQFKRPINPTDQSSYIVDSVTVKNGSIHLYFD       2506
MT072688.1       LHNWNCVNCDTFCAGSTFISDEVARDLSLQFKRPINPTDQSSYIVDSVTVKNGSIHLYFD       2506
NC_045512.2      LHNWNCVNCDTFCAGSTFISDEVARDLSLQFKRPINPTDQSSYIVDSVTVKNGSIHLYFD       2506
MN994467.1       LHNWNCVNCDTFCAGSTFISDEVARDLSLQFKRPINPTDQSSYIVDSVTVKNGSIHLYFD       2506
MG772933.1       LHNWNCINCDTFCAGSTFISDEVARDLSLQFKRPINPTDQSSYIVDSVTVKNGSIHLYFD       2502
MG772934.1       LHNWNCINCDTFCAGSTFISDEVARDLSLQFKRPINPTDQSSYIVDSVTVKNGSIHLYFD       2480
NC_014470.1      LHNWNCLNCDTFCSGSTFISDEVARDLSLQFKRPINPTDQSSYNVDSVTVKDGTLYLYFQ       2473
GQ153542.1       AHNWNCLNCDTFCAGSTFISDEVARDLSLQFKRPINPTDQSAYVVDSVTVKNGALHLYFD       2481
DQ022305         AHNWNCLNCDTFCAGSTFISDEVARDLSLQFKRPINPTDQSAYVVDSVTVKNGALHLYFD       2477
KF569996.1       THNWNCLNCDTFCAGSTFISDEVARDLSLQFKRPINPTDQSSYVVDSVAVKNGALHLYFD       2483
KP886809.1       THNWNCLNCDTFCTGSTFISDEVARDLSLQFKRPINPTDQSSYIVDSVAVKNGALHLYFD       2483
AY278488.2       THNWNCLNCDTFCTGSTFISDEVARDLSLQFKRPINPTDQSSYIVDSVAVKNGALHLYFD       2483
AY485277.1       THNWNCLNCDTFCTGSTFISDEVARDLSLQFKRPINPTDQSSYIVDSVAVKNGALHLYFD       2483
AP006560.1       THNWNCLNCDTFCTGSTFISDEVARDLSLQFKRPINPTDQSSYIVDSVAVKNGALHLYFD       2483
AP006557.1       THNWNCLNCDTFCTGSTFISDEVARDLSLQFKRPINPTDQSSYIVDSVAVKNGALHLYFD       2483
AY274119         THNWNCLNCDTFCTGSTFISDEVARDLSLQFKRPINPTDQSSYIVDSVAVKNGALHLYFD       2483
AY572038.1       THNWNCLNCDTFCTGSTFISDEVARDLSLQFKRPINPTDQSSYIVDSVAVKNGALHLYFD       2483
AY572034.1       THNWNCLNCDTFCTGSTFISDEVARDLSLQFKRPINPTDQSSYIVDSVAVKNGALHLYFD       2483
FJ588686.1       THNWNCLNCDTFCTGSTFISDEVARDLSLQFKRSINPTDQSSYIVDSVAVKNGALHLYFD       2290
KY417145.1       THNWNCLNCDTFCTGSTFISDEVARDLSLQFKRPINPTDQSSYIVDSVAVKNGALHLYFD       2483
KY417144.1       THNWNCLNCDTFCAGSTFISDEVARDLSLQFKRPINPTDQSSYVVDSVAVKNGALHLYFD       2483
KY417147.1       THNWNCLNCDTFCTGSTFISDEVARDLSLQFKRPINPTDQSSYIVDSVAVKNGALHLYFD       2483
KY417148.1       THNWNCLNCDTFCTGSTFISDEVARDLSLQFKRPINPTDQSSYIVDSVAVKNGALHLYFD       2483
KY417143.1       THNWNCLNCDTFCTGSTFISDEVARDLSLQFKRPINPTDQSSYIVDSVAVKNGALHLYFD       2483
KT444582.1       THNWNCLNCDTFCTGSTFISDEVARDLSLQFKRPINPTDQSSYIVDSVAVKNGALHLYFD       2483
KC881005.1       THNWNCLNCDTFCTGSTFISDEVARDLSLQFKRPINPTDQSSYVVDSVAVKNGALHLYFD       2483
KC881006.1       THNWNCLNCDTFCTGSTFISDEVARDLSLQFKRPINPTDQSSYIVDSVAVKNGALHLYFD       2483
KF367457.1       THNWNCLNCDTFCTGSTFISDEVARDLSLQFKRPINPTDQSSYIVDSVAVKNGALHLYFD       2483
KY417152.1       THNWNCLNCDTFCTGSTFISDEVARDLSLQFKRPINPTDQSSYIVDSVAVKNGALHLYFD       2483
KY417146.1       THNWNCLNCDTFCTGSTFISDEVARDLSLQFKRPINPTDQSSYIVDSVAVKNGALHLYFD       2483
KY417151.1       THNWNCLNCDTFCTGSTFISDEVARDLSLQFKRPINPTDQSSYIVDSVAVKNGALHLYFD       2483
KY417142.1       THNWNCLNCDTFCTGSTFISDEVARDLSLQFKRPINPTDQSSYIVDSVAVKNGALHLYFD       2483
                  *****:******:******************* *******:* ****:**:*:::***:
```

```
MW532698.1      KAGQKTYERHSLSHFVNLDNLRANNTKGSIPINVIVFDGKSKCDESSARAASVYYSQLMC    2558
MT040336.1      KAGQKTYERHSLSHFVNLDNLRANNTKGSIPINVIVFDGKSKCDESSARAASVYYSQLMC    2558
MT040335.1      KAGQKTYERHSLSHFVNLDNLRANNTKGSIPINVIVFDGKSKCDESSARAASVYYSQLMC    2558
MT040334.1      KAGQKTYERHSLSHFVNLDNLRANNTKGSIPINVIVFDGKSKCDESSARAASVYYSQLMC    2558
MT040333.1      KAGQKTYERHSLSHFVNLDNLRANNTKGSIPINVIVFDGKSKCDESSARAASVYYSQLMC    2558
MN996532.2      KAGQKTYERHSLSHFVNLDNLRASNTKGSLPINVIVFDGKSKCEESSAKSASVYYSQLMC    2565
MN988713.1      KAGQKTYERHSLSHFVNLDNLRANNTKGSLPINVIVFDGKSKCEESSAKSASVYYSQLMC    2566
MT093571.1      KAGQKTYERHSLSHFVNLDNLRANNTKGSLPINVIVFDGKSKCEESSAKSASVYYSQLMC    2566
MN996529.1      KAGQKTYERHSLSHFVNLDNLRANNTKGSLPINVIVFDGKSKCEESSAKSASVYYSQLMC    2566
MT072688.1      KAGQKTYERHSLSHFVNLDNLRANNTKGSLPINVIVFDGKSKCEESSAKSASVYYSQLMC    2566
NC_045512.2     KAGQKTYERHSLSHFVNLDNLRANNTKGSLPINVIVFDGKSKCEESSAKSASVYYSQLMC    2566
MN994467.1      KAGQKTYERHSLSHFVNLDNLRANNTKGSLPINVIVFDGKSKCEESSAKSASVYYSQLMC    2566
MG772933.1      KAGQKTYERHSLSHFVNLDNLRANNTKGSLPINVIVFDGKSKCEESSAKSASVYYSQLMC    2562
MG772934.1      KAGQKTYERHSLSHFVNLDNLRANNTKGSLPINVIVFDGKSKCEESSAKSASVYYSQLMC    2540
NC_014470.1     KAGKLTYERHPLSYFVNLDNLRANNVKGTLPINVIVFDGKSKCEEAAAKSASVYYSQLMC    2533
GQ153542.1      KAGQKTYERHPLSHFVNLDNLRANNTKGSLPINVIVFDGKSKCEESAAKSASVYYSQLMC    2541
DQ022305        KAGQKTYERHPLSHFVNLDNLRANNTKGSLPINVIVFDGKSKCEESAAKSASVYYSQLMC    2537
KF569996.1      KAGQKTYERHPLSQFVNLDNLRANNTKGSLPINVIVFDGKSKCDESAAKSASVYYSQLMC    2543
KP886809.1      KAGQKTYERHPLSHFVNLDNLRGNNTKGSLPINVIVFDGKSKCDESAAKSASVYYSQLMC    2543
AY278488.2      KAGQKTYERHPLSHFVNLDNLRANNTKGSLPINVIVFDGKSKCDESASKSASVYYSQLMC    2543
AY485277.1      KAGQKTYERHPLSHFVNLDNLRANNTKGSLPINVIVFDGKSKCDESASKSASVYYSQLMC    2543
AP006560.1      KAGQKTYERHPLSHFVNLDNLRANNTKGSLPINVIVFDGKSKCDESASKSASVYYSQLMC    2543
AP006557.1      KAGQKTYERHPLSHFVNLDNLRANNTKGSLPINVIVFDGKSKCDESASKSASVYYSQLMC    2543
AY274119        KAGQKTYERHPLSHFVNLDNLRANNTKGSLPINVIVFDGKSKCDESASKSASVYYSQLMC    2543
AY572038.1      KAGQKTYERHPLSHFVNLDNLRANNTKGSLPINVIVFDGKSKCDESASKSASVYYSQLMC    2543
AY572034.1      KAGQKTYERHPLSHFVNLDNLRANNTKGSLPINVIVFDGKSKCDESASKSASVYYSQLMC    2543
FJ588686.1      KAGQKTYERHPLSHFVNLDNLRANNTKGSLPINVIVFDGKSKCDESAAKSASVYYSQLMC    2350
KY417145.1      KAGQKTYERHPLSHFVNLDNLRANNTKGSLPINVIVFDGKSKCDESAARSASVYYSQLMC    2543
KY417144.1      KAGQKTYERHPLSHFVNLDNLRTNNTKGSLPINVIVFDGKSKCDESAAKSASVYYSQLMC    2543
KY417147.1      KAGQKTYERHPLSHFVNLDNLRANNTKGSLPINVIVFDGKSKCDESAARSASVYYSQLMC    2543
KY417148.1      KAGQKTYERHPLSHFVNLDNLRANNTKGSLPINVIVFDGKSKCDESAARSASVYYSQLMC    2543
KY417143.1      KAGQKTYERHPLSHFVNLDNLRANNTKGSLPINVIVFDGKSKCDESAARSASVYYSQLMC    2543
KT444582.1      KAGQKTYERHPLSHFVNLDNLRANNTKGSLPINVIVFDGKSKCDESAARSASVYYSQLMC    2543
KC881005.1      KAGQKTYERHPLSHFVNLDNLRANNTKGSLPINVIVFDGKSKCDESAARSASVYYSQLMC    2543
KC881006.1      KAGQKTYERHPLSHFINLDNLRANNTKGSLPINVIVFDGKSKCDESAARSASVYYSQLMC    2543
KF367457.1      KAGQKTYERHPLSHFINLDNLRANNTKGSLPINVIVFDGKSKCDESAARSASVYYSQLMC    2543
KY417152.1      KAGQKTYERHPLSHFVNLDNLRANNTKGSLPINVIVFDGKSKCDESAARSASVYYSQLMC    2543
KY417146.1      KAGQKTYERHPLSHFVNLDNLRANNTKGSLPINVIVFDGKSKCDESAARSASVYYSQLMC    2543
KY417151.1      KAGQKTYERHPLSHFVNLDYLRANNTKGSLPINVIVFDGKSKWDESAAKSASVYYSQLMC    2543
KY417142.1      KAGQKTYERHPLSHFVNLDNLRANNTKGSLPINVIVFDGKSKCDESAARSASVYYSQLMC    2543
                ***: ***** ** *:*** ** .*.**::************ :*:::::**********
```

```
MW532698.1          QPILLLDQALVSDVGDSAEVAVKMFDAYVNTFSSTFNVPMDKLKTLIATAETELAKNVSL          2618
MT040336.1          QPILLLDQALVSDVGDSAEVAVKMFDAYVNTFSSTFNVPMDKLKTLIATAETELAKNVSL          2618
MT040335.1          QPILLLDQALVSDVGDSAEVAVKMFDAYVNTFSSTFNVPMDKLKTLIATAETELAKNVSL          2618
MT040334.1          QPILLLDQALVSDVGDSAEVAVKMFDAYVNTFSSTFNVPMDKLKTLIATAETELAKNVSL          2618
MT040333.1          QPILLLDQALVSDVGDSAEVAVKMFDAYVNTFSSTFNVPMDKLKTLIATAETELAKNVSL          2618
MN996532.2          QPILLLDQALVSDVGDSTEVAVKMFDAYVNTFSSTFNVPMEKLKTLVATAEAELAKNVSL          2625
MN988713.1          QPILLLDQALVSDVGDSAEVAVKMFDAYVNTFSSTFNVPMEKLKTLVATAEAELAKNVSL          2626
MT093571.1          QPILLLDQALVSDVGDSAEVAVKMFDAYVNTFSSTFNVPMEKLKTLVATAEAELAKNVSL          2626
MN996529.1          QPILLLDQALVSDVGDSAEVAVKMFDAYVNTFSSTFNVPMEKLKTLVATAEAELAKNVSL          2626
MT072688.1          QPILLLDQALVSDVGDSAEVAVKMFDAYVNTFSSTFNVPMEKLKTLVATAEAELAKNVSL          2626
NC_045512.2         QPILLLDQALVSDVGDSAEVAVKMFDAYVNTFSSTFNVPMEKLKTLVATAEAELAKNVSL          2626
MN994467.1          QPILLLDQALVSDVGDSAEVAVKMFDAYVNTFSSTFNVPMEKLKTLVATAEAELAKNVSL          2626
MG772933.1          QPILLLDQALVSDVGDSAEVAVKMFDAYVNIFSSTFNVPMEKLKALVATAEAELAKNVSL          2622
MG772934.1          QPILLLDQALVSDVGDSAEVAVKMFDAYVNIFSSTFNVPMEKLKALVATAEAELAKNVSL          2600
NC_014470.1         QPILLLDQALISDVGDSTEVAVKMFDAYVNAFSSTFNAPMEKLKTFIATAHAEIAKGVSL          2593
GQ153542.1          QPILLLDQALVSDVGDSTEVSVKMFDAYVDTFSATFSVPMEKLKALVATAHSELAKGVAL          2601
DQ022305            QPILLLDQALVSDVGDSTEVSVKMFDAYVDTFSATFSVPMEKLKALVATAHSELAKGVAL          2597
KF569996.1          QPILLLDQALVSDVGDSTEVSIKMFDAYVDTFSATFSVPMEKLKALVATAHSELAKGVAL          2603
KP886809.1          QPILLLDQALVSDVGDSTEVSVKMFDAYVDIFSSTFSVPMEKLKALVVTAHSELAKGVAL          2603
AY278488.2          QPILLLDQALVSDVGDSTEVSVKMFDAYVDTFSATFSVPMEKLKALVATAHSELAKGVAL          2603
AY485277.1          QPILLLDQALVSDVGDSTEVSVKMFDAYVDTFSATFSVPMEKLKALVATAHSELAKGVAL          2603
AP006560.1          QPILLLDQALVSDVGDSTEVSVKMFDAYVDTFSATFSVPMEKLKALVATAHSELAKGVAL          2603
AP006557.1          QPILLLDQALVSDVGDSTEVSVKMFDAYVDTFSATFSVPMEKLKALVATAHSELAKGVAL          2603
AY274119            QPILLLDQALVSDVGDSTEVSVKMFDAYVDTFSATFSVPMEKLKALVATAHSELAKGVAL          2603
AY572038.1          QPILLLDQALVSDVGDSTEVSVKMFDAYVDTFSATFSVPMEKLKALVATAHSELAKGVAL          2603
AY572034.1          QPILLLDQALVSDVGDSTEVSVKMFDAYVDTFSATFSVPMEKLKALVATAHSELAKGVAL          2603
FJ588686.1          QPILLLDQSLVSDVGDSTEVSVKMFDAYVDTFSATFSVPMEKLKALVATAHSELAKGVAL          2410
KY417145.1          QPILLLDQALVSDVGDSTEVSVKMFDAYVDTFSATFSVPMEKLKALVATAHSELAKGVAL          2603
KY417144.1          QPILLLDQALVSDVGDSTEVSVKMFDAYVDTFSATFSVPMEKLKALVATAHSELAKGVAL          2603
KY417147.1          QPILLLDQALVSDVGDSTEVSVKMFDAYVDTFSATFSVPMEKLKVLVATAHSELAKGVAL          2603
KY417148.1          QPILLLDQTLVSDVGDSTEVSVKMFDAYVDTFSATFSVPMEKLKALVATAHSELAKGVAL          2603
KY417143.1          QPILLLDQTLVSDVGDSTEVSVKMFDAYVDTFSATFSVPMEKLKALVATAHSELAKGVAL          2603
KT444582.1          QPILLLDQTLVSDVGDSTEVSVKMFDAYVDTFSATFSVPMEKLKALVATAHSELAKGVAL          2603
KC881005.1          QPILLLDQVLVSDVGGSTEVSVKMFDAYVDTFSATFSVPMEKLKALVATAHSELAKGVAL          2603
KC881006.1          QPILLLDQALISDVGDSTEVSVKMFDAYVDTFSATFSVPMEKLRALVATAHSELAKGVAL          2603
KF367457.1          QPILLLDQALISDVGDSTEVSVKMFDAYVDTFSATFSVPMEKLRALVATAHSELAKGVAL          2603
KY417152.1          QPILLLDQALVSDVGDSTEVSVKMFDAYVDTFSATFSVPMEKLKALVATAHSELAKGVAL          2603
KY417146.1          QPILLLDQALVSDVGDSTEVSVKMFDAYVDTFSATFSVPMEKLKVLVATAHSELAKGVAL          2603
KY417151.1          QPILLLDQALVSDVGDSTEVSVKMFDAYVDTFSATFSVPMEKLKALVATAHSELAKGVAL          2603
KY417142.1          QPILLLDQALVSDVGDSTEVSVKMFDAYVDTFSATFSVPMEKLKVLVATAHSELAKGVAL          2603
                    ******** *:****.*:**::*******: **:**..**:**:.::.**.:*:**.*:*
```

MW532698.1	DNVLSTFISAARQGFVDSDVDTKDVVECLKISHQSDIEVTGDSCNNYMLTYNKVENMTPR	2678
MT040336.1	DNVLSTFISAARQGFVDSDVDTKDVVECLKISHQSDIEVTGDSCNNYMLTYNKVENMTPR	2678
MT040335.1	DNVLSTFISAARQGFVDSDVDTKDVVECLKISHQSDIEVTGDSCNNYMLTYNKVENMTPR	2678
MT040334.1	DNVLSTFISAARQGFVDSDVDTKDVVECLKISHQSDIEVTGDSCNNYMLTYNKVENMTPR	2678
MT040333.1	DNVLSTFISAARQGFVDSDVDTKDVVECLKISHQSDIEVTGDSCNNYMLTYNKVENMTPR	2678
MN996532.2	DNVLSTFISAARQGFVDSDVETKDVVECLKLSHQSDIEVTGDSCNNYMLTYNKVENMTPR	2685
MN988713.1	DNVLSTFISAARQGFVDSDVETKDVVECLKLSHQSDIEVTGDSCNNYMLTYNKVENMTPR	2686
MT093571.1	DNVLSTFISAARQGFVDSDVETKDVVECLKLSHQSDIEVTGDSCNNYMLTYNKVENMTPR	2686
MN996529.1	DNVLSTFISAARQGFVDSDVETKDVVECLKLSHQSDIEVTGDSCNNYMLTYNKVENMTPR	2686
MT072688.1	DNVLSTFISAARQGFVDSDVETKDVVECLKLSHQSDIEVTGDSCNNYMLTYNKVENMTPR	2686
NC_045512.2	DNVLSTFISAARQGFVDSDVETKDVVECLKLSHQSDIEVTGDSCNNYMLTYNKVENMTPR	2686
MN994467.1	DNVLSTFISAARQGFVDSDVETKDVVECLKLSHQSDIEVTGDSCNNYMLTYNKVENMTPR	2686
MG772933.1	DNVLSTFISAARQGFVDSDVETKDVVECLKLSHQSDIEVTGDSCNNYMLTYNKVENMTPR	2682
MG772934.1	DNVLSTFISAARQGFVDSDVETKDVVECLKLSHQSDIEVTGDSCNNYMLTYNKVENMTPR	2660
NC_014470.1	DSVLSTFLSAARQGFVDSDVDTKDVMECLKLSHHSDLEITSDSCNNFMLTYNKVENMTPR	2653
GQ153542.1	DGVLSTFMSTARQGVVDTDVDTKDVIECLKLSHHSDIEVTGDSCNNFMLTYNKVENMTPR	2661
DQ022305	DGVLSTFVSAARQGVVDTDVDTKDVIECLKLSHHSDIEVTGDSCNNFMLTYNKVENMTPR	2657
KF569996.1	DGVLSTFVSAARQGVVDTDVDTKDVIECLKLSHHSDLEVTGDSCNNFMLTYNKVENMTPR	2663
KP886809.1	DGVLSTFVSAARQGVVDTDVDTKDVIECLKLSHHSDLEVTGDSCNNFMLTYNKVENMTPR	2663
AY278488.2	DGVLSTFVSAARQGVVDTDVDTKDVIECLKLSHHSDLEVTGDSCNNFMLTYNKVENMTPR	2663
AY485277.1	DGVLSTFVSAARQGVVDTDVDTKDVIECLKLSHHSDLEVTGDSCNNFMLTYNKVENMTPR	2663
AP006560.1	DGVLSTFVSAARQGVVDTDVDTKDVIECLKLSHHSDLEVTGDSCNNFMLTYNKVENMTPR	2663
AP006557.1	DGVLSTFVSAARQGVVDTDVDTKDVIECLKLSHHSDLEVTGDSCNNFMLTYNKVENMTPR	2663
AY274119	DGVLSTFVSAARQGVVDTDVDTKDVIECLKLSHHSDLEVTGDSCNNFMLTYNKVENMTPR	2663
AY572038.1	DGVLSTFVSAARQGVVDTDVDTKDVIECLKLSHHSDLEVTGDSCNNFMLTYNKVENMTPR	2663
AY572034.1	DGVLSTFVSAARQGVVDTDVDTKDVIECLKLSHHSDLEVTGDSCNNFMLTYNKVENMTPR	2663
FJ588686.1	DGVLSTFVSAARQGVVDTDVDTKDVIECLKLSYHSDLEVTGDSCNNFMLTYNKVENMTPR	2470
KY417145.1	DGVLSTFVSAARQGVVDTDVDTKDVIECLKLSHHSDLEVTGDSCNNFMLTYNKVENMTPR	2663
KY417144.1	DGVLSTFVSAARQGVVDTDVDTKDVIECLKLSHHSDLEVTGDSCNNFMLTYNKVENMTPR	2663
KY417147.1	DGVLSTFVSAARQGVVDTDVDTKDVIECLKLSHHSDLEVTGDSCNNFMLTYNKVENMTPR	2663
KY417148.1	DGVLSTFVSAARQGVVDTDVDTKDVIECLKLSHHSDLEVTGDSCNNFMLTYNKVENMTPR	2663
KY417143.1	DGVLSTFVSAARQGVVDTDVDTKDVIECLKLSHHSDLEVTGDSCNNFMLTYNKVENMTPR	2663
KT444582.1	DGVLSTFVSAARQGVVDTDVDTKDVIECLKLSHHSDLEVTGDSCNNFMLTYNKVENMTPR	2663
KC881005.1	DGVLSTFVSAARQGVVDTDVDTKDVIECLKLSHHSDLEVTGDSCNNFMLTYNKVENMTPR	2663
KC881006.1	DGVLSTFVSAARQGVVDTDVDTKDVIECLKLSHHSDLEVTGDSCNNFMLTYNKVENMTPR	2663
KF367457.1	DGVLSTFVSAARQGVVDTDVDTKDVIECLKLSHHSDLEVTGDSCNNFMLTYNKVENMTPR	2663
KY417152.1	DGVLSTFVSAARQGVVDTDVDTKDVIECLKLSHHSDLEVTGDSCNNFMLTYNKVENMTPR	2663
KY417146.1	DGVLSTFVSAARQGVVDTDVDTKDVIECLKLSHHSDLEVTGDSCNNFMLTYNKVENMTPR	2663
KY417151.1	DGVLSTFVSAARQGVVDTDVDTKDVIECLKLSHHSDLEVTGDSCNNFMLTYNKVENMTPR	2663
KY417142.1	DGVLSTFVSAARQGVVDTDVDTKDVIECLKLSHHSDLEVTGDSCNHFMLTYNKVENMTPR	2663
	*.*****:*:****.**:**:****:****:*::**:*:*.****::*************	

```
MW532698.1      DLGACIDCSARHINAQVAKSHNISLIWNIKDFMSLSEQLRKQIRSAAKKNNLPFKLTCAT      2738
MT040336.1      DLGACIDCSARHINAQVAKSHNISLIWNIKDFMSLSEQLRKQIRSAAKKNNLPFKLTCAT      2738
MT040335.1      DLGACIDCSARHINAQVAKSHNISLIWNIKDFMSLSEQLRKQIRSAAKKNNLPFKLTCAT      2738
MT040334.1      DLGACIDCSARHINAQVAKSHNISLIWNIKDFMSLSEQLRKQIRSAAKKNNLPFKLTCAT      2738
MT040333.1      DLGACIDCSARHINAQVAKSHNISLIWNIKDFMSLSEQLRKQIRSAAKKNNLPFKLTCAT      2738
MN996532.2      DLGACIDCSARHINAQVAKSHNIALIWNVKDFMSLSEQLRKQIRSAAKKNNLPFKLTCAT      2745
MN988713.1      DLGACIDCSARHINAQVAKSHNIALIWNVKDFMSLSEQLRKQIRSAAKKNNLPFKLTCAT      2746
MT093571.1      DLGACIDCSARHINAQVAKSHNIALIWNVKDFMSLSEQLRKQIRSAAKKNNLPFKLTCAT      2746
MN996529.1      DLGACIDCSARHINAQVAKSHNIALIWNVKDFMSLSEQLRKQIRSAAKKNNLPFKLTCAT      2746
MT072688.1      DLGACIDCSARHINAQVAKSHNIALIWNVKDFMSLSEQLRKQIRSAAKKNNLPFKLTCAT      2746
NC_045512.2     DLGACIDCSARHINAQVAKSHNIALIWNVKDFMSLSEQLRKQIRSAAKKNNLPFKLTCAT      2746
MN994467.1      DLGACIDCSARHINAQVAKSHNIALIWNVKDFMSLSEQLRKQIRSAAKKNNLPFKLTCAT      2746
MG772933.1      DLGACIDCSARHINAQVAKSHNIALIWNVKDFMSLSEQLRKQIRSAAKKNNLPFRLTCAT      2742
MG772934.1      DLGACIDCSARHINAHVAKSHNIALIWNVKDFMSLSEQLRKQIRSAAKKNNLPFRLTCAT      2720
NC_014470.1     DLGACIDCSARHINAQVAKSHNVSLVWNVKDYMSLSEQLRKQIRSAAKKNNIPFKLTCAT      2713
GQ153542.1      DLGACIDCNARHINAQVAKSHNVSLVWNVKDYMSLSEQLRKQIRSAAKKNNIPFRLTCAT      2721
DQ022305        DLGACIDCNARHINAQVAKSHNVSLVWNVKDYMSLSEQLRKQIRSAAKKNNIPFRLTCAT      2717
KF569996.1      DLGACIDCNARHINAQVAKSHNVSLIWNVKDYMSLSEQLRKQIRSAAKKNNIPFRLTCAT      2723
KP886809.1      DLGACIDCNARHINAPVAKSHNVSLIWNVKDYMSLSEQLRKQIRSAAKKNNIPFRLTCAT      2723
AY278488.2      DLGACIDCNARHINAQVAKSHNVSLIWNVKDYMSLSEQLRKQIRSAAKKNNIPFRLTCAT      2723
AY485277.1      DLGACIDCNARHINAQVAKSHNVSLIWNVKDYMSLSEQLRKQIRSAAKKNNIPFRLTCAT      2723
AP006560.1      DLGACIDCNARHINAQVAKSHNVSLIWNVKDYMSLSEQLRKQIRSAAKKNNIPFRLTCAT      2723
AP006557.1      DLGACIDCNARHINAQVAKSHNVSLIWNVKDYMSLSEQLRKQIRSAAKKNNIPFRLTCAT      2723
AY274119        DLGACIDCNARHINAQVAKSHNVSLIWNVKDYMSLSEQLRKQIRSAAKKNNIPFRLTCAT      2723
AY572038.1      DLGACIDCNARHINAQVAKSHNVSLIWNVKDYMSLSEQLRKQIRSAAKKNNIPFRLTCAT      2723
AY572034.1      DLGACIDCNARHINAQVAKSHNVSLIWNVKDYMSLSEQLRKQIRSAAKKNNIPFRLTCAT      2723
FJ588686.1      DLGACIDCNARHINAQVAKSHNVSLIWNVKDYMSLSEQLRKQIRSAAKKNNIPFRLTCAT      2530
KY417145.1      DLGACIDCNARHINAQVAKSHNVSLIWNVKDYMSLSEQLRKQIRSAAKKNNIPFRLTCAT      2723
KY417144.1      DLGACIDCNARHINAQVAKSHNVSLIWNVKDYMSLSEQLRKQIRSAAKKNNIPFRLTCAT      2723
KY417147.1      DLGACIDCNARHINAQVAKSHNVSLIWNVKDYMSLSEQLRKQIRSAAKKNNIPFRLTCAT      2723
KY417148.1      DLGACIDCNARHINAQVAKSHNVSLIWNVKDYMSLSEQLRKQIRSAAKKNNIPFRLTCAT      2723
KY417143.1      DLGACIDCNARHINAQVAKSHNVSLIWNVKDYMSLSEQLRKQIRSAAKKNNIPFRLTCAT      2723
KT444582.1      DLGACIDCNARHINAQVAKSHNVSLIWNVKDYMSLSEQLRKQIRSAAKKNNIPFRLTCAT      2723
KC881005.1      DLGACIDCNARHINAQVAKSHNVSLIWNVKDYMSLSEQLRKQIRSAAKKNNIPFRLTCAT      2723
KC881006.1      DLGACIDCNARHINAQVAKSHNVSLIWNVKDYMSLSEQLRKQIRSAAKKNNIPFRLTCAT      2723
KF367457.1      DLGACIDCNARHINAQVAKSHNVSLIWNVKDYMSLSEQLRKQIRSAAKKNNIPFRLTCAT      2723
KY417152.1      DLGACIDCNARHINAQVAKSHNVSLIWNVKDYMSLSEQLRKQIRSAAKKNNIPFRLTCAT      2723
KY417146.1      DLGACIDCNARHINAQVAKSHNVSLIWNVKDYMSLSEQLRKQIRSAAKKNNIPFRLTCAT      2723
KY417151.1      DLGACIDCNARHINAQVAKSHNVSLIWNVKDYMSLSEQLRKQIRSAAKKNNIPFRLTCAT      2723
KY417142.1      DLGACIDCNARHINAQVAKSHNVSLIWNVKDYMSLSEQLRKQIRSAAKKNNIPFRLTCAT      2723
                ********.****** ******::*:**:**:*******************:**:*****
```

```
MW532698.1        TRQVVNVVTTKIALKGGKFVT-NWFKYLLKATLVCVVIACVFYFITPVHVLTKHGDFADE        2797
MT040336.1        TRQVVNVVTTKIALKGGKFVT-NWFKYLLKATLVCVVIACVFYFITPVHVLTKHGDFADE        2797
MT040335.1        TRQVVNVVTTKIALKGGKFVT-NWFKYLLKATLVCVVIACVFYFITPVHVLTKHGDFADE        2797
MT040334.1        TRQVVNVVTTKIALKGGKFVT-NWFKYLLKATLVCVVIACVFYFITPVHVLTKHGDFADE        2797
MT040333.1        TRQVVNVVTTKIALKGGKFVT-NWFKYLLKATLVCVVIACVFYFITPVHVLTKHGDFADE        2797
MN996532.2        TRQVVNVVTTKIALKGGKIVN-NWLKQLIKVTLVFLFVAAIFYLITPVHVMSKHTDFSSE        2804
MN988713.1        TRQVVNVVTTKIALKGGKIVN-NWLKQLIKVTLVFLFVAAIFYLITPVHVMSKHTDFSSE        2805
MT093571.1        TRQVVNVVTTKIALKGGKIVN-NWLKQLIKVTLVFLFVAAIFYLITPVHVMSKHTDFSSE        2805
MN996529.1        TRQVVNVVTTKIALKGGKIVN-NWLKQLIKVTLVFLFVAAIFYLITPVHVMSKHTDFSSE        2805
MT072688.1        TRQVVNVVTTKIALKGGKIVN-NWLKQLIKVTLVFLFVAAIFYLITPVHVMSKHTDFSSE        2805
NC_045512.2       TRQVVNVVTTKIALKGGKIVN-NWLKQLIKVTLVFLFVAAIFYLITPVHVMSKHTDFSSE        2805
MN994467.1        TRQVVNVVTTKIALKGGKIVN-NWLKQLIKVTLVFLFVAAIFYLITPVHVMSKHTDFSSE        2805
MG772933.1        TRQVVNVVTTKIALKGGKIVN-NWLKQLIKVTLVFLFITVIFYLITPVHVMFKHNDFSSE        2801
MG772934.1        TRQVVNVVTTKIALRGGKIVN-NWLKQLIKVTLVFLFITVIFYLITPAHVMFKHNDFSSE        2779
NC_014470.1       TRQVVNVITTKISLKGGKFVSNNWFRFLLKMTVLMVLVAFIFYFITPTHTLMGHDVFSSE        2773
GQ153542.1        TRQVVNVITTKISLKGGKVVS-TWFKLLLKVTLVCVLAALFCYVIMPVHSLSVHDGYTNE        2780
DQ022305          TRQVVNVITTKISLKGGKVVS-TWFKLLLKVTLLCVLAALFCYVIMPVHSLSVHDGYTNE        2776
KF569996.1        TRQVVNVITTKISLKGGKVIS-TWFKFMLKATLVCVLAALVCYIVMPVHTLSVYDGYTNE        2782
KP886809.1        TRQVVNVITTKISLKGGKIVS-TWFKLMLKATLLCVLAALVCYIVMPVHTLSIHDGYTNE        2782
AY278488.2        TRQVVNVITTKISLKGGKIVS-TCFKLMLKATLLCVLAALVCYIVMPLHTLSIHDGYTNE        2782
AY485277.1        TRQVVNVITTKISLKGGKIVS-TCFKLMLKATLLCVLAALVCYIVMPVHTLSIHDGYTNE        2782
AP006560.1        TRQVVNVITTKISLKGGKIVS-TCFKLMLKATLLCVLAALVCYIVMPVHTLSIHDGYTNE        2782
AP006557.1        TRQVVNVITTKISLKGGKIVS-TCFKLMLKATLLCVLAALVCYIVMPVHTLSIHDGYTNE        2782
AY274119          TRQVVNVITTKISLKGGKIVS-TCFKLMLKATLLCVLAALVCYIVMPVHTLSIHDGYTNE        2782
AY572038.1        TRQVVNVITTKISLKGGKIVS-TWFKLMLKATLLCVLAALVCYIVMPVHTLSIHDGYTNE        2782
AY572034.1        TRQVVNVITTKISLKGGKIVS-TWFKLMLKATLLCVLAALVCYIVMPVHTLSIHDGYTNE        2782
FJ588686.1        TRQVVNVITTKISLKGGKIVS-TWFKLMLKATLLCVLAAFVCYIVMPVHTLSAHDGYTNE        2589
KY417145.1        TRQVVNVITTKISLKGGKIVS-TWFKLMLKATLLCVFAALVCYIVMPVHTLSAHDGYTNE        2782
KY417144.1        TRQVVNVITTKISLKGGKIVS-TWFKLMLKATLLCVIAALVCYIVMPVHTLSAHDGYTNE        2782
KY417147.1        TRQVVNVITTKISLKGGKIVS-TWFKLMLKATLLCVFAALVCYIVMPVHTLSAHDGYTNE        2782
KY417148.1        TRQVVNVITTKISLKGGKIVS-TWFKLMLKATLLCVFAALVCYIVMPVHTLSAHDGYTNE        2782
KY417143.1        TRQVVNVITTKISLKGGKIVS-TWFKLMLKATLLCVFAALVCYIVMPVHTLSAHDGYTNE        2782
KT444582.1        TRQVVNVITTKISLKGGKIVS-TWFKLMFKATLLCVFAALVCYIVMPVHTLSAHDGYTNE        2782
KC881005.1        TRQVVNVITTKISLKGGKIVS-TWFKLMLKATLLCVFAALVCYIVMPVHTLSAHDGYTNE        2782
KC881006.1        TRQVVNVITTKISLKGGKIVS-TWFKLMLKATLLCVIATLVCYIVMPVHTLSAHDGYTNE        2782
KF367457.1        TRQVVNVITTKISLKGGKIVS-TWFKLMLKATLLCVIATLVCYIVMPVHTLSVHDGYTNE        2782
KY417152.1        TRQVVNVITTKISLKGGKIVS-TWFKLMLKATLLCVFAALVCYIVMPVHTLSAHDGYTNE        2782
KY417146.1        TRQVVNVITTKISLKGGKIVS-TWFKLMLKATLLCVFAALVCYIVMPVHTLSAHDGYTNE        2782
KY417151.1        TRQVVNVITTKISLKGGKIVS-TWFKLMLKATLLCVIAALVCYIVMPVHTLSAHDGYTNE        2782
KY417142.1        TRQVVNVITTKISLKGGKIVS-TWFKLMLKATLLCVFAALVCYIVMPVHTLSAHDGYTNE        2782
                  *******:****:*:***.:. . :: ::* *:: :. : . *.: * * :  :  ::.*
```

```
MW532698.1      IIGYKAIEDGVTRDISSNDNCFANKHVGFDSWFSQRGGSYTNDKTCPIVAAVITRDVGFV    2857
MT040336.1      IIGYKAIEDGVTRDISSNDNCFANKHVGFDSWFSQRGGSYTNDKTCPIVAAVITRDVGFV    2857
MT040335.1      IIGYKAIEDGVTRDISSNDNCFANKHVGFDSWFSQRGGSYTNDKTCPIVAAVITRDVGFV    2857
MT040334.1      IIGYKAIEDGVTRDISSNDNCFANKHVGFDSWFSQRGGSYTNDKTCPIVAAVITRDVGFV    2857
MT040333.1      IIGYKAIEDGVTRDISSNDNCFANKHVGFDSWFSQRGGSYTNDKTCPIVAAVITRDVGFV    2857
MN996532.2      IIGYKAIDGGVTRDIASTDTCFANKHADFDTWFSQRGGSYTNDKACPLIAAVITREVGFV    2864
MN988713.1      IIGYKAIDGGVTRDIASTDTCFANKHADFDTWFSQRGGSYTNDKACPLIAAVITREVGFV    2865
MT093571.1      IIGYKAIDGGVTRDIASTDTCFANKHADFDTWFSQRGGSYTNDKACPLIAAVITREVGFV    2865
MN996529.1      IIGYKAIDGGVTRDIASTDTCFANKHADFDTWFSQRGGSYTNDKACPLIAAVITREVGFV    2865
MT072688.1      IIGYKAIDGGVTRDIASTDTCFANKHADFDTWFSQRGGSYTNDKACPLIAAVITREVGFV    2865
NC_045512.2     IIGYKAIDGGVTRDIASTDTCFANKHADFDTWFSQRGGSYTNDKACPLIAAVITREVGFV    2865
MN994467.1      IIGYKAIDGGVTRDIASTDTCFANKHADFDTWFSQRGGSYTNDKACPLIAAVITREVGFV    2865
MG772933.1      IIGYKAIDGGVTRDIASTDTCFANKHADFDSWFSQRGGSYTNDKACPLVAAVITREVGFV    2861
MG772934.1      IIGYKAIDGGVTRDIAPTDTCFANKHADFDSWFSQRGGSYTNDKACPLIAAVITREVGFV    2839
NC_014470.1     IIGYKAIHNGVTRDVLTTDDCFANKHTGFDHWFSQRGGSYRNDKTCPVIAAVITREVGFI    2833
GQ153542.1      IIGYKAIQDGVTRDIVSTDDCFANKHAGFDSWFSQRGGSYRNNKNCPVVAAIITREIGFI    2840
DQ022305        IIGYKAIQDGVTRDIVSTDDCFANKHAGFDSWFSQRGGSYRNDKNCPVVAAIITREIGFI    2836
KF569996.1      IIGYKAIQDGVTRDILSTDDCFANKHAGFDTWFSQRGGSYRNDKSCPVVAAIITREIGFI    2842
KP886809.1      IIGYKAIQDGVTRDIVSTDDCFANKHAGFDAWFSQRGGSYKNDKSCPVVAAIITREIGFI    2842
AY278488.2      IIGYKAIQDGVTRDIISTDDCFANKHAGFDAWFSQRGGSYKNDKSCPVVAAIITREIGFI    2842
AY485277.1      IIGYKAIQDGVTRDIISTDDCFANKHAGFDAWFSQRGGSYKNDKSCPVVAAIITREIGFI    2842
AP006560.1      IIGYKAIQDGVTRDIISTDDCFANKHAGFDAWFSQRGGSYKNDKSCPVVAAIITREIGFI    2842
AP006557.1      IIGYKAIQDGVTRDIISTDDCFANKHAGFDAWFSQRGGSYKNDKSCPVVAAIITREIGFI    2842
AY274119        IIGYKAIQDGVTRDIISTDDCFANKHAGFDAWFSQRGGSYKNDKSCPVVAAIITREIGFI    2842
AY572038.1      IIGYKAIQDGVTRDIISTDDCFANKHAGFDAWFSQRGGSYKNDKSCPVVAAIITREIGFI    2842
AY572034.1      IIGYKAIQDGVTRDIISTDDCFANKHAGFDAWFSQRGGSYKNDKSCPVVAAIITREIGFI    2842
FJ588686.1      VIGYKAIQDGVTRDIVSTDDCFANKHAGFDSWFSQRGGSYKNDKSCPVVAAIITREIGFI    2649
KY417145.1      IIGYKAIQDGVTRDIVSTDDCFANKHAGFDSWFSQRGGSYKNDKSCPVVAAIITREIGFI    2842
KY417144.1      IIGYKAIQDGVTRDIVSTDDCFANKHAGFDSWFSQRGGSYKNDKSCPVVAAIITREIGFI    2842
KY417147.1      IIGYKAIQDGVTRDIVSTDDCFANKHAGFDSWFSQRGGSYKNDKSCPVVAAIITREIGFI    2842
KY417148.1      IIGYKAIQDGVTRDIVSTDDCFANKHAGFDSWFSQRGGSYKNDKSCPVVAAIITREIGFI    2842
KY417143.1      IIGYKAIQDGVTRDIVSTDDCFANKHAGFDSWFSQRGGSYKNDKSCPVVAAIITREIGFI    2842
KT444582.1      IIGYKAIQDGVTRDIVSTDDCFANKHAGFDSWFSQRGGSYKNDKSCPVVAAIITREIGFI    2842
KC881005.1      IIGYKAIQDGVTRDIISTDDCFANKHAGFDSWFSQRGGSYKNDKSCPVVAAIITREIGFI    2842
KC881006.1      IIGYKAIQDGVTRDIISTDDCFANKHAGFDSWLSQRGGSYKNDKSCPVVAAIITREIGFI    2842
KF367457.1      IIGYKAIQDGVTRDIVSTDDCFANKHAGFDSWLSQRGGSYKNDKSCPVVAAIITREIGFI    2842
KY417152.1      IIGYKAIQDGVTRDIVSTDDCFANKHAGFDSWFSQRGGSYKNDKSCPVVAAIITREIGFI    2842
KY417146.1      IIGYKAIQDGVTRDIVSTDDCFANKHAGFDSWFSQRGGSYKNDKSCPVVAAIITREIGFI    2842
KY417151.1      IIGYKAIQDGVTRDIVSTDDCFANKHAGFDSWFSQRGGSYKNDKSCPVVAAIITREIGFI    2842
KY417142.1      IIGYKAIQDGVTRDIVSTDDCFANKHAGFDSWFSQRGGSYKNDKSCPVVAAIITREIGFI    2842
                :******..*****:  .* ******..** *:******* *:* **::**:***::**:
```

```
MW532698.1      VPGLPGTIFRTLSGDFLHFLPRVFSAVGNICYTPSKLIEYTDFATSACVLAAECTIFKDA      2917
MT040336.1      VPGLPGTIFRTLSGDFLHFLPTVISAVGNICYTPSKLIEYTDFATSACVLAAECTIFKDA      2917
MT040335.1      VPGLPGTIFRTLSGDFLHFLPRVFSAVGNICYTPSKLIEYTDFATSACVLAAECTIFKDA      2917
MT040334.1      VPGLPGTIFRTLSGDFLHFLPRVFSAVGNICYTPSKLIEYTDFATSACVLAAECTIFKDA      2917
MT040333.1      VPGLPGTIFRTLSGDFLHFLPRVFSAVGNICYTPSKLIEYTDFATSACVLAAECTIFKDA      2917
MN996532.2      VPGLPGTILRTTNGDFLHFLPRVFSAVGNICYTPSKLIEYTDFATSACVLAAECTIFKDA      2924
MN988713.1      VPGLPGTILRTTNGDFLHFLPRVFSAVGNICYTPSKLIEYTDFATSACVLAAECTIFKDA      2925
MT093571.1      VPGLPGTILRTTNGDFLHFLPRVFSAVGNICYTPSKLIEYTDFATSACVLAAECTIFKDA      2925
MN996529.1      VPGLPGTILRTTNGDFLHFLPRVFSAVGNICYTPSKLIEYTDFATSACVLAAECTIFKDA      2925
MT072688.1      VPGLPGTILRTTNGDFLHFLPRVFSAVGNICYTPSKLIEYTDFATSACVLAAECTIFKDA      2925
NC_045512.2     VPGLPGTILRTTNGDFLHFLPRVFSAVGNICYTPSKLIEYTDFATSACVLAAECTIFKDA      2925
MN994467.1      VPGLPGTILRTTNGDFLHFLPRVFSAVGNICYTPSKLIEYTDFATSACVLAAECTIFKDA      2925
MG772933.1      VPGLPGTILRTINGDFLHFLPRVFSAVGNICYTPSKLIEYTDFATSACVLAAECTIFKDA      2921
MG772934.1      VPGLPGTILRTINGDFLHFLPRVFSAVGNICYTPSKLIEYTDFATSACVLAAECTIFKDS      2899
NC_014470.1     VPGLPGTVRRASNGDFLHFLPRVFSAVGNICYTPAKLIEYTDFATSACVLAAECTIFKDA      2893
GQ153542.1      VPGLPGTVLRALNGDFLHFLPRVFSAVGNICYTPSKLIEYSDFSTSACVLAAECTIFKDA      2900
DQ022305        VPGLPGTVLRALNGDFLHFLPRVFSAVGNICYTPSKLIEYSDFATSACVLAAECTIFKDA      2896
KF569996.1      VPGLPGTVLRAINGDFLHFLPRVFSAVGNICYTPSKLIEYSDFATSACVLAAECTIFKDA      2902
KP886809.1      VPGLPGTVLRAINGDFLHFLPRVFSAVGNICYTPSKLIEYSDFATSACVLAAECTIFKDA      2902
AY278488.2      VPGLPGTVLRAINGDFLHFLPRVFSAVGNICYTPSKLIEYSDFATSACVLAAECTIFKDA      2902
AY485277.1      VPGLPGTVLRAINGDFLHFLPRVFSAVGNICYTPSKLIEYSDFATSACVLAAECTIFKDA      2902
AP006560.1      VPGLPGTVLRAINGDFLHFLPRVFSAVGNICYTPSKLIEYSDFATSACVLAAECTIFKDA      2902
AP006557.1      VPGLPGTVLRAINGDFLHFLPRVFSAVGNICYTPSKLIEYSDFATSACVLAAECTIFKDA      2902
AY274119        VPGLPGTVLRAINGDFLHFLPRVFSAVGNICYTPSKLIEYSDFATSACVLAAECTIFKDA      2902
AY572038.1      VPGLPGTVLRAINGDFLHFLPRVFSAVGNICYTPSKLIEYSDFATSACVLAAECTIFKDA      2902
AY572034.1      VPGLPGTVLRAINGDFLHFLPRVFSAVGNICYTPSKLIEYSDFATSACVLAAECTIFKDA      2902
FJ588686.1      VPGLPGTVLRAINGDFLHFLPRVFSAVGNICYTPSKLIEYSDFATSACVLAAECTIFKDA      2709
KY417145.1      VPGLPGTVLRAINGDFLHFLPRVFSAVGNICYTPSKLIEYSDFATSACVLAAECTIFKDA      2902
KY417144.1      VPGLPGTVLRAINGDFLHFLPRVFSAVGNICYTPSKLIEYSDFATSACVLAAECTIFKDA      2902
KY417147.1      VPGLPGTVLRAINGDFLHFLPRVFSAVGNICYTPSKLIEYSDFATSACVLAAECTIFKDA      2902
KY417148.1      VPGLPGTVLRAINGDFLHFLPRVFSAVGNICYTPSKLIEYSDFATSACVLAAECTIFKDA      2902
KY417143.1      VPGLPGTVLRAINGDFLHFLPRVFSAVGNICYTPSKLIEYSDFATSACVLAAECTIFKDA      2902
KT444582.1      VPGLPGTVLRAINGDFLHFLPRVFSAVGNICYTPSKLIEYSDFATSACVLAAECTIFKDA      2902
KC881005.1      VPGLPGTVLRAINGDFLHFLPRVFSAVGNICYTPSKLIEYSDFATSACVLAAECTIFKDA      2902
KC881006.1      VPGLPGTVLRAINGDFLHFLPRVFSAVGNICYTPSKLIEYSDFATSACVLAAECTIFKDA      2902
KF367457.1      VPGLPGTVLRAINGDFLHFLPRVFSAVGNICYTPSKLIEYSDFATSACVLAAECTIFKDA      2902
KY417152.1      VPGLPGTVLRAINGDFLHFLPRVFSAVGNICYTPSKLIEYSDFATSACVLAAECTIFKDA      2902
KY417146.1      VPGLPGTVLRAINGDFLHFLPRVFSAVGNICYTPSKLIEYSDFATSACVLAAECTIFKDA      2902
KY417151.1      VPGLPGTVLRAINGDFLHFLPRVFSAVGNICYTPSKLIEYSDFATSACVLAAECTIFKDA      2902
KY417142.1      VPGLPGTVLRAINGDFLHFLPRVFSAVGNICYTPSKLIEYSDFATSACVLAAECTIFKDA      2902
                *******: *: .******** *:**********:*****:**:***************:
```

```
MW532698.1      AGKPVPYCYDTNVLEGSVPYESLRPDTRYVLMDGSIIQFPNTYLEGSVRVVTTFDSEYCR      2977
MT040336.1      AGKPVPYCYDTNVLEGSVPYESLRPDTRYVLMDGSIIQFPNTYLEGSVRVVTTFDSEYCR      2977
MT040335.1      AGKPVPYCYDTNVLEGSVPYESLRPDTRYVLMDGSIIQFPNTYLEGSVRVVTTFDSEYCR      2977
MT040334.1      AGKPVPYCYDTNVLEGSVPYESLRPDTRYVLMDGSIIQFPNTYLEGSVRVVTTFDSEYCR      2977
MT040333.1      AGKPVPYCYDTNVLEGSVPYESLRPDTRYVLMDGSIIQFPNTYLEGSVRVVTTFDSEYCR      2977
MN996532.2      SGKPVPYCYDTNVLEGSVAYESLRPDTRYVLMDGSIIQFPNTYLEGSVRVVTTFDSEYCR      2984
MN988713.1      SGKPVPYCYDTNVLEGSVAYESLRPDTRYVLMDGSIIQFPNTYLEGSVRVVTTFDSEYCR      2985
MT093571.1      SGKPVPYCYDTNVLEGSVAYESLRPDTRYVLMDGSIIQFPNTYLEGSVRVVTTFDSEYCR      2985
MN996529.1      SGKPVPYCYDTNVLEGSVAYESLRPDTRYVLMDGSIIQFPNTYLEGSVRVVTTFDSEYCR      2985
MT072688.1      SGKPVPYCYDTNVLEGSVAYESLRPDTRYVLMDGSIIQFPNTYLEGSVRVVTTFDSEYCR      2985
NC_045512.2     SGKPVPYCYDTNVLEGSVAYESLRPDTRYVLMDGSIIQFPNTYLEGSVRVVTTFDSEYCR      2985
MN994467.1      SGKPVPYCYDTNVLEGSVAYESLRPDTRYVLMDGSIIQFPNTYLEGSVRVVTTFDSEYCR      2985
MG772933.1      SGKPVPYCYDTNVLEGSVAYESLRPDTRYVLMDGSIIQFPNTYLEGSVRVVTTFDSEYCR      2981
MG772934.1      SGKPVPYCYDTNVLEGSVAYESLRPDIRYVLMDGSIIQFPNTYLEGSVRVVTTFDSEYCR      2959
NC_014470.1     QGKPVPYCYDTNLLEGSISYSELRPDTRYVLMDGSIIQFPSTYLEGSVRVVTTFDSEYCR      2953
GQ153542.1      MGKPVPYCYDTNLLEGSISYSELRPDTRYVLMDGSIIQFPNTHLEGSVRVVTTFDAEYCR      2960
DQ022305        MGKPVPYCYDTNLLEGSISYSELRPDTRYVLMDGSIIQFPNTYLEGSVRVVTTFDAEYCR      2956
KF569996.1      MGKPVPYCYDTNLLEGSISYSELRPDTRYVLMDGSIIQFPNTYLEGSVRVVTTFDAEYCR      2962
KP886809.1      MGKPVPYCYDTNLLEGSISYSELRPDTRYVLMDGSIIQFPNTYLEGSVRVVTTFDAEYCR      2962
AY278488.2      MGKPVPYCYDTNLLEGSISYSELRPDTRYVLMDGSIIQFPNTYLEGSVRVVTTFDAEYCR      2962
AY485277.1      MGKPVPYCYDTNLLEGSISYSELRPDTRYVLMDGSIIQFPNTYLEGSVRVVTTFDAEYCR      2962
AP006560.1      MGKPVPYCYDTNLLEGSISYSELRPDTRYVLMDGSIIQFPNTYLEGSVRVVTTFDAEYCR      2962
AP006557.1      MGKPVPYCYDTNLLEGSISYSELRPDTRYVLMDGSIIQFPNTYLEGSVRVVTTFDAEYCR      2962
AY274119        MGKPVPYCYDTNLLEGSISYSELRPDTRYVLMDGSIIQFPNTYLEGSVRVVTTFDAEYCR      2962
AY572038.1      MGKPVPYCYDTNLLEGSISYSELRPDTRYVLMDGSIIQFPNTYLEGSVRVVTTFDAEYCR      2962
AY572034.1      MGKPVPYCYDTNLLEGSISYSELRPDTRYVLMDGSIIQFPNTYLEGSVRVVTTFDAEYCR      2962
FJ588686.1      MGKPVPYCYDTNLLEGSISYSELRPDTRYVLMDGSIIQFPNTYLEGSVRVVTTFDAEYCR      2769
KY417145.1      MGKPVPYCYDTNLLEGSISYSELRPDTRYVLMDGSIIQFPNTYLEGSVRVVTTFDAEYCR      2962
KY417144.1      MGKPVPYCYDTNLLEGSISYSELRPDTRYVLMDGSIIQFPNTYLEGSVRVVTTFDAEYCR      2962
KY417147.1      MGKPVPYCYDTNLLEGSISYSELRPDTRYVLMDGSIIQFPNTYLEGSVRVVTTFDAEYCR      2962
KY417148.1      MGKPVPYCYDTNLLEGSISYSELRPDTRYVLMDGSIIQFPNTYLEGSVRVVTTFDAEYCR      2962
KY417143.1      MGKPVPYCYDTNLLEGSISYSELRPDTRYVLMDGSIIQFPNTYLEGSVRVVTTFDAEYCR      2962
KT444582.1      MGKPVPYCYDTNLLEGSISYSELRPDTRYVLMDGSIIQFPNTYLEGSVRVVTTFDAEYCR      2962
KC881005.1      MGKPVPYCYDTNLLEGSISYSELRPDTRYVLMDGSIIQFPNTYLEGSVRVVTTFDAEYCR      2962
KC881006.1      MGKPVPYCYDTNLLEGSISYSELRPDTRYVLMDGSIIQFPNTYLEGSVRVVTTFDAEYCR      2962
KF367457.1      MGKPVPYCYDTNLLEGSISYSELRPDTRYVLMDGSIIQFPNTYLEGSVRVVTTFDAEYCR      2962
KY417152.1      MGKPVPYCYDTNLLEGSISYSELRPDTRYVLMDGSIIQFPNTYLEGSVRVVTTFDAEYCR      2962
KY417146.1      KGKPVPYCYDTNLLEGSISYSELRPDTRYVLMDGSIIQFPNTYLEGSVRVVTTFDAEYCR      2962
KY417151.1      MGKPVPYCYDTNLLEGSISYSELRPDTRYVLMDGSIIQFPNTYLEGSVRVVTTFDAEYCR      2962
KY417142.1      MGKPVPYCYDTNLLEGSISYSELRPDTRYVLMDGSIIQFPNTYLEGSVRVVTTFDAEYCR      2962
                 ***********:****: *..**** *************.*:************:****
```

```
MW532698.1        HGTCEKSEAGICVSTSGRWVLNNDYYRSLPGVFCGVDSVNLLTNMFTPLIQPIGALDISA         3037
MT040336.1        HGTCEKSEAGICVSTSGRWVLNNDYYRSLPGVFCGVDSVNLLTNMFTPLIQPIGALDISA         3037
MT040335.1        HGTCEKSEAGICVSTSGRWVLNNDYYRSLPGVFCGVDSVNLLTNMFTPLIQPIGALDISA         3037
MT040334.1        HGTCEKSEAGICVSTSGRWVLNNDYYRSLPGVFCGVDSVNLLTNMFTPLIQPIGALDISA         3037
MT040333.1        HGTCEKSEAGICVSTSGRWVLNNDYYRSLPGVFCGVDSVNLLTNMFTPLIQPIGALDISA         3037
MN996532.2        HGTCERSEAGVCVSTSGRWVLNNDYYRSLPGVFCGVDAVNLLTNMFTPLIQPIGALDISA         3044
MN988713.1        HGTCERSEAGVCVSTSGRWVLNNDYYRSLPGVFCGVDAVNLLTNMFTPLIQPIGALDISA         3045
MT093571.1        HGTCERSEAGVCVSTSGRWVLNNDYYRSLPGVFCGVDAVNLLTNMFTPLIQPIGALDISA         3045
MN996529.1        HGTCERSEAGVCVSTSGRWVLNNDYYRSLPGVFCGVDAVNLLTNMFTPLIQPIGALDISA         3045
MT072688.1        HGTCERSEAGVCVSTSGRWVLNNDYYRSLPGVFCGVDAVNLLTNMFTPLIQPIGALDISA         3045
NC_045512.2       HGTCERSEAGVCVSTSGRWVLNNDYYRSLPGVFCGVDAVNLLTNMFTPLIQPIGALDISA         3045
MN994467.1        HGTCERSEAGVCVSTSGRWVLNNDYYRSLPGVFCGVDAVNLLTNMFTPLIQPIGALDISA         3045
MG772933.1        HGTCERSEAGICVSTSGRWVLNNDYYRSLPGVFCGVDAVNLLTNMFTPLIQPIGALDISA         3041
MG772934.1        HGTCERSEAGICVSTSGRWVLNNDYYRSLPGVFCGVDAVNLLTNMFTPLIQPIGALDISA         3019
NC_014470.1       HGTCERSDAGVCLSTNGRWVLNNDYYRSIPGVFCGADASDLLFNIFTPLVRPVGTLDISA         3013
GQ153542.1        HGTCERSEAGVCLSTSGRWVLNNEHYRALPGVFCGVDAMNLIANIFTPLVQPVGALDVSA         3020
DQ022305          HGTCERSEVGVCLSTSGRWVLNNEHYRALPGVFCGVDAMNLIANIFTPLVQPVGALDVSA         3016
KF569996.1        HGTCERSEAGICLSTSGRWVLNNEHYRALPGVFCGVDAMNLIANIFTPLVQPVGALDVSA         3022
KP886809.1        HGTCERSEVGICLSTSGRWVLNNEHYRALPGVFCGVDAMNLIANIFTPLVQPVGALDVSA         3022
AY278488.2        HGTCERSEVGICLSTSGRWVLNNEHYRALSGVFCGVDAMNLIANIFTPLVQPVGALDVSA         3022
AY485277.1        HGTCERSEVGICLSTSGRWVLNNEHYRALSGVFCGVDAMNLIANIFTPLVQPVGALDVSA         3022
AP006560.1        HGTCERSEVGICLSTSGRWVLNNEHYRALSGVFCGVDAMNLIANIFTPLVQPVGALDVSA         3022
AP006557.1        HGTCERSEVGICLSTSGRWVLNNEHYRALSGVFCGVDAMNLIANIFTPLVQPVGALDVSA         3022
AY274119          HGTCERSEVGICLSTSGRWVLNNEHYRALSGVFCGVDAMNLIANIFTPLVQPVGALDVSA         3022
AY572038.1        HGTCERSEAGICLSTSGRWVLNNEHYRALSGVFCGVDAMNLIANIFTPLVQPVGALDVSA         3022
AY572034.1        HGTCERSEAGICLSTSGRWVLNNEHYRALSGVFCGVDAMNLIANIFTPLVQPVGALDVSA         3022
FJ588686.1        HGTCERSEAGICLSTSGRWVLNNEHYRALPGVFCGVDAMNLIANIFTPLVQPVGALDVSA         2829
KY417145.1        HGTCERSEAGICLSTSGRWVLNNEHYRALPGVFCGVDAMNLIANIFTPLVQPVGALDVSA         3022
KY417144.1        HGTCERSEAGICLSTSGRWVLNNEHYRALPGVFCGVDAMNLIANIFTPLVQPVGALDVSA         3022
KY417147.1        HGTCERSEAGICLSTSGRWVLNNEHYRALPGVFCGVDAMNLIANIFTPLVQPVGALDVSA         3022
KY417148.1        HGTCERSEAGICLSTSGRWVLNNEHYRALPGVFCGVDAMNLIANIFTPLVQPVGALDVSA         3022
KY417143.1        HGTCERSEAGICLSTSGRWVLNNEHYRALPGVFCGVDAMNLIANIFTPLVQPVGALDVSA         3022
KT444582.1        HGTCERSEAGICLSTSGRWVLNNEHYRALPGVFCGVDAMNLIANIFTPLVQPVGALDVSA         3022
KC881005.1        HGTCERSEAGICLSTSGRWVLNNEHYRALPGVFCGVDAMNLIANIFTPLVQPVGALDVSA         3022
KC881006.1        HGTCERSEAGICLSTSGRWVLNNEHYRALPGVFCGVDAMNLIANIFTPLVQPVGALDVSA         3022
KF367457.1        HGTCERSEAGICLSTSGRWVLNNEHYRALPGVFCGVDAMNLIANIFTPLVQPVGALDVSA         3022
KY417152.1        HGTCERSEAGICLSTSGRWVLNNEHYRALPGVFCGVDVMNLIANIFTPLVQPVGALDVSA         3022
KY417146.1        HGTCERSEAGICLSTSGRWVLNNEHYRALPGVFCGVDAMNLIANIFTPLVQPVGALDVSA         3022
KY417151.1        HGTCERSEAGICLSTSGRWVLNNEHYRALPGVFCGVDAMNLIANIFTPLVQPVGALDVSA         3022
KY417142.1        HGTCERSEAGICLSTSGRWVLNNEHYRALPGVFCGVDAMNLIANIFTPLVQPVGALDVSA         3022
                  *****:*:.*:*:**.*******::**:: *****.*  :*: *:****::*:*:**:**
```

```
MW532698.1       SIVAGGLVAIFVTCLAYYFMRFRRAFGEYSHVVAFNTLLFLMSFTVLCLTPVYSFLPGVY        3097
MT040336.1       SIVAGGLVAIFVTCLAYYFMRFRRAFGEYSHVVAFNTLLFLMSFTVLCLTPVYSFLPGVY        3097
MT040335.1       SIVAGGLVAIFVTCLAYYFMRFRRAFGEYSHVVAFNTLLFLMSFTVLCLTPVYSFLPGVY        3097
MT040334.1       SIVAGGLVAIFVTCLAYYFMRFRRAFGEYSHVVAFNTLLFLMSFTVLCLTPVYSFLPGVY        3097
MT040333.1       SIVAGGLVAIFVTCLAYYFMRFRRAFGEYSHVVAFNTLLFLMSFTVLCLTPVYSFLPGVY        3097
MN996532.2       SIVAGGIVAIVVTCLAYYFMRFRRAFGEYSHVVAFNTLLFLMSFTVLCLTPVYSFLPGVY        3104
MN988713.1       SIVAGGIVAIVVTCLAYYFMRFRRAFGEYSHVVAFNTLLFLMSFTVLCLTPVYSFLPGVY        3105
MT093571.1       SIVAGGIVAIVVTCLAYYFMRFRRAFGEYSHVVAFNTLLFLMSFTVLCLTPVYSFLPGVY        3105
MN996529.1       SIVAGGIVAIVVTCLAYYFMRFRRAFGEYSHVVAFNTLLFLMSFTVLCLTPVYSFLPGVY        3105
MT072688.1       SIVAGGIVAIVVTCLAYYFMRFRRAFGEYSHVVAFNTLLFLMSFTVLCLTPVYSFLPGVY        3105
NC_045512.2      SIVAGGIVAIVVTCLAYYFMRFRRAFGEYSHVVAFNTLLFLMSFTVLCLTPVYSFLPGVY        3105
MN994467.1       SIVAGGIVAIVVTCLAYYFMRFRRAFGEYSHVVAFNTLLFLMSFTVLCLTPVYSFLPGVY        3105
MG772933.1       SIVAGGVVAIIVTCLAYYFMRFRRAFGEYSHVVAFNTLLFFMSFTVLCLTPVYSFLPGVY        3101
MG772934.1       SIVAGGVVAIIVTCLAYYFMRFRRAFGEYSHVVAFNTLLFFMSFTVLCLTPVYSFLPGVY        3079
NC_014470.1      SVVAGGLIAILVTCVAYYFMKFRRAFGEYNHVVFANALLFLLSFTILCLTPAYTFLPGIY        3073
GQ153542.1       SVVAGGIIAILVTCAAYYFMKFRRAFGEYNHVVAANALLFLMSFTILCLAPAYSFLPGVY        3080
DQ022305         SVVAGGIIAILVTCAAYYFMKFRRAFGEYNHVVAANALLFLMSFTILCLAPAYSFLPGVY        3076
KF569996.1       SVVAGGIIAILVTCAAYYFMKFRRAFGEYNHVVAANALLFLMSFTILCLAPAYSFLPGVY        3082
KP886809.1       SVVAGGIIAILVTCAAYYFMKFRRAFGEYNHVVAANALLFLMSFTILCLAPAYSFLPGVY        3082
AY278488.2       SVVAGGIIAILVTCAAYYFMKFRRAFGEYNHVVAANALLFLMSFTILCLVPAYSFLPGVY        3082
AY485277.1       SVVAGGIIAILVTCAAYYFMKFRRVFGEYNHVVAANALLFLMSFTILCLVPAYSFLPGVY        3082
AP006560.1       SVVAGGIIAILVTCAAYYFMKFRRVFGEYNHVVAANALLFLMSFTILCLVPAYSFLPGVY        3082
AP006557.1       SVVAGGIIAILVTCAAYYFMKFRRVFGEYNHVVAANALLFLMSFTILCLVPAYSFLPGVY        3082
AY274119         SVVAGGIIAILVTCAAYYFMKFRRVFGEYNHVVAANALLFLMSFTILCLVPAYSFLPGVY        3082
AY572038.1       SAVAGGIIAILVTCAAYYFMKFRRAFGEYNHVVAANALLFLMSFTILCLAPAYSFLPGVY        3082
AY572034.1       SAVAGGIIAILVTCAAYYFMKFRRAFGEYNHVVAANALLFLMSFTILCLAPAYSFLPGVY        3082
FJ588686.1       SVVAGGIIAILVTCAAYYFMKFRRAFGEYNHVVAANALLFFMSFTILCLAPAYSFLPGVY        2889
KY417145.1       SVVAGGIIAILVTCAAYYFMKFRRAFGEYNHVVAANALLFLMSFTILCLAPAYSFLPGVY        3082
KY417144.1       SVVAGGIIAILVTCAAYYFMKFRRAFGEYNHVAAANALLVLMSFTILCLAPAYSFLPGVY        3082
KY417147.1       SVVAGGIIAILVTCAAYYFMKFRRAFGEYNHVVAANALLFLMSFTILCLAPAYSFLPGVY        3082
KY417148.1       SVVAGGIIAILVTCAAYYFMKFRRAFGEYNHVVAANALLFLMSFTILCLAPAYSFLPGVY        3082
KY417143.1       SVVAGGIIAILVTCAAYYFMKFRRAFGEYNHVVAANALLFLMSFTILCLAPAYSFLPGVY        3082
KT444582.1       SVVAGGIIAILVTCAAYYFMKFRRAFGEYNHVVAANALLFLMSFTILCLAPAYSFLPGVY        3082
KC881005.1       SVVAGGIIAILVTCAAYYFMKFRRAFGEYNHVVAANALLFLMSFTILCLAPAYSFLPGVY        3082
KC881006.1       SVVAGGIIAILVTCAAYYFMKFRRAFGEYNHVVAANALLFLMSFTILCLAPAYSFLPGVY        3082
KF367457.1       SVVAGGIIAILVTCAAYYFMKFRRAFGEYNHVVAANALLFLMSFTILCLAPAYSFLPGVY        3082
KY417152.1       SVVAGGIIAILVTCAAYYFMKFRRAFGEYNHVVAANALLFLMSFTILCLAPAYSFLPGVY        3082
KY417146.1       SVVAGGIIAILVTCAAYYFMKFRRAFGEYNHVVAANALLFLMSFTILCLTPAYSFLPGVY        3082
KY417151.1       SVVAGGIIAILVTCAAYYFMKFRRAFGEYNHVVAANALLFLMSFTILCLAPAYSFLPGVY        3082
KY417142.1       SVVAGGIIAILVTCAAYYFMKFRRAFGEYNHVVAANALLFLMSFTILCLAPAYSFLPGVY        3082
                 * ****::**.*** *****:***.****.**.  *:**.::***:***.*.*:****:*
```

```
MW532698.1        SVFYLYLTFYLTNDVSFLAHVQWMVMFTPLVPFWITIVYVICISTKHCYWFFSNYLRRRV        3157
MT040336.1        SVFYLYLTFYLTNDVSFLAHVQWMVMFTPLVPFWITIVYVICISTKHCYWFFSNYLRRRV        3157
MT040335.1        SVFYLYLTFYLTNDVSFLAHVQWMVMFTPLVPFWITIVYVICISTKHCYWFFSNYLRRRV        3157
MT040334.1        SVFYLYLTFYLTNDVSFLAHVQWMVMFTPLVPFWITIVYVICISTKHCYWFFSNYLRRRV        3157
MT040333.1        SVFYLYLTFYLTNDVSFLAHVQWMVMFTPLVPFWITIVYVICISTKHCYWFFSNYLRRRV        3157
MN996532.2        SVIYLYLTFYLTNDVSFLAHIQWMVMFTPLVPFWITIVYVICISTKHFYWFFSNYLKRRV        3164
MN988713.1        SVIYLYLTFYLTNDVSFLAHIQWMVMFTPLVPFWITIAYIICISTKHFYWFFSNYLKRRV        3165
MT093571.1        SVIYLYLTFYLTNDVSFLAHIQWMVMFTPLVPFWITIAYIICISTKHFYWFFSNYLKRRV        3165
MN996529.1        SVIYLYLTFYLTNDVSFLAHIQWMVMFTPLVPFWITIAYIICISTKHFYWFFSNYLKRRV        3165
MT072688.1        SVIYLYLTFYLTNDVSFLAHIQWMVMFTPLVPFWITIAYIICISTKHFYWFFSNYLKRRV        3165
NC_045512.2       SVIYLYLTFYLTNDVSFLAHIQWMVMFTPLVPFWITIAYIICISTKHFYWFFSNYLKRRV        3165
MN994467.1        SVIYLYLTFYLTNDVSFLAHIQWMVMFTPLVPFWITIAYIICISTKHFYWFFSNYLKRRV        3165
MG772933.1        SVIYLYLTFYLTNDVSFLAHIQWMVMFTPLVPFWMTIVYVICISTKHFYWFFSNYLKRRV        3161
MG772934.1        SVIYLYLTFYLTNDVSFLAHIQWMVMFTPLVPFWMTIVYVICISTKHFYWFFSNYLKRRV        3139
NC_014470.1       SLLYLYLTFYFTNDVSFLAHLQWLAMFSPIVPFWITVTYVVCISIKHCHWFFSNYLKKRV        3133
GQ153542.1        SIFYLYLTFYFTNDVSFLAHLQWFAMFSPIVPFWITAIYVFCISLKHFHWFFSNYLKKRV        3140
DQ022305          SIFYLYLTFYFTNDVSFLAHLQWFAMFSPIVPFWITAIYVFCISLKHFHWFFSNYLKKRV        3136
KF569996.1        SVFYLYLTFYFTNDVSFLAHLQWFAMFSPIVPFWITAIYVFCISLKHCHWFFNNYLRKRV        3142
KP886809.1        SVFYLYLTFYFTNDVSFLAHLQWFAMFSPIVPFWITAIYAFCISLKHCHWFFNNYLRKRV        3142
AY278488.2        SVFYLYLTFYFTNDVSFLAHLQWFAMFSPIVPFWITAIYVFCISLKHCHWFFNNYLRKRV        3142
AY485277.1        SVFYLYLTFYFTNDVSFLAHLQWFAMFSPIVPFWITAIYVFCISLKHCHWFFNNYLRKRV        3142
AP006560.1        SVFYLYLTFYFTNDVSFLAHLQWFAMFSPIVPFWITAIYVFCISLKHCHWFFNNYLRKRV        3142
AP006557.1        SVFYLYLTFYFTNDVSFLAHLQWFAMFSPIVPFWITAIYVFCISLKHCHWFFNNYLRKRV        3142
AY274119          SVFYLYLTFYFTNDVSFLAHLQWFAMFSPIVPFWITAIYVFCISLKHCHWFFNNYLRKRV        3142
AY572038.1        SVFYLYLTFYFTNDVSFLAHLQWFAMFSPIVPFWITAIYVFCISLKHCHWFFNNYLRKRV        3142
AY572034.1        SVFYLYLTFYFTNDVSFLAHLQWFAMFSPIVPFWITAIYVFCISLKHCHWFFNNYLRKRV        3142
FJ588686.1        SVFYLYLTFYFTNDVSFLAHLQWFAMFSPIVPFWITAIYVFCISLKHCHWFFNNYLRKRV        2949
KY417145.1        SVFYLYLTFYFTNDVSFLAHLQWFAMFSPIVPFWITAIYVFCISLKHCHWFFNNYLRKRV        3142
KY417144.1        SVFYLYLTFYFTNDVSFLAHLQWFAMFSPIVPFWITAIYVFCISLKHCHWFFNNYLRKRV        3142
KY417147.1        SVFYLYLTFYFTNDVSFLAHLQWFAMFSPIVPFWITAIYVFCISLKHCHWFFNNYLRKRV        3142
KY417148.1        SVFYLYLTFYFTNDVSFLAHLQWFAMFSPIVPFWITAIYVFCISLKHCHWFFNNYLRKRV        3142
KY417143.1        SVFYLYLTFYFTNDVSFLAHLQWFAMFSPIVPFWITVIYVFCISLKHCHWFFNNYLRKRV        3142
KT444582.1        SVFYLYLTFYFTNDVSFLAHLQWFAMFSPIVPFWITVIYVFCISLKHCHWFFNNYLRKRV        3142
KC881005.1        SVFYLYLTFYFTNDVSFLAHLQWFAMFSPIVPFWITAIYVFCISLKHCHWFFNNYLRKRV        3142
KC881006.1        SVFYLYLTFYFTNDVSFLAHLQWFAMFSPIVPFWITAIYVFCISLKHCHWFFNNYLRKRV        3142
KF367457.1        SVFYLYLTFYFTNDVSFLAHLQWFAMFSPIVPFWITAIYVFCISLKHCHWFFNNYLRKRV        3142
KY417152.1        SVFYLYLTFYFTNDVSFLAHLQWFAMFSPIVPFWITVIYVFCISLKHCHWFFNNYLRKRV        3142
KY417146.1        SVFYLYLTFYFTNDVSFLAHLQWFAMFSPIVPFWITAIYVFCISLKHCHWFFNNYLRKRV        3142
KY417151.1        SVFYLYLTFYFTNDVSFLAHLQWFAMFSPIVPFWITAIYVFCISLKHCHWFFNNYLRKRV        3142
KY417142.1        SVFYLYLTFYFTNDVSFLAHLQWFAMFSPIVPFWITAIYVFCISLKHCHWFFNNYLRKRV        3142
                  *::*******:*********:**:.**:*:****:*  * .*** ** :***.***::**
```

```
MW532698.1        VFNGTSFSTFEEAALCTFLLNKEMYLKLRSETLLPLTQYNRYLALYNKYKYFSGAMDTTS         3217
MT040336.1        VFNGTSFSTFEEAALCTFLLNKEMYLKLRSETLLPLTQYNRYLALYNKYKYFSGAMDTTS         3217
MT040335.1        VFNGTSFSTFEEAALCTFLLNKEMYLKLRSETLLPLTQYNRYLALYNKYKYFSGAMDTTS         3217
MT040334.1        VFNGTSFSTFEEAALCTFLLNKEMYLKLRSETLLPLTQYNRYLALYNKYKYFSGAMDTTS         3217
MT040333.1        VFNGTSFSTFEEAALCTFLLNKEMYLKLRSETLLPLTQYNRYLALYNKYKYFSGAMDTTS         3217
MN996532.2        VFNGVSFSTFEEAALCTFLLNKEMYLKLRSDVLLPLTQYNRYLALYNKYKYFSGAMDTTS         3224
MN988713.1        VFNGVSFSTFEEAALCTFLLNKEMYLKLRSDVLLPLTQYNRYLALYNKYKYFSGAMDTTS         3225
MT093571.1        VFNGVSFSTFEEAALCTFLLNKEMYLKLRSDVLLPLTQYNRYLALYNKYKYFSGAMDTTS         3225
MN996529.1        VFNGVSFSTFEEAALCTFLLNKEMYLKLRSDVLLPLTQYNRYLALYNKYKYFSGAMDTTS         3225
MT072688.1        VFNGVSFSTFEEAALCTFLLNKEMYLKLRSDVLLPLTQYNRYLALYNKYKYFSGAMDTTS         3225
NC_045512.2       VFNGVSFSTFEEAALCTFLLNKEMYLKLRSDVLLPLTQYNRYLALYNKYKYFSGAMDTTS         3225
MN994467.1        VFNGVSFSTFEEAALCTFLLNKEMYLKLRSDVLLPLTQYNRYLALYNKYKYFSGAMDTTS         3225
MG772933.1        VFNGVSFSTFEEAALCTFLLNKEMYLKLRSDVLLPLTQYNRYLALYNKYKYFSGAMDTTS         3221
MG772934.1        VFNGVSFSTFEEAALCTFLLNKEMYLKLRSDVLLPLTQYNRYLALYNKYKYFSGAMDTTS         3199
NC_014470.1       VFNGVTFSTFEEAALCTFLLNKEMYLKLRSETLLPLTQYNRYLALYNKYKYFSGALDTTS         3193
GQ153542.1        MFNGVTFSTFEEAALCTFLLNKEMYLKLRSETLLPLTQYNRYLALYNKYKYFSGALDTTS         3200
DQ022305          MFNGVTFSTFEEAALCTFLLNKEMYLRLRSETLLPLTQYNRYLALYNKYKYFSGALDTTS         3196
KF569996.1        MFNGVTFSTFEEAALCTFLLNKEMYLKLRSETLLPLTQYNRYLALYNKYKYFSGALDTTS         3202
KP886809.1        MFNGVTFSTFEEAALCTFLLNKEMYLKLRSETLLPLTQYNRYLALYNKYKYFSGALDTTS         3202
AY278488.2        MFNGVTFSTFEEAALCTFLLNKEMYLKLRSETLLPLTQYNRYLALYNKYKYFSGVLDTTS         3202
AY485277.1        MFNGVTFSTFEEAALCTFLLNKEMYLKLRSETLLPLTQYNRYLALYNKYKYFSGALDTTS         3202
AP006560.1        MFNGVTFSTFEEAALCTFLLNKEMYLKLRSETLLPLTQYNRYLALYNKYKYFSGALDTTS         3202
AP006557.1        MFNGVTFSTFEEAALCTFLLNKEMYLKLRSETLLPLTQYNRYLALYNKYKYFSGALDTTS         3202
AY274119          MFNGVTFSTFEEAALCTFLLNKEMYLKLRSETLLPLTQYNRYLALYNKYKYFSGALDTTS         3202
AY572038.1        MFNGVTFSTFEEAALCTFLLNKEMYLKLRSETLLPLTQYNRYLALYNKYKYFSGALDTTS         3202
AY572034.1        MFNGVTFSTFEEAALCTFLLNKEMYLKLRSETLLPLTQYNRYLALYNKYKYFSGALDTTS         3202
FJ588686.1        MFNGVTFSTFEEAALCTFLLNKEMYLKLRSETLLPLTQYNRYLALYNKYKYFSGALDTTS         3009
KY417145.1        MFNGVTFSTFEEAALCTFLLNKEMYLKLRSETLLPLTQYNRYLALYNKYKYFSGALDTTS         3202
KY417144.1        MFNGVTFSTFEEAALCTFLLNKEMYLKLRSETLLPLTQYNRYLALYNKYKYFSGALDTTS         3202
KY417147.1        MFNGVTFSTFEEAALCTFLLNKEMYLKLRSETLLPLTQYNRYLALYNKYKYFSGALDTTS         3202
KY417148.1        MFNGVTFSTFEEAALCTFLLNKEMYLKLRSETLLPLTQYNRYLALYNKYKYFSGALDTTS         3202
KY417143.1        MFNGVTFSTFEEAALCTFLLNKEMYLKLRSETLLPLTQYNRYLALYNKYKYFSGALDTTS         3202
KT444582.1        MFNGVTFSTFEEAALCTFLLNKEMYLKLRSETLLPLTQYNRYLALYNKYKYFSGALDTTS         3202
KC881005.1        MFNGVTFSTFEEAALCTFLLNKEMYLKLRSETLLPLTQYNRYLALYNKYKYFSGALDTTS         3202
KC881006.1        MFNGVTFSTFEEAALCTFLLNKEMYLKLRSETLLPLTQYNRYLALYNKYKYFSGALDTTS         3202
KF367457.1        MFNGVTFSTFEEAALCTFLLNKEMYLKLRSETLLPLTQYNRYLALYNKYKYFSGALDTTS         3202
KY417152.1        MFNGVTFSTFEEAALCTFLLNKEMYLKLRSETLLPLTQYNRYLALYNKYKYFSGALDTTS         3202
KY417146.1        MFNGVTFSTFEEAALCTFLLNKEMYLKLRSETLLPLTQYNRYLALYNKYKYFSGALDTTS         3202
KY417151.1        MFNGVTFSTFEEAALCTFLLNKEMYLKLRSETLLPLTQYNRYLALYNKYKYFSGALDTTS         3202
KY417142.1        MFNGVTFSTFEEAALCTFLLNKEMYLKLRSETLLPLTQYNRYLALYNKYKYFSGALDTTS         3202
                  :***.:********************:***:.**********************.:****
```

```
MW532698.1      YREAACCHLAKALNDFSNSGSDVLYQPPQTSITSAVLQSGFRKMAFPSGKVEGCMVQVTC      3277
MT040336.1      YREAACCHLAKALNDFSNSGSDVLYQPPQTSITSAVLQSGFRKMAFPSGKVEGCMVQVTC      3277
MT040335.1      YREAACCHLAKALNDFSNSGSDVLYQPPQTSITSAVLQSGFRKMAFPSGKVEGCMVQVTC      3277
MT040334.1      YREAACCHLAKALNDFSNSGSDVLYQPPQTSITSAVLQSGFRKMAFPSGKVEGCMVQVTC      3277
MT040333.1      YREAACCHLAKALNDFSNSGSDVLYQPPQTSITSAVLQSGFRKMAFPSGKVEGCMVQVTC      3277
MN996532.2      YREAACCHLAKALNDFSNSGSDVLYQPPQTSITSAVLQSGFRKMAFPSGKVEGCMVQVTC      3284
MN988713.1      YREAACCHLAKALNDFSNSGSDVLYQPPQTSITSAVLQSGFRKMAFPSGKVEGCMVQVTC      3285
MT093571.1      YREAACCHLAKALNDFSNSGSDVLYQPPQTSITSAVLQSGFRKMAFPSGKVEGCMVQVTC      3285
MN996529.1      YREAACCHLAKALNDFSNSGSDVLYQPPQTSITSAVLQSGFRKMAFPSGKVEGCMVQVTC      3285
MT072688.1      YREAACCHLAKALNDFSNSGSDVLYQPPQTSITSAVLQSGFRKMAFPSGKVEGCMVQVTC      3285
NC_045512.2     YREAACCHLAKALNDFSNSGSDVLYQPPQTSITSAVLQSGFRKMAFPSGKVEGCMVQVTC      3285
MN994467.1      YREAACCHLAKALNDFSNSGSDVLYQPPQTSITSAVLQSGFRKMAFPSGKVEGCMVQVTC      3285
MG772933.1      YREAACCHLAKAINDFSNSGSDVLYQPPQTSITSAVLQSGFRKMAFPSGKVEGCMVQVTC      3281
MG772934.1      YREAACCHLAKALNDFSNSGSDVLYQPPQTSITSAILQSGFRKMAFPSGKVEGCMVQVTC      3259
NC_014470.1     YREAACCHLAKALNDFSNSGADVLYQPPQTSITSAVLQSGFRKMAFPSGKVEGCMVQVTC      3253
GQ153542.1      YREAACCHLAKALNDFSNSGADVLYQPPQTSITSAVLQSGFRKMAFPSGKVEGCMVQVTC      3260
DQ022305        YREAACCHLAKALNDFSNSGADVLYQPPQTSITSAVLQSGFRKMAFPSGKVEGCMVQVTC      3256
KF569996.1      YREAACCHLAKALNDFSNSGSDVLYQPPQTSITSAVLQSGFRKMAFPSGKVEGCMVQVTC      3262
KP886809.1      YREAACCHLAKALNDFSNSGSDVLYQPPQTSITSAVLQSGFRKMAFPSGKVEGCMVQVTC      3262
AY278488.2      YREAACCHLAKALNDFSNSGADVLYQPPQTSITSAVLQSGFRKMAFPSGKVEGCMVQVTC      3262
AY485277.1      YREAACCHLAKALNDFSNSGADVLYQPPQTSITSAVLQSGFRKMAFPSGKVEGCMVQVTC      3262
AP006560.1      YREAACCHLAKALNDFSNSGADVLYQPPQTSITSAVLQSGFRKMAFPSGKVEGCMVQVTC      3262
AP006557.1      YREAACCHLAKALNDFSNSGADVLYQPPQTSITSAVLQSGFRKMAFPSGKVEGCMVQVTC      3262
AY274119        YREAACCHLAKALNDFSNSGADVLYQPPQTSITSAVLQSGFRKMAFPSGKVEGCMVQVTC      3262
AY572038.1      YREAACCHLAKALNDFSNSGADVLYQPPQTSITSAVLQSGFRKMAFPSGKVEGCMVQVTC      3262
AY572034.1      YREAACCHLAKALNDFSNSGADVLYQPPQTSITSAVLQSGFRKMAFPSGKVEGCMVQVTC      3262
FJ588686.1      YREAACCHLAKALNDFSNSGADVLYQPPQTSITSAVLQSGFRKMAFPSGKVEGCMVQVTC      3069
KY417145.1      YREAACCHLAKALNDFSNSGADVLYQPPQTSITSAVLQSGFRKMAFPSGKVEGCMVQVTC      3262
KY417144.1      YREAACCHLAKALNDFSNSGADVLYQPPQTSITSAVLQSGFRKMAFPSGKVEGCMVQVTC      3262
KY417147.1      YREAACCHLAKALNDFSNSGADVLYQPPQTSITSAVLQSGFRKMAFPSGKVEGCMVQVTC      3262
KY417148.1      YREAACCHLAKALNDFSNSGADVLYQPPQTSITSAVLQSGFRKMAFPSGKVEGCMVQVTC      3262
KY417143.1      YREAACCHLAKALNDFSNSGADVLYQPPQTSITSAVLQSGFRKMAFPSGKVEGCMVQVTC      3262
KT444582.1      YREAACCHLAKALNDFSNSGADVLYQPPQTSITSAVLQSGFRKMAFPSGKVEGCMVQVTC      3262
KC881005.1      YREAACCHLAKALNDFSNSGADVLYQPPQTSITSAVLQSGFRKMAFPSGKVEGCMVQVTC      3262
KC881006.1      YREAACCHLAKALNDFSNSGADVLYQPPQTSITSAVLQSGFRKMAFPSGKVEGCMVQVTC      3262
KF367457.1      YREAACCHLAKALNDFSNSGADVLYQPPQTSITSAVLQSGFRKMAFPSGKVEGCMVQVTC      3262
KY417152.1      YREAACCHLAKALNDFSNSGADVLYQPPQTSITSAVLQSGFRKMAFPSGKVEGCMVQVTC      3262
KY417146.1      YREAACCHLAKALNDFSNSGADVLYQPPQTSITSAVLQSGFRKMAFPSGKVEGCMVQVTC      3262
KY417151.1      YREAACCHLAKALNDFSNSGADVLYQPPQTSITSAVLQSGFRKMAFPSGKVEGCMVQVTC      3262
KY417142.1      YREAACCHLAKALNDFSNSGADVLYQPPQTSITSAVLQSGFRKMAFPSGKVEGCMVQVTC      3262
                ************:*******:**************:************************
```

```
MW532698.1      GTTTLNGLWLDDVVYCPRHVICTAEDMLNPNYEDLLIRKSNHNFLVQAGNVHLRVIGHSM     3337
MT040336.1      GTTTLNGLWLDDVVYCPRHVICTAEDMLNPNYEDLLIRKSNHNFLVQAGNVHLRVIGHSM     3337
MT040335.1      GTTTLNGLWLDDVVYCPRHVICTAEDMLNPNYEDLLIRKSNHNFLVQAGNVHLRVIGHSM     3337
MT040334.1      GTTTLNGLWLDDVVYCPRHVICTAEDMLNPNYEDLLIRKSNHNFLVQAGNVHLRVIGHSM     3337
MT040333.1      GTTTLNGLWLDDVVYCPRHVICTAEDMLNPNYEDLLIRKSNHNFLVQAGNVHLRVIGHSM     3337
MN996532.2      GTTTLNGLWLDDVVYCPRHVICTSEDMLNPNYEDLLIRKSNHNFLVQAGNVQLRVIGHSM     3344
MN988713.1      GTTTLNGLWLDDVVYCPRHVICTSEDMLNPNYEDLLIRKSNHNFLVQAGNVQLRVIGHSM     3345
MT093571.1      GTTTLNGLWLDDVVYCPRHVICTSEDMLNPNYEDLLIRKSNHNFLVQAGNVQLRVIGHSM     3345
MN996529.1      GTTTLNGLWLDDVVYCPRHVICTSEDMLNPNYEDLLIRKSNHNFLVQAGNVQLRVIGHSM     3345
MT072688.1      GTTTLNGLWLDDVVYCPRHVICTSEDMLNPNYEDLLIRKSNHNFLVQAGNVQLRVIGHSM     3345
NC_045512.2     GTTTLNGLWLDDVVYCPRHVICTSEDMLNPNYEDLLIRKSNHNFLVQAGNVQLRVIGHSM     3345
MN994467.1      GTTTLNGLWLDDVVYCPRHVICTSEDMLNPNYEDLLIRKSNHNFLVQAGNVQLRVIGHSM     3345
MG772933.1      GTTTLNGLWLDDVVYCPRHVICTSEDMLNPNYEDLLIRKSNHNFLVQAGNVQLRVVGHSM     3341
MG772934.1      GTTTLNGLWLDDVVYCPRHVICTSEDMLNPNYEDLLIRKSNHNFLVQAGNVQLRVVGHSM     3319
NC_014470.1     GTTTLNGLWLDDVVYCPRHVICTLEDMLNPNYDDLLIRKSNHNFLVQASNVQLRVIGHTM     3313
GQ153542.1      GTTTLNGLWLDDTVYCPRHVICTAEDMLNPNYDDLLIRKSNHSFLVQAGNVQLRVIGHSM     3320
DQ022305        GTTTLNGLWLDDTVYCPRHVVCTAEDMLNPNYDDLLIRKSNHSFLVQAGNVQLRVIGHSM     3316
KF569996.1      GTTTLNGLWLDDTVYCPRHVICTAEDMLNPNYEDLLIRKSNHSFLVQAGNVQLRVIGHSM     3322
KP886809.1      GTTTLNGLWLDDTVYCPRHVICTAEDMLNPNYEDLLIRKSNHSFLVQAGNVQLRVIGHSM     3322
AY278488.2      GTTTLNGLWLDDTVYCPRHVICTAEDMLNPNYEDLLIRKSNHSFLVQAGNVQLRVIGHSM     3322
AY485277.1      GTTTLNGLWLDDTVYCPRHVICTAEDMLNPNYEDLLIRKANHSFLVQAGNVQLRVIGHSM     3322
AP006560.1      GTTTLNGLWLDDTVYCPRHVICTAEDMLNPNYEDLLIRKSNHSFLVQAGNVQLRVIGHSM     3322
AP006557.1      GTTTLNGLWLDDTVYCPRHVICTAEDMLNPNYEDLLIRKSNHSFLVQAGNVQLRVIGHSM     3322
AY274119        GTTTLNGLWLDDTVYCPRHVICTAEDMLNPNYEDLLIRKSNHSFLVQAGNVQLRVIGHSM     3322
AY572038.1      GTTTLNGLWLDDTVYCPRHVICTAEDMLNPNYEDLLIRKSNHSFLVQAGNVQLRVIGHSM     3322
AY572034.1      GTTTLNGLWLDDTVYCPRHVICTAEDMLNPNYEDLLIRKSNHSFLVQAGNVQLRVIGHSM     3322
FJ588686.1      GTTTLNGLWLDDTVYCPRHVICTAEDMLNPNYEDLLIRKSNHSFLVQAGNVQLRVIGHSM     3129
KY417145.1      GTTTLNGLWLDDTVYCPRHVICTAEDMLNPNYEDLLIRKSNHSFLVQAGNVQLRVIGHSM     3322
KY417144.1      GTTTLNRLWLDDTVYCPRHVICTAEDMLNPNYEDLLIRKSNHSFLVQAGNVQLRVIGHSM     3322
KY417147.1      GTTTLNGLWLDDTVYCPRHVICTAEDMLNPNYEDLLIRKSNHSFLVQAGNVQLRVIGHSM     3322
KY417148.1      GTTTLNGLWLDDTVYCPRHVICTAEDMLNPNYEDLLIRKSNHSFLVQAGNVQLRVIGHSM     3322
KY417143.1      GTTTLNGLWLDDTVYCPRHVICTAEDMLNPNYEDLLIRKSNHSFLVQAGNVQLRVIGHSM     3322
KT444582.1      GTTTLNGLWLDDTVYCPRHVICTAEDMLNPNYEDLLIRKSNHSFLVQAGNVQLRVIGHSM     3322
KC881005.1      GTTTLNGLWLDDTVYCPRHVICTAEDMLNPNYEDLLIRKSNHSFLVQAGNVQLRVIGHSM     3322
KC881006.1      GTTTLNGLWLDDTVYCPRHVICTAEDMLNPNYEDLLIRKSNHSFLVQAGNVQLRVIGHSM     3322
KF367457.1      GTTTLNGLWLDDTVYCPRHVICTAEDMLNPNYEDLLIRKSNHSFLVQAGNVQLRVIGHSM     3322
KY417152.1      GTTTLNGLWLDDTVYCPRHVICTAEDMLNPNYEDLLIRKSNHSFLVQAGNVQLRVIGHSM     3322
KY417146.1      GTTTLNGLWLDDTVYCPRHVICTAEDMLNPNYEDLLIRKSNHSFLVQAGNVQLRVIGHSM     3322
KY417151.1      GTTTLNGLWLDDTVYCPRHVICTAEDMLNPNYEDLLIRKSNHSFLVQAGNVQLRVIGHSM     3322
KY417142.1      GTTTLNGLWLDDTVYCPRHVICTAEDMLNPNYEDLLIRKSNHSFLVQAGNVQLRVIGHSM     3322
                ****** *****.*******:** ********:******:**.*****.**:***:**:*
```

```
MW532698.1       QNCVLKLKVDAANPKTPKYKFVRIQPGQTFSVLACYNGSPSGVYQCAMRPNFTIKGSFLN         3397
MT040336.1       QNCVLKLKVDAANPKTPKYKFVRIQPGQTFSVLACYNGSPSGVYQCAMRPNFTIKGSFLN         3397
MT040335.1       QNCVLKLKVDAANPKTPKYKFVRIQPGQTFSVLACYNGSPSGVYQCAMRPNFTIKGSFLN         3397
MT040334.1       QNCVLKLKVDAANPKTPKYKFVRIQPGQTFSVLACYNGSPSGVYQCAMRPNFTIKGSFLN         3397
MT040333.1       QNCVLKLKVDAANPKTPKYKFVRIQPGQTFSVLACYNGSPSGVYQCAMRPNFTIKGSFLN         3397
MN996532.2       QNCVLKLKVDTANTKTPKYKFVRIQPGQTFSVLACYNGSPSGVYQCAMRPNFTIKGSFLN         3404
MN988713.1       QNCVLKLKVDTANPKTPKYKFVRIQPGQTFSVLACYNGSPSGVYQCAMRPNFTIKGSFLN         3405
MT093571.1       QNCVLKLKVDTANPKTPKYKFVRIQPGQTFSVLACYNGSPSGVYQCAMRPNFTIKGSFLN         3405
MN996529.1       QNCVLKLKVDTANPKTPKYKFVRIQPGQTFSVLACYNGSPSGVYQCAMRPNFTIKGSFLN         3405
MT072688.1       QNCVLKLKVDTANPKTPKYKFVRIQPGQTFSVLACYNGSPSGVYQCAMRPNFTIKGSFLN         3405
NC_045512.2      QNCVLKLKVDTANPKTPKYKFVRIQPGQTFSVLACYNGSPSGVYQCAMRPNFTIKGSFLN         3405
MN994467.1       QNCVLKLKVDTANPKTPKYKFVRIQPGQTFSVLACYNGSPSGVYQCAMRPNFTIKGSFLN         3405
MG772933.1       QNCVLKLKVDTANPKTPKYKFVRIQPGQTFSVLACYNGSPSGVYQCAMRPNFTIKGSFLN         3401
MG772934.1       QNCVLKLKVDTANPKTPKYKFVRIQPGQTFSVLACYNGSPSGVYQCAMRPNFTIKGSFLN         3379
NC_014470.1      QNCLLKLKVDIANPKTPKYKFVRIQPGQTFSVLACYNGAPSGVYQCAMRSNHTIKGSFLN         3373
GQ153542.1       QNCLLRLKVDTSNPKTPKYKFVRIQPGQTFSVLACYNGSPSGVYQCAMRPNYTIKGSFLN         3380
DQ022305         QNCLLRLKVDTSNPKTPKYKFVRIQPGQTFSVLACYNGSPSGVYQCAMRPNHTIKGSFLN         3376
KF569996.1       QNCLLRLKVDTSNPKTPKYKFVHIQPGQTFSVLACYNGSPSGVYQCAMRPNYTIKGSFLN         3382
KP886809.1       QNCLLRLKVDTSNPKTPKYKFVRIQPGQTFSVLACYNGSPSGVYQCAMRPNHTIKGSFLN         3382
AY278488.2       QNCLLRLKVDTSNPKTPKYKFVRIQPGQTFSVLACYNGSPSGVYQCAMRPNHTIKGSFLN         3382
AY485277.1       QNCLLRLKVDTSNPKTPKYKFVRIQPGQTFSVLACYNGSPSGVYQCAMRPNHTIKGSFLN         3382
AP006560.1       QNCLLRLKVDTSNPKTPKYKFVRIQPGQTFSVLACYNGSPSGVYQCAMRPNHTIKGSFLN         3382
AP006557.1       QNCLLRLKVDTSNPKTPKYKFVRIQPGQTFSVLACYNGSPSGVYQCAMRPNHTIKGSFLN         3382
AY274119         QNCLLRLKVDTSNPKTPKYKFVRIQPGQTFSVLACYNGSPSGVYQCAMRPNHTIKGSFLN         3382
AY572038.1       QNCLLRLKVDTSNPKTPKYKFVRIQPGQTFSVLACYNGSPSGVYQCAMRPNHTIKGSFLN         3382
AY572034.1       QNCLLRLKVDTSNPKTPKYKFVRIQPGQTFSVLACYNGSPSGVYQCAMRPNHTIKGSFLN         3382
FJ588686.1       QNCLLRLKVDTSNPKTPKYKFVRIQPGQTFSVLACYNGSPSGVYQCAMRSNHTIKGSFLN         3189
KY417145.1       QNCLLRLKVDTSNPKTPKYKFVRIQPGQTFSVLACYNGSPSGVYQCAMRPNHTIKGSFLN         3382
KY417144.1       QNCLLRLKVDTSNPKTPKYKFVRIQPGQTFSVLACYNGSPSGVYQCAMRPNHTIKGSFLN         3382
KY417147.1       QNCLLRLKVDTSNPKTPKYKFVRIQPGQTFSVLACYNGSPSGVYQCAMRPNHTIKGSFLN         3382
KY417148.1       QNCLLRLKVDTSNPKTPKYKFVRIQPGQTFSVLACYNGSPSGVYQCAMRPNHTIKGSFLN         3382
KY417143.1       QNCLLRLKVDTSNPKTPKYKFVRIQPGQTFSVLACYNGSPSGVYQCAMRPNHTIKGSFLN         3382
KT444582.1       QNCLLRLKVDTSNPKTPKYKFVRIQPGQTFSVLACYNGSPSGVYQCAMRPNHTIKGSFLN         3382
KC881005.1       QNCLLRLKVDTSNPKTPKYKFVRIQPGQTFSVLACYNGSPSGVYQCAMRPNHTIKGSFLN         3382
KC881006.1       QNCLLRLKVDTSNPKTPKYKFVRIQPGQTFSVLACYNGSPSGVYQCAMRPNHTIKGSFLN         3382
KF367457.1       QNCLLRLKVDTSNPKTPKYKFVRIQPGQTFSVLACYNGSPSGVYQCAMRPNHTIKGSFLN         3382
KY417152.1       QNCLLRLKVDTSNPKTPKYKFVRIQPGQTFSVLACYNGSPSGVYQCAMRPNHTIKGSFLN         3382
KY417146.1       QNCLLRLKVDTSNPKTPKYKFVRIQPGQTFSVLACYNGSPSGVYQCAMRPNHTIKGSFLN         3382
KY417151.1       QNCLLRLKVDTSNPKTPKYKFVRIQPGQTFSVLACYNGSPSGVYQCAMRPNHTIKGSFLN         3382
KY417142.1       QNCLLRLKVDTSNPKTPKYKFVRIQPGQTFSVLACYNGSPSGVYQCAMRPNHTIKGSFLN         3382
                 ***:*:**** :* ********:***************:********** *.********
```

```
MW532698.1      GSCGSVGFNIDYDCVSFCYMHHMELPTGVHAGTDLEGTFYGPFVDRQTAQAAGTDTVITI      3457
MT040336.1      GSCGSVGFNIDYDCVSFCYMHHMELPTGVHAGTDLEGTFYGPFVDRQTAQAAGTDTVITI      3457
MT040335.1      GSCGSVGFNIDYDCVSFCYMHHMELPTGVHAGTDLEGTFYGPFVDRQTAQAAGTDTVITI      3457
MT040334.1      GSCGSVGFNIDYDCVSFCYMHHMELPTGVHAGTDLEGTFYGPFVDRQTAQAAGTDTVITI      3457
MT040333.1      GSCGSVGFNIDYDCVSFCYMHHMELPTGVHAGTDLEGTFYGPFVDRQTAQAAGTDTVITI      3457
MN996532.2      GSCGSVGFNIDYDCVSFCYMHHMELPTGVHAGTDLEGTFYGPFVDRQTAQAAGTDTTITV      3464
MN988713.1      GSCGSVGFNIDYDCVSFCYMHHMELPTGVHAGTDLEGNFYGPFVDRQTAQAAGTDTTITV      3465
MT093571.1      GSCGSVGFNIDYDCVSFCYMHHMELPTGVHAGTDLEGNFYGPFVDRQTAQAAGTDTTITV      3465
MN996529.1      GSCGSVGFNIDYDCVSFCYMHHMELPTGVHAGTDLEGNFYGPFVDRQTAQAAGTDTTITV      3465
MT072688.1      GSCGSVGFNIDYDCVSFCYMHHMELPTGVHAGTDLEGNFYGPFVDRQTAQAAGTDTTITV      3465
NC_045512.2     GSCGSVGFNIDYDCVSFCYMHHMELPTGVHAGTDLEGNFYGPFVDRQTAQAAGTDTTITV      3465
MN994467.1      GSCGSVGFNIDYDCVSFCYMHHMELPTGVHAGTDLEGNFYGPFVDRQTAQAAGTDTTITV      3465
MG772933.1      GSCGSVGFNIDYDCVSFCYMHHMELPTGVHAGTDLEGTFYGPFVDRQTAQAAGTDTTITV      3461
MG772934.1      GSCGSVGFNIDYDCVSFCYMHHMELPTGVHAGTDLEGTFYGPFVDRQTAQAAGTDTTITV      3439
NC_014470.1     GSCGSVGFNIDYDCVSFCYMHHMELPTGVHAGTDLEGNFYGPFVDRQTAQAAGTDTTITL      3433
GQ153542.1      GSCGSVGFNIDYDCVSFCYMHHMELPTGVHAGTDLEGKFYGPFVDRQTAQAAGTDTTITL      3440
DQ022305        GSCGSVGFNIDYDCVSFCYMHHMELPTGVHAGTDLEGKFYGPFVDRQTAQAAGTDTTITL      3436
KF569996.1      GSCGSVGFNIDYDCVSFCYMHHMELPTGVHAGTDLEGKFYGPFVDRQTAQAAGTDTTITL      3442
KP886809.1      GSCGSVGFNIDYDCVSFCYMHHMELPTGVHAGTDLEGKFYGPFVDRQTAQAAGTDTTITL      3442
AY278488.2      GSCGSVGFNIDYDCVSFCYMHHMELPTGVHAGTDLEGKFYGPFVDRQTAQAAGTDTTITL      3442
AY485277.1      GSCGSVGFNIDYDCVSFCYMHHMELPTGVHAGTDLEGKFYGPFVDRQTAQAAGTDTTITL      3442
AP006560.1      GSCGSVGFNIDYDCVSFCYMHHMELPTGVHAGTDLEGKFYGPFVDRQTAQAAGTDTTITL      3442
AP006557.1      GSCGSVGFNIDYDCVSFCYMHHMELPTGVHAGTDLEGKFYGPFVDRQTAQAAGTDTTITL      3442
AY274119        GSCGSVGFNIDYDCVSFCYMHHMELPTGVHAGTDLEGKFYGPFVDRQTAQAAGTDTTITL      3442
AY572038.1      GSCGSVGFNIDYDCVSFCYMHHMELPTGVHAGTDLEGKFYGPFVDRQTAQAAGTDTTITL      3442
AY572034.1      GSCGSVGFNIDYDCVSFCYMHHMELPTGVHAGTDLEGKFYGPFVDRQTAQAAGTDTTITL      3442
FJ588686.1      GSCGSVGFNVDYDCVSFCYMHHMELPTGVHAGTDLEGKFYGPFVDRQTAQAAGTDTTITL      3249
KY417145.1      GSCGSVGFNIDYDCVSFCYMHHMELPTGVHAGTDLEGKFYGPFVDRQTAQAAGTDTTITL      3442
KY417144.1      GSCGSVGFNIDYDCVSFCYMHHMELPTGVHAGTDLEGKFYGPFVDRQTAQAAGTDTTITL      3442
KY417147.1      GSCGSVGFNIDYDCVSFCYMHHMELPTGVHAGTDLEGKFYGPFVDRQTAQAAGTDTTITL      3442
KY417148.1      GSCGSVGFNIDYDCVSFCYMHHMELPTGVHAGTDLEGKFYGPFVDRQTAQAAGTDTTITL      3442
KY417143.1      GSCGSVGFNIDYDCVSFCYMHHMELPTGVHAGTDLEGKFYGPFVDRQTAQAAGTDTTITL      3442
KT444582.1      GSCGSVGFNIDYDCVSFCYMHHMELPTGVHAGTDLEGKFYGPFVDRQTAQAAGTDTTITL      3442
KC881005.1      GSCGSVGFNIDYDCVSFCYMHHMELPTGVHAGTDLEGKFYGPFVDRQTAQAAGTDTAITL      3442
KC881006.1      GSCGSVGFNIDYDCVSFCYMHHMELPTGVHAGTDLEGKFYGPFVDRQTAQAAGTDTTITL      3442
KF367457.1      GSCGSVGFNIDYDCVSFCYMHHMELPTGVHAGTDLEGKFYGPFIDRQTAQAAGTDTTITL      3442
KY417152.1      GSCGSVGFNIDYDCVSFCYMHHMELPTGVHAGTDLEGKFYGPFVDRQTAQAAGTDTTITL      3442
KY417146.1      GSCGSVGFNIDYDCVSFCYMHHMELPTGVHAGTDLEGKFYGPFVDRQTAQAAGTDTTITL      3442
KY417151.1      GSCGSVGFNIDYDCVSFCYMHHMELPTGVHAGTDLEGKFYGPFVDRQTAQAAGTDTTITL      3442
KY417142.1      GSCGSVGFNIDYDCVSFCYMHHMELPTGVHAGTDLEGKFYGPFVDRQTAQAAGTDTTITL      3442
                *********:**************************.*****:*************.**:
```

```
MW532698.1       NVLAWLYAAVINGDRWFLNRYTTTLNDFNLVAMKFNYEPLTQDHVDILGPLSAQTGIAVL         3517
MT040336.1       NVLAWLYAAVINGDRWFLNRYTTTLNDFNLVAMKFNYEPLTQDHVDILGPLSAQTGIAVL         3517
MT040335.1       NVLAWLYAAVINGDRWFLNRYTTTLNDFNLVAMKFNYEPLTQDHVDILGPLSAQTGIAVL         3517
MT040334.1       NVLAWLYAAVINGDRWFLNRYTTTLNDFNLVAMKFNYEPLTQDHVDILGPLSAQTGVAVL         3517
MT040333.1       NVLAWLYAAVINGDRWFLNRYTTTLNDFNLVAMKFNYEPLTQDHVDILGPLSAQTGIAVL         3517
MN996532.2       NVLAWLYAAVINGDRWFLNRFTTTLNDFNLVAMKYNYEPLTQDHVDILGPLSAQTGIAVL         3524
MN988713.1       NVLAWLYAAVINGDRWFLNRFTTTLNDFNLVAMKYNYEPLTQDHVDILGPLSAQTGIAVL         3525
MT093571.1       NVLAWLYAAVINGDRWFLNRFTTTLNDFNLVAMKYNYEPLTQDHVDILGPLSAQTGIAVL         3525
MN996529.1       NVLAWLYAAVINGDRWFLNRFTTTLNDFNLVAMKYNYEPLTQDHVDILGPLSAQTGIAVL         3525
MT072688.1       NVLAWLYAAVINGDRWFLNRFTTTLNDFNLVAMKYNYEPLTQDHVDILGPLSAQTGIAVL         3525
NC_045512.2      NVLAWLYAAVINGDRWFLNRFTTTLNDFNLVAMKYNYEPLTQDHVDILGPLSAQTGIAVL         3525
MN994467.1       NVLAWLYAAVINGDRWFLNRFTTTLNDFNLVAMKYNYEPLTQDHVDILGPLSAQTGIAVL         3525
MG772933.1       NVLAWLYAAVINGDRWFLNRFTTTLNDFNLVAMKYNYEPLTQDHLDILGPLSAQTGIAVL         3521
MG772934.1       NVLAWLYAAVINGDRWFLNRFTTTLNDFNLVAMKYNYEPLTQDHLDILGPLSAQTGIAVL         3499
NC_014470.1      NVLAWLYAAVINGERWFLNRFTTTLNDFNLVAMKYNYEPLTQDQVDILGPLSAQTGVAVM         3493
GQ153542.1       NVLAWLYAAVINGDRWFLNRFTTTLNDFNLVAMKYNYEPLTQDHVDILGPLSAQTGIAVL         3500
DQ022305         NVLAWLYAAVINGDRWFLNRFTTTLNDFNLVAMKYNYEPLTQDHVDILGPLSAQTGIAVL         3496
KF569996.1       NVLAWLYAAVINGDRWFLNRFTTTLNDFNLVAMKYNYEPLTQDHVDILGPLSAQTGIAVL         3502
KP886809.1       NVLAWLYAAVINGDRWFLNRFTTTLNDFNLVAMKYNYEPLTQDHVDVLGPLSAQTGIAVL         3502
AY278488.2       NVLAWLYAAVINGDRWFLNRFTTTLNDFNLVAMKYNYEPLTQDHVDILGPLSAQTGIAVL         3502
AY485277.1       NVLAWLYAAVINGDRWFLNRFTTTLNDFNLVAMKYNYEPLTQDHVDILGPLSAQTGIAVL         3502
AP006560.1       NVLAWLYAAVINGDRWFLNRFTTTLNDFNLVAMKYNYEPLTQDHVDILGPLSAQTGIAVL         3502
AP006557.1       NVLAWLYAAVINGDRWFLNRFTTTLNDFNLVAMKYNYEPLTQDHVDILGPLSAQTGIAVL         3502
AY274119         NVLAWLYAAVINGDRWFLNRFTTTLNDFNLVAMKYNYEPLTQDHVDILGPLSAQTGIAVL         3502
AY572038.1       NVLAWLYAAVINGDRWFLNRFTTTLNDFNLVAMKYNYEPLTQDHVDILGPLSAQTGIAVL         3502
AY572034.1       NVLAWLYAAVINGDRWFLNRFTTTLNDFNLVAMKYNYEPLTQDHVDILGPLSAQTGIAVL         3502
FJ588686.1       NVLAWLYAAVINGDRWFLNRFTTTLNDFNLVAMKYNYEPLTQDHVDILGPLSAQTGIAVL         3309
KY417145.1       NVLAWLYAAVINGDRWFLNRFTTTLNDFNLVAMKYNYEPLTQDHVDILGPLSAQTGIAVL         3502
KY417144.1       NVLAWLYAAVINGDRWFLNRFTTTLNDFNLVAMKYNYEPLTQDHVDILGPLSAQTGIAVL         3502
KY417147.1       NVLAWLYAAVINGDRWFLNRFTTTLNDFNLVAMKYNYEPLTQDHVDILGPLSAQTGIAVL         3502
KY417148.1       NVLAWLYAAVINGDRWFLNRFTTTLNDFNLVAMKYNYEPLTQDHVDILGPLSAQTGIAVL         3502
KY417143.1       NVLAWLYAAVINGDRWFLNRFTTTLNDFNLVAMKYNYEPLTQDHVDILGPLSAQTGIAVL         3502
KT444582.1       NVLAWLYAAVINGDRWFLNRFTTTLNDFNLVAMKYNYEPLTQDHVDILGPLSAQTGIAVL         3502
KC881005.1       NVLAWLYAAVINGDRWFLNRFTTTLNDFNLVAMKYNYEPLTQDHVDILGPLSAQTGIAVL         3502
KC881006.1       NVLAWLYAAVINGDRWFLNRFTTTLNDFNLVAMKYNYEPLTQDHVDILGPLSAQTGIAVL         3502
KF367457.1       NVLAWLYAAVINGDRWFLNRFTTTLNDFNLVAMKYNYEPLTQDHVDILGPLSAQTGIAVL         3502
KY417152.1       NVLAWLYAAVINGDRWFLNRFTTTLNDFNLVAMKYNYEPLTQDHVDILGPLSAQTGIAVL         3502
KY417146.1       NVLAWLYAAVINGDRWFLNRFTTTLNDFNLVAMKYNYEPLTQDHVDILGPLSAQTGIAVL         3502
KY417151.1       NVLAWLYAAVINGDRWFLNRFTTTLNDFNLVAMKYNYEPLTQDHVDILGPLSAQTGIAVL         3502
KY417142.1       NVLAWLYAAVINGDRWFLNRFTTTLNDFNLVAMKYNYEPLTQDHVDILGPLSAQTGIAVL         3502
                 *************:******:*************:********::*:*********:**:
```

```
MW532698.1      DMCASLKELLQNGVNGRTILGSAILEDEFTPFDVVRQCSGVTFQSAIKRTVKGTHHWLLL     3577
MT040336.1      DMCASLKELLQNGMNGRTILGSAILEDEFTPFDVVRQCSGVTFQSAIKRTVKGTHHWLLL     3577
MT040335.1      DMCASLKELLQNGMNGRTILGSAILEDEFTPFDVVRQCSGVTFQSAIKRTVKGTHHWLLL     3577
MT040334.1      DMCASLKELLQNGMNGRTILGSAILEDEFTPFDVVRQCSGVTFQSAIKRTVKGTHHWLLL     3577
MT040333.1      DMCASLKELLQNGMNGRTILGSAILEDEFTPFDVVRQCSGVTFQSAIKRTVKGTHHWLLL     3577
MN996532.2      DMCASLKELLQNGMNGRTILGSALLEDEFTPFDVVRQCSGVTFQSAVKRTIKGTHHWLLL     3584
MN988713.1      DMCASLKELLQNGMNGRTILGSALLEDEFTPFDVVRQCSGVTFQSAVKRTIKGTHHWLLL     3585
MT093571.1      DMCASLKELLQNGMNGRTILGSALLEDEFTPFDVVRQCSGVTFQSAVKRTIKGTHHWLLL     3585
MN996529.1      DMCASLKELLQNGMNGRTILGSALLEDEFTPFDVVRQCSGVTFQSAVKRTIKGTHHWLLL     3585
MT072688.1      DMCASLKELLQNGMNGRTILGSALLEDEFTPFDVVRQCSGVTFQSAVKRTIKGTHHWLLL     3585
NC_045512.2     DMCASLKELLQNGMNGRTILGSALLEDEFTPFDVVRQCSGVTFQSAVKRTIKGTHHWLLL     3585
MN994467.1      DMCASLKELLQNGMNGRTILGSALLEDEFTPFDVVRQCSGVTFQSAVKRTIKGTHHWLLL     3585
MG772933.1      DMCASLKELLQNGMNGRTILGSALLEDEFTPFDVVRQCSGVTFQSAVKRTIKGTHHWLLL     3581
MG772934.1      DMCASLKELLQNGMNGRTILGSALLEDEFTPFDVVRQCSGVTFQSAVKRTIKGTHHWLLL     3559
NC_014470.1     DMCAALKELLQNGLNGRTILGSTILEDEFTPFDVVRQCSGVTFQGKFKKVVKGTHHWLLL     3553
GQ153542.1      DMCAALKELLQNGMNGRTILGSTILEDEFTPFDVVRQCSGVTFQGKFKKIVKGTHHWMLL     3560
DQ022305        DMCAALKELLQNGMNGRTILGSTILEDEFTPFDVVRQCSGVTFQGKFKKIVKGTHHWMLL     3556
KF569996.1      DMCAALKELLQNGMNGRTILGSTILEDEFTPFDVVRQCSGVTFQGKFKKIVKGTHHWMLL     3562
KP886809.1      DMCAALKELLQNGMNGRTILGSTILEDEFTPFDVVRQCSGVTFQGKFKKIVKGTHHWMLL     3562
AY278488.2      DMCAALKELLQNGMNGRTILGSTILEDEFTPFDVVRQCSGVTFQGKFKKIVKGTHHWMLL     3562
AY485277.1      DMCAALKELLQNGMNGRTILGSTILEDEFTPFDVVRQCSGVTFQGKFKKIVKGTHHWMLL     3562
AP006560.1      DMCAALKELLQNGMNGRTILGSTILEDEFTPFDVVRQCSGVTFQGKFKKIVKGTHHWMLL     3562
AP006557.1      DMCAALKELLQNGMNGRTILGSTILEDEFTPFDVVRQCSGVTFQGKFKKIVKGTHHWMLL     3562
AY274119        DMCAALKELLQNGMNGRTILGSTILEDEFTPFDVVRQCSGVTFQGKFKKIVKGTHHWMLL     3562
AY572038.1      DMCAALKELLQNGMNGRTILGSTILEDEFTPFDVVRQCSGVTFQGKFKKIVKGTHHWMLL     3562
AY572034.1      DMCAALKELLQNGMNGRTILGSTILEDEFTPFDVVRQCSGVTFQGKFKKIVKGTHHWMLL     3562
FJ588686.1      DMCAALKELLQNGMNGRTILGSTILEDEFTPFDVVRQCSGVTFQGKFKKIVKGTHHWMLL     3369
KY417145.1      DMCAALKELLQNGMNGRTILGSTILEDEFTPFDVVRQCSGVTFQGKFKKIVKGTHHWMLL     3562
KY417144.1      DMCAALKELLQNGMNGRTILGSTILEDEFTPFDVVRQCSGVTFQGKFKKIVKGTHHWMLL     3562
KY417147.1      DMCAALKELLQNGMNGRTILGSTILEDEFTPFDVVRQCSGVTFQGKFKKIVKGTHHWLLL     3562
KY417148.1      DMCAALKELLQNGMNGRTILGSTILEDEFTPFDVVRQCSGVTFQGKFKKIVKGTHHWLLL     3562
KY417143.1      DMCAALKELLQNGMNGRTILGSTILEDEFTPFDVVRQCSGVTFQGKFKKIVKGTHHWLLL     3562
KT444582.1      DMCAALKELLQNGMNGRTILGSTILEDEFTPFDVVRQCSGVTFQGTFKKIVKGTHHWLLL     3562
KC881005.1      DMCAALKELLQNGMNGRTILGSTILEDEFTPFDVVRQCSGVTFQGKFKKIVKGTHHWMLL     3562
KC881006.1      DMCAALKELLQNGMNGRTILGSTILEDEFTPFDVVRQCSGVTFQGKFKKIVKGTHHWMLL     3562
KF367457.1      DMCAALKELLQNGMNGRTILGSTILEDEFTPFDVVRQCSGVTFQGKFKKIVKGTHHWMLL     3562
KY417152.1      DMCAVLKELLQNGMNGRTILGSTILEDEFTPFDVVRQCSGVTFQGKFKKIVKGTHHWMLL     3562
KY417146.1      DMCAALKELLQNGMNGRTILGSTILEDEFTPFDVVRQCSGVTFQGKFKKIVKGTHHWMLL     3562
KY417151.1      DMCAALKELLQNGMNGRTILGSTILEDEFTPFDVVRQCSGVTFQGKFKKIVKGTHHWMLL     3562
KY417142.1      DMCAALKDLLQNGMNGRTILGSTILEDEFTPFDVVRQCSGVTFQGKFKKIVKGTHHWMLL     3562
                **** **:*****:********::********************. .*: :******:**
```

```
MW532698.1      TILTSLLVLVQSTQWSLFFFVYENAFLPFALGIIAMSAFAMMFVKHKHAFLCLFLLPSLA   3637
MT040336.1      TILTSLLVLVQSTQWSLFFFVYENAFLPFALGIIAMSAFAMMFVKHKHAFLCLFLLPSLA   3637
MT040335.1      TILTSLLVLVQSTQWSLFFFVYENAFLPFALGIIAMSAFAMMFVKHKHAFLCLFLLPSLA   3637
MT040334.1      TILTSLLVLVQSTQWSLFFFVYENAFLPFALGIIAMSAFAMMFVKHKHAFLCLFLLPSLA   3637
MT040333.1      TILTSLLVLVQSTQWSLFFFVYENAFLPFALGIIAMSAFAMMFVKHKHAFLCLFLLPSLA   3637
MN996532.2      TILTSLLVLVQSTQWSLFFFVYENAFLPFAMGIIAMSAFAMMFVKHKHAFLCLFLLPSLA   3644
MN988713.1      TILTSLLVLVQSTQWSLFFFLYENAFLPFAMGIIAMSAFAMMFVKHKHAFLCLFLLPSLA   3645
MT093571.1      TILTSLLVLVQSTQWSLFFFLYENAFLPFAMGIIAMSAFAMMFVKHKHAFLCLFLLPSLA   3645
MN996529.1      TILTSLLVLVQSTQWSLFFFLYENAFLPFAMGIIAMSAFAMMFVKHKHAFLCLFLLPSLA   3645
MT072688.1      TILTSLLVLVQSTQWSLFFFLYENAFLPFAMGIIAMSAFAMMFVKHKHAFLCLFLLPSLA   3645
NC_045512.2     TILTSLLVLVQSTQWSLFFFLYENAFLPFAMGIIAMSAFAMMFVKHKHAFLCLFLLPSLA   3645
MN994467.1      TILTSLLVLVQSTQWSLFFFLYENAFLPFAMGIIAMSAFAMMFVKHKHAFLCLFLLPSLA   3645
MG772933.1      TVLTSLLVLVQSTQWSLFFFVYENAFMPFAMGIIAMSAFAMMFVKHKHAFLCLFLLPSLA   3641
MG772934.1      TVLTSLLVLVQSTQWSLFFFVYENAFMPFAMGIIAMSAFAMMFVKHKHAFLCLFLLPSLA   3619
NC_014470.1     TLLTSLLILVQSTQWSLFFFVYEHAFLPFTMGVVCFAACAMVLVKHKHAFLCLFLLPSLI   3613
GQ153542.1      TFLTSLLILVQSTQWSLFFFVYENAFLPFALGIMAVAACAMLLVKHKHAFLCLFLLPSLA   3620
DQ022305        TFLTSLLILVQSTQWSLFFFVYENAFLPFALGIMAVAACAMLLVKHKHAFLCLFLLPSLA   3616
KF569996.1      TFLTSLLILVQSTQWSLFFFVYENAFLPFTFGIMAIAACAMLLVKHKHAFLCLFLLPSLA   3622
KP886809.1      TFLMSLLILVQSTQWSLFFFVYENAFLPFTLGIMAIAACAMLLVKHKHAFLCLFLLPSLA   3622
AY278488.2      TFLTSLLILVQSTQWSLFFFVYENAFLPFTLGIMAIAACAMLLVKHKHAFLCLFLLPSLA   3622
AY485277.1      TFLTSLLILVQSTQWSLFFFVYENAFLPFTLGIMAIAACAMLLVKHKHAFLCLFLLPSLA   3622
AP006560.1      TFLTSLLILVQSTQWSLFFFVYENAFLPFTLGIMAIAACAMLLVKHKHAFLCLFLLPSLA   3622
AP006557.1      TFLTSLLILVQSTQWSLFFFVYENAFLPFTLGIMAIAACAMLLVKHKHAFLCLFLLPSLA   3622
AY274119        TFLTSLLILVQSTQWSLFFFVYENAFLPFTLGIMAIAACAMLLVKHKHAFLCLFLLPSLA   3622
AY572038.1      TFLTSLLILVQSTQWSLFFFVYENAFLPFTLGIMAIAACAMLLVKHKHAFLCLFLLPSLA   3622
AY572034.1      TFLTSLLILVQSTQWSLFFFVYENAFLPFTLGIMAIAACAMLLVKHKHAFLCLFLLPSLA   3622
FJ588686.1      TFLTSLLILVQSTQWSLFFFVYENAFLPFTLGIMAIAACAMLLVKHKHAFLCLFLLPSLA   3429
KY417145.1      TFLTSLLILVQSTQWSLFFFVYENAFLPFTLGIMAIAACAMLLVKHKHAFLCLFLLPSLA   3622
KY417144.1      TFLTSLLILVQSTQWSLFFFVYENAFLPFTLGIMAIAACAMLLVKHKHAFLCLFLLPSLA   3622
KY417147.1      TFLTSLLILVQSTQWSLFFFVYENAFLPFTLGIMAIAACAMLLVKHKHAFLCLFLLPSLA   3622
KY417148.1      TFLTSLLILVQSTQWSLFFFVYENAFLPFTLGIMAIAACAMLLVKHKHAFLCLFLLPSLA   3622
KY417143.1      TFLTSLLILVQSTQWSLFFFVYENAFLPFTLGIMAIAACAMLLVKHKHAFLCLFLLPSLA   3622
KT444582.1      TFLTSLLILVQSTQWSLFFFVYENAFLPFTLGIMAIAACAMLLVKHKHAFLCLFLLPSLA   3622
KC881005.1      TFLTSLLILVQSTQWSLFFFVYENAFLPFTLGIMAIAACAMLLVKHKHAFLCLFLLPSLA   3622
KC881006.1      TFLTSLLILVQSTQWSLFFFVYENAFLPFTLGIMAIAACAMLLVKHKHAFLCLFLLPSLA   3622
KF367457.1      TFLTSLLILVQSTQWSLFFFVYENAFLPFTLGIMAIAACAMLLVKHKHAFLCLFLLPSLA   3622
KY417152.1      TFLTSLLILVQSTQWSLFFFVYENAFLPFTLGIMAIAACAMLLVKHKHAFLCLFLLPSLA   3622
KY417146.1      TFLTSLLILVQSTQWSLFFFVYENAFLPFTLGIMAIAACAMLLVKHKHAFLCLFLLPSLA   3622
KY417151.1      TFLTSLLILVQSTQWSLFFFVYENAFLPFTLGIMAIAACAMLLVKHKHAFLCLFLLPSLA   3622
KY417142.1      TFLTSLLILVQSTQWSLFFFVYENAFLPFTLGIMAIAACAMLLVKHKHAFLCLFLLPSLA   3622
                *.* ***:************:**:**:**::*::..:* **::****************
```

```
MW532698.1      TVAYFNMVYMPASWVMRIMTWLDMVDTSLSGYKLKDCIMYASAIILLILMTARTVYDDGA    3697
MT040336.1      TVAYFNMVYMPASWVMRIMTWLDMVDTSLSGYKLKDCIMYASAIILLILMTARTVYDDGA    3697
MT040335.1      TVAYFNMVYMPASWVMRIMTWLDMVDTSLSGYKLKDCIMYASAIILLILMTARTVYDDGA    3697
MT040334.1      TVAYFNMVYMPASWVMRIMTWLDMVDTSLSGYKLKDCIMYASAIILLILMTARTVYDDGA    3697
MT040333.1      TVAYFNMVYMPASWVMRIMTWLDMVDTSLSGYKLKDCIMYASAIILLILMTARTVYDDGA    3697
MN996532.2      TVAYFNMVYMPASWVMRIMTWLDMVDTSLSGFKLKDCVMYASAVVLLILMTARTVYDDGA    3704
MN988713.1      TVAYFNMVYMPASWVMRIMTWLDMVDTSLSGFKLKDCVMYASAVVLLILMTARTVYDDGA    3705
MT093571.1      TVAYFNMVYMPASWVMRIMTWLDMVDTSLSGFKLKDCVMYASAVVLLILMTARTVYDDGA    3705
MN996529.1      TVAYFNMVYMPASWVMRIMTWLDMVDTSLSGFKLKDCVMYASAVVLLILMTARTVYDDGA    3705
MT072688.1      TVAYFNMVYMPASWVMRIMTWLDMVDTSLSGFKLKDCVMYASAVVLLILMTARTVYDDGA    3705
NC_045512.2     TVAYFNMVYMPASWVMRIMTWLDMVDTSLSGFKLKDCVMYASAVVLLILMTARTVYDDGA    3705
MN994467.1      TVAYFNMVYMPASWVMRIMTWLDMVDTSLSGFKLKDCVMYASAVVLLILMTARTVYDDGA    3705
MG772933.1      TVAYFNMVYMPASWVMRIMTWLDIVDTSLSGFKLKDCVMYASAVVLLILMTARTVYDDGA    3701
MG772934.1      TVAYFNMVYMPASWVMRIMTWLDIVDTSLSGFKLKDCVMYASAVVLLILMTARTVYDDSA    3679
NC_014470.1     TVAYFNMIYMPASWVMRVMTWLDLVDTSLSGYRLKDCVMYALAAFLLILMTARTVYDDAA    3673
GQ153542.1      TVAYFNMVYMPASWVMRIMTWLELADTSLSGYRLKDCVMYASALVLLILMTARTVYDDAA    3680
DQ022305        TVAYFNMVYMPASWVMRIMTWLELADTSLSGYRLKDCVMYASALVLLILMTARTVYDDAA    3676
KF569996.1      TVAYFNMVYMPASWVMRIMTWLELADTSLSGYRLKDCVMYASALVLLVLMTARTVYDDAA    3682
KP886809.1      TVAYFNMVYMPASWVMRIMTWLELADTSLSGYRLKDCVMYASALVLLILMTARTVYDDAA    3682
AY278488.2      TVAYFNMVYMPASWVMRIMTWLELADTSLSGYRLKDCVMYASALVLLILMTARTVYDDAA    3682
AY485277.1      TVAYFNMVYMPASWVMRIMTWLELADTSLSGYRLKDCVMYASALVLLILMTARTVYDDAA    3682
AP006560.1      TVAYFNMVYMPASWVMRIMTWLELADTSLSGYRLKDCVMYASALVLLILMTARTVYDDAA    3682
AP006557.1      TVAYFNMVYMPASWVMRIMTWLELADTSLSGYRLKDCVMYASALVLLILMTARTVYDDAA    3682
AY274119        TVAYFNMVYMPASWVMRIMTWLELADTSLSGYRLKDCVMYASALVLLILMTARTVYDDAA    3682
AY572038.1      TVAYFNMVYMPASWVMRIMTWLELADTSLSGYRLKDCVMYASALVLLILMTARTVYDDAA    3682
AY572034.1      TVAYFNMVYMPASWVMRIMTWLELADTSLSGYRLKDCVMYASALVLLILMTARTVYDDAA    3682
FJ588686.1      TVAYFNMVYMPASWVMRIMTWLELADTSLSGYRLKDCVMYASALVLLILMTARTVYDDAA    3489
KY417145.1      TVAYFNMVYMPASWVMRIMTWLELADTSLSGYRLKDCVMYASALVLLILMTARTVYDDAA    3682
KY417144.1      TVAYFNMVYMPASWVMRIMTWLELADTSLSGYRLKDCVMYASALVLLILMTARTVYDDAA    3682
KY417147.1      TVAYFNMVYMPASWVMRIMTWLELADTSLSGYRLKDCVMYASALVLLILMTARTVYDDAA    3682
KY417148.1      TVAYFNMVYMPASWVMRIMTWLELADTSLSGYRLKDCVMYASALVLLILMTARTVYDDAA    3682
KY417143.1      TVAYFNMVYMPASWVMRIMTWLELADTSLSGYRLKDCVMYASALVLLILMTARTVYDDAA    3682
KT444582.1      TVAYFNMVYMPASWVMRIMTWLELADTSLSGYRLKDCVMYASALVLLILMTARTVYDDAA    3682
KC881005.1      TVAYFNMVYMPASWVMRIMTWLELADTSLSGYRLKDCVMYASALVLLILMTARTVYDDAA    3682
KC881006.1      TVAYFNMVYMPASWVMRIMTWFELADTSLSGYRLKDCVMYASALVLLILMTARTVYDDAA    3682
KF367457.1      TVAYFNMVYMPASWVMRIMTWFELADTSLSGYRLKDCVMYASALVLLILMTARTVYDDAA    3682
KY417152.1      TVAYFNMVYMPASWVMRIMTWLELADTSLSGYRLKDCVMYASALVLLILMTARTVYDDAA    3682
KY417146.1      TVAYFNMVYMPASWVMRIMTWLELADTSLSGYRLKDCVMYASALVLLILMTARTVYDDAA    3682
KY417151.1      TVAYFNMVYMPASWVMRIMTWLELADTSLSGYRLKDCVMYASALVLLILMTARTVYDDAA    3682
KY417142.1      TVAYFNMVYMPASWVMRIMTWLELADTSLSGYRLKDCVMYASALVLLILMTARTVYDDAA    3682
                *******:*********:***:::.******::****:*** * .**:**********.*
```

```
MW532698.1      RRVWTLMNVLTLVYKVYYGNALDQAISMWALIISVTSNYSGVVTTVMFLARGIVFMCVEY    3757
MT040336.1      RRVWTLMNVLTLVYKVYYGNALDQAISMWALIISVTSNYSGVVTTVMFLARGIVFMCVEY    3757
MT040335.1      RRVWTLMNVLTLVYKVYYGNALDQAISMWALIISVTSNYSGVVTAVMFLARGIVFMCVEY    3757
MT040334.1      RRVWTLMNVLTLVYKVYYGNALDQAISMWALIISVTSNYSGVVTTVMFLARGIVFMCVEY    3757
MT040333.1      RRVWTLMNVLTLVYKVYYGNALDQAISMWALIISVTSNYSGVVTTVMFLARGIVFMCVEY    3757
MN996532.2      RRVWTLMNVLTLVYKVYYGNALDQAISMWALIISVTSNYSGVVTTVMFLARGIVFMCVEY    3764
MN988713.1      RRVWTLMNVLTLVYKVYYGNALDQAISMWALIISVTSNYSGVVTTVMFLARGIVFMCVEY    3765
MT093571.1      RRVWTLMNVLTLVYKVYYGNALDQAISMWALIISVTSNYSGVVTTVMFLARGIVFMCVEY    3765
MN996529.1      RRVWTLMNVLTLVYKVYYGNALDQAISMWALIISVTSNYSGVVTTVMFLARGIVFMCVEY    3765
MT072688.1      RRVWTLMNVLTLVYKVYYGNALDQAISMWALIISVTSNYSGVVTTVMFLARGIVFMCVEY    3765
NC_045512.2     RRVWTLMNVLTLVYKVYYGNALDQAISMWALIISVTSNYSGVVTTVMFLARGIVFMCVEY    3765
MN994467.1      RRVWTLMNVLTLVYKVYYGNALDQAISMWALIISVTSNYSGVVTTVMFLARGIVFMCVEY    3765
MG772933.1      RRVWTLMNVLTLVYKVYYGNALDQAISMWALIISVTSNYSGVVTTVMFLARGIVFMCVEY    3761
MG772934.1      RRVWTLMNVLTLVYKVYYGNALDQAISMWALIISVTSNYSGVVTTVMFLARGIVFMCVEY    3739
NC_014470.1     RRVWTVMNVITLVYKVYYGNSLDQALAMWALVISVTSNYSGVVTTIMFLARAIVFLCVEY    3733
GQ153542.1      RRVWTLMNVITLVYKVYYGNSLDQAISMWALVISVTSNYSGVVTTIMFLARAIVFVCVEY    3740
DQ022305        RRVWTLMNVITLVYKVYYGNSLDQAISMWALVISVTSNYSGVVTTIMFLARAIVFVCVEY    3736
KF569996.1      RRVWTLMNVITLVYKVYYGNALDQAISMWALVISVTSNYSGVVTTIMFLARAIVFVCVEY    3742
KP886809.1      RRVWTLMNVITLVYKVYYGNALDQAISMWALVISVTSNYSGVVTTIMFLARAIVFVCVEY    3742
AY278488.2      RRVWTLMNVITLVYKVYYGNALDQAISMWALVISVTSNYSGVVTTIMFLARAIVFVCVEY    3742
AY485277.1      RRVWTLMNVITLVYKVYYGNALDQAISMWALVISVTSNYSGVVTTIMFLARAIVFVCVEY    3742
AP006560.1      RRVWTLMNVITLVYKVYYGNALDQAISMWALVISVTSNYSGVVTTIMFLARAIVFVCVEY    3742
AP006557.1      RRVWTLMNVITLVYKVYYGNALDQAISMWALVISVTSNYSGVVTTIMFLARAIVFVCVEY    3742
AY274119        RRVWTLMNVITLVYKVYYGNALDQAISMWALVISVTSNYSGVVTTIMFLARAIVFVCVEY    3742
AY572038.1      RRVWTLMNVITLVYKVYYGNALDQAISMWALVISVTSNYSGVVTTIMFLARAIVFVCVEY    3742
AY572034.1      RRVWTLMNVITLVYKVYYGNALDQAISMWALVISVTSNYSGVVTTIMFLARAIVFVCVEY    3742
FJ588686.1      RRVWTLMNVITLVYKVYYGNALDQAISMWALVISVTSNYSGVVTTIMFLARAIVFVCVEY    3549
KY417145.1      RRVWTLMNVITLVYKVYYGNALDQAISMWALVISVTSNYSGVVTTIMFLARAIVFVCVEY    3742
KY417144.1      RRVWTLMNVITLVYKVYYGNALDQAISMWALVISVTSNYSGVVTTIMFLARAIVFVCVEY    3742
KY417147.1      RRVWTLMNVITLVYKVYYGNALDQAISMWALVISVTSNYSGVVTTIMFLARAIVFVCVEY    3742
KY417148.1      RRVWTLMNVITLVYKVYYGNALDQAISMWALVISVTSNYSGVVTTIMFLARAIVFVCVEY    3742
KY417143.1      RRVWTLMNVITLVYKVYYGNALDQAISMWALVISVTSNYSGVVTTIMFLARAIVFVCVEY    3742
KT444582.1      RRVWTLMNVITLVYKVYYGNALDQAISMWALVISVTSNYSGVVTTIMFLARAIVFVCVEY    3742
KC881005.1      RRVWTLMNVITLVYKVYYGNALDQAISMWALVISVTSNYSGVVTTIMFLARAIVFVCVEY    3742
KC881006.1      RRVWTLMNVITLVYKVYYGNALDQAISMWALVISVTSNYSGVVTTIMFLARAIVFVCVEY    3742
KF367457.1      RRVWTLMNVITLVYKVYYGNALDQAISMWALVISVTSNYSGVVTTIMFLARAIVFVCVEY    3742
KY417152.1      RRVWTLMNVITLVYKVYYGNALDQAISMWALVISVTSNYSGVVTTIMFLARAIVFVCVEY    3742
KY417146.1      RRVWTLMNVITLVYKVYYGNALDQAISMWALVISVTSNYSGVVTTIMFLARAIVFVCVEY    3742
KY417151.1      RRVWTLMNVITLVYKVYYGNALDQAISMWALVISVTSNYSGVVTTIMFLARAIVFVCVEY    3742
KY417142.1      RRVWTLMNVITLVYKVYYGNALDQAISMWALVISVTSNYSGVVTTIMFLARAIVFVCVEY    3742
                *****:***:**********:****::****:************::*****.***:****
```

```
MW532698.1       CPILFITGNTLQCIMLVYCFLGYFCTCYFGLFCLLNRYFRLTLGVYDYLVSTQEFRYMNS   3817
MT040336.1       CPILFITGNTLQCIMLVYCFLGYFCTCYFGLFCLLNRYFRLTLGVYDYLVSTQEFRYMNS   3817
MT040335.1       CPILFITGNTLQCIMLVYCFLGYFCTCYFGLFCLLNRYFRLTLGVYDYLVSTQEFRYMNS   3817
MT040334.1       CPILFITGNTLQCIMLVYCFLGYFCTCYFGLFCLLNRYFRLTLGVYDYLVSTQEFRYMNS   3817
MT040333.1       CPILFITGNTLQCIMLVYCFLGYFCTCYFGLFCLLNRYFRLTLGVYDYLVSTQEFRYMNS   3817
MN996532.2       CPIFFITGNTLQCIMLVYCFLGYFCTCYFGLFCLLNRYFRLTLGVYDYLVSTQEFRYMNS   3824
MN988713.1       CPIFFITGNTLQCIMLVYCFLGYFCTCYFGLFCLLNRYFRLTLGVYDYLVSTQEFRYMNS   3825
MT093571.1       CPIFFITGNTLQCIMLVYCFLGYFCTCYFGLFCLLNRYFRLTLGVYDYLVSTQEFRYMNS   3825
MN996529.1       CPIFFITGNTLQCIMLVYCFLGYFCTCYFGLFCLLNRYFRLTLGVYDYLVSTQEFRYMNS   3825
MT072688.1       CPIFFITGNTLQCIMLVYCFLGYFCTCYFGLFCLLNRYFRLTLGVYDYLVSTQEFRYMNS   3825
NC_045512.2      CPIFFITGNTLQCIMLVYCFLGYFCTCYFGLFCLLNRYFRLTLGVYDYLVSTQEFRYMNS   3825
MN994467.1       CPIFFITGNTLQCIMLVYCFLGYFCTCYFGLFCLLNRYFRLTLGVYDYLVSTQEFRYMNS   3825
MG772933.1       CPIFFITGNTLQCIMLVYCFLGYFCTCYFGLFCLLNRYFRLTLGVYDYLVSTQEFRYMNS   3821
MG772934.1       CPIFFITGNTLQCIMLVYCFLGYFCTCYFGLFCLLNRYFRLTLGVYDYLVSTQEFRYMNS   3799
NC_014470.1      YPILFITGNTLQCIMLVYCFLGYCCCCYFGLFCLLNRYFRLTLGVYDYFVSTQEFRYMNS   3793
GQ153542.1       YPLLFITGNTLQCIMLVYCFLGYCCCCYFGLFCLLNRYFRLTLGVYDYLVSTQEFRYMNS   3800
DQ022305         YPLLFITGNTLQCIMLVYCFLGYCCCCYFGLFCLLNRYFRLTLGVYDYLVSTQEFRYMNS   3796
KF569996.1       YPLLFITGNNLQCIMLVYCFLGYCCCCYFGLFCLLNRYFRLTLGVYDYLVSTQEFRYMNS   3802
KP886809.1       YPLLFITGNTLQCIMLVYCFLGYCCCCYFGLFCLLNRYFRLTLGVYDYLVSTQEFRYMNS   3802
AY278488.2       YPLLFITGNTLQCIMLVYCFLGYCCCCYFGLFCLLNRYFRLTLGVYDYLVSTQEFRYMNS   3802
AY485277.1       YPLLFITGNTLQCIMLVYCFLGYCCCCYFGLFCLLNRYFRLTLGVYDYLVSTQEFRYMNS   3802
AP006560.1       YPLLFITGNTLQCIMLVYCFLGYCCCCYFGLFCLLNRYFRLTLGVYDYLVSTQEFRYMNS   3802
AP006557.1       YPLLFITGNTLQCIMLVYCFLGYCCCCYFGLFCLLNRYFRLTLGVYDYLVSTQEFRYMNS   3802
AY274119         YPLLFITGNTLQCIMLVYCFLGYCCCCYFGLFCLLNRYFRLTLGVYDYLVSTQEFRYMNS   3802
AY572038.1       YPLLFITGNTLQCIMLVYCFLGYCCCCYFGLFCLLNRYFRLTLGVYDYLVSTQEFRYMNS   3802
AY572034.1       YPLLFITGNTLQCIMLVYCFLGYCCCCYFGLFCLLNRYFRLTLGVYDYLVSTQEFRYMNS   3802
FJ588686.1       YPLLFITGNTLQCIMLVYCFLGYCCCCYFGLFCLLNRYFRLTLGVYDYLVSTQEFRYMNS   3609
KY417145.1       YPLLFITGNTLQCIMLVYCFLGYCCCCYFGLFCLLNRYFRLTLGVYDYLVSTQEFRYMNS   3802
KY417144.1       YPLLFITGNTLQCIMLVYCFLGYCCCCYFGLFCLLNRYFRLTLGVYDYLVSTQEFRYMNS   3802
KY417147.1       YPLLFITGNTLQCIMLVYCFLGYCCCCYFGLFCLLNRYFRLTLGVYDYLVSTQEFRYMNS   3802
KY417148.1       YPLLFITGNTLQCIMLVYCFLGYCCCCYFGLFCLLNRYFRLTLGVYDYLVSTQEFRYMNS   3802
KY417143.1       YPLLFITGNTLQCIMLVYCFLGYCCCCYFGLFCLLNRYFRLTLGVYDYLVSTQEFRYMNS   3802
KT444582.1       YPLLFITGNTLQCIMLVYCFLGYCCCCYFGLFCLLNRYFRLTLGVYDYLVSTQEFRYMNS   3802
KC881005.1       YPLLFITGNTLQCIMLVYCFLGYCCCCYFGLFCLLNRYFRLTLGVYDYLVSTQEFRYMNS   3802
KC881006.1       YPLLFITGNTLQCIMLVYCFLGYCCCCYFGLFCLLNRYFRLTLGVYDYLVSTQEFRYMNS   3802
KF367457.1       YPLLFITGNTLQCIMLVYCFLGYCCCCYFGLFCLLNRYFRLTLGVYDYLVSTQEFRYMNS   3802
KY417152.1       YPLLFITGNTLQCIMLVYCFLGYCCCCYFGLFCLLNRYFRLTLGVYDYLVSTQEFRYMNS   3802
KY417146.1       YPLLFITGNTLQCIMLVYCFLGYCCCCYFGLFCLLNRYFRLTLGVYDYLVSTQEFRYMNS   3802
KY417151.1       YPLLFITGNTLQCIMLVYCFLGYCCCCYFGLFCLLNRYFRLTLGVYDYLVSTQEFRYMNS   3802
KY417142.1       YPLLFITGNTLQCIMLVYCFLGYCCCCYFGLFCLLNRYFRLTLGVYDYLVSTQEFRYMNS   3802
                  *::*****.************* * **********************:***********
```

```
MW532698.1      QGLLPPKNSIDAFKLNVKLLGIGGKPCIKVATVQSKMSDVKCTSVVLLSVLQQLRVESSS    3877
MT040336.1      QGLLPPKNSIDAFKLNVKLLGIGGKPCIKVATVQSKMSDVKCTSVVLLSVLQQLRVESSS    3877
MT040335.1      QGLLPPKNSIDAFKLNVKPLGIGGKPCIKVATVQSKMSDVKCTSVVLLSVLQQLRVESSS    3877
MT040334.1      QGLLPPKNSIDAFKLNVKLLGIGGKPCIKVATVQSKMSDVKCTSVVLLSVLQQLRVESSS    3877
MT040333.1      QGLLPPKNSIDAFKLNVKLLGIGGKPCIKVATVQSKMSDVKCTSVVLLSVLQQLRVESSS    3877
MN996532.2      QGLLPPKNSIDAFKLNIKLLGVGGKPCIKVATVQSKMSDVKCTSVVLLSVLQQLRVESSS    3884
MN988713.1      QGLLPPKNSIDAFKLNIKLLGVGGKPCIKVATVQSKMSDVKCTSVVLLSVLQQLRVESSS    3885
MT093571.1      QGLLPPKNSIDAFKLNIKLLGVGGKPCIKVATVQSKMSDVKCTSVVLLSVLQQLRVESSS    3885
MN996529.1      QGLLPPKNSIDAFKLNIKLLGVGGKPCIKVATVQSKMSDVKCTSVVLLSVLQQLRVESSS    3885
MT072688.1      QGLLPPKNSIDAFKLNIKLLGVGGKPCIKVATVQSKMSDVKCTSVVLLSVLQQLRVESSS    3885
NC_045512.2     QGLLPPKNSIDAFKLNIKLLGVGGKPCIKVATVQSKMSDVKCTSVVLLSVLQQLRVESSS    3885
MN994467.1      QGLLPPKNSIDAFKLNIKLLGVGGKPCIKVATVQSKMSDVKCTSVVLLSVLQQLRVESSS    3885
MG772933.1      QGLLPPKSSIDAFKLNIKLLGIGGKPCIKVATVQSKMSDVKCTSVVLLSVLQQLRVESSS    3881
MG772934.1      QGLLPPKSSIDAFKLNIKLLGIGGKPCIKVATVQSKMSDVKCTSVVLLSVLQQLRVESSS    3859
NC_014470.1     QGLLPPKTSLDAFKLNVKLLGIGGKPCIKVATVQSKMSDIKCTSVVLLSVLQQLRIESSS    3853
GQ153542.1      QGLLPPKSSIDAFKLNIKLLGIGGKPCIKVATVQSKMSDVKCTSVVLLSVLQQLRVESSS    3860
DQ022305        QGLLPPKSSIDAFKLNIKLLGIGGKPCIKVATVQSKMSDVKCTSVVLLSVLQQLRVESSS    3856
KF569996.1      QGLLPPKSSIDAFKLNIKLLGIGGKPCIKVATVQSKMSDVKCTSVVLLSVLQQLRVESSS    3862
KP886809.1      QGLLPPKSSIDAFRLNIKLLGIGGKPCIKVATVQSKMSDVKCTSVVLLSVLQQLRVESSS    3862
AY278488.2      QGLLPPKSSIDAFKLNIKLLGIGGKPCIKVATVQSKMSDVKCTSVVLLSVLQQLRVESSS    3862
AY485277.1      QGLLPPKSSIDAFKLNIKLLGIGGKPCIKVATVQSKMSDVKCTSVVLLSVLQQLRVESSS    3862
AP006560.1      QGLLPPKSSIDAFKLNIKLLGIGGKPCIKVATVQSKMSDVKCTSVVLLSVLQQLRVESSS    3862
AP006557.1      QGLLPPKSSIDAFKLNIKLLGIGGKPCIKVATVQSKMSDVKCTSVVLLSVLQQLRVESSS    3862
AY274119        QGLLPPKSSIDAFKLNIKLLGIGGKPCIKVATVQSKMSDVKCTSVVLLSVLQQLRVESSS    3862
AY572038.1      QGLLPPKSSIDAFKLNIKLLGIGGKPCIKVATVQSKMSDVRCTSVVLLSVLQQLRVESSS    3862
AY572034.1      QGLLPPKSSIDAFKLNIKLLGIGGKPCIKVATVQSKMSDVKCTSVVLLSVLQQLRVESSS    3862
FJ588686.1      QGLLPPKSSIDAFKLNIKLLGIGGKPCIKVATVQSKMSDVKCTSVVLLSVLQQLRVESSS    3669
KY417145.1      QGLLPPKSSIDAFKLNIKLLGIGGKPCIKVATVQSKMSDVKCTSVVLLSVLQQLRVESSS    3862
KY417144.1      QGLLPPKSSIDAFKLNIKLLGIGGKPCIKVATVQSKMSDVKCTSVVLLSVLQQLRVESSS    3862
KY417147.1      QGLLPPKSSIDAFKLNIKLLGIGGKPCIKVATVQSKMSDVKCTSVVLLSVLQQLRVESSS    3862
KY417148.1      QGLLPPKSSIDAFKLNIKLLGIGGKPCIKVATVQSKMSDVKCTSVVLLSVLQQLRVESSS    3862
KY417143.1      QGLLPPKSSIDAFKLNIKLLGIGGKPCIKVATVQSKMSDVKCTSVVLLSVLQQLRVESSS    3862
KT444582.1      QGLLPPKSSIDAFKLNIKLLGIGGKPCIKVATVQSKMSDVKCTSVVLLSVLQQLRVESSS    3862
KC881005.1      QGLLPPKSSIDAFKLNIKLLGIGGKPCIKVATVQSKMSDVKCTSVVLLSVLQQLRVESSS    3862
KC881006.1      QGLLPPKSSIDAFKLNIKLLGIGGKPCIKVATVQSKMSDVKCTSVVLLSVLQQLRVESSS    3862
KF367457.1      QGLLPPKSSIDAFKLNIKLLGIGGKPCIKVATVQSKMSDVKCTSVVLLSVLQQLRVESSS    3862
KY417152.1      QGLLPPKSSIDAFKLNIKLLGIGGKPCIKVATVQSKMSDVKCTSVVLLSVLQQLRVESSS    3862
KY417146.1      QGLLPPKSSIDAFKLNIKLLGIGGKPCIKVATVQSKMSDVKCTSVVLLSVLQQLRVESSS    3862
KY417151.1      QGLLPPKSSIDAFKLNIKLLGIGGKPCIKVATVQSKMSDVKCTSVVLLSVLQQLRVESSS    3862
KY417142.1      QGLLPPKSSIDAFKLNIKLLGIGGKPCIKVATVQSKMSDVKCTSVVLLSVLQQLRVESSS    3862
                *******.*:***:**:* **:*****************::**************:****
```

CoVAX_ORF1ab#6

MW532698.1	KLWAQCVQLHNDILLAKDTTEAFEKMVSLLSVLLSMQGAVDINKLCEEMLDNRATLQAIA	3937
MT040336.1	KLWAQCVQLHNDILLAKDTTEAFEKMVSLLSVLLSMQGAVDINKLCEEMLDNRATLQAIA	3937
MT040335.1	KLWAQCVQLHNDILLAKDTTEAFEKMVSLLSVLLSMQGAVDINKLCEEMLDNRATLQAIA	3937
MT040334.1	KLWAQCVQLHNDILLAKDTTEAFEKMVSLLSVLLSMQGAVDINKLCEEMLDNRATLQAIA	3937
MT040333.1	KLWAQCVQLHNDILLAKDTTEAFEKMVSLLSVLLSMQGAVDINKLCEEMLDNRATLQAIA	3937
MN996532.2	KLWAQCVQLHNDILLAKDTTEAFEKMVSLLSVLLSMQGAVDINKLCEEMLDNRATLQAIA	3944
MN988713.1	KLWAQCVQLHNDILLAKDTTEAFEKMVSLLSVLLSMQGAVDINKLCEEMLDNRATLQAIA	3945
MT093571.1	KLWAQCVQLHNDILLAKDTTEAFEKMVSLLSVLLSMQGAVDINKLCEEMLDNRATLQAIA	3945
MN996529.1	KLWAQCVQLHNDILLAKDTTEAFEKMVSLLSVLLSMQGAVDINKLCEEMLDNRATLQAIA	3945
MT072688.1	KLWAQCVQLHNDILLAKDTTEAFEKMVSLLSVLLSMQGAVDINKLCEEMLDNRATLQAIA	3945
**NC_045512.2**	**KLWAQCVQLHNDILLAKDTTEAFEKMVSLLSVLLSMQGAVDINKLCEEMLDNRATLQAIA**	**3945**
MN994467.1	KLWAQCVQLHNDILLAKDTTEAFEKMVSLLSVLLSMQGAVDINKLCEEMLDNRATLQAIA	3945
MG772933.1	KLWAQCVQLHNDILLAKDTTEAFEKMVSLLSVLLSMQGAVDINKLCEEMLDNRATLQAIA	3941
MG772934.1	KLWAQCVQLHNDILLAKDTTEAFEKMVSLLSVLLSMQGAVDLNKLCEEMLDNRATLQAIA	3919
NC_014470.1	KLWAQCVQLHNDILLAKDTTEAFEKMVSLLSVLLSMQGAVDINKLCDEMLNNRATLQAIA	3913
GQ153542.1	KLWAQCVQLHNDILLAKDTTEAFEKMVSLLSVLLSMQGAVDINKLCEEMLDNRATLQAIA	3920
DQ022305	KLWAQCVQLHNDILLAKDTTEAFEKMVSLLSVLLSMQGAVDINKLCEEMLDNRATLQAIA	3916
KF569996.1	KLWAQCVQLHNDILLAKDTTEAFEKMVSLLSVLLSMQGAVDINKLCEEMLDNRATLQAIA	3922
KP886809.1	KLWAQCVQLHNDILLAKDTTAAFEKMVSLLSVLLSMQGAVDISKLCEEMLDNRATLQAIA	3922
AY278488.2	KLWAQCVQLHNDILLAKDTTEAFEKMVSLLSVLLSMQGAVDINRLCEEMLDNRATLQAIA	3922
AY485277.1	KLWAQCVQLHNDILLAKDTTEAFEKMVSLLSVLLSMQGAVDINRLCEEMLDNRATLQAIA	3922
AP006560.1	KLWAQCVQLHNDILLAKDTTEAFEKMVSLLSVLLSMQGAVDINRLCEEMLDNRATLQAIA	3922
AP006557.1	KLWAQCVQLHNDILLAKDTTEAFEKMVSLLSVLLSMQGAVDINRLCEEMLDNRATLQAIA	3922
AY274119	KLWAQCVQLHNDILLAKDTTEAFEKMVSLLSVLLSMQGAVDINRLCEEMLDNRATLQAIA	3922
AY572038.1	KLWAQCVQLHNDILLAKDTTEAFEKMVSLLSVLLSMQGAVDINRLCEEMLDNRATLQAIA	3922
AY572034.1	KLWAQCVQLHNDILLAKDTTEAFEKMVSLLSVLLSMQGAVDINRLCEEMLDNRATLQAIA	3922
FJ588686.1	KLWAQCVQLHNDILLAKDTTEAFEKMVSLLSVLLSMQGAVDINKLCEEMLDNRATLQAIA	3729
KY417145.1	KLWAQCVQLHNDILLAKDTTEAFEKMVSLLSVLLSMQGAIDINKLCEEMLDNRATLQAIA	3922
KY417144.1	KLWAQCVQLHNDILLAKDTTEAFEKMVSLLSVLLSMQGAVDINKLCEEMLDNRATLQAIA	3922
KY417147.1	KLWAQCVQLHNDILLAKDTTEAFEKMVSLLSVLLSMQGAVDISKLCEEMLDNRATLQAIA	3922
KY417148.1	KLWAQCVQLHNDILLAKDTTEAFEKMVSLLSVLLSMQGAVDISKLCEEMLDNRATLQAIA	3922
KY417143.1	KLWAQCVQLHNDILLAKDTTEAFEKMVSLLSVLLSMQGAVDINKLCEEMLDNRATLQAIA	3922
KT444582.1	KLWAQCVQLHNDILLAKDTTEAFEKMVSLLSVLLSMQGAVDINKLCEEMLDNRATLQAIA	3922
KC881005.1	KLWAQCVQLHNDILLAKDTTEAFEKMVSLLSVLLSMQGAVDINKLCEEMLDNRATLQAIA	3922
KC881006.1	KLWAQCVQLHNDILLAKDTTEAFEKMVSLLSVLLSMQGAVDINKLCEEMLDNRATLQAIA	3922
KF367457.1	KLWAQCVQLHNDILLAKDTTEAFEKMVSLLSVLLSMQGAVDINKLCEEMLDNRATLQAIA	3922
KY417152.1	KLWAQCVQLHNDILLAKDTTEAFEKMVSLLSVLLSMQGAVDINKLCEEMLDNRATLQAIA	3922
KY417146.1	KLWAQCVQLHNDILLAKDTTEAFEKMVSLLSVLLSMQGAVDINKLCEEMLDNRATLQAIA	3922
KY417151.1	KLWAQCVQLHNDILLAKDTTEAFEKMVSLLSVLLSMQGAVDINKLCEEMLDNRATLQAIA	3922
KY417142.1	KLWAQCVQLHNDILLAKDTTEAFEKMVSLLSVLLSMQGAVDINKLCEEMLDNRATLQAIA	3922
	******************** *****************:*:..:**:***:*********	

```
MW532698.1      SEFSSLPSYAAFATAQEAYEQAVANGDSEVVLKKLKKSLNVAKSEFDRDAAMQRKLEKMA     3997
MT040336.1      SEFSSLPSYAAFATAQEAYEQAVANGDSEVVLKKLKKSLNVAKSEFDRDAAMQRKLEKMA     3997
MT040335.1      SEFSSLPSYAAFATAQEAYEQAVANGDSEVVLKKLKKSLNVAKSEFDRDAAMQRKLEKMA     3997
MT040334.1      SEFSSLPSYAAFATAQEAYEQAVANGDSEVVLKKLKKSLNVAKSEFDRDAAMQRKLEKMA     3997
MT040333.1      SEFSSLPSYAAFATAQEAYEQAVANGDSEVVLKKLKKSLNVAKSEFDRDAAMQRKLEKMA     3997
MN996532.2      SEFSSLPSYAAFATAQEAYEQAVANGDSEVVLKKLKKSLNVAKSEFDRDAAMQRKLEKMA     4004
MN988713.1      SEFSSLPSYAAFATAQEAYEQAVANGDSEVVLKKLKKSLNVAKSEFDRDAAMQRKLEKMA     4005
MT093571.1      SEFSSLPSYAAFATAQEAYEQAVANGDSEVVLKKLKKSLNVAKSEFDRDAAMQRKLEKMA     4005
MN996529.1      SEFSSLPSYAAFATAQEAYEQAVANGDSEVVLKKLKKSLNVAKSEFDRDAAMQRKLEKMA     4005
MT072688.1      SEFSSLPSYAAFATAQEAYEQAVANGDSEVVLKKLKKSLNVAKSEFDRDAAMQRKLEKMA     4005
NC_045512.2     SEFSSLPSYAAFATAQEAYEQAVANGDSEVVLKKLKKSLNVAKSEFDRDAAMQRKLEKMA     4005
MN994467.1      SEFSSLPSYAAFATAQEAYEQAVANGDSEVVLKKLKKSLNVAKSEFDRDAAMQRKLEKMA     4005
MG772933.1      SEFSSLPSYAAYATAQEAYEQAVANGDSEVVLKKLKKSLNVAKSEFDRDAAMQRKLEKMA     4001
MG772934.1      SKFSSLPSYAAYATAQEAYEQAVVNGDSEVVLKKLKKSLNVAKSEFDRDAAMQRKLEKMA     3979
NC_014470.1     SEFSSLPSYAAYATAQEAYEQAVANGDSEVVLKKLKKSLNVAKSEFDRDAAMQRKLEKMA     3973
GQ153542.1      SEFSSLPSYAAYATAQEAYEQAVSNGDSEVVLKKLKKSLNVAKSEFDRDAAMQRKLEKMA     3980
DQ022305        SEFSSLPSYAAYATAQEAYEQAVSNGDSEVVLKKLKKSLNVAKSEFDHDAAMQRKLEKMA     3976
KF569996.1      SEFSSLPSYAAYATAQEAYEQAVANGDSEVVLKKLKKSLNVAKSEFDRDAAMQRKLEKMA     3982
KP886809.1      SEFSSLPSYAAYATAQEAYEQAVANGDSEVVLKKLKKSLNVAKSEFDRDAAMQRKLEKMA     3982
AY278488.2      SEFSSLPSYAAYATAQEAYEQAVANGDSEVVLKKLKKSLNVAKSEFDRDAAMQRKLEKMA     3982
AY485277.1      SEFSSLPSYAAYATAQEAYEQAVANGDSEVVLKKLKKSLNVAKSEFDRDAAMQRKLEKMA     3982
AP006560.1      SEFSSLPSYAAYATAQEAYEQAVANGDSEVVLKKLKKSLNVAKSEFDRDAAMQRKLEKMA     3982
AP006557.1      SEFSSLPSYAAYATAQEAYEQAVANGDSEVVLKKLKKSLNVAKSEFDRDAAMQRKLEKMA     3982
AY274119        SEFSSLPSYAAYATAQEAYEQAVANGDSEVVLKKLKKSLNVAKSEFDRDAAMQRKLEKMA     3982
AY572038.1      SEFSSLPSYAAYATAQEAYEQAVANGDSEAVLKKLKKSLNVAKSEFDRDAAMQRKLEKMA     3982
AY572034.1      SEFSSLPSYAAYATAQEAYEQAVANGDSEAVLKKLKKSLNVAKSEFDRDAAMQRKLEKMA     3982
FJ588686.1      SEFSSLPSYAAYATAQEAYEQAVANGDSEVVLKKLKKSLNVAKSEFDRDAAMQRKLEKMA     3789
KY417145.1      SEFSSLPSYATYATAQEAYEQAVANGDSEVVLKKLKKSLNVAKSEFDRDAAMQRKLEKMA     3982
KY417144.1      SEFSSLPSYAAYATAQEAYEQAVANGDSEVVLKKLKKSLNVAKSEFDRDAAMQRKLEKMA     3982
KY417147.1      SEFSSLPSYAAYATAQEAYEQAVANGDSEVVLKKLKKSLNVAKSEFDRDAAMQRKLEKMA     3982
KY417148.1      SEFSSLPSYAAYATAQEAYEQAVANGDSEVVLKKLKKSLNVAKSEFDRDAAMQRKLEKMA     3982
KY417143.1      SEFSSLPSYAAYATAQEAYEQAVANGDSEVVLKKLKKSLNVAKSEFDRDAAMQRKLEKMA     3982
KT444582.1      SEFSSLPSYAAYATAQEAYEQAVANGDSEVVLKKLKKSLNVAKSEFDRDAAMQRKLEKMA     3982
KC881005.1      SEFSSLPSYAAYATAQEAYEQAVANGDSEVVLKKLKKSLNVAKSEFDRDAAMQRKLEKMA     3982
KC881006.1      SEFSSLPSYAAYATAQEAYEQAVANGDSEVVLKKLKKSLNVAKSEFDRDAAMQRKLEKMA     3982
KF367457.1      SEFSSLPSYAAYATAQEAYEQAVANGDSEVVLKKLKKSLNVAKSEFDRDAAMQRKLEKMA     3982
KY417152.1      SEFSSLPSYAAYATAQEAYEQAVANGDSEVVLKKLKKSLNVAKSEFDRDAAMQRKLEKMA     3982
KY417146.1      SEFSSLPSYAAYATAQEAYEQAVANGDSEVVLKKLKKSLNVAKSEFDRDAAMQRKLEKMA     3982
KY417151.1      SEFSSLPSYAAYATAQEAYEQAVANGDSEVVLKKLKKSLNVAKSEFDRDAAMQRKLEKMA     3982
KY417142.1      SEFSSLPSYAAYATAQEAYEQAVANGDSEVVLKKLKKSLNVAKSEFDRDAAMQRKLEKMA     3982
                *:********::*********** *****.*****************:************
```

CoVAX_ORF1ab#12

MW532698.1	DQAMTQMYKQARSEDKRAKVTSAMQTMLFTMLRKLDNDALNNIINNARDGCVPLNIIPLT	4057
MT040336.1	DQAMTQMYKQARSEDKRAKVTSAMQTMLFTMLRKLDNDALNNIINNARDGCVPLNIIPLT	4057
MT040335.1	DQAMTQMYKQARSEDKRAKVTSAMQTMLFTMLRKLDNDALNNIINNARDGCVPLNIIPLT	4057
MT040334.1	DQAMTQMYKQARSEDKRAKVTSAMQTMLFTMLRKLDNDALNNIINNARDGCVPLNIIPLT	4057
MT040333.1	DQAMTQMYKQARSEDKRAKVTSAMQTMLFTMLRKLDNDALNNIINNARDGCVPLNIIPLT	4057
MN996532.2	DQAMTQMYKQARSEDKRAKVTSAMQTMLFTMLRKLDNDALNNIINNARDGCVPLNIIPLT	4064
MN988713.1	DQAMTQMYKQARSEDKRAKVTSAMQTMLFTMLRKLDNDALNNIINNARDGCVPLNIIPLT	4065
MT093571.1	DQAMTQMYKQARSEDKRAKVTSAMQTMLFTMLRKLDNDALNNIINNARDGCVPLNIIPLT	4065
MN996529.1	DQAMTQMYKQARSEDKRAKVTSAMQTMLFTMLRKLDNDALNNIINNARDGCVPLNIIPLT	4065
MT072688.1	DQAMTQMYKQARSEDKRAKVTSAMQTMLFTMLRKLDNDALNNIINNARDGCVPLNIIPLT	4065
**NC_045512.2**	**DQAMTQMYKQARSEDKRAKVTSAMQTMLFTMLRKLDNDALNNIINNARDGCVPLNIIPLT**	**4065**
MN994467.1	DQAMTQMYKQARSEDKRAKVTSAMQTMLFTMLRKLDNDALNNIINNARDGCVPLNIIPLT	4065
MG772933.1	DQAMTQMYKQARSEDKRAKVTSAMQTMLFTMLRKLDNDALNNIINNARDGCVPLNIIPLT	4061
MG772934.1	DQTMTQMYKQARSEDKRAKVTSAMQTMLFTMLRKLDNDALNNIINNARDGCVPLNIIPLT	4039
NC_014470.1	DQAMTQMYKQARSEDKRAKVTSAMQTMLFTMLRKLDNDALNNIINNARDGCVPLNIIPLT	4033
GQ153542.1	DQAMTQMYKQARSEDKRAKVTSAMQTMLFTMLRKLDNDALNNIINNARDGCVPLNIIPLT	4040
DQ022305	DQAMTQMYKQARSEDKRAKVTSAMQTMLFTMLRKLDNDALNNIINNARDGCVPLNIIPLT	4036
KF569996.1	DQAMTQMYKQARSEDKRAKVTSAMQTMLFTMLRKLDNDALNNIINNARDGCVPLNIIPLT	4042
KP886809.1	DQAMTQMYKQARSEDKRAKVTSAMQTMLFTMLRKLDNDALNNIINNARDGCVPLNIIPLT	4042
AY278488.2	DQAMTQMYKQARSEDKRAKVTSAMQTMLFTMLRKLDNDALNNIINNARDGCVPLNIIPLT	4042
AY485277.1	DQAMTQMYKQARSEDKRAKVTSAMQTMLFTMLRKLDNDALNNIINNARDGCVPLNIIPLT	4042
AP006560.1	DQAMTQMYKQARSEDKRAKVTSAMQTMLFTMLRKLDNDALNNIINNARDGCVPLNIIPLT	4042
AP006557.1	DQAMTQMYKQARSEDKRAKVTSAMQTMLFTMLRKLDNDALNNIINNARDGCVPLNIIPLT	4042
AY274119	DQAMTQMYKQARSEDKRAKVTSAMQTMLFTMLRKLDNDALNNIINNARDGCVPLNIIPLT	4042
AY572038.1	DQAMTQMYKQARSEDKRAKVTSAMQTMLFTMLRKLDNDALNNIINNARDGCVPLNIIPLT	4042
AY572034.1	DQAMTQMYKQARSEDKRAKVTSAMQTMLFTMLRKLDNDALNNIINNARDGCVPLNIIPLT	4042
FJ588686.1	DQAMTQMYKQARSEDKRAKVTSAMQTMLFTMLRKLDNDALNNIINNARDGCVPLNIIPLT	3849
KY417145.1	DQAMTQMYKQARSEDKRAKVTSAMQTMLFTMLRKLDNDALNNIINNARDGCVPLNIIPLT	4042
KY417144.1	DQAMTQMYKQARSEDKRAKVTSAMQTMLFTMLRKLDNDALNNIINNARDGCVPLNIIPLT	4042
KY417147.1	DQAMTQMYKQARSEDKRAKVTSAMQTMLFTMLRKLDNDALNNIINNARDGCVPLNIIPLT	4042
KY417148.1	DQAMTQMYKQARSEDKRAKVTSAMQTMLFTMLRKLDNDALNNIINNARDGCVPLNIIPLT	4042
KY417143.1	DQAMTQMYKQARSEDKRAKVTSAMQTMLFTMLRKLDNDALNNIINNARDGCVPLNIIPLT	4042
KT444582.1	DQAMTQMYKQARSEDKRAKVTSAMQTMLFTMLRKLDNDALNNIINNARDGCVPLNIIPLT	4042
KC881005.1	DQAMTQMYKQARSEDKRAKVTSAMQTMLFTMLRKLDNDALNNIINNARDGCVPLNIIPLT	4042
KC881006.1	DQAMTQMYKQARSEDKRAKVTSAMQTMLFTMLRKLDNDALNNIINNARDGCVPLNIIPLT	4042
KF367457.1	DQAMTQMYKQARSEDKRAKVTSAMQTMLFTMLRKLDNDALNNIINNARDGCVPLNIIPLT	4042
KY417152.1	DQAMTQMYKQARSEDKRAKVTSAMQTMLFTMLRKLDNDALNNIINNARDGCVPLNIIPLT	4042
KY417146.1	DQAMTQMYKQARSEDKRAKVTSAMQTMLFTMLRKLDNDALNNIINNARDGCVPLNIIPLT	4042
KY417151.1	DQAMTQMYKQARSEDKRAKVTSAMQTMLFTMLRKLDNDALNNIINNARDGCVPLNIIPLT	4042
KY417142.1	DQAMTQMYKQARSEDKRAKVTSAMQTMLFTMLRKLDNDALNNIINNARDGCVPLNIIPLT	4042
	**:***	

CoVAX_ORF1ab#12

```
MW532698.1      TAAKLMVVVPDYNTYKNTCEGSTFTYASALWEIQQVVDADSKIVQLSEITMDNSPNIAWP    4117
MT040336.1      TAAKLMVVVPDYNTYKNTCEGSTFTYASALWEIQQVVDADSKIVQLSEITMDNSPNIAWP    4117
MT040335.1      TAAKLMVVVPDYNTYKNTCEGSTFTYASALWEIQQVVDADSKIVQLSEITMDNSPNIAWP    4117
MT040334.1      TAAKLMVVVPDYNTYKNTCEGSTFTYASALWEIQQVVDADSKIVQLSEITMDNSPNIAWP    4117
MT040333.1      TAAKLMVVVPDYNTYKNTCEGSTFTYASALWEIQQVVDADSKIVQLSEITMDNSPNIAWP    4117
MN996532.2      TAAKLMVVIPDYNTYKNTCDGTTFTYASALWEIQQVVDADSKIVQLSEISMDNSPNLAWP    4124
MN988713.1      TAAKLMVVIPDYNTYKNTCDGTTFTYASALWEIQQVVDADSKIVQLSEISMDNSPNLAWP    4125
MT093571.1      TAAKLMVVIPDYNTYKNTCDGTTFTYASALWEIQQVVDADSKIVQLSEISMDNSPNLAWP    4125
MN996529.1      TAAKLMVVIPDYNTYKNTCDGTTFTYASALWEIQQVVDADSKIVQLSEISMDNSPNLAWP    4125
MT072688.1      TAAKLMVVIPDYNTYKNTCDGTTFTYASALWEIQQVVDADSKIVQLSEISMDNSPNLAWP    4125
NC_045512.2     TAAKLMVVIPDYNTYKNTCDGTTFTYASALWEIQQVVDADSKIVQLSEISMDNSPNLAWP    4125
MN994467.1      TAAKLMVVIPDYNTYKNTCDGTTFTYASALWEIQQVVDADSKIVQLSEISMDNSPNLAWP    4125
MG772933.1      TAAKLMVVVPDYGTYKNTCDGNTFTYASALWEIQQVVDADSKIVQLSEINMDNSPNLAWP    4121
MG772934.1      TAAKLMVVVPDYGTYKNTCDGNTFTYASALWEIQQVVDADSKIVQLSEINMDNSPNLAWP    4099
NC_014470.1     TAAKLMVVVPDYNTYKNTCDGNTFTYASALWEIQQVVDADSKVVQLSEINMDNSQNLAWP    4093
GQ153542.1      TAAKLMVVVPDYGTYKNTCDGNTFTYASALWEIQQVVDADSKIVQLSEINMDNSSNLAWP    4100
DQ022305        TAAKLMVVVPDYGTYKNTCDGNTFTYASALWEIQQVVDADSKIVQLSEINMDNSPNLAWP    4096
KF569996.1      TAAKLMVVVPDYGTYKNTCDGNTFTYASALWEIQQVVDADSKIVQLSEINMDNSPNLAWP    4102
KP886809.1      TAAKLMVVVPDYGTYKNTCDGNTFTYASALWEIQQVVDADSKIIQLSEINMDNSPNLAWP    4102
AY278488.2      TAAKLMVVVPDYGTYKNTCDGNTFTYASALWEIQQVVDADSKIVQLSEINMDNSPNLAWP    4102
AY485277.1      TAAKLMVVVPDYGTYKNTCDGNTFTYASALWEIQQVVDADSKIVQLSEINMDNSPNLAWP    4102
AP006560.1      TAAKLMVVVPDYGTYKNTCDGNTFTYASALWEIQQVVDADSKIVQLSEINMDNSPNLAWP    4102
AP006557.1      TAAKLMVVVPDYGTYKNTCDGNTFTYASALWEIQQVVDADSKIVQLSEINMDNSPNLAWP    4102
AY274119        TAAKLMVVVPDYGTYKNTCDGNTFTYASALWEIQQVVDADSKIVQLSEINMDNSPNLAWP    4102
AY572038.1      TAAKLMVVVPDYGTYKNTCDGNTFTYASALWEIQQVVDADSKIVQLSEINMDNSPNLAWP    4102
AY572034.1      TAAKLMVVVPDYGTYKNTCDGNTFTYASALWEIQQVVDADSKIVQLSEINMDNSPNLAWP    4102
FJ588686.1      TAAKLMVVVPDYGTYKNTCDGNTFTYASALWEIQQVVDADSKIVQLSEINMDNSPNLAWP    3909
KY417145.1      TAAKLMVVVPDYGTYKNTCDGNTFTYASALWEIQQVVDADSKIVQLSEINMDNSPNLAWP    4102
KY417144.1      TAAKLMVVVPDYGTYKNTCDGNTFTYASALWEIQQVVDADSKIVQLSEINMDNSPNLAWP    4102
KY417147.1      TAAKLMVVVPDYGTYKNTCDGNTFTYASALWEIQQVVDADSKIVQLSEINMDNSPNLAWP    4102
KY417148.1      TAAKLMVVVPDYGTYKNTCDGNTFTYASALWEIQQVVDADSKIVQLSEINMDNSPNLAWP    4102
KY417143.1      TAAKLMVVVPDYGTYKNTCDGNTFTYASALWEIQQVVDADSKIVQLSEINMDNSPNLAWP    4102
KT444582.1      TAAKLMVVVPDYGTYKNTCDGNTFTYASALWEIQQVVDADSKIVQLSEINMDNSPNLAWP    4102
KC881005.1      TAAKLMVVVPDYGTYKNTCDGNTFTYASALWEIQQVVDADSKIVQLSEINMDNSPNLAWP    4102
KC881006.1      TAAKLMVVVPDYGTYKNTCDGNTFTYASALWEIQQVVDADSKIVQLSEINMDNSPNLAWP    4102
KF367457.1      TAAKLMVVVPDYGTYKNTCDGNTFTYASALWEIQQVVDADSKIVQLSEINMDNSPNLAWP    4102
KY417152.1      TAAKLMVVVPDYGTYKNTCDGNTFTYASALWEIQQVVDADSKIVQLSEINMDNSPNLAWP    4102
KY417146.1      TAAKLMVVVPDYGTYKNTCDGNTFTYASALWEIQQVVDADSKIVQLSEINMDNSSNLAWP    4102
KY417151.1      TAAKLMVVVPDYGTYKNTCDGNTFTYASALWEIQQVVDADSKIVQLSEINMDNSPNLAWP    4102
KY417142.1      TAAKLMVVVPDYGTYKNTCDGNTFTYASALWEIQQVVDADSKIVQLSEINMDNSPNLAWP    4102
                ********:***.******:*.********************::*****.**** *:***
```

```
MW532698.1      LIVTALRANSAVKLQNNELSPVALRQMSCAAGTTQTACNEDNALAYYNTSKGGRFVLALL    4177
MT040336.1      LIVTALRANSAVKLQNNELSPVALRQMSCAAGTTQTACNEDNALAYYNTSKGGRFVLALL    4177
MT040335.1      LIVTALRANSAVKLQNNELSPVALRQMSCAAGTTQTACNEDNALAYYNTSKGGRFVLALL    4177
MT040334.1      LIVTALRANSAVKLQNNELSPVALRQMSCAAGTTQTACNEDNALAYYNTSKGGRFVLALL    4177
MT040333.1      LIVTALRANSAVKLQNNELSPVALRQMSCAAGTTQTACNEDNALAYYNTSKGGRFVLALL    4177
MN996532.2      LIVTALRANSAVKLQNNELSPVALRQMSCAAGTTQTACTDDNALAYYNTTKGGRFVLALL    4184
MN988713.1      LIVTALRANSAVKLQNNELSPVALRQMSCAAGTTQTACTDDNALAYYNTTKGGRFVLALL    4185
MT093571.1      LIVTALRANSAVKLQNNELSPVALRQMSCAAGTTQTACTDDNALAYYNTTKGGRFVLALL    4185
MN996529.1      LIVTALRANSAVKLQNNELSPVALRQMSCAAGTTQTACTDDNALAYYNTTKGGRFVLALL    4185
MT072688.1      LIVTALRANSAVKLQNNELSPVALRQMSCAAGTTQTACTDDNALAYYNTTKGGRFVLALL    4185
NC_045512.2     LIVTALRANSAVKLQNNELSPVALRQMSCAAGTTQTACTDDNALAYYNTTKGGRFVLALL    4185
MN994467.1      LIVTALRANSAVKLQNNELSPVALRQMSCAAGTTQTACTDDNALAYYNTTKGGRFVLALL    4185
MG772933.1      LIVTALRANSAVKLQNNELSPVALRQMSCAAGTTQTACTDDNALAYYNNSKGGRFVLALL    4181
MG772934.1      LIVTALRANSAVKLQNNELSPVALRQMSCAAGTTQTACTDDNALAYYNNSKGGRFVLALL    4159
NC_014470.1     LIVTALRSNSAVKLQNNELSPVALRQMSCAAGTTQTACTDDNALAYYNTSKGGRFVLALL    4153
GQ153542.1      LIVTALRANSAVKLQNNELSPVALRQMSCAAGTTQTACTDDNALAYYNNAKGGRFVLALL    4160
DQ022305        LIVTALRANSAVKLQNNELSPVALRQMSCAAGTTQTACTDDNALAYYNNAKGGRFVLALL    4156
KF569996.1      LIVTALRANSAVKLQNNELSPVALRQMSCAAGTTQTACTDDNALAYYNNSKGGRFILALL    4162
KP886809.1      LIVTALRANSAVKLQNNELSPVALRQMSCAAGTTQTACTDDNALAYYNNSKGGRFVLALL    4162
AY278488.2      LIVTALRANSAVKLQNNELSPVALRQMSCAAGTTQTACTDDNALAYYNNSKGGRFVLALL    4162
AY485277.1      LIVTALRANSAVKLQNNELSPVALRQMSCAAGTTQTACTDDNALAYYNNSKGGRFVLALL    4162
AP006560.1      LIVTALRANSAVKLQNNELSPVALRQMSCAAGTTQTACTDDNALAYYNNSKGGRFVLALL    4162
AP006557.1      LIVTALRANSAVKLQNNELSPVALRQMSCAAGTTQTACTDDNALAYYNNSKGGRFVLALL    4162
AY274119        LIVTALRANSAVKLQNNELSPVALRQMSCAAGTTQTACTDDNALAYYNNSKGGRFVLALL    4162
AY572038.1      LIVTALRANSAVKLQNNELSPVALRQMSCAAGTTQTACTDDNALAYYNNSKGGRFVLALL    4162
AY572034.1      LIVTALRANSAVKLQNNELSPVALRQMSCAAGTTQTACTDDNALAYYNNSKGGRFVLALL    4162
FJ588686.1      LIVTALRANSAVKLQNNELSPVALRQMSCAAGTTQTACTDDNALAYYNNSKGGRFVLALL    3969
KY417145.1      LIVTAIRANSAVKLQNNELSPVALRQMSCAAGTTQTACTDDNALAYYNNSKGGRFVLALL    4162
KY417144.1      LIVTALRANSAVKLQNNELSPVALRQMSCAAGTTQTACTDDNALAYYNNSKGGRFVLALL    4162
KY417147.1      LIVTALRANSAVKLQNNELSPVALRQMSCAAGTTQTACTDDNALAYYNNSKGGRFVLALL    4162
KY417148.1      LIVTALRANSAVKLQNNELSPVALRQMSCAAGTTQTACTDDNALAYYNNSKGGRFVLALL    4162
KY417143.1      LIVTALRANSAVKLQNNELSPVALRQMSCAAGTTQTACTDDNALAYYNNSKGGRFVLALL    4162
KT444582.1      LIVTALRANSAVKLQNNELSPVALRQMSCAAGTTQTACTDDNALAYYNNSKGGRFVLALL    4162
KC881005.1      LIVTALRANSAVKLQNNELSPVALRQMSCAAGTTQTACTDDNALAYYNNSKGGRFVLALL    4162
KC881006.1      LIVTALRANSAVKLQNNELSPVALRQMSCAAGTTQTACTDDNALAYYNNSKGGRFVLALL    4162
KF367457.1      LIVTALRANSAVKLQNNELSPVALRQMSCAAGTTQTACTDDNALAYYNNSKGGRFVLALL    4162
KY417152.1      LIVTALRANSAVKLQNNELSPVALRQMSCAAGTTQTACTDDNALAYYNNSKGGRFVLALL    4162
KY417146.1      LIVTALRANSAVKLQNNELSPVALRQMSCAAGTTQTACTDDNALAYYNNSKGGRFVLALL    4162
KY417151.1      LIVTALRANSAVKLQNNELSPVALRQMSCAAGTTQTACTDDNALAYYNNSKGGRFVLALL    4162
KY417142.1      LIVTALRANSAVKLQNNELSPVALRQMSCAAGTTQTACTDDNALAYYNNSKGGRFVLALL    4162
                *****:*:******************************.:********.:*****:****
```

MW532698.1	SDLQDLKWARFPKSDGTGTIYTELEPPCRFVTDTPKGPKVKYLYFIKGLNNLNRGMVLGS	4237
MT040336.1	SDLQDLKWARFPKSDGTGTIYTELEPPCRFVTDTPKGPKVKYLYFIKGLNNLNRGMVLGS	4237
MT040335.1	SDLQDLKWARFPKSDGTGTIYTELEPPCRFVTDTPKGPKVKYLYFIKGLNNLNRGMVLGS	4237
MT040334.1	SDLQDLKWARFPKSDGTGTIYTELEPPCRFVTDTPKGPKVKYLYFIKGLNNLNRGMVLGS	4237
MT040333.1	SDLQDLKWARFPKSDGTGTIYTELEPPCRFVTDTPKGPKVKYLYFIKGLNNLNRGMVLGS	4237
MN996532.2	SDLQDLKWARFPKSDGTGTIYTELEPPCRFVTDTPKGPKVKYLYFIKGLNNLNRGMVLGS	4244
MN988713.1	SDLQDLKWARFPKSDGTGTIYTELEPPCRFVTDTPKGPKVKYLYFIKGLNNLNRGMVLGS	4245
MT093571.1	SDLQDLKWARFPKSDGTGTIYTELEPPCRFVTDTPKGPKVKYLYFIKGLNNLNRGMVLGS	4245
MN996529.1	SDLQDLKWARFPKSDGTGTIYTELEPPCRFVTDTPKGPKVKYLYFIKGLNNLNRGMVLGS	4245
MT072688.1	SDLQDLKWARFPKSDGTGTIYTELEPPCRFVTDTPKGPKVKYLYFIKGLNNLNRGMVLGS	4245
**NC_045512.2**	**SDLQDLKWARFPKSDGTGTIYTELEPPCRFVTDTPKGPKVKYLYFIKGLNNLNRGMVLGS**	**4245**
MN994467.1	SDLQDLKWARFPKSDGTGTIYTELEPPCRFVTDTPKGPKVKYLYFIKGLNNLNRGMVLGS	4245
MG772933.1	SDHQDLKWARFPKSDGTGTIYTELEPPCRFVTDTPKGPKVKYLYFIKGLNNLNRGMVLGS	4241
MG772934.1	SDHQDLKWSRFPKSDGTGTIYTELEPPCRFVTDTPKGPKVKYLYFIKGLNNLNRGMVLGS	4219
NC_014470.1	SDHQDLKWARFPKSDGTGTIYTELEPPCRFVTDTPKGPKVKYLYFIKGLNNLNRGMVLGS	4213
GQ153542.1	SDHQDLKWARFPKSDGTGTIYTELEPPCRFVTDTPKGPKVKYLYFIKGLNNLNRGMVLGS	4220
DQ022305	SDHQDLKWARFPKSDGTGTIYTELEPPCRFVTDTPKGPKVKYLYFIKGLNNLNRGMVLGS	4216
KF569996.1	SDHQDLKWARFPKSDGTGTIYTELEPPCRFVTDTPKGPKVKYLYFIKGLNNLNRGMVLGS	4222
KP886809.1	SDHQDLKWARFPKSDGTGTIYTELEPPCRFVTDTPKGPKVKYLYFIKGLNNLNRGMVLGS	4222
AY278488.2	SDHQDLKWARFPKSDGTGTIYTELEPPCRFVTDTPKGPKVKYLYFIKGLNNLNRGMVLGS	4222
AY485277.1	SDHQDLKWARFPKSDGTGTIYTELEPPCRFVTDTPKGPKVKYLYFIKGLNNLNRGMVLGS	4222
AP006560.1	SDHQDLKWARFPKSDGTGTIYTELEPPCRFVTDTPKGPKVKYLYFIKGLNNLNRGMVLGS	4222
AP006557.1	SDHQDLKWARFPKSDGTGTIYTELEPPCRFVTDTPKGPKVKYLYFIKGLNNLNRGMVLGS	4222
AY274119	SDHQDLKWARFPKSDGTGTIYTELEPPCRFVTDTPKGPKVKYLYFIKGLNNLNRGMVLGS	4222
AY572038.1	SDHQDLKWARFPKSDGTGTIYTELEPPCRFVTDTPKGPKVKYLYFIKGLNNLNRGMVLGS	4222
AY572034.1	SDHQDLKWARFPKSDGTGTIYTELEPPCRFVTDTPKGPKVKYLYFIKGLNNLNRGMVLGS	4222
FJ588686.1	SDHQDLKWARFPKSDGTGTIYTELEPPCRFVTDTPKGPKVKYLYFIKGLNNLNRGMVLGS	4029
KY417145.1	SDHQDLKWARFPKSDGTGTIYTELEPPCRFVTDTPKGPKVKYLYFIKGLNNLNRGMVLGS	4222
KY417144.1	SDHQDLKWARFPKSDGTGTIYTELEPPCRFVTDTPKGPKVKYLYFIKGLNNLNRGMVLGS	4222
KY417147.1	SDQQDLKWARFPKSDGTGTIYTELEPPCRFVTDTPKGPKVKYLYFIKGLNNLNRGMVLGS	4222
KY417148.1	SDQQDLKWARFPKSDGTGTIYTELEPPCRFVTDTPKGPKVKYLYFIKGLNNLNRGMVLGS	4222
KY417143.1	SDHQDLKWARFPKSDGTGTIYTELEPPCRFVTDTPKGPKVKYLYFIKGLNNLNRGMVLGS	4222
KT444582.1	SDHQDLKWARFPKSDGTGTIYTELEPPCRFVTDTPKGPKVKYLYFIKGLNNLNRGMVLGS	4222
KC881005.1	SDHQDLKWARFPKSDGTGTIYTELEPPCRFVTDTPKGPKVKYLYFIKGLNNLNRGMVLGS	4222
KC881006.1	SDHQDLKWARFPKSDGTGTIYTELEPPCRFVTDTPKGPKVKYLYFIKGLNNLNRGMVLGS	4222
KF367457.1	SDHQDLKWARFPKSDGTGTIYTELEPPCRFVTDTPKGPKVKYLYFIKGLNNLNRGMVLGS	4222
KY417152.1	SDHQDLKWARFPKSDGTGTIYTELEPPCRFVTDTPKGPKVKYLYFIKGLNNLNRGMVLGS	4222
KY417146.1	SDHQDLKWARFPKSDGTGTIYTELEPPCRFVTDTPKGPKVKYLYFIKGLNNLNRGMVLGS	4222
KY417151.1	SDHQDLKWARFPKSDGTGTIYTELEPPCRFVTDTPKGPKVKYLYFIKGLNNLNRGMVLGS	4222
KY417142.1	SDHQDLKWARFPKSDGTGTIYTELEPPCRFVTDTPKGPKVKYLYFIKGLNNLNRGMVLGS	4222
	** *****:***	

MW532698.1	LAATVRLQAGNATEVPANSTVLSFCAFAVDASKAYRDYLASGGQPITNCVKMLCTHTGTG	4297
MT040336.1	LAATVRLQAGNATEVPANSTVLSFCAFAVDASKAYRDYLASGGQPITNCVKMLCTHTGTG	4297
MT040335.1	LAATVRLQAGNATEVPANSTVLSFCAFAVDASKAYRDYLASGGQPITNCVKMLCTHTGTG	4297
MT040334.1	LAATVRLQAGNATEVPANSTVLSFCAFAVDASKAYRDYLASGGQPITNCVKMLCTHTGTG	4297
MT040333.1	LAATVRLQAGNATEVPANSTVLSFCAFAVDASKAYRDYLASGGQPITNCVKMLCTHTGTG	4297
MN996532.2	LAATVRLQAGNATEVPANSTVLSFCAFAVDAAKAYKDYLASGGQPITNCVKMLCTHTGTG	4304
MN988713.1	LAATVRLQAGNATEVPANSTVLSFCAFAVDAAKAYKDYLASGGQPITNCVKMLCTHTGTG	4305
MT093571.1	LAATVRLQAGNATEVPANSTVLSFCAFAVDAAKAYKDYLASGGQPITNCVKMLCTHTGTG	4305
MN996529.1	LAATVRLQAGNATEVPANSTVLSFCAFAVDAAKAYKDYLASGGQPITNCVKMLCTHTGTG	4305
MT072688.1	LAATVRLQAGNATEVPANSTVLSFCAFAVDAAKAYKDYLASGGQPITNCVKMLCTHTGTG	4305
**NC_045512.2**	**LAATVRLQAGNATEVPANSTVLSFCAFAVDAAKAYKDYLASGGQPITNCVKMLCTHTGTG**	**4305**
MN994467.1	LAATVRLQAGNATEVPANSTVLSFCAFAVDAAKAYKDYLASGGQPITNCVKMLCTHTGTG	4305
MG772933.1	LAATVRLQAGNATEVPANSTVLSFCAFAVDPAKAYKDYLASGGQPITNCVKMLCTHTGTG	4301
MG772934.1	LAATVRLQAGNATEVPANSTVLSFCAFAVDPAKAYKDYLASGGQPITNCVKMLCTHTGTG	4279
NC_014470.1	LAATVRLQAGNATEVPANSTVLSFCAFAVDPAKAYKDYLASGGQPITNCVKMLCTHTGTG	4273
GQ153542.1	LAATVRLQAGNATEVPANSTVLSFCAFAVDPAKAYKDYLASGGQPITNCVKMLCTHTGTG	4280
DQ022305	LAATVRLQAGNATEVPANSTVLSFCAFAVDPAKAYKDYLASGGQPITNCVKMLCTHTGTG	4276
KF569996.1	LAATVRLQAGNATEVPANSTVLSFCAFAVDPAKAYKDYLASGGQPITNCVKMLCTHTGTG	4282
KP886809.1	LAATVRLQAGNATEVPANSTVLSFCAFAVDPAKAYKDYLASGGQPITNCVKMLCTHTGTG	4282
AY278488.2	LAATVRLQAGNATEVPANSTVLSFCAFAVDPAKAYKDYLASGGQPITNCVKMLCTHTGTG	4282
AY485277.1	LAATVRLQAGNATEVPANSTVLSFCAFAVDPAKAYKDYLASGGQPITNCVKMLCTHTGTG	4282
AP006560.1	LAATVRLQAGNATEVPANSTVLSFCAFAVDPAKAYKDYLASGGQPITNCVKMLCTHTGTG	4282
AP006557.1	LAATVRLQAGNATEVPANSTVLSFCAFAVDPAKAYKDYLASGGQPITNCVKMLCTHTGTG	4282
AY274119	LAATVRLQAGNATEVPANSTVLSFCAFAVDPAKAYKDYLASGGQPITNCVKMLCTHTGTG	4282
AY572038.1	LAATVRLQAGNATEVPANSTVLSFCAFAVDPAKAYKDYLASGGQPITNCVKMLCTHTGTG	4282
AY572034.1	LAATVRLQAGNATEVPANSTVLSFCAFAVDPAKAYKDYLASGGQPITNCVKMLCTHTGTG	4282
FJ588686.1	LAATVRLQAGNATEVPANSTVLSFCAFAVDPAKAYKDYLASGGQPITNCVKMLCTHTGTG	4089
KY417145.1	LAATVRLQAGNATEVPANSTVLSFCAFAVDPAKAYKDYLASGGQPITNCVKMLCTHTGTG	4282
KY417144.1	LAATVRLQAGNATEVPANSTVLSFCAFAVDPAKAYKDYLASGGQPITNCVKMLCTHTGTG	4282
KY417147.1	LAATVRLQAGNATEVPANSTVLSFCAFAVDPAKAYKDYLASGGQPITNCVKMLCTHTGTG	4282
KY417148.1	LAATVRLQAGNATEVPANSTVLSFCAFAVDPAKAYKDYLASGGQPITNCVKMLCTHTGTG	4282
KY417143.1	LAATVRLQAGNATEVPANSTVLSFCAFAVDPAKAYKDYLASGGQPITNCVKMLCTHTGTG	4282
KT444582.1	LAATVRLQAGNATEVPANSTVLSFCAFAVDPAKAYKDYLASGGQPITNCVKMLCTHTGTG	4282
KC881005.1	LAATVRLQAGNATEVPANSTVLSFCAFAVDPAKAYKDYLASGGQPITNCVKMLCTHTGTG	4282
KC881006.1	LAATVRLQAGNATEVPANSTVLSFCAFAVDPAKAYKDYLASGGQPITNCVKMLCTHTGTG	4282
KF367457.1	LAATVRLQAGNATEVPANSTVLSFCAFAVDPAKAYKDYLASGGQPITNCVKMLCTHTGTG	4282
KY417152.1	LAATVRLQAGNATEVPANSTVLSFCAFAVDPAKAYKDYLASGGQPITNCVKMLCTHTGTG	4282
KY417146.1	LAATVRLQAGNATEVPANSTVLSFCAFAVDPAKAYKDYLASGGQPITNCVKMLCTHTGTG	4282
KY417151.1	LAATVRLQAGNATEVPANSTVLSFCAFAVDPAKAYKDYLASGGQPITNCVKMLCTHTGTG	4282
KY417142.1	LAATVRLQAGNATEVPANSTVLSFCAFAVDPAKAYKDYLASGGQPITNCVKMLCTHTGTG	4282
	****************************** :***:************************	

```
MW532698.1      QAITVTPEANMDQESFGGASCCLYCRCHIDHPNPKGYCELKGKYVQIPTTCANDPVGFTL    4357
MT040336.1      QAITVTPEANMDQESFGGASCCLYCRCHIDHPNPKGYCELKGKYVQIPTTCANDPVGFTL    4357
MT040335.1      QAITVTPEANMDQESFGGASCCLYCRCHIDHPNPKGYCELKGKYVQIPTTCANDPVGFTL    4357
MT040334.1      QAITVTPEANMDQESFGGASCCLYCRCHIDHPNPKGYCELKGKYVQIPTTCANDPVGFTL    4357
MT040333.1      QAITVTPEANMDQESFGGASCCLYCRCHIDHPNPKGYCELKGKYVQIPTTCANDPVGFTL    4357
MN996532.2      QAITVTPEANMDQESFGGASCCLYCRCHIDHPNPKGFCDLKGKYVQIPTTCANDPVGFTL    4364
MN988713.1      QAITVTPEANMDQESFGGASCCLYCRCHIDHPNPKGFCDLKGKYVQIPTTCANDPVGFTL    4365
MT093571.1      QAITVTPEANMDQESLGGASCCLYCRCHIDHPNPKGFCDLKGKYVQIPTTCANDPVGFTL    4365
MN996529.1      QAITVTPEANMDQESFGGASCCLYCRCHIDHPNPKGFCDLKGKYVQIPTTCANDPVGFTL    4365
MT072688.1      QAITVTPEANMDQESFGGASCCLYCRCHIDHPNPKGFCDLKGKYVQIPTTCANDPVGFTL    4365
NC_045512.2     QAITVTPEANMDQESFGGASCCLYCRCHIDHPNPKGFCDLKGKYVQIPTTCANDPVGFTL    4365
MN994467.1      QAITVTPEANMDQESFGGASCCLYCRCHIDHPNPKGFCDLKGKYVQIPTTCANDPVGFTL    4365
MG772933.1      QAITVTPEANMDQESFGGASCCLYCRCHIDHPNPKGFCDLKGKYVQIPTTCVNDPVGFTL    4361
MG772934.1      QAITVTPEANMDQESFGGASCCLYCRCHIDHPNPKGFCDLKGKYVQIPTTCANDPVGFTL    4339
NC_014470.1     QAITVIPEANMDQESFGGASCCLYCRCHIDHPNPKGFCDLKGKYVQIPTTCTNDPVGFIL    4333
GQ153542.1      QAITVTPEANMDQESFGGASCCLYCRCHIDHPNPKGFCDLKGKYVQIPTTCANDPVGFTL    4340
DQ022305        QAITVTPEANMDQESFGGASCCLYCRCHIDHPNPKGFCDLKGKYVQIPTTCANDPVGFTL    4336
KF569996.1      QAITVTPEANMDQESFGGASCCLYCRCHIDHPNPKGFCDLKGKYVQIPTTCANDPVGFTL    4342
KP886809.1      QAITVTPEANMDQESFGGASCFLYCRCQIDHPNPKGFCDLKGKYFQIPATCANDPVGFTF    4342
AY278488.2      QAITVTPEANMDQESFGGASCCLYCRCHIDHPNPKGFCDLKGKYVQIPTTCANDPVGFTL    4342
AY485277.1      QAITVTPEANMDQESFGGASCCLYCRCHIDHPNPKGFCDLKGKYVQIPTTCANDPVGFTL    4342
AP006560.1      QAITVTPEANMDQESFGGASCCLYCRCHIDHPNPKGFCDLKGKYVQIPTTCANDPVGFTL    4342
AP006557.1      QAITVTPEANMDQESFGGASCCLYCRCHIDHPNPKGFCDLKGKYVQIPTTCANDPVGFTL    4342
AY274119        QAITVTPEANMDQESFGGASCCLYCRCHIDHPNPKGFCDLKGKYVQIPTTCANDPVGFTL    4342
AY572038.1      QAITVTPEANMDQESFGGASCCLYCRCHIDHPNPKGFCDLKGKYVQIPTTCANDPVGFTL    4342
AY572034.1      QAITVTPEANMDQESFGGASCCLYCRCHIDHPNPKGFCDLKGKYVQIPTTCANDPVGFTL    4342
FJ588686.1      QAITVTPEANMDQESFGGASCCLYCRCHIDHPNPKGFCDLKGKYVQIPTTCANDPVGFTL    4149
KY417145.1      QAITVTPEANMDQESFGGASCCLYCRCHIDHPNPKGFCDLKGKYVQIPTTCANDPVGFTL    4342
KY417144.1      QAITVTPEANMDQESFGGASCCLYCRCHIDHPNPKGFCDLKGKYVQIPTTCANDPVGFTL    4342
KY417147.1      QAITVTPEANMDQESFGGASCCLYCRCHIDHPNPKGFCDLKGKYVQIPTTCANDPVGFTL    4342
KY417148.1      QAITVTPEANMDQESFGGASCCLYCRCHIDHPNPKGFCDLKGKYVQIPTTCANDPVGFTL    4342
KY417143.1      QAITVTPEANMDQESFGGASCCLYCRCHIDHPNPKGFCDLKGKYVQIPTTCANDPVGFTL    4342
KT444582.1      QAITVTPEANMDQESFGGASCCLYCRCHIDHPNPKGFCDLKGKYVQIPTTCANDPVGFTL    4342
KC881005.1      QAITVTPEANMDQESFGGASCCLYCRCHIDHPNPKGFCDLKGKYVQIPTTCANDPVGFTL    4342
KC881006.1      QAITVTPEANMDQESFGGASCCLYCRCHIDHPNPKGFCDLKGKYVQIPTTCANDPVGFTL    4342
KF367457.1      QAITVTPEANMDQESFGGASCCLYCRCHIDHPNPKGFCDLKGKYVQIPTTCANDPVGFTL    4342
KY417152.1      QAITVTPEANMDQESFGGASCCLYCRCHIDHPNPKGFCDLKGKYVQIPTTCANDPVGFTL    4342
KY417146.1      QAITVTPEANMDQESFGGASCCLYCRCHIDHPNPKGFCDLKGKYVQIPTTCANDPVGFTL    4342
KY417151.1      QAITVTPEANMDQESFGGASCCLYCRCHIDHPNPKGFCDLKGKYVQIPTTCANDPVGFTL    4342
KY417142.1      QAITVTPEANMDQESFGGASCCLYCRCHIDHPNPKGFCDLKGKYVQIPTTCANDPVGFTL    4342
                ***** *********:***** *****:********:*:*****.***:**.****** :
```

```
MW532698.1       KNTVCTVCGMWKGYGCSCDQLREPMLQSADAQSFLNRVCGVSAARLTPCGTGTSTDVVYR         4417
MT040336.1       KNTVCTVCGMWKGYGCSCDQLREPMLQSADAQSFLNRVCGVSAARLTPCGTGTSTDVVYR         4417
MT040335.1       KNTVCTVCGMWKGYGCSCDQLREPMLQSADAQSFLNRVCGVSAARLTPCGTGTSTDVVYR         4417
MT040334.1       KNTVCTVCGMWKGYGCSCDQLREPMLQSADAQSFLNRVCGVSAARLTPCGTGTSTDVVYR         4417
MT040333.1       KNTVCTVCGMWKGYGCSCDQLREPMLQSADAQSFLNRVCGVSAARLTPCGTGTSTDVVYR         4417
MN996532.2       KNTVCTVCGMWKGYGCSCDQLREPMLQSADAQSFLNRVCGVSAARLTPCGTGTSTDVVYR         4424
MN988713.1       KNTVCTVCGMWKGYGCSCDQLREPMLQSADAQSFLNRVCGVSAARLTPCGTGTSTDVVYR         4425
MT093571.1       KNTVCTVCGMWKGYGCSCDQLREPMLQSADAQSFLNRVCGVSAARLTPCGTGTSTDVVYR         4425
MN996529.1       KNTVCTVCGMWKGYGCSCDQLREPMLQSADAQSFLNRVCGVSAARLTPCGTGTSTDVVYR         4425
MT072688.1       KNTVCTVCGMWKGYGCSCDQLREPMLQSADAQSFLNRVCGVSAARLTPCGTGTSTDVVYR         4425
NC_045512.2      KNTVCTVCGMWKGYGCSCDQLREPMLQSADAQSFLNRVCGVSAARLTPCGTGTSTDVVYR         4425
MN994467.1       KNTVCTVCGMWKGYGCSCDQLREPMLQSADAQSFLNRVCGVSAARLTPCGTGTSTDVVYR         4425
MG772933.1       RNTVCTVCGMWKGYGCSCDQLREPMMQSADASTFLNRVCGVSAARLTPCGTGTSTDVVYR         4421
MG772934.1       RNTVCTVCGMWKGYGCSCDQLREPMMQSADASTFLNRVCGVSAARLTPCGSGISTDVVYR         4399
NC_014470.1      RNTVCTVCGMWKGYGCSCDQLREPVMQAADAPAFLNRVCGVSAARLTPCGTGTSTDVVYR         4393
GQ153542.1       RNTVCTVCGMWKGYGCSCDQLREPMMQSADASTFLNRVCGVSAARLTPCGTGTSTDVVYR         4400
DQ022305         RNTVCTVCGMWKGYGCSCDQLREPMMQSADASTFLNRVCGVSAARLTPCGTGTSTDVVYR         4396
KF569996.1       RNTVCTICGMWKGYGCSCDQLRESMMQSEDASTFLNGF----------------------         4380
KP886809.1       RNTVCTVCGMWKGYGCSCDQLREPMMQSADASTFLNRVCGVSAARLTPCGTGISTDVVYR         4402
AY278488.2       RNTVCTVCGMWKGYGCSCDQLREPLMQSADASTFLNRVCGVSAARLTPCGTGTSTDVVYR         4402
AY485277.1       RNTVCTVCGMWKGYGCSCDQLREPLMQSADASTFLNRVCGVSAARLTPCGTGTSTDVVYR         4402
AP006560.1       RNTVCTVCGMWKGYGCSCDQLREPLMQSADASTFLNRVCGVSAARLTPCGTGTSTDVVYR         4402
AP006557.1       RNTVCTVCGMWKGYGCSCDQLREPLMQSADASTFLNRVCGVSAARLTPCGTGTSTDVVYR         4402
AY274119         RNTVCTVCGMWKGYGCSCDQLREPLMQSADASTFFKRVCGVSAARLTPCGTGTSTDVVYR         4402
AY572038.1       RNTVCTVCGMWKGYGCSCDQLREPLMQSADASTFFKRVCGVSAARLTPCGTGTSTDVVYR         4402
AY572034.1       RNTVCTVCGMWKGYGCSCDQLREPLMQSADASTFFKRVCGVSAARLTPCGTGTSTDVVYR         4402
FJ588686.1       RNTVCTVCGMWKGYGCSCDQLREPMMQSADASTFLNRVCGVSAARLTPCGTGTSTDVVYR         4209
KY417145.1       RNTVCTVCGMWKGYGCSCDQLREPMMQSADASTFLNRVCGVSAARLTPCGTGTSTDVVYR         4402
KY417144.1       RNTVCTVCGMWKGYGCSCDQLREPMMQSADASTFLNRVCGVSAARLTPCGTGTSTDVVYR         4402
KY417147.1       RNTVCTVCGMWKGYGCSCDQLREPMMQSADASTFLNRVCGVSAARLTPCGTGTSTDVVYR         4402
KY417148.1       RNTVCTVCGMWKGYGCSCDQLREPMMQSADASTFLNRVCGVSAARLTPCGTGTSTDVVYR         4402
KY417143.1       RNTVCTVCGMWKGYGCSCDQLREPMMQSADASTFLNRVCGVSAARLTPCGTGTSTDVVYR         4402
KT444582.1       RNTVCTVCGMWKGYGCSCDQLREPMMQSADASTFLNRVCGVSAARLTPCGTGTSTDVVYR         4402
KC881005.1       RNTVCTVCGMWKGYGCSCDQLREPMMQSADASTFLNRVCGVSAARLTPCGTGTSTDVVYR         4402
KC881006.1       RNTVCTVCGMWKGYGCSCDQLREPMMQSADASTFLNRVCGVSAARLTPCGTGTSTDVVYR         4402
KF367457.1       RNTVCTVCGMWKGYGCSCDQLREPMMQSADASTFLNRVCGVSAARLTPCGTGTSTDVVYR         4402
KY417152.1       RNTVCTVCGMWKGYGCSCDQLREPMMQSADASTFLNRVCGVSAARLTPCGTGTSTDVVYR         4402
KY417146.1       RNTVCTVCGMWKGYGCSCDQLREPMMQSADASTFLNRVCGVSAARLTPCGTGTSTDVVYR         4402
KY417151.1       RNTVCTVCGMWKGYGCSCDQLREPMMQSADASTFLNRVCGVSAARLTPCGTGTSTDVVYR         4402
KY417142.1       RNTVCTVCGMWKGYGCSCDQLREPMMQSADASTFLNRVCGVSAARLTPCGTGTSTDVVYR         4402
                 :*****:**************** ::*: ** :*:: .
```

```
MW532698.1      AFDIYNEKVAGFAKFLKTNCCRFQEKDEDGNLIDSYFIVKRHTFSNYQHEEAIYNLLKDC      4477
MT040336.1      AFDIYNEKVAGFAKFLKTNCCRFQEKDEDGNLIDSYFIVKRHTFSNYQHEEAIYNLLKDC      4477
MT040335.1      AFDIYNEKVAGFAKFLKTNCCRFQEKDEDGNLIDSYFIVKRHTFSNYQHEEAIYNLLKDC      4477
MT040334.1      AFDIYNEKVAGFAKFLKTNCCRFQEKDEDGNLIDSYFIVKRHTFSNYQHEEAIYNLLKDC      4477
MT040333.1      AFDIYNEKVAGFAKFLKTNCCRFQEKDEDGNLIDSYFIVKRHTFSNYQHEEAIYNLLKDC      4477
MN996532.2      AFDIYNDKVAGFAKFLKTNCCRFQEKDEDDNLIDSYFVVKRHTFSNYQHEETIYNLLKDC      4484
MN988713.1      AFDIYNDKVAGFAKFLKTNCCRFQEKDEDDNLIDSYFVVKRHTFSNYQHEETIYNLLKDC      4485
MT093571.1      AFDIYNDKVAGFAKFLKTNCCRFQEKDEDDNLIDSYFVVKRHTFSNYQHEETIYNLLKDC      4485
MN996529.1      AFDIYNDKVAGFAKFLKTNCCRFQEKDEDDNLIDSYFVVKRHTFSNYQHEETIYNLLKDC      4485
MT072688.1      AFDIYNDKVAGFAKFLKTNCCRFQEKDEDDNLIDSYFVVKRHTFSNYQHEETIYNLLKDC      4485
NC_045512.2     AFDIYNDKVAGFAKFLKTNCCRFQEKDEDDNLIDSYFVVKRHTFSNYQHEETIYNLLKDC      4485
MN994467.1      AFDIYNDKVAGFAKFLKTNCCRFQEKDEDDNLIDSYFVVKRHTFSNYQHEETIYNLLKDC      4485
MG772933.1      AFDIYNEKVAGFAKFLKTNCCRFQEKDEEGNLLDSYFVVKRHTMSNYQHEETIYNLVKGC      4481
MG772934.1      AFDIYNEKVAGFAKFLKTNCCRFQEKDEEGNLLDSYFVVKRHTMSNYQHEENIYNLVKEC      4459
NC_014470.1     AFDIYNEKVAGFAKFLKTNCCRFQEVDEEGNLLDSYFVVKRHTMSNYQHEETMYNLVKEC      4453
GQ153542.1      AFDIYNEKVAGFAKFLKTNCCRFQEKDEEGNLLDSYFVVKRHTMSNYQHEETIYNLIKEC      4460
DQ022305        AFDIYNEKVAGFAKFLKTNCCRFQEKDEEGNLLDSYFVVKRHTMSNYQHEETIYNLIKEC      4456
KF569996.1      -----------------------------------------AVMSNYQHEETIYNLVKEC      4399
KP886809.1      AFDIYNEKVAGFAKFLKTNCCRFQEKDEEGNLLDSYFVVKRHTMSNYQHEETIYNLVKDC      4462
AY278488.2      AFDIYNEKVAGFAKFLKTNCCRFQEKDEEGNLLDSYFVVKRHTMSNYQHEETIYNLVKDC      4462
AY485277.1      AFDIYNEKVAGFAKFLKTNCCRFQEKDEEGNLLDSYFVVKRHTMSNYQHEETIYNLVKDC      4462
AP006560.1      AFDIYNEKVAGFAKFLKTNCCRFQEKDEEGNLLDSYFVVKRHTMSNYQHEETIYNLVKDC      4462
AP006557.1      AFDIYNEKVAGFAKFLKTNCCRFQEKDEEGNLLDSYFVVKRHTMSNYQHEETIYNLVKDC      4462
AY274119        AFDIYNEKVAGFAKFLKTNCCRFQEKDEEGNLLDSYFVVKRHTMSNYQHEETIYNLVKDC      4462
AY572038.1      AFDIYNEKVAGFAKFLKTNCCRFQEKDEEGNLLDSYFVVKRHTMSNYQHEETIYNLVKDC      4462
AY572034.1      AFDIYNEKVAGFAKFLKTNCCRFQEKDEEGNLLDSYFVVKRHTMSNYQHEETIYNLVKDC      4462
FJ588686.1      AFDIYNEKVAGFAKFLKTNCCRFQEKDEEGNLLDSYFVVKRHTMSNYQHEETIYNLVKDC      4269
KY417145.1      AFDIYNEKVAGFAKFLKTNCCRFQEKDEEGNLLDSYFVVKRHTMSNYQHEETIYNLVKDC      4462
KY417144.1      AFDIYNEKVAGFAKFLKTNCCRFQEKDEEGNLLDSYFVVKRHTMSNYQHEETIYNLVKNC      4462
KY417147.1      AFDIYNEKVAGFAKFLKTNCCRFQEKDEEGNLLDSYFVVKRHTMSNYQHEETIYNLVKDC      4462
KY417148.1      AFDIYNEKVAGFAKFLKTNCCRFQEKDEEGNLLDSYFVVKRHTMSNYQHEETIYNLVKDC      4462
KY417143.1      AFDIYNEKVAGFAKFLKTNCCRFQEKDEEGNLLDSYFVVKRHTMSNYQHEEAIYNLVKDC      4462
KT444582.1      AFDIYNEKVAGFAKFLKTNCCRFQEKDEEGNLLDSYFVVKRHTMSNYQHEEAIYNLVKDC      4462
KC881005.1      AFDIYNEKVAGFAKFLKTNCCRFQEKDEEGNLLDSYFVVKRHTMSNYQHEETIYNLVKNC      4462
KC881006.1      AFDIYNEKVAGFAKFLKTNCCRFQEKDEEGNLLDSYFVVKRHTMSNYQHEETIYNLVKNC      4462
KF367457.1      AFDIYNEKVAGFAKFLKTNCCRFQEKDEEGNLLDSYFVVKRHTMSNYQHEETIYNLVKNC      4462
KY417152.1      AFDIYNEKVAGFAKFLKTNCCRFQEKDEEGNLLDSYFVVKRHTMSNYQHEETIYNLVKNC      4462
KY417146.1      AFDIYNEKVAGFAKFLKTNCCRFQEKDEEGNLLDSYFVVKRHTMSNYQHEETIYNLVKDC      4462
KY417151.1      AFDIYNEKVAGFAKFLKTNCCRFQEKDEEGNLLDSYFVVKRHTMSNYQHEETIYNLVKNC      4462
KY417142.1      AFDIYNEKVAGFAKFLKTNCCRFQEKDEEGNLLDSYFVVKRHTMSNYQHEETIYNLVKNC      4462
                                                          .:******* :***:* *
```

MW532698.1	PAVAVHDFFKFRVDGDMVPHISRQRLTKYTMADLVYALRHFDEGNCDTLKEILVTYNCCT	4537
MT040336.1	PAVAVHDFFKFRVDGDMVPHISRQRLTKYTMADLVYALRHFDEGNCDTLKEILVTYNCCT	4537
MT040335.1	PAVAVHDFFKFRVDGDMVPHISRQRLTKYTMADLVYALRHFDEGNCDTLKEILVTYNCCT	4537
MT040334.1	PAVAVHDFFKFRVDGDMVPHISRQRLTKYTMADLVYALRHFDEGNCDTLKEILVTYNCCT	4537
MT040333.1	PAVAVHDFFKFRVDGDMVPHISRQRLTKYTMADLVYALRHFDEGNCDTLKEILVTYNCCT	4537
MN996532.2	PAVAKHDFFKFRIDGDMVPHISRQRLTKYTMADLVYALRHFDEGNCDTLREILVTYNCCD	4544
MN988713.1	PAVAKHDFFKFRIDGDMVPHISRQRLTKYTMADLVYALRHFDEGNCDTLKEILVTYNCCD	4545
MT093571.1	PAVAKHDFFKFRIDGDMVPHISRQRLTKYTMADLVYALRHFDEGNCDTLKEILVTYNCCD	4545
MN996529.1	PAVAKHDFFKFRIDGDMVPHISRQRLTKYTMADLVYALRHFDEGNCDTLKEILVTYNCCD	4545
MT072688.1	PAVAKHDFFKFRIDGDMVPHISRQRLTKYTMADLVYALRHFDEGNCDTLKEILVTYNCCD	4545
**NC_045512.2**	**PAVAKHDFFKFRIDGDMVPHISRQRLTKYTMADLVYALRHFDEGNCDTLKEILVTYNCCD**	**4545**
MN994467.1	PAVAKHDFFKFRIDGDMVPHISRQRLTKYTMADLVYALRHFDEGNCDTLKEILVTYNCCD	4545
MG772933.1	PAVAVHDFFKFRVDGDMVPHISRQRLTKYTMADLVYALRHFDEGNCDTLKEILVTYKCCD	4541
MG772934.1	PAVAVHDFFKFRVDGDMVPHISRQRLTKYTMADLVYALRHFDEGNCDTLKEILVTYNCCD	4519
NC_014470.1	PAVAVHDFFKFRVDGDMVPHISRQRLTKYTMADLVYALRHFDEGNCDTLKEILVTYNCCD	4513
GQ153542.1	PAVAVHDFFKFRVDGDMVPHISRQRLTKYTMADLVYALRHFDEGNCDTLKEILVTYNCCD	4520
DQ022305	PAVAVHDFFKFRVDGDMVPHISRQRLTKYTMADLVYALRHFDEGNCDTLKEILVTYNCCD	4516
KF569996.1	PAVAVHDFFKFRVDGDMVPHISRQRLTKYTMADLVYALRHFDEGNCDTLKEILVTYNCCD	4459
KP886809.1	PAVAVHDFFKFRVDGDMVPHISRQRLTKYTMADLVYALRHFDEGNCDTLKEILVTYNCCD	4522
AY278488.2	PAVAVHDFFKFRVDGDMVPHISRQRLTKYTMADLVYALRHFDEGNCDTLKEILVTYNCCD	4522
AY485277.1	PAVAVHDFFKFRVDGDMVPHISRQRLTKYTMADLVYALRHFDEGNCDTLKEILVTYNCCD	4522
AP006560.1	PAVAVHDFFKFRVDGDMVPHISRQRLTKYTMADLVYALRHFDEGNCDTLKEILVTYNCCD	4522
AP006557.1	PAVAVHDFFKFRVDGDMVPHISRQRLTKYTMADLVYALRHFDEGNCDTLKEILVTYNCCD	4522
AY274119	PAVAVHDFFKFRVDGDMVPHISRQRLTKYTMADLVYALRHFDEGNCDTLKEILVTYNCCD	4522
AY572038.1	PAVAVHDFFKFRVDGDMVPHISRQRLTKYTMADLVYALRHFDEGNCDTLKEILVTYNCCD	4522
AY572034.1	PAVAVHDFFKFRVDGDMVPHISRQRLTKYTMADLVYALRHFDEGNCDTLKEILVTYNCCD	4522
FJ588686.1	PAVAVHDFFKFRVDGDMVPHISRQRLTKYTMADLVYALRHFDEGNCDTLKEILVTYNCCD	4329
KY417145.1	PAVAVHDFFKFRVDGDMVPHISRQRLTKYTMADLVYALRHFDEGNCDTLKEILVTYNCCD	4522
KY417144.1	PAVAVHDFFKFRVDGDMVPHISRQRLTKYTMADLVYALRHFDEGNCDTLKEILVTYNCCD	4522
KY417147.1	PAVAVHDFFKFRVDGDMVPHISRQRLTKYTMADLVYALRHFDEGNCDTLKEILVTYNCCD	4522
KY417148.1	PAVAVHDFFKFRVDGDMVPHISRQRLTKYTMADLVYALRHFDEGNCDTLKEILVTYNCCD	4522
KY417143.1	PAVAVHDFFKFRVDGDMVPHISRQRLTKYTMADLVYALRHFDEGNCDTLKEILVTYNCCD	4522
KT444582.1	PAVAVHDFFKFRVDGDMVPHISRQRLTKYTMADLVYALRHFDEGNCDTLKEILVTYNCCD	4522
KC881005.1	PAVAVHDFFKFRVDGDMVPHISRQRLTKYTMADLVYALRHFDEGNCDTLKEILVTYNCCD	4522
KC881006.1	PAVAVHDFFKFRVDGDMVPHISRQRLTKYTMADLVYALRHFDEGNCDTLKEILVTYNCCD	4522
KF367457.1	PAVAVHDFFKFRVDGDMVPHISRQRLTKYTMADLVYALRHFDEGNCDTLKEILVTYNCCD	4522
KY417152.1	PAVAVHDFFKFRVDGDMVPHISRQRLTKYTMADLVYALRHFDEGNCDTLKEILVTYNCCD	4522
KY417146.1	PAVAVHDFFKFRVDGDMVPHISRQRLTKYTMADLVYALRHFDEGNCDTLKEILVTYNCCD	4522
KY417151.1	PSVAVHDFFKFRVDGDMVPHISRQRLTKYTMADLVYALRHFDEGNCDTLKEILVTYNCCD	4522
KY417142.1	PAVAVHDFFKFRVDGDMVPHISRQRLTKYTMADLVYALRHFDEGNCDTLKEILVTYNCCD	4522
	*:** *******:************************************:******:**	

```
MW532698.1      DDYFNKKDWYDFVENPDILRVYANLGERVRQALLKTVQFCDAMRDAGIVGVLTLDNQDLN    4597
MT040336.1      DDYFNKKDWYDFVENPDILRVYANLGERVRQALLKTVQFCDAMRDAGIVGVLTLDNQDLN    4597
MT040335.1      DDYFNKKDWYDFVENPDILRVYANLGERVRQALLKTVQFCDAMRDAGIVGVLTLDNQDLN    4597
MT040334.1      DDYFNKKDWYDFVENPDILRVYANLGERVRQALLKTVQFCDAMRDAGIVGVLTLDNQDLN    4597
MT040333.1      DDYFNKKDWYDFVENPDILRVYANLGERVRQALLKTVQFCDAMRDAGIVGVLTLDNQDLN    4597
MN996532.2      DDYFNKKDWYDFVENPDILRVYANLGERVRQALLKTVQFCDAMRDAGIVGVLTLDNQDLN    4604
MN988713.1      DDYFNKKDWYDFVENPDILRVYANLGERVRQALLKTVQFCDAMRNAGIVGVLTLDNQDLN    4605
MT093571.1      DDYFNKKDWYDFVENPDILRVYANLGERVRQALLKTVQFCDAMRNAGIVGVLTLDNQDLN    4605
MN996529.1      DDYFNKKDWYDFVENPDILRVYANLGERVRQALLKTVQFCDAMRNAGIVGVLTLDNQDLN    4605
MT072688.1      DDYFNKKDWYDFVENPDILRVYANLGERVRQALLKTVQFCDAMRNAGIVGVLTLDNQDLN    4605
NC_045512.2     DDYFNKKDWYDFVENPDILRVYANLGERVRQALLKTVQFCDAMRNAGIVGVLTLDNQDLN    4605
MN994467.1      DDYFNKKDWYDFVENPDILRVYANLGERVRQALLKTVQFCDAMRNAGIVGVLTLDNQDLN    4605
MG772933.1      DNYFNKKDWYDFVENPDVLRVYANLGERVRQALLKTVQFCDAMRDAGIVGVLTLDNQDLN    4601
MG772934.1      GDYFNKKDWYDFVENPDILRVYANLGERVRQALLKTVQFCDAMRDAGIVGVLTLDNQDLN    4579
NC_014470.1     DAYFNKKDWYDFVENPDILRVYACLGERVRQALLKTVQFCDAMRDAGIVGVLTLDNQDLN    4573
GQ153542.1      DNYFNKKDWYDFVENPDVLRVYANLGERVRQALLKTVQFCDAMRDAGIVGVLTLDNQDLN    4580
DQ022305        DNYFNKKDWYDFVENPDVLRVYANLGERVRRALLKTVQFCDAMRDAGIVGVLTLDNQDLN    4576
KF569996.1      DDYFNKKDWYDFVENPDILRVYANLGERVRQALLKTVQFCDAMRDAGIVGVLTLDNQDLN    4519
KP886809.1      DDYFNKKDWYDFVENPDILRVYANLGERVRQALLKTVQFCDAMRDAGIVGVLTLDNQDLN    4582
AY278488.2      DDYFNKKDWYDFVENPDILRVYANLGERVRQSLLKTVQFCDAMRDAGIVGVLTLDNQDLN    4582
AY485277.1      DDYFNKKDWYDFVENPDILRVYANLGERVRQSLLKTVQFCDAMRDAGIVGVLTLDNQDLN    4582
AP006560.1      DDYFNKKDWYDFVENPDILRVYANLGERVRQSLLKTVQFCDAMRDAGIVGVLTLDNQDLN    4582
AP006557.1      DDYFNKKDWYDFVENPDILRVYANLGERVRQSLLKTVQFCDAMRDAGIVGVLTLDNQDLN    4582
AY274119        DDYFNKKDWYDFVENPDILRVYANLGERVRQSLLKTVQFCDAMRDAGIVGVLTLDNQDLN    4582
AY572038.1      DDYFNKKDWYDFVENPDILRVYANLGERVRQSLLKTVQFCDAMRDAGIVGVLTLDNQDLN    4582
AY572034.1      DDYFNKKDWYDFVENPDILRVYANLGERVRQSLLKTVQFCDAMRDAGIVGVLTLDNQDLN    4582
FJ588686.1      DDYFNKKDWYDFVENPDILRVYANLGERVRQALLKTVQFCDAMRDAGIVGVLTLDNQDLN    4389
KY417145.1      DDYFNKKDWYDFVENPDILRVYANLGERVRQALLKTVQFCDAMRDAGIVGVLTLDNQDLN    4582
KY417144.1      DDYFNKKDWYDFVENPDILRVYANLGERVRQALLKTVQFCDAMRDAGIVGVLTLDNQDLN    4582
KY417147.1      DDYFNKKDWYDFVENPDILRVYANLGERVRQALLKTVQFCDAMRNAGIVGVLTLDNQDLN    4582
KY417148.1      DDYFNKKDWYDFVENPDILRVYANLGERVRQALLKTVQFCDAMRNAGIVGVLTLDNQDLN    4582
KY417143.1      DDYFNKKDWYDFVENPDILRVYANLGERVRQALLKTVQFCDAMRDAGIVGVLTLDNQDLN    4582
KT444582.1      DDYFNKKDWYDFVENPDILRVYANLGERVRQALLKTVQFCDAMRDAGIVGVLTLDNQDLN    4582
KC881005.1      DDYFNKKDWYDFVENPDILRVYANLGERVRQALLKTVQFCDAMRDAGIVGVLTLDNQDLN    4582
KC881006.1      DDYFNKKDWYDFVENPDILRVYANLGERVRQALLKTVQFCDAMRDAGIVGVLTLDNQDLN    4582
KF367457.1      DDYYNKKDWYDFVENPDILRVYANLGERVRQALLKTVQFCDAMRDAGIVGVLTLDNQDLN    4582
KY417152.1      DDYFNKKDWYDFVENPDILRVYANLGERVRQALLKTVQFCDAMRDAGIVGVLTLDNQDLN    4582
KY417146.1      DDYFNKKDWYDFVENPDILRVYANLGERVRQALLKTVQFCDAMRDAGIVGVLTLDNQDLN    4582
KY417151.1      DDYFNKKDWYDFVENPDILRVYANLGERVRQALLKTVQFCDAMRDAGIVGVLTLDNQDLN    4582
KY417142.1      DDYFNKKDWYDFVENPDILRVYANLGERVRQALLKTVQFCDAMRDAGIVGVLTLDNQDLN    4582
                . *:*************:***** ******::************:***************
```

CoVAX_ORF1ab#10

```
MW532698.1      GNWYDFGDFIQTTPGSGVPIVDSYYSLLMPILTLTRALAAESHLDADLTKPYVKWDLLKY      4657
MT040336.1      GNWYDFGDFIQTTPGSGVPIVDSYYSLLMPILTLTRALAAESHLDADLTKPYVKWDLLKY      4657
MT040335.1      GNWYDFGDFIQTTPGSGVPIVDSYYSLLMPILTLTRALAAESHLDADLTKPYVKWDLLKY      4657
MT040334.1      GNWYDFGDFIQTTPGSGVPIVDSYYSLLMPILTLTRALAAESHLDADLTKPYVKWDLLKY      4657
MT040333.1      GNWYDFGDFIQTTPGSGVPIVDSYYSLLMPILTLTRALAAESHLDADLTKPYVKWDLLKY      4657
MN996532.2      GNWYDFGDFIQTTPGSGVPIVDSYYSLLMPILTLTRALTAESHVDTDLTKPYIKWDLLKY      4664
MN988713.1      GNWYDFGDFIQTTPGSGVPVVDSYYSLLMPILTLTRALTAESHVDTDLTKPYIKWDLLKY      4665
MT093571.1      GNWYDFGDFIQTTPGSGVPVVDSYYSLLMPILTLTRALTAESHVDTDLTKPYIKWDLLKY      4665
MN996529.1      GNWYDFGDFIQTTPGSGVPVVDSYYSLLMPILTLTRALTAESHVDTDLTKPYIKWDLLKY      4665
MT072688.1      GNWYDFGDFIQTTPGSGVPVVDSYYSLLMPILTLTRALTAESHVDTDLTKPYIKWDLLKY      4665
NC_045512.2     GNWYDFGDFIQTTPGSGVPVVDSYYSLLMPILTLTRALTAESHVDTDLTKPYIKWDLLKY      4665
MN994467.1      GNWYDFGDFIQTTPGSGVPVVDSYYSLLMPILTLTRALTAESHVDTDLTKPYIKWDLLKY      4665
MG772933.1      GNWYDFGDFVQVAPGCGVPIVDSYYSLLMPILTLTRALAAESHMDADLAKPLIKWDLLKY      4661
MG772934.1      GNWYDFGDFVQVAPGCGVPIVDSYYSLLMPILTLTRALAAESHMDADLAKPLIKWDLLKY      4639
NC_014470.1     GNWYDFGDFVQVAPGAGIPIVDSYYSLLMPILTLTKALAAESHMDCDTTKPLIKWDLLKY      4633
GQ153542.1      GNWYDFGDFVQVAPGCGVPIVDSYYSLLMPILTLTKALAAESHMDADLAKPLVKWDLLKY      4640
DQ022305        GNWYDFGDFVQVAPGCGVPIVDSYYSLLMPILTLTKALAAESHMDADLAKPLVKWDLLKY      4636
KF569996.1      GNWYDFGDFVQVAPGCGVPIVDSYYSLLMPILTLTRALAAESHMDADLAKPLIKWDLLKY      4579
KP886809.1      GNWYDFGDFVQVAPGCGVPIVDSYYSLLMPILTMTRALAAESHMDADLAKPLIKWDLLKY      4642
AY278488.2      GNWYDFGDFVQVAPGCGVPIVDSYYSLLMPILTLTRALAAESHMDADLAKPLIKWDLLKY      4642
AY485277.1      GNWYDFGDFVQVAPGCGVPIVDSYYSLLMPILTLTRALAAESHMDADLAKPLIKWDLLKY      4642
AP006560.1      GNWYDFGDFVQVAPGCGVPIVDSYYSLLMPILTLTRALAAESHMDADLAKPLIKWDLLKY      4642
AP006557.1      GNWYDFGDFVQVAPGCGVPIVDSYYSLLMPILTLTRALAAESHMDADLAKPLIKWDLLKY      4642
AY274119        GNWYDFGDFVQVAPGCGVPIVDSYYSLLMPILTLTRALAAESHMDADLAKPLIKWDLLKY      4642
AY572038.1      GNWYDFGDFVQVAPGCGVPIVDSYYSLLMPILTLTRALAAESHMDADLAKPLIKWDLLKY      4642
AY572034.1      GNWYDFGDFVQVAPGCGVPIVDSYYSLLMPILTLTRALAAESHMDADLAKPLIKWDLLKY      4642
FJ588686.1      GNWYDFGDFVQVAPGCGVPIVDSYYSLLMPILTLTRALAAESHMDADLAKPLIKWDLLKY      4449
KY417145.1      GNWYDFGDFVQVAPGCGVPIVDSYYSLLMPILTLTRALAAESHMDADLAKPLIKWDLLKY      4642
KY417144.1      GNWYDFGDFVQVAPGCGVPIVDSYYSLLMPILTLTRALAAESHMDADLAKPLIKWDLLKY      4642
KY417147.1      GNWYDFGDFVQVAPGCGVPIVDSYYSLLMPILTLTRALAAESHMDADLAKPLIKWDLLKY      4642
KY417148.1      GNWYDFGDFVQVAPGCGVPIVDSYYSLLMPILTLTRALAAESHMDADLAKPLIKWDLLKY      4642
KY417143.1      GNWYDFGDFVQVAPGCGVPIVDSYYSLLMPILTLTRALAAESHMDADLAKPLIKWDLLKY      4642
KT444582.1      GNWYDFGDFVQVAQGCGVPIVDSYYSLLMPILTLTRALAAESHMDADFAKPLIKWDLLKY      4642
KC881005.1      GNWYDFGDFVQVAPGCGVPIVDSYYSLLMPILTLTRALAAESHMDADLAKPLIKWDLLKY      4642
KC881006.1      GNWYDFGDFVQVAPGCGVPIVDSYYSLLMPILTLTRALAAESHMDADLAKPLIKWDLLKY      4642
KF367457.1      GNWYDFGDFVQVAPGCGVPIVDSYYSLLMPILTLTRALAAESHMDADLAKPLIKWDLLKY      4642
KY417152.1      GNWYDFGDFVQVAPGCGVPIVDSYYSLLMPILTLTRALAAESHMDADLAKPLIKWDLLKY      4642
KY417146.1      GNWYDFGDFVQVAPGCGVPIVDSYYSLLMPILTLTRALAAESHMDADLAKPLIKWDLLKY      4642
KY417151.1      GNWYDFGDFVQVAPGCGVPIVDSYYSLLMPILTLTRALAAESHMDADLAKPLIKWDLLKY      4642
KY417142.1      GNWYDFGDFVQVAPGCGVPIVDSYYSLLMPILTLTRALAAESHMDADLAKPLIKWDLLKY      4642
                *********:*.: *.*:*:*************:*:**:****:* * :** :*******
```

CoVAX_ORF1ab#10

```
MW532698.1      DFTEERLNLFNRYFKYWDQTYHPNCVNCLDDRCILHCANFNVLFSTVFPPTSFGPLVRKI   4717
MT040336.1      DFTEERLNLFNRYFKYWDQTYHPNCVNCLDDRCILHCANFNVLFSTVFPPTSFGPLVRKI   4717
MT040335.1      DFTEERLNLFNRYFKYWDQTYHPNCVNCLDDRCILHCANFNVLFSTVFPPTSFGPLVRKI   4717
MT040334.1      DFTEERLNLFNRYFKYWDQTYHPNCVNCLDDRCILHCANFNVLFSTVFPPTSFGPLVRKI   4717
MT040333.1      DFTEERLNLFNRYFKYWDQTYHPNCVNCLDDRCILHCANFNVLFSTVFPPTSFGPLVRKI   4717
MN996532.2      DFTEERLKLFDRYFKYWDQTYHPNCVNCLDDRCILHCANFNVLFSTVFPPTSFGPLVRKI   4724
MN988713.1      DFTEERLKLFDRYFKYWDQTYHPNCVNCLDDRCILHCANFNVLFSTVFPPTSFGPLVRKI   4725
MT093571.1      DFTEERLKLFDRYFKYWDQTYHPNCVNCLDDRCILHCANFNVLFSTVFPPTSFGPLVRKI   4725
MN996529.1      DFTEERLKLFDRYFKYWDQTYHPNCVNCLDDRCILHCANFNVLFSTVFPPTSFGPLVRKI   4725
MT072688.1      DFTEERLKLFDRYFKYWDQTYHPNCVNCLDDRCILHCANFNVLFSTVFPPTSFGPLVRKI   4725
NC_045512.2     DFTEERLKLFDRYFKYWDQTYHPNCVNCLDDRCILHCANFNVLFSTVFPPTSFGPLVRKI   4725
MN994467.1      DFTEERLKLFDRYFKYWDQTYHPNCVNCLDDRCILHCANFNVLFSTVFPPTSFGPLVRKI   4725
MG772933.1      DFTEERLCLFDRYFKYWDQTYHPNCINCLDDRCILHCANFNVLFSTVFPPTSFGPLVRKI   4721
MG772934.1      DFTEERLCLFDRYFKYWDQTYHPNCINCLDDRCILHCANFNVLFSTVFPPTSFGPLVRKI   4699
NC_014470.1     DFTEERLCLFNRYFKYWDQTYHPNCINCLDDRCILHCANFNVLFSTVFPPTSFGPLVRKI   4693
GQ153542.1      DFTEERLCLFDRYFKYWDQIYHPNCINCLDDRCILHCANFNVLFSTVFPPTSFGPLVRKI   4700
DQ022305        DFTEERLCLFDRYFKYWDQTYHPNCINCLDDRCILHCANFNVLFSTVFPPTSFGPLVRKI   4696
KF569996.1      DFTEERLCLFDRYFKYWDQIYHPNCINCLDDRCILHCANFNVLFSTVFPPTSFGPLVRKI   4639
KP886809.1      DFTEERLCLFDRYFKYWDQTYHPNCINCLDDRCILHCANFNVLFSTVFPPTSFGPLVRKI   4702
AY278488.2      DFTEERLCLFDRYFKYWDQTYHPNCINCLDDRCILHCANFNVLFSTVFPPTSFGPLVRKI   4702
AY485277.1      DFTEERLCLFDRYFKYWDQTYHPNCINCLDDRCILHCANFNVLFSTVFPPTSFGPLVRKI   4702
AP006560.1      DFTEERLCLFDRYFKYWDQTYHPNCINCLDDRCILHCANFNVLFSTVFPPTSFGPLVRKI   4702
AP006557.1      DFTEERLCLFDRYFKYWDQTYHPNCINCLDDRCILHCANFNVLFSTVFPPTSFGPLVRKI   4702
AY274119        DFTEERLCLFDRYFKYWDQTYHPNCINCLDDRCILHCANFNVLFSTVFPPTSFGPLVRKI   4702
AY572038.1      DFTEERLCLFDRYFKYWDQTYHPNCINCLDDRCILHCANFNVLFSTVFPPTSFGPLVRKI   4702
AY572034.1      DFTEERLCLFDRYFKYWDQTYHPNCINCLDDRCILHCANFNVLFSTVFPPTSFGPLVRKI   4702
FJ588686.1      DFTEERLCLFDRYFKYWDQTYHPNCINCLDDRCILHCANFNVLFSTVFPPTSFGPLVRKI   4509
KY417145.1      DFTEERLCLFDRYFKYWDQTYHPNCINCLDDRCILHCANFNVLFSTVFPPTSFGPLVRKI   4702
KY417144.1      DFTEERLCLFDRYFKYWDQTYHPNCINCLDDRCILHCANFNVLFFTVFPPTSFGPLVRKI   4702
KY417147.1      DFTEERLCLFDRYFKYWDQTYHPNCINCLDDRCILHCANFNVLFSTVFPPTSFGPLVRKI   4702
KY417148.1      DFTEERLCLFDRYFKYWDQTYHPNCINCLDDRCILHCANFNVLFSTVFPPTSFGPLVRKI   4702
KY417143.1      DFTEERLCLFDRYFKYWDQTYHPNCINCLDDRCILHCANFNVLFSTVFPPTSFGPLVRKI   4702
KT444582.1      DFTEERLCLFDRYFKYWDQTYHPNCINCLDDRCILHCANFNVLFSTVFPPTSFGPLVRKI   4702
KC881005.1      DFTEERLCLFDRYFKYWDQTYHPNCINCLDDRCILHCANFNVLFSTVFPPTSFGPLVRKI   4702
KC881006.1      DFTEERLCLFDRYFKYWDQTYHPNCINCLDDRCILHCANFNVLFSTVFPPTSFGPLVRKI   4702
KF367457.1      DFTEERLCLFDRYFKYWDQTYHPNCINCLDDRCILHCANFNVLFSTVFPPTSFGPLVRKI   4702
KY417152.1      DFTEERLCLFDRYFKYWDQTYHPNCINCLDDRCILHCANFNVLFFTVFPPTSFGPLVRKI   4702
KY417146.1      DFTEERLCLFDRYFKYWDQTYHPNCINCLDDRCILHCANFNVLFSTVFPPTSFGPLVRKI   4702
KY417151.1      DFTEERLCLFDRYFKYWDQTYHPNCINCLDDRCILHCANFNVLFSTVFPPTSFGPLVRKI   4702
KY417142.1      DFMEERLCLFDRYFKYWDQTYHPNCINCLDDRCILHCANFNVLFSTVFPPTSFGPLVRKI   4702
                ** **** **:******** *****:****************** ***************
```

CoVAX_ORF1ab#16

```
MW532698.1      FVDGVPFVVSTGYHFRELGVVHNQDVNIHSSRLSFKELLVYAADPAMHAASGNLLLDKRT    4777
MT040336.1      FVDGVPFVVSTGYHFRELGVVHNQDVNIHSSRLSFKELLVYAADPAMHAASGNLLLDKRT    4777
MT040335.1      FVDGVPFVVSTGYHFRELGVVHNQDVNIHSSRLSFKELLVYAADPAMHAASGNLLLDKRT    4777
MT040334.1      FVDGVPFVVSTGYHFRELGVVHNQDVNIHSSRLSFKELLVYAADPAMHAASGNLLLDKRT    4777
MT040333.1      FVDGVPFVVSTGYHFRELGVVHNQDVNIHSSRLSFKELLVYAADPAMHAASGNLLLDKRT    4777
MN996532.2      FVDGVPFVVSTGYHFRELGVVHNQDVNLHSSRLSFKELLVYAADPAMHAASGNLLLDKRT    4784
MN988713.1      FVDGVPFVVSTGYHFRELGVVHNQDVNLHSSRLSFKELLVYAADPAMHAASGNLLLDKRT    4785
MT093571.1      FVDGVPFVVSTGYHFRELGVVHNQDVNLHSSRLSFKELLVYAADPAMHAASGNLLLDKRT    4785
MN996529.1      FVDGVPFVVSTGYHFRELGVVHNQDVNLHSSRLSFKELLVYAADPAMHAASGNLLLDKRT    4785
MT072688.1      FVDGVPFVVSTGYHFRELGVVHNQDVNLHSSRLSFKELLVYAADPAMHAASGNLLLDKRT    4785
NC_045512.2     FVDGVPFVVSTGYHFRELGVVHNQDVNLHSSRLSFKELLVYAADPAMHAASGNLLLDKRT    4785
MN994467.1      FVDGVPFVVSTGYHFRELGVVHNQDVNLHSSRLSFKELLVYAADPAMHAASGNLLLDKRT    4785
MG772933.1      FVDGVPFVVSTGYHFRELGVVHNQDVNLHNSRLSFKELLVYAADPAMHAASGNLLLDKRT    4781
MG772934.1      FVDGVPFVVSTGYHFRELGVVHNQDVNLHSSRLSFKELLVYAADPAMHAASGNLLLDKRT    4759
NC_014470.1     FVDGVPFVVSTGYHFRELGVVHNQDVNLHSSRLSFKELLVYAADPAMHAASGNLLLDKRT    4753
GQ153542.1      FVDGVPFVVSTGYHFRELGVVHNQDVNLHSSRLSFKELLVYAADPAMHAASGNLLLDKRT    4760
DQ022305        FVDGVPFVVSTGYHFRELGVVHNQDVNLHSSRLSFKELLVYAADPAMHAASGNLLLDKRT    4756
KF569996.1      FVDGVPFVVSTGYHFRELGVVHNQDVNLHSSRLSFKELLVYAADPAMHAASGNLLLDKRT    4699
KP886809.1      FVDGVPFVVSTGYHFRELGVVHNQDVNLHSSRLSFKELLVYAADPAMHAASGNLLLDKRT    4762
AY278488.2      FVDGVPFVVSTGYHFRELGVVHNQDVNLHSSRLSFKELLVYAADPAMHAASGNLLLDKRT    4762
AY485277.1      FVDGVPFVVSTGYHFRELGVVHNQDVNLHSSRLSFKELLVYAADPAMHAASGNLLLDKRT    4762
AP006560.1      FVDGVPFVVSTGYHFRELGVVHNQDVNLHSSRLSFKELLVYAADPAMHAASGNLLLDKRT    4762
AP006557.1      FVDGVPFVVSTGYHFRELGVVHNQDVNLHSSRLSFKELLVYAADPAMHAASGNLLLDKRT    4762
AY274119        FVDGVPFVVSTGYHFRELGVVHNQDVNLHSSRLSFKELLVYAADPAMHAASGNLLLDKRT    4762
AY572038.1      FVDGVPFVVSTGYHFRELGVVHNQDVNLHSSRLSFKELLVYAADPAMHAASGNLLLDKRT    4762
AY572034.1      FVDGVPFVVSTGYHFRELGVVHNQDVNLHSSRLSFKELLVYAADPAMHAASGNLLLDKRT    4762
FJ588686.1      FVDGVPFVVSTGYHFRELGVVHNQDVNLHSSRLSFKELLVYAADPAMHAASGNLLLDKRT    4569
KY417145.1      FVDGVPFVVSTGYHFRELGVVHNQDVNLHSSRLSFKELLVYAADPAMHAASGNLLLDKRT    4762
KY417144.1      FVDGVPFVVSTGYHFRELGVVHNQDVNLHSSRLSFKELLVYAADPAMHAASGNLLLDKRT    4762
KY417147.1      FVDGVPFVVSTGYHFRELGVVHNQDVNLHSSRLSFKELLVYAADPAMHAASGNLLLDKRT    4762
KY417148.1      FVDGVPFVVSTGYHFRELGVVHNQDVNLHSSRLSFKELLVYAADPAMHAASGNLLLDKRT    4762
KY417143.1      FVDGVPFVVSTGYHFRELGVVHNQDVNLHSSRLSFKELLVYAADPAMHAASGNLLLDKRT    4762
KT444582.1      FVDGVPFVVSTGYHFRELGVVHNQDVNLHSSRLSFKELLVYAADPAMHAASGNLLLDKRT    4762
KC881005.1      FVDGVPFVVSTGYHFRELGVVHNQDVNLHSSRLSFKELLVYAADPAMHAASGNLLLDKRT    4762
KC881006.1      FVDGVPFVVSTGYHFRELGVVHNQDVNLHSSRLSFKELLVYAADPAMHAASGNLLLDKRT    4762
KF367457.1      FVDGVPFVVSTGYHFRELGVVHNQDVNLHSSRLSFKELLVYAADPAMHAASGNLLLDKRT    4762
KY417152.1      FVDGVPFVVSTGYHFRELGVVHNQDVNLHSSRLSFKELLVYAADPAMHAASGNLLLDKRT    4762
KY417146.1      FVDGVPFVVSTGYHFRELGVVHNQDVNLHSSRLSFKELLVYAADPAMHAASGNLLLDKRT    4762
KY417151.1      FVDGVPFVVSTGYHFRELGVVHNQDVNLHSSRLSFKELLVYAADPAMHAASGNLLLDKRT    4762
KY417142.1      FVDGVPFVVSTGYHFRELGVVHNQDVNLHSSRLSLKELLVYAADPAMHAASGNLLLDKRT    4762
                ***************************:*.****:*************************
```

CoVAX_ORF1ab#16

```
MW532698.1      TCFSVAALTNNVAFQTVKPGNFNKDFYDFAVSKGFFKEGSSVELKHFFFAQDGNAAISDY      4837
MT040336.1      TCFSVAALTNNVAFQTVKPGNFNKDFYDFAVSKGFFKEGSSVELKHFFFAQDGNAAISDY      4837
MT040335.1      TCFSVAALTNNVAFQTVKPGNFNKDFYDFAVSKGFFKEGSSVELKHFFFAQDGNAAISDY      4837
MT040334.1      TCFSVAALTNNVAFQTVKPGNFNKDFYDFAVSKGFFKEGSSVELKHFFFAQDGNAAISDY      4837
MT040333.1      TCFSVAALTNNVAFQTVKPGNFNKDFYDFAVSKGFFKEGSSVELKHFFFAQDGNAAISDY      4837
MN996532.2      TCFSVAALTNNVAFQTVKPGNFNKDFYDFAVSKGFFKEGSSVELKHFFFAQDGNAAISDY      4844
MN988713.1      TCFSVAALTNNVAFQTVKPGNFNKDFYDFAVSKGFFKEGSSVELKHFFFAQDGNAAISDY      4845
MT093571.1      TCFSVAALTNNVAFQTVKPGNFNKDFYDFAVSKGFFKEGSSVELKHFFFAQDGNAAISDY      4845
MN996529.1      TCFSVAALTNNVAFQTVKPGNFNKDFYDFAVSKGFFKEGSSVELKHFFFAQDGNAAISDY      4845
MT072688.1      TCFSVAALTNNVAFQTVKPGNFNKDFYDFAVSKGFFKEGSSVELKHFFFAQDGNAAISDY      4845
NC_045512.2     TCFSVAALTNNVAFQTVKPGNFNKDFYDFAVSKGFFKEGSSVELKHFFFAQDGNAAISDY      4845
MN994467.1      TCFSVAALTNNVAFQTVKPGNFNKDFYDFAVSKGFFKEGSSVELKHFFFAQDGNAAISDY      4845
MG772933.1      TCFSVAALTNNVAFQTVKPGNFNKDFYDFAVSKGFFKEGSSVELKHFFFAQDGNAAISDY      4841
MG772934.1      TCFSVAALTNNVAFQTVKPGNFNKDFYDFAVSKGFFKEGSSVELKHFFFAQDGNAAISDY      4819
NC_014470.1     TCFSVAALTNSVAFQTVKPGNFNKDFYDFAVSKGFFKEGSSVELKHFFFAQDGNAAISDY      4813
GQ153542.1      TCFSVAALTNNVAFQTVKPGNFNKDFYDFAVSKGFFKEGSSVELKHFFFAQDGNAAISDY      4820
DQ022305        TCFSVAALTNNVAFQTVKPGNFNKDFYDFAVSKGFFKEGSSVELKHFFFAQDGNAAISDY      4816
KF569996.1      TCFSVAALTNNVAFQTVKPGNFNKDFYDFAVSKGFFKEGSSVELKHFFFAQDGNAAISDY      4759
KP886809.1      TCFSVAALTNNVAFQTVKPGNFNKDFYDFAVSKGFFKEGSSVELKHFFFAQDGNAAISDY      4822
AY278488.2      TCFSVAALTNNVAFQTVKPGNFNKDFYDFAVSKGFFKEGSSVELKHFFFAQDGNAAISDY      4822
AY485277.1      TCFSVAALTNNVAFQTVKPGNFNKDFYDFAVSKGFFKEGSSVELKHFFFAQDGNAAISDY      4822
AP006560.1      TCFSVAALTNNVAFQTVKPGNFNKDFYDFAVSKGFFKEGSSVELKHFFFAQDGNAAISDY      4822
AP006557.1      TCFSVAALTNNVAFQTVKPGNFNKDFYDFAVSKGFFKEGSSVELKHFFFAQDGNAAISDY      4822
AY274119        TCFSVAALTNNVAFQTVKPGNFNKDFYDFAVSKGFFKEGSSVELKHFFFAQDGNAAISDY      4822
AY572038.1      TCFSVAALTNNVAFQTVKPGNFNKDFYDFAVSKGFFKEGSSVELKHFFFAQDGNAAISDY      4822
AY572034.1      TCFSVAALTNNVAFQTVKPGNFNKDFYDFAVSKGFFKEGSSVELKHFFFAQDGNAAISDY      4822
FJ588686.1      TCFSVAALTNNVAFQTVKPGNFNKDFYDFAVSKGFFKEGSSVELKHFFFAQDGNAAISDY      4629
KY417145.1      TCFSVAALTNNVAFQTVKPGNFNKDFYDFAVSKGFFKEGSSVELKHFFFAQDGNAAISDY      4822
KY417144.1      TCFSVAALTNNVAFQTVKPGNFNKDFYDFAVSKGFFKEGSSVELKHFFFAQDGNAAISDY      4822
KY417147.1      TCFSVAALTNNVAFQTVKPGNFNKDFYDFAVSKGFFKEGSSVELKHFFFAQDGNAAISDY      4822
KY417148.1      TCFSVAALTNNVAFQTVKPGNFNKDFYDFAVSKGFFKEGSSVELKHFFFAQDGNAAISDY      4822
KY417143.1      TCFSVAALTNNVAFQTVKPGNFNKDFYDFAVSKGFFKEGSSVELKHFFFAQDGNAAISDY      4822
KT444582.1      TCFSVAALTNNVAFQTVKPGNFNKDFYDFAVSKGFFKEGSSVELKHFFFAQDGNAAISDY      4822
KC881005.1      TCFSVAALTNNVAFQTVKPGNFNKDFYDFAVSKGFFKEGSSVELKHFFFAQDGNAAISDY      4822
KC881006.1      TCFSVAALTNNVAFQTVKPGNFNKDFYDFAVSKGFFKEGSSVELKHFFFAQDGNAAISDY      4822
KF367457.1      TCFSVAALTNNVAFQTVKPGNFNKDFYDFAVSKGFFKEGSSVELKHFFFAQDGNAAISDY      4822
KY417152.1      TCFSVAALTNNVAFQTVKPGNFNKDFYDFAVSKGFFKEGSSVELKHFFFAQDGNAAISDY      4822
KY417146.1      TCFSVAALTNNVAFQTVKPGNFNKDFYDFAVSKGFFKEGSSVELKHFFFAQDGNAAISDY      4822
KY417151.1      TCFSVAALTNNVAFQTVKPGNFNKDFYDFAVSKGFFKEGSSVELKHFFFAQDGNAAISDY      4822
KY417142.1      TCFSVAALTNNVAFQTVKPGNFNKDFYDFAVSKGFFKEGSSVELKHFFFAQDGNAAISDY      4822
                **********.*************************************************
```

CoVAX_ORF1ab#4

```
MW532698.1      DYYRYNLPTMCDIRQLLFVVEVVDKYFDCYDGGCINANQVIVNNLDKSAGFPFNKWGKAR      4897
MT040336.1      DYYRYNLPTMCDIRQLLFVVEVVDKYFDCYDGGCINANQVIVNNLDKSAGFPFNKWGKAR      4897
MT040335.1      DYYRYNLPTMCDIRQLLFVVEVVDKYFDCYDGGCINANQVIVNNLDKSAGFPFNKWGKAR      4897
MT040334.1      DYYRYNLPTMCDIRQLLFVVEVVDKYFDCYDGGCINANQVIVNNLDKSAGFPFNKWGKAR      4897
MT040333.1      DYYRYNLPTMCDIRQLLFVVEVVDKYFDCYDGGCINANQVIVNNLDKSAGFPFNKWGKAR      4897
MN996532.2      DYYRYNLPTMCDIRQLLFVVEVVDKYFDCYDGGCINANQVIVNNLDKSAGFPFNKWGKAR      4904
MN988713.1      DYYRYNLPTMCDIRQLLFVVEVVDKYFDCYDGGCINANQVIVNNLDKSAGFPFNKWGKAR      4905
MT093571.1      DYYRYNLPTMCDIRQLLFVVEVVDKYFDCYDGGCINANQVIVNNLDKSAGFPFNKWGKAR      4905
MN996529.1      DYYRYNLPTMCDIRQLLFVVEVVDKYFDCYDGGCINANQVIVNNLDKSAGFPFNKWGKAR      4905
MT072688.1      DYYRYNLPTMCDIRQLLFVVEVVDKYFDCYDGGCINANQVIVNNLDKSAGFPFNKWGKAR      4905
NC_045512.2     DYYRYNLPTMCDIRQLLFVVEVVDKYFDCYDGGCINANQVIVNNLDKSAGFPFNKWGKAR      4905
MN994467.1      DYYRYNLPTMCDIRQLLFVVEVVDKYFDCYDGGCINANQVIVNNLDKSAGFPFNKWGKAR      4905
MG772933.1      DYYRYNLPTMCDIRQLLFVVEVVDKYFDCYDGGCINANQVIVNNLDKSAGFPFNKWGKAR      4901
MG772934.1      DYYRYNLPTMCDITQLLFVIEVVDKYFDCYDGGCINANQVIVNNLDKSAGFPFNKWGKAR      4879
NC_014470.1     DYYRYNLPTMCDIRQLLFVVEVVDKYFDCYDGGCINANQVIVNNLDKSAGFPFNKWGKAR      4873
GQ153542.1      DYYRYNLPTMCDIRQLLFVVEVVDKYFDCYDGGCINANQVIVNNLDKSAGFPFNKWGKAR      4880
DQ022305        DYYRYNLPTMCDIRQLLFVVEVVDKYFDCYDGGCINANQVIVNNLDKSAGFPFNKWGKAR      4876
KF569996.1      DYYRYNLPTMCDIRQLLFVVEVVDKYFDCYDGGCINANQVIVNNLDKSAGFPFNKWGKAR      4819
KP886809.1      DYYRYNLPTMCDIRQLLFVVEVVDKYFDCYDGGCINANQVIVNNLDKSAGFPFNKWGKAR      4882
AY278488.2      DYYRYNLPTMCDIRQLLFVVEVVDKYFDCYDGGCINANQVIVNNLDKSAGFPFNKWGKAR      4882
AY485277.1      DYYRYNLPTMCDIRQLLFVVEVVDKYFDCYDGGCINANQVIVNNLDKSAGFPFNKWGKAR      4882
AP006560.1      DYYRYNLPTMCDIRQLLFVVEVVDKYFDCYDGGCINANQVIVNNLDKSAGFPFNKWGKAR      4882
AP006557.1      DYYRYNLPTMCDIRQLLFVVEVVDKYFDCYDGGCINANQVIVNNLDKSAGFPFNKWGKAR      4882
AY274119        DYYRYNLPTMCDIRQLLFVVEVVDKYFDCYDGGCINANQVIVNNLDKSAGFPFNKWGKAR      4882
AY572038.1      DYYRYNLPTMCDIRQLLFVVEVVDKYFDCYDGGCINANQVIVNNLDKSAGFPFNKWGKAR      4882
AY572034.1      DYYRYNLPTMCDIRQLLFVVEVVDKYFDCYDGGCINANQVIVNNLDKSAGFPFNKWGKAR      4882
FJ588686.1      DYYRYNLPTMCDIRQLLFVVEVVDKYFDCYDGGCINANQVIVNNLDKSAGFPFNKWGKAR      4689
KY417145.1      DYYRYNLPTMCDIRQLLFVVEVVDKYFDCYDGGCINANQVIVNNLDKSAGFPFNKWGKAR      4882
KY417144.1      DYYRYNLPTMCDIRQLLFVVEVVDKYFDCYDGGCINANQVIVNNLDKSAGFPFNKWGKAR      4882
KY417147.1      DYYRYNLPTMCDIRQLLFVVEVVDKYFDCYDGGCINANQVIVNNLDKSAGFPFNKWGKAR      4882
KY417148.1      DYYRYNLPTMCDIRQLLFVVEVVDKYFDCYDGGCINANQVIVNNLDKSAGFPFNKWGKAR      4882
KY417143.1      DYYRYNLPTMCDIRQLLFVVEVVDKYFDCYDGGCINANQVIVNNLDKSAGFPFNKWGKAR      4882
KT444582.1      DYYRYNLPTMCDIRQLLFVVEVVDKYFDCYDGGCINANQVIVNNLDKSAGFPFNKWGKAR      4882
KC881005.1      DYYRYNLPTMCDIRQLLFVVEVVDKYFDCYDGGCINANQVIVNNLDKSAGFPFNKWGKAR      4882
KC881006.1      DYYRYNLPTMCDIRQLLFVVEVVDKYFDCYDGGCINANQVIVNNLDKSAGFPFNKWGKAR      4882
KF367457.1      DYYRYNLPTMCDIRQLLFVVEVVDKYFDCYDGGCINANQVIVNNLDKSAGFPFNKWGKAR      4882
KY417152.1      DYYRYNLPTMCDIRQLLFVVEVVDKYFDCYDGGCINANQVIVNNLDKSAGFPFNKWGKAR      4882
KY417146.1      DYYRYNLPTMCDIRQLLFVVEVVDKYFDCYDGGCINANQVIVNNLDKSAGFPFNKWGKAR      4882
KY417151.1      DYYRYNLPTMCDIRQLLFVVEVVDKYFDCYDGGCINANQVIVNNLDKSAGFPFNKWGKAR      4882
KY417142.1      DYYRYNLPTMCDIRQLLFVVEVVDKYFDCYDGGCINANQVIVNNLDKSAGFPFNKWGKAR      4882
                ************* *****:****************************************
```

CoVAX_ORF1ab#4 CoVAX_ORF1ab#7

```
MW532698.1      LYYDSMSYEDQDALFAYTKRNVIPTITQMNLKYAISAKNRARTVAGVSICSTMTNRQFHQ    4957
MT040336.1      LYYDSMSYEDQDALFAYTKRNVIPTITQMNLKYAISAKNRARTVAGVSICSTMTNRQFHQ    4957
MT040335.1      LYYDSMSYEDQDALFAYTKRNVIPTITQMNLKYAISAKNRARTVAGVSICSTMTNRQFHQ    4957
MT040334.1      LYYDSMSYEDQDALFAYTKRNVIPTITQMNLKYAISAKNRARTVAGVSICSTMTNRQFHQ    4957
MT040333.1      LYYDSMSYEDQDALFAYTKRNVIPTITQMNLKYAISAKNRARTVAGVSICSTMTNRQFHQ    4957
MN996532.2      LYYDSMSYEDQDALFAYTKRNVIPTITQMNLKYAISAKNRARTVAGVSICSTMTNRQFHQ    4964
MN988713.1      LYYDSMSYEDQDALFAYTKRNVIPTITQMNLKYAISAKNRARTVAGVSICSTMTNRQFHQ    4965
MT093571.1      LYYDSMSYEDQDALFAYTKRNVIPTITQMNLKYAISAKNRARTVAGVSICSTMTNRQFHQ    4965
MN996529.1      LYYDSMSYEDQDALFAYTKRNVIPTITQMNLKYAISAKNRARTVAGVSICSTMTNRQFHQ    4965
MT072688.1      LYYDSMSYEDQDALFAYTKRNVIPTITQMNLKYAISAKNRARTVAGVSICSTMTNRQFHQ    4965
NC_045512.2     LYYDSMSYEDQDALFAYTKRNVIPTITQMNLKYAISAKNRARTVAGVSICSTMTNRQFHQ    4965
MN994467.1      LYYDSMSYEDQDALFAYTKRNVIPTITQMNLKYAISAKNRARTVAGVSICSTMTNRQFHQ    4965
MG772933.1      LYYDSMSYEDQDALFAYTKRNVIPTITQMNLKYAISAKNRARTVAGVSICSTMTNRQFHQ    4961
MG772934.1      LYYDSMSYEDQDALFAYTKRNVIPTITQMNLKYAISAKNRARTVAGVSICSTMTNRQFHQ    4939
NC_014470.1     LYYDSMSYEDQDALFAYTKRNVIPTITQMNLKYAISAKNRARTVAGVSICSTMTNRQFHQ    4933
GQ153542.1      LYYDSMSYEDQDALFAYTKRNVIPTITQMNLKYAISAKNRARTVAGVSICSTMTNRQFHQ    4940
DQ022305        LYYDSMSYEDQDALFAYTKRNVIPTITQMNLKYAISAKNRARTVAGVSICSTMTNRQFHQ    4936
KF569996.1      LYYDSMSYEDQDALFAYTKRNVIPTITQMNLKYAISAKNRARTVAGVSICSTMTNRQFHQ    4879
KP886809.1      LYYDSMSYEDQDALFAYTKRNVIPTITQMNLKYAISAKNRARTVAGVSICSTMTNRQFHQ    4942
AY278488.2      LYYDSMSYEDQDALFAYTKRNVIPTITQMNLKYAISAKNRARTVAGVSICSTMTNRQFHQ    4942
AY485277.1      LYYDSMSYEDQDALFAYTKRNVIPTITQMNLKYAISAKNRARTVAGVSICSTMTNRQFHQ    4942
AP006560.1      LYYDSMSYEDQDALFAYTKRNVIPTITQMNLKYAISAKNRARTVAGVSICSTMTNRQFHQ    4942
AP006557.1      LYYDSMSYEDQDALFAYTKRNVIPTITQMNLKYAISAKNRARTVAGVSICSTMTNRQFHQ    4942
AY274119        LYYDSMSYEDQDALFAYTKRNVIPTITQMNLKYAISAKNRARTVAGVSICSTMTNRQFHQ    4942
AY572038.1      LYYDSMSYEDQDALFAYTKRNVIPTITQMNLKYAISAKNRARTVAGVSICSTMTNRQFHQ    4942
AY572034.1      LYYDSMSYEDQDALFAYTKRNVIPTITQMNLKYAISAKNRARTVAGVSICSTMTNRQFHQ    4942
FJ588686.1      LYYDSMSYEDQDALFAYTKRNVIPTITQMNLKYAISAKNRARTVAGVSICSTMTNRQFHQ    4749
KY417145.1      LYYDSMSYEDQDALFAYTKRNVIPTITQMNLKYAISAKNRARTVAGVSICSTMTNRQFHQ    4942
KY417144.1      LYYDSMSYEDQDALFAYTKRNVIPTITQMNLKYAISAKNRARTVAGVSICSTMTNRQFHQ    4942
KY417147.1      LYYDSMSYEDQDALFAYTKRNVIPTITQMNLKYAISAKNRARTVAGVSICSTMTNRQFHQ    4942
KY417148.1      LYYDSMSYEDQDALFAYTKRNVIPTITQMNLKYAISAKNRARTVAGVSICSTMTNRQFHQ    4942
KY417143.1      LYYDSMSYEDQDALFAYTKRNVIPTITQMNLKYAISAKNRARTVAGVSICSTMTNRQFHQ    4942
KT444582.1      LYYDSMSYEDQDALFAYTKRNVIPTITQMNLKYAISAKNRARTVAGVSICSTMTNRQFHQ    4942
KC881005.1      LYYDSMSYEDQDALFAYTKRNVIPTITQMNLKYAISAKNRARTVAGVSICSTMTNRQFHQ    4942
KC881006.1      LYYDSMSYEDQDALFAYTKRNVIPTITQMNLKYAISAKNRARTVAGVSICSTMTNRQFHQ    4942
KF367457.1      LYYDSMSYEDQDALFAYTKRNVIPTITQMNLKYAISAKNRARTVAGVSICSTMTNRQFHQ    4942
KY417152.1      LYYDSMSYEDQDALFAYTKRNVIPTITQMNLKYAISAKNRARTVAGVSICSTMTNRQFHQ    4942
KY417146.1      LYYDSMSYEDQDALFAYTKRNVIPTITQMNLKYAISAKNRARTVAGVSICSTMTNRQFHQ    4942
KY417151.1      LYYDSMSYEDQDALFAYTKRNVIPTITQMNLKYAISAKNRARTVAGVSICSTMTNRQFHQ    4942
KY417142.1      LYYDSMSYEDQDALFAYTKRNVIPTITQMNLKYAISAKNRARTVAGVSICSTMTNRQFHQ    4942
                ************************************************************
```

CoVAX_ORF1ab#7 CoVAX_ORF1ab#8

```
MW532698.1      KLLKSIAATRGATVVIGTSKFYGGWNNMLKTVYSDVENPHLMGWDYPKCDRAMPNMLRIM   5017
MT040336.1      KLLKSIAATRGATVVIGTSKFYGGWNNMLKTVYSDVENPHLMGWDYPKCDRAMPNMLRIM   5017
MT040335.1      KLLKSIAATRGATVVIGTSKFYGGWNNMLKTVYSDVENPHLMGWDYPKCDRAMPNMLRIM   5017
MT040334.1      KLLKSIAATRGATVVIGTSKFYGGWNNMLKTVYSDVENPHLMGWDYPKCDRAMPNMLRIM   5017
MT040333.1      KLLKSIAATRGATVVIGTSKFYGGWNNMLKTVYSDVENPHLMGWDYPKCDRAMPNMLRIM   5017
MN996532.2      KLLKSIAATRGATVVIGTSKFYGGWHNMLKTVYSDVENPHLMGWDYPKCDRAMPNMLRIM   5024
MN988713.1      KLLKSIAATRGATVVIGTSKFYGGWHNMLKTVYSDVENPHLMGWDYPKCDRAMPNMLRIM   5025
MT093571.1      KLLKSIAATRGATVVIGTSKFYGGWHNMLKTVYSDVENPHLMGWDYPKCDRAMPNMLRIM   5025
MN996529.1      KLLKSIAATRGATVVIGTSKFYGGWHNMLKTVYSDVENPHLMGWDYPKCDRAMPNMLRIM   5025
MT072688.1      KLLKSIAATRGATVVIGTSKFYGGWHNMLKTVYSDVENPHLMGWDYPKCDRAMPNMLRIM   5025
NC_045512.2     KLLKSIAATRGATVVIGTSKFYGGWHNMLKTVYSDVENPHLMGWDYPKCDRAMPNMLRIM   5025
MN994467.1      KLLKSIAATRGATVVIGTSKFYGGWHNMLKTVYSDVENPHLMGWDYPKCDRAMPNMLRIM   5025
MG772933.1      KLLKSIAATRGATVVIGTSKFYGGWHNMLKTVYSDVESPHLMGWDYPKCDRAMPNMLRIM   5021
MG772934.1      KLLKSIAATRGATVVIGTSKFYGGWHNMLKTVYSDVESPHLMGWDYPKCDRAMPNMLRIM   4999
NC_014470.1     KLLKSIAATRGATVVIGTSKFYGGWHNMLKTVYSDVETPNLMGWDYPKCDRAMPNMLRIM   4993
GQ153542.1      KLLKSIAATRGATVVIGTSKFYGGWHNMLKTVYSDVESPHLMGWDYPKCDRAMPNMLRIM   5000
DQ022305        KLLKSIAATRGATVVIGTSKFYGGWHNMLKTVYSDVESPHLMGWDYPKCDRAMPNMLRIM   4996
KF569996.1      KLLKSIAATRGATVVIGTSKFYGGWHNMLKTVYSDVETPHLMGWDYPKCDRAMPNMLRIM   4939
KP886809.1      KLLKSIAATRGATVVIGTSKFYGGWHNMLKTVYSDVETPHLMGWDYPKCDRAMPNMLRIM   5002
AY278488.2      KLLKSIAATRGATVVIGTSKFYGGWHNMLKTVYSDVETPHLMGWDYPKCDRAMPNMLRIM   5002
AY485277.1      KLLKSIAATRGATVVIGTSKFYGGWHNMLKTVYSDVETPHLMGWDYPKCDRAMPNMLRIM   5002
AP006560.1      KLLKSIAATRGATVVIGTSKFYGGWHNMLKTVYSDVETPHLMGWDYPKCDRAMPNMLRIM   5002
AP006557.1      KLLKSIAATRGATVVIGTSKFYGGWHNMLKTVYSDVETPHLMGWDYPKCDRAMPNMLRIM   5002
AY274119        KLLKSIAATRGATVVIGTSKFYGGWHNMLKTVYSDVETPHLMGWDYPKCDRAMPNMLRIM   5002
AY572038.1      KLLKSIAATRGATVVIGTSKFYGGWHNMLKTVYSDVETPHLMGWDYPKCDRAMPNMLRIM   5002
AY572034.1      KLLKSIAATRGATVVIGTSKFYGGWHNMLKTVYSDVETPHLMGWDYPKCDRAMPNMLRIM   5002
FJ588686.1      KLLKSIAATRGATVVIGTSKFYGGWHNMLKTVYSDVETPHLMGWDYPKCDRAMPNMLRIM   4809
KY417145.1      KLLKSIAATRGATVVIGTSKFYGGWHNMLKTVYSDVETPHLMGWDYPKCDRAMPNMLRIM   5002
KY417144.1      KLLKSIAATRGATVVIGTSKFYGGWHNMLKTVYSDVETPHLMGWDYPKCDRAMPNMLRIM   5002
KY417147.1      KLLKSIAATRGATVVIGTSKFYGGWHNMLKTVYSDVETPHLMGWDYPKCDRAMPNMLRIM   5002
KY417148.1      KLLKSIAATRGATVVIGTSKFYGGWHNMLKTVYSDVETPHLMGWDYPKCDRAMPNMLRIM   5002
KY417143.1      KLLKSIAATRGATVVIGTSKFYGGWHNMLKTVYSDVETPHLMGWDYPKCDRAMPNMLRIM   5002
KT444582.1      KLLKSIAATRGATVVIGTSKFYGGWHNMLKTVYSDVETPHLMGWDYPKCDRAMPNMLRIM   5002
KC881005.1      KLLKSIAATRGATVVIGTSKFYGGWHNMLKTVYSDVETPHLMGWDYPKCDRAMPNMLRIM   5002
KC881006.1      KLLKSIAATRGATVVIGTSKFYGGWHNMLKTVYSDVETPHLMGWDYPKCDRAMPNMLRIM   5002
KF367457.1      KLLKSIAATRGATVVIGTSKFYGGWHNMLKTVYSDVETPHLMGWDYPKCDRAMPNMLRIM   5002
KY417152.1      KLLKSIAATRGATVVIGTSKFYGGWHNMLKTVYSDVETPHLMGWDYPKCDRAMPNMLRIM   5002
KY417146.1      KLLKSIAATRGATVVIGTSKFYGGWHNMLKTVYSDVETPHLMGWDYPKCDRAMPNMLRIM   5002
KY417151.1      KLLKSIAATRGATVVIGTSKFYGGWHNMLKTVYSDVETPHLMGWDYPKCDRAMPNMLRIM   5002
KY417142.1      KLLKSIAATRGATVVIGTSKFYGGWHNMLKTVYSDVETPHLMGWDYPKCDRAMPNMLRIM   5002
                *************************:***********.*:********************
```

CoVAX_ORF1ab#8 CoVAX_ORF1ab#17

```
MW532698.1      ASLVLARKHTTCCSLSHRFYRLANECAQVLSEMVMCGGSLYVKPGGTSSGDATTAYANSV    5077
MT040336.1      ASLVLARKHTTCCSLSHRFYRLANECAQVLSEMVMCGGSLYVKPGGTSSGDATTAYANSV    5077
MT040335.1      ASLVLARKHTTCCSLSHRFYRLANECAQVLSEMVMCGGSLYVKPGGTSSGDATTAYANSV    5077
MT040334.1      ASLVLARKHTTCCSLSHRFYRLANECAQVLSEMVMCGGSLYVKPGGTSSGDATTAYANSV    5077
MT040333.1      ASLVLARKHTTCCSLSHRFYRLANECAQVLSEMVMCGGSLYVKPGGTSSGDATTAYANSV    5077
MN996532.2      ASLVLARKHTTCCSLSHRFYRLANECAQVLSEMVMCGGSLYVKPGGTSSGDATTAYANSV    5084
MN988713.1      ASLVLARKHTTCCSLSHRFYRLANECAQVLSEMVMCGGSLYVKPGGTSSGDATTAYANSV    5085
MT093571.1      ASLVLARKHTTCCSLSHRFYRLANECAQVLSEMVMCGGSLYVKPGGTSSGDATTAYANSV    5085
MN996529.1      ASLVLARKHTTCCSLSHRFYRLANECAQVLSEMVMCGGSLYVKPGGTSSGDATTAYANSV    5085
MT072688.1      ASLVLARKHTTCCSLSHRFYRLANECAQVLSEMVMCGGSLYVKPGGTSSGDATTAYANSV    5085
NC_045512.2     ASLVLARKHTTCCSLSHRFYRLANECAQVLSEMVMCGGSLYVKPGGTSSGDATTAYANSV    5085
MN994467.1      ASLVLARKHTTCCSLSHRFYRLANECAQVLSEMVMCGGSLYVKPGGTSSGDATTAYANSV    5085
MG772933.1      ASLVLARKHSTCCNLSHRFYRLANECAQVLSEMVMCGGSLYVKPGGTSSGDATTAYANSV    5081
MG772934.1      ASLVLARKHSTCCNLSHRFYRLANECAQVLSEMVMCGGSLYVKPSGTSSGDATTAYANSV    5059
NC_014470.1     ASLVLARKHSTCCNLSHRFYGLANECAQVLSEMVMCGGSLYVKPGGTSSGDATTAYANSV    5053
GQ153542.1      ASLVLARKHSTCCNLSHRFYRLANECAQVLSEMVMCGGSLYVKPGGTSSGDATTAYANSV    5060
DQ022305        ASLILARKHSTCCNLSHRFYRLANECAQVLSEMVMCGGSLYVKPGGTSSGDATTAYANSV    5056
KF569996.1      ASLVLARKHSTCCNLSHRFYRLANECAQVLSEMVMCGGSLYVKPGGTSSGDATTAYANSV    4999
KP886809.1      ASLVLARKHSTCCNLSHRFYRLANECAQVLSEMVMCGGSLYVKPGGTSSGDATTAYANSV    5062
AY278488.2      ASLVLARKHNTCCNLSHRFYRLANECAQVLSEMVMCGGSLYVKPGGTSSGDATTAYANSV    5062
AY485277.1      ASLVLARKHNTCCNLSHRFYRLANECAQVLSEMVMCGGSLYVKPGGTSSGDATTAYANSV    5062
AP006560.1      ASLVLARKHNTCCNLSHRFYRLANECAQVLSEMVMCGGSLYVKPGGTSSGDATTAYANSV    5062
AP006557.1      ASLVLARKHNTCCNLSHRFYRLANECAQVLSEMVMCGGSLYVKPGGTSSGDATTAYANSV    5062
AY274119        ASLVLARKHNTCCNLSHRFYRLANECAQVLSEMVMCGGSLYVKPGGTSSGDATTAYANSV    5062
AY572038.1      ASLVLARKHNTCCNLSHRFYRLANECAQVLSEMVMCGGSLYVKPGGTSSGDATTAYANSV    5062
AY572034.1      ASLVLARKHNTCCNLSHRFYRLANECAQVLSEMVMCGGSLYVKPGGTSSGDATTAYANSV    5062
FJ588686.1      ASLVLARKHSTCCNLSHRFYRLANECAQVLSEMVMCGGSLYVKPGGTSSGDATTAYANSV    4869
KY417145.1      ASLVLARKHSTCCNLSHRFYRLANECAQVLSEMVMCGGSLYVKPGGTSSGDATTAYANSV    5062
KY417144.1      ASLVLARKHSTCCNLSHRFYRLANECAQVLSEMVMCGGSLYVKPGGTSSGDATTAYANSV    5062
KY417147.1      ASLVLARKHSTCCNLSHRFYRLANECAQVLSEMVMCGGSLYVKPGGTSSGDATTAYANSV    5062
KY417148.1      ASLVLARKHSTCCNLSHRFYRLANECAQVLSEMVMCGGSLYVKPGGTSSGDATTAYANSV    5062
KY417143.1      ASLVLARKHSTCCNLSHRFYRLANECAQVLSEMVMCGGSLYVKPGGTSSGDATTAYANSV    5062
KT444582.1      ASLVLARKHSTCCNLSHRFYRLANECAQVLSEMVMCGGSLYVKPGGTSSGDATTAYANSV    5062
KC881005.1      ASLVLARKHSTCCNLSHRFYRLANECAQVLSEMVMCGGSLYVKPGGTSSGDATTAYANSV    5062
KC881006.1      ASLVLARKHSTCCNLSHRFYRLANECAQVLSEMVMCGGSLYVKPGGTSSGDATTAYANSV    5062
KF367457.1      ASLVLARKHSTCCNLSHRFYRLANECAQVLSEMVMCGGSLYVKPGGTSSGDATTAYANSV    5062
KY417152.1      ASLVLARKHSTCCNLSHRFYRLANECAQVLSEMVMCGGSLYVKPGGTSSGDATTAYANSV    5062
KY417146.1      ASLVLARKHSTCCNLSHRFYRLANECAQVLSEMVMCGGSLYVKPGGTSSGDATTAYANSV    5062
KY417151.1      ASLVLARKHSTCCNLSHRFYRLANECAQVLSEMVMCGGSLYVKPGGTSSGDATTAYANSV    5062
KY417142.1      ASLVLARKHSTCCNLSHRFYRLANECAQVLSEMVMCGGSLYVKPGGTSSGDATTAYANSV    5062
                ***:*****.***.***** ***********************.***************
```

CoVAX_ORF1ab#17 CoVAX_ORF1ab#14

```
MW532698.1     FNICQAVTANVNALLSTDGNKIGDKYIRNLQHRLYECLYRNRDVDTDFVNEFYAYLRKHF     5137
MT040336.1     FNICQAVTANVNALLSTDGNKIGDKYIRNLQHRLYECLYRNRDVDTDFVNEFYAYLRKHF     5137
MT040335.1     FNICQAVTANVNALLSTDGNKIGDKYIRNLQHRLYECLYRNRDVDTDFVNEFYAYLRKHF     5137
MT040334.1     FNICQAVTANVNALLSTDGNKIGDKYIRNLQHRLYECLYRNRDVDTDFVNEFYAYLRKHF     5137
MT040333.1     FNICQAVTANVNALLSTDGNKIGDKYIRNLQHRLYECLYRNRDVDTDFVNEFYAYLRKHF     5137
MN996532.2     FNICQAVTANVNALLSTDGNKIADKHVRNLQHRLYECLYRNRDVDTDFVNEFYAYLRKHF     5144
MN988713.1     FNICQAVTANVNALLSTDGNKIADKYVRNLQHRLYECLYRNRDVDTDFVNEFYAYLRKHF     5145
MT093571.1     FNICQAVTANVNALLSTDGNKIADKYVRNLQHRLYECLYRNRDVDTDFVNEFYAYLRKHF     5145
MN996529.1     FNICQAVTANVNALLSTDGNKIADKYVRNLQHRLYECLYRNRDVDTDFVNEFYAYLRKHF     5145
MT072688.1     FNICQAVTANVNALLSTDGNKIADKYVRNLQHRLYECLYRNRDVDTDFVNEFYAYLRKHF     5145
NC_045512.2    FNICQAVTANVNALLSTDGNKIADKYVRNLQHRLYECLYRNRDVDTDFVNEFYAYLRKHF     5145
MN994467.1     FNICQAVTANVNALLSTDGNKIADKYVRNLQHRLYECLYRNRDVDTDFVNEFYAYLRKHF     5145
MG772933.1     FNICQAVTANVNALLSTDGNKIADKYVRNLQHRLYECLYRNRDVDQEFVDEFYAYLRKHF     5141
MG772934.1     FNICQAVTANVNALLSTDGNKIADKYVRNLQHRLYECLYRNRDVDHEFVDEFYAYLRKHF     5119
NC_014470.1    FNICQAVTANVNALLSTDGNKIADKYVRNLQHRLYECLYRNRDVDHEFVEEFYAYLRKHF     5113
GQ153542.1     FNICQAVTANVNALLSTDGNKIADKYVRNLQHRLYECLYRNRDVDHEFVDEFYAYLRKHF     5120
DQ022305       FNICQAVTANVNALLSTDGNKIADKYVRNLQHRLYECLYRNRDVDHEFVDEFYAYLRKHF     5116
KF569996.1     FNICQAVTANVNALLSTDGNKIADKYVRNLQHRLYECLYRNRDVDHEFVDEFYAYLRKHF     5059
KP886809.1     FNICQAVTANVNALLSTDGNKIADKYVRNLQHRLYECLYRNRDVDHEFVDEFYAYLRKHF     5122
AY278488.2     FNICQAVTANVNALLSTDGNKIADKYVRNLQHRLYECLYRNRDVDHEFVDEFYAYLRKHF     5122
AY485277.1     FNICQAVTANVNALLSTDGNKIADKYVRNLQHRLYECLYRNRDVDHEFVDEFYAYLRKHF     5122
AP006560.1     FNICQAVTANVNALLSTDGNKIADKYVRNLQHRLYECLYRNRDVDHEFVDEFYAYLRKHF     5122
AP006557.1     FNICQAVTANVNALLSTDGNKIADKYVRNLQHRLYECLYRNRDVDHEFVDEFYAYLRKHF     5122
AY274119       FNICQAVTANVNALLSTDGNKIADKYVRNLQHRLYECLYRNRDVDHEFVDEFYAYLRKHF     5122
AY572038.1     FNICQAVTANVNALLSTDGNKIADKYVRNLQHRLYECLYRNRDVDHEFVDEFYAYLRKHF     5122
AY572034.1     FNICQAVTANVNALLSTDGNKIADKYVRNLQHRLYECLYRNRDVDHEFVDEFYAYLRKHF     5122
FJ588686.1     FNICQAVTANVNALLSTDGNKIADKYVRNLQHRLYECLYRNRDVDHEFVDEFYAYLRKHF     4929
KY417145.1     FNICQAVTANVNALLSTDGNKIADKYVRNLQHRLYECLYRNRDVDHEFVDEFYAYLRKHF     5122
KY417144.1     FNICQAVTANVNALLSTDGNKIADKYVRNLQHRLYECLYRNRDVDHEFVDEFYAYLRKHF     5122
KY417147.1     FNICQAVTANVNALLSTDGNKIADKYVRNLQHRLYECLYRNRDVDHEFVNEFYAYLRKHF     5122
KY417148.1     FNICQAVTANVNALLSTDGNKIADKYVRNLQHRLYECLYRNRDVDHEFVDEFYAYLRKHF     5122
KY417143.1     FNICQAVTANVNALLSTDGNKIADKYVRNLQHRLYECLYRNRDVDHEFVDEFYAYLRKHF     5122
KT444582.1     FNICQAVTANVNALLSTDGNKIADKYVRNLQHRLYECLYRNRDVDHEFVDEFYAYLRKHF     5122
KC881005.1     FNICQAVTANVNALLSTDGNKIADKYVRNLQHRLYECLYRNRDVDHEFVDEFYAYLRKHF     5122
KC881006.1     FNICQAVTANVNALLSTDGNKIADKYVRNLQHRLYECLYRNRDVDHEFVDEFYAYLRKHF     5122
KF367457.1     FNICQAVTANVNALLSTDGNKIADKYVRNLQHRLYECLYRNRDVDHEFVDEFYAYLRKHF     5122
KY417152.1     FNICQAVTANVNALLSTDGNKIADKYVCNLQHRLYECLYRNRDVDHEFVDEFYAYLRKHF     5122
KY417146.1     FNICQAVTANVNALLSTDGNKIADKYVRNLQHRLYECLYRNRDIDHEFVDEFYAYLRKHF     5122
KY417151.1     FNICQAVTANVNALLSTDGNKIADKYVRNLQHRLYECLYRNRDVDHEFVDEFYAYLRKHF     5122
KY417142.1     FNICQAVTANVNALLSTDGNKIADKYVRNLQHRLYECLYRNRDVDHEFVDEFYAYLRKHF     5122
               **********************.**:: ***************:* :**:**********
```

CoVAX_ORF1ab#14

CoVAX_ORF1ab#1

```
MW532698.1     SMMILSDDAVVCFNSTYASQGLVASIKNFKSVLYYQNNVFMSEAKCWTETDLTKGPHEFC     5197
MT040336.1     SMMILSDDAVVCFNSTYASQGLVASIKNFKSVLYYQNNVFMSEAKCWTETDLTKGPHEFC     5197
MT040335.1     SMMILSDDAVVCFNSTYASQGLVASIKNFKSVLYYQNNVFMSEAKCWTETDLTKGPHEFC     5197
MT040334.1     SMMILSDDAVVCFNSTYASQGLVASIKNFKSVLYYQNNVFMSEAKCWTETDLTKGPHEFC     5197
MT040333.1     SMMILSDDAVVCFNSTYASQGLVASIKNFKSVLYYQNNVFMSEAKCWTETDLTKGPHEFC     5197
MN996532.2     SMMILSDDAVVCFNSTYASQGLVASIKNFKSVLYYQNNVFMSEAKCWTETDLTKGPHEFC     5204
MN988713.1     SMMILSDDAVVCFNSTYASQGLVASIKNFKSVLYYQNNVFMSEAKCWTETDLTKGPHEFC     5205
MT093571.1     SMMILSDDAVVCFNSTYASQGLVASIKNFKSVLYYQNNVFMSEAKCWTETDLTKGPHEFC     5205
MN996529.1     SMMILSDDAVVCFNSTYASQGLVASIKNFKSVLYYQNNVFMSEAKCWTETDLTKGPHEFC     5205
MT072688.1     SMMILSDDAVVCFNSTYASQGLVASIKNFKSVLYYQNNVFMSEAKCWTETDLTKGPHEFC     5205
NC_045512.2    SMMILSDDAVVCFNSTYASQGLVASIKNFKSVLYYQNNVFMSEAKCWTETDLTKGPHEFC     5205
MN994467.1     SMMILSDDAVVCFNSTYASQGLVASIKNFKSVLYYQNNVFMSEAKCWTETDLTKGPHEFC     5205
MG772933.1     SMMILSDDAVVCYNSNYAAQGLVASIKNFKAVLYYQNNVFMSEAKCWTETDLTKGPHEFC     5201
MG772934.1     SMMILSDDAVVCYNSNYAAQGLVASIKNFKAVLYYQNNVFMSEAKCWTETDLTKGPHEFC     5179
NC_014470.1    SMMILSDDAVVCYNSNYAAQGLVASIKNFKAVLYYQNNVFMSEAKCWTETDLTKGPHEFC     5173
GQ153542.1     SMMILSDDAVVCYNSNYAAQGLVASIKNFKAVLYYQNNVFMSEAKCWTETDLTKGPHEFC     5180
DQ022305       SMMILSDDAVVCYNSNYAAQGLVASIKNFKAVLYYQNNVFMSEAKCWTETDLTRGPHEFC     5176
KF569996.1     SMMILSDDAVVCYNSNYAAQGLVASIKNFKAVLYYQNNVFMSEAKCWTETDLTKGPHEFC     5119
KP886809.1     SMMILSDDAVVCYNSNYAAQGLVASIKNFKAVLYYQNNVFMSEAKCWTETDLTKGPHEFC     5182
AY278488.2     SMMILSDDAVVCYNSNYAAQGLVASIKNFKAVLYYQNNVFMSEAKCWTETDLTKGPHEFC     5182
AY485277.1     SMMILSDDAVVCYNSNYAAQGLVASIKNFKAVLYYQNNVFMSEAKCWTETDLTKGPHEFC     5182
AP006560.1     SMMILSDDAVVCYNSNYAAQGLVASIKNFKAVLYYQNNVFMSEAKCWTETDLTKGPHEFC     5182
AP006557.1     SMMILSDDAVVCYNSNYAAQGLVASIKNFKAVLYYQNNVFMSEAKCWTETDLTKGPHEFC     5182
AY274119       SMMILSDDAVVCYNSNYAAQGLVASIKNFKAVLYYQNNVFMSEAKCWTETDLTKGPHEFC     5182
AY572038.1     SMMILSDDAVVCYNSNYAAQGLVASIKNFKAVLYYQNNVFMSEAKCWTETDLTKGPHEFC     5182
AY572034.1     SMMILSDDAVVCYNSNYAAQGLVASIKNFKAVLYYQNNVFMSEAKCWTETDLTKGPHEFC     5182
FJ588686.1     SMMILSDDAVVCYNSNYAAQGLVASIKNFKAVLYYQNNVFMSEAKCWTETDLTKGPHEFC     4989
KY417145.1     SMMILSDDAVVCYNSNYAAQGLVASIKNFKAVLYYQNNVFMSEAKCWTETDLTKGPHEFC     5182
KY417144.1     SMMILSDDAVVCYNSNYAAQGLVASIKNFKAVLYYQNNVFMSEAKCWTETDLTKGPHEFC     5182
KY417147.1     SMMILSDDAVVCYNSNYAAQGLVASIKNFKAVLYYQNNVFMSEAKCWTETDLTKGPHEFC     5182
KY417148.1     SMMILSDDAVVCYNSNYAAQGLVASIKNFKAVLYYQNNVFMSEAKCWTETDLTKGPHEFC     5182
KY417143.1     SMMILSDDAVVCYNSNYAAQGLVASIKNFKAVLYYQNNVFMSEAKCWTETDLTKGPHEFC     5182
KT444582.1     SMMILSDDAVVCYNSNYAAQGLVASIKNFKAVLYYQNNVFMSEAKCWTETDLTKGPHEFC     5182
KC881005.1     SMMILSDDAVVCYNSNYAAQGLVASIKNFKAVLYYQNNVFMSEAKCWTETDLTKGPHEFC     5182
KC881006.1     SMMILSDDAVVCYNSNYAAQGLVASIKNFKAVLYYQNNVFMSEAKCWTETDLTKGPHEFC     5182
KF367457.1     SMMILSDDAVVCYNSNYAAQGLVASIKNFKAVLYYQNNVFMSEAKCWTETDLTKGPHEFC     5182
KY417152.1     SMMILSDDAVVCYNSNYAAQGLVASIKNFKAVLYYQNNVFMSEAKCWTETDLTKGPHEFC     5182
KY417146.1     SMMILSDDAVVCYNSNYAAQGLVASIKNFKAVLYYQNNVFMSEAKCWTETDLTKGPHEFC     5182
KY417151.1     SMMILSDDAVVCYNSNYAAQGLVASIKNFKAVLYYQNNVFMSEAKCWTETDLTKGPHEFC     5182
KY417142.1     SMMILSDDAVVCYNSNYAAQGLVASIKNFKAVLYYQNNVFMSEAKCWTETDLTKGPHEFC     5182
               ************:**.**:***********:**********************:******
```

CoVAX_ORF1ab#1   CoVAX_ORF1ab#9

```
MW532698.1     SQHTMLVKQGDDYVYLPYPDPSRILGAGCFVDDIVKTDGTLMIERFVSLAIDAYPLTKHP     5257
MT040336.1     SQHTMLVKQGDDYVYLPYPDPSRILGAGCFVDDIVKTDGTLMIERFVSLAIDAYPLTKHP     5257
MT040335.1     SQHTMLVKQGDDYVYLPYPDPSRILGAGCFVDDIVKTDGTLMIERFVSLAIDAYPLTKHP     5257
MT040334.1     SQHTMLVKQGDDYVYLPYPDPSRILGAGCFVDDIVKTDGTLMIERFVSLAIDAYPLTKHP     5257
MT040333.1     SQHTMLVKQGDDYVYLPYPDPSRILGAGCFVDDIVKTDGTLMIERFVSLAIDAYPLTKHP     5257
MN996532.2     SQHTMLVKQGDDYVYLPYPDPSRILGAGCFVDDIVKTDGTLMIERFVSLAIDAYPLTKHP     5264
MN988713.1     SQHTMLVKQGDDYVYLPYPDPSRILGAGCFVDDIVKTDGTLMIERFVSLAIDAYPLTKHP     5265
MT093571.1     SQHTMLVKQGDDYVYLPYPDPSRILGAGCFVDDIVKTDGTLMIERFVSLAIDAYPLTKHP     5265
MN996529.1     SQHTMLVKQGDDYVYLPYPDPSRILGAGCFVDDIVKTDGTLMIERFVSLAIDAYPLTKHP     5265
MT072688.1     SQHTMLVKQGDDYVYLPYPDPSRILGAGCFVDDIVKTDGTLMIERFVSLAIDAYPLTKHP     5265
NC_045512.2    SQHTMLVKQGDDYVYLPYPDPSRILGAGCFVDDIVKTDGTLMIERFVSLAIDAYPLTKHP     5265
MN994467.1     SQHTMLVKQGDDYVYLPYPDPSRILGAGCFVDDIVKTDGTLMIERFVSLAIDAYPLTKHP     5265
MG772933.1     SQHTMLVKQGDDYVYLPYPDPSRILGAGCFVDDIVKTDGTLMIERFVSLAIDAYPLTKHP     5261
MG772934.1     SQHTMLVKQGDDYVYLPYPDPSRILGAGCFVDDIVKTDGTLMIERFVSLAIDAYPLTKHP     5239
NC_014470.1    SQHTMLVKQGDDYVYLPYPDPSRILGAGCFVDDIVKTDGTLMIERFVSLAIDAYPLTKHP     5233
GQ153542.1     SQHTMLVKQGDDYVYLPYPDPSRILGAGCFVDDIVKTDGTLMIERFVSLAIDAYPLTKHP     5240
DQ022305       SQHTMLVKQGDDYVYLPYPDPSRILGAGCFVDDIVKTDGTLMIERFVSLAIDAYPLTKHP     5236
KF569996.1     SQHTMLVKQGDDYVYLPYPDPSRILGAGCFVDDIVKTDGTLMIERFVSLAIDAYPLTKHP     5179
KP886809.1     SQHTMLVKQGDDYVYLPYPDPSRILGAGCFVDDIVKTDGTLMIERFVSLAIDAYPLTKHP     5242
AY278488.2     SQHTMLVKQGDDYVYLPYPDPSRILGAGCFVDDIVKTDGTLMIERFVSLAIDAYPLTKHP     5242
AY485277.1     SQHTMLVKQGDDYVYLPYPDPSRILGAGCFVDDIVKTDGTLMIERFVSLAIDAYPLTKHP     5242
AP006560.1     SQHTMLVKQGDDYVYLPYPDPSRILGAGCFVDDIVKTDGTLMIERFVSLAIDAYPLTKHP     5242
AP006557.1     SQHTMLVKQGDDYVYLPYPDPSRILGAGCFVDDIVKTDGTLMIERFVSLAIDAYPLTKHP     5242
AY274119       SQHTMLVKQGDDYVYLPYPDPSRILGAGCFVDDIVKTDGTLMIERFVSLAIDAYPLTKHP     5242
AY572038.1     SQHTMLVKQGDDYVYLPYPDPSRILGAGCFVDDIVKTDGTLMIERFVSLAIDAYPLTKHP     5242
AY572034.1     SQHTMLVKQGDDYVYLPYPDPSRILGAGCFVDDIVKTDGTLMIERFVSLAIDAYPLTKHP     5242
FJ588686.1     SQHTMLVKQGDDYVYLPYPDPSRILGAGCFVDDIVKTDGTLMIERFVSLAIDAYPLTKHP     5049
KY417145.1     SQHTMLVKQGDDYVYLPYPDPSRILGAGCFVDDIVKTDGTLMIERFVSLAIDAYPLTKHP     5242
KY417144.1     SQHTMLVKQGDDYVYLPYPDPSRILGAGCFVDDIVKTDGTLMIERFVSLAIDAYPLTKHP     5242
KY417147.1     SQHTMLVKQGDDYVYLPYPDPSRILGAGCFVDDIVKTDGTLMIERFVSLAIDAYPLTKHP     5242
KY417148.1     SQHTMLVKQGDDYVYLPYPDPSRILGAGCFVDDIVKTDGTLMIERFVSLAIDAYPLTKHP     5242
KY417143.1     SQHTMLVKQGDDYVYLPYPDPSRILGAGCFVDDIVKTDGTLMIERFVSLAIDAYPLTKHP     5242
KT444582.1     SQHTMLVKQGDDYVYLPYPDPSRILGAGCFVDDIVKTDGTLMIERFVSLAIDAYPLTKHP     5242
KC881005.1     SQHTMLVKQGDDYVYLPYPDPSRILGAGCFVDDIVKTDGTLMIERFVSLAIDAYPLTKHP     5242
KC881006.1     SQHTMLVKQGDDYVYLPYPDPSRILGAGCFVDDIVKTDGTLMIERFVSLAIDAYPLTKHP     5242
KF367457.1     SQHTMLVKQGDDYVYLPYPDPSRILGAGCFVDDIVKTDGTLMIERFVSLAIDAYPLTKHP     5242
KY417152.1     SQHTMLVKQGDDYVYLPYPDPSRILGAGCFVDDIVKTDGTLMIERFVSLAIDAYPLTKHP     5242
KY417146.1     SQHTMLVKQGDDYVYLPYPDPSRILGAGCFVDDIVKTDGTLMIERFVSLAIDAYPLTKHP     5242
KY417151.1     SQHTMLVKQGDDYVYLPYPDPSRILGAGCFVDDIVKTDGTLMIERFVSLAIDAYPLTKHP     5242
KY417142.1     SQHTMLVKQGDDYVYLPYPDPSRILGAGCFVDDIVKTDGTLMIERFVSLAIDAYPLTKHP     5242
               ************************************************************
```

CoVAX_ORF1ab#9   CoVAX_ORF1ab#13

MW532698.1	NQEYADVFHLYLQYIRKLHDELTGHMLDMYSVMLTNDSTSRYWEPEFYEAMYTPHTVLQA	5317
MT040336.1	NQEYADVFHLYLQYIRKLHDELTGHMLDMYSVMLTNDSTSRYWEPEFYEAMYTPHTVLQA	5317
MT040335.1	NQEYADVFHLYLQYIRKLHDELTGHMLDMYSVMLTNDSTSRYWEPEFYEAMYTPHTVLQA	5317
MT040334.1	NQEYADVFHLYLQYIRKLHDELTGHMLDMYSVMLTNDSTSRYWEPEFYEAMYTPHTVLQA	5317
MT040333.1	NQEYADVFHLYLQYIRKLHDELTGHMLDMYSVMLTNDSTSRYWEPEFYEAMYTPHTVLQA	5317
MN996532.2	NQEYADVFHLYLQYIRKLHDELTGHMLDMYSVMLTNDNTSRYWEPEFYEAMYTPHTVLQA	5324
MN988713.1	NQEYADVFHLYLQYIRKLHDELTGHMLDMYSVMLTNDNTSRYWEPEFYEAMYTPHTVLQA	5325
MT093571.1	NQEYADVFHLYLQYIRKLHDELTGHMLDMYSVMLTNDNTSRYWEPEFYEAMYTPHTVLQA	5325
MN996529.1	NQEYADVFHLYLQYIRKLHDELTGHMLDMYSVMLTNDNTSRYWEPEFYEAMYTPHTVLQA	5325
MT072688.1	NQEYADVFHLYLQYIRKLHDELTGHMLDMYSVMLTNDNTSRYWEPEFYEAMYTPHTVLQA	5325
**NC_045512.2**	**NQEYADVFHLYLQYIRKLHDELTGHMLDMYSVMLTNDNTSRYWEPEFYEAMYTPHTVLQA**	**5325**
MN994467.1	NQEYADVFHLYLQYIRKLHDELTGHMLDMYSVMLTNDNTSRYWEPEFYEAMYTPHTVLQA	5325
MG772933.1	NQEYADVFHLYLQYIRKLHDELTGHMLDMYSVMLTNDNTSRYWEPEFYEAMYTPHTVLQA	5321
MG772934.1	NQEYADVFHLYLQYIRKLHDELTGHMLDMYSVMLTNDNTSRYWEPEFYEAMYTPHTVLQA	5299
NC_014470.1	NQEYADVFHLYLQYIRKLHDELTGHMLDMYSVMLTNDNTSRYWEPEFYEAMYTPHTVLQA	5293
GQ153542.1	NQEYADVFHLYLQYIRKLHDELTGHMLDMYSVMLTNDNTSRYWEPEFYEAMYTPHTVLQA	5300
DQ022305	NQEYADVFHLYLQYIRKLHDELTGHMLDMYSVMLTNDNTSRYWEPEFYEAMYTPHTVLQA	5296
KF569996.1	NQEYADVFHLYLQYIRKLHDELTGHMLDMYSVMLTNDNTSRYWEPEFYEAMYTPHTVLQA	5239
KP886809.1	NQEYADVFHLYLQYIRKLHDELTGHMLDMYSVMLTNDNTSRYWEPEFYEAMYTPHTVLQA	5302
AY278488.2	NQEYADVFHLYLQYIRKLHDELTGHMLDMYSVMLTNDNTSRYWEPEFYEAMYTPHTVLQA	5302
AY485277.1	NQEYAAVFHLYLQYIRKLHDELTGHMLDMYSVMLTNDNTSRYWEPEFYEAMYTPHTVLQA	5302
AP006560.1	NQEYADVFHLYLQYIRKLHDELTGHMLDMYSVMLTNDNTSRYWEPEFYEAMYTPHTVLQA	5302
AP006557.1	NQEYADVFHLYLQYIRKLHDELTGHMLDMYSVMLTNDNTSRYWEPEFYEAMYTPHTVLQA	5302
AY274119	NQEYADVFHLYLQYIRKLHDELTGHMLDMYSVMLTNDNTSRYWEPEFYEAMYTPHTVLQA	5302
AY572038.1	NQEYADVFHLYLQYIRKLHDELTGHMLDMYSVMLTNDNTSRYWEPEFYEAMYTPHTVLQA	5302
AY572034.1	NQEYADVFHLYLQYIRKLHDELTGHMLDMYSVMLTNDNTSRYWEPEFYEAMYTPHTVLQA	5302
FJ588686.1	NQEYADVFHLYLQYIRKLHDELTGHMLDMYSVMLTNDNTSRYWEPEFYEAMYTPHTVLQA	5109
KY417145.1	NQEYADVFHLYLQYIRKLHDELTGHMLDMYSVMLTNDNTSRYWEPEFYEAMYTPHTVLQA	5302
KY417144.1	NQEYADVFHLYLQYIRKLHDELTGHMLDMYSVMLTNDNTSRYWEPEFYEAMYTPHTVLQA	5302
KY417147.1	NQEYADVFHLYLQYIRKLHDELTGHMLDMYSVMLTNDNTSRYWEPEFYEAMYTPHTVLQA	5302
KY417148.1	NQEYADVFHLYLQYIRKLHDELTGHMLDMYSVMLTNDNTSRYWEPEFYEAMYTPHTVLQA	5302
KY417143.1	NQEYADVFHLYLQYIRKLHDELTGHMLDMYSVMLTNDNTSRYWEPEFYEAMYTPHTVLQA	5302
KT444582.1	NQEYADVFHLYLQYIRKLHDELTGHMLDMYSVMLTNDNTSRYWEPEFYEAMYTPHTVLQA	5302
KC881005.1	NQEYADVFHLYLQYIRKLHDELTGHMLDMYSVMLTNDNTSRYWEPEFYEAMYTPHTVLQA	5302
KC881006.1	NQEYADVFHLYLQYIRKLHDELTGHMLDMYSVMLTNDNTSRYWEPEFYEAMYTPHTVLQA	5302
KF367457.1	NQEYADVFHLYLQYIRKLHDELTGHMLDMYSVMLTNDNTSRYWEPEFYEAMYTPHTVLQA	5302
KY417152.1	NQEYADVFHLYLQYIRKLHDELTGHMLDMYSVMLTNDNTSRYWEPEFYEAMYTPHTVLQA	5302
KY417146.1	NQEYADVFHLYLQYIRKLHDELTGHMLDMYSVMLTNDNTSRYWEPEFYEAMYTPHTVLQA	5302
KY417151.1	NQEYADVFHLYLQYIRKLHDELTGHMLDMYSVMLTNDNTSRYWEPEFYEAMYTPHTVLQA	5302
KY417142.1	NQEYADVFHLYLQYIRKLHDELTGHMLDMYSVMLTNDNTSRYWEPEFYEAMYTPHTVLQA	5302
	***** *******************************.**********************	

```
MW532698.1      VGACVLCNSQTSLRCGACIRRPFLCCKCCYDHVISTSHKLVLSVNPYVCNATGCDVTDVT      5377
MT040336.1      VGACVLCNSQTSLRCGACIRRPFLCCKCCYDHVISTSHKLVLSVNPYVCNATGCDVTDVT      5377
MT040335.1      VGACVLCNSQTSLRCGACIRRPFLCCKCCYDHVISTSHKLVLSVNPYVCNATGCDVTDVT      5377
MT040334.1      VGACVLCNSQTSLRCGACIRRPFLCCKCCYDHVISTSHKLVLSVNPYVCNATGCDVTDVT      5377
MT040333.1      VGACVLCNSQTSLRCGACIRRPFLCCKCCYDHVISTSHKLVLSVNPYVCNATGCDVTDVT      5377
MN996532.2      VGACVLCNSQTSLRCGACIRRPFLCCKCCYDHVISTSHKLVLSVNPYVCNAPGCDVTDVT      5384
MN988713.1      VGACVLCNSQTSLRCGACIRRPFLCCKCCYDHVISTSHKLVLSVNPYVCNAPGCDVTDVT      5385
MT093571.1      VGACVLCNSQTSLRCGACIRRPFLCCKCCYDHVISTSHKLVLSVNPYVCNAPGCDVTDVT      5385
MN996529.1      VGACVLCNSQTSLRCGACIRRPFLCCKCCYDHVISTSHKLVLSVNPYVCNAPGCDVTDVT      5385
MT072688.1      VGACVLCNSQTSLRCGACIRRPFLCCKCCYDHVISTSHKLVLSVNPYVCNAPGCDVTDVT      5385
NC_045512.2     VGACVLCNSQTSLRCGACIRRPFLCCKCCYDHVISTSHKLVLSVNPYVCNAPGCDVTDVT      5385
MN994467.1      VGACVLCNSQTSLRCGACIRRPFLCCKCCYDHVISTSHKLVLSVNPYVCNAPGCDVTDVT      5385
MG772933.1      VGACVLCNSQTSLRCGACIRRPFLCCKCCYDHVISTSHKLVLSVNPYVCNAPGCDVTDVT      5381
MG772934.1      VGACVLCNSQTSLRCGACIRRPFLCCKCCYDHVISTSHKLVLSVNPYVCNALGCDVTDVT      5359
NC_014470.1     VGACVLCNSQTSLRCGSCIRRPFLCCKCCYDHVISTSHKLVLSVNPYVCNAPGCDVTDVT      5353
GQ153542.1      VGACVLCNSQTSLRCGACIRRPFLCCKCCYDHVISTSHKLVLSVNPYVCNAPGCDVTDVT      5360
DQ022305        VGACVLCNSQTSLRCGACIRRPFLCCKCCYDHVISTSHKLVLSVNPYVCNAPGCDVTDVT      5356
KF569996.1      VGACVLCNSQTSLRCGACIRRPFLCCKCCYDHVISTSHKLVLSVNPYVCNAPGCDVTDVT      5299
KP886809.1      VGACVLCNSQTSLRCGACIRRPFLCCKCCYDHVISTSHKLVLSVNPYVCNAPGCDVTDVT      5362
AY278488.2      VGACVLCNSQTSLRCGACIRRPFLCCKCCYDHVISTSHKLVLSVNPYVCNAPGCDVTDVT      5362
AY485277.1      VGACVLCNSQTSLRCGACIRRPFLCCKCCYDHVISTSHKLVLSVNPYVCNAPGCDVTDVT      5362
AP006560.1      VGACVLCNSQTSLRCGACIRRPFLCCKCCYDHVISTSHKLVLSVNPYVCNAPGCDVTDVT      5362
AP006557.1      VGACVLCNSQTSLRCGACIRRPFLCCKCCYDHVISTSHKLVLSVNPYVCNAPGCDVTDVT      5362
AY274119        VGACVLCNSQTSLRCGACIRRPFLCCKCCYDHVISTSHKLVLSVNPYVCNAPGCDVTDVT      5362
AY572038.1      VGACVLCNSQTSLRCGACIRRPFLCCKCCYDHVISTSHKLVLSVNPYVCNAPGCDVTDVT      5362
AY572034.1      VGACVLCNSQTSLRCGACIRRPFLCCKCCYDHVISTSHKLVLSVNPYVCNAPGCDVTDVT      5362
FJ588686.1      VGACVLCNSQTSLRCGACIRRPFLCCKCCYDHVVSTSHKLVLSVNPYVCNAPGCDVTDVT      5169
KY417145.1      VGACVLCNSQTSLRCGACIRRPFLCCKCCYDHVISTSHKLVLSVNPYVCNAPGCDVTDVT      5362
KY417144.1      VGACVLCNSQTSLRCGACIRRPFLCCKCCYDHVISTSHKLVLSVNPYVCNAPGCDVTDVT      5362
KY417147.1      VGACVLCNSQTSLRCGACIRRPFLCCKCCYDHVISTSHKLVLSVNPYVCNAPGCDVTDVT      5362
KY417148.1      VGACVLCNSQTSLRCGACIRRPFLCCKCCYDHVISTSHKLVLSVNPYVCNAPGCDVTDVT      5362
KY417143.1      VGACVLCNSQTSLRCGACIRRPFLCCKCCYDHVISTSHKLVLSVNPYVCNAPGCDVTDVT      5362
KT444582.1      VGACVLCNSQTSLRCGACIRRPFLCCKCCYDHVISTSHKLVLSVNPYVCNAPGCDVTDVT      5362
KC881005.1      VGACVLCNSQTSLRCGACIRRPFLCCKCCYDHVISTSHKLVLSVNPYVCNAPGCDVTDVT      5362
KC881006.1      VGACVLCNSQTSLRCGACIRRPFLCCKCCYDHVISTSHKLVLSVNPYVCNAPGCDVTDVT      5362
KF367457.1      VGACVLCNSQTSLRCGACIRRPFLCCKCCYDHVISTSHKLVLSVNPYVCNAPGCDVTDVT      5362
KY417152.1      VGACVLCNSQTSLRCGACIRRPFLCCKCCYDHVISTSHKLVLSVNPYVCNAPGCDVTDVT      5362
KY417146.1      VGACVLCNSQTSLRCGACIRRPFLCCKCCYDHVISTSHKLVLSVNPYVCNAPGCDVTDVT      5362
KY417151.1      VGACVLCNSQTSLRCGACIRRPFLCCKCCYDHVISTSHKLVLSVNPYVCNAPGCDVTDVT      5362
KY417142.1      VGACVLCNSQTSLRCGACIRRPFLCCKCCYDHVISTSHKLVLSVNPYVCNAPGCDVTDVT      5362
                ****************:****************:***************** ********
```

CoVAX_ORF1ab#15

MW532698.1	QLYLGGMSYYCKAHKPPISFPLCANGQVFGLYKNTCVGSDNVTDFNAIATCDWTNAGDYI	5437
MT040336.1	QLYLGGMSYYCKAHKPPISFPLCANGQVFGLYKNTCVGSDNVTDFNAIATCDWTNAGDYI	5437
MT040335.1	QLYLGGMSYYCKAHKPPISFPLCANGQVFGLYKNTCVGSDNVTDFNAIATCDWTNAGDYI	5437
MT040334.1	QLYLGGMSYYCKAHKPPISFPLCANGQVFGLYKNTCVGSDNVTDFNAIATCDWTNAGDYI	5437
MT040333.1	QLYLGGMSYYCKAHKPPISFPLCANGQVFGLYKNTCVGSDNVTDFNAIATCDWTNAGDYI	5437
MN996532.2	QLYLGGMSYYCKSHKPPISFPLCANGQVFGLYKNTCVGSDNVTDFNAIATCDWTNAGDYI	5444
MN988713.1	QLYLGGMSYYCKSHKPPISFPLCANGQVFGLYKNTCVGSDNVTDFNAIATCDWTNAGDYI	5445
MT093571.1	QLYLGGMSYYCKSHKPPISFPLCANGQVFGLYKNTCVGSDNVTDFNAIATCDWTNAGDYI	5445
MN996529.1	QLYLGGMSYYCKSHKPPISFPLCANGQVFGLYKNTCVGSDNVTDFNAIATCDWTNAGDYI	5445
MT072688.1	QLYLGGMSYYCKSHKPPISFPLCANGQVFGLYKNTCVGSDNVTDFNAIATCDWTNAGDYI	5445
**NC_045512.2**	**QLYLGGMSYYCKSHKPPISFPLCANGQVFGLYKNTCVGSDNVTDFNAIATCDWTNAGDYI**	**5445**
MN994467.1	QLYLGGMSYYCKSHKPPISFPLCANGQVFGLYKNTCVGSDNVTDFNAIATCDWTNAGDYI	5445
MG772933.1	QLYLGGMSYYCKSHKPPISFPLCANGQVFGLYKNTCVGSDNVTDFNAIATCDWTNAGDYI	5441
MG772934.1	QLYLGGMSYYCKLHKPPISFPLCANGQVFGLYKNTCVGSDNVTDFNAIATCDWTNAGDYI	5419
NC_014470.1	QLYLGGMSYYCKSHKPPISFPLCANGQVFGLYKNTCVGSDNVTDFNAIATCDWTNAGDYI	5413
GQ153542.1	QLYLGGMSYYCKSHKPPISFPLCANGQVFGLYKNTCVGSDNVTDFNAIATCDWTNAGDYI	5420
DQ022305	QLYLGGMSYYCKSHKPPISFPLCANGQVFGLYKNTCVGSDNVTDFNAIATCDWTNAGDYI	5416
KF569996.1	QLYLGGMSYYCKSHKPPISFPLCANGQVFGLYKNTCVGSDNVTDFNAIATCDWTNAGDYI	5359
KP886809.1	QLYLGGMSYYCKSHKPPISFPLCANGQVFGLYKNTCVGSDNVTDFNAIATCDWTNAGDYI	5422
AY278488.2	QLYLGGMSYYCKSHKPPISFPLCANGQVFGLYKNTCVGSDNVTDFNAIATCDWTNAGDYI	5422
AY485277.1	QLYLGGMSYYCKSHKPPISFPLCANGQVFGLYKNTCVGSDNVTDFNAIATCDWTNAGDYI	5422
AP006560.1	QLYLGGMSYYCKSHKPPISFPLCANGQVFGLYKNTCVGSDNVTDFNAIATCDWTNAGDYI	5422
AP006557.1	QLYLGGMSYYCKSHKPPISFPLCANGQVFGLYKNTCVGSDNVTDFNAIATCDWTNAGDYI	5422
AY274119	QLYLGGMSYYCKSHKPPISFPLCANGQVFGLYKNTCVGSDNVTDFNAIATCDWTNAGDYI	5422
AY572038.1	QLYLGGMSYYCKSHKPPISFPLCANGQVFGLYKNTCVGSDNVTDFNAIATCDWTNAGDYI	5422
AY572034.1	QLYLGGMSYYCKSHKPPISFPLCANGQVFGLYKNTCVGSDNVTDFNAIATCDWTNAGDYI	5422
FJ588686.1	QLYLGGVSYYCKSHKPPISFPLCANGQVFGLYKNTCVGSDNVTDFNAIATCDWTNAGDYI	5229
KY417145.1	QLYLGGMSYYCKSHKPPISFPLCANGQVFGLYKNTCVGSDNVTDFNAIATCDWTNAGDYI	5422
KY417144.1	QLYLGGMSYYCKSHKPPISFPLCANGQVFGLYKNTCVGSDNVTDFNAIATCDWTNAGDYI	5422
KY417147.1	QLYLGGMSYYCKSHKPPISFPLCANGQVFGLYKNTCVGSDNVTDFNAIATCDWTNAGDYI	5422
KY417148.1	QLYLGGMSYYCKSHKPPISFPLCANGQVFGLYKNTCVGSDNVTDFNAIATCDWTNAGDYI	5422
KY417143.1	QLYLGGMSYYCKSHKPPISFPLCANGQVFGLYKNTCVGSDNVTDFNAIATCDWTNAGDYI	5422
KT444582.1	QLYLGGMSYYCKSHKPPISFPLCANGQVFGLYKNTCVGSDNVTDFNAIATCDWTNAGDYI	5422
KC881005.1	QLYLGGMSYYCKSHKPPISFPLCANGQVFGLYKNTCVGSDNVTDFNAIATCDWTNAGDYI	5422
KC881006.1	QLYLGGMSYYCKSHKPPISFPLCANGQVFGLYKNTCVGSDNVTDFNAIATCDWTNAGDYI	5422
KF367457.1	QLYLGGMSYYCKSHKPPISFPLCANGQVFGLYKNTCVGSDNVTDFNAIATCDWTNAGDYI	5422
KY417152.1	QLYLGGMSYYCKSHKPPISFPLCANGQVFGLYKNTCVGSDNVTDFNAIATCDWTNAGDYI	5422
KY417146.1	QLYLGGMSYYCKSHKPPISFPLCANGQVFGLYKNTCVGSDNVTDFNAIATCDWTNAGDYI	5422
KY417151.1	QLYLGGMSYYCKSHKPPISFPLCANGQVFGLYKNTCVGSDNVTDFNAIATCDWTNAGDYI	5422
KY417142.1	QLYLGGMSYYCKSHKPPISFPLCANGQVFGLYKNTCVGSDNVTDFNAIATCDWTNAGDYI	5422
	******:***** ***	

```
MW532698.1      LANTCTERLKLFAAETLKATEETFKLSYGIATVREVLSDRELYLSWEVGKPRPPLNRNYV     5497
MT040336.1      LANTCTERLKLFAAETLKATEETFKLSYGIATVREVLSDRELYLSWEVGKPRPPLNRNYV     5497
MT040335.1      LANTCTERLKLFAAETLKATEETFKLSYGIATVREVLSDRELYLSWEVGKPRPPLNRNYV     5497
MT040334.1      LANTCTERLKLFAAETLKATEETFKLSYGIATVREVLSDRELYLSWEVGKPRPTLNRNYV     5497
MT040333.1      LANTCTERLKLFAAETLKATEETFKLSYGIATVREVLSDRELYLSWEVGKPRPPLNRNYV     5497
MN996532.2      LANTCTERLKLFAAETLKATEETFKLSYGIATVREVLSDRELHLSWEVGKPRPPLNRNYV     5504
MN988713.1      LANTCTERLKLFAAETLKATEETFKLSYGIATVREVLSDRELHLSWEVGKPRPPLNRNYV     5505
MT093571.1      LANTCTERLKLFAAETLKATEETFKLSYGIATVREVLSDRELHLSWEVGKPRPPLNRNYV     5505
MN996529.1      LANTCTERLKLFAAETLKATEETFKLSYGIATVREVLSDRELHLSWEVGKPRPPLNRNYV     5505
MT072688.1      LANTCTERLKLFAAETLKATEETFKLSYGIATVREVLSDRELHLSWEVGKPRPPLNRNYV     5505
NC_045512.2     LANTCTERLKLFAAETLKATEETFKLSYGIATVREVLSDRELHLSWEVGKPRPPLNRNYV     5505
MN994467.1      LANTCTERLKLFAAETLKATEETFKLSYGIATVREVLSDRELHLSWEVGKPRPPLNRNYV     5505
MG772933.1      LANTCTERLKLFAAETLKATEETFKLSYGIATVREVLSDRELHLSWEVGKPRPPLNRNYV     5501
MG772934.1      LANTCTERLKLFAAETLKATEETFKLSYGIATVREVLSDRELYLSWEVGKPRPPLNRNYV     5479
NC_014470.1     LANTCTERLKLFAAETLKANEETFKLSYGIATVREVLSDRELHLSWEIGKPRPPLNRNYV     5473
GQ153542.1      LANTCTERLKLFAAETLKATEETFKLSYGIATVREVLSDRELYLSWEVGKPRPPLNRNYV     5480
DQ022305        LANTCTERLKLFAAETLKATEETFKLSYGIATVREVLSDRELYLSWEVGKPRPPLNRNYV     5476
KF569996.1      LANTCTERLKLFAAETLKATEETFKLSYGIATVREVLSDRELHLSWEVGKPRPPLNRNYV     5419
KP886809.1      LANTCTERLKLFAAETLKATEETFKLSYGIATVREVLSDRELHLSWEVGKPRPPLNRNYV     5482
AY278488.2      LANTCTERLKLFAAETLKATEETFKLSYGIATVREVLSDRELHLSWEVGKPRPPLNRNYV     5482
AY485277.1      LANTCTERLKLFAAETLKATEETFKLSYGIATVREVLSDRELHLSWEVGKPRPPLNRNYV     5482
AP006560.1      LANTCTERLKLFAAETLKATEETFKLSYGIATVREVLSDRELHLSWEVGKPRPPLNRNYV     5482
AP006557.1      LANTCTERLKLFAAETLKATEETFKLSYGIATVREVLSDRELHLSWEVGKPRPPLNRNYV     5482
AY274119        LANTCTERLKLFAAETLKATEETFKLSYGIATVREVLSDRELHLSWEVGKPRPPLNRNYV     5482
AY572038.1      LANTCTERLKLFAAETLKATEETFKLSYGIATVREVLSDRELHLSWEVGKPRPPLNRNYV     5482
AY572034.1      LANTCTERLKLFAAETLKATEETFKLSYGIATVREVLSDRELHLSWEVGKPRPPLNRNYV     5482
FJ588686.1      LANTCTERLKLFAAETLKATEETFKLSYGIATVREVLSDRELHLSWEVGKPRPPLNRNYV     5289
KY417145.1      LANTCTERLKLFAAETLKATEETFKLSYGIATVREVLSDRELHLSWEVGKPRPPLNRNYV     5482
KY417144.1      LANTCTERLKLFAAETLKATEETFKLSYGIATVREVLSDRELHLSWEVGKPKPPLNRNYV     5482
KY417147.1      LANTCTERLKLFAAETLKATEETFKLSYGIATVREVLSDRELHLSWEVGKPRPPLNRNYV     5482
KY417148.1      LANTCTERLKLFAAETLKATEETFKLSYGIATVREVLSDRELHLSWEVGKPRPPLNRNYV     5482
KY417143.1      LANTCTERLKLFAAETLKATEETFKLSYGIATVREVLSDRELHLSWEVGKPRPPLNRNYV     5482
KT444582.1      LANTCTERLKLFAAETLKATEETFKLSYGIATVREVLSDRELHLSWEVGKPRPPLNRNYV     5482
KC881005.1      LANTCTERLKLFAAETLKATEETFKLSYGIATVREVLSDRELHLSWEVGKPRPPLNRNYV     5482
KC881006.1      LANTCTERLKLFAAETLKATEETFKLSYGIATVREVLSDRELHLSWEVGKPRPPLNRNYV     5482
KF367457.1      LANTCTERLKLFAAETLKATEETFKLSYGIATVREVLSDRELHLSWEVGKPRPPLNRNYV     5482
KY417152.1      LANTCTERLKLFAAETLKATEETFKLSYGIATVREVLSDRELHLSWEVGKPRPPLNRNYV     5482
KY417146.1      LANTCTERLKLFAAETLKATEETFKLSYGIATVREVLSDRELHLSWEVGKPRPPLNRNYV     5482
KY417151.1      LANTCTERIKLFAAETLKATEETFKLSYGIATVREVLSDRELHLSWEVGKPRPPLNRNYV     5482
KY417142.1      LANTCTERLKLFAAETLKATEETFKLSYGIATVREVLSDRELHLSWEVGKPRPPLNRNYV     5482
                ********:**********.**********************:****:***:* ******
```

CoVAX_ORF1ab#11 CoVAX_ORF1ab#5

```
MW532698.1      FTGYRVTKNSKTQIGEYTFEKGDYGDAVVYRGTTTYKLNVGDYFVLTSHTVMPLSAPTLV   5557
MT040336.1      FTGYRVTKNSKTQIGEYTFEKGDYGDAVVYRGTTTYKLNVGDYFVLTSHTVMPLSAPTLV   5557
MT040335.1      FTGYRVTKNSKTQIGEYTFEKGDYGDAVVYRGTTTYKLNVGDYFVLTSHTVMPLSAPTLV   5557
MT040334.1      FTGYRVTKNSKTQIGEYTFEKGDYGDAVVYRGTTTYKLNVGDYFVLTSHTVMPLSAPTLV   5557
MT040333.1      FTGYRVTKNSKTQIGEYTFEKGDYGDAVVYRGTTTYKLNVGDYFVLTSHTVMPLSAPTLV   5557
MN996532.2      FTGYRVTKNSKVQIGEYTFEKGDYGDAVVYRGTTTYKLNVGDYFVLTSHTVMPLSAPTLV   5564
MN988713.1      FTGYRVTKNSKVQIGEYTFEKGDYGDAVVYRGTTTYKLNVGDYFVLTSHTVMPLSAPTLV   5565
MT093571.1      FTGYRVTKNSKVQIGEYTFEKGDYGDAVVYRGTTTYKLNVGDYFVLTSHTVMPLSAPTLV   5565
MN996529.1      FTGYRVTKNSKVQIGEYTFEKGDYGDAVVYRGTTTYKLNVGDYFVLTSHTVMPLSAPTLV   5565
MT072688.1      FTGYRVTKNSKVQIGEYTFEKGDYGDAVVYRGTTTYKLNVGDYFVLTSHTVMPLSAPTLV   5565
NC_045512.2     FTGYRVTKNSKVQIGEYTFEKGDYGDAVVYRGTTTYKLNVGDYFVLTSHTVMPLSAPTLV   5565
MN994467.1      FTGYRVTKNSKVQIGEYTFEKGDYGDAVVYRGTTTYKLNVGDYFVLTSHTVMPLSAPTLV   5565
MG772933.1      FTGYRVTKNSKVQIGEYTFEKGDYGDAVVYRGTTTYKLNVGDYFVLTSHTVMPLSAPTLV   5561
MG772934.1      FTGYRVTKNSKVQIGEYTFEKGDYGDAVVYRGTTTYKLNVGDYFVLTSHTVMPLSAPTLV   5539
NC_014470.1     FTGYRVTKNSKVQIGEYTFEKGDYGDAVVYRGTTTYKLNVGDYFVLTSHTVMPLTAPTLV   5533
GQ153542.1      FTGYRVTKNSKVQIGEYTFEKGDYGDAVVYRGTTTYKLNVGDYFVLTSHTVMPLSAPTLV   5540
DQ022305        FTGYRVTKNSKVQIGEYTFEKGDYGDAVVYRGTTTYKLNVGDYFVLTSHTVMPLSAPTLV   5536
KF569996.1      FTGYRVTKNSKVQIGEYTFEKGDYGDAVVYRGTTTYKLNVGDYFVLTSHTVMPLSAPTLV   5479
KP886809.1      FTGYRVTKNSKVQIGEYTFEKGDYGDAVVYRGTTTYKLNVGDYFVLTSHTVMPLSAPTLV   5542
AY278488.2      FTGYRVTKNSKVQIGEYTFEKGDYGDAVVYRGTTTYKLNVGDYFVLTSHTVMPLSAPTLV   5542
AY485277.1      FTGYRVTKNSKVQIGEYTFEKGDYGDAVVYRGTTTYKLNVGDYFVLTSHTVMPLSAPTLV   5542
AP006560.1      FTGYRVTKNSKVQIGEYTFEKGDYGDAVVYRGTTTYKLNVGDYFVLTSHTVMPLSAPTLV   5542
AP006557.1      FTGYRVTKNSKVQIGEYTFEKGDYGDAVVYRGTTTYKLNVGDYFVLTSHTVMPLSAPTLV   5542
AY274119        FTGYRVTKNSKVQIGEYTFEKGDYGDAVVYRGTTTYKLNVGDYFVLTSHTVMPLSAPTLV   5542
AY572038.1      FTGYRVTKNSKVQIGEYTFEKGDYGDAVVYRGTTTYKLNVGDYFVLTSHTVMPLSAPTLV   5542
AY572034.1      FTGYRVTKNSKVQIGEYTFEKGDYGDAVVYRGTTTYKLNVGDYFVLTSHTVMPLSAPTLV   5542
FJ588686.1      FTGYRVTKNSKVQIGEYTFEKGDYGDAVVYRGTTTYKLNVGDYFVLTSHTVMPLSAPTLV   5349
KY417145.1      FTGYRVTKNSKVQIGEYTFEKGDYGDAVVYRGTTTYKLNVGDYFVLTSHTVMPLSAPTLV   5542
KY417144.1      FTGYRVTKNSKVQIGEYTFEKGDYGDAVVYRGTTTYKLNVGDYFVLTSHTVMPLTAPTLV   5542
KY417147.1      FTGYRVTKNSKVQIGEYTFEKGDYGDAVVYRGTTTYKLNVGDYFVLTSHTVMPLSAPTLV   5542
KY417148.1      FTGYRVTKNSKVQIGEYTFEKGDYGDAVVYRGTTTYKLNVGDYFVLTSHTVMPLSAPTLV   5542
KY417143.1      FTGYRVTKNSKVQIGEYTFEKGDYGDAVVYRGTTTYKLNVGDYFVLTSHTVMPLSAPTLV   5542
KT444582.1      FTGYRVTKNSKVQIGEYTFEKGDYGDAVVYRGTTTYKLNVGDYFVLTSHTVMPLSAPTLV   5542
KC881005.1      FTGYRVTKNSKVQIGEYTFEKGDYGDAVVYRGTTTYKLNVGDYFVLTSHTVMPLSAPTLV   5542
KC881006.1      FTGYRVTKNSKVQIGEYTFEKGDYGDAVVYRGTTTYKLNVGDYFVLTSHTVMPLSAPTLV   5542
KF367457.1      FTGYRVTKNSKVQIGEYTFEKGDYGDAVVYRGTTTYKLNVGDYFVLTSHTVMPLSAPTLV   5542
KY417152.1      FTGYRVTKNSKVQIGEYTFEKGDYGDAVVYRGTTTYKLNVGDYFVLTSHTVMPLSAPTLV   5542
KY417146.1      FTGYRVTKNSKVQIGEYTFEKGDYGDAVVYRGTTTYKLNVGDYFVLTSHTVMPLSAPTLV   5542
KY417151.1      FTGYRVTKNSKVQIGEYTFEKGDYGDAVVYRGTTTYKLNVGDYFVLTSHTVMPLSAPTLV   5542
KY417142.1      FTGYRVTKNSKVQIGEYTFEKGDYGDAVVYRGTTTYKLNVGDYFVLTSHTVMPLSAPTLV   5542
                ***********.******************************************:*****
```

CoVAX_ORF1ab#5

```
MW532698.1    PQEHYVRITGLYPTLNISDEFSSNVANYQKVGMQKYSTLQGPPGTGKSHFAIGLALYYPS    5617
MT040336.1    PQEHYVRITGLYPTLNISDEFSSNVANYQKVGMQKYSTLQGPPGTGKSHFAIGLALYYPS    5617
MT040335.1    PQEHYVRITGLYPTLNISDEFSSNVANYQKVGMQKYSTLQGPPGTGKSHFAIGLALYYPS    5617
MT040334.1    PQEHYVRITGLYPTLNISDEFSSNVANYQKVGMQKYSTLQGPPGTGKSHFAIGLALYYPS    5617
MT040333.1    PQEHYVRITGLYPTLNISDEFSSNVANYQKVGMQKYSTLQGPPGTGKSHFAIGLALYYPS    5617
MN996532.2    PQEHYVRITGLYPTLNISDEFSSNVANYQKVGMQKYSTLQGPPGTGKSHFAIGLALYYPS    5624
MN988713.1    PQEHYVRITGLYPTLNISDEFSSNVANYQKVGMQKYSTLQGPPGTGKSHFAIGLALYYPS    5625
MT093571.1    PQEHYVRITGLYPTLNISDEFSSNVANYQKVGMQKYSTLQGPPGTGKSHFAIGLALYYPS    5625
MN996529.1    PQEHYVRITGLYPTLNISDEFSSNVANYQKVGMQKYSTLQGPPGTGKSHFAIGLALYYPS    5625
MT072688.1    PQEHYVRITGLYPTLNISDEFSSNVANYQKVGMQKYSTLQGPPGTGKSHFAIGLALYYPS    5625
NC_045512.2   PQEHYVRITGLYPTLNISDEFSSNVANYQKVGMQKYSTLQGPPGTGKSHFAIGLALYYPS    5625
MN994467.1    PQEHYVRITGLYPTLNISDEFSSNVANYQKVGMQKYSTLQGPPGTGKSHFAIGLALYYPS    5625
MG772933.1    PQEHYVRITGLYPTLNISDEFSSNVANYQKVGMQKYSTLQGPPGTGKSHFAIGLALYYPS    5621
MG772934.1    PQEHYVRITGLYPTLNISDEFSSNVANYQKVGMQKYSTLQGPPGTGKSHFAIGLALYYPS    5599
NC_014470.1   PQEHYVRITGLYPTLNISDEFSSNVANYQKVGMQKYSTLQGPPGTGKSHFAIGLALYYPS    5593
GQ153542.1    PQEHYVRITGLYPTLNISDEFSSNVANYQKVGMQKYSTLQGPPGTGKSHFAIGLALYYPS    5600
DQ022305      PQEHYVRITGLYPTLNISNEFSSNVANYQKIGMQKYSTLQGPPGTGKSHFAIGLALYYPS    5596
KF569996.1    PQEHYVRITGLYPTLNISDEFSSNVVNYQKVGMQKYSTLQGPPGTGKSHFAIGLALYYPS    5539
KP886809.1    PQEYYVRITGLYPTLNISDEFSSNVANYQKVGMQKYSTLQGPPGTGKSHFAIGLALYYPS    5602
AY278488.2    PQEHYVRITGLYPTLNISDEFSSNVANYQKVGMQKYSTLQGPPGTGKSHFAIGLALYYPS    5602
AY485277.1    SQEHYVRITGLYPTLNISDEFSSNVANYQKVGMQKYSTLQGPPGTGKSHFAIGLALYYPS    5602
AP006560.1    PQEHYVRITGLYPTLNISDEFSSNVANYQKVGMQKYSTLQGPPGTGKSHFAIGLALYYPS    5602
AP006557.1    PQEHYVRITGLYPTLNISDEFSSNVANYQKVGMQKYSTLQGPPGTGKSHFAIGLALYYPS    5602
AY274119      PQEHYVRITGLYPTLNISDEFSSNVANYQKVGMQKYSTLQGPPGTGKSHFAIGLALYYPS    5602
AY572038.1    PQEHYVRITGLYPTLNISDEFSSNVANYQKVGMQKYSTLQGPPGTGKSHFAIGLALYYPS    5602
AY572034.1    PQEHYVRITGLYPTLNISDEFSSNVANYQKVGMQKYSTLQGPPGTGKSHFAIGLALYYPS    5602
FJ588686.1    PQEHYVRITGLYPTLNISDEFSSNVANYQKVGMQKYSTLQGPPGTGKSHFAIGLALYYPS    5409
KY417145.1    PQEHYVRITGLYPTLNISDEFSSNVANYQKVGMQKYSTLQGPPGTGKSHFAIGLALYYPS    5602
KY417144.1    PQEHYVRITGLYPTLNISDDFSNNVANYQKVGMQKYSTLQGPPGTGKNHFAIGLALYYPS    5602
KY417147.1    PQEHYVRITGLYPTLNISDEFSSNVANYQKVGMQKYSTLQGPPGTGKSHFAIGLALYYPS    5602
KY417148.1    PQEHYVRITGLYPTLNISDEFSSNVANYQKVGMQKYSTLQGPPGTGKSHFAIGLALYYPS    5602
KY417143.1    PQEHYVRITGLYPTLNISDEFSSNVANYQKVGMQKYSTLQGPPGTGKSHFAIGLALYYPS    5602
KT444582.1    PQEHYVRITGLYPTLNISDEFSSNVANYQKVGMQKYSTLQGPPGTGKSHFAIGLALYYPS    5602
KC881005.1    PQEHYVRITGLYPTLNISDEFSSNVANYQKVGMQKYSTLQGPPGTGKSHFAIGLALYYPS    5602
KC881006.1    PQEHYVRITGLYPTLNISDEFSSNVANYQKVGMQKYSTLQGPPGTGKSHFAIGLALYYPS    5602
KF367457.1    PQEHYVRITGLYPTLNISDEFSSNVANYQKVGMQKYSTLQGPPGTGKSHFAIGLALYYPS    5602
KY417152.1    PQEHYVRITGLYPTLNISDEFSSNVANYQKVGMQKYSTLQGPPGTGKSHFAIGLALYYPS    5602
KY417146.1    PQEHYVRITGLYPTLNISDEFSSNVANYQKVGMQKYSTLQGPPGTGKSHFAIGLALYYPS    5602
KY417151.1    PQEHYVRITGLYPTLNISDEFSSNVANYQKVGMQKYSTLQGPPGTGKSHFAIGLALYYPS    5602
KY417142.1    PQEHYVRITGLYPTLNISDEFSSNVANYQKVGMQKYSTLQGPPGTGKSHFAIGLALYYPS    5602
               **:**************::**.**.****:****************.************
```

CoVAX_ORF1ab#2

MW532698.1	ARIVYTACSHAAVDALCEKALKYLPIDKCSRIIPARARVECFDKFKVNSTLEQYVFCTVN	5677
MT040336.1	ARIVYTACSHAAVDALCEKALKYLPIDKCSRIIPARARVECFDKFKVNSTLEQYVFCTVN	5677
MT040335.1	ARIVYTACSHAAVDALCEKALKYLPIDKCSRIIPARARVECFDKFKVNSTLEQYVFCTVN	5677
MT040334.1	ARIVYTACSHAAVDALCEKALKYLPIDKCSRIIPARARVECFDKFKVNSTLEQYVFCTVN	5677
MT040333.1	ARIVYTACSHAAVDALCEKALKYLPIDKCSRIIPARARVECFDKFKVNSTLEQYVFCTVN	5677
MN996532.2	ARIVYTACSHAAVDALCEKALKYLPIDKCSRIIPARARVECFDKFKVNSTLEQYVFCTVN	5684
MN988713.1	ARIVYTACSHAAVDALCEKALKYLPIDKCSRIIPARARVECFDKFKVNSTLEQYVFCTVN	5685
MT093571.1	ARIVYTACSHAAVDALCEKALKYLPIDKCSRIIPARARVECFDKFKVNSTLEQYVFCTVN	5685
MN996529.1	ARIVYTACSHAAVDALCEKALKYLPIDKCSRIIPARARVECFDKFKVNSTLEQYVFCTVN	5685
MT072688.1	ARIVYTACSHAAVDALCEKALKYLPIDKCSRIIPARARVECFDKFKVNSTLEQYVFCTVN	5685
**NC_045512.2**	**ARIVYTACSHAAVDALCEKALKYLPIDKCSRIIPARARVECFDKFKVNSTLEQYVFCTVN**	**5685**
MN994467.1	ARIVYTACSHAAVDALCEKALKYLPIDKCSRIIPARARVECFDKFKVNSTLEQYVFCTVN	5685
MG772933.1	ARIVYTACSHAAVDALCEKALKYLPIDKCSRIIPARARVECFDKFKVNSTLEQYVFCTVN	5681
MG772934.1	ARIVYTACSHAAVDALCEKALKYLPIDKCSRIIPARARVECFDKFKVNSTLEQYVFCTVN	5659
NC_014470.1	ARIVYTACSHAAVDALCEKALKYLPIDKCSRIIPARARVECFDKFKVNSTLEQYVFCTVN	5653
GQ153542.1	ARIVYTACSHAAVDALCEKALKYLPIDKCSRIIPARARVECFDKFKVNSTLEQYVFCTVN	5660
DQ022305	ARIVYTACSHAAVDALCEKALKYLPIDKCSRIIPARARVECFDKFKVNSTLEQYVFCTVN	5656
KF569996.1	ARIVYTACSHAAVDALCEKALKYLPIDKCSRIIPARARVECFDKFKVNSTLEQYVFCTVN	5599
KP886809.1	ARIVYTACSHAAVDALCEKALKYLPIDKCSRIIPARARVECFDKFKVNSTLEQYVFCTVN	5662
AY278488.2	ARIVYTACSHAAVDALCEKALKYLPIDKCSRIIPARARVECFDKFKVNSTLEQYVFCTVN	5662
AY485277.1	ARIVYTACSHAAVDALCEKALKYLPIDKCSRIIPARARVECFDKFKVNSTLEQYVFCTVN	5662
AP006560.1	ARIVYTACSHAAVDALCEKALKYLPIDKCSRIIPARARVECFDKFKVNSTLEQYVFCTVN	5662
AP006557.1	ARIVYTACSHAAVDALCEKALKYLPIDKCSRIIPARARVECFDKFKVNSTLEQYVFCTVN	5662
AY274119	ARIVYTACSHAAVDALCEKALKYLPIDKCSRIIPARARVECFDKFKVNSTLEQYVFCTVN	5662
AY572038.1	ARIVYTACSHAAVDALCEKALKYLPIDKCSRIIPARARVECFDKFKVNSTLEQYVFCTVN	5662
AY572034.1	ARIVYTACSHAAVDALCEKALKYLPIDKCSRIIPARARVECFDKFKVNSTLEQYVFCTVN	5662
FJ588686.1	ARIVYTACSHAAVDALCEKALKYLPIDKCSRIIPARARVECFDKFKVNSTLEQYVFCTVN	5469
KY417145.1	ARIVYTACSHAAVDALCEKALKYLPIDKCSRIIPARARVECFDKFKVNSTLEQYVFCTVN	5662
KY417144.1	ARIVYTACSHAAVDALCEKALKYLPIDKCSRIIPARARVECFDKFKVNSTLEQYVFCTVN	5662
KY417147.1	ARIVYTACSHAAVDALCEKALKYLPIDKCSRIIPARARVECFDKFKVNSTLEQYVFCTVN	5662
KY417148.1	ARIVYTACSHAAVDALCEKALKYLPIDKCSRIIPARARVECFDKFKVNSTLEQYVFCTVN	5662
KY417143.1	ARIVYTACSHAAVDALCEKALKYLPIDKCSRIIPARARVECFDKFKVNSTLEQYVFCTVN	5662
KT444582.1	ARIVYTACSHAAVDALCEKALKYLPIDKCSRIIPARARVECFDKFKVNSTLEQYVFCTVN	5662
KC881005.1	ARIVYTACSHAAVDALCEKALKYLPIDKCSRIIPARARVECFDKFKVNSTLEQYVFCTVN	5662
KC881006.1	ARIVYTACSHAAVDALCEKALKYLPIDKCSRIIPARARVECFDKFKVNSTLEQYVFCTVN	5662
KF367457.1	ARIVYTACSHAAVDALCEKALKYLPIDKCSRIIPARARVECFDKFKVNSTLEQYVFCTVN	5662
KY417152.1	ARIVYTACSHAAVDALCEKALKYLPIDKCSRIIPARARVECFDKFKVNSTLEQYVFCTVN	5662
KY417146.1	ARIVYTACSHAAVDALCEKALKYLPIDKCSRIIPARARVECFDKFKVNSTLEQYVFCTVN	5662
KY417151.1	ARIVYTACSHAAVDALCEKALKYLPIDKCSRIIPARARVECFDKFKVNSTLEQYVFCTVN	5662
KY417142.1	ARIVYTACSHAAVDALCEKALKYLPIDKCSRIIPARARVECFDKFKVNSTLEQYVFCTVN	5662
	**	

CoVAX_ORF1ab#2 CoVAX_ORF1ab#3

```
MW532698.1      ALPEATADIVVFDEISMATNYDLSVVNARLRAKHYVYIGDPAQLPAPRTLLTKGTLEPEY     5737
MT040336.1      ALPETTADIVVFDGISMATNYDLSVVNARLRAKHYVYIGDPAQLPAPRTLLTKGTLEPEY     5737
MT040335.1      ALPETTADIVVFDEISMATNYDLSVVNARLRAKHYVYIGDPAQLPAPRTLLTKGTLEPEY     5737
MT040334.1      ALPETTADIVVFDEISMATNYDLSVVNARLRAKHYVYIGDPAQLPAPRTLLTKGTLEPEY     5737
MT040333.1      ALPETTADIVVFDEISMATNYDLSVVNARLRAKHYVYIGDPAQLPAPRTLLTKGTLEPEY     5737
MN996532.2      ALPETTADIVVFDEISMATNYDLSVVNARLRAKHYVYIGDPAQLPAPRTLLTKGTLEPEY     5744
MN988713.1      ALPETTADIVVFDEISMATNYDLSVVNARLRAKHYVYIGDPAQLPAPRTLLTKGTLEPEY     5745
MT093571.1      ALPETTADIVVFDEISMATNYDLSVVNARLRAKHYVYIGDPAQLPAPRTLLTKGTLEPEY     5745
MN996529.1      ALPETTADIVVFDEISMATNYDLSVVNARLRAKHYVYIGDPAQLPAPRTLLTKGTLEPEY     5745
MT072688.1      ALPETTADIVVFDEISMATNYDLSVVNARLRAKHYVYIGDPAQLPAPRTLLTKGTLEPEY     5745
NC_045512.2     ALPETTADIVVFDEISMATNYDLSVVNARLRAKHYVYIGDPAQLPAPRTLLTKGTLEPEY     5745
MN994467.1      ALPETTADIVVFDEISMATNYDLSVVNARLRAKHYVYIGDPAQLPAPRTLLTKGTLEPEY     5745
MG772933.1      ALPETTADIVVFDEISMATNYDLSVVNARLRAKHYVYIGDPAQLPAPRTLLTKGTLEPEY     5741
MG772934.1      ALPETTADIVVFDEISMATNYDLSVVNARLRAKHYVYIGDPAQLPAPRTLLTKGTLEPEY     5719
NC_014470.1     ALPETTADIVVFDEVSMATNYDLSVVNARLRAKHYVYIGDPAQLPAPRTLLTKGTLEPEY     5713
GQ153542.1      ALPETTADIVVFDEISMATNYDLSVVNARLRAKHYVYIGDPAQLPAPRTLLTKGTLEPEY     5720
DQ022305        ALPETTADIVVFDEISMATNYDLSVVNARLRAKHYVYIGDPAQLPAPRTLLTKGTLEPEY     5716
KF569996.1      ALPETTADIVVFDEISMATNYDLSVVNARLRAKHYVYIGDPAQLPAPRTLLTKGTLEPEY     5659
KP886809.1      ALPETTADIVVFDEISMATNYDLSVVNARLRAKHYVYIGDPAQLPAPRTLLTKGTLEPEY     5722
AY278488.2      ALPETTADIVVFDEISMATNYDLSVVNARLRAKHYVYIGDPAQLPAPRTLLTKGTLEPEY     5722
AY485277.1      ALPETTADIVVFDEISMATNYDLSVVNARLRAKHYVYIGDPAQLPAPRTLLTKGTLEPEY     5722
AP006560.1      ALPETTADIVVFDEISMATNYDLSVVNARLRAKHYVYIGDPAQLPAPRTLLTKGTLEPEY     5722
AP006557.1      ALPETTADIVVFDEISMATNYDLSVVNARLRAKHYVYIGDPAQLPAPRTLLTKGTLEPEY     5722
AY274119        ALPETTADIVVFDEISMATNYDLSVVNARLRAKHYVYIGDPAQLPAPRTLLTKGTLEPEY     5722
AY572038.1      ALPETTADIVVFDEISMATNYDLSVVNARLRAKHYVYIGDPVQLPAPRTLLTKGTLEPEY     5722
AY572034.1      ALPETTADIVVFDEISMATNYDLSVVNARLRAKHYVYIGDPAQLPAPRTLLTKGTLEPEY     5722
FJ588686.1      ALPETTADIVVFDEISMATNYDLSVVNARLRAKHYVYIGDPAQLPAPRTLLTKGTLEPEY     5529
KY417145.1      ALPETTADIVVFDEISMATNYDLSVVNARLRAKHYVYIGDPAQLPAPRTLLTKGTLEPEY     5722
KY417144.1      ALPETTADIVVFDEISMATNYDLSVVNARLRAKHYVYIGDPAQLPAPRTLLTKGTLEPEY     5722
KY417147.1      ALPETTADIVVFDEISMATNYDLSVVNARLRAKHYVYIGDPAQLPAPRTLLTKGTLEPEY     5722
KY417148.1      ALPETTADIVVFDEISMATNYDLSVVNARLRAKHYVYIGDPAQLPAPRTLLTKGTLEPEY     5722
KY417143.1      ALPETTADIVVFDEISMATNYDLSVVNARLRAKHYVYIGDPAQLPAPRTLLTKGTLEPEY     5722
KT444582.1      ALPETTADIVVFDEISMATNYDLSVVNARLRAKHYVYIGDPAQLPAPRTLLTKGTLEPEY     5722
KC881005.1      ALPETTADIVVFDEISMATNYDLSVVNARLRAKHYVYIGDPAQLPAPRTLLTKGTLEPEY     5722
KC881006.1      ALPETTADIVVFDEISMATNYDLSVVNARLRAKHYVYIGDPAQLPAPRTLLTKGTLEPEY     5722
KF367457.1      ALPETTADIVVFDEISMATNYDLSVVNARLRAKHYVYIGDPAQLPAPRTLLTKGTLEPEY     5722
KY417152.1      ALPETTADIVVFDEISMATNYDLSVVNARLRAKHYVYIGDPAQLPAPRTLLTKGTLEPEY     5722
KY417146.1      ALPETTADIVVFDEISMATNYDLSVVNARLRAKHYVYIGDPAQLPAPRTLLTKGTLEPEY     5722
KY417151.1      ALPETTADIVVFDEISMATNYDLSVVNARLRAKHYVYIGDPAQLPAPRTLLTKGTLEPEY     5722
KY417142.1      ALPETTADIVVFDEISMATNYDLSVVNARLRAKHYVYIGDPAQLPAPRTLLTKGTLEPEY     5722
                ****:******** :**************************.******************
```

CoVAX_ORF1ab#3

MW532698.1	FNSVCRLMKTIGPDMFLGTCRRCPAEIVDTVSALVYDNKLRAHKDKSQQCFKMFYKGVIT	5797
MT040336.1	FNSVCRLMKTIGPDMFLGTCRRCPAEIVDTVSALVYDNKLRAHKDKSQQCFKMFYKGVIT	5797
MT040335.1	FNSVCRLMKTIGPDMFLGTCRRCPAEIVDTVSALVYDNKLRAHKDKSQQCFKMFYKGVIT	5797
MT040334.1	FNSVCRLMKTIGPDMFLGTCRRCPAEIVDTVSALVYDNKLRAHKDKSQQCFKMFYKGVIT	5797
MT040333.1	FNSVCRLMKTIGPDMFLGTCRRCPAEIVDTVSALVYDNKLRAHKDKSQQCFKMFYKGVIT	5797
MN996532.2	FNSVCRLMKTIGPDMFLGTCRRCPAEIVDTVSALVYDNKLKAHKDKSAQCFKMFYKGVIT	5804
MN988713.1	FNSVCRLMKTIGPDMFLGTCRRCPAEIVDTVSALVYDNKLKAHKDKSAQCFKMFYKGVIT	5805
MT093571.1	FNSVCRLMKTIGPDMFLGTCRRCPAEIVDTVSALVYDNKLKAHKDKSAQCFKMFYKGVIT	5805
MN996529.1	FNSVCRLMKTIGPDMFLGTCRRCPAEIVDTVSALVYDNKLKAHKDKSAQCFKMFYKGVIT	5805
MT072688.1	FNSVCRLMKTIGPDMFLGTCRRCPAEIVDTVSALVYDNKLKAHKDKSAQCFKMFYKGVIT	5805
**NC_045512.2**	**FNSVCRLMKTIGPDMFLGTCRRCPAEIVDTVSALVYDNKLKAHKDKSAQCFKMFYKGVIT**	**5805**
MN994467.1	FNSVCRLMKTIGPDMFLGTCRRCPAEIVDTVSALVYDNKLKAHKDKSAQCFKMFYKGVIT	5805
MG772933.1	FNSVCRLMKTIGPDMFLGTCRRCPAEIVDTVSALVYDNKLKAHKEKSAQCFKMFYKGVIT	5801
MG772934.1	FNSVCRLMKTIGPDMFLGTCRRCPAEIVDTVSALVYDNKLKAHKEKSAQCFKMFYKGVIT	5779
NC_014470.1	FNSVCRLMKTIGPDMFLGTCRRCPAEIVDTVSALVYDNKLRAHKGKSSQCFKMFYKGVIT	5773
GQ153542.1	FNSVCRLMKTIGPDMFLGTCRRCPAEIVDTVSALVYDNKLKAHKEKSAQCFKMYYKGVIT	5780
DQ022305	FNSVCRLMKTIGPDMFLGTCRRCPAEIVDTVSALVYDNKLKAHKEKSAQCFKMYYKGVIT	5776
KF569996.1	FNSVCRLMKTIGPDMFLGTCRRCPAEIVDTVSALVYDNKLRAHKEKSAQCFKMFYKGVIT	5719
KP886809.1	FNSVCRLMKTIGPDMFLGTCRRCPAEIVDTVSALVYDNKLKAHKEKSAQCFKMFYKGVIT	5782
AY278488.2	FNSVCRLMKTIGPDMFLGTCRRCPAEIVDTVSALVYDNKLKAHKEKSAQCFKMFYKGVIT	5782
AY485277.1	FNSVCRLMKTIGPDMFLGTCRRCPAEIVDTVSALVYDNKLKAHKDKSAQCFKMFYKGVIT	5782
AP006560.1	FNSVCRLMKTIGPDMFLGTCRRCPAEIVDTVSALVYDNKLKAHKDKSAQCFKMFYKGVIT	5782
AP006557.1	FNSVCRLMKTIGPDMFLGTCRRCPAEIVDTVSALVYDNKLKAHKDKSAQCFKMFYKGVIT	5782
AY274119	FNSVCRLMKTIGPDMFLGTCRRCPAEIVDTVSALVYDNKLKAHKDKSAQCFKMFYKGVIT	5782
AY572038.1	FNSVCRLMKTIGPDMFLGTCRRCPAEIVDTVSALVYDNKLKAHKEKSAQCFKMFYKGVIT	5782
AY572034.1	FNSVCRLMKTIGPDMFLGTCRRCPAEIVDTVSALVYDNKLKAHKEKSAQCFKMFYKGVIT	5782
FJ588686.1	FNSVCRLMKTIGPDMFLGTCRRCPAEIVDTVSALVYDNKLRAHKEKSAQCFKMFYKGVIT	5589
KY417145.1	FNSVCRLMKTIGPDMFLGTCRRCPAEIVDTVSALVYDNKLKAHKDKSAQCFKMFYKGVIT	5782
KY417144.1	FNSVCRLMKTIGPDMFLGTCRRCPAEIVDTVSALVYDNKLKAHKEKSAQCFKMFYKGVIT	5782
KY417147.1	FNSVCRLMKTIGPDMFLGTCRRCPAEIVDTVSALVYDNKLKAHKDKSAQCFKMFYKGVIT	5782
KY417148.1	FNSVCRLMKTIGPDMFLGTCRRCPAEIVDTVSALVYDNKLKAHKEKSAQCFKMFYKGVIT	5782
KY417143.1	FNSVCRLMKTIGPDMFLGTCRRCPAEIVDTVSALVYDNKLKAHKEKSAQCFKMFYKGVIT	5782
KT444582.1	FNSVCRLMKTIGPDMFLGTCRRCPAEIVDTVSALVYDNKLKAHKEKSAQCFKMFYKGVIT	5782
KC881005.1	FNSVCRLMKTIGPDMFLGTCRRCPAEIVDTVSALVYDNKLKAHKEKSAQCFKMFYKGVIT	5782
KC881006.1	FNSVCRLMKTIGPDMFLGTCRRCPAEIVDTVSALVYDNKLKAHKEKSAQCFKMFYKGVIT	5782
KF367457.1	FNSVCRLMKTIGPDMFLGTCRRCPAEIVDTVSALVYDNKLKAHKEKSAQCFKMFYKGVIT	5782
KY417152.1	FNSVCRLMKTIGPDMFLGTCRRCPAEIVDTVSALVYDNKLKAHKEKSAQCFKMFYKGVIT	5782
KY417146.1	FNSVCRLMKTIGPDMFLGTCRRCPAEIVDTVSALVYDNKLKAHKEKSAQCFKMFYKGVIT	5782
KY417151.1	FNSVCRLMKTIGPDMFLGTCRRCPAEIVDTVSALVYDNKLKAHKEKSAQCFKMFYKGVIT	5782
KY417142.1	FNSVCRLMKTIGPDMFLGTCRRCPAEIVDTVSALVYDNKLKAHKEKSAQCFKMFYKGVIT	5782
	:* ** *****:******	

MW532698.1	HDVSSAINRPQIGVVREFLTRNPTWRKAVFISPYNSQNAVAAKILGLPTQTVDSSQGSEY	5857
MT040336.1	HDVSSAINRPQIGVVREFLTRNPTWRKAVFISPYNSQNAVAAKILGLPTQTVDSSQGSEY	5857
MT040335.1	HDVSSAINRPQIGVVREFLTRNPTWRKAVFISPYNSQNAVAAKILGLPTQTVDSSQGSEY	5857
MT040334.1	HDVSSAINRPQIGVVREFLTRNPTWRKAVFISPYNSQNAVAAKILGLPTQTVDSSQGSEY	5857
MT040333.1	HDVSSAINRPQIGVVREFLTRNPTWRKAVFISPYNSQNAVAAKILGLPTQTVDSSQGSEY	5857
MN996532.2	HDVSSAINRPQIGVVREFLTRNPTWRKAVFISPYNSQNAVASKILGLPTQTVDSSQGSEY	5864
MN988713.1	HDVSSAINRPQIGVVREFLTRNPAWRKAVFISPYNSQNAVASKILGLPTQTVDSSQGSEY	5865
MT093571.1	HDVSSAINRPQIGVVREFLTRNPAWRKAVFISPYNSQNAVASKILGLPTQTVDSSQGSEY	5865
MN996529.1	HDVSSAINRPQIGVVREFLTRNPAWRKAVFISPYNSQNAVASKILGLPTQTVDSSQGSEY	5865
MT072688.1	HDVSSAINRPQIGVVREFLTRNPAWRKAVFISPYNSQNAVASKILGLPTQTVDSSQGSEY	5865
**NC_045512.2**	**HDVSSAINRPQIGVVREFLTRNPAWRKAVFISPYNSQNAVASKILGLPTQTVDSSQGSEY**	**5865**
MN994467.1	HDVSSAINRPQIGVVREFLTRNPAWRKAVFISPYNSQNAVASKILGLPTQTVDSSQGSEY	5865
MG772933.1	HDVSSAINRPQIGVVREFLTRNPAWRKAVFISPYNSQNAVASKILGLPTQTVDSSQGSEY	5861
MG772934.1	HDVSSAINRPQIGVVREFLTRNPAWRKAVFISPYNSQNTVASKILGLPTQTVDSSQGSEY	5839
NC_014470.1	HDVSSAINRPQIGVVREFLTRNPAWRKAVFISPYNSQNAVASKILGLPTQTVDSSQGSEY	5833
GQ153542.1	HDVSSAINRPQIGVVREFLTRNPAWRKAVFISPYNSQNAVASKILGLPTQTVDSSQGSEY	5840
DQ022305	HDVSSAINRPQIGVVREFLTRNPAWRKAVFISPYNSQNAVASKILGLPTQTVDSSQGSEY	5836
KF569996.1	HDVSSAINRPQIGVVREFLTRNPAWRKAVFISPYNSQNAVAAKILGLPTQTVDSSQGSEY	5779
KP886809.1	HDVSSAINRPQIGVVREFLTRNPAWRKAVFISPYNSQNAVASKILGLPTQTVDSSQGSEY	5842
AY278488.2	HDVSSAINRPQIGVVREFLTRNPAWRKAVFISPYNSQNAVASKILGLPTQTVDSSQGSEY	5842
AY485277.1	HDVSSAINRPQIGVVREFLTRNPAWRKAVFISPYNSQNAVASKILGLPTQTVDSSQGSEY	5842
AP006560.1	HDVSSAINRPQIGVVREFLTRNPAWRKAVFISPYNSQNAVASKILGLPTQTVDSSQGSEY	5842
AP006557.1	HDVSSAINRPQIGVVREFLTRNPAWRKAVFISPYNSQNAVASKILGLPTQTVDSSQGSEY	5842
AY274119	HDVSSAINRPQIGVVREFLTRNPAWRKAVFISPYNSQNAVASKILGLPTQTVDSSQGSEY	5842
AY572038.1	HDVSSAINRPQIGVVREFLTRNPAWRKAVFISPYNSQNAVASKILGLPTQTVDSSQGSEY	5842
AY572034.1	HDVSSAINRPQIGVVREFLTRNPAWRKAVFISPYNSQNAVASKILGLPTQTVDSSQGSEY	5842
FJ588686.1	HDVSSAINRPQIGVVREFLTRNPAWRKAVFISPYNSQNAVASKILGLPTQTVDSSQGSEY	5649
KY417145.1	HDVSSAINRPQIGVVREFLTRNPAWRKAVFISPYNSQNAVASKILGLPTQTVDSSQGSEY	5842
KY417144.1	HDVSSAINRPQIGVVREFLTRNPAWRKAVFISPYNSQNAVASKILGLPTQTVDSSQGSEY	5842
KY417147.1	HDVSSAINRPQIGVVREFLTRNPAWRKAVFISPYNSQNAVASKILGLPTQTVDSSQGSEY	5842
KY417148.1	HDVSSAINRPQIGVVREFLTRNPAWRKAVFISPYNSQNAVASKILGLPTQTVDSSQGSEY	5842
KY417143.1	HDVSSAINRPQIGVVREFLTRNPVWRKAVFISPYNSQNAVASKILGLPTQTVDSSQGSEY	5842
KT444582.1	HDVSSAINRPQIGVVREFLTRNPAWRKAVFISPYNSQNAVASKILGLPTQTVDSSQGSEY	5842
KC881005.1	HDVSSAINRPQIGVVREFLTRNPAWRKAVFISPYNSQNAVASKILGLPTQTVDSSQGSEY	5842
KC881006.1	HDVSSAINRPQIGVVREFLTRNPAWRKAVFISPYNSQNAVASKILGLPTQTVDSSQGSEY	5842
KF367457.1	HDVSSAINRPQIGVVREFLTRNPAWRKAVFISPYNSQNAVASKILGLPTQTVDSSQGSEY	5842
KY417152.1	HDVSSAINRPQIGVVREFLTRNPAWRKAVFISPYNSQNAVASKILGLPTQTVDSSQGSEY	5842
KY417146.1	HDVSSAINRPQIGVVREFLTRNPAWRKAVFISPYNSQNAVASKILGLPTQTVDSSQGSEY	5842
KY417151.1	HDVSSAINRPQIGVVREFLTRNPAWRKAVFISPYNSQNAVASKILGLPTQTVDSSQGSEY	5842
KY417142.1	HDVSSAINRPQIGVVREFLTRNPAWRKAVFISPYNSQNAVASKILGLPTQTVDSSQGSEY	5842
	***********************.**************:**:******************	

MW532698.1	DYVIFTQTTETAHSCNVNRFNVAITRAKIGILCIMSDRDLYDKLQFTSLEVPRRNVATLQ	5917
MT040336.1	DYVIFTQTTETAHSCNVNRFNVAITRAKIGILCIMSDRDLYDKLQFTSLEVPRRNVATLQ	5917
MT040335.1	DYVIFTQTTETAHSCNVNRFNVAITRAKIGILCIMSDRDLYDKLQFTSLEVPRRNVATLQ	5917
MT040334.1	DYVIFTQTTETAHSCNVNRFNVAITRAKIGILCIMSDRDLYDKLQFTSLEVPRRNVATLQ	5917
MT040333.1	DYVIFTQTTETAHSCNVNRFNVAITRAKIGILCIMSDRDLYDKLQFTSLEVPRRNVATLQ	5917
MN996532.2	DYVIFTQTTETAHSCNVNRFNVAITRAKVGILCIMSDRDLYDKLQFTSLEIPRRNVATLQ	5924
MN988713.1	DYVIFTQTTETAHSCNVNRFNVAITRAKVGILCIMSDRDLYDKLQFTSLEIPRRNVATLQ	5925
MT093571.1	DYVIFTQTTETAHSCNVNRFNVAITRAKVGILCIMSDRDLYDKLQFTSLEIPRRNVATLQ	5925
MN996529.1	DYVIFTQTTETAHSCNVNRFNVAITRAKVGILCIMSDRDLYDKLQFTSLEIPRRNVATLQ	5925
MT072688.1	DYVIFTQTTETAHSCNVNRFNVAITRAKVGILCIMSDRDLYDKLQFTSLEIPRRNVATLQ	5925
**NC_045512.2**	**DYVIFTQTTETAHSCNVNRFNVAITRAKVGILCIMSDRDLYDKLQFTSLEIPRRNVATLQ**	**5925**
MN994467.1	DYVIFTQTTETAHSCNVNRFNVAITRAKVGILCIMSDRDLYDKLQFTSLEIPRRNVATLQ	5925
MG772933.1	DYVIFTQTTETAHSCNVNRFNVAITRAKIGILCIMSDRDLYDKLQFMSLEVPRRNVATLQ	5921
MG772934.1	DYVIFTQTTETAHSCNVNRFNVAITRAKIGILCIMSDRDLYDKLQFTSLEVPRRNVATLQ	5899
NC_014470.1	DYVIFAQTTETAHSCNVNRFNVAITRAKVGILCIMSDKDLYDKLQFTSLEVPRRSVAVLQ	5893
GQ153542.1	DYVIFTQTTETAHSCNVNRFNVAITRAKIGILCIMSDRDLYDKLQFTSLEIPRRNVATLQ	5900
DQ022305	DYVIFTQTTETAHSCNVNRFNVAITRAKIGILCIMSDRDLYDKLQFTSLEVPRRNVATLQ	5896
KF569996.1	DYVIFTQTTETAHSCNVNRFNVAITRAKIGILCIMSDRDLYDKLQFTSLEVPRRNVATLQ	5839
KP886809.1	DYVIFTQTTETAHSCNVNRFNVAITRAKIGILCIMSDRDLYDKLQFTSLEVPRRNVATLQ	5902
AY278488.2	DYVIFTQTTETAHSCNVNRFNVAITRAKIGILCIMSDRDLYDKLQFTSLEIPRRNVATLQ	5902
AY485277.1	DYVIFTQTTETAHSCNVNRFNVAITRAKIGILCIMSDRDLYDKLQFTSLEIPRRNVATLQ	5902
AP006560.1	DYVIFTQTTETAHSCNVNRFNVAITRAKIGILCIMSDRDLYDKLQFTSLEIPRRNVATLQ	5902
AP006557.1	DYVIFTQTTETAHSCNVNRFNVAITRAKIGILCIMSDRDLYDKLQFTSLEIPRRNVATLQ	5902
AY274119	DYVIFTQTTETAHSCNVNRFNVAITRAKIGILCIMSDRDLYDKLQFTSLEIPRRNVATLQ	5902
AY572038.1	DYVIFTQTTETAHSCNVNRFNVAITRAKIGILCIMSDRDLYDKLQFTSLEIPRRNVATLQ	5902
AY572034.1	DYVIFTQTTETAHSCNVNRFNVAITRAKIGILCIMSDRDLYDKLQFTSLEIPRRNVATLQ	5902
FJ588686.1	DYVIFTQTTETAHSCNVNRFNVAITRAKIGILCIMSDRDLYDKLQFTSLEVPRRNVATLQ	5709
KY417145.1	DYVIFTQTTETAHSCNVNRFNVAITRAKIGILCIMSDRDLYDKLQFTSLEVPRRNVATLQ	5902
KY417144.1	DYVIFTQTTETAHSCNVNRFNVAITRAKIGILCIMSDRDLYDKLQFTSLEVPRRNVATLQ	5902
KY417147.1	DYVIFTQTTETAHSCNVNRFNVAITRAKIGILCIMSDRDLYDKLQFTSLEVPRRNVATLQ	5902
KY417148.1	DYVIFTQTTETAHSCNVNRFNVAITRAKIGILCIMSDRDLYDKLQFTSLEVPRRNVATLQ	5902
KY417143.1	DYVIFTQTTETAHSCNVNRFNVAITRAKIGILCIMSDRDLYDKLQFTSLEVPRRNVATLQ	5902
KT444582.1	DYVIFTQTTETAHSCNVNRFNVAITRAKIGILCIMSDRDLYDKLQFTSLEVPRRNVATLQ	5902
KC881005.1	DYVIFTQTTETAHSCNVNRFNVAITRAKIGILCIMSDRDLYDKLQFTSLEVPRRNVATLQ	5902
KC881006.1	DYVIFTQTTETAHSCNVNRFNVAITRAKIGILCIMSDRDLYDKLQFTSLEVPRRNVATLQ	5902
KF367457.1	DYVIFTQTTETAHSCNVNRFNVAITRAKIGILCIMSDRDLYDKLQFTSLEVPRRNVATLQ	5902
KY417152.1	DYVIFTQTTETAHSCNVNRFNVAITRAKIGILCIMSDRDLYDKLQFTSLEVPRRNVATLQ	5902
KY417146.1	DYVIFTQTTETAHSCNVNRFNVAITRAKIGILCIMSDRDLYDKLQFTSLEVPRRNVATLQ	5902
KY417151.1	DYVIFTQTTETAHSCNVNRFNVAITRAKIGILCIMSDRDLYDKLQFTSLEVPRRNVATLQ	5902
KY417142.1	DYVIFTQTTETAHSCNVNRFNVAITRAKIGILCIMSDRDLYDKLQFTSLEVPRRNVATLQ	5902
	*****:**********************:********:******** ***:***.**.**	

MW532698.1	AENVTGLFKDCSKVITGLHPTQAPTYLSVDTKFKTEGLCVDIPGIPKDMTYRRLISMMGF	5977
MT040336.1	AENVTGLFKDCSKVITGLHPTQAPTYLSVDTKFKTEGLCVDIPGIPKDMTYRRLISMMGF	5977
MT040335.1	AENVTGLFKDCSKVITGLHPTQAPTYLSVDTKFKTEGLCVDIPGIPKDMTYRRLISMMGF	5977
MT040334.1	AENVTGLFKDCSKVITGLHPTQAPTYLSVDTKFKTEGLCVDIPGIPKDMTYRRLISMMGF	5977
MT040333.1	AENVTGLFKDCSKVITGLHPTQAPTYLSVDTKFKTEGLCVDIPGIPKDMTYRRLISMMGF	5977
MN996532.2	AENVTGLFKDCSKVITGLHPTQAPTHLSVDTKFKTEGLCVDIPGIPKDMTYRRLISMMGF	5984
MN988713.1	AENVTGLFKDCSKVITGLHPTQAPTHLSVDTKFKTEGLCVDIPGIPKDMTYRRLISMMGF	5985
MT093571.1	AENVTGLFKDCSKVITGLHPTQAPTHLSVDTKFKTEGLCVDIPGIPKDMTYRRLISMMGF	5985
MN996529.1	AENVTGLFKDCSKVITGLHPTQAPTHLSVDTKFKTEGLCVDIPGIPKDMTYRRLISMMGF	5985
MT072688.1	AENVTGLFKDCSKVITGLHPTQAPTHLSVDTKFKTEGLCVDIPGIPKDMTYRRLISMMGF	5985
**NC_045512.2**	**AENVTGLFKDCSKVITGLHPTQAPTHLSVDTKFKTEGLCVDIPGIPKDMTYRRLISMMGF**	**5985**
MN994467.1	AENVTGLFKDCSKVITGLHPTQAPTHLSVDTKFKTEGLCVDIPGIPKDMTYRRLISMMGF	5985
MG772933.1	AENVTGLFKDCSKIITGLHPTQAPTHLSVDTKFKTEGLCVDIPGIPKDMTYRRLISMMGF	5981
MG772934.1	AENVTGLFKDCSKIITGLHPTQAPTHLSVDTKFKTEGLCVDIPGIPKDMTYRRLISMMGF	5959
NC_014470.1	SENVTGLFKDCSKLITGLHPTQAPTYLSVDTKFKTEGLCVDIPGIPKDMTYRRLISMMGF	5953
GQ153542.1	AENVTGLFKDCSKIITGLHPTQAPTHLSVDTKFKTEGLCVDIPGIPKDMTYRRLISMMGF	5960
DQ022305	AENVTGLFKDCSKIITGLHPTQAPTHLSVDTKFKTEGLCVDIPGIPKDMTYRRLISMMGF	5956
KF569996.1	AENVTGLFKDCSKIITGLHPTQAPTHLSVDTKFKTEGLCVDIPGIPKDMTYRRLISMMGF	5899
KP886809.1	AENVTGLFKDCSKIITGLHPTQAPTHLSVDTKFKTEGLCVDIPGIPKDMTYRRLISMMGF	5962
AY278488.2	AENVTGLFKDCSKIITGLHPTQAPTHLSVDTKFKTEGLCVDIPGIPKDMTYRRLISMMGF	5962
AY485277.1	AENVTGLFKDCSKIITGLHPTQAPTHLSVDIKFKTEGLCVDIPGIPKDMTYRRLISMMGF	5962
AP006560.1	AENVTGLFKDCSKIITGLHPTQAPTHLSVDIKFKTEGLCVDIPGIPKDMTYRRLISMMGF	5962
AP006557.1	AENVTGLFKDCSKIITGLHPTQAPTHLSVDIKFKTEGLCVDIPGIPKDMTYRRLISMMGF	5962
AY274119	AENVTGLFKDCSKIITGLHPTQAPTHLSVDIKFKTEGLCVDIPGIPKDMTYRRLISMMGF	5962
AY572038.1	AENVTGLFKDCSKIITGLHPTQAPTHLSVDIKFKTEGLCVDIPGIPKDMTYRRLISMMGF	5962
AY572034.1	AENVTGLFKDCSKIITGLHPTQAPTHLSVDIKFKTEGLCVDIPGIPKDMTYRRLISMMGF	5962
FJ588686.1	AENVTGLFKDCSKIITGLHPTQAPTHLSVDTKFKTEGLCVDIPGIPKDMTYRRLISMMGF	5769
KY417145.1	AENVTGLFKDCSKIITGLHPTQAPTHLSVDTKFKTEGLCVDIPGIPKDMTYRRLISMMGF	5962
KY417144.1	AENVTGLFKDCSKIITGLHPTQAPTHLSVDTKFKTEGLCVDIPGIPKDMTYRRLISMMGF	5962
KY417147.1	AENVTGLFKDCSKIINGLHPTQAPTHLSVDTKFKTEGLCVDIPGIPKDMTYRRLISMMGF	5962
KY417148.1	AENVTGLFKDCSKIITGLHPTQAPTHLSVDTKFKTEGLCVDIPGIPKDMTYRRLISMMGF	5962
KY417143.1	AENVTGLFKDCSKIITGLHPTQAPTHLSVDTKFKTEGLCVDIPGIPKDMTYRRLISMMGF	5962
KT444582.1	AENVTGLFKDCSKIITGLHPTQAPTHLSVDTKFKTEGLCVDIPGIPKDMTYRRLISMMGF	5962
KC881005.1	AENVTGLFKDCSKIITGLHPTQAPTHLSVDTKFKTEGLCVDIPGIPKDMTYRRLISMMGF	5962
KC881006.1	AENVTGLFKDCSKIITGLHPTQAPTHLSVDTKFKTEGLCVDIPGIPKDMTYRRLISMMGF	5962
KF367457.1	AENVTGLFKDCSKIITGLHPTQAPTHLSVDTKFKTEGLCVDIPGIPKDMTYRRLISMMGF	5962
KY417152.1	AENVTGLFKDCSKIITGLHPTQAPTHLSVDTKFKTEGLCVDIPGIPKDMTYRRLISMMGF	5962
KY417146.1	AENVTGLFKDCSKIITGLHPTQAPTHLSVDTKFKTEGLCVDIPGIPKDMTYRRLISMMGF	5962
KY417151.1	AENVTGLFKDCSKIITGLHPTQAPTHLSVDTKFKTEGLCVDIPGIPKDMTYRRLISMMGF	5962
KY417142.1	AENVTGLFKDCSKIITGLHPTQAPTHLSVDTKFKTEGLCVDIPGIPKDMTYRRLISMMGF	5962
	:************:*.*********:**** *****************************	

MW532698.1	KMNYQVNGYPNMFITREEAIKHVRAWVGFDVEGCHATREAVGTNLPLQLGFSTGVNLVAV	6037
MT040336.1	KMNYQVNGYPNMFITREEAIKHVRAWVGFDVEGCHATREAVGTNLPLQLGFSTGVNLVAV	6037
MT040335.1	KMNYQVNGYPNMFITREEAIKHVRAWVGFDVEGCHATREAVGTNLPLQLGFSTGVNLVAV	6037
MT040334.1	KMNYQVNGYPNMFITREEAIKHVRAWVGFDVEGCHATREAVGTNLPLQLGFSTGVNLVAV	6037
MT040333.1	KMNYQVNGYPNMFITREEAIKHVRAWVGFDVEGCHATREAVGTNLPLQLGFSTGVNLVAV	6037
MN996532.2	KMNYQVNGYPNMFITREEAVRHVRAWIGFDVEGCHATREAIGTNLPLQLGFSTGVNLVAV	6044
MN988713.1	KMNYQVNGYPNMFITREEAIRHVRAWIGFDVEGCHATREAVGTNLPLQLGFSTGVNLVAV	6045
MT093571.1	KMNYQVNGYPNMFITREEAIRHVRAWIGFDVEGCHATREAVGTNLPLQLGFSTGVNLVAV	6045
MN996529.1	KMNYQVNGYPNMFITREEAIRHVRAWIGFDVEGCHATREAVGTNLPLQLGFSTGVNLVAV	6045
MT072688.1	KMNYQVNGYPNMFITREEAIRHVRAWIGFDVEGCHATREAVGTNLPLQLGFSTGVNLVAV	6045
**NC_045512.2**	**KMNYQVNGYPNMFITREEAIRHVRAWIGFDVEGCHATREAVGTNLPLQLGFSTGVNLVAV**	**6045**
MN994467.1	KMNYQVNGYPNMFITREEAIRHVRAWIGFDVEGCHATREAVGTNLPLQLGFSTGVNLVAV	6045
MG772933.1	KMNYQVNGYPNMFITREEAIRHVRAWIGFDVEGCHATRDAVGTNLPLQLGFSTGVNLVAV	6041
MG772934.1	KMNYQVNGYPNMFITREEAIRHVRAWIGFDVEGCHATRDAVGTNLPLQLGFSTGVNLVTV	6019
NC_014470.1	KMNYQVNGYPNMFITRDEAIKHVRAWIGFDVEGCHATRDAVGTNLPLQLGFSTGVNLVAV	6013
GQ153542.1	KMNYQVNGYPNMFITREEAIRHVRAWIGFDVEGCHATRDAVGTNLPLQLGFSTGVNLVAV	6020
DQ022305	KMNYQVNGYPNMFITREEAIRHVRAWIGFDVEGCHATRDAVGTNLPLQLGFSTGVNLVAV	6016
KF569996.1	KMNYQVNGYPNMFITREEAIRHVRAWIGFDVEGCHATRDAVGTNLPLQLGFSTGVNLVAV	5959
KP886809.1	KMNYQVNGYPNMFITREEAIRHVRAWIGFDVEGCHATRDAVGTNLPLQLGFSTGVNLVAV	6022
AY278488.2	KMNYQVNGYPNMFITREEAIRHVRAWIGFDVEGCHATRDAVGTNLPLQLGFSTGVNLVAV	6022
AY485277.1	KMNYQVNGYPNMFITREEAIRHVRAWIGFDVEGCHATRDAVGTNLPLQLGFSTGVNLVAV	6022
AP006560.1	KMNYQVNGYPNMFITREEAIRHVRAWIGFDVEGCHATRDAVGTNLPLQLGFSTGVNLVAV	6022
AP006557.1	KMNYQVNGYPNMFITREEAIRHVRAWIGFDVEGCHATRDAVGTNLPLQLGFSTGVNLVAV	6022
AY274119	KMNYQVNGYPNMFITREEAIRHVRAWIGFDVEGCHATRDAVGTNLPLQLGFSTGVNLVAV	6022
AY572038.1	KMNYQVNGYPHMFITREEAIRHVRAWIGFDVEGCHATRDAVGTNLPLQLGFSTGVNLVAV	6022
AY572034.1	KMNYQVNGYPHMFITREEAIRHVRAWIGFDVEGCHATRDAVGTNLPLQLGFSTGVNLVAV	6022
FJ588686.1	KMNYQVNGYPNMFITREEAIRHVRAWIGFDVEGCHATRDAVGTNLPLQLGFSTGVNLVAV	5829
KY417145.1	KMNYQVNGYPNMFITREEAIRHVRAWIGFDVEGCHATRDAVGTNLPLQLGFSTGVNLVAV	6022
KY417144.1	KMNYQVNGYPNMFITREEAIRHVRAWIGFDVEGCHATRDAVGTNLPLQLGFSTGVNLVAV	6022
KY417147.1	KMNYQVNGYPNMFITREEAIRHVRAWIGFDVEGCHATRDAVGTNLPLQLGFSTGVNLVAV	6022
KY417148.1	KMNYQVNGYPNMFITREEAIRHVRAWIGFDVEGCHATRDAVGTNLPLQLGFSTGVNLVAV	6022
KY417143.1	KMNYQVNGYPNMFITREEAIRHVRAWIGFDVEGCHATRDAVGTNLPLQLGFSTGVNLVAV	6022
KT444582.1	KMNYQVNGYPNMFITREEAIRHVRAWIGFDVEGCHATRDAVGTNLPLQLGFSTGVNLVAV	6022
KC881005.1	KMNYQVNGYPNMFITREEAIRHVRAWIGFDVEGCHATRDAVGTNLPLQLGFSTGVNLVAV	6022
KC881006.1	KMNYQVNGYPNMFITREEAIRHVRAWIGFDVEGCHATRDAVGTNLPLQLGFSTGVNLVAV	6022
KF367457.1	KMNYQVNGYPNMFITREEAIRHVRAWIGFDVEGCHATRDAVGTNLPLQLGFSTGVNLVAV	6022
KY417152.1	KMNYQVNGYPNMFITREEAIRHVRAWIGFDVEGCHATRDAVGTNLPLQLGFSTGVNLVAV	6022
KY417146.1	KMNYQVNGYPNMFITREEAIRHVRAWIGFDVEGCHATRDAVGTNLPLQLGFSTGVNLVAV	6022
KY417151.1	KMNYQVNGYPNMFITREEAIRHVRAWIGFDVEGCHATRDAVGTNLPLQLGFSTGVNLVAV	6022
KY417142.1	KMNYQVNGYPNMFITREEAIRHVRAWIGFDVEGCHATRDAVGTNLPLQLGFSTGVNLVAV	6022
	**********:*****:**::*****:***********:*:*****************:*	

MW532698.1	PTGYVDTPNATEFSRVSAKPPPGDQFKHLIPLMYKGLPWNIVRIKIVQMLSDTLKNLSDR	6097
MT040336.1	PTGYVDTSNATEFSRVSAKPPPGDQFKHLIPLMYKGLPWNIVRIKIVQMLSDTLKNLSDR	6097
MT040335.1	PTGYVDTSNATEFSRVSAKPPPGDQFKHLIPLMYKGLPWNIVRIKIVQMLSDTLKNLSDR	6097
MT040334.1	PTGYVDTSNATEFSRVSAKPPPGDQFKHLIPLMYKGLPWNIVRIKIVQMLSDTLKNLSDR	6097
MT040333.1	PTGYVDTSNATEFSRVSAKPPPGDQFKHLIPLMYKGLPWNIVRIKIVQMLSDTLKNLSDR	6097
MN996532.2	PTGYVDTPNNTDFSRVSAKPPPGDQFKHLIPLMYKGLPWNVVRIKIVQMLSDTLKNLSDR	6104
MN988713.1	PTGYVDTPNNTDFSRVSAKPPPGDQFKHLIPLMYKGLPWNVVRIKIVQMLSDTLKNLSDR	6105
MT093571.1	PTGYVDTPNNTDFSRVSAKPPPGDQFKHLIPLMYKGLPWNVVRIKIVQMLSDTLKNLSDR	6105
MN996529.1	PTGYVDTPNNTDFSRVSAKPPPGDQFKHLIPLMYKGLPWNVVRIKIVQMLSDTLKNLSDR	6105
MT072688.1	PTGYVDTPNNTDFSRVSAKPPPGDQFKHLIPLMYKGLPWNVVRIKIVQMLSDTLKNLSDR	6105
**NC_045512.2**	**PTGYVDTPNNTDFSRVSAKPPPGDQFKHLIPLMYKGLPWNVVRIKIVQMLSDTLKNLSDR**	**6105**
MN994467.1	PTGYVDTPNNTDFSRVSAKPPPGDQFKHLIPLMYKGLPWNVVRIKIVQMLSDTLKNLSDR	6105
MG772933.1	PTGYVDTEHSTEFTRVNAKPPPGDQFKHLIPLMYKGLPWNVVRIKIVQMLSDTLKGLSDR	6101
MG772934.1	PTGYVDTENSTEFTRVNAKPPPGDQFKHLIPLMYKGLPWNVVRIKIVQMLSDTLKGLSDR	6079
NC_014470.1	PTGYVDTSAATEFSRVNAKPPPGDQFKHLIPLMYKGLPWNIVRVKIVQMLSDTLKDLSDR	6073
GQ153542.1	PTGYVDTENSTEFTRVNAKPPPGDQFKHLIPLMYKGLPWNVVRIKIVQMLSDTLRGLSDR	6080
DQ022305	PTGYVDTENSTEFTRVNAKPPPGDQFKHLIPLMYKGLPWNVVRIKIVQMLSDTLKGLSDR	6076
KF569996.1	PTGYVDTENNTEVTRVNAKPPPGDQFKHLIPLMYKGLPWNIVRIKIVQMLSDTLKGLSDR	6019
KP886809.1	PTGYVDTENNTEFTRVNAKPPPGDQFKHLIPLMYKGLPWNVVRIKIVQMLSDTLKGLSDR	6082
AY278488.2	PTGYVDTENNTEFTRVNAKPPPGDQFKHLIPLMYKGLPWNVVRIKIVQMLSDTLKGLSDR	6082
AY485277.1	PTGYVDTENNTEFTRVNAKPPPGDQFKHLIPLMYKGLPWNVVRIKIVQMLSDTLKGLSDR	6082
AP006560.1	PTGYVDTENNTEFTRVNAKPPPGDQFKHLIPLMYKGLPWNVVRIKIVQMLSDTLKGLSDR	6082
AP006557.1	PTGYVDTENNTEFTRVNAKPPPGDQFKHLIPLMYKGLPWNVVRIKIVQMLSDTLKGLSDR	6082
AY274119	PTGYVDTENNTEFTRVNAKPPPGDQFKHLIPLMYKGLPWNVVRIKIVQMLSDTLKGLSDR	6082
AY572038.1	PTGYVDTENNTEFTRVNAKPPPGDQFKHLIPLMYKGLPWNVVRIKIVQMLSDTLKGLSDR	6082
AY572034.1	PTGYVDTENNTEFTRVNAKPPPGDQFKHLIPLMYKGLPWNVVRIKIVQMLSDTLKGLSDR	6082
FJ588686.1	PTGYVDTENNTECTRVNAKPPPGDQFKHLIPLMYKGLPWNVVRIKIVQMLSDTLKGLSDR	5889
KY417145.1	PTGYVDTENNTEFTRVNAKPPPGDQFKHLIPLMYKGLPWNVVRIKIVQMLSDTLKGLSDR	6082
KY417144.1	PTGYVDTENNTEFTRVNARPPPGDQFKHLIPLMYKGLPWNVVRIKIVQMLSDTLKGLSDR	6082
KY417147.1	PTGYVDTENNTEFTRVNAKPPPGDQFKHLIPLMYKGLPWNVVRIKIVQMLSDTLKGLSDR	6082
KY417148.1	PTGYVDTENNTEFTRVNAKPPPGDQFKHLIPLMYKGLPWNVVRIKIVQMLSDTLKGLSDR	6082
KY417143.1	PTGYVDTENNTEFTRVNAKPPPGDQFKHLIPLMYKGLPWNVVRIKIVQMLSDTLKGLSDR	6082
KT444582.1	PTGYVDTENNTEFTRVNAKPPPGDQFKHLIPLMYKGLPWNIVRIKIVQMLNDTLKGLSDR	6082
KC881005.1	PTGYVDTENNTEFTRVNARPPPGDQFKHLIPLMYKGLPWNVVRIKIVQMLSDTLKGLSDR	6082
KC881006.1	PTGYVDTENNTEFTRVNARPPPGDQFKHLIPLMYKGLPWNVVRIKIVQMLSDTLKGLSDR	6082
KF367457.1	PTGYVDTENNTEFTRVNARPPPGDQFKHLIPLMYKGLPWNVVRIKIVQMLSDTLKGLSDR	6082
KY417152.1	PTGYVDTENNTEFTRVNAKPPPGDQFKHLIPLMYKGLPWNVVRIKIVQMLSDTLKGLSDR	6082
KY417146.1	PTGYVDTENNTEFTRVNAKPPPGDQFKHLIPLMYKGLPWNVVRIKIVQMLSDTLKGLSDR	6082
KY417151.1	PTGYVDTENNTEFTRVNAKPPPGDQFKHLIPLMYKGLPWNVVRIKIVQMLSDTLKGLSDR	6082
KY417142.1	PTGYVDTENNTEFTRVNAKPPPGDQFKHLIPLMYKGLPWNVVRIKIVQMLSDTLKGLSDR	6082
	******* *: :**.*:*********************:**:******.***:.****	

```
MW532698.1      VVFVLWAHGFELTSMKYFVKIGPERTCCLCDKRATCFCTASDTYACWHHSVGFDYVYNPF   6157
MT040336.1      VVFVLWAHGFELTSMKYFVKIGPERTCCLCDKRATCFCTASDTYACWHHSVGFDYVYNPF   6157
MT040335.1      VVFVLWAHGFELTSMKYFVKIGPERTCCLCDKRATCFCTASDTYACWHHSVGFDYVYNPF   6157
MT040334.1      VVFVLWAHGFELTSMKYFVKIGPERTCCLCDKRATCFCTASDTYACWHHSVGFDYVYNPF   6157
MT040333.1      VVFVLWAHGFELTSMKYFVKIGPERTCCLCDKRATCFCTASDTYACWHHSVGFDYVYNPF   6157
MN996532.2      VVFVLWAHGFELTSMKYFVKIGPERTCCLCDKRATCFSTASDTYACWHHSIGFDYVYNPF   6164
MN988713.1      VVFVLWAHGFELTSMKYFVKIGPERTCCLCDRRATCFSTASDTYACWHHSIGFDYVYNPF   6165
MT093571.1      VVFVLWAHGFELTSMKYFVKIGPERTCCLCDRRATCFSTASDTYACWHHSIGFDYVYNPF   6165
MN996529.1      VVFVLWAHGFELTSMKYFVKIGPERTCCLCDRRATCFSTASDTYACWHHSIGFDYVYNPF   6165
MT072688.1      VVFVLWAHGFELTSMKYFVKIGPERTCCLCDRRATCFSTASDTYACWHHSIGFDYVYNPF   6165
NC_045512.2     VVFVLWAHGFELTSMKYFVKIGPERTCCLCDRRATCFSTASDTYACWHHSIGFDYVYNPF   6165
MN994467.1      VVFVLWAHGFELTSMKYFVKIGPERTCCLCDRRATCFSTASDTYACWHHSIGFDYVYNPF   6165
MG772933.1      VVFVLWAHGFELTSMKYFVKIGPERTCCLCDKRATCFSTSSDTYACWNHSVGFDYVYNPF   6161
MG772934.1      VVFVLWAHGFELTSMKYFVKIGPERTCCLCDKRATCFSTSSDTYACWNHSVGFDYVYNPF   6139
NC_014470.1     VVFVLWAHGFELTSMKYFVKIGPERTCCLCDKRATCFSTSSDTYACWHHSVGFDYVYNPF   6133
GQ153542.1      VVFVLWAHGFELTSMKYFVKIGPERTCCLCDKRATCFSTSSDTYACWNHSVSFDYVYNPF   6140
DQ022305        VVFVLWAHGFELTSMKYFVKIGPERTCCLCDKRATCFSTSSDTYACWNHSVGFDYVYNPF   6136
KF569996.1      VVFVLWAHGFELTSMKYFVKIGPERTCCLCDKRATCFSTSSDTYACWNHSVGFDYVYNPF   6079
KP886809.1      VVFVLWAHGFELTSMKYFVKIGPERTCCLCDKRATCFSTSSDTYACWNHSVGFDYVYNPF   6142
AY278488.2      VVFVLWAHGFELTSMKYFVKIGPERTCCLCDKRATCFSTSSDTYACWNHSVGFDYVYNPF   6142
AY485277.1      VVFVLWAHGFELTSMKYFVKIGPERTCCLCDKRATCFSTSSDTYACWNHSVGFDYVYNPF   6142
AP006560.1      VVFVLWAHGFELTSMKYFVKIGPERTCCLCDKRATCFSTSSDTYACWNHSVGFDYVYNPF   6142
AP006557.1      VVFVLWAHGFELTSMKYFVKIGPERTCCLCDKRATCFSTSSDTYACWNHSVGFDYVYNPF   6142
AY274119        VVFVLWAHGFELTSMKYFVKIGPERTCCLCDKRATCFSTSSDTYACWNHSVGFDYVYNPF   6142
AY572038.1      VVFVLWAHGFELTSMKYFVKIGPERTCCLCDKRATCFSTSSDTYACWNHSVGFDYVYNPF   6142
AY572034.1      VVFVLWAHGFELTSMKYFVKIGPERTCCLCDKRATCFSTSSDTYACWNHSVGFDYVYNPF   6142
FJ588686.1      VVFVLWAHGFELTSMKYFVKIGPERTCCLCDRRATCFSTSSDTYACWNHSVGFDYVYNPF   5949
KY417145.1      VVFVLWAHGFELTSMKYFVKIGPERTCCLCDKRATCFSTSSDTYACWNHSVGFDYVYNPF   6142
KY417144.1      VVFVLWAHGFELTSMKYFVKIGPERTCCLCDKRATCFSTSSDTYACWNHSVGFDYVYNPF   6142
KY417147.1      VVFVLWAHGFELTSMKYFVKIGPERTCCLCDKRATCFSTSSDTYACWNHSVGFDYVYNPF   6142
KY417148.1      VVFVLWAHGFELTSMKYFVKIGPERTCCLCDKRATCFSTSSDTYACWNHSVGFDYVYNPF   6142
KY417143.1      VVFVLWAHGFELTSMKYFVKIGPERTCCLCDKRATCFSTSSDTYACWNHSVGFDYVYNPF   6142
KT444582.1      VVFVLWAHGFELTSMKYFVKIGPERTCCLCDKRATCFSTSSDTYACWNHSVGFDYVYNPF   6142
KC881005.1      VVFVLWAHGFELTSMKYFVKIGPERTCCLCDKRATCFSTSSDTYACWNHSVGFDYVYNPF   6142
KC881006.1      VVFVLWAHGFELTSMKYFVKIGPERTCCLCDKRATCFSTSSDTYACWNHSVGFDYVYNPF   6142
KF367457.1      VVFVLWAHGFELTSMKYFVKIGPERTCCLCDKRATCFSTSSDTYACWNHSVGFDYVYNPF   6142
KY417152.1      VVFVLWAHGFELTSMKYFVKIGPERTCCLCDKRATCFSTSSDTYACWNHSVGFDYVYNPF   6142
KY417146.1      VVFVLWAHGFELTSMKYFVKIGPERTCCLCDKRATCFSTSSDTYACWNHSVGFDYVYNPF   6142
KY417151.1      VVFVLWAHGFELTSMKYFVKIGPERTCCLCDKRATCFSTSSDTYACWNHSVGFDYVYNPF   6142
KY417142.1      VVFVLWAHGFELTSMKYFVKIGPERTCCLCDKRATCFSTSSDTYACWNHSVGFDYVYNPF   6142
                *******************************:*****.*:*******:**:.********
```

```
MW532698.1      MIDVQQWGFTGNLQSNHDQYCQVHGNAHVASCDAIMTRCLAVHECFVKRVDWTIEYPIIG    6217
MT040336.1      MIDVQQWGFTGNLQSNHDQYCQVHGNAHVASCDAIMTRCLAVHECFVKRVDWTIEYPIIG    6217
MT040335.1      MIDVQQWGFTGNLQSNHDQYCQVHGNAHVASCDAIMTRCLAVHECFVKRVDWTIEYPIIG    6217
MT040334.1      MIDVQQWGFTGNLQSNHDQYCQVHGNAHVASCDAIMTRCLAVHECFVKRVDWTIEYPIIG    6217
MT040333.1      MIDVQQWGFTGNLQSNHDQYCQVHGNAHVASCDAIMTRCLAVHECFVKRVDWTIEYPIIG    6217
MN996532.2      MIDVQQWGFTGNLQSNHDLYCQVHGNAHVASCDAIMTRCLAVHECFVKRVDWTIEYPIIG    6224
MN988713.1      MIDVQQWGFTGNLQSNHDLYCQVHGNAHVASCDAIMTRCLAVHECFVKRVDWTIEYPIIG    6225
MT093571.1      MIDVQQWGFTGNLQSNHDLYCQVHGNAHVASCDAIMTRCLAVHECFVKRVDWTIEYPIIG    6225
MN996529.1      MIDVQQWGFTGNLQSNHDLYCQVHGNAHVASCDAIMTRCLAVHECFVKRVDWTIEYPIIG    6225
MT072688.1      MIDVQQWGFTGNLQSNHDLYCQVHGNAHVASCDAIMTRCLAVHECFVKRVDWTIEYPIIG    6225
NC_045512.2     MIDVQQWGFTGNLQSNHDLYCQVHGNAHVASCDAIMTRCLAVHECFVKRVDWTIEYPIIG    6225
MN994467.1      MIDVQQWGFTGNLQSNHDLYCQVHGNAHVASCDAIMTRCLAVHECFVKRVDWTIEYPIIG    6225
MG772933.1      MIDVQQWGFTGNLQSNHDQHCQVHGNAHVASCDAIMTRCLAVHECFVKRVDWSVEYPIIG    6221
MG772934.1      MIDVQQWGFTGNLQSNHDQHCQVHGNAHVASCDAIMTRCLAVHECFVKRVDWSVEYPIIG    6199
NC_014470.1     MIDVQQWGFTGNLQSNHDQHCQVHGNAHVASCDAIMTRCLAIHECFVKRVDWSVEYPIIG    6193
GQ153542.1      MIDVQQWGFTGNLQSNHDQHCQVHGNAHVASCDAIMTRCLAVHECFVKRVDWSIEYPIIG    6200
DQ022305        MIDVQQWGFTGNLQSNHDQHCQVHGNAHVASCDAIMTRCLAVHECFVKRVDWSVEYPIIG    6196
KF569996.1      MIDVQQWGFTGNLQSNHDQHCQVHGNAHVASCDAIMTRCLAVHECFVKRVDWSVEYPIIG    6139
KP886809.1      MIDVQQWGFTGNLQSNHDQHCQVHGNAHVASCDAIMTRCLAVHECFVKRVDWSVEYPIIG    6202
AY278488.2      MIDVQQWGFTGNLQSNHDQHCQVHGNAHVASCDAIMTRCLAVHECFVKRVDWSVEYPIIG    6202
AY485277.1      MIDVQQWGFTGNLQSNHDQHCQVHGNAHVASCDAIMTRCLAVHECFVKRVDWSVEYPIIG    6202
AP006560.1      MIDVQQWGFTGNLQSNHDQHCQVHGNAHVASCDAIMTRCLAVHECFVKRVDWSVEYPIIG    6202
AP006557.1      MIDVQQWGFTGNLQSNHDQHCQVHGNAHVASCDAIMTRCLAVHECFVKRVDWSVEYPIIG    6202
AY274119        MIDVQQWGFTGNLQSNHDQHCQVHGNAHVASCDAIMTRCLAVHECFVKRVDWSVEYPIIG    6202
AY572038.1      MIDVQQWGFTGNLQSNHDQHCQVHGNAHVASCDAIMTRCLAVHECFVKRVDWSVEYPIIG    6202
AY572034.1      MIDVQQWGFTGNLQSNHDQHCQVHGNAHVASCDAIMTRCLAVHECFVKRVDWSVEYPIIG    6202
FJ588686.1      MIDVQQWGFTGNLQSNHDQHCQVHGNAHVASCDAIMTRCLAVHECFVKRVDWSVEYPIIG    6009
KY417145.1      MIDVQQWGFTGNLQSNHDQHCQVHGNAHVASCDAIMTRCLAIHECFVKRVDWSVEYPIIG    6202
KY417144.1      TIDVQQWGFTGNLQSNHDQHCQVHGNAHVASCDAIMTRCLAVHECFVKRVDWSVEYPIIG    6202
KY417147.1      MIDVQQWGFTGNLQSNHDQHCQVHGNAHVASCDAIMTRCLAVHECFVKRVDWSVEYPIIG    6202
KY417148.1      MIDVQQWGFTGNLQSNHDQHCQVHGNAHVASCDAIMTRCLAVHECFVKRVDWSVEYPIIG    6202
KY417143.1      MIDVQQWGFTGNLQSNHDQYCQVHGNAHVASCDAIMTRCLAVHECFVKRVDWSVEYPIIG    6202
KT444582.1      MIDVQQWGFTGNLQSNHDQHCQVHGNAHVASCDAIMTRCLAVHECFVKRVDWSVEYPIIG    6202
KC881005.1      MIDVQQWGFTGNLQSNHDQHCQVHGNAHVASCDAIMTRCLAVHECFVKRVDWSVEYPIIG    6202
KC881006.1      MIDVQQWGFTGNLQSNHDQHCQVHGNAHVASCDAIMTRCLAVHECFVKRVDWSVEYPIIG    6202
KF367457.1      MIDVQQWGFTGNLQSNHDQHCQVHGNAHVASCDAIMTRCLAVHECFVKRVDWSVEYPIIG    6202
KY417152.1      MIDVQQWGFTGNLQSNHDQHCQVHGNAHVASCDAIMTRCLAVHECFVKRVDWSVEYPIIG    6202
KY417146.1      MIDVQQWGFTGNLQSNHDQHCQVHGNAHVASCDAIMTRCLAVHECFVKRVDWSVEYPIIG    6202
KY417151.1      MIDVQQWGFTGNLQSNHDQHCQVHGNAHVASCDAIMTRCLAVHECFVKRVDWSVEYPIIG    6202
KY417142.1      MIDVQQWGFTGNLQSNHDQHCQVHGNAHVASCDAIMTRCLAVHECFVKRVDWSVEYPIIG    6202
                 ***************** :********************:**********::******
```

MW532698.1	DELKINAACRKVQHMVVKAALLADKFPVLHDIGNPKAIKCVPQADTDWKFYDAQPCSDKA	6277
MT040336.1	DELKINAACRKVQHMVVKAALLADKFPVLHDIGNPKAIKCVPQADTDWKFYDAQPCSDKA	6277
MT040335.1	DELKINAACRKVQHMVVKAALLADKFPVLHDIGNPKAIKCVPQADTDWKFYDAQPCSDKA	6277
MT040334.1	DELKINAACRKVQHMVVKAALLADKFPVLHDIGNPKAIKCVPQADTDWKFYDAQPCSDKA	6277
MT040333.1	DELKINAACRKVQHMVVKAALLADKFPVLHDIGNPKAIKCVPQADTDWKFYDAQPCSDKA	6277
MN996532.2	DELKINAACRKVQHMVVKAALLADKFPVLHDIGNPKAIKCVPQADVEWKFYDAQPCSDKA	6284
MN988713.1	DELKINAACRKVQHMVVKAALLADKFPVLHDIGNPKAIKCVPQADVEWKFYDAQPCSDKA	6285
MT093571.1	DELKINAACRKVQHMVVKAALLADKFPVLHDIGNPKAIKCVPQADVEWKFYDAQPCSDKA	6285
MN996529.1	DELKINAACRKVQHMVVKAALLADKFPVLHDIGNPKAIKCVPQADVEWKFYDAQPCSDKA	6285
MT072688.1	DELKINAACRKVQHMVVKAALLADKFPVLHDIGNPKAIKCVPQADVEWKFYDAQPCSDKA	6285
NC_045512.2	DELKINAACRKVQHMVVKAALLADKFPVLHDIGNPKAIKCVPQADVEWKFYDAQPCSDKA	6285
MN994467.1	DELKINAACRKVQHMVVKAALLADKFPVLHDIGNPKAIKCVPQADVEWKFYDAQPCSDKA	6285
MG772933.1	DELKINAACRKVQHMVVKSALLADKFPVLHDIGNPKAIRCVPQSEVDWKFYDAQPCSDKA	6281
MG772934.1	DELKINAACRKVQHMVVKSALLADKFPVLHDIGNPKAIRCVPQAEVDWKFYDAQPCSDKA	6259
NC_014470.1	DELRINVACRKVQHMVVKSALLADKFPVLHDIGNPKAIKCVPQADVEWKFYDVQPCSDKA	6253
GQ153542.1	DELKINAACRKVQHMVVKSALLADKFPVLHDIGNPKAIRCVPQAEVDWKFYDAQPCIDKA	6260
DQ022305	DELKINAACRKVQHMVVKSALLADKFTVLHDIGNPKAIRCVPQAEVDWKFYDAQPCSDKA	6256
KF569996.1	DELKINAACRKVQHMVVKSALLADKFSVLHDIGNPKAIKCVPQAEVDWKFYDAQPCSDKA	6199
KP886809.1	DELKINSACRKVQHMVVKSALLADKFPVLHDIGNPKAIKCVPQAEVEWKFYDAQPCSDKA	6262
AY278488.2	DELRVNSACRKVQHMVVKSALLADKFPVLHDIGNPKAIKCVPQAEVEWKFYDAQPCSDKA	6262
AY485277.1	DELRVNSACRKVQHMVVKSALLADKFPVLHDIGNPKAIKCVPQAEVEWKFYDAQPCSDKA	6262
AP006560.1	DELRVNSACRKVQHMVVKSALLADKFPVLHDIGNPKAIKCVPQAEVEWKFYDAQPCSDKA	6262
AP006557.1	DELRVNSACRKVQHMVVKSALLADKFPVLHDIGNPKAIKCVPQAEVEWKFYDAQPCSDKA	6262
AY274119	DELRVNSACRKVQHMVVKSALLADKFPVLHDIGNPKAIKCVPQAEVEWKFYDAQPCSDKA	6262
AY572038.1	DELRVNSACRKVQHMVVKSALLADKFPVLHDIGNPKAIKCVPQAEVEWKFYDAQPCSDKA	6262
AY572034.1	DELRVNSACRKVQHMVVKSALLADKFPVLHDIGNPKAIKCVPQAEVEWKFYDAQPCSDKA	6262
FJ588686.1	DELKINSACRKVQHMVVKSALLADKFPVLHDIGNPKAIKCVPQAEVEWKFYDAQPCSDKA	6069
KY417145.1	DELKINSACRKVQHMVVKSALLADKFPVLYDIGNPKAIKCVPQAEVEWKFYDAQPCSDKA	6262
KY417144.1	DELKINSACRKVQHMVVKSALLADKFPVLHDIGSPKAIKCVPQAEVEWKFYDAQPCSDKA	6262
KY417147.1	DELKINSACRKVQHMVVKSALLADKFPVLHDIGNPKAIKCVPQAEVEWKFYDAQPCSDKA	6262
KY417148.1	DELKINSACRKVQHMVVKSALLADKFPVLHDIGNPKAIKCVPQAEVEWKFYDAQPCSDKA	6262
KY417143.1	DELKINSACRKVQHMVVKSALLADKFPVLHDIGNPKAIKCVPQAEVEWKFYDAQPCSDKA	6262
KT444582.1	EELKINSACRKVQHMVVKSALLADKFPVLHDIGNPKAIKCVPQAEVEWKFYDAQPCSDKA	6262
KC881005.1	DELKINSACRKVQHMVVKSALLADKFPVLHDIGNPKAIKCVPQAEVEWKFYDAQPCSDKA	6262
KC881006.1	DELKINSACRKVQHMVVKSALLADKFPVLHDIGNPKAIKCVPQAEVEWKFYDAQPCSDKA	6262
KF367457.1	DELKINSACRKVQHMVVKSALLADKFPVLHDIGNPKAIKCVPQAEVEWKFYDAQPCSDKA	6262
KY417152.1	DELKINSACRKVQHMVVKSALLADKFPVLHDIGNPKAIKCVPQAEVEWKFYDAQPCSDKA	6262
KY417146.1	DELKINSACRKVQHMVVKSALLADKFPVLHDIGNPKAIKCVPQAEVEWKFYDAQPCSDKA	6262
KY417151.1	DELKINSACRKVQHMVVKSALLADKFPVLHDIGNPKAIKCVPQAEVEWKFYDAQPCSDKA	6262
KY417142.1	DELKINSACRKVQHMVVKSALLADKFPVLHDIGNPKAIKCVPQAEVEWKFYDAQPCSDKA	6262
	:**::* ***********:******* **:***.****:****::.:*****.*** ***	

```
MW532698.1      YKIEELFYSYATHSDKFKDGVCLFWNCNVDRYPANAIVCRFDTRVLSNLNLPGCDGGSLY    6337
MT040336.1      YKIEELFYSYATHSDKFKDGVCLFWNCNVDRYPANAIVCRFDTRVLSNLNLPGCDGGSLY    6337
MT040335.1      YKIEELFYSYATHSDKFKDGVCLFWNCNVDRYPANAIVCRFDTRVLSNLNLPGCDGGSLY    6337
MT040334.1      YKIEELFYSYATHSDKFKDGVCLFWNCNVDRYPANAIVCRFDTRVLSNLNLPGCDGGSLY    6337
MT040333.1      YKIEELFYSYATHSDKFKDGVCLFWNCNVDRYPANAIVCRFDTRVLSNLNLPGCDGGSLY    6337
MN996532.2      YKIEELFYSYATHSDKFTDGVCLFWNCNVDRYPANSIVCRFDTRVLSNLNLPGCDGGSLY    6344
MN988713.1      YKIEELFYSYATHSDKFTDGVCLFWNCNVDRYPANSIVCRFDTRVLSNLNLPGCDGGSLY    6345
MT093571.1      YKIEELFYSYATHSDKFTDGVCLFWNCNVDRYPANSIVCRFDTRVLSNLNLPGCDGGSLY    6345
MN996529.1      YKIEELFYSYATHSDKFTDGVCLFWNCNVDRYPANSIVCRFDTRVLSNLNLPGCDGGSLY    6345
MT072688.1      YKIEELFYSYATHSDKFTDGVCLFWNCNVDRYPANSIVCRFDTRVLSNLNLPGCDGGSLY    6345
NC_045512.2     YKIEELFYSYATHSDKFTDGVCLFWNCNVDRYPANSIVCRFDTRVLSNLNLPGCDGGSLY    6345
MN994467.1      YKIEELFYSYATHSDKFTDGVCLFWNCNVDRYPANSIVCRFDTRVLSNLNLPGCDGGSLY    6345
MG772933.1      YKIEELFYSYATHHDKFTDGVCLFWNCNVDRYPANAIVCRFDTRVLSNLNLPGCDGGSLY    6341
MG772934.1      YKIEELFYSYATHHDKFTDGVCLFWNCNVDRYPANAIVCRFDTRVLSNLNLPGCDGGSLY    6319
NC_014470.1     YKIEELFYSYATHHDKFTDGVCLFWNCNVDRYPSNAIVCRFDTRVLSNLNLPGCDGGSLY    6313
GQ153542.1      YKIEELFYSYATHHDKFTDGVCLFWNCNVDRYPANAIVCRFDTRVLSNLNLPGCDGGSLY    6320
DQ022305        YKIEELFYSYATHHDKFTDGVCLFWNCNVDRYPANAIVCRFDTRVLSNLNLPGCDGGSLY    6316
KF569996.1      YKIEELFYSYATHHDKFTDGVCLFWNCNVDRYPANAIVCRFDTRVLSNLNLPGCDGGSLY    6259
KP886809.1      YKIEELFYSYATHHDKFTDGVCLFWNCNVDRYPANAIVCRFDTRVLSNLNLPGCDGGSLY    6322
AY278488.2      YKIEELFYSYATHHDKFTDGVCLFWNCNVDRYPANAIVCRFDTRVLSNLNLPGCDGGSLY    6322
AY485277.1      YKIEELFYSYATHHDKFTDGVCLFWNCNVDRYPANAIVCRFDTRVLSNLNLPGCDGGSLY    6322
AP006560.1      YKIEELFYSYATHHDKFTDGVCLFWNCNVDRYPANAIVCRFDTRVLSNLNLPGCDGGSLY    6322
AP006557.1      YKIEELFYSYATHHDKFTDGVCLFWNCNVDRYPANAIVCRFDTRVLSNLNLPGCDGGSLY    6322
AY274119        YKIEELFYSYATHHDKFTDGVCLFWNCNVDRYPANAIVCRFDTRVLSNLNLPGCDGGSLY    6322
AY572038.1      YKIEELFYSYATHHDKFTDGVCLFWNCNVDRYPANAIVCRFDTRVLSNLNLPGCDGGSLY    6322
AY572034.1      YKIEELFYSYATHHDKFTDGVCLFWNCNVDRYPANAIVCRFDTRVLSNLNLPGCDGGSLY    6322
FJ588686.1      YKIEELFYSYATHHDKFTDGVCLFWNCNVDRYPANAIVCRFDTRVLSNLNLPGCDGGSLY    6129
KY417145.1      YKIEELFYSYATHHDKFTDGVCLFWNCNVDRYPANAIVCRFDTRVLSNLNLPGCDGGSLY    6322
KY417144.1      YKIEELFYSYATHHDKFTDGVCLFWNCNVDRYPANAIVCRFDTRVLSNLNLPGCDGGSLY    6322
KY417147.1      YKIEELFYSYATHHDKFTDGVCLFWNCNVDRYPANAIVCRFDTRVLSNLNLPGCDGGSLY    6322
KY417148.1      YKIEELFYSYATHHDKFTDGVCLFWNCNVDRYPANAIVCRFDTRVLSNLNLPGCDGGSLY    6322
KY417143.1      YKIEELFYSYATHHDKFTDGVCLFWNCNVDRYPANAIVCRFDTRVLSNLNLPGCDGGSLY    6322
KT444582.1      YKIEELFYSYATHHDKFTDGVCLFWNCNVDRYPANAFVCKFDTRVLSNLNLPGCDGGSLY    6322
KC881005.1      YKIEELFYSYATHHDKFTDGVCLFWNCNVDRYPANAIVCRFDTRVLSNLNLPGCDGGSLY    6322
KC881006.1      YKIEELFYSYATHHDKFTDGVCLFWNCNVDRYPANAIVCRFDTRVLSNLNLPGCDGGSLY    6322
KF367457.1      YKIEELFYSYATHHDKFTDGVCLFWNCNVDRYPANAIVCRFDTRVLSNLNLPGCDGGSLY    6322
KY417152.1      YKIEELFYSYATHHDKFTDGVCLFWNCNVDRYPANAIVCRFDTRVLSNLNLPGCDGGSLY    6322
KY417146.1      YKIEELFYSYATHHDKFTDGVCLFWNCNVDRYPANAIVCRFDTRVLSNLNLPGCDGGSLY    6322
KY417151.1      YKIEELFYSYATHHDKFTDGVCLFWNCNVDRYPANAIVCRFDTRVLSNLNLPGCDGGSLY    6322
KY417142.1      YKIEELFYSYATHHDKFTDGVCLFWNCNVDRYPANAIVCRFDTRVLSNLNLPGCDGGSLY    6322
                ************* ***.***************:*::**:********************
```

```
MW532698.1      VNKHAFHTPAFDKSAFVNLKQLPFFYYSDSPCESHGKQVVSDIDYVPLKSATCITRCNLG    6397
MT040336.1      VNKHAFHTPAFDKSAFVNLKQLPFFYYSDSPCESHGKQVVSDIDYVPLKSATCITRCNLG    6397
MT040335.1      VNKHAFHTPAFDKSAFVNLKQLPFFYYSDSPCESHGKQVVSDIDYVPLKSATCITRCNLG    6397
MT040334.1      VNKHAFHTPAFDKSAFVNLKQLPFFYYSDSPCESHGKQVVSDIDYVPLKSATCITRCNLG    6397
MT040333.1      VNKHAFHTPAFDKSAFVNLKQLPFFYYSDSPCESHGKQVVSDIDYVPLKSATCITRCNLG    6397
MN996532.2      VNKHAFHTPAFDKSAFVNLKQLPFFYYSDSPCESHGKQVVSDIDYVPLKSATCITRCNLG    6404
MN988713.1      VNKHAFHTPAFDKSAFVNLKQLPFFYYSDSPCESHGKQVVSDIDYVPLKSATCITRCNLG    6405
MT093571.1      VNKHAFHTPAFDKSAFVNLKQLPFFYYSDSPCESHGKQVVSDIDYVPLKSATCITRCNLG    6405
MN996529.1      VNKHAFHTPAFDKSAFVNLKQLPFFYYSDSPCESHGKQVVSDIDYVPLKSATCITRCNLG    6405
MT072688.1      VNKHAFHTPAFDKSAFVNLKQLPFFYYSDSPCESHGKQVVSDIDYVPLKSATCITRCNLG    6405
NC_045512.2     VNKHAFHTPAFDKSAFVNLKQLPFFYYSDSPCESHGKQVVSDIDYVPLKSATCITRCNLG    6405
MN994467.1      VNKHAFHTPAFDKSAFVNLKQLPFFYYSDSPCESHGKQVVSDIDYVPLKSATCITRCNLG    6405
MG772933.1      VNKHAFHTPAFDKSAFTHLKQLPFFYYSDSPCESHGKQVVSDIDYVPLKSATCITRCNLG    6401
MG772934.1      VNKHAFHTPAFDKSAFTHLKQLPFFYYSDSPCESHGKQVVSDIDYVPLKSATCITRCNLG    6379
NC_014470.1     VNKHAFHTPAFDKGAFANLKQLPFFYYSDSPCESHGKQVVSDIDYVPLKSATCITRCNLG    6373
GQ153542.1      VNKHAFHTPAFDKSAFTHLKQLPFFYYSDSPCESHGKQVVSDIDYVPLKSATCITRCNLG    6380
DQ022305        VNKHAFHTPAFDKSAFTHLKQLPFFYYSDSPCESHGKQVVSDIDYVPLKSATCITRCNLG    6376
KF569996.1      VNKHAFHTPAFDKSAFTNLKQLPFFYYSDSPCESHGKQVVSDIDYVPLKSATCITRCNLG    6319
KP886809.1      VNKHAFHTPAFDKSAFTNLKQLPFFYYSDSPCESHGKQVVSDIDYVPLKSATCITRCNLG    6382
AY278488.2      VNKHAFHTPAFDKSAFTNLKQLPFFYYSDSPCESHGKQVVSDIDYVPLKSATCITRCNLG    6382
AY485277.1      VNKHAFHTPAFDKSAFTNLKQLPFFYYSDSPCESHGKQVVSDIDYVPLKSATCITRCNLG    6382
AP006560.1      VNKHAFHTPAFDKSAFTNLKQLPFFYYSDSPCESHGKQVVSDIDYVPLKSATCITRCNLG    6382
AP006557.1      VNKHAFHTPAFDKSAFTNLKQLPFFYYSDSPCESHGKQVVSDIDYVPLKSATCITRCNLG    6382
AY274119        VNKHAFHTPAFDKSAFTNLKQLPFFYYSDSPCESHGKQVVSDIDYVPLKSATCITRCNLG    6382
AY572038.1      VNKHAFHTPAFDKSAFTNLKQLPFFYYSDSPCESHGKQVVSDVDYVPLKSATCITRCNLG    6382
AY572034.1      VNKHAFHTPAFDKSAFTNLKQLPFFYYSDSPCESHGKQVVSDIDYVPLKSATCITRCNLG    6382
FJ588686.1      VNKHAFHTPAFDKSAFTNLKQLPFFYYSDSPCESHGKQVVSDIDYVPLKSATCITRCNLG    6189
KY417145.1      VNKHAFHTPAFDKSAFTNLKQLPFFYYSDSPCESHGKQVVSDIDYVPLKSVTCITRCNLG    6382
KY417144.1      VNKHAFHTPAFDKSAFTNLKQLPFFYYSDSPCESHGKQVVSDIDYVPLKSATCITRCNLG    6382
KY417147.1      VNKHAFHTPAFDKSAFTNLKQLPFFYYSDSPCESHGKQVVSDIDYVPLKSATCITRCNLG    6382
KY417148.1      VNKHAFHTPAFDKSAFTNLKQLPFFYYSDSPCESHGKQVVSDIDYVPLKSATCITRCNLG    6382
KY417143.1      VNKHAFHTPAFDKSAFTNLKQLPFFYYSDSPCESHGKQVVSDIDYVPLKSATCITRCNLG    6382
KT444582.1      VNKHAFHTPAFDKSAFTNLKQLPFFYYSDSPCESHGKQVVSDIDYVPLKSATCITRCNLG    6382
KC881005.1      VNKHAFHTPAFDKSAFTNLKQLPFFYYSDSPCESHGKQVVSDIDYVPLKSATCITRCNLG    6382
KC881006.1      VNKHAFHTPAFDKSAFTNLKQLPFFYYSDSPCESHGKQVVSDIDYVPLKSATCITRCNLG    6382
KF367457.1      VNKHAFHTPAFDKSAFTNLKQLPFFYYSDSPCESHGKQVVSDIDYVPLKSATCITRCNLG    6382
KY417152.1      VNKHAFHTPAFDKSAFTNLKQLPFFYYSDSPCESHGKQVVSDIDYVPLKSATCITRCNLG    6382
KY417146.1      VNKHAFHTPAFDKSAFTNLKQLPFFYYSDSPCESHGKQVVSDIDYVPLKSATCITRCNLG    6382
KY417151.1      VNKHAFHTPAFDKSAFTNLKQLPFFYYSDSPCESHGKQVVSDIDYVPLKSATCITRCNLG    6382
KY417142.1      VNKHAFHTPAFDKSAFTNLKQLPFFYYSDSPCESHGKQVVSDIDYVPLKSATCITRCNLG    6382
                *************.**.:************************:*******.*********
```

```
MW532698.1       GAVCRHHANEYRLYLDAYNMMISAGFSLWIYKQFDTYNLWNTFTRLQSLENVAFNVINKG   6457
MT040336.1       GAVCRHHANEYRLYLDAYNMMISAGFSLWIYKQFDTYNLWNTFTRLQSLENVAFNVINKG   6457
MT040335.1       GAVCRHHANEYRLYLDAYNMMISAGFSLWIYKQFDTYNLWNTFTRLQSLENVAFNVINKG   6457
MT040334.1       GAVCRHHANEYRLYLDAYNMMISAGFSLWIYKQFDTYNLWNTFTRLQSLENVAFNVINKG   6457
MT040333.1       GAVCRHHANEYRLYLDAYNMMISAGFSLWIYKQFDTYNLWNTFTRLQSLENVAFNVINKG   6457
MN996532.2       GAVCRHHANEYRLYLDAYNMMISAGFSLWVYKQFDTYNLWNTFTRLQSLENVAFNVVNKG   6464
MN988713.1       GAVCRHHANEYRLYLDAYNMMISAGFSLWVYKQFDTYNLWNTFTRLQSLENVAFNVVNKG   6465
MT093571.1       GAVCRHHANEYRLYLDAYNMMISAGFSLWVYKQFDTYNLWNTFTRLQSLENVAFNVVNKG   6465
MN996529.1       GAVCRHHANEYRLYLDAYNMMISAGFSLWVYKQFDTYNLWNTFTRLQSLENVAFNVVNKG   6465
MT072688.1       GAVCRHHANEYRLYLDAYNMMISAGFSLWVYKQFDTYNLWNTFTRLQSLENVAFNVVNKG   6465
NC_045512.2      GAVCRHHANEYRLYLDAYNMMISAGFSLWVYKQFDTYNLWNTFTRLQSLENVAFNVVNKG   6465
MN994467.1       GAVCRHHANEYRLYLDAYNMMISAGFSLWVYKQFDTYNLWNTFTRLQSLENVAFNVVNKG   6465
MG772933.1       GAVCRHHANEYRQYLDAYNMMISAGFSLWIYKQFDTYNLWNTFTKLQSLENVAYNVINKG   6461
MG772934.1       GAVCRHHANEYRQYLDAYNMMISAGFSLWIYKQFDTYNLWNTFTKLQSLENVAYNVINKG   6439
NC_014470.1      GAVCRHHASEYRQYLDAYNMMISAGFSLWIYKQFDTYNLWNTFTRLQSLENVAYNVVNKG   6433
GQ153542.1       GAVCRHHANEYRQYLDAYNMMISAGFSLWIYKQFDTYNLWNTFTKLQSLENVAYNVVNKG   6440
DQ022305         GAVCRHHANEYRQYLDAYNMMISAGFSLWIYKQFDTYNLWNTFTKLQSLENVAYNVVNKG   6436
KF569996.1       GAVCRHHANEYRQYLDAYNMMISAGFSLWIYKQFDTYNLWNTFTRLQCLENVAYNVVNKG   6379
KP886809.1       GAVCRHHANEYRQYLDAYNMMISAGFSLWIYKQFDTYNLWNTFTRLQSLENVAYNVVNKG   6442
AY278488.2       GAVCRHHANEYRQYLDAYNMMISAGFSLWIYKQFDTYNLWNTFTRLQSLENVAYNVVNKG   6442
AY485277.1       GAVCRHHANEYRQYLDAYNMMISAGFSLWIYKQFDTYNLWNTFTRLQSLENVAYNVVNKG   6442
AP006560.1       GAVCRHHANEYRQYLDAYNMMISAGFSLWIYKQFDTYNLWNTFTRLQSLENVAYNVVNKG   6442
AP006557.1       GAVCRHHANEYRQYLDAYNMMISAGFSLWIYKQFDTYNLWNTFTRLQSLENVAYNVVNKG   6442
AY274119         GAVCRHHANEYRQYLDAYNMMISAGFSLWIYKQFDTYNLWNTFTRLQSLENVAYNVVNKG   6442
AY572038.1       GAVCRHHANEYRQYLDAYNMMISAGFSLWIYKQFDTYNLWNTFTRLQSLENVAYNVVNKG   6442
AY572034.1       GAVCRHHANEYRQYLDAYNMMISAGFSLWIYKQFDTYNLWNTFTRLQSLENVAYNVVNKG   6442
FJ588686.1       GAVCRHHANEYRQYLDAYNMMISAGFSLWIYKQFDTYNLWNTFTRLQSLENVAYNVVNKG   6249
KY417145.1       GAVCRHHANEYRQYLDAYNMMISAGFSLWIYKQFDTYNLWNTFTRLQSLENVAYNVVNKG   6442
KY417144.1       GAVCRHHANEYRQYLDAYNMMISAGFSLWIYKQFDTYNLWNTFTRLQSLENVAYNVVNKG   6442
KY417147.1       GAVCRHHANEYRQYLDAYNMMISAGFSLWIYKQFDTYNLWNTFTRLQSLENVAYNVVNKG   6442
KY417148.1       GAVCRHHANEYRQYLDAYNMMISAGFSLWIYKQFDTYNLWNTFTRLQSLENVAYNVVNKG   6442
KY417143.1       GAVCRHHANEYRQYLDAYNMMISAGFSLWIYKQFDTYNLWNTFTRLQSLENVAYNVVNKG   6442
KT444582.1       GAVCRHHANEYRQYLDAYNMMISAGFSLWIYKQFDTYNLWNTFTRLQSLENVAYNVVNKG   6442
KC881005.1       GAVCRHHANEYRQYLDAYNMMISAGFSLWIYKQFDTYNLWNTFTRLQSLENVAYNVVNKG   6442
KC881006.1       GAVCRHHANEYRQYLDAYNMMISAGFSLWIYKQFDTYNLWNTFTRLQSLENVAYNVVNKG   6442
KF367457.1       GAVCRHHANEYRQYLDAYNMMISAGFSLWIYKQFDTYNLWNTFTRLQSLENVAYNVVNKG   6442
KY417152.1       GAVCRHHANEYRQYLDAYNMMISAGFSLWIYKQFDTYNLWNTFTRLQSLENVAYNVVNKG   6442
KY417146.1       GAVCRHHANEYRQYLDAYNMMISAGFSLWIYKQFDTYNLWNTFTRLQSLENVAYNVVNKG   6442
KY417151.1       GAVCRHHANEYRQYLDAYNMMISAGFSLWIYKQFDTYNLWNTFTRLQSLENVAYNVVNKG   6442
KY417142.1       GAVCRHHANEYRQYLDAYNMMISAGFSLWIYKQFDTYNLWNTFTRLQSLENVAYNVVNKG   6442
                 ********.*** ****************:**************:**.*****:**:***
```

```
MW532698.1     HFDGQQGETPVSIVNNTVYTKVDGVDVELFENKTILPVNVAFELWAKRNIKPVPEVKILN   6517
MT040336.1     HFDGQQGETPVSIVNNTVYTKVDGVDVELFENKTTLPVNVAFELWAKRNIKPVPEVKILN   6517
MT040335.1     HFDGQQGETPVSIVNNTVYTKVDGVDVELFENKTTLPVNVAFELWAKRNIKPVPEVKILN   6517
MT040334.1     HFDGQQGETPVSIVNNTVYTKVDGVDVELFENKTTLPVNVAFELWAKRNIKPVPEVKILN   6517
MT040333.1     HFDGQQGETPVSIVNNTVYTKVDGVDVELFENKTTLPVNVAFELWAKRNIKPVPEVKILN   6517
MN996532.2     HFDGQQGEVPVSIINNTVYTKVDGVDVELFENKTTLPVNVAFELWAKRNIKPVPEVKILN   6524
MN988713.1     HFDGQQGEVPVSIINNTVYTKVDGVDVELFENKTTLPVNVAFELWAKRNIKPVPEVKILN   6525
MT093571.1     HFDGQQGEVPVSIINNTVYTKVDGVDVELFENKTTLPVNVAFELWAKRNIKPVPEVKILN   6525
MN996529.1     HFDGQQGEVPVSIINNTVYTKVDGVDVELFENKTTLPVNVAFELWAKRNIKPVPEVKILN   6525
MT072688.1     HFDGQQGEVPVSIINNTVYTKVDGVDVELFENKTTLPVNVAFELWAKRNIKPVPEVKILN   6525
NC_045512.2    HFDGQQGEVPVSIINNTVYTKVDGVDVELFENKTTLPVNVAFELWAKRNIKPVPEVKILN   6525
MN994467.1     HFDGQQGEVPVSIINNTVYTKVDGVDVELFENKTTLPVNVAFELWAKRNIKPVPEVKILN   6525
MG772933.1     HFDGQNGEAPVSIVNNAVYTKLDGVDVEIFENKTTLPVNVAFELWAKRNIKPVPEIKILN   6521
MG772934.1     HFDGQSGEAPVSIINNAVYTKVDGIDVEIFENKTTLPVNVAFELWAKRNIKPVPEIKILN   6499
NC_014470.1    HFDGQAGEKPVSIINNTVYTKVDGVDVEIFENKTTLPVNVAFELWAKRNIKPVPEIKILN   6493
GQ153542.1     HFDGQSGEAPVSIINNAVYTKVDGIDVEIFENKTTLPVNVAFELWAKRNIKPVPEIKILN   6500
DQ022305       HFDGQSGEAPVSIINNAVYTKVDGIDVEIFENKTTLPVNVAFELWAKRNIKPVPEIKILN   6496
KF569996.1     HFDGQTGEAPVSIINNAVYTKVDGVDVEIFENKTTLPVNVAFELWAKRNIKPVPEIKILN   6439
KP886809.1     HFDGQAGEAPVSIINNAVYTKVDGVDVEIFENKTTLPVNVAFELWAKRNIKPVPEIKILN   6502
AY278488.2     HFDGHAGEAPVSIINNAVYTKVDGIDVEIFENKTTLPVNVAFELWAKRNIKPVPEIKILN   6502
AY485277.1     HFDGHAGEAPVSIINNAVYTKVDGIDVEIFENKTTLPVNVAFELWAKRNIKPVPEIKILN   6502
AP006560.1     HFDGHAGEAPVSIINNAVYTKVDGIDVEIFENKTTLPVNVAFELWAKRNIKPVPEIKILN   6502
AP006557.1     HFDGHAGEAPVSIINNAVYTKVDGIDVEIFENKTTLPVNVAFELWAKRNIKPVPEIKILN   6502
AY274119       HFDGHAGEAPVSIINNAVYTKVDGIDVEIFENKTTLPVNVAFELWAKRNIKPVPEIKILN   6502
AY572038.1     HFDGHAGEAPVSIINNAVYTKVDGIDVEIFENKTTLPVNVAFELWAKRNIKPVPEIKILN   6502
AY572034.1     HFDGHAGEAPVSIINNAVYTKVDGIDVEIFENKTTLPVNVAFELWAKRNIKPVPEIKILN   6502
FJ588686.1     HFDGQAGEAPVSVINNAVYTKVDGIDVEIFENKTTLPVNVAFELWAKRNIKPVPEIKILN   6309
KY417145.1     HFDGQAGEAPVSIINNAVYTKVDGVDVEIFENKTTLPVNVAFELWAKRNIKPVPEIKILN   6502
KY417144.1     YFDGQAGEAPVSIINNAVYTKVDGVDVEIFENKTTLPVNVAFELWAKRNIKPVPEIKILN   6502
KY417147.1     HFDGQAGEAPVSIINNAVYTKVDGVDVEIFENKTTLPVNVAFELWAKRNIKPVPEIKILN   6502
KY417148.1     HFDGQAGEAPVSIINNAVYTKVDGVDVEIFENKTTLPVNVAFELWAKRNIKPVPEIKILN   6502
KY417143.1     HFDGQAGEAPVSIINNAVYTKVDGVDVEIFENKTTLPVNVAFELWAKRNIKPVPEIKILN   6502
KT444582.1     HFDGQAGEAPVSIINNAVYTKVDGIDVEIFENKTTLPVNVAFELWAKRNIKPVPEIKILN   6502
KC881005.1     HFDGQAGEAPVSIINNAVYTKVDGVDVEIFENKTTLPVNVAFELWAKRNIKPVPEIKILN   6502
KC881006.1     HFDGQAGEAPVSIINNAVYTKVDGVDVEIFENKTTLPVNVAFELWAKRNIKPVPEIKILN   6502
KF367457.1     HFDGQAGEAPVSIINNAVYTKVDGVDVEIFENKTTLPVNVAFELWAKRNIKPVPEIKILN   6502
KY417152.1     HFDGQAGEAPVSIINNAVYTKVDGVDVEIFENKTTLPVNVAFELWAKRNIKPVPEIKILN   6502
KY417146.1     HFDGQAGEAPVSIINNAVYTKVDGIDVEIFENKTTLPVNVAFELWAKRNIKPVPEIKILN   6502
KY417151.1     HFDGQAGEAPVSIINNAVYTKVDGVDVEIFENKTTLPVNVAFELWAKRNIKPVPEIKILN   6502
KY417142.1     HFDGQAGEAPVSIINNAVYTKVDGVDVEIFENKTTLPVNVAFELWAKRNIKPVPEIKILN   6502
               :***: ** ***::**:****:**:***:***** ********************:****
```

```
MW532698.1        NLGVDIAANTVIWDYKREAPAHVSTIGVCTMTDIAKKSTETACSPLTILFDGRVEGQVDL        6577
MT040336.1        NLGVDIAANTVIWDYKREAPAHVSTIGVCTMTDIAKKSTETACSPLTILFDGRVEGQVDL        6577
MT040335.1        NLGVDIAANTVIWDYKREAPAHVSTIGVCTMTDIAKKSTETACSPLTILFDGRVEGQVDL        6577
MT040334.1        NLGVDIAANTVIWDYKREAPAHVSTIGVCTMTDIAKKSTETACSPLTILFDGRVEGQVDL        6577
MT040333.1        NLGVDIAANTVIWDYKREAPAHVSTIGVCTMTDIAKKSTETACSPLTILFDGRVEGQVDL        6577
MN996532.2        NLGVDIAANTVIWDYKRDAPAHISTIGVCSMTDIAKKPTENICAPLTVFFDGRVNGQVDL        6584
MN988713.1        NLGVDIAANTVIWDYKRDAPAHISTIGVCSMTDIAKKPTETICAPLTVFFDGRVDGQVDL        6585
MT093571.1        NLGVDIAANTVIWDYKRDAPAHISTIGVCSMTDIAKKPTETICAPLTVFFDGRVDGQVDL        6585
MN996529.1        NLGVDIAANTVIWDYKRDAPAHISTIGVCSMTDIAKKPTETICAPLTVFFDGRVDGQVDL        6585
MT072688.1        NLGVDIAANTVIWDYKRDAPAHISTIGVCSMTDIAKKPTETICAPLTVFFDGRVDGQVDL        6585
NC_045512.2       NLGVDIAANTVIWDYKRDAPAHISTIGVCSMTDIAKKPTETICAPLTVFFDGRVDGQVDL        6585
MN994467.1        NLGVDIAANTVIWDYKRDAPAHISTIGVCSMTDIAKKPTETICAPLTVFFDGRVDGQVDL        6585
MG772933.1        NLGVDIAANTVIWDYKREAPAHVSTIGVCTMTDIAKKPTESACSSLTVLFDGRVEGQVDL        6581
MG772934.1        NLGVDIAANTVIWDHKREAPAHVSTIGVCTMTDIAEKPTENACSSLTVLFDGRVEGQANL        6559
NC_014470.1       NLGVDIAANTVIWDYKRESPAYISTIGVCTMTDIAKKPTENACSSLTVFFDGRVDGQVDS        6553
GQ153542.1        NLGVDIAANTVIWDYKKEAPAYVSTIGVCTMTDIAKKPTESACSSLTVLFDGRVEGQVDL        6560
DQ022305          NLGVDIAANNVIWDYKREAPAHVSTIGVCTMTDIAKKPTESACSSLIVLFDGRVEGQVDF        6556
KF569996.1        NLGVDIAANTVIWDYKREAPAHVSTIGVCTMTDIAKKPTESACSSLTVLFDGRAEGQVDL        6499
KP886809.1        NLGVDIAANTVIWDYKREAPAHMSTIGVSTMTDIAKKPTESACSSLTVLFDGRVEGQVDL        6562
AY278488.2        NLGVDIAANTVIWDYKREAPAHVSTIGVCTMTDIAKKPTESACSSLTVLFDGRVEGQVDL        6562
AY485277.1        NLGVDIAANTVIWDYKREAPAHVSTIGVCTMTDIAKKPTESACSSLTVLFDGRVEGQVDL        6562
AP006560.1        NLGVDIAANTVIWDYKREAPAHVSTIGVCTMTDIAKKPTESACSSLTVLFDGRVEGQVDL        6562
AP006557.1        NLGVDIAANTVIWDYKREAPAHVSTIGVCTMTDIAKKPTESACSSLTVLFDGRVEGQVDL        6562
AY274119          NLGVDIAANTVIWDYKREAPAHVSTIGVCTMTDIAKKPTESACSSLTVLFDGRVEGQVDL        6562
AY572038.1        NLGVDIAANTVIWDYKREAPAHVSTIGVCTMTDIAKKPTESACSSLTVLFDGRVEGQVDL        6562
AY572034.1        NLGVDIAANTVIWDYKREAPAHVSTIGVCTMTDIAKKPTESACSSLTVLFDGRVEGQVDL        6562
FJ588686.1        NLGVDIAANTVIWDYNREAPAHVSTIGVCTMTDIAKKPTESACSSLTVLFDGRVEGQVDL        6369
KY417145.1        NLGVDIAANTVVWDYKREAPAHMSTIGVCTMTDIAKKPTESACSSLTVLFDGRVEGQVDL        6562
KY417144.1        NLGVDIAANTVVWDYKREAPAHMSTIGVCTMTDIAKKPTESACSSLTVLFDGRVEGQVDL        6562
KY417147.1        NLGVDIAANTVVWDYKREAPAHMSTIGVCTMTDIAKKPTESACSSLTVLFDGRVEGQVDL        6562
KY417148.1        NLGVDIAANTVVWDYKREAPAHMSTIGVCTMTDIAKKPTESACSSLTVLFDGRVEGQVDL        6562
KY417143.1        NLGVDIAANTVVWDYKREAPAHMSTIGVCTMTDIAKKPTESACSSLTVLFDGRVEGQVDL        6562
KT444582.1        NLGVDIAANTVIWDYKREAPAHMSTIGVCTMTDIAKKPTESACSSLTVLFDGRVEGQVDL        6562
KC881005.1        NLGVDIAANTVVWDYKREAPAHMSTIGVCTMTDIAKKPTESACSSLTVLFDGRVEGQVDL        6562
KC881006.1        NLGVDIAANTVVWDYKREAPAHMSTIGVCTMTDIAKKPTESACSSLTVLFDGRVVGQADL        6562
KF367457.1        NLGVDIAANTVVWDYKREAPAHMSTIGVCTMTDIAKKPTESACSSLTVLFDGRVEGQVDL        6562
KY417152.1        NLGVDIAANTVVWDYKREAPAHMSTIGVCTMTDIAKKPTESACSSLTVLFDGRVEGQVDL        6562
KY417146.1        NLGVDIAANTVIWDYKREAPAHMSTIGVCTMTDIAKKPTESACSSLTVLFDGRVEGQVDL        6562
KY417151.1        NLGVDIAANTVVWDYKREAPAHMSTIGVCTMTDIAKKPTESACSSLTVLFDGRVEGQVDL        6562
KY417142.1        NLGVDIAANTVVWDYKREAPAHMSTIGVCTMTDIAKKPTESACSSLTVLFDGRVEGQVDL        6562
                  *********.*:**:::::**::*****.:*****:* **. *: * ::****. **.:
```

```
MW532698.1      FRNARNGVLITEGNVKGLQPSVGPKQASLNGVTLIGEAVKTQFNYYKKVDGVVQQLPETY      6637
MT040336.1      FRNARNGVLITEGNVKGLQPSVGPKQASLNGVTLIGEAVKTQFNYYKKVDGVVQQLPETY      6637
MT040335.1      FRNARNGVLITEGNVKGLQPSVGPKQASLNGVTLIGEAVKTQFNYYKKVDGVVQQLPETY      6637
MT040334.1      FRNARNGVLITEGNVKGLQPSVGPKQASLNGVTLIGEAVKTQFNYYKKVDGVVQQLPETY      6637
MT040333.1      FRNARNGVLITEGNVKGLQPSVGPKQASLNGVTLIGEAVKTQFNYYKKVDGVVQQLPETY      6637
MN996532.2      FRNARNGVLITEGSVKGLQPSVGPKQASLNGVTLIGEALKTQFNYYKKVNGVVQQLPETY      6644
MN988713.1      FRNARNGVLITEGSVKGLQPSVGPKQASLNGVTLIGEAVKTQFNYYKKVDGVVQQLPETY      6645
MT093571.1      FRNARNGVLITEGSVKGLQPSVGPKQASLNGVTLIGEAVKTQFNYYKKVDGVVQQLPETY      6645
MN996529.1      FRNARNGVLITEGSVKGLQPSVGPKQASLNGVTLIGEAVKTQFNYYKKVDGVVQQLPETY      6645
MT072688.1      FRNARNGVLITEGSVKGLQPSVGPKQASLNGVTLIGEAVKTQFNYYKKVDGVVQQLPETY      6645
NC_045512.2     FRNARNGVLITEGSVKGLQPSVGPKQASLNGVTLIGEAVKTQFNYYKKVDGVVQQLPETY      6645
MN994467.1      FRNARNGVLITEGSVKGLQPSVGPKQASLNGVTLIGEAVKTQFNYYKKVDGVVQQLPETY      6645
MG772933.1      FRNARNGVLITEGSVKGLTPSKGPAQASVNGVTLIGESVKTQFNYFKKVDGIIQQLPETY      6641
MG772934.1      FRNARNGVLITEGSVKGLTPSKGPAQASVNGVTLIGESVKTQFNYFKKVDGIIQQLPETY      6619
NC_014470.1     FRNARNGVLITEGSVKGLNPSKGPPQASLNGVTLIGESVKTQFNYFKKVDGVVQQLPETY      6613
GQ153542.1      FRNARNGVLITEGSVKGLTPSKGPIQASVNGVTLIGESVKTQFNYFKKVDGIIQQLPETY      6620
DQ022305        FRNARNGVLITEGSVKGLTPSKGPAQASVNGVTLIGESVKTQFNYFKKVDGIIQQLPETY      6616
KF569996.1      FRNARNGVLITEGSVKGLTPSKGPAQASVNGVTLIGESVKTQFNYFKKVDGIIQQLPETY      6559
KP886809.1      FRNARNGVLITEGSVKGLTPSKGPAQASVNGVTLIGESVKTQFNYFKKVDGIIQQLPETY      6622
AY278488.2      FRNARNGVLITEGSVKGLTPSKGPAQASVNGVTLIGESVKTQFNYFKKVDGIIQQLPETY      6622
AY485277.1      FRNARNGVLITEGSVKGLTPSKGPAQASVNGVTLIGESVKTQFNYFKKVDGIIQQLPETY      6622
AP006560.1      FRNARNGVLITEGSVKGLTPSKGPAQASVNGVTLIGESVKTQFNYFKKVDGIIQQLPETY      6622
AP006557.1      FRNARNGVLITEGSVKGLTPSKGPAQASVNGVTLIGESVKTQFNYFKKVDGIIQQLPETY      6622
AY274119        FRNARNGVLITEGSVKGLTPSKGPAQASVNGVTLIGESVKTQFNYFKKVDGIIQQLPETY      6622
AY572038.1      FRNARNGVLITEGSVKGLTPSKGPAQASVNGVTLIGESVKTQFNYFKKVDGIIQQLPETY      6622
AY572034.1      FRNARNGVLITEGSVKGLTPSKGPAQASVNGVTLIGESVKTQFNYFKKVDGIIQQLPETY      6622
FJ588686.1      FRNARNGVLITEGSVKGLTPSKGPAQASVNGVTLIGESVKTQFNYFKKVDGIIQQLPEAY      6429
KY417145.1      FRNARNGVLITEGSVKGLTPSKGPAQASVNGVTLIGESVKTQFNYFKKVDGIIQQLPETY      6622
KY417144.1      FRNARNGVLITEGSVKGLTPSKGPAQASVNGVTLIGESVKTQFNYFKKVDGIIQQLPETY      6622
KY417147.1      FRNARNGVLITEGSVKGLTPSKGPAQASVNGVTLIGESVKTQFNYFKKVDGIIQQLPETY      6622
KY417148.1      FRNARNGVLITEGSVKGLIPSKGPVQASVNGVTLIGESVKTQFNYFKKVDGIIQQLPETY      6622
KY417143.1      FRNARNGVLITEGSVKGLTPSKGPAQASVNGVTLIGESVKTQFNYFKKVDGIIQQLPETY      6622
KT444582.1      FRNARNGVLITEGSVKGLIPSKGPVQASVNGVTLIGESVKTQFNYFKKVDGIIQQLPETY      6622
KC881005.1      FRNARNGVLITEGSVKGLTPSKGPAQASVNGVTLIGESVKTQFNYFKKVDGIIQQLPETY      6622
KC881006.1      FRNARNGVLITEGSVKGLTPSKGPAQASVNGVTLIGESVKTQFNYFKKVDGIIQQLPETY      6622
KF367457.1      FRNARNGVLITEGSVKGLTPSKGPAQASVNGVTLIGESVKTQFNYFKKVDGIIQQLPETY      6622
KY417152.1      FRNARNGVLITEGSVKGLTPSKGPAQASVNGVTLIGESVKTQFNYFKKVDGIIQQLPETY      6622
KY417146.1      FRNARNGVLITEGSVKGLIPSKGPVQASVNGVTLIGESVKTQFNYFKKVDGIIQQLPETY      6622
KY417151.1      FRNARNGVLITEGSVKGLTPSKGPAQASVNGVTLIGESVKTQFNYFKKVDGIIQQLPETY      6622
KY417142.1      FRNARNGVLITEGSVKGLTPSKGPAQASVNGVTLIGESVKTQFNYFKKVDGIIQQLPETY      6622
                *************.**** ** ** ***:********::******:***:*::*****:*
```

```
MW532698.1      FTQSRNLQEFKPRSQMEIDFLELAMDEFIERYKLEGYAFEHIVYGDFSHGQLGGLHLLIG      6697
MT040336.1      FTQSRNLQEFKPRSQMEIDFLELAMDEFIERYKLEGYAFEHIVYGDFSHGQLGGLHLLIG      6697
MT040335.1      FTQSRNLQEFKPRSQMEIDFLELAMDEFIERYKLEGYAFEHIVYGDFSHGQLGGLHLLIG      6697
MT040334.1      FTQSRNLQEFKPRSQMEIDFLELAMDEFIERYKLEGYAFEHIVYGDFSHGQLGGLHLLIG      6697
MT040333.1      FTQSRNLQEFKPRSQMEIDFLELAMDEFIERYKLEGYAFEHIVYGDFSHGQLGGLHLLIG      6697
MN996532.2      FTQSRNLKEFKPRSQMEIDFLELAMDEFIERYKLEGYAFEHIVYGDFSHRQLGGLHLLIG      6704
MN988713.1      FTQSRNLQEFKPRSQMEIDFLELAMDEFIERYKLEGYAFEHIVYGDFSHSQLGGLHLLIG      6705
MT093571.1      FTQSRNLQEFKPRSQMEIDFLELAMDEFIERYKLEGYAFEHIVYGDFSHSQLGGLHLLIG      6705
MN996529.1      FTQSRNLQEFKPRSQMEIDFLELAMDEFIERYKLEGYAFEHIVYGDFSHSQLGGLHLLIG      6705
MT072688.1      FTQSRNLQEFKPRSQMEIDFLELAMDEFIERYKLEGYAFEHIVYGDFSHSQLGGLHLLIG      6705
NC_045512.2     FTQSRNLQEFKPRSQMEIDFLELAMDEFIERYKLEGYAFEHIVYGDFSHSQLGGLHLLIG      6705
MN994467.1      FTQSRNLQEFKPRSQMEIDFLELAMDEFIERYKLEGYAFEHIVYGDFSHSQLGGLHLLIG      6705
MG772933.1      FTQSRDLEDFKPRSQMETDFLELAMDEFIERYKLDGYAFEHIVYGDFSHGQLGGLHLLIG      6701
MG772934.1      FTQSRDLEDFKPRSQMETDFLELAMDEFIERYKLDGYAFEHIVYGDFSHGQLGGLHLLIG      6679
NC_014470.1     FTQSRSLDDFKPRSQMEVDFLQLAMDEFIERYKLEGYAFEHIVYGDFSHGQLGGLHLMIG      6673
GQ153542.1      FTQSRDLEDFKPRSQMETDFLELAMDEFIQRYKLEGYAFEHIVYGDFSHGQLGGLHLMIG      6680
DQ022305        FTQSRDLEDFKPRSQMETDFLELAMDEFIQRYKLEGYAFEHIVYGDFSHGQLGGLHLMIG      6676
KF569996.1      FTQSRDLEDFKPRSQMETDFLELAMDEFIQRYKLEGYAFEHIVYGDFSHGQLGGLHLMIG      6619
KP886809.1      FTQSRDLEDFKPRSQMETDFLELAMDEFIQRYKLEGYAFEHIVYGDFSHGQLGGLHLMIG      6682
AY278488.2      FTQSRDLEDFKPRSQMETDFLELAMDEFIQRYKLEGYAFEHIVYGDFSHGQLGGLHLMIG      6682
AY485277.1      FTQSRDLEDFKPRSQMETDFLELAMDEFIQRYKLEGYAFEHIVYGDFSHGQLGGLHLMIG      6682
AP006560.1      FTQSRDLEDFKPRSQMETDFLELAMDEFIQRYKLEGYAFEHIVYGDFSHGQLGGLHLMIG      6682
AP006557.1      FTQSRDLEDFKPRSQMETDFLELAMDEFIQRYKLEGYAFEHIVYGDFSHGQLGGLHLMIG      6682
AY274119        FTQSRDLEDFKPRSQMETDFLELAMDEFIQRYKLEGYAFEHIVYGDFSHGQLGGLHLMIG      6682
AY572038.1      FTQSRDLEDFKPRSQMETDFLELAMDEFIQRYKLEGYAFEHIVYGDFSHGQLGGLHLMIG      6682
AY572034.1      FTQSRDLEDFKPRSQMETDFLELAMDEFIQRYKLEGYAFEHIVYGDFSHGQLGGLHLMIG      6682
FJ588686.1      FTQSRDLENFKPRSQMETDFLELAMDEFIQRYKLEGYAFEHIVYGDFSHGQLGGLHLMIG      6489
KY417145.1      FTQSRDLEDFKPRSQMETDFLELAMDEFIQRYKLEGYAFEHIVYGDFSHGQLGGLHLMIG      6682
KY417144.1      FTQSRDLEDFKPRSQMETDFLELAMDEFIQRYKLEGYAFEHIVYGDFSHGQLGGLHLMIG      6682
KY417147.1      FTQSRDLEDFKPRSQMETDFLELAMDEFIQRYKLEGYAFEHIVYGDFSHGQLGGLHLMIG      6682
KY417148.1      FTQSRDLEDFKPRSQMETDFLELAMDEFIQRYKLEGYAFEHIVYGDFSHGQLGGLHLMIG      6682
KY417143.1      FTQSRDLEDFKPRSQMETDFLELAMDEFIQRYKLEGYAFEHIVYGDFSHGQLGGLHLMIG      6682
KT444582.1      FTQSRDLEDFRPRSQMETDFLELAMDEFIQRYKLEGYAFEHIVYGDFSHGQLGGLHLMIG      6682
KC881005.1      FTQSRDLEDFKPRSQMETDFLELAMDEFIQRYKLEGYAFEHIVYGDFSHGQLGGLHLMIG      6682
KC881006.1      FTQSRDLEDFKPRSQMETDFLELAMDEFIQRYKLEGYAFEHIVYGDFSHGQLGGLHLMIG      6682
KF367457.1      FTQSRDLEDFKPRSQMETDFLELAMDEFIQRYKLEGYAFEHIVYGDFSHGQLGGLHLMIG      6682
KY417152.1      FTQSRDLEDFKPRSQMETDFLELAMDEFIQRYKLEGYAFEHIVYGDFSHGQLGGLHLMIG      6682
KY417146.1      FTQSRDLEDFKPRSQMETDFLELAMDEFIQRYKLEGYAFEHIVYGDFSHGQLGGLHLMIG      6682
KY417151.1      FTQSRDLEDFKPRSQMETDFLELAMDEFIQRYKLEGYAFEHIVYGDFSHGQLGGLHLMIG      6682
KY417142.1      FTQSRDLEDFKPRSQMETDFLELAMDEFIQRYKLEGYAFEHIVYGDFSHGQLGGLHLMIG      6682
                *****.*.:*:****** ***:*******:****:************** *******:**
```

```
MW532698.1      LAKRSKDSPLELEDFIPMDSTVKNYFITDAQTGSSKCVCSVIDLLLDDFVEIIKSQDLSV    6757
MT040336.1      LAKRSKDSPLELEDFIPMDSTVKNYFITDAQTGSSKCVCSVIDLLLDDFVEIIKSQDLSV    6757
MT040335.1      LAKRSKDSPLELEDFIPMDSTVKNYFITDAQTGSSKCVCSVIDLLLDDFVEIIKSQDLSV    6757
MT040334.1      LAKRSKDSPLELEDFIPMDSTVKNYFITDAQTGSSKCVCSVIDLLLDDFVEIIKSQDLSV    6757
MT040333.1      LAKRSKDSPLELEDFIPMDSTVKNYFITDAQTGSSKCVCSVIDLLLDDFVEIIKSQDLSV    6757
MN996532.2      LAKRSKESPLELEDFIPMDSTVKNYFITDAQTGSSKCVCSVIDLLLDDFVEIIKSQDLSV    6764
MN988713.1      LAKRFKESPFELEDFIPMDSTVKNYFITDAQTGSSKCVCSVIDLLLDDFVEIIKSQDLSV    6765
MT093571.1      LAKRFKESPFELEDFIPMDSTVKNYFITDAQTGSSKCVCSVIDLLLDDFVEIIKSQDLSV    6765
MN996529.1      LAKRFKESPFELEDFIPMDSTVKNYFITDAQTGSSKCVCSVIDLLLDDFVEIIKSQDLSV    6765
MT072688.1      LAKRFKESPFELEDFIPMDSTVKNYFITDAQTGSSKCVCSVIDLLLDDFVEIIKSQDLSV    6765
NC_045512.2     LAKRFKESPFELEDFIPMDSTVKNYFITDAQTGSSKCVCSVIDLLLDDFVEIIKSQDLSV    6765
MN994467.1      LAKRFKESPFELEDFIPMDSTVKNYFITDAQTGSSKCVCSVIDLLLDDFVEIIKSQDLSV    6765
MG772933.1      LAKRSQDSLLKLEDFIPMDSTVKNYFITDAQTGSSKCVCSVIDLLLDDFVEIIKSQDLSV    6761
MG772934.1      LAKRSQDSLLKLEDFIPMDSTVKNYFITDAQTGSSKCVCSVIDLLLDDFVEIIKSQDLSV    6739
NC_014470.1     LAKRSLESLLKLEDFIPIDSTVKNYFVTDAQTGSSKCVCSVIDLLLDDFVEIIKSQDLSV    6733
GQ153542.1      LAKRSQDSPLKLEDFIPMDSTVKNYFITDAQTGSSKCVCSVIDLLLDDFVEIIKAQDLSV    6740
DQ022305        LAKRSQDSLLKLEDFIPMDSTVKNYFITDAQTGSSKCVCSVIDLLLDDFVEIIKSQDLSV    6736
KF569996.1      LAKRSQDSPLKLEDFIPMDSTVKNYFITDAQTGSSKCVCSVIDLLLDDFVEIIKSQDLSV    6679
KP886809.1      LAKRSQDSPLKLEDFIPMDSTVKNYFITDAQTGSSKCVCSVIDLLLDDFVEIIKSQDLSV    6742
AY278488.2      LAKRSQDSPLKLEDFIPMDSTVKNYFITDAQTGSSKCVCSVIDLLLDDFVEIIKSQDLSV    6742
AY485277.1      LAKRSQDSPLKLEDFIPMDSTVKNYFITDAQTGSSKCVCSVIDLLLDDFVEIIKSQDLSV    6742
AP006560.1      LAKRSQDSPLKLEDFIPMDSTVKNYFITDAQTGSSKCVCSVIDLLLDDFVEIIKSQDLSV    6742
AP006557.1      LAKRSQDSPLKLEDFIPMDSTVKNYFITDAQTGSSKCVCSVIDLLLDDFVEIIKSQDLSV    6742
AY274119        LAKRSQDSPLKLEDFIPMDSTVKNYFITDAQTGSSKCVCSVIDLLLDDFVEIIKSQDLSV    6742
AY572038.1      LAKRSQDSPLKLEDFIPMDSTVKNYFITDAQTGSSKCVCSVIDLLLDDFVEIIKSQDLSV    6742
AY572034.1      LAKRSQDSPLKLEDFIPMDSTVKNYFITDAQTGSSKCVCSVIDLLLDDFVEIIKSQDLSV    6742
FJ588686.1      LAKRSQDSPLKLEDFIPMDSTVKNYFITDAQTGSSKCVCSVIDLLLDDFVEIIKSQDLSV    6549
KY417145.1      LAKRSQDSPLKLEDFIPMDSTVKNYFITDAQTGSSKCVCSVIDLLLDDFVEIIKSQDLSV    6742
KY417144.1      LAKRSQDSPLKLEDFIPMDSTVKNYFITDAQTGSSKCVCSVIDLLLDDFVEIIKSQDLSV    6742
KY417147.1      LAKRSQDSPLKLEDFIPMDSTVKNYFITDAQTGSSKCVCSVIDLLLDDFVEIIKSQDLSV    6742
KY417148.1      LAKRSQDSPLKLEDFIPMDSTVKNYFITDAQTGSSKCVCSVIDLLLDDFVEIIKSQDLSV    6742
KY417143.1      LAKRSQDSPLKLEDFIPMDSTVKNYFITDAQTGSSKCVCSVIDLLLDDFVEIIKSQDLSV    6742
KT444582.1      LAKRSQDSPLKLEDFIPMDSTVKNYFITDAQTGSSKCVCSVIDLLLDDFVEIIKSQDLSV    6742
KC881005.1      LAKRSQDSPLKLEDFIPMDSTVKNYFITDAQTGSSKCVCSVIDLLLDDFVEIIKSQDLSV    6742
KC881006.1      LAKRSQDSPLKLEDFIPMDSTVKNYFITDAQTGSSKCVCSVIDLLLDDFVEIIKSQDLSV    6742
KF367457.1      LAKRSQDSPLKLEDFIPMDSTVKNYFITDAQTGSSKCVCSVIDLLLDDFVEIIKSQDLSV    6742
KY417152.1      LAKRSQDSPLKLEDFIPMDSTVKNYFITDAQTGSSKCVCSVIDLLLDDFVEIIKSQDLSV    6742
KY417146.1      LAKRSQDSPLKLEDFIPMDSTVKNYFITDAQTGSSKCVCSVIDLLLDDFVEIIKSQDLSV    6742
KY417151.1      LAKRSQDSPLKLEDFIPMDSTVKNYFITDAQTGSSKCVCSVIDLLLDDFVEIIKSQDLSV    6742
KY417142.1      LAKRSQDSPLKLEDFIPMDSTVKNYFITDAQTGSSKCVCSVIDLLLDDFVEIIKSQDLSV    6742
                ****  :* ::******:********:***************************:*****
```

```
MW532698.1        VSKVVKVTIDYAEIAFMLWCKDGHVETFYPKLQSSQAWQPGVAMPNLYKMQRMLLDKCDL         6817
MT040336.1        VSKVVKVTIDYAEIAFMLWCKDGHVETFYPKLQSSQAWQPGVAMPNLYKMQRMLLDKCDL         6817
MT040335.1        VSKVVKVTIDYAEIAFMLWCKDGHVETFYPKLQSSQAWQPGVAMPNLYKMQRMLLDKCDL         6817
MT040334.1        VSKVVKVTIDYAEIAFMLWCKDGHVETFYPKLQSSQAWQPGVAMPNLYKMQRMLLDKCDL         6817
MT040333.1        VSKVVKVTIDYAEIAFMLWCKDGHVETFYPKLQSSQAWQPGVAMPNLYKMQRMLLDKCDL         6817
MN996532.2        VSKVVKVTIDYTEISFMLWCKDGHVETFYPKLQSSQAWQPGVAMPNLYKMQRMLLEKCDL         6824
MN988713.1        VSKVVKVTIDYTEISFMLWCKDGHVETFYPKLQSSQAWQPGVAMPNLYKMQRMLLEKCDL         6825
MT093571.1        VSKVVKVTIDYTEISFMLWCKDGHVETFYPKLQSSQAWQPGVAMPNLYKMQRMLLEKCDL         6825
MN996529.1        VSKVVKVTIDYTEISFMLWCKDGHVETFYPKLQSSQAWQPGVAMPNLYKMQRMLLEKCDL         6825
MT072688.1        VSKVVKVTIDYTEISFMLWCKDGHVETFYPKLQSSQAWQPGVAMPNLYKMQRMLLEKCDL         6825
NC_045512.2       VSKVVKVTIDYTEISFMLWCKDGHVETFYPKLQSSQAWQPGVAMPNLYKMQRMLLEKCDL         6825
MN994467.1        VSKVVKVTIDYTEISFMLWCKDGHVETFYPKLQSSQAWQPGVAMPNLYKMQRMLLEKCDL         6825
MG772933.1        VSKVVKVTIDYTEISFMLWCKDGHVETFYPKLQSSQAWQPGVAMPNLYKMQRMLLEKCDL         6821
MG772934.1        VSKVVKVTIDYTEISFMLWCKDGHVETFYPKLQSSHAWQPGVAMPNLYKMQRMLLEKCDL         6799
NC_014470.1       VSKVVTVTIDYAEISFMLWCKDGHVETFYPKLQANQTWQPGVAMPNLYKMQRMLLDKCDL         6793
GQ153542.1        ISKVVKVTIDYAEISFMLWCKNGHVETFYPKLQASQAWQPGVAMPNLYKMQRMLLEKCDL         6800
DQ022305          VSKVVKVTIDYAEISFMLWCKDGHVETFYPKLQASQAWQPGVAMPNLYKMQRMLLEKCDL         6796
KF569996.1        VSKVVKVTIDYAEISFMLWCKDGHVETFYPKLQASQAWQPGVAMPNLYKMQRMLLEKCDL         6739
KP886809.1        ISKVVKVTIDYVEISFMLWCKDGHVETFYPKLQASQAWQPGVAMPNLYKMQRMLLEKCDL         6802
AY278488.2        ISKVVKVTIDYAEISFMLWCKDGHVETFYPKLQASQAWQPGVAMPNLYKMQRMLLEKCDL         6802
AY485277.1        ISKVVKVTIDYAEISFMLWCKDGHVETFYPKLQASQAWQPGVAMPNLYKMQRMLLEKCDL         6802
AP006560.1        ISKVVKVTIDYAEISFMLWCKDGHVETFYPKLQASQAWQPGVAMPNLYKMQRMLLEKCDL         6802
AP006557.1        ISKVVKVTIDYAEISFMLWCKDGHVETFYPKLQASQAWQPGVAMPNLYKMQRMLLEKCDL         6802
AY274119          ISKVVKVTIDYAEISFMLWCKDGHVETFYPKLQASQAWQPGVAMPNLYKMQRMLLEKCDL         6802
AY572038.1        ISKVVKVTIDYAEISFMLWCKDGHVETFYPKLQASQAWQPGVAMPNLYKMQRMLLEKCDL         6802
AY572034.1        ISKVVKVTIDYAEISFMLWCKDGHVETFYPKLQASQAWQPGVAMPNLYKMQRMLLEKCDL         6802
FJ588686.1        ISKVVKVTIDYAEISFMLWCKDGHVETFYPKLQASQAWQPGVAMPNLYKMQRMLLEKCDL         6609
KY417145.1        ISKVVKVTIDYAEISFMLWCKDGHVETFYPKLQASQAWQPGVAMPNLYKMQRMLLEKCDL         6802
KY417144.1        ISKVVKVTIDYAEISFMLWCKDGHVETFYPKLQASQAWQPGVAMPNLYKMQRMLLEKCDL         6802
KY417147.1        ISKVVKVTIDYAEISFMLWCKDGHVETFYPKLQASQAWQPGVAMPNLYKMQRMLLEKCDL         6802
KY417148.1        ISKVVKVTIDYAEISFMLWCKDGHVETFYPKLQASQAWQPGVAMPNLYKMQRMLLEKCDL         6802
KY417143.1        ISKVVKVTIDYAEISFMLWCKDGHVETFYPKLQASQAWQPGVAMPNLYKMQRMLLEKCDL         6802
KT444582.1        ISKVVKVTIDYAEISFMLWCKDGHVETFYPKLQASQAWQPGVAMPNLYKMQRMLLEKCDL         6802
KC881005.1        ISKVVKVTIDYAEISFMLWCKDGHVETFYPKLQASQAWQPGVAMPNLYKMQRMLLEKCDL         6802
KC881006.1        ISKVVKVTIDYAEISFMLWCKDGHVETFYPKLQASQAWQPGVAMPNLYKMQRMLLEKCDL         6802
KF367457.1        ISKVVKVTIDYAEISFMLWCKDGHVETFYPKLQASQAWQPGVAMPNLYKMQRMLLEKCDL         6802
KY417152.1        ISKVVKVTIDYAEISFMLWCKDGHVETFYPKLQASQAWQPGVAMPNLYKMQRMLLEKCDL         6802
KY417146.1        ISKVVKVTIDYAEISFMLWCKDGHVETFYPKLQASQAWQPGVAMPNLYKMQRMLLEKCDL         6802
KY417151.1        ISKVVKVTIDYAEISFMLWCKDGHVETFYPKLQASQAWQPGVAMPNLYKMQRMLLEKCDL         6802
KY417142.1        ISKVVKVTIDYAEISFMLWCKDGHVETFYPKLQASQAWQPGVAMPNLYKMQRMLLEKCDL         6802
                  :****.*****.**:******:***********:.::******************:****
```

MW532698.1	QNYGEAATLPKGIMMNVAKYTQLCQYLNTLTLAVPYNMRVIHFGAGSDKGVAPGTAVLRQ	6877
MT040336.1	QNYGEAATLPKGIMMNVAKYTQLCQYLNTLTLAVPYNMRVIHFGAGSDKGVAPGTAVLRQ	6877
MT040335.1	QNYGEAATLPKGIMMNVAKYTQLCQYLNTLTLAVPYNMRVIHFGAGSDKGVAPGTAVLRQ	6877
MT040334.1	QNYGEAATLPKGIMMNVAKYTQLCQYLNTLTLAVPYNMRVIHFGAGSDKGVAPGTAVLRQ	6877
MT040333.1	QNYGEAATLPKGIMMNVAKYTQLCQYLNTLTLAVPYNMRVIHFGAGSDKGVAPGTAVLRQ	6877
MN996532.2	QNYGDSATLPKGIMMNVAKYTQLCQYLNTLTLAVPYNMRVIHFGAGSDKGVAPGTAVLRQ	6884
MN988713.1	QNYGDSATLPKGIMMNVAKYTQLCQYLNTLTLAVPYNMRVIHFGAGSDKGVAPGTAVLRQ	6885
MT093571.1	QNYGDSATLPKGIMMNVAKYTQLCQYLNTLTLAVPYNMRVIHFGAGSDKGVAPGTAVLRQ	6885
MN996529.1	QNYGDSATLPKGIMMNVAKYTQLCQYLNTLTLAVPYNMRVIHFGAGSDKGVAPGTAVLRQ	6885
MT072688.1	QNYGDSATLPKGIMMNVAKYTQLCQYLNTLTLAVPYNMRVIHFGAGSDKGVAPGTAVLRQ	6885
NC_045512.2	QNYGDSATLPKGIMMNVAKYTQLCQYLNTLTLAVPYNMRVIHFGAGSDKGVAPGTAVLRQ	6885
MN994467.1	QNYGDSATLPKGIMMNVAKYTQLCQYLNTLTLAVPYNMRVIHFGAGSDKGVAPGTAVLRQ	6885
MG772933.1	QNYGDSAILPKGIMMNVAKYTQLCQYLNTLTLAVPYNMRVIHFGAGSDKGVAPGTAVLRQ	6881
MG772934.1	QNYGDSATLPKGIMMNVAKYTQLCQYLNTLTLAVPYNMRVIHFGAGSDKGVAPGTAVLRQ	6859
NC_014470.1	HNYGENAVIPKGIMMNVAKYTQLCQYLNTLTIAVPYNMRVIHFGAGSDKGVAPGSAVLKQ	6853
GQ153542.1	QNYGENAVIPKGIMMNVAKYTQLCQYLNTLTLAVPYNMRVIHFGAGSDKGVAPGTAVLRQ	6860
DQ022305	QNYGENAVIPKGIMMNVAKYTQLCQYLNTLTLAVPYNMRVIHFGAGSDKGVAPGTAVLRQ	6856
KF569996.1	QNYGENAVIPKGIMMNVAKYTQLCQYLNTLTLAVPYNMRVIHFGAGSDKGVAPGTAVLRQ	6799
KP886809.1	QNYGENAVIPKGIMRNVAKYTQLCQYLNTLTLAVPYNMRVIHFGAGSDKGVAPGTAVLRQ	6862
AY278488.2	QNYGENAVIPKGIMMNVAKYTQLCQYLNTLTLAVPYNMRVIHFGAGSDKGVAPGTAVLRQ	6862
AY485277.1	QNYGENAVIPKGIMMNVAKYTQLCQYLNTLTLAVPYNMRVIHFGAGSDKGVAPGTAVLRQ	6862
AP006560.1	QNYGENAVIPKGIMMNVAKYTQLCQYLNTLTLAVPYNMRVIHFGAGSDKGVAPGTAVLRQ	6862
AP006557.1	QNYGENAVIPKGIMMNVAKYTQLCQYLNTLTLAVPYNMRVIHFGAGSDKGVAPGTAVLRQ	6862
AY274119	QNYGENAVIPKGIMMNVAKYTQLCQYLNTLTLAVPYNMRVIHFGAGSDKGVAPGTAVLRQ	6862
AY572038.1	QNYGENAVIPKGIMMNVAKYTQLCQYLNTLTLAVPYNMRVIHFGAGSDKGVAPGTAVLRQ	6862
AY572034.1	QNYGENAVIPKGIMMNVAKYTQLCQYLNTLTLAVPYNMRVIHFGAGSDKGVAPGTAVLRQ	6862
FJ588686.1	QNYGENAVIPKGIMMNVAKYTQLCQYLNTLTLAVPYNMRVIHFGAGSDKGVAPGTAVLRQ	6669
KY417145.1	QNYGENAVIPKGIMMNVAKYTQLCQYLNTLTLAVPYNMRVIHFGAGSDKGVAPGTAVLRQ	6862
KY417144.1	QNYGENAVIPKGIMMNVAKYTQLCQYLNTLTLAVPYNMRVIHFGAGSDKGVAPGTAVLRQ	6862
KY417147.1	QNYGENAVIPKGIMMNVAKYTQLCQYLNTLTLAVPYNMRVIHFGAGSDKGVAPGTAVLRQ	6862
KY417148.1	QNYGENAVIPKGIMMNVAKYTQLCQYLNTLTLAVPYNMRVIHFGAGSDKGVAPGTAVLRQ	6862
KY417143.1	QNYGENAVIPKGIMMNVAKYTQLCQYLNTLTLAVPYNMRVIHFGAGSDKGVAPGTAVLRQ	6862
KT444582.1	RNYGENAVIPKGIMMNVAKYTQLCQYLNTLTLAVPYNMRVIHFGAGSDKGVAPGTAVLRQ	6862
KC881005.1	QNYGENAVIPKGIMMNVAKYTQLCQYLNTLTLAVPYNMRVIHFGAGSDKGVAPGTAVLRQ	6862
KC881006.1	QNYGENAVIPKGIMMNVAKYTQLCQYLNTLTLAVPYNMRVIHFGAGSDKGVAPGTAVLRQ	6862
KF367457.1	QNYGENAVIPKGIMMNVAKYTQLCQYLNTLTLAVPYNMRVIHFGAGSDKGVAPGTAVLRQ	6862
KY417152.1	QNYGENAVIPKGIMMNVAKYTQLCQYLNTLTLAVPYNMRVIHFGAGSDKGVAPGTAVLRQ	6862
KY417146.1	QNYGENAVIPKGIMMNVAKYTQLCQYLNTLTLAVPYNMRVMHFGAGSDKGVAPGTAVLRQ	6862
KY417151.1	QNYGENAVIPKGIMMNVAKYTQLCQYLNTLTLAVPYNMRVIHFGAGSDKGVAPGTAVLRQ	6862
KY417142.1	QNYGENAVIPKGIMMNVAKYTQLCQYLNTLTLAVPYNMRVIHFGAGSDKGVAPGTAVLRQ	6862
	:***: * :***** ****************:********:*************:***:*	

```
MW532698.1      WLPTGTLLVDSDLNDFVSDADSTLIGDCATVHTANKWDLIISDMYDPKTKHVTRENDSKE    6937
MT040336.1      WLPTGTLLVDSDLNDFVSDADSTLIGDCATVHTANKWDLIISDMYDPKTKHVTRENDSKE    6937
MT040335.1      WLPTGTLLVDSDLNDFVSDADSTLIGDCATVHTANKWDLIISDMYDPKTKHVTRENDSKE    6937
MT040334.1      WLPTGTLLVDSDLNDFVSDADSTLIGDCATVHTANKWDLIISDMYDPKTKHVTRENDSKE    6937
MT040333.1      WLPTGTLLVDSDLNDFVSDADSTLIGDCATVHTANKWDLIISDMYDPKTKHVTRENDSKE    6937
MN996532.2      WLPTGTLLVDSDLNDFVSDADSTLIGDCATVHTANKWDLIISDMYDPKTKNVTKENDSKE    6944
MN988713.1      WLPTGTLLVDSDLNDFVSDADSTLIGDCATVHTANKWDLIISDMYDPKTKNVTKENDSKE    6945
MT093571.1      WLPTGTLLVDSDLNDFVSDADSTLIGDCATVHTANKWDLIISDMYDPKTKNVTKENDSKE    6945
MN996529.1      WLPTGTLLVDSDLNDFVSDADSTLIGDCATVHTANKWDLIISDMYDPKTKNVTKENDSKE    6945
MT072688.1      WLPTGTLLVDSDLNDFVSDADSTLIGDCATVHTANKWDLIISDMYDPKTKNVTKENDSKE    6945
NC_045512.2     WLPTGTLLVDSDLNDFVSDADSTLIGDCATVHTANKWDLIISDMYDPKTKNVTKENDSKE    6945
MN994467.1      WLPTGTLLVDSDLNDFVSDADSTLIGDCATVHTANKWDLIISDMYDPKTKNVTKENDSKE    6945
MG772933.1      WLPTGTLLVDSDLNDFVSDADSTLIGHCATVHTANKWDLIISDMYDPKTKNVTKENDSKE    6941
MG772934.1      WLPTGTLLVDSDLNDFVSDADSTLIGDCATVHTANKWDLIISDMYDPKTKNVTKENDSKE    6919
NC_014470.1     WLPVGTLLVDSDINDFVSDADSTLIGDCSTVYTANKWDLIISDMYDPKTKHILKENDSKE    6913
GQ153542.1      WLPTGTLLVDSDLNDFVSDADSTLIGDCATVHTANKWDLIISDMYDPKTKHVTKENDSKE    6920
DQ022305        WLPTGTLLVDSDLNDFVSDADSTLIGDCATVHTANKWDLIISDMYDPKTKHVLKDNDSKE    6916
KF569996.1      WLPIGTLLVDSDLNDFVSDADSTLIGDCATVHTANKWDLIISDMYDPKTKHVTKENDSKE    6859
KP886809.1      WLPTGTLLVDSDLNDFVSDADSTLIGDCATVHTANKWDLIISDMYDPKTKHVTKENDSKE    6922
AY278488.2      WLPTGTLLVDSDLNDFVSDADSTLIGDCATVHTANKWDLIISDMYDPRTKHVTKENDSKE    6922
AY485277.1      WLPTGTLLVDSDLNDFVSDADSTLIGDCATVHTANKWDLIISDMYDPRTKHVTKENDSKE    6922
AP006560.1      WLPTGTLLVDSDLNDFVSDADSTLIGDCATVHTANKWDLIISDMYDPRTKHVTKENDSKE    6922
AP006557.1      WLPTGTLLVDSDLNDFVSDADSTLIGDCATVHTANKWDLIISDMYDPRTKHVTKENDSKE    6922
AY274119        WLPTGTLLVDSDLNDFVSDADSTLIGDCATVHTANKWDLIISDMYDPRTKHVTKENDSKE    6922
AY572038.1      WLPTGTLLVDSDLNDFVSDADSTLIGDCATVHTANKWDLIISDMYDPRTKHVTKENDSKE    6922
AY572034.1      WLPTGTLLVDSDLNDFVSDADSTLIGDCATVHTANKWDLIISDMYDPRTKHVTKENDSKE    6922
FJ588686.1      WLPTGTLLVDSDLNDFVSDADSTLIGDCATVHTANKWDLIISDMYDPKTKHVTKENDSKE    6729
KY417145.1      WLPTGTLLVDSDLNDFVSDADSTLIGDCATVHTANKWDLIISDMYDPKTKHVARENDSKE    6922
KY417144.1      WLPTGTLLVDSDLNDFVSDADSTLIGDCATVHTANKWDLIISDMYDPKTKHVTKENDSKE    6922
KY417147.1      WLPTGTLLVDSDLNDFVSDADSTLIGDCATVHTANKWDLIISDMYDPKTKHVTKENDSKE    6922
KY417148.1      WLPTGTLLVDSDLNDFVSDADSTLIGDCATVHTANKWDLIISDMYDPKTKHVTKENDSKE    6922
KY417143.1      WLPTGTLLVDSDLNDFVSDADSTLIGDCATVHTANKWDLIISDMYDPKTKHVTKENDSKE    6922
KT444582.1      WLPTGTLLVDSDLNDFVSDADSTLIGDCATVHTANKWDLIISDMYDPKTKHVTRENDSKE    6922
KC881005.1      WLPTGTLLVDSDLNDFVSDADSTLIGDCATVHTANKWDLIISDMYDPKTKHVTKENDSKE    6922
KC881006.1      WLPTGTLLVDSDLNDFVSDADSTLIGDCATVHTANKWDLIISDMYDPKTKHVTKENDSKE    6922
KF367457.1      WLPTGTLLVDSDLNDFVSDADSTLIGDCATVHTANKWDLIISDMYDPKTKHVTKENDSKE    6922
KY417152.1      WLPTGTLLVDSDLNDFVSDADSTLIGDCATVHTANKWDLIISDMYDPKTKHVTKENDSKE    6922
KY417146.1      WLPTGTLLVDSDLNDFVSDADSTLIGDCATVHTANKWDLIISDMYDPKTKHVTRENDSKE    6922
KY417151.1      WLPTGTLLVDSDLNDFVSDADSTLIGDCATVHTANKWDLIISDMYDPKTKHVTKENDSKE    6922
KY417142.1      WLPTGTLLVDSDLNDFVSDADSTLIGDCATVHTANKWDLIISDMYDPKTKHVTKENDSKE    6922
                *** ********:*************.*:**:***************:**:: ::*****
```

```
MW532698.1        GFFTYICGFIQQKLALGGSVAIKITEHSWNADLYKLMGHFAWWTAFVTNVNASSSEAFLI      6997
MT040336.1        GFFTYICGFIQQKLALGGSVAIKITEHSWNADLYKLMGHFAWWTAFVTNVNASSSEAFLI      6997
MT040335.1        GFFTYICGFIQQKLALGGSVAIKITEHSWNADLYKLMGHFAWWTAFVTNVNASSSEAFLI      6997
MT040334.1        GFFTYICGFIQQKLALGGSVAIKITEHSWNADLYKLMGHFAWWTAFVTNVNASSSEAFLI      6997
MT040333.1        GFFTYICGFIQQKLALGGSVAIKITEHSWNADLYKLMGHFAWWTAFVTNVNASSSEAFLI      6997
MN996532.2        GFFTYICGFIQQKLALGGSVAIKITEHSWNADLYKLMGHFAWWTAFVTNVNASSSEAFLI      7004
MN988713.1        GFFTYICGFIQQKLALGGSVAIKITEHSWNADLYKLMGHFAWWTAFVTNVNASSSEAFLI      7005
MT093571.1        GFFTYICGFIQQKLALGGSVAIKITEHSWNADLYKLMGHFAWWTAFVTNVNASSSEAFLI      7005
MN996529.1        GFFTYICGFIQQRLALGGSVAIKITEHSWNADLYKLMGHFAWWTAFVTNVNASSSEAFLI      7005
MT072688.1        GFFTYICGFIQQKLALGGSVAIKITEHSWNADLYKLMGHFAWWTAFVTNVNASSSEAFLI      7005
NC_045512.2       GFFTYICGFIQQKLALGGSVAIKITEHSWNADLYKLMGHFAWWTAFVTNVNASSSEAFLI      7005
MN994467.1        GFFTYICGFIQQKLALGGSVAIKITEHSWNADLYKLMGHFAWWTAFVTNVNASSSEAFLI      7005
MG772933.1        GFFTYICGFIQQKLALGGSVAVKITEHSWNADLYKLMGHFAWWTAFVTNVNASSSEAFLI      7001
MG772934.1        GFFTYICGFIQQKLALGGSVAVKITEHSWNADLYKLMGYFAWWTAFVTNVNASSSEAFLI      6979
NC_014470.1       GFFTYLCGFIKQKLALGGSVAIKITEHSWNADLYKLMGYFSWWTAFVTNVNASSSEAFLI      6973
GQ153542.1        GFFTYLCGFIKQKLALGGSVAVKITEHSWNADLYKLMGHFSWWTAFVTNVNASSSEAFLI      6980
DQ022305          GFFTYLCGFIKQKLALGGSVAVKITEHSWNADLYKLMGHFSWWTAFVTNVNASSSEAFLI      6976
KF569996.1        GFFTYLCGFIKQKLALGGSVAVKITEHSWNADLYKLMGHFSWWTAFVTNVNASSSEAFLI      6919
KP886809.1        GFFTYLCGFIKQKLALGGSAAVKITEHSWNADLYKLMGHFSWWTAFVTNVNASSSEAFLI      6982
AY278488.2        GFFTYLCGFIKQKLALGGSIAVKITEHSWNADLYKLMGHFSWWTAFVTNVNASSSEAFLI      6982
AY485277.1        GFFTYLCGFIKQKLALGGSIAVKITEHSWNADLYKLMGHFSWWTAFVTNVNASSSEAFLI      6982
AP006560.1        GFFTYLCGFIKQKLALGGSIAVKITEHSWNADLYKLMGHFSWWTAFVTNVNASSSEAFLI      6982
AP006557.1        GFFTYLCGFIKQKLALGGSIAVKITEHSWNADLYKLMGHFSWWTAFVTNVNASSSEAFLI      6982
AY274119          GFFTYLCGFIKQKLALGGSIAVKITEHSWNADLYKLMGHFSWWTAFVTNVNASSSEAFLI      6982
AY572038.1        GFFTYLCGFIKQKLALGGSIAVKITEHSWNADLYKLMGHFSWWTAFVTNVNASSSEAFLI      6982
AY572034.1        GFFTYLCGFIKQKLALGGSIAVKITEHSWNADLYKLMGHFSWWTAFVTNVNASSSEAFLI      6982
FJ588686.1        GFFTYLCGFIKQKLALGGSVAVKITEHSWNADLYKLMGHFSWWTAFVTNVNASSSEAFLI      6789
KY417145.1        GFFTYLCGFIKQKLALGGSVAVKITEHSWNADLYKLMGHFSWWTAFVTNVNASSSEAFLI      6982
KY417144.1        GFFTYLCGFIKQKLALGGSVAVKITEHSWNADLYKLMGHFSWWTAFVTNVNASSSEAFLI      6982
KY417147.1        GFFTYLCGFIKQKLALGGSVAIKITEHSWNADLYKLMGHFSWWTAFVTNVNASSSEAFLI      6982
KY417148.1        GFFTYLCGFIKQKLALGGSVAIKITEHSWNADLYKLMGHFSWWTAFVTNVNASSSEAFLI      6982
KY417143.1        GFFTYLCGFIKQKLALGGSVAVKITEHSWNADLYKLMGHFSWWTAFVTNVNASSSEAFLI      6982
KT444582.1        GFFTYLCGFIKQKLALGGSVAVKITEHSWNADLYKLMGHFSWWTAFVTNVNASSSEAFLI      6982
KC881005.1        GFFTYLCGFIKQKLALGGSVAVKITEHSWNADLYKLMGHFSWWTAFVTNVNASSSEAFLI      6982
KC881006.1        GFFTYLCGFIKQKLALGGSVAVKITEHSWNADLYKLMGHFSWWTAFVTNVNASSSEAFLI      6982
KF367457.1        GFFTYLCGFIKQKLALGGSVAVKITEHSWNADLYKLMGHFSWWTAFVTNVNASSSEAFLI      6982
KY417152.1        GFFTYLCGFIKQKLALGGSVAVKITEHSWNADLYKLMGYFSWWTAFVTNVNASSSEAFLI      6982
KY417146.1        GFFTYLCGFIKQKLALGGSVAVKITEHSWNADLYKLMGHFSWWTAFVTNVNASSSEAFLI      6982
KY417151.1        GFFTYLCGFIKQKLALGGSVAVKITEHSWNADLYKLMGYFSWWTAFVTNVNASSSEAFLI      6982
KY417142.1        GFFTYLCGFIKQKLALGGSVAIKITEHSWNADLYKLMGHFSWWTAFVTNVNASSSEAFLI      6982
                  *****:****:*:****** *:****************:*:*******************
```

```
MW532698.1      GCNYLGKPREQIDGYVMHANYIFWRNTNPIQLSSYSLFDMSKFPLKLRGTAVMSLKEGQI      7057
MT040336.1      GCNYLGKPREQIDGYVMHANYIFWRNTNPIQLSSYSLFDMSKFPLKLRGTAVMSLKEGQI      7057
MT040335.1      GCNYLGKPREQIDGYVMHANYIFWRNTNPIQLSSYSLFDMSKFPLKLRGTAVMSLKEGQI      7057
MT040334.1      GCNYLGKPREQIDGYVMHANYIFWRNTNPIQLSSYSLFDMSKFPLKLRGTAVMSLKEGQI      7057
MT040333.1      GCNYLGKPREQIDGYVMHANYIFWRNTNPIQLSSYSLFDMSKFPLKLRGTAVMSLKEGQI      7057
MN996532.2      GCNYLGKPREQIDGYVMHANYIFWRNTNPIQLSSYSLFDMSKFPLKLRGTAVMSLKEGQI      7064
MN988713.1      GCNYLGKPREQIDGYVMHANYIFWRNTNPIQLSSYSLFDMSKFPLKLRGTAVMSLKEGQI      7065
MT093571.1      GCNYLGKPREQIDGYVMHANYIFWRNTNPIQLSSYSLFDMSKFPLKLRGTAVMSLKEGQI      7065
MN996529.1      GCNYLGKPREQIDGYVMHANYIFWRNTNPIQLSSYSLFDMSKFPLKLRGTAVMSLKEGQI      7065
MT072688.1      GCNYLGKPREQIDGYVMHANYIFWRNTNPIQLSSYSLFDMSKFPLKLRGTAVMSLKEGQI      7065
NC_045512.2     GCNYLGKPREQIDGYVMHANYIFWRNTNPIQLSSYSLFDMSKFPLKLRGTAVMSLKEGQI      7065
MN994467.1      GCNYLGKPREQIDGYVMHANYIFWRNTNPIQLSSYSLFDMSKFPLKLRGTAVMSLKEGQI      7065
MG772933.1      GCNYLGKPREQIDGYVMHANYIFWRNTNPIQLSSYSLFDMSKFPLKLRGTAVMSLKDGQI      7061
MG772934.1      GCNYLGKPREQIDGYVMHANYIFWRNTNPIQLSSYSLFDMSKFPLKLRGTAVMSLKDGQI      7039
NC_014470.1     GVNYLGKQKESIDGYTMHANYIFWRNTNPIQLSSYSLFDMSKFPLKLRGTAVMSLKDNQI      7033
GQ153542.1      GVNYLGKPKEQIDGYTMHANYIFWRNTNPIQLSSYSLFDMSKFPLKLRGTAVMSLKENQI      7040
DQ022305        GVNYLGKPKEQIDGYTMHANYIFWRNTNPIQLSSYSLFDMSKFPLKLRGTAVMSLKENQI      7036
KF569996.1      GVNYLGKPKEQIDGYTMHANYIFWRNTNPIQLSSYSLFDMSKFPLKLRGTAVMSLKENQI      6979
KP886809.1      GVNYLGKPKEQIDGYTMHANYIFWRNTNPIQLSSYSLFDMSKFPLKLRGTAVMFLKENQI      7042
AY278488.2      GANYLGKPKEQIDGYTMHANYIFWRNTNPIQLSSYSLFDMSKFPLKLRGTAVMSLKENQI      7042
AY485277.1      GANYLGKPKEQIDGYTMHANYIFWRNTNPIQLSSYSLFDMSKFPLKLRGTAVMSLKENQI      7042
AP006560.1      GANYLGKPKEQIDGYTMHANYIFWRNTNPIQLSSYSLFDMSKFPLKLRGTAVMSLKENQI      7042
AP006557.1      GANYLGKPKEQIDGYTMHANYIFWRNTNPIQLSSYSLFDMSKFPLKLRGTAVMSLKENQI      7042
AY274119        GANYLGKPKEQIDGYTMHANYIFWRNTNPIQLSSYSLFDMSKFPLKLRGTAVMSLKENQI      7042
AY572038.1      GANYLGKPKEQIDGYTMHANYIFWRNTNPIQLSSYSLFDMSKFPLKLRGTAVMSLKENQI      7042
AY572034.1      GANYLGKLKEQIDGYTMHANYIFWRNTNPIQLSSYSLFDMSKFPLKLRGTAVMSLKENQI      7042
FJ588686.1      GANYLGKPKEQIDGYTMHANYIFWRNTNPIQLSSYSLFDMSKFPLKLRGTAVMSLKENQI      6849
KY417145.1      GVNYLGKPKEQIDGYTMHANYIFWRNTNPIQLSSYSLFDMSKFPLKLRGTAVMSLKENQI      7042
KY417144.1      GVNYLGKPKEQIDGYTMHANYIFWRNTNPIQLSSYSLFDMSKFPLKLRGTAVMYLKENQI      7042
KY417147.1      GANYLGKPKEQIDGYTMHANYIFWRNTNPIQLSSYSLFDMSKFPLKLRGTAVMSLKENQI      7042
KY417148.1      GANYLGKPKEQIDGYTMHANYIFWRNTNPIQLSSYSLFDMSKFPLKLRGTAVMSLKENQI      7042
KY417143.1      GANYLGKPKEQIDGYTMHANYIFWRNTNPIQLSSYSLFDMSKFPLKLRGTAVMSLKENQI      7042
KT444582.1      GANYLGKPKEQIDGYTMHANYIFWRNTNPIQLSSYSLFDMSKFPLKLRGTAVMSLKENQI      7042
KC881005.1      GVNYLGKPKEQIDGYTMHANYIFWRNTNPIQLSSYSLFDMSKFPLKLRGTAVMYLKENQI      7042
KC881006.1      GVNYLGKPKEQIDGYTMHANYIFWRNTNPIQLSSYSLFDMSKFPLKLRGTAVMYLKENQI      7042
KF367457.1      GVNYLGKPKEQIDGYTMHANYIFWRNTNPIQLSSYSLFDMSKFPLKLRGTAVMYLKENQI      7042
KY417152.1      GVNYLGKPKEQIDGYTMHANYIFWRNTNPIQLSSYSLFDMSKFPLKLRGTAVMSLKENQI      7042
KY417146.1      GANYLGKPKEQIDGYTMHANYIFWRNTNPIQLSSYSLFDMSKFPLKLRGTAVMSLKENQI      7042
KY417151.1      GVNYLGKPKEQIDGYTMHANYIFWRNTNPIQLSSYSLFDMSKFPLKLRGTAVMSLKENQI      7042
KY417142.1      GANYLGKPKEQIDGYTMHANYIFWRNTNPIQLSSYSLFDMSKFPLKLRGTAVMSLKENQI      7042
                * ***** :*.****.************************************* **:.**
```

```
MW532698.1        NDMILSLLSKGRLIIRENNKVVISSDVLVNN         7088
MT040336.1        NDMILSLLSKGRLIIRENNKVVVSSDVLVNN         7088
MT040335.1        NDMILSLLSKGRLIIRENNKVVVSSDVLVNN         7088
MT040334.1        NDMILSLLSKGRLIIRENNKVVVSSDVLVNN         7088
MT040333.1        NDMILSLLSKGRLIIRENNKVVVSSDVLVNN         7088
MN996532.2        NDMILSLLSKGRLIIRENNRVVISSDVLVNN         7095
MN988713.1        NDMILSLLSKGRLIIRENNRVVISSDVLVNN         7096
MT093571.1        NDMILSLLSKGRLIIRENNRVVISSDVLVNN         7096
MN996529.1        NDMILSLLSKGRLIIRENNRVVISSDVLVNN         7096
MT072688.1        NDMILSLLSKGRLIIRENNRVVISSDVLVNN         7096
NC_045512.2       NDMILSLLSKGRLIIRENNRVVISSDVLVNN         7096
MN994467.1        NDMILSLLSKGRLIIRENNRVVISSDVLVNN         7096
MG772933.1        NDMILSLLSKGRLIVRENNRVIISSDVLVNN         7092
MG772934.1        NDMILSLLSKGRLIVRENNRVIISSDVLVNN         7070
NC_014470.1       NDMICSLLEKGRLIIRENNKVVFSSDVLVNN         7064
GQ153542.1        NDMIYSLLEKGRLIIRENNRVVVSSDILVNN         7071
DQ022305          NDMIYSLLEKGRLIIRENNRVVVSSDILVNN         7067
KF569996.1        NEMIYSLIEKGRLIIRENNRVVVSSDILVNN         7010
KP886809.1        NDMIYSLLEKGRLIIRENNTVVVSSDVLVNH         7073
AY278488.2        NDMIYSLLEKGRLIIRENNRVVVSSDILVNN         7073
AY485277.1        NDMIYSLLEKGRLIIRENNRVVVSSDILVNN         7073
AP006560.1        NDMIYSLLEKGRLIIRENNRVVVSSDILVNN         7073
AP006557.1        NDMIYSLLEKGRLIIRENNRVVVSSDILVNN         7073
AY274119          NDMIYSLLEKGRLIIRENNRVVVSSDILVNN         7073
AY572038.1        NDMIYSLLEKGRLIIRENNRVVVSSDILVNN         7073
AY572034.1        NDMIYSLLEKGRLIIRENNRVVVSSDILVNN         7073
FJ588686.1        NDMIYSLLEKGRLIIRENNRVVVSSDVLVNN         6880
KY417145.1        NEMIYSLLENGRLIIRENNRVVVSSDVLVNN         7073
KY417144.1        NDMIYSLLEKGRLIIRESNKVVVSSDILVNI         7073
KY417147.1        NDMIYSLLEKGRLIIRENNRVVVSSDVLVNN         7073
KY417148.1        NDMIYSLLEKGRLIIRENNRVVVSSDVLVNN         7073
KY417143.1        NDMIYSLLEKGRLIIRENNRVVVSSDVLVNN         7073
KT444582.1        NDMIYSLLEKGRLIIRENNRVVVSSDILVNN         7073
KC881005.1        NDMIYSLLEKGRLIIRESNKVVVSSDILVNI         7073
KC881006.1        NDMIYSLLEKGRLIIRESNKVVVSSDILVNN         7073
KF367457.1        NDMIYSLLEKGRLIIRESNKVVVSSDILVNN         7073
KY417152.1        NDMIYSLLEKGRLIIRESNKVVVSSDILVNN         7073
KY417146.1        NDMIYSLLEKGRLIIRENNRVVVSSDILVNN         7073
KY417151.1        NDMIYSLLEKGRLIIRESNKVVVSSDILVNN         7073
KY417142.1        NDMIYSLLEKGRLIIRENNRVVVSSDVLVNN         7073
                  *:** **:.:****:**.* *:.***:***
```
